# The Synthesis and Absolute Configuration of Enantiomeric Pure (R)- and (S)-3-(piperidin-3-yl)-1H-Indole Derivatives

**DOI:** 10.3390/ijms24010517

**Published:** 2022-12-28

**Authors:** Marek Król, Grzegorz Ślifirski, Jerzy Kleps, Piotr Podsadni, Ilona Materek, Anna E. Kozioł, Franciszek Herold

**Affiliations:** 1 Department of Drug Technology and Pharmaceutical Biotechnology, Faculty of Pharmacy, Medical University of Warsaw, 1, Banacha Street, 02-097 Warsaw, Poland; 2 Faculty of Chemistry, Maria Curie-Skłodowska University, 3, M. Curie-Skłodowskiej Sq., 20-031 Lublin, Poland

**Keywords:** serotonin analogs, piperidin-3-yl-1H-indoles, enantiomers, chiral auxiliaries, HPLC separation, X-ray crystallography

## Abstract

This article describes the synthesis of new chiral 3-(piperidin-3-yl)-1H-indole derivatives **(R)-10a-c** and **(S)-11a-c** from the corresponding diastereomers: (3R, 2R) and (3S, 2R)-2-[3-(1H-indol-3-yl)-1-piperidyl]-2-phenyl-acetamides **(3R, 2R)-4a, (3R, 2R)-6b, (3R, 2R)-8c** and **(3S, 2R)-5a, (3S, 2R)-7b, (3S, 2R)-9c**. Diastereomers were obtained by N-alkylation of derivatives of racemic 3-(piperidin-3-yl)-1H-indoles **1a-c** using (S)-2-(4-toluenesulfonyloxy)-phenylacetic amide **(S)–II**. The same method was applied to obtain (3R, 2S)-methyl-2-[3-(1H-indole-3-yl)-1-piperidyl]-2-phenylacetate **(3R, 2S)-2a** and (3S, 2S)-methyl-2-[3-(1H-indole-3-yl)-1-piperidyl]-2-phenylacetate **(3S, 2S)-3a** diastereomers by treating amine **1a** with (R)-2-(4-toluenesulfonyloxy)-phenylacetic acid methylester **(R)-I**. Systematic studies via single crystal X-ray crystallography were used to determine the molecular structure of the racemates **1a-c** and the absolute configuration of the enantiomers. The solid racemates **1b** and **1c** were “true racemates” crystallizing in a centrosymmetric space group, while **1a** formed a racemic conglomerate of homoenantiomeric crystals. The absolute configuration was determined for the enantiomeric pairs **(R)-10a/(S)-11a**, **(R)-10b/(S)-11b**, and **(R)-12c/(S)-13c**, as well as for **(3S,2S)-3a**. Spectra of ^1^H, ^13^CNMR, HPLC, and HRMS for diastereomers and enantiomers were consistent with the determined structures.

## 1. Introduction

**Figure 1:**
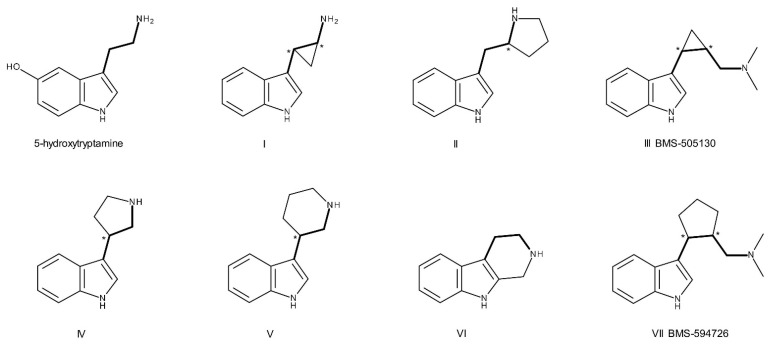
5-hydroxytryptamine and its selected rigidized analogs I–VII.

Analogs of 5-hydroxytryptamine and homotryptamine are a subject of research for new drugs that affect the central nervous system through the serotoninergic mechanism and that show multidirectional pharmacological activity [1,2,3,4]. Of particular interest are derivatives with a conformationally limited aminoethyl residue at the 3 position of the indole ring that also exhibit chiral centers in their structures, e.g., I–V, VII (Figure 1). The conformational constraints of the side chain have been shown to be an effective tool to optimize both the activity and selectivity of numerous SERT serotonin transporter protein inhibitor compounds and the 5-HT_1A_ receptor [5,6].

**Figure 2:**
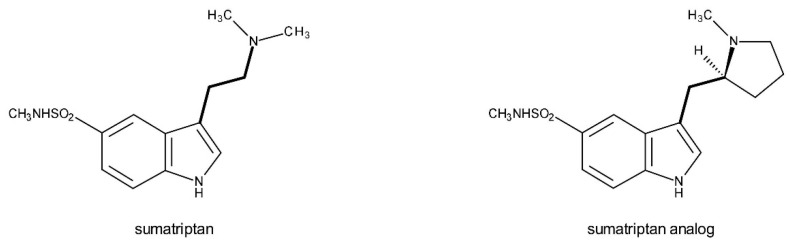
Chemical structures of sumatriptan and its analog.

The search for new and more effective drugs for the pharmacotherapy of migraine in the form of sumatriptan analogs (agonists of 5-HT_1A_ receptor) has yielded an analog with reduced conformational aminoethyl portion and an R-configuration, which has exhibited analgesic activity 10^4^ times greater than sumatriptan (Figure 2) [7].

An additional advantage of derivatives with a conformationally constrained amino-ethyl constituent, in addition to their greater biological activity, is their higher stability in the first-pass phase of metabolic biotransformation processes [7,8]. Recently, there has been a significant increase in interest in new ligands with high binding to the 5-HT_6_ receptor, due to the role of this receptor in patho-mechanisms of depression, schizophrenia, Alzheimer’s disease, and Parkinson’s disease [9]. There are particularly high expectations for these molecules due to their influence on improving cognitive processes and memory [10].

**Figure 3:**
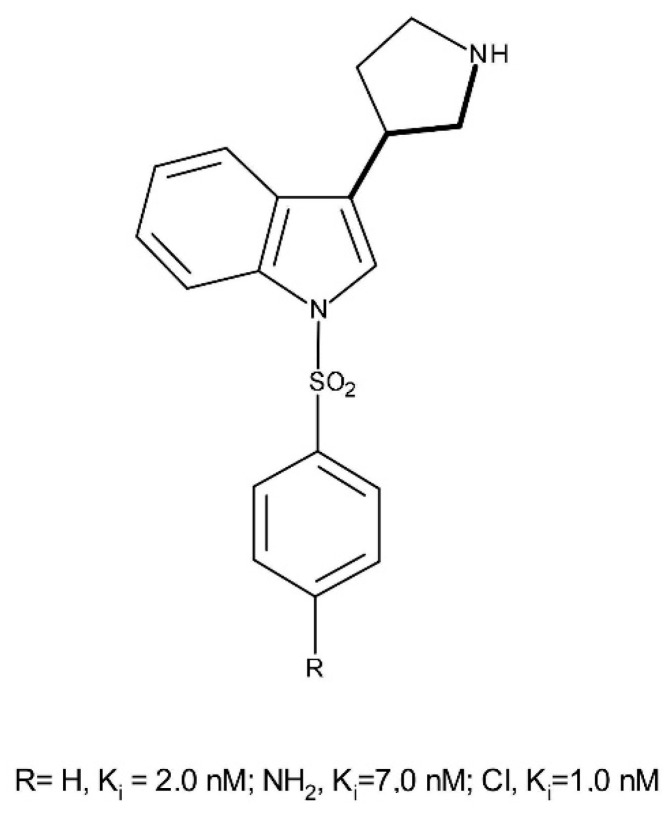
5-HT_6_ binding affinity of constrained N1-arylsulfonyltryptamine derivatives [14,15].

A number of 5-hydroxytryptamine derivatives with constrained conformation of N-arylsulfonyl constituent derivatives that show very high affinity to this receptor have been described (Figure 3) [11,12,13,14,15,16]. 

The aim of this study was to synthesize and determine the absolute configuration for a number of chiral derivatives of (R)-3-(piperidin-3-yl)-1H-indole **(R)-10a**-**c** and (S)-3-(piperidin-3-yl)-1H-indole **(S)-11a-c**. Diastereomers used as substrates for their synthesis were obtained by the N-alkylation of racemic derivatives of 3-(piperidin-3-yl)-1H-indole **1a-c** with the aid of a chiral reagent, (S)-2-(4-toluenesulfonyloxy)-phenylacetic amide **S-II**. The obtained mixture of diastereomers was chromatographically separated into analytically pure compounds: (3R,2R)-2-[3-(1H-indole-3-yl)-1-piperidyl]-2-phenylacetamide **(3R,2R)-4a**; (3R,2R)-2-[3-(5-fluoro-1H-indol-3-yl)-1-piperidyl]-2-phenylacetamide **(3R,2R)-6b**; (3R,2R)-2-[3-(5-methoxy-1H-indol-3-yl)-1-piperidyl]-2-phenylacet-amide **(3R,2R)-8c**; and the series (3S,2R)-2-[3-(1H-indole-3-yl)-1-piperidyl]-2-phenylacetamide **(3S,2R)-5a**; (3S,2R)-2-[3-(5-fluoro-1H-indol-3-yl)-1-piperidyl]-2-phenylacetamide **(3S,2R)-7b**; (3S,2R)-2-[3-(5-methoxy-1H-indol-3-yl)-1-piperidyl]-2-phenylacetamide **(3S,2R)-9c**.

The hydrogenolysis of the diastereomers obtained above led to the pure enantiomers (R)-3-(piperidin-3-yl)-1H-indole **(R)-10a**, (R)-5-fluoro-3-(piperidin-3-yl)-1H-indole **(R)-10b**, (R)-5-methoxy-3-(piperidin-3-yl)-1H-indole **(R)-10c**, and the series (S)-3-(piperidin-3-yl)-1H-indole **(S)-11a**, (S)-5-fluoro-3-(piperidin-3-yl)-1H-indole **(S)-11b**, (S)-5-methoxy-3-(piperidin-3-yl)-1H-indole **(S)-11c**.

**Figure 4:**
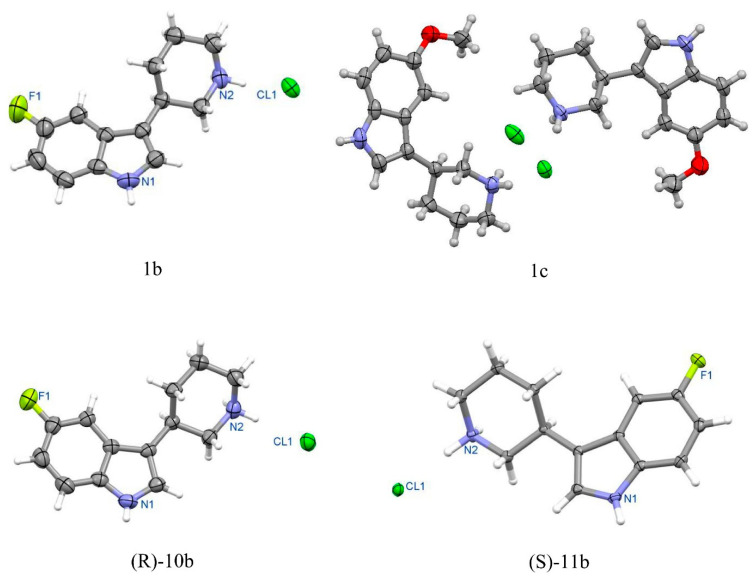
Perspective view of molecule **1b** and **1c** in racemic crystal [17,18] and view of molecular structure of enantiomeric fluoro-derivatives.

Finally, the absolute configuration was determined for representative compounds. This was preceded by a structural analysis of the solid-phase racemates **1a**-**c**. Structural X-ray studies have shown that racemate **1b** (Figure 4), as previously analyzed for **1c**, are racemic compounds (“true racemates”) crystallizing in a centrosymmetric space group [17,18].

**Figure 5:**
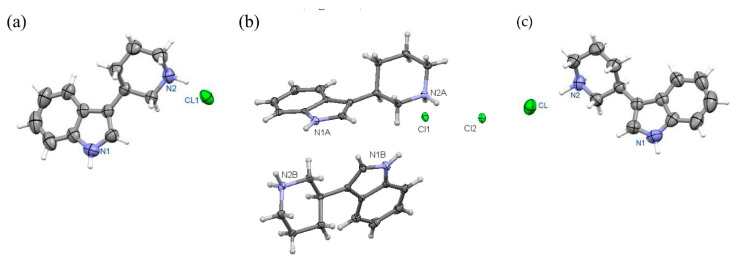
Perspective view of molecules: **(R)-10a** in orthorhombic (**a**) and monoclinic (**b**) polymorph and (**c**) molecule **(S)-11a**.

These results are in contrast to racemate **1a**, which was a racemic conglomerate; i.e., as a result of crystallization from methanol, the enantiomers spontaneously separated and produced a phase of a 1:1 mixture of homoenantiomeric crystals. Hence, compound **1a** is a mixture of the crystalline compounds **(R)-10a** and **(S)-11a**. This was confirmed during measurements performed for a dozen or so crystals from mixture **1a**. The absolute configuration was also determined for the pure enantiomers **(R)-10a** and **(S)-11a** obtained by applying the procedures described above (Figure 5).

The obtained **(R)-10a-c** and **(S)-11a-c** compounds were chiral substrates for the synthesis of ligands with double-binding to the 5-HT_1A_ receptor and the SERT transporter protein as part of the search for new SSRI+ antidepressants within our research.

## 2. Results and Discussion

### 2.1. Synthesis

**Scheme 1:**
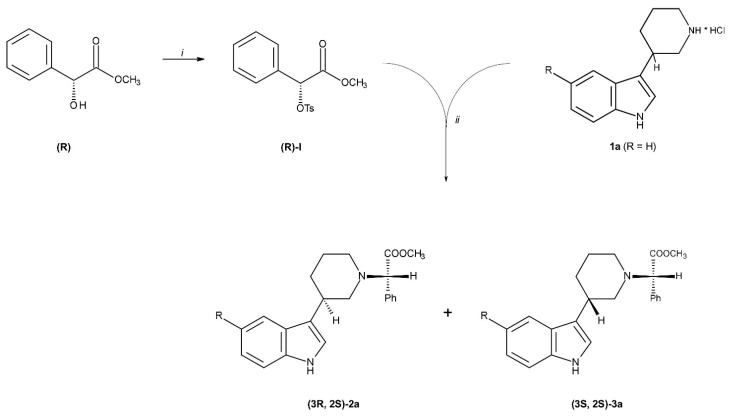
Reagents and conditions: (*i*) TsCl, Et_3_N, CH_2_Cl_2_, 0 °C; (*ii*) K_2_CO_3_, CH_3_CN, 45 °C.

**Scheme 2:**
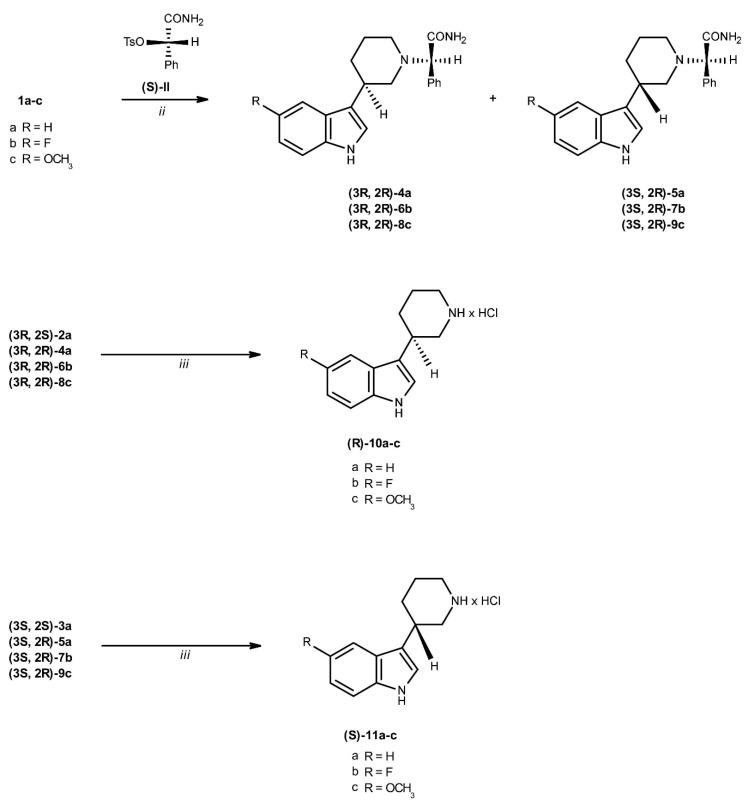
Reagents and conditions: (*ii*) K_2_CO_3_, CH_3_CN, 45 °C; (*iii*) H_2_, Pd/C 10%, MeOH, r.t. 5 atm.

The synthesis of title compounds in the form of pure enantiomers ((R)-3-(piperidin-3-yl)-1H-indole **(R)-10a**, (R)-5-fluoro-3-(piperidin-3-yl)-1H-indole **(R)-10b**, (R)-5-methoxy-3-(piperidin-3-yl)-1H-indole **(R)-10c** and (S)-3-(piperidin-3-yl)-1H-indole **(S)-11a**, (S)-5-fluoro-3-(piperidin-3-yl)-1H-indole **(S)-11b**, (S)-5-methoxy-3-(piperidin-3-yl)-1H-indole **(S)-11c**) within the study was performed according to Scheme 1 and Scheme 2.

Substrates for the synthesis were racemic 3-(piperidin-3-yl)-1H-indole **1a**, 5-fluoro-3-(piperidin-3-yl)-1H-indole **1b** and 5-methoxy-3-(piperidin-3-yl)-1H-indole **1c**, which were obtained according to the formula provided by Gharagozloo [19] and the modifications introduced by prior work [17,18]. The chiral (R)-2-(4-toluenesulfonyloxy)-phenylacetic acid methylester **(R)-I**, which is essential as a reagent for the reaction, was obtained according to the formulation described by us earlier [20]. The obtained amine **1a** was subjected to an N-alkylation reaction with a chiral **(R)-I** reagent to give a mixture of diastereomers: (3R,2S)-methyl-2-[3-(1H-indole-3-yl)-1-piperidil]-2-phenylacetate **(3R,2S)-2a** and (3S,2S)-methyl-2-[3-(1H-indole-3-yl)-1-piperidil]-2-phenylacetate **(3S,2S)-3a**. The content of diastereomers (dr) in the product obtained from the post-reaction mixture was determined by the HPLC method. Separation into analytically pure diastereomers **(3R,2S)-2a** and **(3S,2S)-3a** was carried out using a semi-preparative HPLC method. The structure and composition of both diastereomers was confirmed by ^1^H and ^13^C NMR, HRMS, and the absolute configuration of **(3S, 2S)-3a** was determined (Section 3).

To obtain a mixture of diastereomers with higher yields and stability, and which would be easier to separate, an alternative method was applied, where the chiral reagent, (S)-2-(4-toluenesulfonyloxy)-phenylacetic amide **(S)-II**, was used in the N-alkylation reaction of amines **1a-c**; this process being described elsewhere in our paper. The **(S)-II** compound has been mentioned in prior works, but the respective authors did not provide important physicochemical data, such as melting point and optical rotation [21,22,23]. The reaction of N-alkylation of amines **1a-c** with **(S)-II** led to a mixture of diastereomers in high yield, and the value of dr for these compounds was determined by HPLC method; results are given in Table 1. The obtained diastereomer mixtures were separated by flash chromatography into analytically pure compounds: **(3R,2R)-4a**, **(3R,2R)-6b**, **(3R,2R)-8c** and **(3S, 2R)-5a**, **(3S, 2R)-7b**, **(3S, 2R)-9c**. For individual analytical diastereomer samples, ^1^H, ^13^C NMR and HRMS tests were performed, as well as dr value tests by HPLC method as described in Section 3. In both reactions of the formation of diastereomers of the R- **(3R, 2S)-2a**, **(3R, 2R)-4a** series and **(3R, 2R)-6b**, **(3R, 2R)-8c** and **S-(3S, 2S)-3a, (3S, 2R)-5a, (3S, 2R)-7b**, **(3S, 2R)-9c**, a small excess of the S-series diastereomers was been observed, which was probably the result of the stereo induction of the chiral center with **(R)-1** or **(S)-2** (Table 1). 

The obtained analytically pure diastereomers of the series R—**(3R,2S)-2a**, **(3R,2R)-4a**, **(3R,2R)-6b**, **(3R,2R)-8c** and S—**(3S,2S)-3a**, **(3S,2R)-5a**, **(3S,2R)-7b**, **(3S,2R)-9c** were subjected to hydrogenation reaction to remove residues of the chiral auxiliary bonded to the nitrogen of the piperidine ring.

The chiral amines of the series **(R)-10a-c** and **(S)-11a-c** were obtained with good yields and their structure and purity were confirmed by ^1^H i ^13^C NMR, HRMS, HPLC, and X-ray structural analysis for **(R)-10a**, **(R)-10b**, **(S)-11a**, and **(S)-11b**. 

**Scheme 3:**
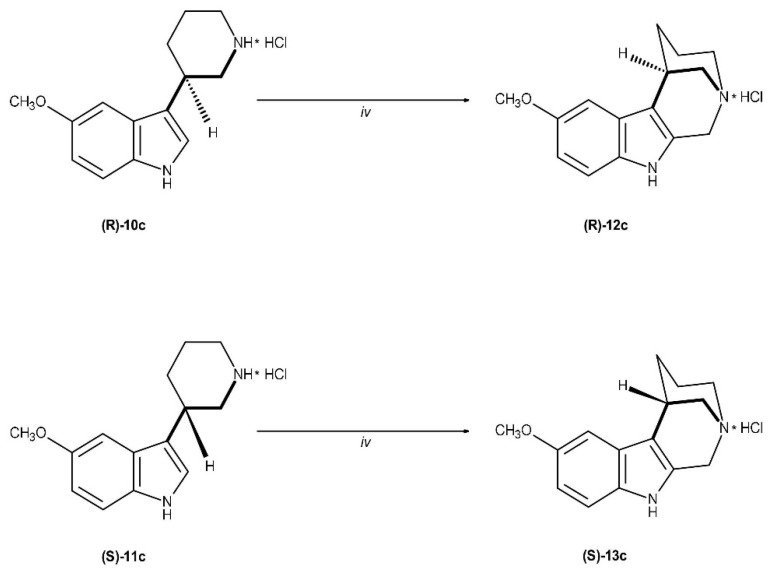
Reagents and conditions: (*iv*) MeOH/HCl g, 40 °C.

After the isolation of amine **(R)-10c** and **(S)-11c** via concentration of the methanol solution, hydrogenolysis and acidification with methanolic HCl resulted in new compounds, in addition to amines **(R)-10c** and **(S)-11c**, that were observed in the TLC test. These compounds were isolated, and their structures were proposed via ^1^H, ^13^C NMR, HRMS, and ER using HPLC (Scheme 3). X-ray crystallography showed that tetracyclic structures **(R)-12c** and **(S)-13c** were formed as a result of intramolecular cyclocondensation reactions of starting compounds. Their absolute structure was then determined (Section 3).

TLC tests related to concentration of solution before addition of HCl/MeOH did not show creation of those new structures, nor did they when a weak acid, e.g., tartaric acid, had been used for acidification. Optical rotation measurements for these compounds showed a reverse polarity rotation with respect to the starting amines **(R)-10c** and **(S)-11c**. In the literature, one can find a description of obtaining 11-substituted teracycles of this type, which were obtained by the Pictet–Spengler reaction, where racemic amine **1a** condensed with selected aldehydes was a substrate [24,25]. 

### 2.2. NMR Spectroscopy Study

Obtained spectra of ^1^H and ^13^C-NMR diastereomers of the compounds **(3R,2S)-2a, (3S,2S)-3a, (3R,2R)-4a, (3S,2R)-5a, (3R,2R)-6b, (3S,2R)-7b, (3R,2R)-8c, (3S,2R)-9c** are consistent with the determined absolute structures. Differences in chemical signal shifts and their appearance indicate stereoisomers. The biggest differences in both proton and carbon spectra were found for C_2_ and C_6_ carbons from the C_2_H_2_ and C_6_H_2_ piperidine rings, which would indicate the essential role of the nitrogen atom in the spatial configuration. 

For example, for compounds **(3R,2R)-4a** and **(3S,2R)-5a**, chemical shifts were obtained as follows: ^13^C: **(3R,2R)-4a**: C_2_ = 60.353 ppm, **(3S,2R)-5a**: C_2_ = 56.959 ppm, Δδ = 3.394; **(3R,2R)-4a**: C_6_ = 50.628 ppm, **(3S,2R)-5a**: C_6_ = 53.962 ppm, Δδ = 3.334. For other carbons, Δδ did not exceed 0.03 ppm. 

We found the following for protons ^1^H: **(3R,2R)-4a**: C_2_H_ax_ = 2.255, **(3S,2R)-5a**: C_2_H_ax_ = 1.70–1.92, average 1.81, Δδ ~0.45, **(3R,2R)-4a**: C_2_H_eq_ = 3.339 ppm, **(3S,2R)-5a**: C_2_H_eq_ = 3.085 ppm, Δδ ~0.25, **(3R,2R)-4a**: C_6_H_ax_ = 1.848 ppm, **(3S, 2R)-5a**: C_6_H_ax_ = 2.249 ppm, Δδ ~0.40, **(3R, 2R)-4a**: C_6_H_eq_ = 2.783 ppm, **(3S, 2R)-5a**: C_6_H_eq_ = 3.085 ppm, Δδ ~0.30. 

For other protons, Δδ did not exceed 0.1 ppm.

A similar dependency was found for diastereomer ester derivatives **(3R, 2S)-2a** and **(3S, 2S)-3a**. For example, the chemical shifts for carbons at the 2- and 6-piperidine positions were as follows: ^13^C: **(3R, 2S)-2a**: C_2_ = 58.050 ppm, **(3S, 2S)-3a**: C_2_ = 58.941 ppm, Δδ = 0.891, **(3R, 2S)-2a**: C_6_ = 52.167 ppm, **(3S, 2S)-3a**: C_6_ = 51.615 ppm, Δδ = 0.552; for other carbons, Δδ did not exceed 0.19 ppm. 

We found the following for protons ^1^H: **(3R, 2S)-2a**: C_2_H_ax_ = 2.057 ppm, **(3S, 2S)-3a**: C_2_H_ax_ = 2.256 ppm, Δδ = 0.2, **(3R, 2S)-2a**: C_2_H_eq_ = 3.086 ppm, **(3S, 2S)-3a**: C_2_H_eq_~3.27 ppm, Δδ~0.2, **(3R, 2S)-2a**: C_6_H_ax_ = 2.273 ppm, **(3S, 2S)-3a**: C_6_H_ax_ = 1.978 ppm, Δδ = 0.3, **(3R, 2S)-2a**: C_6_H_ex_ = 2.928 ppm, **(3S, 2S)-3a**: C_6_H_eq_ = 2.828 ppm, Δδ = 0.1. 

For other protons, Δδ did not exceed 0.045 ppm (except for C4”H, where Δδ = 0.1).

The configuration of the diastereomeric molecule was similar to the previous structures.

In turn, the proton and carbon spectra of amine hydrochlorides **(R)-10a** and **(S)-11a**, **(R)-10b** and **(S)-11b**, **(R)-10c** and **(S)-11c**, respectively, had the same spectra of enantiomers identical with the spectra of racemic amine hydrochlorides **1a**, **1b**, **1c**.

In turn, the proton and carbon spectra for the **(R)-12c** and **(S)-13c** enantiomers were the same and confirmed their structure.

## 3. Materials and Methods

### 3.1. General Remarks

Melting points were determined on an Electrothermal iA9200 apparatus with open capillary tubes and are uncorrected. The hydrogenolysis reactions were carried out in a Roth autoclave type 50 S. TLC was performed on 0.25 mm E. Merck silica gel 60 F 254 plates and visualized under UV light (λ = 254 nM) or by staining with *p*-chloranil ethylacetate solution. Flash chromatography was performed on E Merck 250–400 mesh silica gel 60. The HPLC analyses were performed on a Shimadzu Prominence Preparative Liquid Chromatograph under control of CBM 20A, UV–VIS detector SPD-20A, binary pump LC-20AP, and Fraction Collector FRC-10A apparatus. Chiral HPLC was performed on chiralpak IA 4.6 × 250 mm (Daicel Chemical Industries LTD, Osaka, Japan) column with 225 nm detector. 

The NMR spectra were recorded on Varian Unity Plus 500 MHz using CDCl_3_, D_2_O or CD_3_OD as solvents. NMR data are reported as follows: chemical shift (δ) (parts per million, ppm relative to tetramethylsilane used as the internal references), multiplicity; s (singlet), d (doublet), t (triplet), q (quartet), and br (broad); coupling constants (J) are given in Hertz (Hz). All NMR spectra can be found in Supplementary Materials. The HRMS spectra were obtained on a Thermo Q-Exactive mass spectrometer. The optical rotation was performed on a Perkin-Elmer 241 polarimeter at 20 °C. (R)-(-) methyl mandelate and (S)-(+)-mandelate amides were high-grade commercial products purchased from Aldrich and used without further purification.

### 3.2. X-ray Crystallography

The single crystal X-ray diffraction data were collected at either 293 K or 120 K on a SuperNova diffractometer with CuKα radiation. Some crystals disintegrated on cooling; therefore, their structural analysis was based on measurements at room temperature. The exception was crystal **10a**, which underwent a phase transition to give a second polymorph at low temperature. Cell refinement and data collection as well as data reduction and analysis were performed with the CrysAlisPro [26]. Structures were solved with the use of SHELXS program and refined with the SHELXL−2018/3 [27]. All non-hydrogen atoms were refined with anisotropic displacement parameters. The hydrogen atoms were positioned either on the electron difference maps or were calculated from the geometry at idealized positions, depending on the quality of the crystal and diffraction data. The absolute configuration was determined using the Flack method [28,29]. The experimental details and final atomic parameters for all crystal structures have been deposited with the Cambridge Crystallographic Data Centre as supplementary material (ID CCDC No.: 2085100-2085108). 

### 3.3. Synthesis of Compounds

#### 3.3.1. S-(+)-2-(4-toluenesulfonyloxy)-phenylacetic amide **(S)-II**

To a stirred, cooled (−15 °C) solution of 4-toluenesulfonyl chloride (2.5 g, 13.2 mmol) in dry dichloromethane (20 mL), (S)-(+)-mandelamide (0.5 g, 3.3 mmol) and triethylamine (0.46 mL, 3.3 mmol) were added in one portion. The solution was stirred at −5 to +5 °C for 5 h. The mixture was then filtered and concentrated under reduced pressure. The crude product was purified by flash chromatography (dichloromethane/methanol 98:2, then dichloromethane/ethylacetate 98:2, *v*/*v*) to yield the title compound. Yield: 70.6%, M.P. 143.2–143.7 °C, [α] = +29.99°.

^1^H NMR (500 MHz, CDCl_3_): δ 7.65 (C2′H,C6′H, [2H], d, ^3^J = 8.0), 7.2–7.3 (C2H-C6H,C3′H,C5′H, [7H], m), 6.50 (N8H(2), [1H], bs), 6.10 (N8H(1), [1H], bs), 5.72 (C7H, [1H], s), 2.39 (C7′H_3_, [3H], s).

^13^C NMR (125 MHz, CDCl_3_): δ 169.6 (C9, s), 145.4 (C4′, s), 133.6 (C1, s), 132.9 (C1′, s), 129.8 (C3′,C5′, s), 129.4 (C4, s), 128.7 (C3, C5, s), 127.9 (C2′, C6′, s), 127.5 (C2, C6, s), 81.0 (C7, s), 21.6 (C7′, s).

ESI-HRMS *m*/*z*: Calcd for C_15_H_16_NO_4_S [M+H]^+^ 306.08000. Found: 306.07950

#### 3.3.2. General Procedure for the Synthesis of Diasteromers **(3R,2S)-2a**, **(3S,2S)-3a**, **(3R,2R)-4a**, **(3S,2R)-5a**, **(3R,2R)-6b**, **(3S,2R)-7b**, **(3R,2R)-8c** and **(3S,2R)-9c**

**Figure 6:**
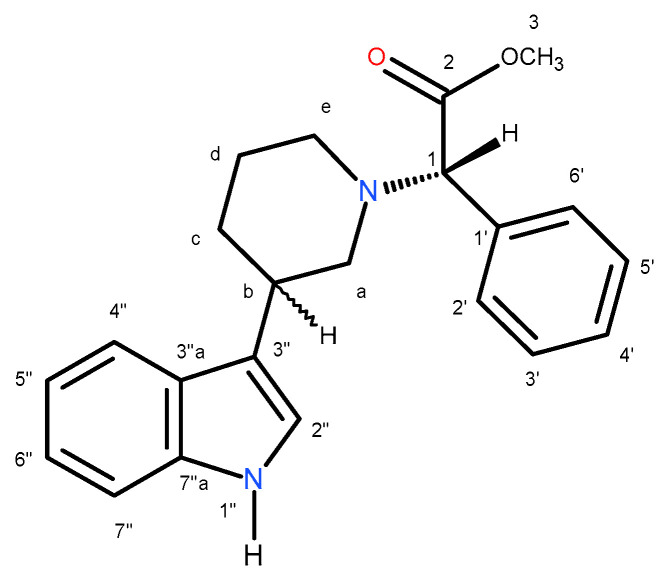
Numbering system for NMR spectra interpretation of compounds **(3R,2S)-2a** and **(3S, 2S)-3a**.

A suitable 3- (piperidin-3-yl) -1H-indole **1a-c** (0.01 m) derivative, chiral reagent **(R)-I** or **(S)-II** (0.01 m), and potassium carbonate (0.011 m) were added to 100 mL of acetonitrile. The mixture was stirred at 45 °C; reaction times are given in Table 2. The post-reaction mixture was concentrated dry at 45 °C under vacuum. The residue was pre-purified on a silica gel column using an eluent CH_2_Cl_2_/MeOH/TEA 98:2:0.1. A mixture of **(3R,2S)-2a** and **(3S,2S)-3a** diastereomers was separated by semipreparative HPLC. The remaining diastereomers were separated on a silica gel column using 50:50 acetone/cyclohexane eluent. Table 1 lists dr for mixtures; in Table 2, m.p., [α] and dr for diastereomers are given. Numbering system, which was used in NMR spectra interpretation of compounds **(3R,2S)-2a** and **(3S, 2S)-3a** is shown in Figure 6.

##### Methyl (2S)-2-[(3R)-3-(1H-indol-3-yl)piperidin-1-yl-2-phenylacetate **(3R,2S)-2a**

^1^H NMR (500 MHz, CDCl_3_): δ 8.00 (NH, [1H], bs), 7.52 (C4″H, [1H], dd, ^3^J = 8.0, ^4^J = 1.0), 7.45 (C2′H,C6′H, [2H], dt, ^3^J = 7.0, ^4^J = 1.5), 7.22–7.33 (C3′H,C4′H,C5′H,C7″H, [4H], m), 7.13 (C6″H, [1H], m, ^3^J_1_ = 8.0, ^3^J_2_ = 7.0, ^4^J = 1.0), 7.05 (C5″H, [1H], m, ^3^J_1_ = 8.0, ^3^J_2_ = 7.0, ^4^J = 1.0), 6.98 (C2″H, [1H], bs), 410 (C1H, [1H], s), 3.68 (OCH_3_, [3H], s), 3.22 (CbH(A), [1H], tt, ^3^J_A-A_ = 11.0, ^3^J_A-E_ = 3.5), 3.09 (CaH(E),[1H], m), 2.93 (CeH(E), [1H], pd), 2.27 (CeH(A), [1H], td), 2.06 (CaH(A), CcH(E), [2H], m), 1.73–1.90 (CdH_2_, [2H], m) 1.55 (CcH(A), [1H], m).

^13^C NMR (125 MHz, CDCl_3_): δ 172.4 (C2, s), 136.1 (C7″a, s), 136.1 (C1′, s), 128.8 (C2′,C6′, s), 128.2 (C3′,C5′, s), 128.2 (C4′, s), 126.7 (C3″a, s), 121.8 (C6″, s), 120.4 (C2″, s), 119.5 (C3″, s), 119.1 (C5″, s), 119.0 (C4″, s), 111.1 (C7″, s),74.5 (C1, s), 58.1 (Ca, s), 52.2 (Ce, s), 52.0 (OC3H_3_, s), 33.8 (Cb, s), 31.1 (Cc, s), 25.4 (Cd, s).

ESI-HRMS *m*/*z*: Calcd for C_22_H_25_N_2_O_2_ [M+H]^+^ 349.19160. Found: 349.19136 

##### Methyl (2S)-2-[(3S)-3-(1H-indol-3-yl)piperidin-1-yl]-2-phenylacetate **(3S,2S)-3a** (Figure 7)

^1^H NMR (500 MHz, CDCl_3_): δ 8.00 (NH, [1H], bs), 7.63 (C4″H, [1H], d, ^3^J = 8.0), 7.46 (C2′H,C6′H, [2H], dt, ^3^J = 8.0, ^4^J = 2.0), 7.28–7.38 (C3′H, C4′H, C5′H, C7″H, [4H], m), 7.17 (C6″H, [1H], m, ^3^J_1_ = 8.5, ^3^J_2_ = 7.0, ^4^J = 1.0), 7.09 (C5″H, [1H], m, ^3^J_1_ = 7.5, ^3^J_2_ = 7.0, ^4^J = 1.0), 7.00 (C2″H, [1H], d, ^3^J = 2.5), 4.09 (C1H, [1H], s), 3.66 (OCH_3_, [3H], s), 3.22–3.32 (CbH (A), CaH(E), [2H], m, ^3^J_A-A_ = 11.0), 2.83 (CeH(E), [1H], pd), 2.26 (CaH(A), [1H], t, ^2^J = ^3^J_A-A_ = 10.5), 2.07 (CcH(E), [1H], m), 1.98 (CeH(A), [1H], td), 1.68–1.86 (CdH(A),CdH(E), [2H], m), 1.53 (CcH(A), [1H], m).

^13^C NMR (125 MHz, CDCl_3_): δ 172.4 (C2, s), 136.2 (C7″a, s), 136.1 (C1′, s), 128.9 (C2′,C6′, s), 128.5 (C3′,C5′, s), 128.2 (C4′, s), 126.7 (C3″a, s), 121.9 (C6″, s), 120.3 (C2″, s), 119.6 (C3″, s), 119.2 (C5″, s), 119.2 (C4″, s), 111.1 (C7″, s), 74.6 (C1, s), 58.9 (Ca, s), 52.0 (OCH_3_, s), 51.6 (Ce, s), 33.9 (Cb, s), 31.3 (Cc, s), 25.5 (Cd, s).

ESI-HRMS *m*/*z*: Calcd for C_22_H_25_N_2_O_2_ [M+H]^+^ 349.19160. Found: 349.19136

Crystal data for **(3S,2S)-3a**. Formula C_22_H_24_N_2_O_2_; M_w_ 348.43. Crystal system orthorhombic, space group *P*2_1_2_1_2_1_, unit cell dimensions a = 9.023(2) Å, b = 11.615(3) Å, c = 18.937(4) Å, V = 1984.6(8) Å^3^, Z = 4, D_calc_ = 1.166 g/cm^3^, μ = 0.594 mm^−1^, F(000) = 744. θ range 4.66 to 73.946°; reflections collected/independent 7902/3718 [R(int) = 0.0203]. Goodness-of-fit on F^2^ 1.046; final R indices [I > 2σ(I)]: R1 = 0.0356, wR2 = 0.0866; R indices (all data) R1 = 0.0422, wR2 = 0.0950, GOOF = 1.046. CCDC No 2085108.

**Figure 7:**
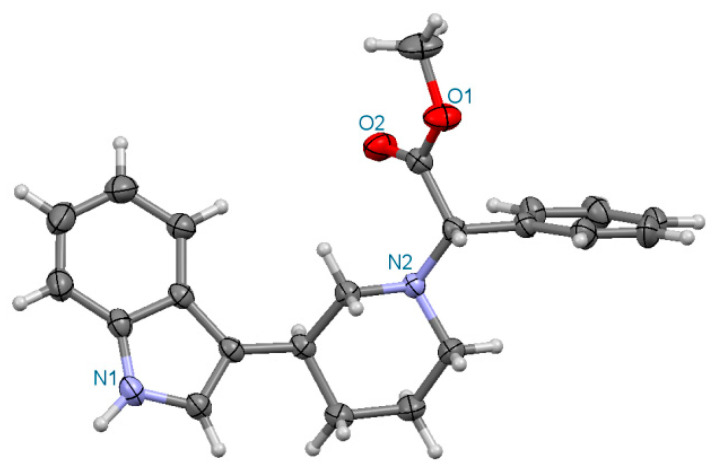
Perspective drawing of the molecule **(3S,2S)-3a**.

**Figure 8:**
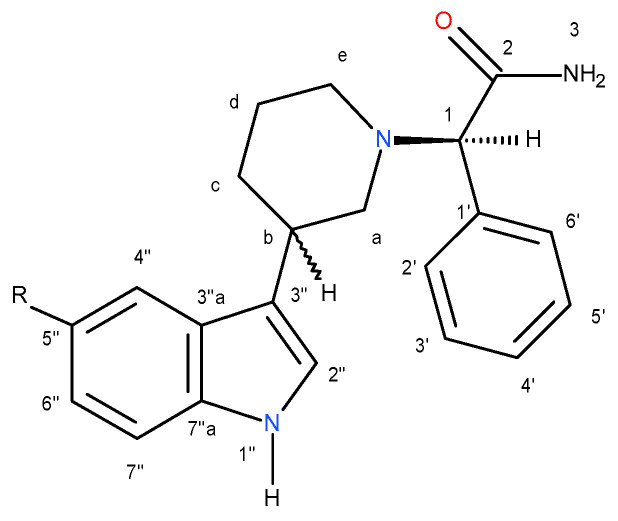
Numbering system for NMR spectra interpretation of compounds **(3R,2R)-4a**, **(3S,2R)**-**5a**, **(3R,2R)**-**6b**, **(3S,2R)**-**7b**, **(3R,2R)**-**8c** and **(3S,2R)**-**9c**.

Numbering system, which was used in NMR spectra interpretation of compounds **(3R,2R)-4a**, **(3S,2R)**-**5a**, **(3R,2R)**-**6b**, **(3S,2R)**-**7b**, **(3R,2R)**-**8c** and **(3S,2R)**-**9c** is shown in Figure 8.

##### (2R)-2-[(3R)-3-(1H-indol-3-yl)piperidin-1-yl]-2-phenylacetamide **(3R,2R)-4a**

^1^H NMR (500 MHz, CDCl_3_): δ 8.11 (N1″H, [1H], bs), 7.59 (C4″H, [1H], 4d, ^3^J = 8.0), 7.28–7.36 (C2′-6′H,C7″H, [6H], m), 7.21 (N3H(2), [1H], d, ^2^J = 4.5), 7.17 (C6″H, [1H], m, ^3^J_1_ = 8.0, ^3^J_2_ = 7.0, ^4^J = 1.0), 7.09 (C5″H, [1H], m, ^3^J_1_ = 7.5, ^3^J_2_ = 7.0, ^4^J = 1.0), 6.91 (C2″H, [1H], d, ^3^J = 2.0), 5.83 (N3H(1), [1H], d, ^2^J = 4.0), 3.97 (C1H, [1H], s), 3.34 (CaH(E), [1H], pd), 3.20 (CbH, [1H], tt, ^3^J_A-A_ = 11.0, ^3^J_A-E_ = 5.0), 2.78 (CeH(E), [1H], pd), 2.26 (CaH(A), [1H], t, ^2^J = ^3^J_A-A_ = 11.0), 2.09 (CcH(E), [1H], m), 1.85 (CeH(A), [1H], td, ^2^J = ^3^J_A-A_ = 11.0, ^3^J_A-E_ = 3.0), 1.66–1.77 (CdH(E),CdH(A), [2H], m), 1.47 (CcH(A), [1H], kd, ^2^J = ^3^J_A-A_ = 11.0, ^3^J_A-E_ = 5.0). 

^13^C-NMR (125 MHz, CDCl_3_): δ 174.8 (C2, s), 136.2 (C7″a, s), 135.3 (C1′, s), 129.2 (C2′,C6′, s), 128.4 (C3′,C5′, s), 128.1 (C4′, s), 126.6 (C3″a, s), 122.0 (C6″, s), 120.1 (C2″, s), 119.2 (C5″, s), 119.0 (C3″, s), 118.9 (C4″, s), 111.3 (C7″, s), 75.9 (C1, s), 60.4 (Ca, s), 50.6 (Ce, s), 34.4 (Cb, s), 31.1 (Cc, s), 25.9 (Cd, s). 

ESI-HRMS *m*/*z*: Calcd for C_21_H_24_N_3_O [M+H]^+^ 334.19193. Found: 334.19148

##### (2R)-2-[(3S)-3-(1H-indol-3-yl)piperidin-1-yl]-2-phenylacetamide **(3S,2R)-5a**

^1^H-NMR (500 MHz, CDCl_3_): δ 8.05 (N1″H, [1H], bs), 7.50 (C4″H, [1H], d, ^3^J = 8.0), 7.26–7.35 (C2′H,C3′H,C5′H,C6′H,C7″H, [5H], m), 7.23 (C4′H, [1H], tt, ^3^J = 7.0, ^4^J = 1.5), ~7,14 (N3H(2), [1H] bs*), 7.14 (C6″H, [1H], m, ^3^J_1_ = 8.0, ^3^J_2_ = 7.0, ^4^J = 1.0), 7.05 (C5″H, [1H], m, ^3^J_1_ = 7.5, ^3^J_2_ = 7.5, ^4^J = 1.0), 6.83 (C2″H, [1H], d, ^3^J = 2.0), 5.87 (N3H(1), [1H], d, ^2^J = 2.5), 3.95 (C1H, [1H], s), 3.09 (CaH(E), CbH, CeH(E), [3H], m), 2.25 (CeH(A), [1H], m), 2.08 (CcH(E), [1H], m), 1.70–1.92 (CaH(A), CdH(E), CdH(A), [3H], m), 1.47 (CcH(A), [1H], 4d, ^2^J = ^3^J_A-A_ = 11.5, ^3^J_A-E_ = 5.5). 

^13^C-NMR (125 MHz, CDCl_3_): δ 174.9 (C2, s), 136.1 (C7″a, s), 135.3 (C1′, s), 129.0 (C2′,C6′, s), 128.5 (C3′, C5′, s), 128.1 (C4′, s), 126.6 (C3″a, s), 121.9 (C6″, s), 120.0 (C2″, s), 119.2 (C3″, s), 119.2 (C5″, s), 118.8 (C4″, s), 111.2 (C7″, s), 76.1 (C1, s), 57.0 (Ca, s), 54.0 (Ce, s), 34.4 (Cb, s), 31.0 (Cc, s), 26.1 (Cd, s).

ESI-HRMS *m*/*z*: Calcd for C_21_H_24_N_3_O [M+H]^+^ 334.19193. Found: 334.19147

##### (2R)-2-[(3R)-3-(5-fluoro-1H-indol-3-yl)piperidin-1-yl]-2-phenylacetamide **(3R,2R)-6b**

^1^H-NMR (500 MHz, CDCl_3_): δ 8.13 (N1″H, [1H], bs), 7.28–7.38 (C2′-6′H, [5H], m), 7.24 (C7″H, [1H], dd, ^3^J = 8.5, ^4^J_H-F_ = 4.5), 7.20 (C4″H, [1H], dd, ^3^J_H-F_ = 9.5, ^4^J = 2.5), ~7.18 (N3H(2), [1H], bs), 6.97 (C2″H, [1H], d, ^3^J = 2.5), 6.92 (C6″H, [1H], td, ^3^J = 9.0, ^4^J = 2.5), 5.72 (N3H(1), [1H], d ^3^J = 4.5), 3.97 (C1H, [1H], s), 3.30 (CaH(E), [1H], pd), 3.12 (CbH, [1H], tt, ^3^J_A-A_ = 11.5, ^3^J_A-E_ = 3.5), 2.79 (CeH(E), [1H], pd), 2.24 (CaH(A), [1H], t, ^2^J = ^3^J_A-A_ = 11.5), 2.07 (CcH(E), [1H], m), 1.86 (CeH(A), [1H], td, ^2^J = ^3^J_A-A_ = 11.0, ^3^J_A-E_ = 3.0), 1.66–1.78 (CdH_2_, [2H], m), 1.43 (CcH(A), [1H], kd, ^2^J = ^3^J_A-A_ = 11.5, ^3^J_A-E_ = 5.0).

^13^C-NMR (125 MHz, CDCl_3_): δ 174.6 (C2, s), 157.6 (C5″, d, ^1^J = 234.5), 135.3 (C1′, s), 132.7 (C7″a, s), 129.2 (C2′, C6′, s), 128.5 (C3′, C5′, s), 128.2 (C4′, s), 127.0 (C3″a, d, ^3^J = 9.6), 121.9 (C2″, s), 119.2 (C3″, d, ^4^J = 4.6), 111.9 (C7″, d, ^3^J = 9.6), 110.4 (C6″, d, ^2^J = 26.4), 103.8 (C4″, d, ^2^J = 23.4), 75.9 (C1, s), 60.1 (Ca, s), 50.7 (Ce, s), 34.4 (Cb, s), 31.1 (Cc,s), 25.8 (Cd, s). 

ESI-HRMS *m*/*z*: Calcd for C_21_H_23_FN_3_O [M+H]^+^ 352.18251. Found: 352.18202

##### (2R)-2-[(3S)-3-(5-fluoro-1H-indol-3-yl)piperidin-1-yl]-2-phenylacetamide **(3S, 2R)-7b**

^1^H-NMR (500 MHz, CDCl_3_): δ 8.07 (N1″H, [1H], bs), 7.28–7.35 (C2′, 3′, 5′, 6′H, [4H], m), 7.25 (C4′H, [1H], tt, ^3^J = 8.5), 7.19 (C7″H, [1H], dd, ^3^J = 9.0, ^4^J_H-F_ = 4.5), ~7.12 (N3H(2), [1H], bs), 7.11 (C4″H, [1H], dd, ^3^J_H-F_ = 9.5, ^4^J = 2.5), 6.88 (C2″H, C6″H, [2H], m), 5.75 (N3H(1), [1H], d, ^3^J = 3.0), 3.94 (C1H, [1H], s), 3.09 (CaH(E), [1H], pd), 2.98–3.07 (CbF, CeH(E), [2H], m), 2.24 (CeH(A), [1H], td, ^2^J = ^3^J_A-A_ = 11.5, ^3^J_A-E_ = 3.5), 2.07 (CcH(E), [1H], m), 1.76–1.90 (CaH(A), CdH_2_, [3H], m), 1.46 (CcH(A), [1H], kd, ^2^J = ^3^J_A-A_ = 11.5, ^3^J_A-E_ = 5.0).

^13^C-NMR (125 MHz, CDCl_3_): δ 174.8 (C2, s), 157.5 (C5″, d, ^1^J = 234.3), 135.4 (C1′, s), 132.6 (C7″a, s), 128.9 (C2′, C6′, s), 128.5 (C3′, C5′, s), 128.2 (C4′, s), 126.9 (C3″a, d, ^3^J = 9.6), 121.9 (C2″, s), 119.3 (C3″, d, ^4^J = 4.8), 111.8 (C7″, d, ^3^J = 9.8), 110.3 (C6″, d, ^2^J = 26.3), 103.7 (C4″, d, ^2^J = 23.5), 76.1 (C1, s), 56.8 (Ca, s), 54.0 (Ce, s), 34.4 (Cb, s), 31.0 (Cc, s), 26.1 (Cd, s). 

ESI-HRMS *m*/*z*: Calcd for C_21_H_23_FN_3_O [M+H]^+^ 352.18251. Found: 352.18197

##### (2R)-2-[(3R)-3-(5-methoxy-1H-indol-3-yl)piperidin-1-yl]-2-phenylacetamide **(3R,2R)-8c**

^1^H-NMR (500 MHz, CDCl_3_): δ 7.98 (N1″H, [1H], bs), 7.29–7.39 (C2′-6′H, [5H], m), 7.24 (C7″H, [1H], d, ^3^J = 9.0), 7.19 (N3H(2), [1H], bs), 7.04 (C4″H, [1H], d, ^4^J = 2.5), 6.93 (C2″H, [1H], d, ^3^J = 2.5), 6.86 (C6″H, [1H], dd, ^3^J = 9.0, ^4^J = 2.5), 5.75 (N3H(1), [1H], d, ^2^J = 4.0), 3.97 (C1H, [1H], s), 3.86 (OCH_3_, [3H], s), 3.53 (CaH(E), [1H], pd), 3.15 (CbH, [1H], tt, ^2^J = ^3^J_A-A_ = 15.0), 2.79 (CeH(E), [1H], pd), 2.25 (CaH(A), [1H], t, ^2^J = ^3^J_A-A_ = 11.0), 2.09 (CcH(E), [1H],m), 1.85 (CeH(A), [1H], m), 1.65–1.80 (CdH, [2H], m), 1.44 (CcH(A), [1H], kd, ^2^J = ^3^J_A-A_ = 11.5, ^3^J_A-E_ = 5.0).

^13^C-NMR (125 MHz, CDCl_3_): δ 174.5 (C2, s), 153.9 (C5″, s), 135.3 (C1′, s), 131.4 (C7″a, s), 129.2 (C2′, C6′, s), 128.5 (C3′, C5′, s), 128.1 (C4′, s), 127.0 (C3″a, s), 120.9 (C2″, s), 119.0 (C3″, s), 112.1 (C7″, s), 112.0 (C6″, s), 101.1 (C4″, s), 75.9 (C1, s), 60.2 (Ca, s), 56.1 (OCH_3_, s), 50.7 (Ce, s), 34.5 (Cb, s), 31.1 (Cc, s), 26.0 (Cd, s). 

ESI-HRMS *m*/*z*: Calcd for C_22_H_26_N_3_O_2_ [M+H]^+^ 364.20250. Found: 364.20180

##### (2R)-2-[(3S)-3-(5-methoxy-1H-indol-3-yl)piperidin-1-yl]-2-phenylacetamide **(3S,2R)-9c**

^1^H-NMR(500 MHz, CDCl_3_): δ 7.84 (N1″H, [1H], bs), 7.36 (C2′H, C6′H, [2H], dt, ^3^J = 7.0, ^4^J = 1.5), 7.30 (C3′H, C5′H, [2H], tt, ^3^J = 7.0, ^4^J = 1.5), 7.26 (C4′H, [1H], tt, ^3^J = 7.0, ^4^J = 1.5), 7.19 (C7″H, [1H], dd, ^3^J = 8.5, ^5^J = 0.5), 7.09 (N3H(2), [1H], bs), 6.89 (C4″H, [1H], d, ^4^J = 2.5), 6.85 (C2′H, [1H], d, ^3^J = 2.5), 6.81 (C6″H, [1H], dd, ^3^J = 8.5, ^4^J = 2.5), 5.55 (N3H(1), [1H], bs), 3.93 (C1H, [1H], s), 3.81 (OCH_3_), [3H], s), 3.03–3.16 (CaH(E),CeH(E),CbH, [3H], m), 2.24 (CeH(A), [1H], td, ^2^J = ^3^J_A-A_ = 11.0, ^3^J_A-E_ = 3.5), 2.06–2.14 (Cc(E), [1H], m), 1.78–1.91 (CaH(A), CdH_2_, [3H], m), 1.50 (CcH(A), [1H], kd, ^2^J = ^3^J_A-A_ = 11.5, ^3^J_A-E_ = 5.0).

^13^C-NMR (125 MHz, CDCl_3_): δ 175.0 (C2, s), 153.7 (C5″, s), 135.6 (C1′, s), 131.2 (C7″a, s), 128.9 (C2′, C6′, s), 128.5 (C3′, C5′, s), 128.1 (C4′, s), 126.9 (C3″a, s), 120.9 (C2″, s), 118.7 (C3″, s), 112.2 (C7″, s), 111.9 (C6″, s), 100.6 (C4″, s), 76.2 (C1, s), 57.0 (Ca, s), 55.9 (OCH_3_, s), 54.0 (Ce, s), 34.3 (Cb, s), 30.8 (Cc, s), 26.0 (Cd, s). 

ESI-HRMS *m*/*z*: Calcd for C_22_H_26_N_3_O_2_ [M+H]^+^ 364.20250. Found: 364.20188

#### 3.3.3. General Procedure for the Synthesis of Chiral (R) and (S) 3-(piperidin-3-yl) -1H-indol derivatives **(R)-10a-c** and **(S)-11a-c**

**Figure 9:**
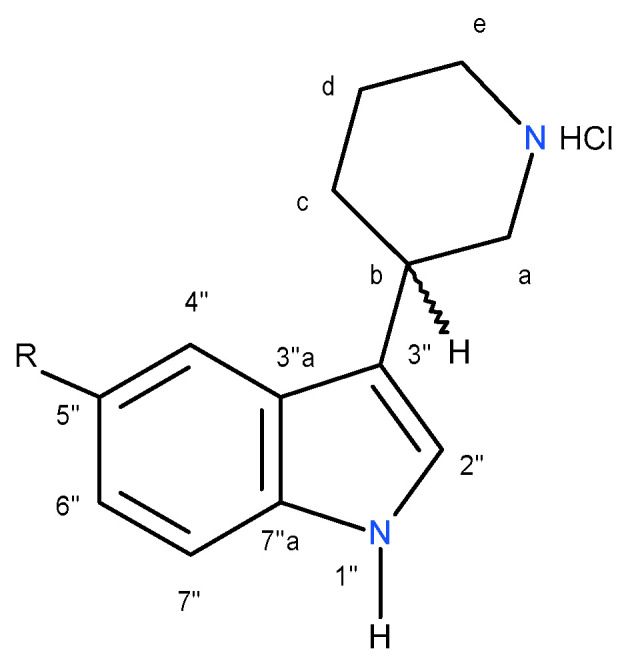
Numbering system for NMR spectra interpretation of compounds **(R)-10a-c** and **(S)-11a-c**.

An ampoule with the appropriate diastereomer (0.014m), Pd/C 10% 0.3 g catalyst, and 180 mL methanol were placed in the autoclave. The hydrogenolysis process was carried out at 30 °C under the pressure of 1–3 atm. The mixture was blended for 8 h. The catalyst was filtered off from the postreaction mixture and the filtrate was cooled down; after that, it was acidified with a methanolic HCl solution to a pH of about 2 and concentrated at 45 °C under vacuum until dry. After concentrating solutions of **(R)-10a or (S)-11a** compounds to dryness, 5 mL of absolute EtOH and Et2O were added to the remaining part to obtain a cloudy structure in the solutions, which were then placed in a refrigerator. As for the **(R)-10-b, (S)-11-b** enantiomers, after concentration to dryness, 10 mL of acetone was added to the residue, which was then placed in the refrigerator. In the case of **(R)-10c** and **(S)-11c** compounds, the solutions were concentrated to a volume of 10 mL and placed in a refrigerator, and the isolated crystals **(R)-12-c** or **(S)-13c** were filtered off. Then, 20 mL of acetone was added to the filtrate and placed in a refrigerator to obtain salt **(R)-10c** and **(S)-11c**. The reaction yield, melting temp, er, and [α] are given in Table 3. Numbering system, which was used in NMR spectra interpretation of compounds **(R)-10a-c** and **(S)-11a-c** is shown in Figure 9. 

##### (3R)-3-(piperidin-3-yl)-1H-indole hydrochloride **(R)-10a** (Figure 10)

^1^H-NMR (500 MHz, D_2_O): δ 7.70 (C4″H, [1H], dd, ^3^J = 8.0), 7.53 (C7″H, [1H], m, ^3^J = 7.0, ^4^J = 0.5), 7.27 (C6″H, [1H], m), 7.21 (C2″H, [1H], s), 7.18 (C5″H, [1H], m), 3.57 (CaH(E), [1H], m), 3.47 (CeH(E), [1H], m), 3.29 (CbH(A), [1H], tt, ^3^J_A-A_ = 12.0, ^3^J_A-E_ = 4.0), 2.98 (CaH(A), CeH(A), [2H], m), 2.14 (CcH(E), [1H], m), 2.04 (CdH(E), [1H], m), 1.89 (CdH(A), [1H], m), 1.74 (CcH(A), [1H], m).

^13^C-NMR (125 MHz, D_2_O): δ 135.7 (C7″a, s), 124.9 (C3″a, s), 121.8 (C6″, s), 121.6 (C2″, s), 119.0 (C5″, s), 118.0 (C4″, s), 114.4 (C3″, s), 111.7 (C7″, s), 48.3 (Ca,s), 43.7 (Ce, s), 30.6 (Cb,s), 28.3 (Cc, s), 21.9 (Cd, s).

ESI-HRMS *m*/*z*: Calcd for C_13_H_17_N_2_ [M+H]^+^ 201.13917. Found: 201.13874

HPLC separation method: LUX Cellulose-3 5 µm, 250 × 4.6 mm column; T = 35 °C; phase A: 0.05% diisopropylamine in ethanol; phase B: hexane; flow: 1 mL/min, 40% of phase B, isocratic elution; detection at 220 nm.

Crystal data for orthorhombic polymorph of **(R)-10a**. Formula C_13_H_17_N_2_Cl, M_w_ = 236.74. Crystal system orthorhombic at 293 K, space group *P*2_1_2_1_2_1_, unit cell dimensions a = 6.173(2) Å, b = 10.788(3) Å, c = 18.982(4) Å, V = 1264.1(6) Å^3^, Z = 4, D_calc_ = 1.244 g/cm^3^, μ = 2.457 mm^−1^, F(000) = 504. θ range for data collection 4.66 to 76.14; reflections collected/independent 8343/2605 [R(int) = 0.0285]. Goodness-of-fit on F^2^ 1.266, final R indices [I > 2σ(I)] R1 = 0.0346, wR2 = 0.0882; R indices (all data) R1 = 0.0415, wR2 = 0.0939. CCDC No. 2085101.

Crystal data for monoclinic polymorph of **(R)-10a**. Formula C_13_H_17_N_2_Cl, M_w_ = 236.74. Crystal system monoclinic at 120K, space group *P*2_1_, unit cell dimensions a = 6.091(1) Å, b = 18.884(3) Å, c = 10.795(2) Å, β = 90.57(2)°, V = 1241.6(4) Å^3^, Z = 4, D_calc_ = 1.266 g/cm^3^, μ = 2.502 mm^−1^, F(000) = 504. θ range for data collection 4.66 to 73.28°; reflections collected/independent 3220/2645 [R(int) = 0.019]. Goodness-of-fit on F^2^ 1.202; final R indices [I > 2σ(I)] R1 = 0.0416, wR2 = 0.0891, R indices (all data) R1 = 0.0503, wR2 = 0.1111. CCDC No. 2085100.

**Figure 10:**
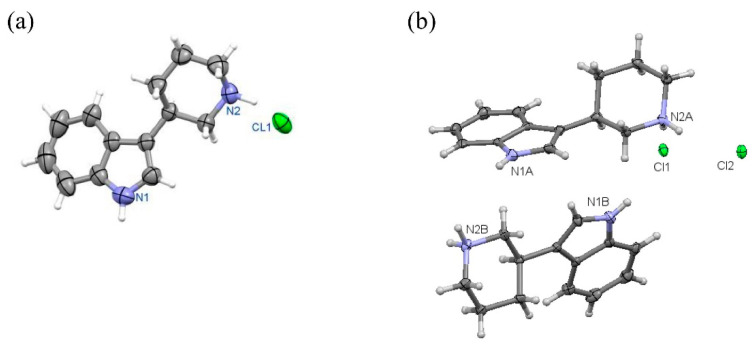
Perspective view of molecules: **(R)-10a** in orthorhombic (**a**) and monoclinic (**b**) polymorph.

##### (3S)-3-(piperidin-3-yl)-1H-indole hydrochloride **(S)-11a** (Figure 11)

^1^H-NMR (500 MHz, D_2_O): δ 7.70 (C4″H, [1H], dd, ^3^J = 8.0), 7.53 (C7″H, [1H], m, ^3^J = 7.0, ^4^J = 0.5), 7.27 (C6″H, [1H], m), 7.20 (C2″H, [1H], s), 7.18 (C5″H, [1H], m), 3.57 (CaH(E), [1H], m), 3.47 (CeH(E), [1H], m), 3.28 (CbH(A), [1H], tt, ^3^J_A-A_ = 12.0, ^3^J_A-E_ = 4.0), 2.99 (CaH(A), CeH(A), [2H], m), 2.14 (CcH(E), [1H], m), 2.05 (CdH(E), [1H], m), 1.89 (CdH(A), [1H], m), 1.76 (CcH(A), [1H], m).

^13^C-NMR (125 MHz, D_2_O): δ 135.7 (C7″a, s), 124.9 (C3″a, s), 121.8 (C6″, s), 121.6 (C2″, s), 119.0 (C5″, s), 118.0 (C4″, s), 114.4 (C3″, s), 111.7 (C7″, s), 48.3 (Ca,s), 43.7 (Ce, s), 30.6 (Cb,s), 28.3 (Cc, s), 21.9 (Cd, s).

ESI-HRMS *m*/*z*: Calcd for C_13_H_17_N_2_ [M+H]^+^ 201.13917. Found: 201.13874

HPLC separation method: LUX Cellulose-3 5 µm, 250 × 4.6 mm column; T = 35 °C; phase A: 0.05% diisopropylamine in ethanol; phase B: hexane; flow: 1 mL/min, 40% of phase B, isocratic elution; detection at 220 nm.

Crystal data for **(S)-11a**. Formula C_13_H_17_N_2_Cl, M_w_ = 236.74. Crystal system orthorhombic, space group *P*2_1_2_1_2_1_, unit cell dimensions a = 6.181(1) Å, b = 10.795(2) Å, c = 18.967(3) Å, V = 1265.6(4) Å^3^, Z = 4, D_calc_ = 1.242 g/cm^3^, μ = 2.455 mm^−1^, F(000) = 504. θ range for data collection 4.66 to 73.28°; reflections collected/independent 3248/2162 [R(int) = 0.0263]. Goodness-of-fit on F^2^ 1.116; final R indices [I > 2σ(I)] R1 = 0.0427, wR2 = 0.0878; R indices (all data) R1 = 0.0576, wR2 = 0.1138. CCDC No. 2085102.

**Figure 11:**
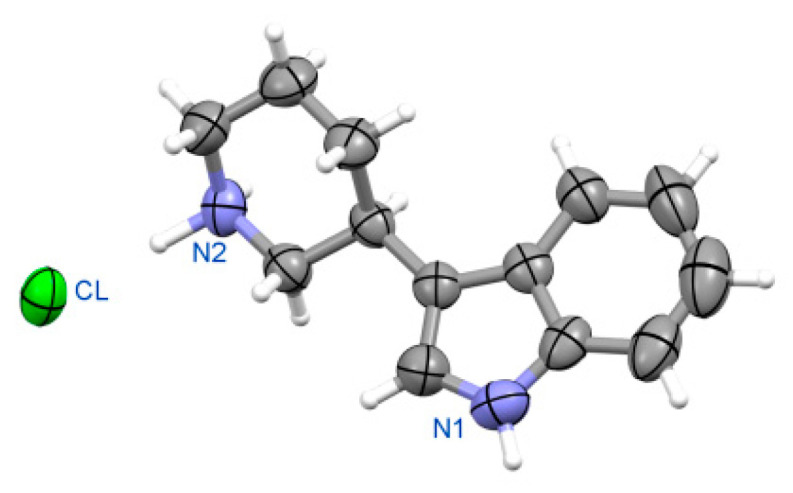
Perspective view of molecule **(S)-11a**.

##### (3R)-5-fluoro-3-(piperidin-3-yl)-1H-indole hydrochloride **(R)-10b** (Figure 12)

^1^H-NMR (500 MHz, D_2_O): δ 7.43 (C7″H, [1H], 4d, ^3^J = 9.0, ^4^J_H-F_ = 5.0, ^5^J = 0.5), 7.34 (C4″H, [1H], 4d, ^3^J_H-F_ = 10.0, ^4^J = 2.5, ^5^J = 0.5), 7.24 (C2″H, [1H], s), 7.02 (C6″H, [1H], 8d, ^3^J = 9.5, ^3^J_H-F_ = 8.0, ^4^J = 2.5, ^5^J = 0.5), 3.56 (CaH(E). [1H], m), 3.49 (CeH(E), [1H], m), 3.22 (CbH, [1H], tt, ^3^J_A-A_ = 12.0, ^3^J_A-E_ = 3.5), 3.01 (CeH(A), [1H], td, ^2^J = ^3^J_A-A_ = 13.0, ^3^J_A-E_ = 3.5), 2.98 (CaH(A), [1H], t, ^2^J = ^3^J_A-A_ = 12.5), 2.01–2.14 (CcH(E),CdH(E), [2H], m), 1.83–1.95 (CdH(A), [1H], m), 1.72 (CcH(A), [1H], kd, ^2^J = ^3^J_A-A_ = 12.0, ^3^J_A-E_ = 4.0).

^13^C-NMR (125 MHz, D_2_O): δ 156.9 (C5″, d, ^1^J = 231.9), 132.3 (C3″, s), 125.1 (C3″a, d, ^3^J = 9.9), 123.3 (C2″, s), 114.6 (C7″a, d, ^4^J = 4.8), 112.6 (C7″, d, ^3^J = 9.9), 109.8 (C6″, d, ^2^J = 26.4), 102.5 (C4″, d, ^2^J = 23.8), 48.2 (Ca, s), 43.7 (Ce, s), 30.6 (Cb, s), 28.2 (Cc, s), 21.9 (Cd, s).

ESI-HRMS *m*/*z*: Calcd for C_13_H_16_FN_2_ [M+H]^+^ 219.12975. Found: 219.12935

HPLC separation method: LUX Cellulose-3 5 µm, 250 × 4.6 mm column; T = 35 °C; phase A: 0.05% diisopropylamine in ethanol/methanol 80/20; phase B: hexane; flow: 0.5 mL/min, 92% of phase B, isocratic elution; detection at 220 nm.

Crystal data for **(R)-10b**. Formula C_13_H_16_FN_2_Cl; M_w_ 254.73. Crystal system orthorhombic, space group *P*2_1_2_1_2_1_, unit cell dimensions a = 5.510(1) Å, b = 11.905(2) Å, c = 19.148(3) Å, V = 1256.0(4) Å^3^, Z = 4, D_calc_ = 1.347 g/cm^3^, μ = 2.631 mm^−1^, F(000) = 536. θ range for data collection 4.37 to 76.33°; reflections collected/independent 8559/2577 [R(int) = 0.0341]. Goodness-of-fit on F^2^ 1.156; final R indices [I > 2σ(I)] R1 = 0.0308, wR2 = 0.0715, R indices (all data) R1 = 0.0385, wR2 = 0.0964. CCDC No. 2085104.

Crystal data for racemic **1b**. Formula C_13_H_16_FN_2_Cl; M_w_ 254.73. Crystal system monoclinic, space group *P*2_1_/*c*, unit cell dimensions a = 10.736(3) Å, b = 6.117(2) Å, c = 20.151(4) Å, β = 97.84(3)°, V = 1311.0(6) Å^3^, Z = 4, D_calc_ = 1.291 g/cm^3^, μ = 2.521 mm^−1^, F(000) = 536. θ range 4.16 to 73.53°; reflections collected/independent 4524/2572 [R(int) = 0.0244]. Goodness-of-fit on F^2^ 1.067; final R indices [I > 2σ(I)]: R1 = 0.0456, wR2 = 0.1161; R indices (all data) R1 = 0.0536, wR2 = 0.1254. CCDC No. 2085103. See Figure 4, Perspective view of molecule 1b in racemic crystal.

**Figure 12:**
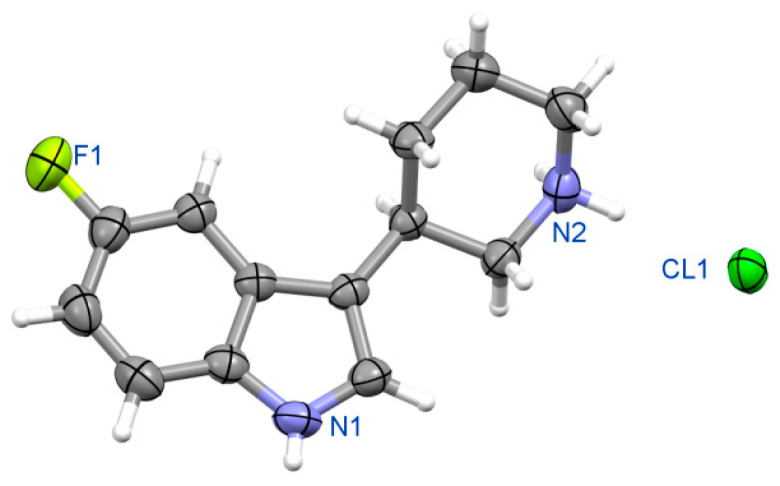
View of molecular structure of enantiomeric fluoro- derivative **(R)-10b**.

##### (3S)-5-fluoro-3-(piperidin-3-yl)-1H-indole hydrochloride **(S)-11b** (Figure 13)

^1^H-NMR (500 MHz, D_2_O): δ 7.44 (C7″H, [1H], 4d, ^3^J = 9.0, ^4^J_H-F_ = 5.0, ^5^J = 0.5), 7.35 (C4″H, [1H], 4d, ^3^J_H-F_ = 10.0, ^4^J = 2.5, ^5^J = 0.5), 7.24 (C2″H, [1H], s), 7.03 (C6″H, [1H], 8d, ^3^J = 9.5, ^3^J_H-F_ = 8.0, ^4^J = 2.5, ^p^J = 0.5), 3.56 (CaH(E). [1H], m), 3.49 (CeH(E), [1H], m), 3.22 (CbH, [1H], tt, ^3^J_A-A_ = 12.0, ^3^J_A-E_ = 3.5), 3.01 (CeH(A), [1H], td, ^2^J = ^3^J_A-A_ = 13.0, ^3^J_A-E_ = 3.5), 2.98 (CaH(A), [1H], t, ^2^J = ^3^J_A-A_ = 12.5), 2.01–2.14 (CcH(E),CdH(E), [2H], m), 1.83–1.95 (CdH(A), [1H], m), 1.72 (CcH(A), [1H], kd, ^2^J = ^3^J_A-A_ = 12.0, ^3^J_A-E_ = 4.0).

^13^C-NMR (125 MHz, D_2_O): δ 156.9 (C5″, d, ^1^J = 231.9), 132.3 (C3″, s), 125.1 (C3″a, d, ^3^J = 9.9), 123.3 (C2″, s), 114.6 (C7″a, d, ^4^J = 4.8), 112.6 (C7″, d, ^3^J = 9.9), 109.8 (C6″, d, ^2^J = 26.4), 102.5 (C4″, d, ^2^J = 23.8), 48.2 (Ca, s), 43.7 (Ce, s), 30.6 (Cb, s), 28.2 (Cc, s), 21.9 (Cd, s).

ESI-HRMS *m*/*z*: Calcd for C_13_H_16_FN_2_ [M+H]^+^ 219.12975. Found: 219.12936

HPLC separation method: LUX Cellulose-3 5 µm, 250 × 4.6 mm column; T = 35 °C; phase A: 0.05% diisopropylamine in ethanol/methanol 80/20; phase B: hexane; flow: 0.5 mL/min, 92% of phase B, isocratic elution; detection at 220 nm.

Crystal data for **(S)-11b**. Formula C_13_H_16_N_2_FCl; M_w_ 254.73. Crystal system orthorhombic, space group *P*2_1_2_1_2_1_, unit cell dimensions a = 5.499(1) Å, b = 11.719(2) Å, c = 19.103(3) Å, V = 1231.1(4) Å3, Z = 4, D_calc_ = 1.374 g/cm3, μ = 2.685 mm−1, F(000) = 536. θ range for data collection 4.43 to 76.35; reflections collected/independent 8080/2514 [R(int) = 0.0352]. Goodness-of-fit on F2 1.136; final R indices [I > 2σ(I)] R1 = 0.0277, wR2 = 0.0648, R indices (all data) R1 = 0.0365, wR2 = 0.0764. CCDC No. 2085105.

**Figure 13:**
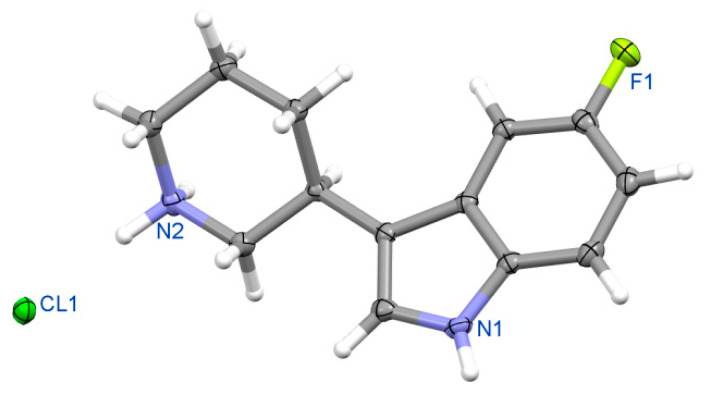
View of molecular structure of enantiomeric fluoro- derivative **(S)-11b**.

##### (3R)-5-methoxy-3-(piperidin-3-yl)-1H-indole hydrochloride **(R)-10c**

^1^H-NMR (500 MHz, D_2_O): δ 7.43 (C7″H, [1H], dd, ^3^J = 9.0, ^4^J = 0.5), 7.23 (C2″H, [1H], s), 7.18 (C4″H, [1H], d, ^4^J = 2.5), 6.93 (C6″H, [1H], dd, ^3^J = 9.0, ^4^J = 2.5), 3.90 (OCH_3_, [3H], s), 3.63 (CaH(E), [1H], m), 3.51 (CeH(E), [1H], m), 3.32 (CbH, [1H], tt, ^3^J_A-A_ = 12.0, ^3^J_A-E_ = 4.0), 3.08 (CaH(A), [1H], t, ^2^J = ^3^J_A-A_ = 12.5), 3.06 (CeH(A), [1H], td, ^2^J = ^3^J_A-A_ = 13.0, ^3^J_A-E_ = 3.0), 2.22 (CcH(E), [1H], m), 2.09 (CdH(E), [1H], m), 1.93 (CdH(A), [1H], m), 1.81 (CcH(A), [1H], kd, ^2^J = ^3^J_A-A_ = 13.0, ^3^J_A-E_ = 3.5).

^13^C-NMR (125 MHz, D_2_O): δ 153.0 (C5″, s), 131.6 (C7″a, s), 125.7 (C3″a, s), 123.0 (C2″, s), 114.8 (C3″, s), 113.1 (C6″, s), 112.0 (C7″, s), 100.9 (C4″, s), 56.3 (OCH_3_, 2s), 48.7 (Ca, s), 44.2 (Ce, s), 31.0 (Cb, s), 28.9 (Cc, s), 22.4 (Cd, s).

ESI-HRMS *m*/*z*: Calcd for C_14_H_19_N_2_O [M+H]^+^ 231.14973. Found: 231.14941

HPLC separation method: LUX Cellulose-3 5 µm, 250 × 4.6 mm column; T = 35 °C; phase A: 0.05% diisopropylamine in ethanol; phase B: hexane; flow: 1 mL/min, 80% of phase B, isocratic elution; detection at 220 nm.

##### (3S)-5-methoxy-3-(piperidin-3-yl)-1H-indole hydrochloride **(S)-11c**

^1^H-NMR (500 MHz, D_2_O): δ 7.46 (C7″H, [1H], d, ^3^J = 9.0), 7.27 (C2″H, [1H], s), 7.22 (C4″H, [1H], d, ^4^J = 2.5), 6.96 (C6″H, [1H], dd, ^3^J = 9.0, ^4^J = 2.5), 3.90 (OCH_3_, [3H], s), 3.63 (CaH(E), [1H], m), 3.51 (CeH(E), [1H], m), 3.32 (CbH, [1H], tt, ^3^J_A-A_ = 12.0, ^3^J_A-E_ = 4.0), 3.08 (CaH(A), [1H], t, ^2^J = ^3^J_A-A_ = 12.5), 3.06 (CeH(A), [1H], td, ^2^J = ^3^J_A-A_ = 13.0, ^3^J_A-E_ = 3.0), 2.22 (CcH(E), [1H], m), 2.09 (CdH(E), [1H], m), 1.93 (CdH(A), [1H], m), 1.81 (CcH(A), [1H], kd, ^2^J = ^3^J_A-A_ = 13.0, ^3^J_A-E_ = 3.5).

^13^C-NMR (125 MHz, D_2_O): δ 153.0 (C5″, s), 131.6 (C7″a, s), 125.7 (C3″a, s), 123.0 (C2″, s), 114.8 (C3″, s), 113.1 (C6″, s), 112.0 (C7″, s), 100.9 (C4″, s), 56.3 and 56.3 (OCH_3_, 2s*), 48.7 (Ca, s), 44.2 (Ce, s), 31.0 (Cb, s), 28.9 (Cc, s), 22.4 (Cd, s).

ESI-HRMS *m*/*z*: Calcd for C_14_H_19_N_2_O [M+H]^+^ 231.14973. Found: 231.14931

HPLC separation method: LUX Cellulose-3 5 µm, 250 × 4.6 mm column; T = 35 °C; phase A: 0.05% diisopropylamine in ethanol; phase B: hexane; flow: 1 mL/min, 80% of phase B, isocratic elution; detection at 220 nm.

**Figure 14:**
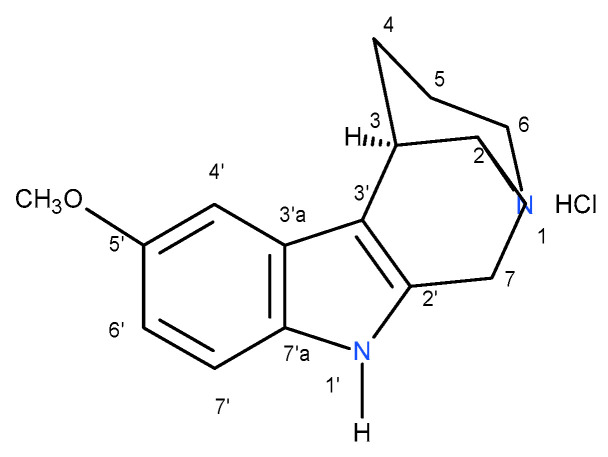
Numbering system for NMR spectra interpretation of compounds (R)-**12c** and (S)-**13c**.

Numbering system for NMR spectra interpretation of compounds (R)-**12c** and (S)-**13c** is shown in Figure 14.

##### (R)-5-methoxy-9,12-diazatetracyclo[10.3.1.0^2,10^.0^3,8^]hexadeca-2(10),3(8),4,6-tetraene hydrochloride **(R)-12c** (Figure 15)

^1^H-NMR (500 MHz, MeOD): δ 7.27 (C7′H, [1H], dd, ^3^J = 8.5, ^5^J = 0.5), 6.99 (C4′H, [1H], d, ^4^J = 2.5), 6.80 (C6′H, [1H], dd, ^3^J = 8.5, ^4^J = 2.5), 4.86 (C7H(2), [1H], d, ^2^J = 16.5), 4.47 (C7H(1), [1H], ^2^J = 16.5), 3.81 (OCH_3_, [3H], s), 3.68 (C2H(2), [1H], dd, ^2^J = 12.5, ^3^J = 3.0), 3.49–3.57 (C2H(1),C3H,C6H_2_, [4H], m), 1.95–2.03 (C4H(2), [1H] m), 1.83–1.90 (C4H(1), [1H], m), 1.60–1.72 (C5H_2_, [2H], m).

^13^C-NMR (125MHz, MeOD): δ 155.7 (C5′, s), 133.6 (C7′a, s), 128.0 (C2′, s), 126.5 (C3′a, s), 113.3 (C6′, s), 113.1 (C7′, s), 110.4 (C3′, s), 100.8 (C4′, s), 56.3 (OCH_3_, s), 55.5 (C6, s), 53.8 (C2, s), 50.5 (C7, s), 26.8 (C4, s), 26.4 (C3, s), 17.5 (C5, s).

ESI-HRMS *m*/*z*: Calcd for C_15_H_19_N_2_O [M+H]^+^ 243,14973. Found: 243,14919 

Crystal data for **(R)-12c**. Formula C_15_H_19_N_2_OCl; M_w_ 278.77. Crystal system orthorhombic, space group *P*2_1_2_1_2_1_, unit cell dimensions a = 5.670(1) Å, b = 7.534(1) Å, c = 32.732(4) Å, V = 1398.2(4) Å^3^, Z = 4, D_calc_ = 1.324 g/cm^3^, μ = 2.360 mm^−1^, F(000) = 592. θ range for data collection 5.41 to 76.46°; reflections collected/independent 9422/2875 [R(int) = 0.0263]. Goodness-of-fit on F^2^ 1.112; final R indices [I > 2σ(I)] R1 = 0.0293, wR2 = 0.0692, R indices (all data) R1 = 0.0357, wR2 = 0.0797. CCDC No. 2085106.

**Figure 15:**
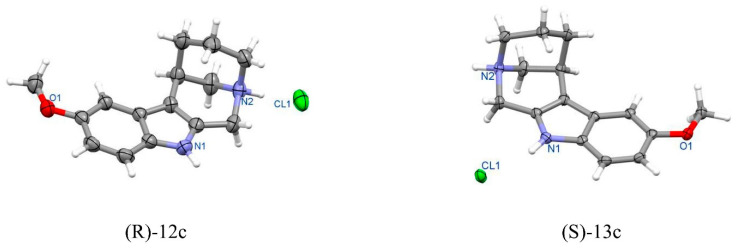
Perspective view of enantiomeric tetracyclic molecules **(R)-12c** and **(S)-13c**.

##### (S)-5-methoxy-9,12-diazatetracyclo[10.3.1.0^2,10^.0^3,8^]hexadeca-2(10),3(8),4,6-tetraene hydrochloride **(S)-13c** (Figure 15)

^1^H-NMR (500 MHz, MeOD): δ 7.26 (C7′H, [1H], dd, ^3^J = 8.5, ^5^J = 0.5), 7.07 (C4′H, [1H], dd, ^3^J = 2.5, ^4^J = 0.5), 6.79 (C6′H, [1H], 4d, ^3^J = 8.5, ^4^J = 2.5, ^5^J = 0.5), 3.81 (OCH_3_, [3H], s), 3.58 (C7H(2), [1H], m), 3.43–3.60 (C7H(1), [1H], m), 3.26–3.35 (C2H(2), [1H], m), 2.57–3.09 (C2H(1),,C6H(1), [2H], m), 2.20 (C6(1), [1H], m, 2.05–2.11 (C4H(2), [1H], m), 1.91–2.02 (C4H(1), [1H], m), 1.81–1.92 (C5H_2_, [2H], m).

^13^C-NMR (125MHz, MeOD): δ 155.7 (C5′, s), 133.6 (C7′a, s), 128.0 (C2′, s), 126.5 (C3′a, s), 113.3 (C6′, s), 113.1 (C7′, s), 110.4 (C3′, s), 100.8 (C4′, s), 56.3 (OCH_3_, s), 55.5 (C6, s), 53.8 (C2, s), 50.5 (C7, s), 26.8 (C4, s), 26.4 (C3, s), 17.5 (C5, s).

ESI-HRMS *m*/*z*: Calcd for C_15_H_19_N_2_O [M+H]^+^ 243.14973. Found: 243.14708 

Crystal data for **(S)-13c**. Formula C_15_H_19_N_2_OCl; M_w_ 278.77. Crystal system orthorhombic, space group *P*2_1_2_1_2_1_, unit cell dimensions a = 5.583(1) Å, b = 7.486(1) Å, c = 32.589(3) Å,V = 1362.0(3) Å3, Z = 4, D_calc_ = 1.359 g/cm^3^, μ = 2.423 mm^−1^, F(000) = 592. θ range for data collection 5.43 to 76.64°; reflections collected/independent 19,347/2830 [R(int) = 0.0507]. Goodness-of-fit on F2 1.078; final R indices [I > 2σ(I)] R1 = 0.0269, wR2 = 0.0609, R indices (all data) R1 = 0.0285, wR2 = 0.0621. CCDC No. 2085107.

## 4. Conclusions

N-alkylation reactions of 3-(piperidin-3-yl)-1H-indole **1a** with (R)-2-(4-toluenesulfonyloxy)-phenyl acetic acid methyl ester **(R)-I** led to the formation of a mixture of diastereomers (3R, 2S)-methyl-2-[3-(1H-indol-3-yl)-1-piperidyl}-2-phenyl-acetamide **(3R,2S)-2a** and diastereomer **(3S,2S)-3a**. In the next step, to improve their purity (dr) and their easier separation into the N-alkylation reaction of the **1a-c** derivatives, (S)-(+)-2-(4-toluenesulfonyloxy)-phenylacetic amide **(S)-II** was used. Mixtures of the diastereomers of the series (3R,2R)-2-[3-(1H-indol-3-yl)-1-piperidyl]-2-phenyl acetamides **(3R,2R)-4a**, **(3R,2R)-6b**, **(3R,2R)-8c** and of the series (3S,2R)-2-[3-(1H-indol-3-yl)-1-piperidyl]-2-phenyl acetamides **(3S,2R)-5a**, **(3S,2R)-7b**, **(3S,2R)-9c** were obtained. Column chromatography was used to separate the diastereomers, and analytically pure compounds were obtained and subjected to hydrogenolysis to give the final (R)-3-(piperidin-3-yl)-1H-indole **(R)-10 a-c** and a series of (S)-3-(piperidine)-3-yl)-1H-indole **(S)-11a-c**. During isolation, after the hydrogenolysis of **(3R,2R)-8c** and **(3S,2R)-9c** diastereomers, new **(R)-12c** and **(S)-13c** compounds were obtained in addition to amines **(R)-10c** and **(S)-11c**, the structures of which have been confirmed. The structure of the compounds was tetracyclic. The structure of all the new compounds obtained was confirmed by spectroscopy tests ^1^H and ^13^C NMR, HRMS, HPLC, dr, ee. 

Finally, the absolute configuration for the enantiomeric pairs **(R)-10a/(S)-11a**, **(R)-10b/(S)-11b** and **(R)-12c**/**(S)-13c**, as well for **(3S,2S)-3a** was determined using anomalous dispersion effect observed in X-ray crystallography. 

## Figures and Tables

**Table 1:** N-alkylation of 3-(piperidin-3-yl)-1H-indole derivatives **1a-c** using (R)-2-(4-toluene-sulfonyloxy)-phenylacetic acid methylester (R)-I and (S)-2-(4-toluenesulfonyloxy)-phenylacetic amide **(S)-II**.

Compound	Chiral Substrate (R)-I or (S)-II	Substrate 1a-c	Yield (%) ^a^	dr ^b^ of	(3R,2S)-2a:(3S,2S)-3a
					(3R,2R)-4a:(3S,2R)-5a
					(3R,2R)-6b:(3S,2R)-7b
					(3R,2R)-8c:(3S,2R)-9c
**1**	**(R)-I**	**1a**	74		47.6:52.4
**2**	**(S)-II**	**1a**	85		46.6:53.4
**3**	**(S)-II**	**1b**	79		48.3:51.7
**4**	**(S)-II**	**1c**	91		48.1:51.9

For all entries, the reaction was performed by heating the mixture in 45 °C in acetonitrile; ^a^ isolated yield; ^b^ estimated by means of HPLC.

**Table 2:** Physico-chemical data of diastereomers **2a**, **3a**, **4a**, **5a**, **6b**, **7b**, **8c**, and **9c**.

Compound	Substrate 1a-c/Chiral Substrate (R)-I or (S)-II	Reaction Time (h)	m.p. (°C)	Yield (%) ^a^	dr ^b^	[α]
**(3R,2S)-2a**	**1a/(R)-I**	5	149–150	12.9	93.9	+3.9° c = 10.0, CHCl_3_
**(3S,2S)-3a**	**1a/(R)-I**	5	140–143	12.8	93.2	+19.9° c = 8.40, CHCl_3_
**(3R,2R)-4a**	**1a/(S)-II**	14	118–122	51.2	100.0	−66.53° c = 2.40, CHCl_3_
**(3S,2R)-5a**	**1a/(S)-II**	14	103–108	48.6	100.0	+16.63° c = 2.40, CHCl_3_
**(3R,2R)-6b**	**1b/(S)-II**	18	202–206	27.3	99.9	−71.99° c = 6.00, CHCl_3_
**(3S,2R)-7b**	**1b/(S)-II**	18	124–126	21.9	98.4	+20.51° c = 2.34, CHCl_3_
**(3R,2R)-8c**	**1c/(S)-II**	20	165–169	38.6	99.5	−58.99° c = 1.00, CHCl_3_
**(3S,2R)-9c**	**1c/(S)-II**	20	202–207	19.7	97.3	+58.99° c = 1.00, CHCl_3_

^a^ isolated yield. ^b^ estimated by means of HPLC.

**Table 3:** Data on chiral (R) and (S) 3-(piperidin-3-yl)-1H-indol derivatives **(R)**-**10a-c**, **(S)**-**11a-c** and **(R)-12c**, **(S)-13c**.

Compound	Substrate	Yield (%) ^a^	m.p. (°C)	ee ^b^	[α]
**(R)-10a**	**(3R,2S)-2a** **(3R,2R)-4a**	80	301–303	100.0	−30.53° c = 1.70, MeOH
**(S)-11a**	**(3S,2S)-3a** **(3S,2R)-5a**	82	295–297	95.9	+30.93° c = 1.00, MeOH
**(R)-10b**	**(3R,2R)-6b**	61	287–289	100.0	−24.79° c = 0.36, MeOH
**(S)-11b**	**(3S,2R)-7b**	66	289–291	100.0	+28.19° c = 0.53, MeOH
**(R)-10c**	**(3R,2R)-8c**	70	267–269	99.2	−39.21° c = 0.19, MeOH
**(S)-11c**	**(3S,2R)-9c**	76	280–282	100.0	+31.69° c = 0.65, MeOH
**(R)-12c**	**(3R,2R)-8c**	18	300–302	99.7	+95.89° c = 0.64, MeOH
**(S)-13c**	**(3S,2R)-9c**	15	270–272	99.7	−97.96° c = 0.35, MeOH

^a^ isolated yield. ^b^ estimated by means of chiral HPLC.

## Data Availability

Data are contained within the article or supplementary material.

## Supplementary Materials

The supporting information can be downloaded at: https://www.mdpi.com/article/10.3390/ijms24010517/s1.
