# Supplementary material for: The Synthesis and Absolute Configuration of Enantiomeric Pure (R)- and (S)-3-(piperidin-3-yl)-1H-Indole Derivatives
Source: Int J Mol Sci. 2022 Dec 28;24(1):517. doi: 10.3390/ijms24010517 (PMC9820373; doi:10.3390/ijms24010517)

(R)-10a

Analysis Report

<Sample Information>

Sample Name	: Pr A rozc
Sample ID	: 0,05 DIPA
Data Filename	: 2021-06-22_1101_Pr A rozc_0,05 DIPA_001 OK.lcd
Method Filename	: LUX-1 PDA 18.06.2021r.lcm
Batch Filename	: LUXy 18.06.2021r.lcb
Vial #	: 1-1
Injection Volume	: 2 uL
Acquired by	: PP

<Chromatogram>

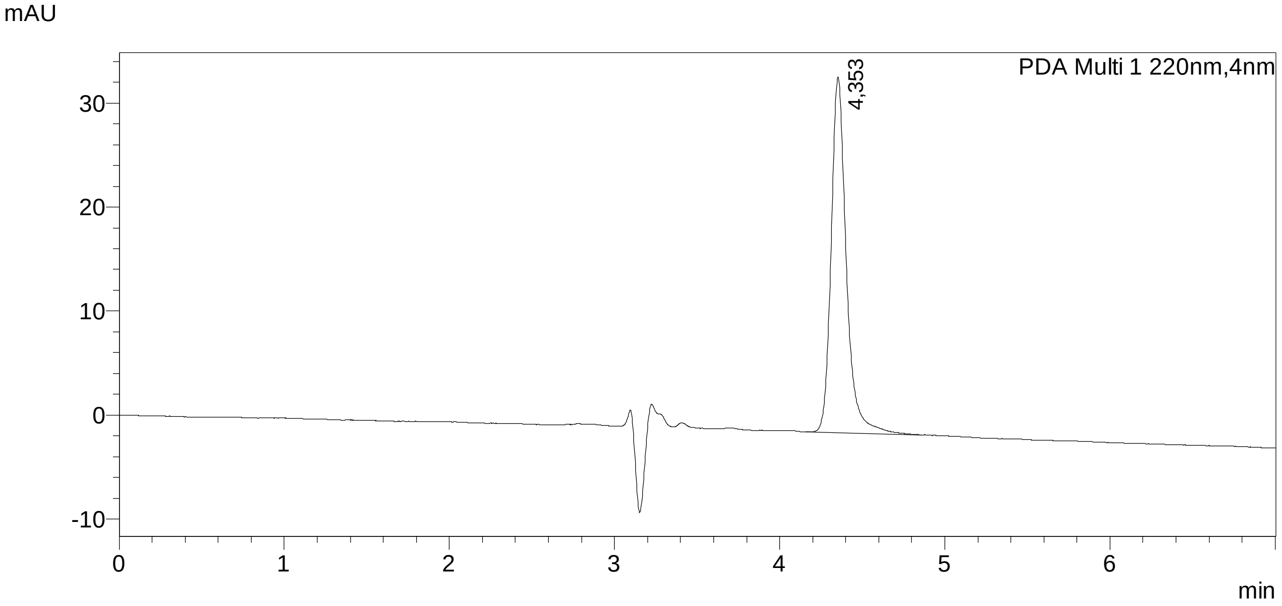

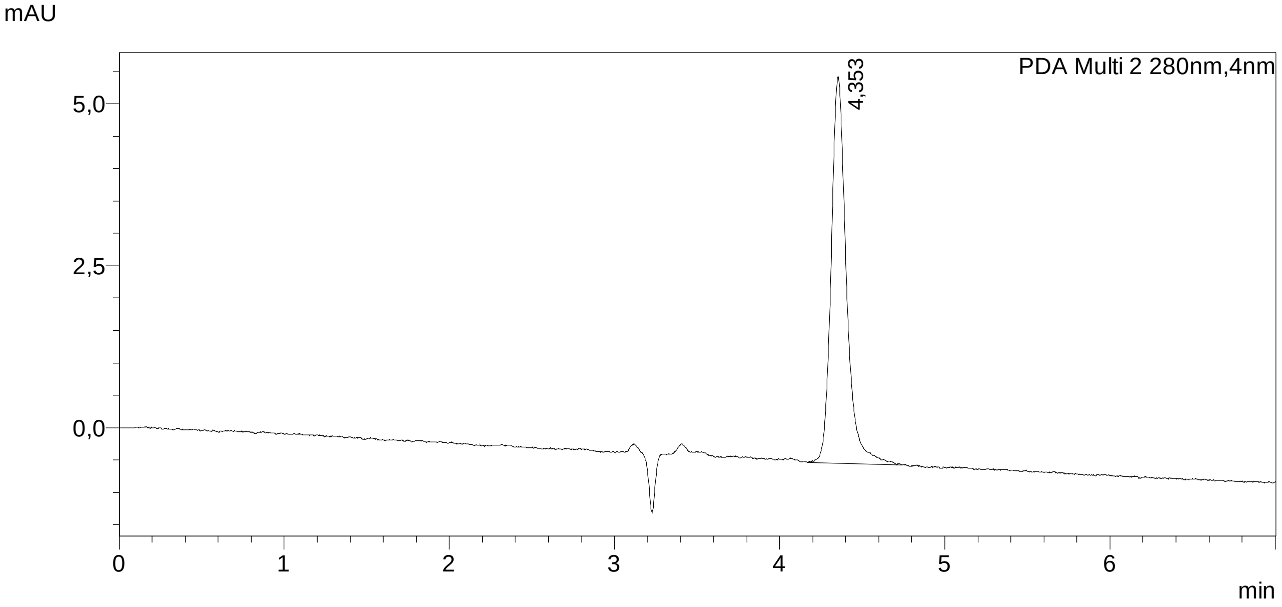

Peak Table

PDA Ch1 220nm

Peak#	Ret. Time	Area	Area%	Height	Height%
1	4,353	219709	100,000	33984	100,000
Total		219709	100,000	33984	100,000

PDA Ch2 280nm

Peak#	Ret. Time	Area	Area%	Height	Height%
1	4,353	38388	100,000	5926	100,000
Total		38388	100,000	5926	100,000

SHIMADZU LabSolutions

Analysis Report

<Sample Information>

Sample Name	: Pr B rozc
Sample ID	: 0,05 DIPA
Data Filename	: 2021-06-22_1101_Pr B rozc_0,05 DIPA_002 OK.lcd
Method Filename	: LUX-1 PDA 18.06.2021r.lcm
Batch Filename	: LUXy 18.06.2021r.lcb
Vial #	: 1-2
Injection Volume	: 1 uL
Acquired by	: PP

<Chromatogram>

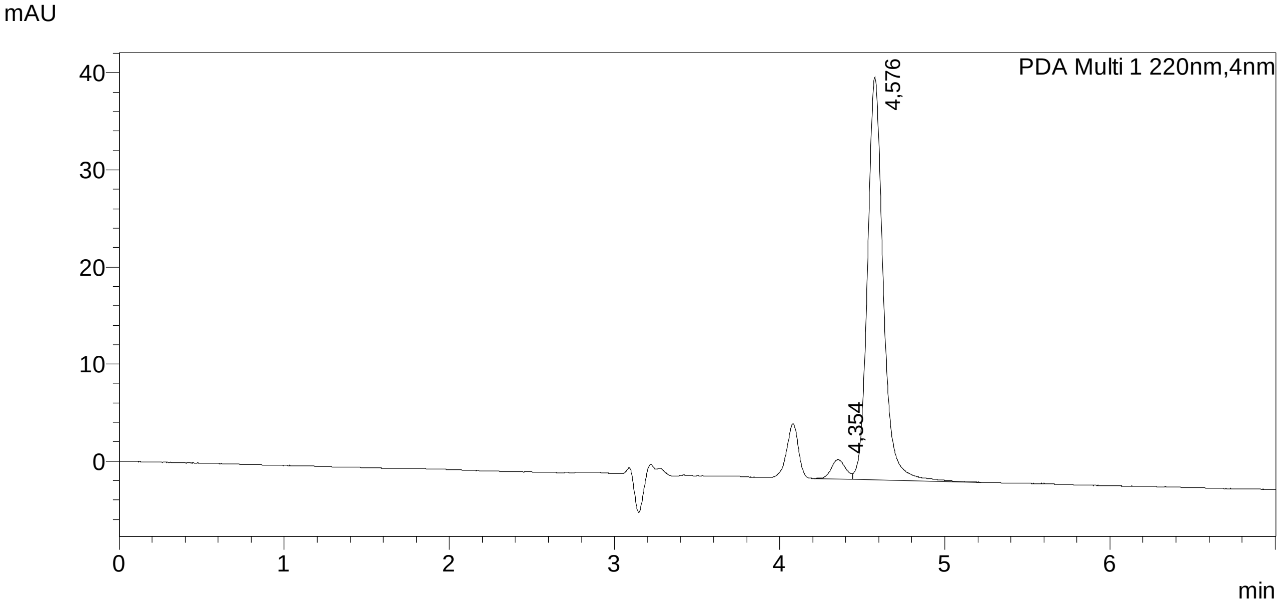

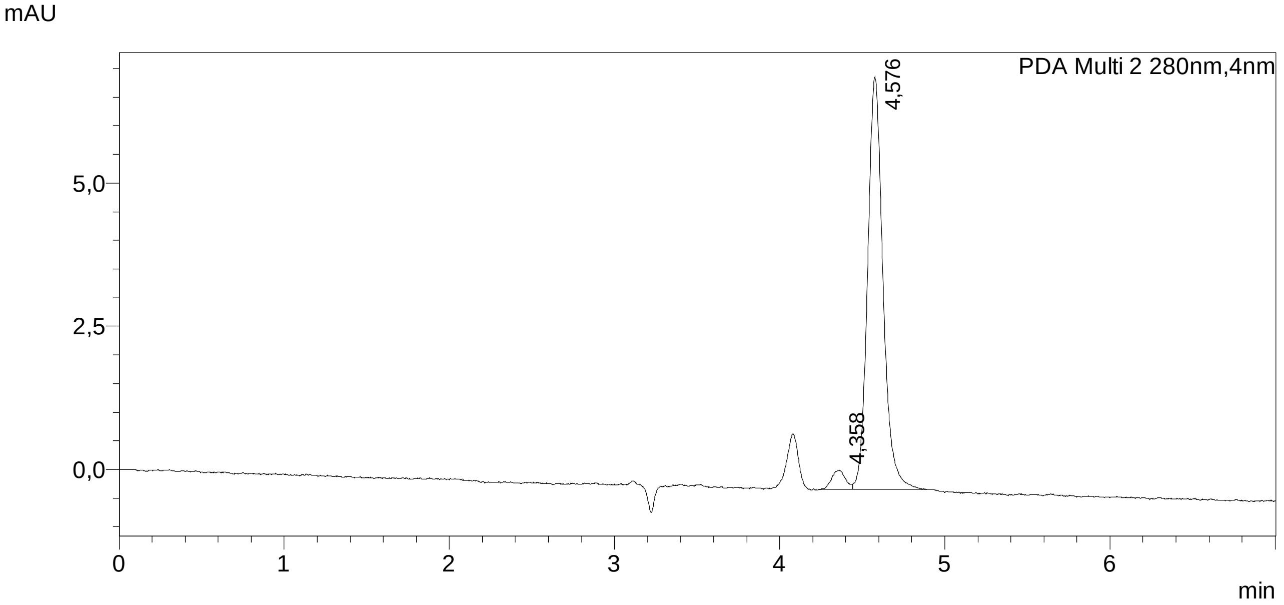

Peak Table

PDA Ch1 220nm

Peak#	Ret. Time	Area	Area%	Height	Height%
1	4,354	12060	4,205	1986	4,589
2	4,576	274719	95,795	41283	95,411
Total		286780	100,000	43268	100,000

PDA Ch2 280nm

Peak#	Ret. Time	Area	Area%	Height	Height%
1	4,358	1984	4,075	337	4,497
2	4,576	46709	95,925	7164	95,503
Total		48693	100,000	7502	100,000

(R)-10b

Analysis Report

<Sample Information>

Sample Name : Pr C r20
Sample ID : DIPA
Data Filename : 2021-07-07_1331_Pr C r20_DIPA_001 OK.lcd
Method Filename : LUX-1 PDA 18.06.2021r.lcm
Batch Filename : LUXy 18.06.2021r.lcb
Vial # : 1-6
Injection Volume : 10 uL
Acquired by : PP

<Chromatogram>

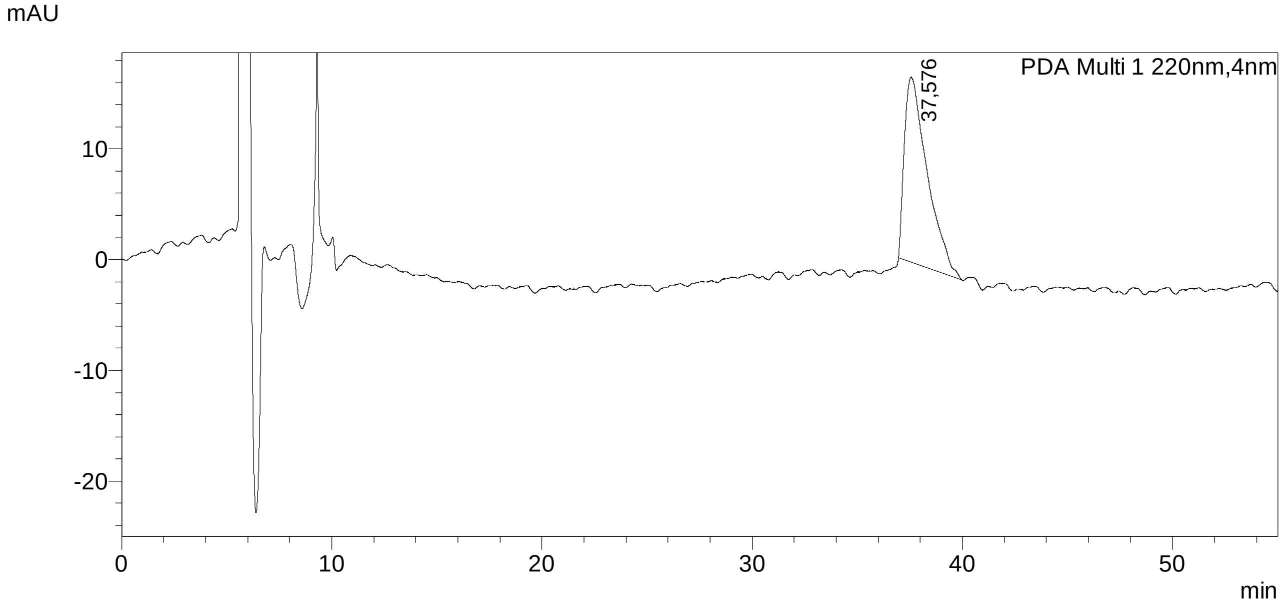

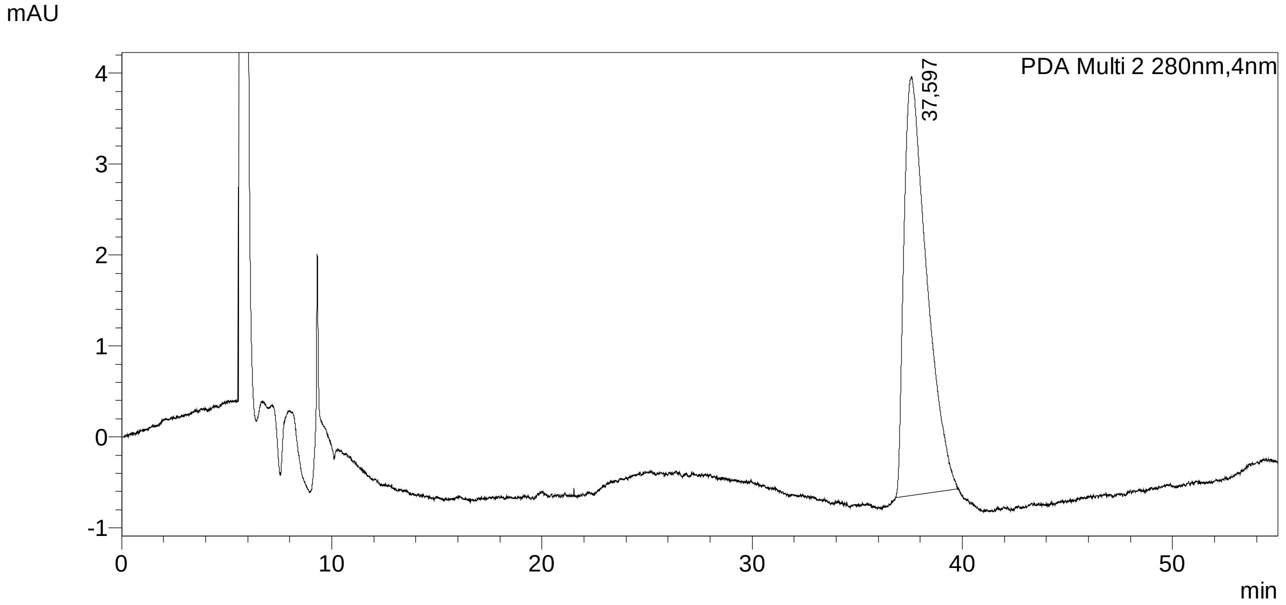

Peak Table

PDA Ch1 220nm

Peak#	Ret. Time	Area	Area%	Height	Height%
1	37,576	1269390	100,000	16698	100,000
Total		1269390	100,000	16698	100,000

PDA Ch2 280nm

Peak#	Ret. Time	Area	Area%	Height	Height%
1	37,597	346882	100,000	4601	100,000
Total		346882	100,000	4601	100,000

(S)-11b

SHIMADZU LabSolutions

Analysis Report

<Sample Information>

Sample Name	: Pr D r20
Sample ID	: DIPA
Data Filename	: 2021-07-07_1331_Pr D r20_DIPA_002 OK.lcd
Method Filename	: LUX-1 PDA 18.06.2021r.lcm
Batch Filename	: LUXy 18.06.2021r.lcb
Vial #	: 1-7
Injection Volume	: 10 uL
Acquired by	: PP

<Chromatogram>

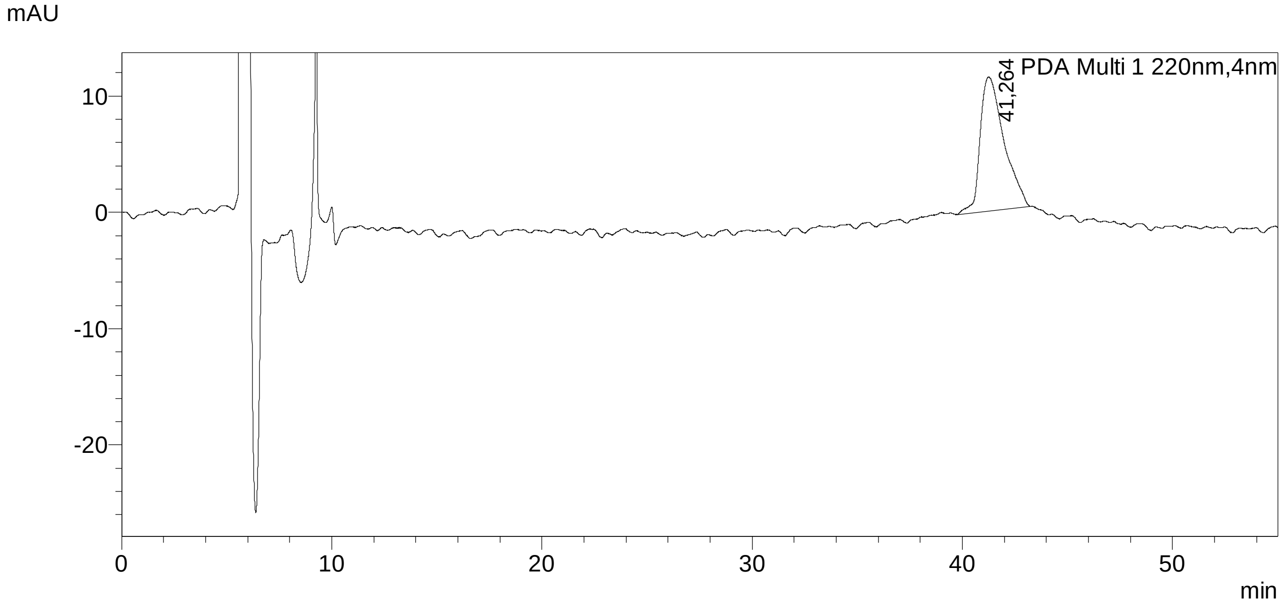

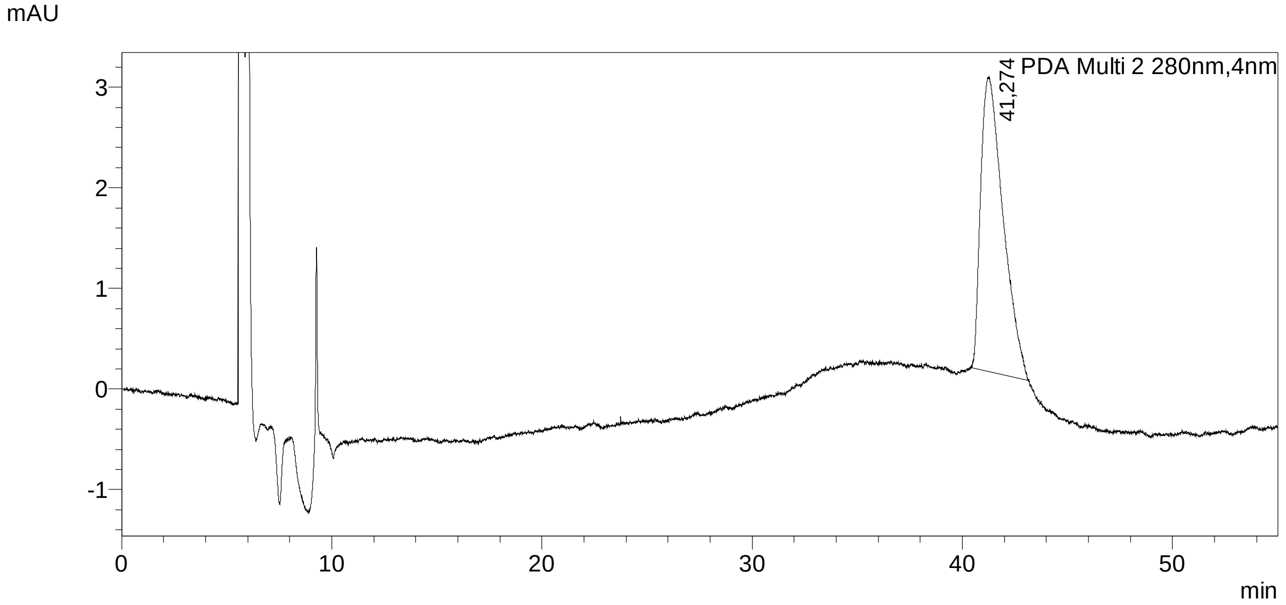

Peak Table

PDA Ch1 220nm

Peak#	Ret. Time	Area	Area%	Height	Height%
1	41,264	906164	100,000	11494	100,000
Total		906164	100,000	11494	100,000

PDA Ch2 280nm

Peak#	Ret. Time	Area	Area%	Height	Height%
1	41,274	217240	100,000	2924	100,000
Total		217240	100,000	2924	100,000

Analysis Report

SHIMADZU LabSolutions

<Sample Information>

Sample Name	: Pr E II
Sample ID	:
Data Filename	: 2021-07-09_1332_Pr E II_DIPA_001 OK.lcd
Method Filename	: LUX-1 PDA 18.06.2021r.lcm
Batch Filename	: LUXy 18.06.2021r.lcb
Vial #	: 1-13
Injection Volume	: 4 uL
Acquired by	: PP

<Chromatogram>

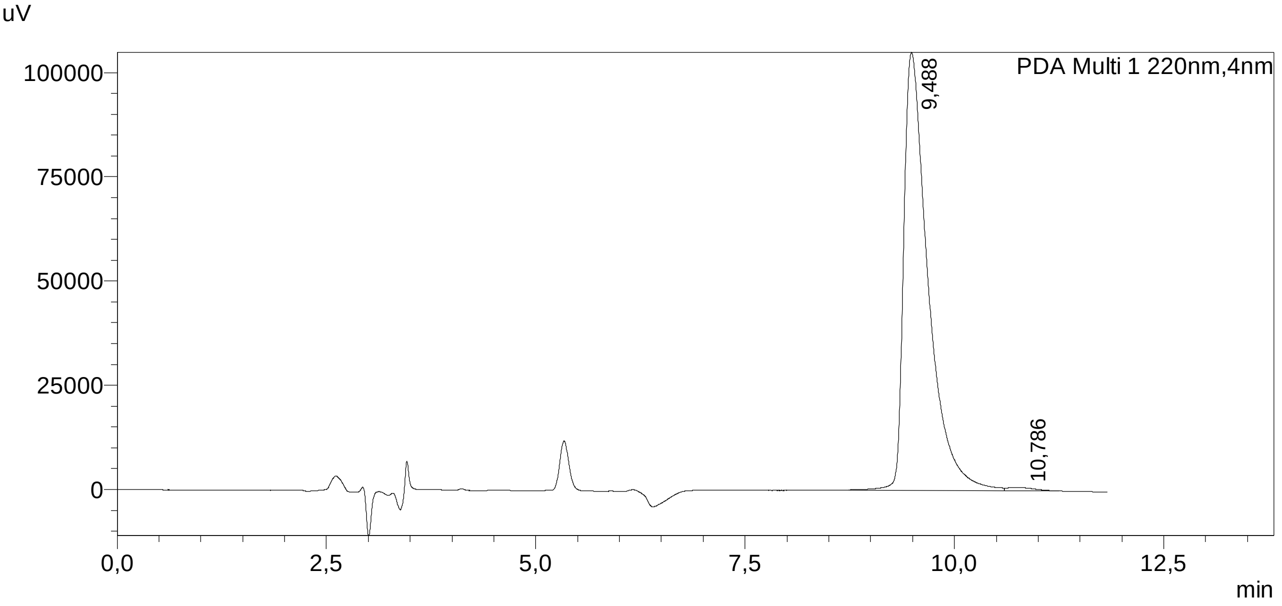

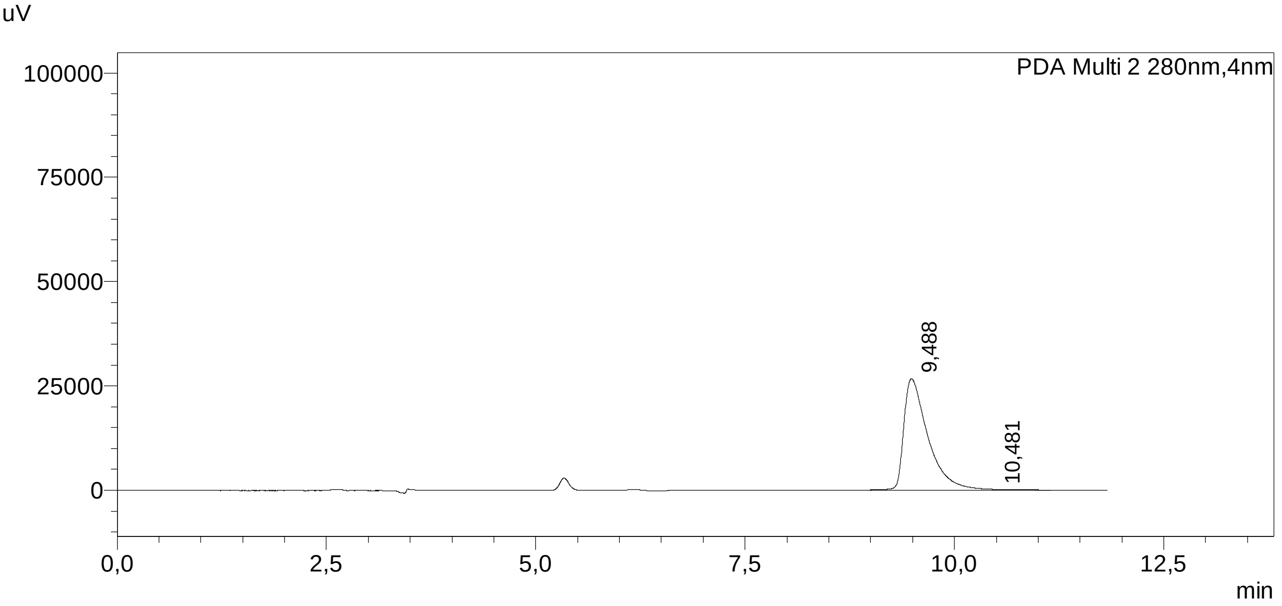

Peak Table

PDA Ch1 220nm

Peak#	Ret. Time	Area	Area%	Height	Height%
1	9,488	2127203	99,182	104877	99,275
2	10,786	17549	0,818	765	0,725
Total		2144753	100,000	105642	100,000

PDA Ch2 280nm

Peak#	Ret. Time	Area	Area%	Height	Height%
1	9,488	535678	99,167	26635	99,297
2	10,481	4499	0,833	189	0,703
Total		540176	100,000	26824	100,000

SHIMADZU LabSolutions

Analysis Report

<Sample Information>

Sample Name	: Pr F II
Sample ID	:
Data Filename	: 2021-07-09_1332_Pr F II_DIPA_002 OK.lcd
Method Filename	: LUX-1 PDA 18.06.2021r.lcm
Batch Filename	: LUXy 18.06.2021r.lcb
Vial #	: 1-14
Injection Volume	: 5 uL
Acquired by	: PP

<Chromatogram>

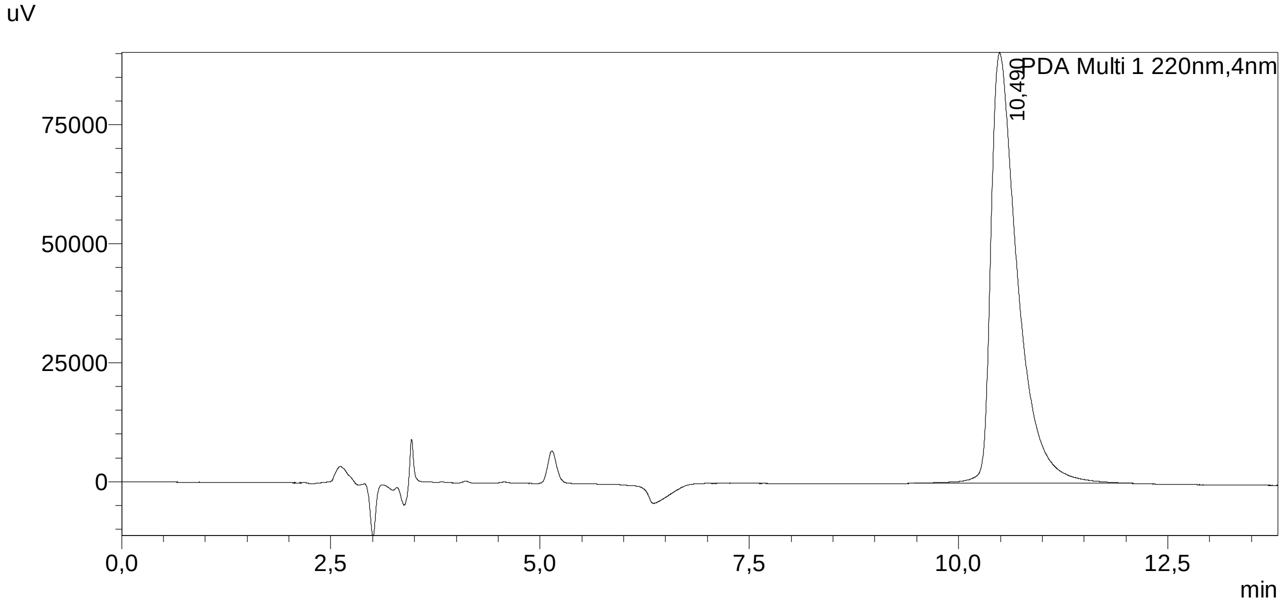

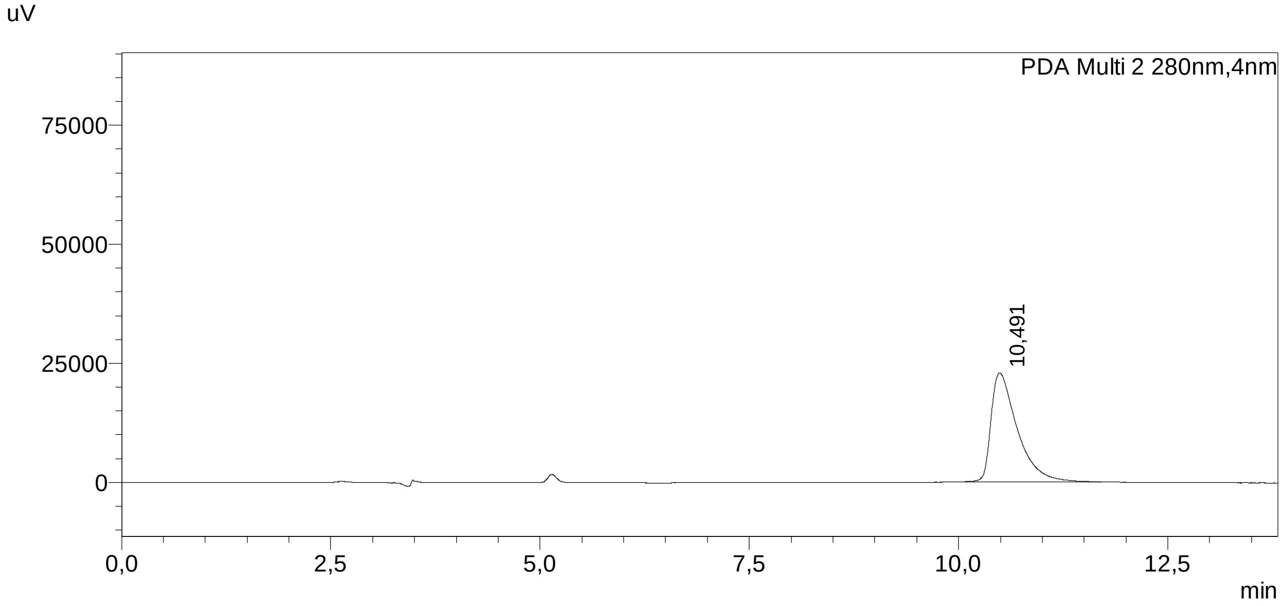

Peak Table

PDA Ch1 220nm

Peak#	Ret. Time	Area	Area%	Height	Height%
1	10,490	2016170	100,000	90448	100,000
Total		2016170	100,000	90448	100,000

PDA Ch2 280nm

Peak#	Ret. Time	Area	Area%	Height	Height%
1	10,491	498794	100,000	22876	100,000
Total		498794	100,000	22876	100,000

==== Shimadzu LabSolutions Data Comparison ====

Data1 : C:\LabSolutions\Data\Król\Analizy 18.06.2021r\2021-06-22_1101_Pr A rozc_0,05 DIPA_001 OK.lcd
Data2 : C:\LabSolutions\Data\Król\Analizy 18.06.2021r\2021-06-22_1101_Pr B rozc_0,05 DIPA_002 OK.lcd

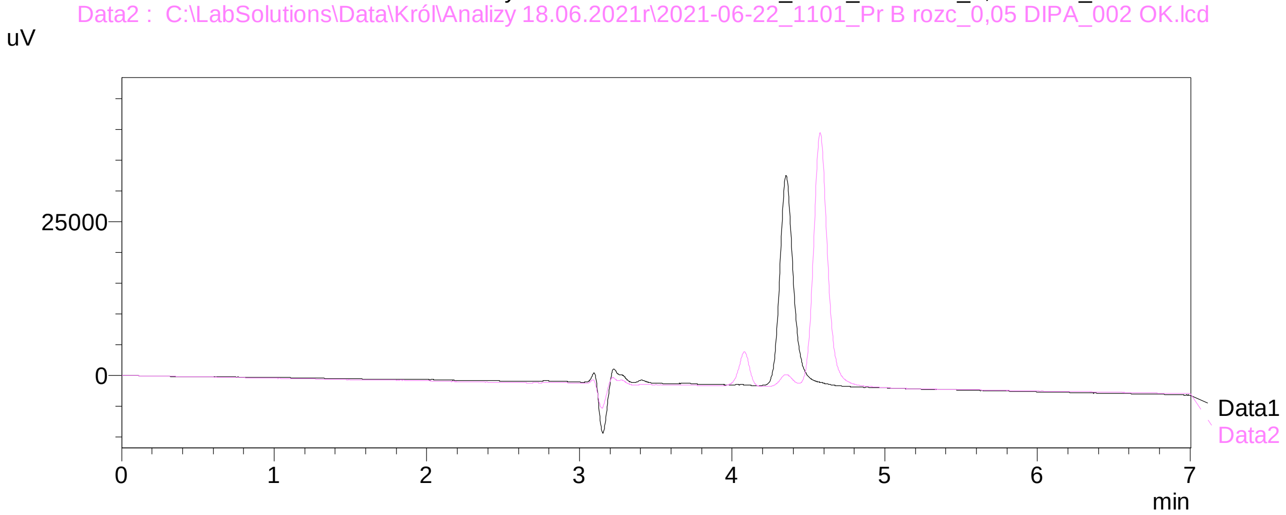

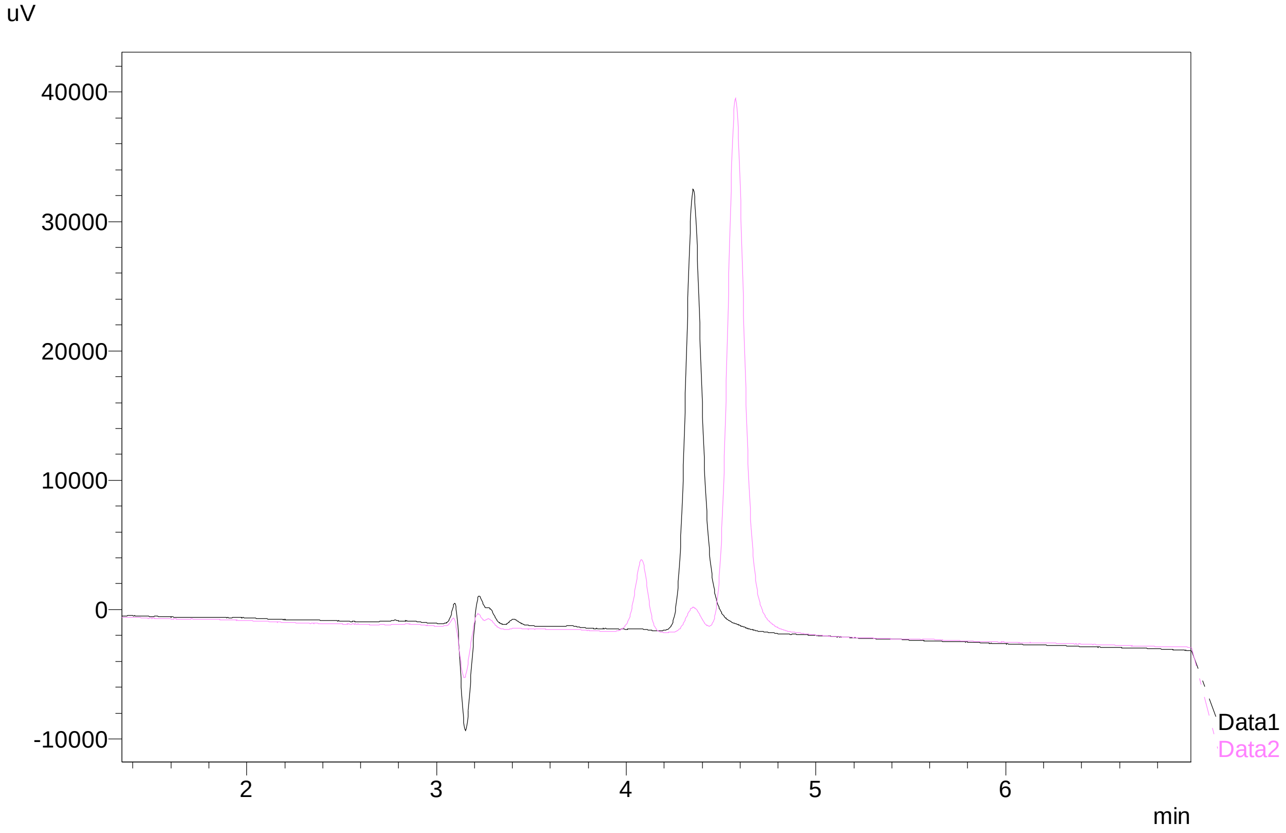

(R)-10b : (S)-11b

==== Shimadzu LabSolutions Data Comparison ====

Data1 : C:\LabSolutions\Data\Król\Analizy 18.06.2021r\2021-07-07_1331_Pr C r20_DIPA_001 OK.lcd
Data2 :* C:\LabSolutions\Data\Król\Analizy 18.06.2021r\2021-07-07_1331_Pr D r20_DIPA_002 OK.lcd

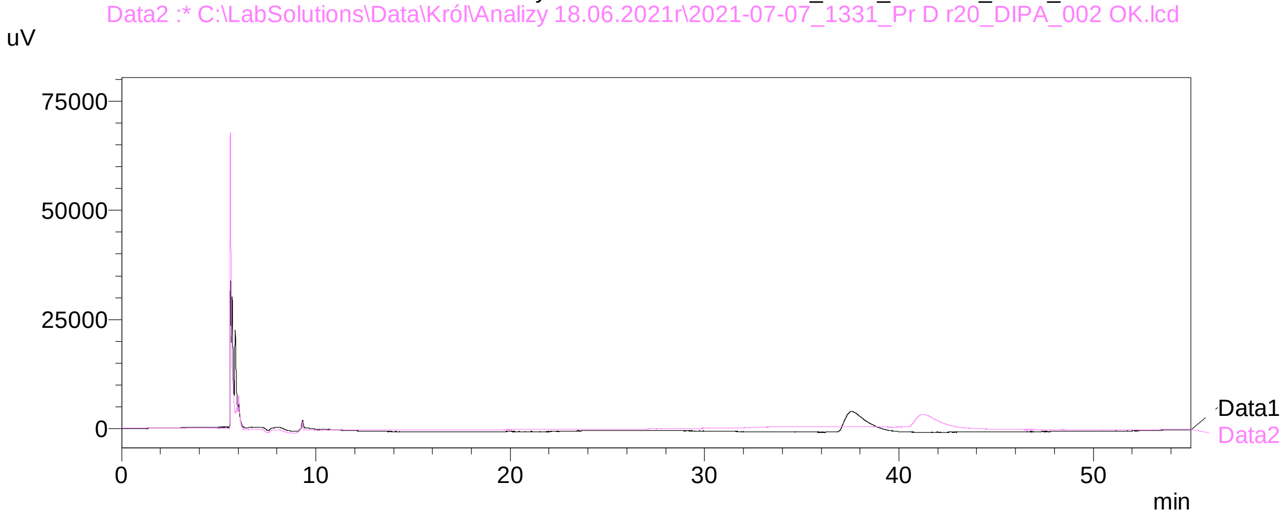

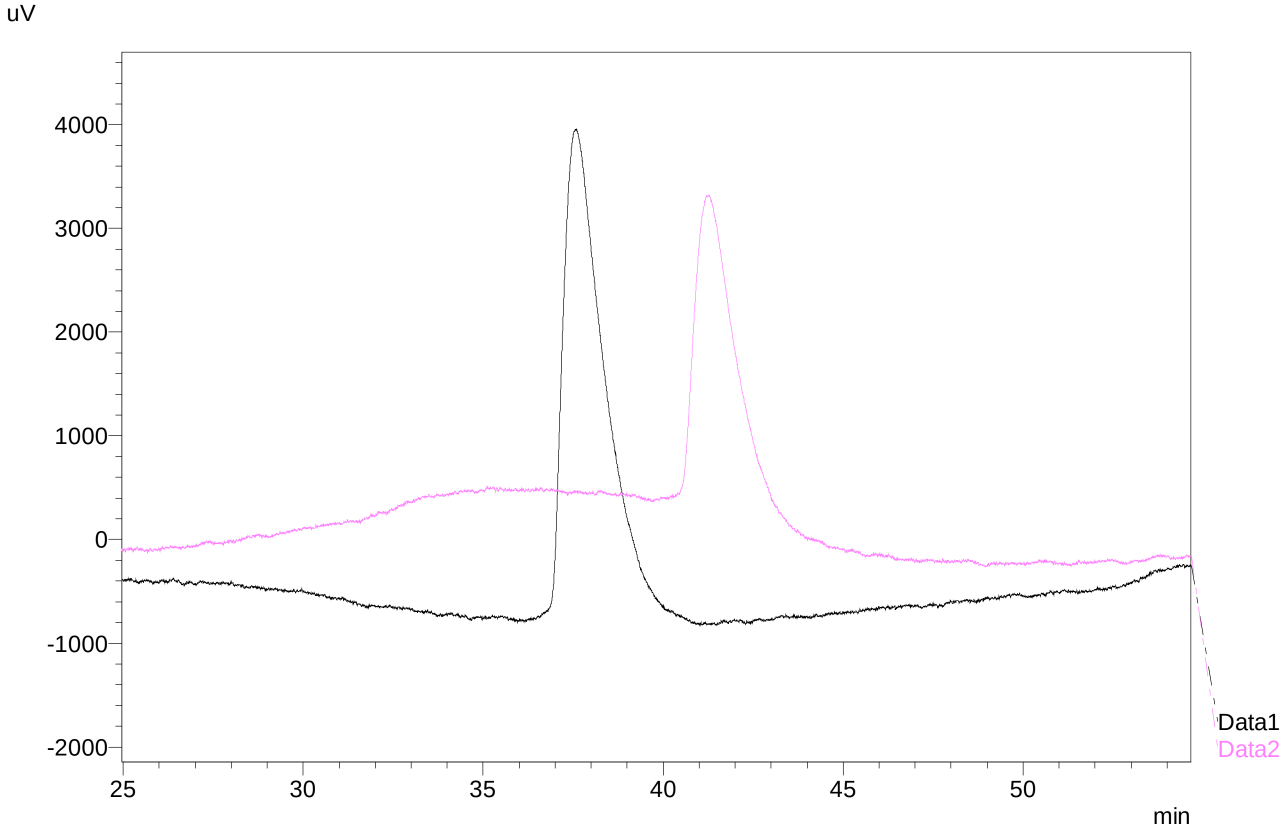

(R)-10c : (S)-11c

==== Shimadzu LabSolutions Data Comparison ====

Data1 : C:\LabSolutions\Data\Król\Analizy 18.06.2021r\2021-07-09_1332_Pr E II_DIPA_001 OK.lcd
Data2 : C:\LabSolutions\Data\Król\Analizy 18.06.2021r\2021-07-09_1332_Pr F II_DIPA_002 OK.lcd

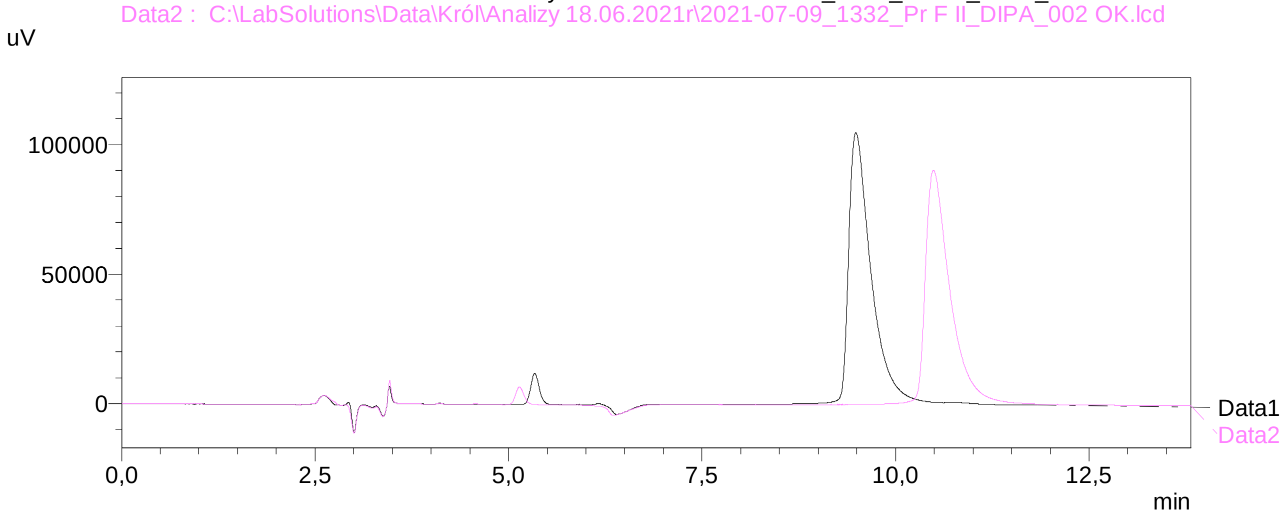

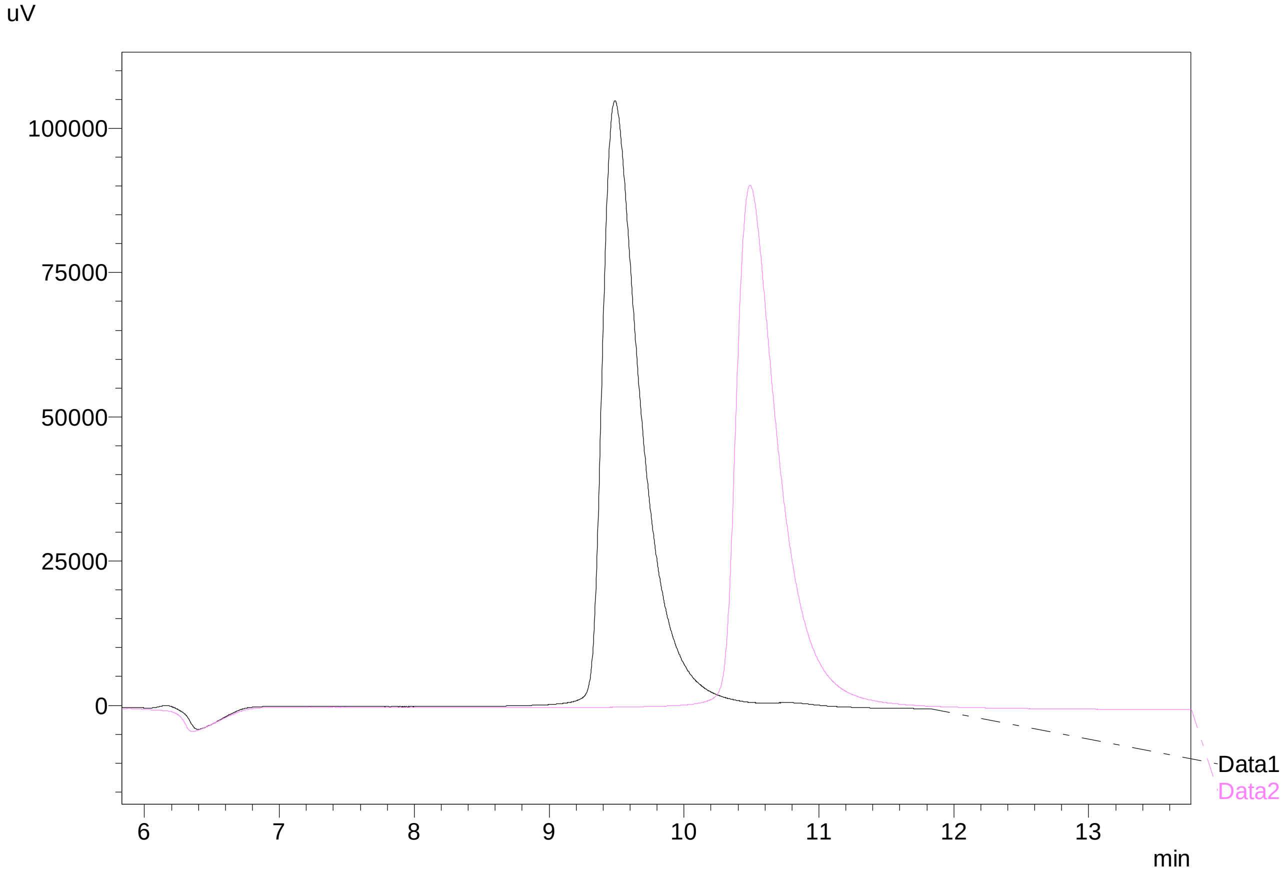

(R)-12c

Analysis Report

<Sample Information>

Sample Name	: cE		
Sample ID	: r10 B70 t25		
Data Filename	: 2022-03-22_1140_cE_r10 B70 t25_001.lcd		
Method Filename	: LUX Cykle PDA 14.03.2022r.lcm		
Batch Filename	: Cykle cE i cF LUX.lcb		
Vial #	: 1-1	Sample Type	: Unknown
Injection Volume	: 40 uL		
Date Acquired	: 2022-03-22 11:41:12	Acquired by	: System Administrator
Date Processed	: 2022-03-22 14:51:16	Processed by	: System Administrator

<Chromatogram>

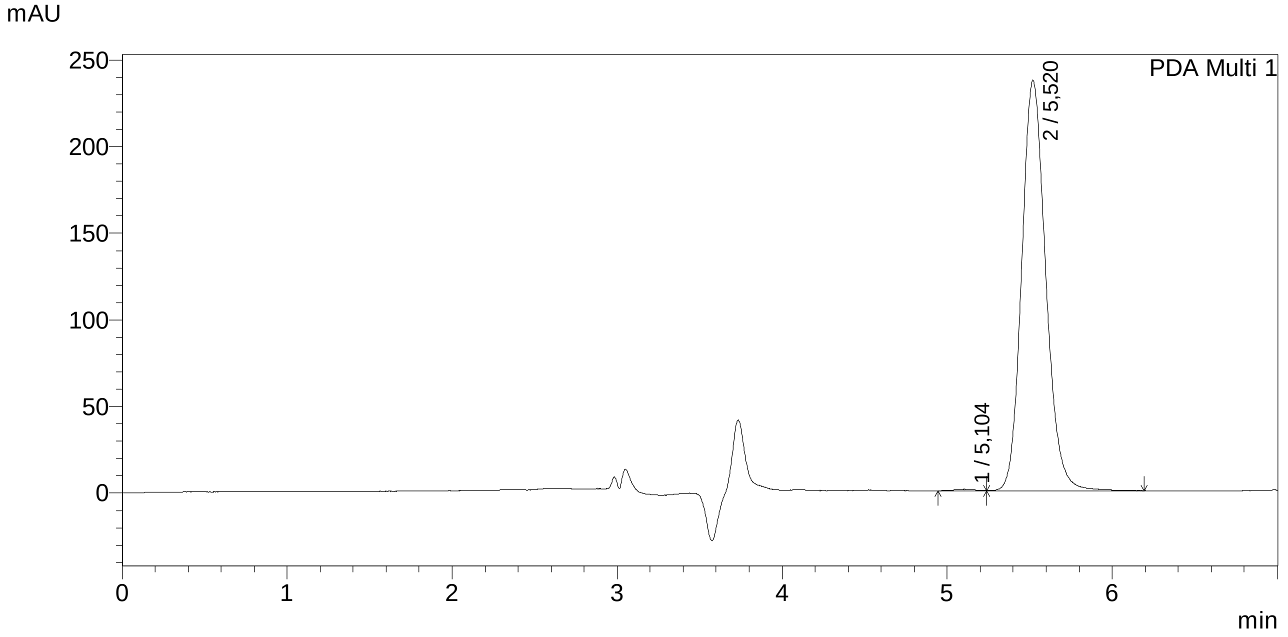

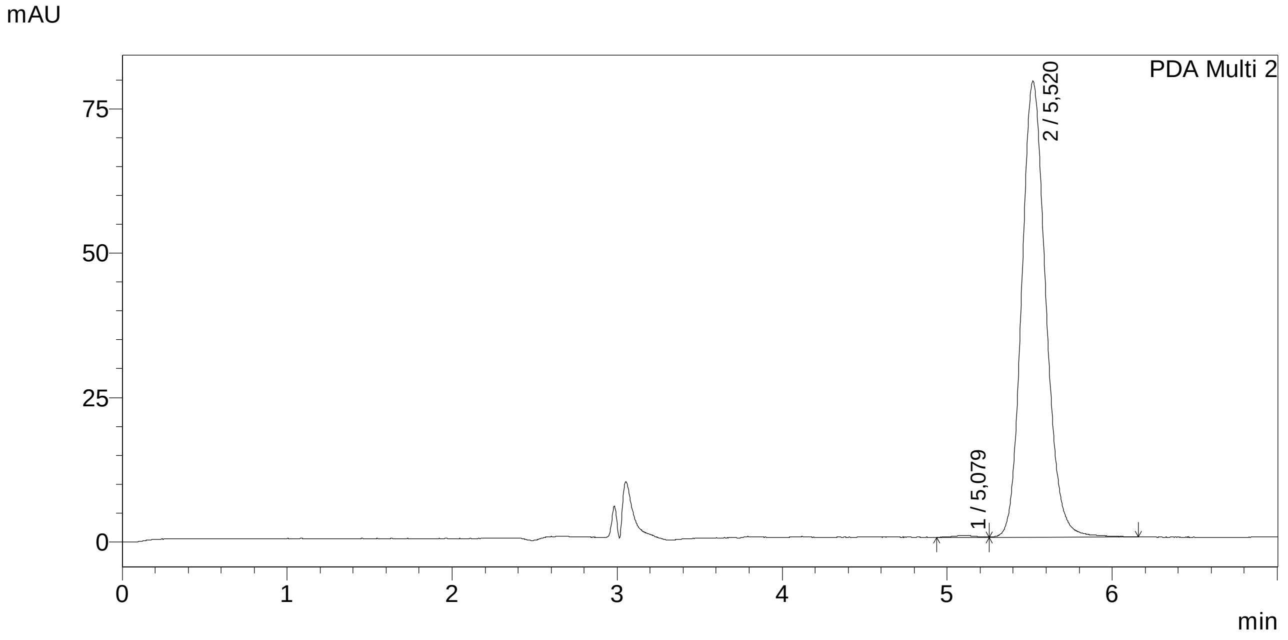

PDA Ch1 220nm

Peak#	Ret. Time	Area	Area%	Height	Height%
1	5,104	8306	0,344	885	0,372
2	5,520	2407246	99,656	236671	99,628
Total		2415551	100,000	237555	100,000

PDA Ch2 280nm

Peak#	Ret. Time	Area	Area%	Height	Height%
1	5,079	2782	0,346	283	0,357
2	5,520	800979	99,654	78989	99,643
Total		803761	100,000	79272	100,000

(S)-13c

SHIMADZU LabSolutions

Analysis Report

<Sample Information>

Sample Name	: cF		
Sample ID	: r10 B70 t25		
Data Filename	: 2022-03-22_1140_cF_r10 B70 t25_002.lcd		
Method Filename	: LUX Cykle PDA 14.03.2022r.lcm		
Batch Filename	: Cykle cE i cF LUX.lcb		
Vial #	: 1-2	Sample Type	: Unknown
Injection Volume	: 40 uL		
Date Acquired	: 2022-03-22 11:51:49	Acquired by	: System Administrator
Date Processed	: 2022-03-22 14:52:03	Processed by	: System Administrator

<Chromatogram>

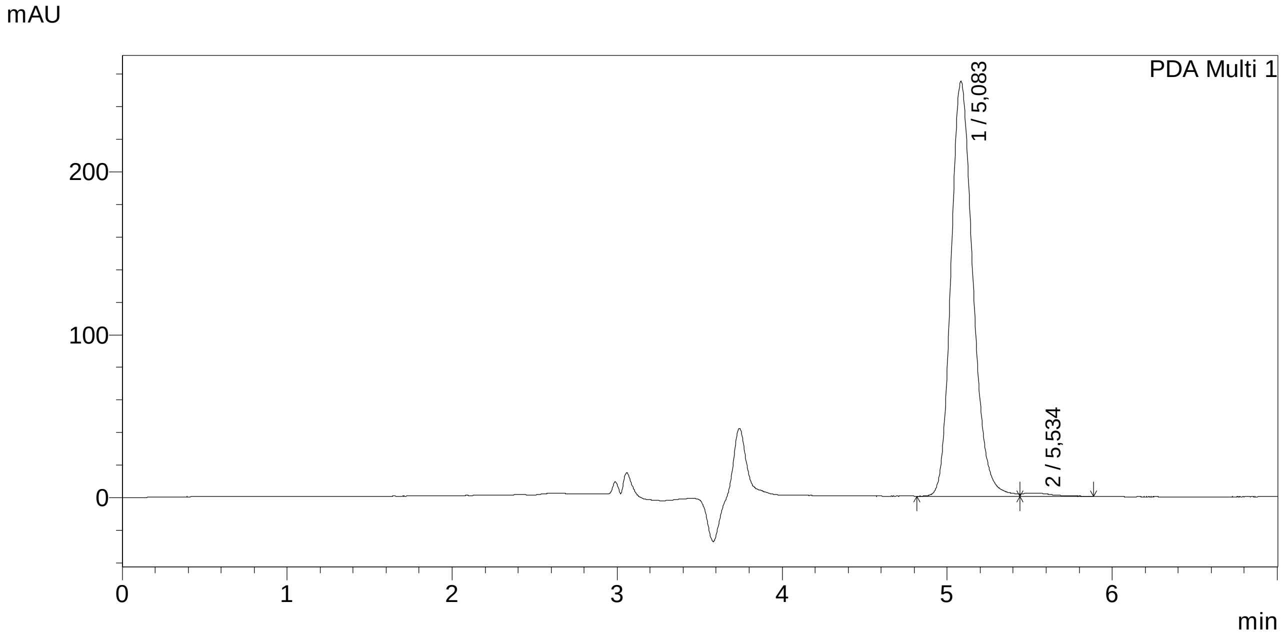

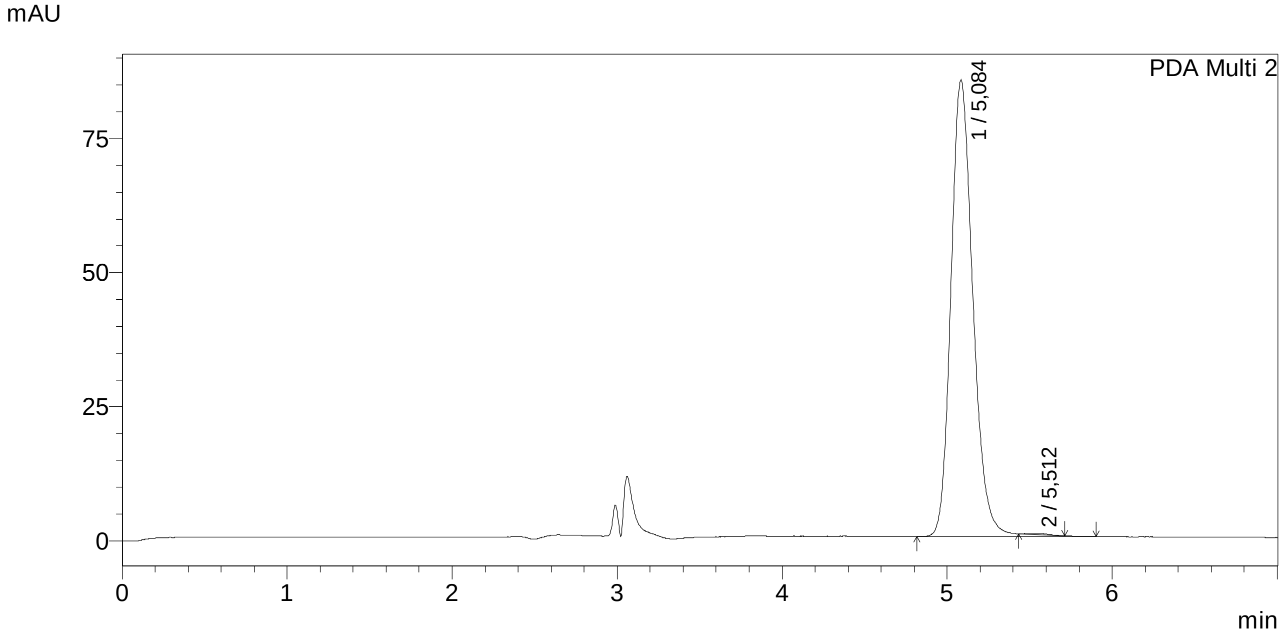

PDA Ch1 220nm

Peak#	Ret. Time	Area	Area%	Height	Height%
1	5,083	2356867	98,993	254400	99,214
2	5,534	23966	1,007	2015	0,786
Total		2380833	100,000	256415	100,000

PDA Ch2 280nm

Peak#	Ret. Time	Area	Area%	Height	Height%
1	5,084	793401	99,719	85095	99,717
2	5,512	2232	0,281	242	0,283
Total		795633	100,000	85337	100,000

(R)-12c : (S)-13c

==== Shimadzu LabSolutions Data Comparison ====

Data1 : C:\LabSolutions\Data\Król\Analizy 10.03.2022r\2022-03-22_1140_cF_r10 B70 t25_002.lcd
Data2 : C:\LabSolutions\Data\Król\Analizy 10.03.2022r\2022-03-22_1140_cE_r10 B70 t25_001.lcd

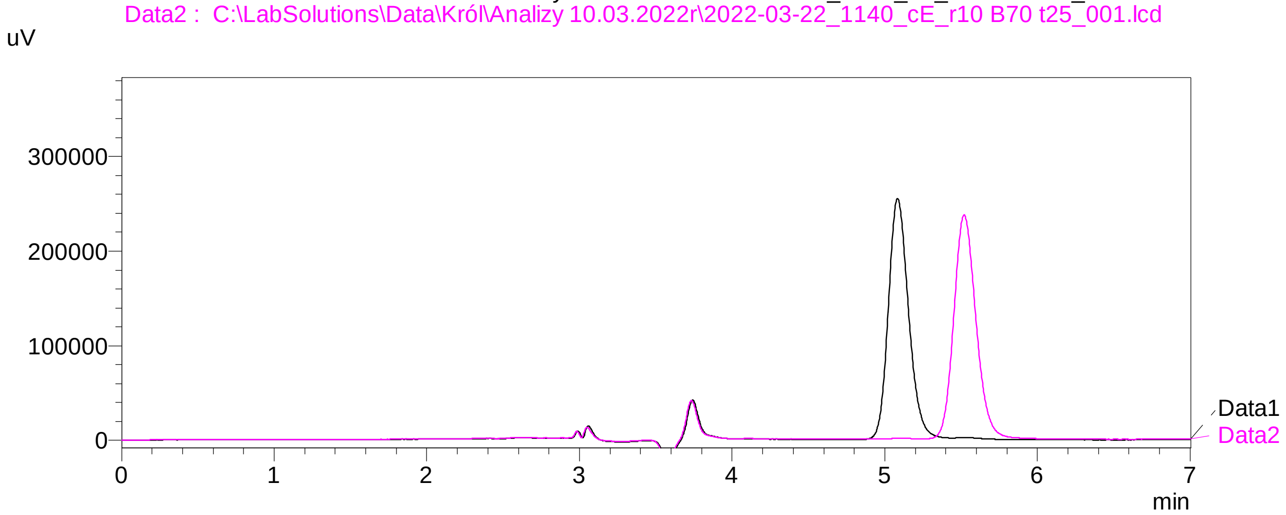

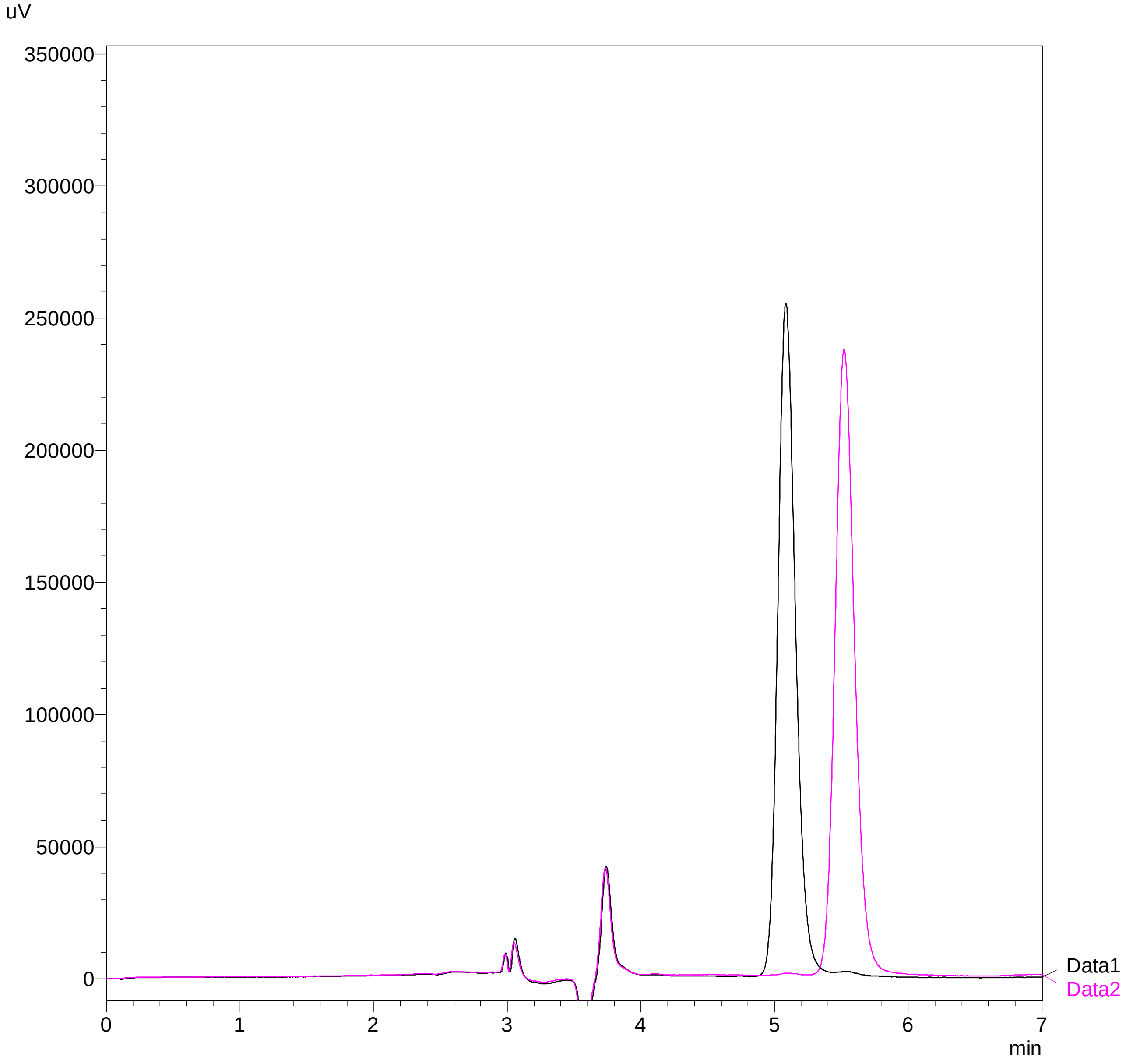

Supplement: Supplementary file 1 [file ijms-24-00517-s001.zip › ijms-2050719-supplementary/HPLC/HPLC.pdf]
