# Supplementary material for: The Synthesis and Absolute Configuration of Enantiomeric Pure (R)- and (S)-3-(piperidin-3-yl)-1H-Indole Derivatives
Source: Int J Mol Sci. 2022 Dec 28;24(1):517. doi: 10.3390/ijms24010517 (PMC9820373; doi:10.3390/ijms24010517)
Supplement: Supplementary file 1 [file ijms-24-00517-s001.zip › ijms-2050719-supplementary/HRMS/HRMS.pdf]

(S)-II

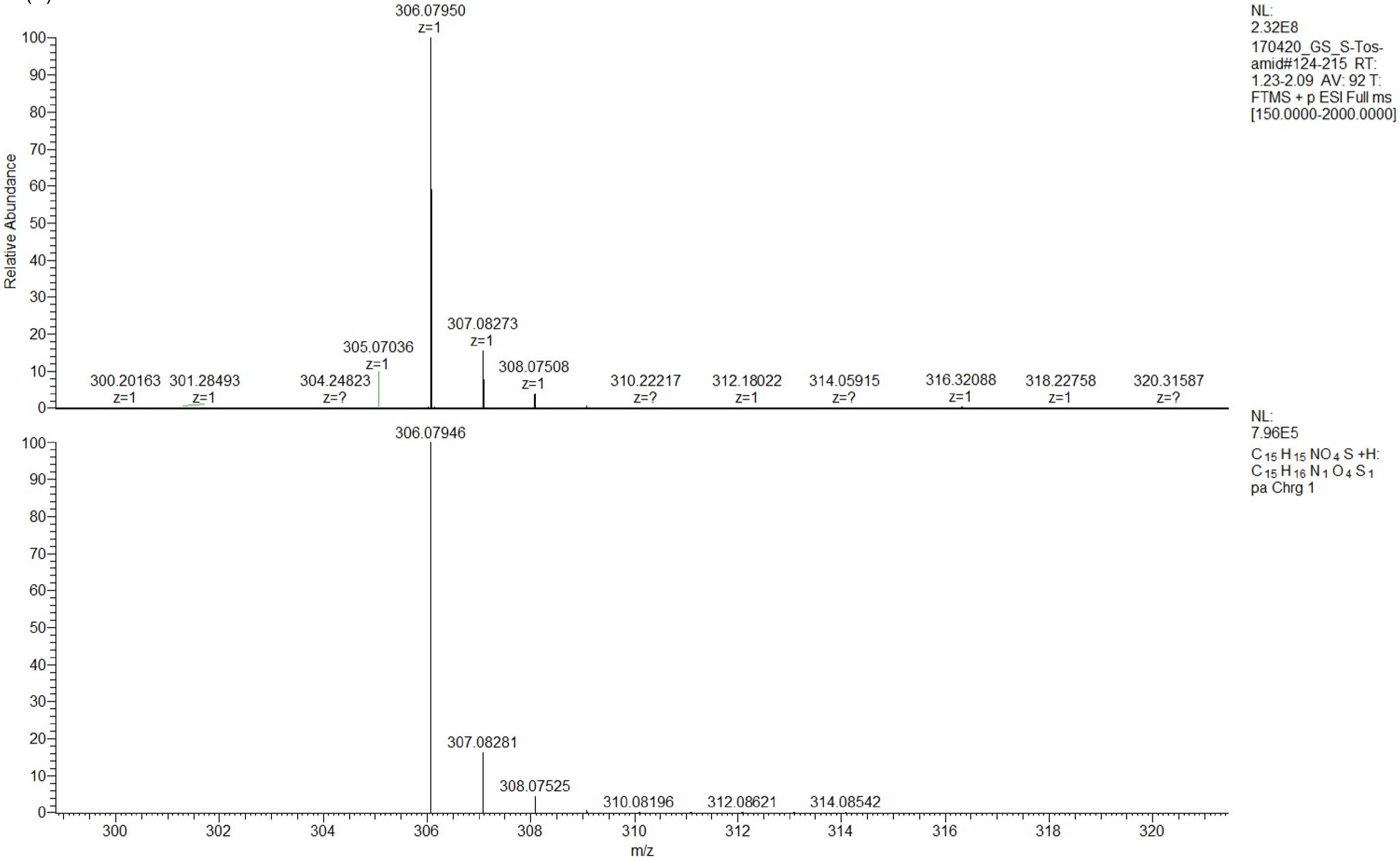
NL: 2.32E8
170420_GS_S-Tos-amid#124-215 RT: 1.23-2.09 AV: 92 T: FTMS + p ESI Full ms [150.0000-2000.0000]
306.07950 z=1
307.08273 z=1
305.07036 z=1
308.07508 z=1
300.20163 z=1
301.28493 z=1
304.24823 z=?
310.22217 z=?
312.18022 z=1
314.05915 z=?
316.32088 z=1
318.22758 z=1
320.31587 z=?
Relative Abundance
NL: 7.96E5
C 15 H 15 NO 4 S +H: C 15 H 16 N 1 O 4 S 1 pa Chrg 1
306.07946
307.08281
308.07525
310.08196
312.08621
314.08542
m/z

(3R,2S)-2a/(3S/2S)-3a

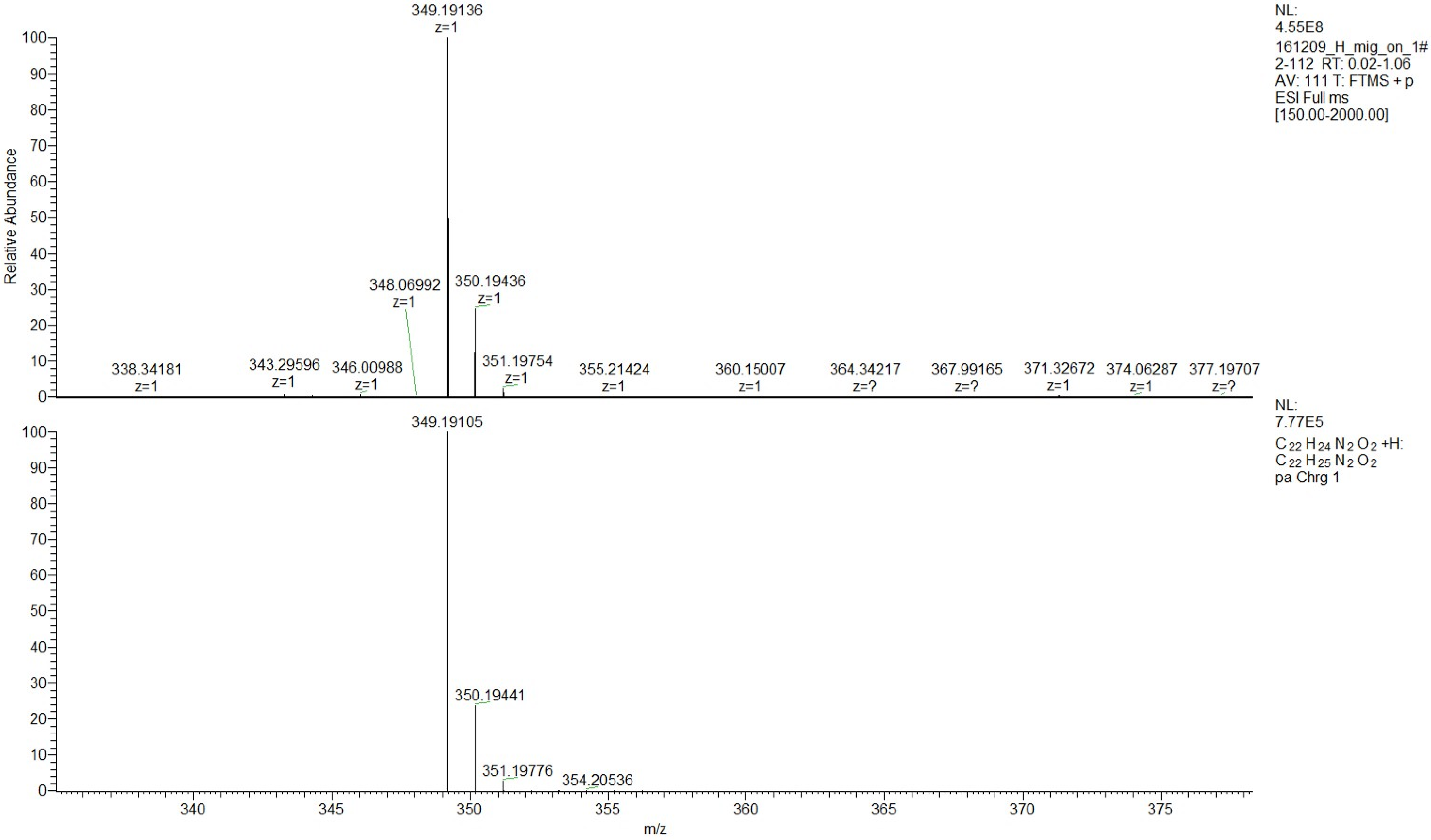

(3R,2R)-4a

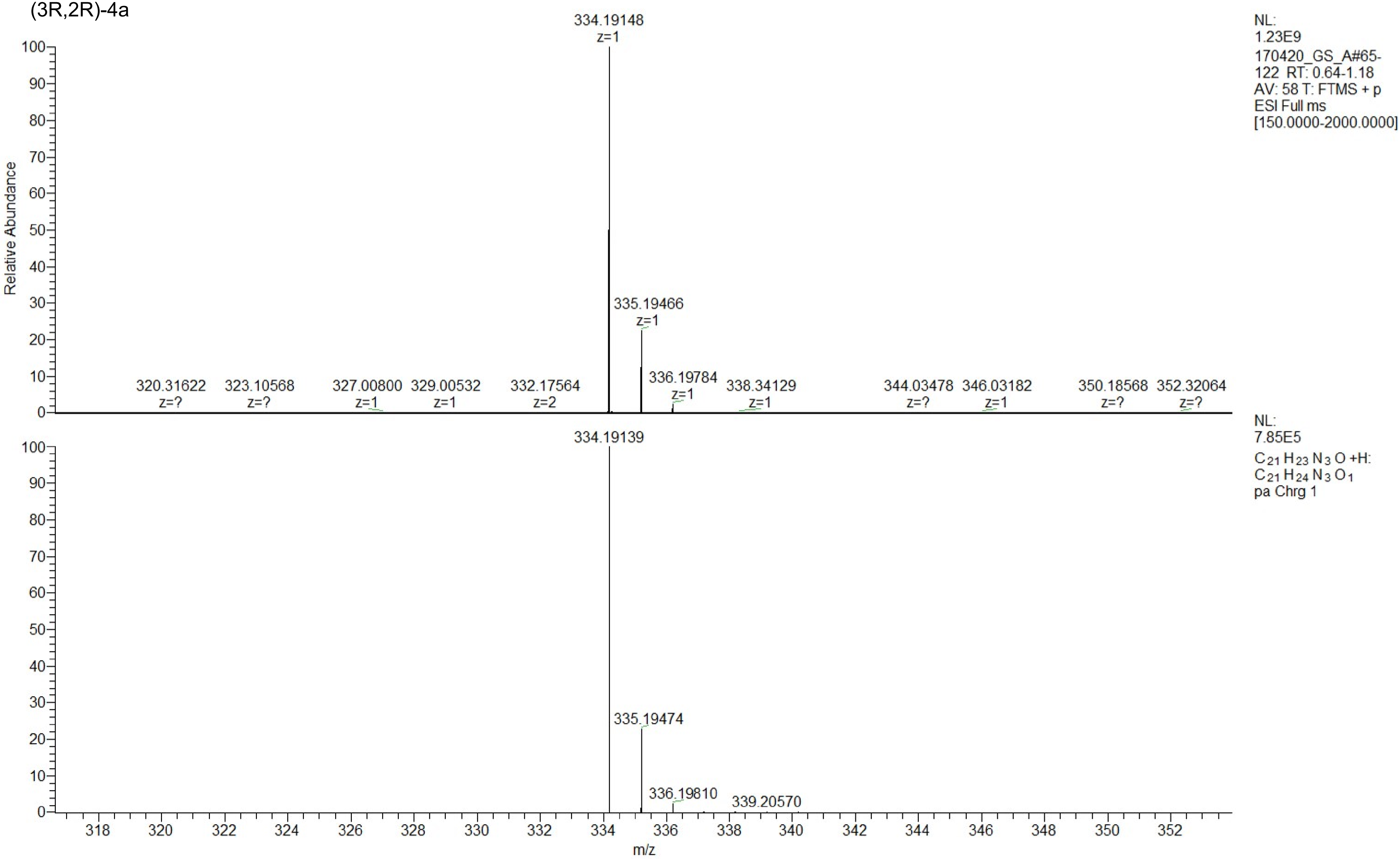

334.19148
z=1
NL:
1.23E9
170420_GS_A#65-122 RT: 0.64-1.18
AV: 58 T: FTMS + p ESI Full ms [150.0000-2000.0000]
Relative Abundance
335.19466
z=1
336.19784
z=1
320.31622 z=?
323.10568 z=?
327.00800 z=1
329.00532 z=1
332.17564 z=2
338.34129 z=1
344.03478 z=?
346.03182 z=1
350.18568 z=?
352.32064 z=?
334.19139
NL:
7.85E5
C21 H23 N3 O +H:
C21 H24 N3 O1
pa Chrg 1
335.19474
336.19810
339.20570
m/z

(3S,2R)-5a

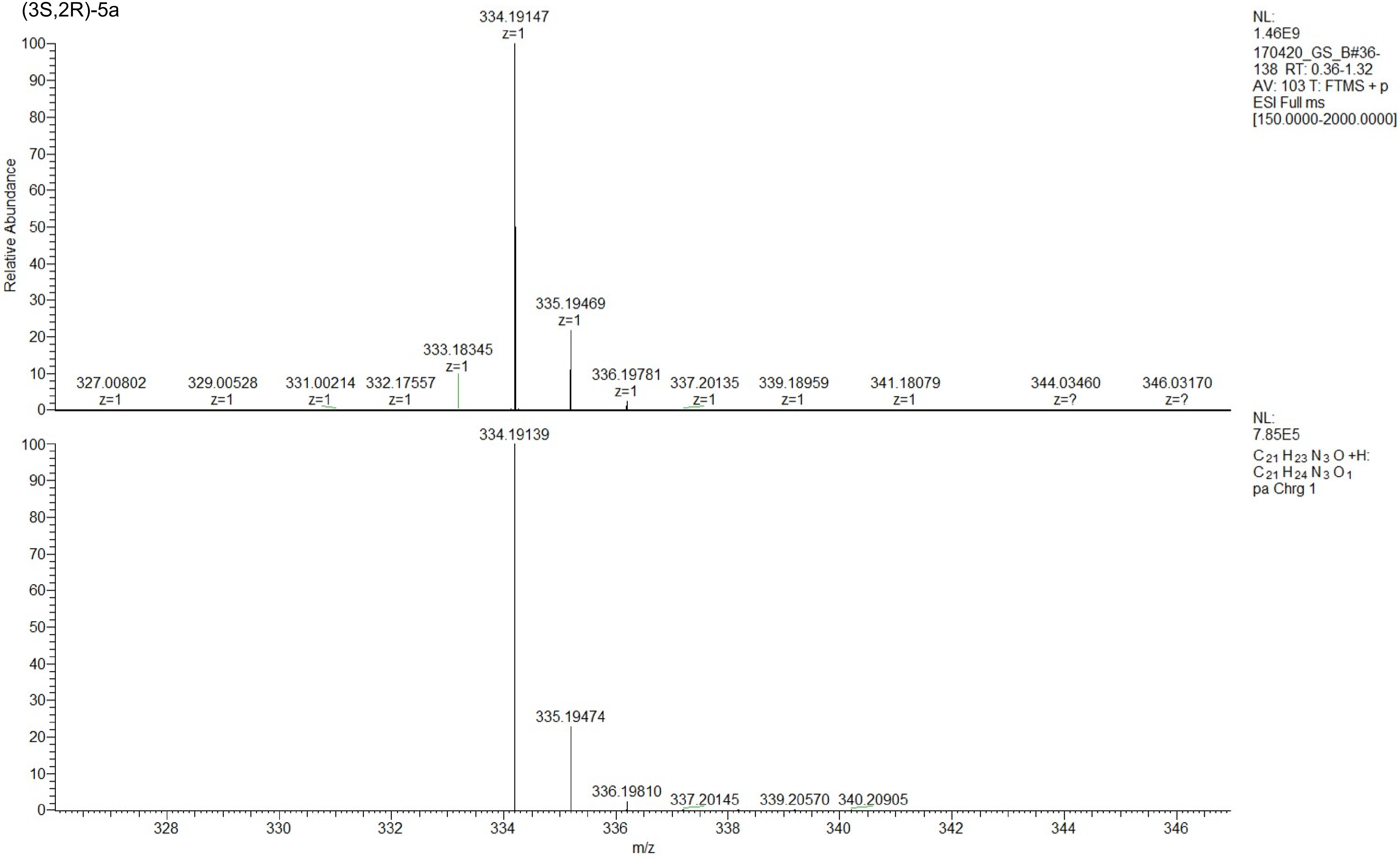

334.19147 z=1
335.19469 z=1
333.18345 z=1
327.00802 z=1
329.00528 z=1
331.00214 z=1
332.17557 z=1
336.19781 z=1
337.20135 z=1
339.18959 z=1
341.18079 z=1
344.03460 z=?
346.03170 z=?
NL: 1.46E9
170420_GS_B#36-138 RT: 0.36-1.32 AV: 103 T: FTMS + p ESI Full ms [150.0000-2000.0000]
334.19139
335.19474
336.19810
337.20145
339.20570
340.20905
NL: 7.85E5
C 21 H 23 N 3 O +H: C 21 H 24 N 3 O 1 pa Chrg 1
Relative Abundance
m/z

(3R,2R)-6b

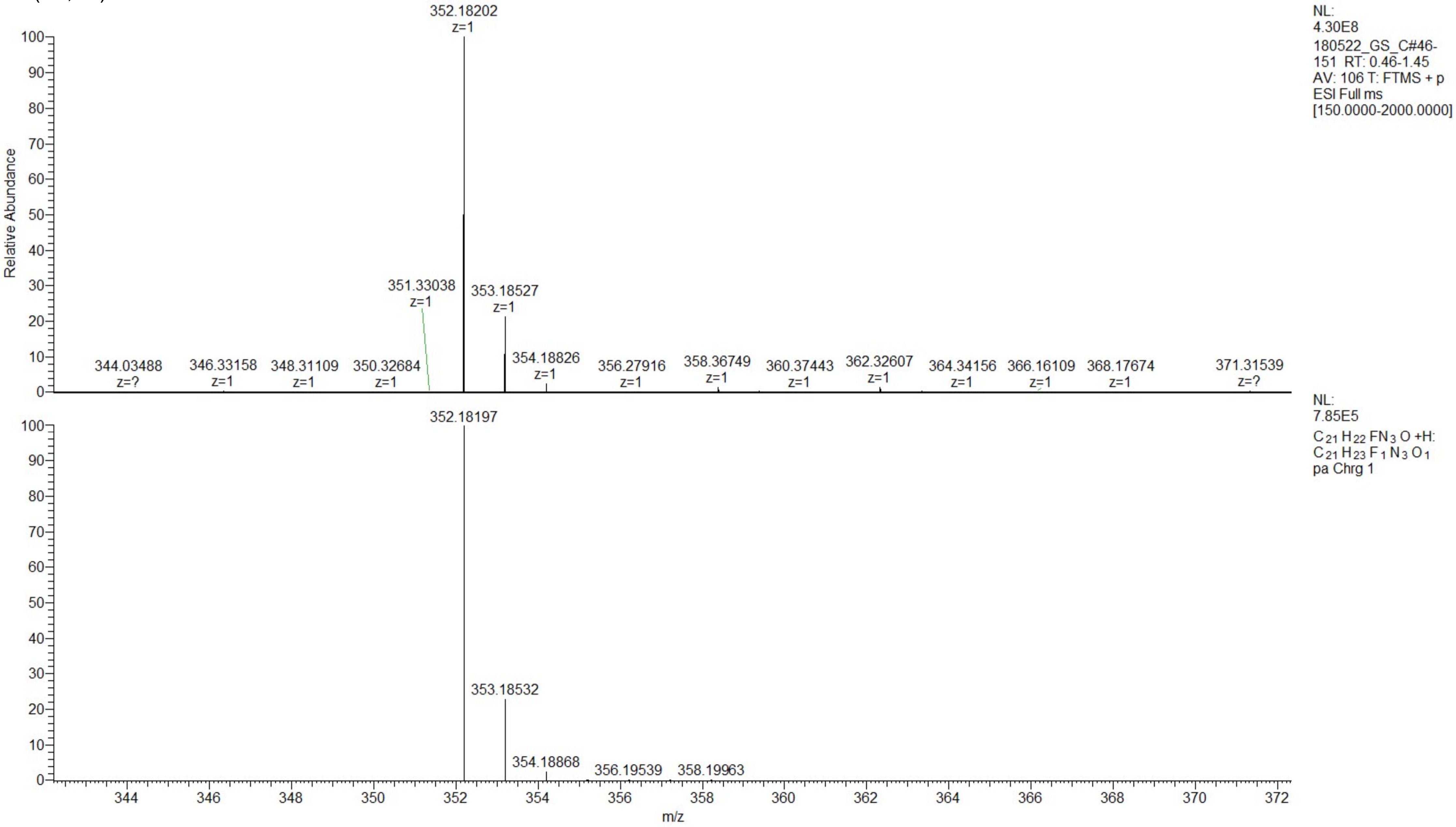

352.18202
z=1
NL:
4.30E8
180522_GS_C#46-151 RT: 0.46-1.45
AV: 106 T: FTMS + p ESI Full ms [150.0000-2000.0000]
Relative Abundance
351.33038
z=1
353.18527
z=1
354.18826
z=1
344.03488
z=?
346.33158
z=1
348.31109
z=1
350.32684
z=1
356.27916
z=1
358.36749
z=1
360.37443
z=1
362.32607
z=1
364.34156
z=1
366.16109
z=1
368.17674
z=1
371.31539
z=?
NL:
7.85E5
C 21 H 22 FN 3 O +H:
C 21 H 23 F 1 N 3 O 1
pa Chrg 1
352.18197
353.18532
354.18868
356.19539
358.19963
m/z

(3S,2R)-7b

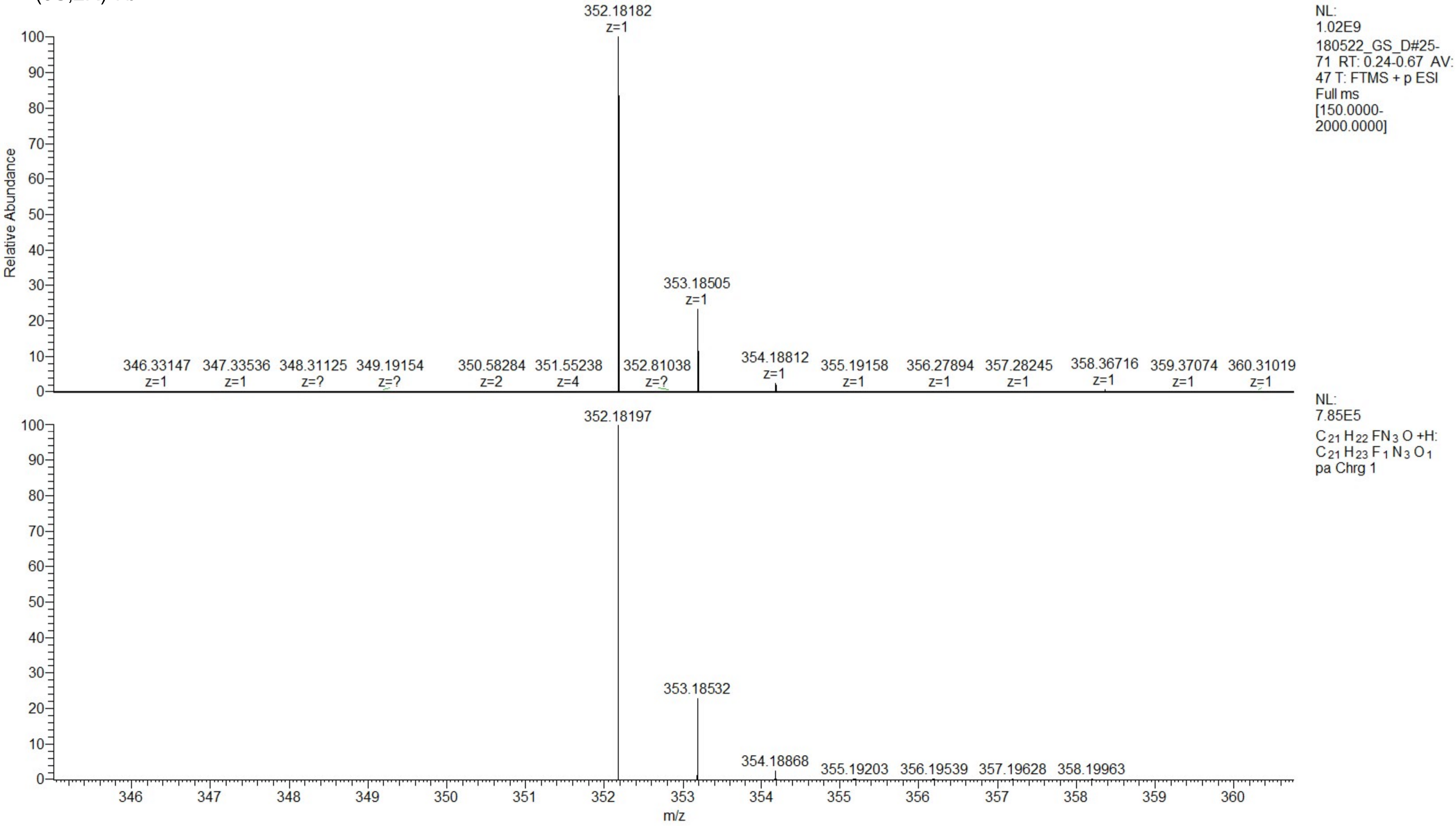
NL:
1.02E9
180522_GS_D#25-71 RT: 0.24-0.67 AV: 47 T: FTMS + p ESI Full ms [150.0000-2000.0000]
352.18182 z=1
353.18505 z=1
346.33147 z=1
347.33536 z=1
348.31125 z=?
349.19154 z=?
350.58284 z=2
351.55238 z=4
352.81038 z=?
354.18812 z=1
355.19158 z=1
356.27894 z=1
357.28245 z=1
358.36716 z=1
359.37074 z=1
360.31019 z=1
Relative Abundance
NL:
7.85E5
C 21 H 22 FN 3 O +H: C 21 H 23 F 1 N 3 O 1 pa Chrg 1
352.18197
353.18532
354.18868
355.19203
356.19539
357.19628
358.19963
m/z

(3R,2R)-8c

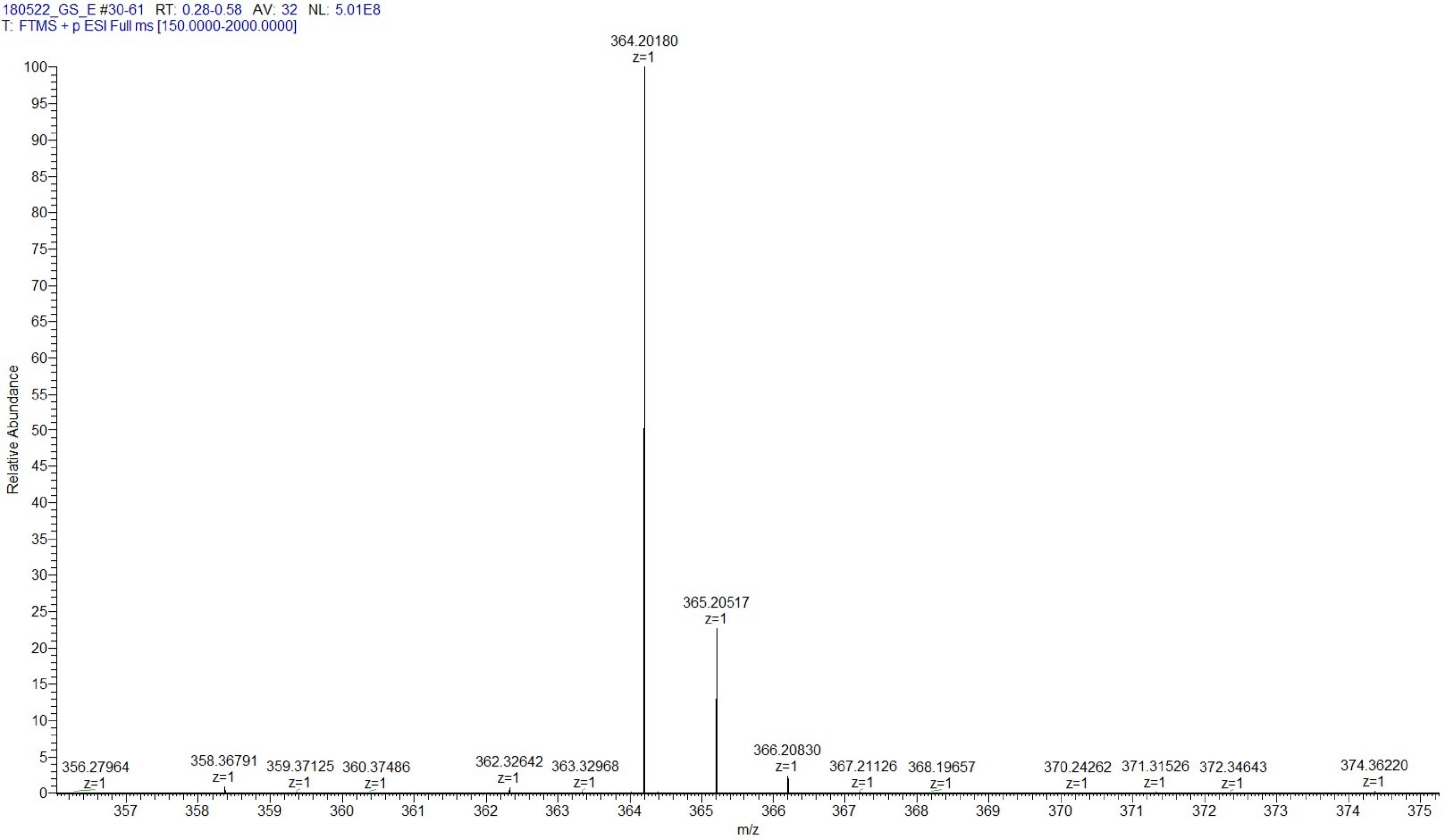
180522_GS_E #30-61 RT: 0.28-0.58 AV: 32 NL: 5.01E8
T: FTMS + p ESI Full ms [150.0000-2000.0000]
364.20180 z=1
365.20517 z=1
366.20830 z=1
356.27964 z=1
358.36791 z=1
359.37125 z=1
360.37486 z=1
362.32642 z=1
363.32968 z=1
367.21126 z=1
368.19657 z=1
370.24262 z=1
371.31526 z=1
372.34643 z=1
374.36220 z=1
Relative Abundance
m/z

(3S,2R)-9c

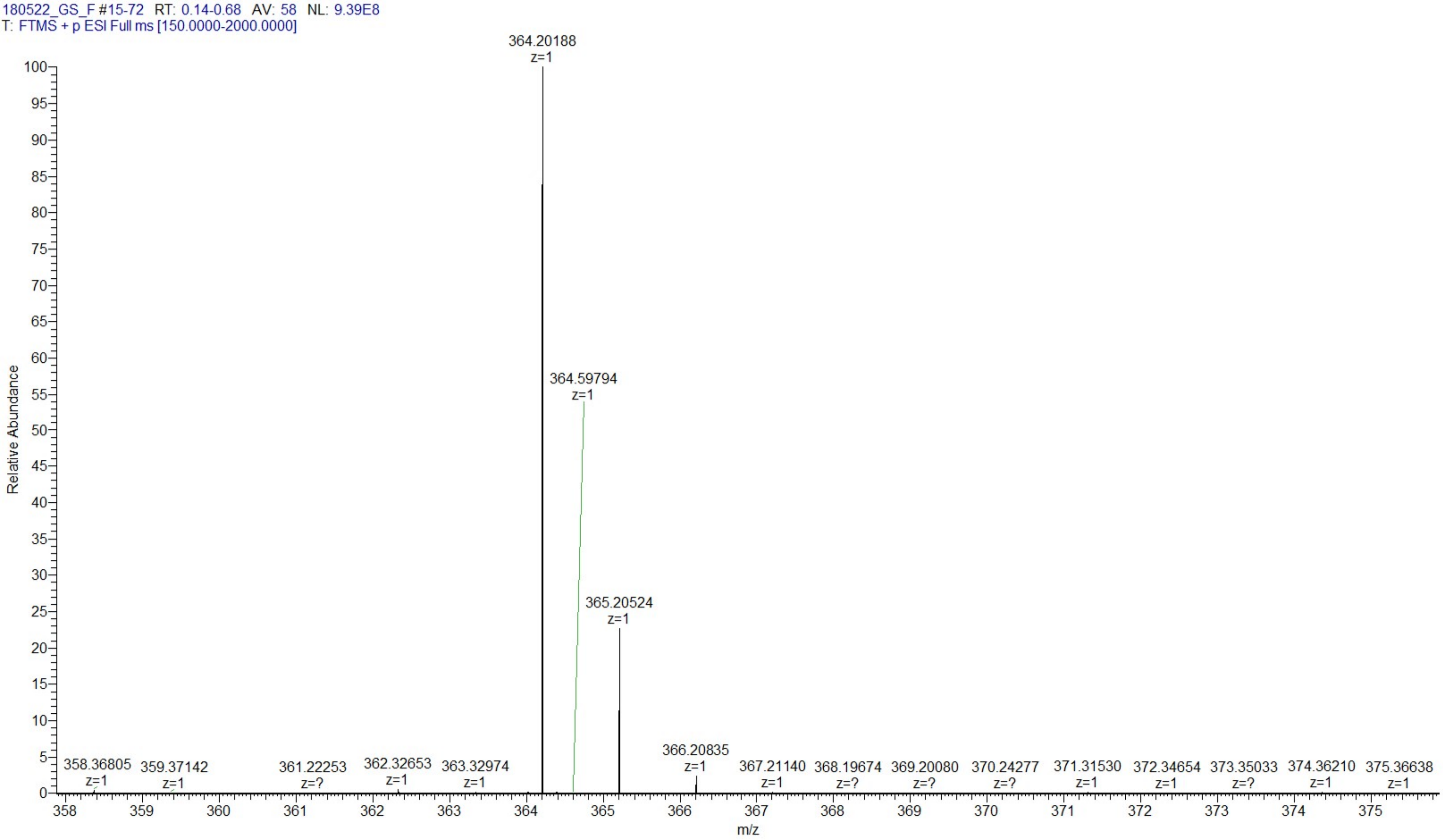
180522_GS_F #15-72 RT: 0.14-0.68 AV: 58 NL: 9.39E8
T: FTMS + p ESI Full ms [150.0000-2000.0000]
364.20188 z=1
364.59794 z=1
365.20524 z=1
366.20835 z=1
358.36805 z=1
359.37142 z=1
361.22253 z=?
362.32653 z=1
363.32974 z=1
367.21140 z=1
368.19674 z=?
369.20080 z=?
370.24277 z=?
371.31530 z=1
372.34654 z=1
373.35033 z=?
374.36210 z=1
375.36638 z=1
Relative Abundance
m/z

(R)-10a

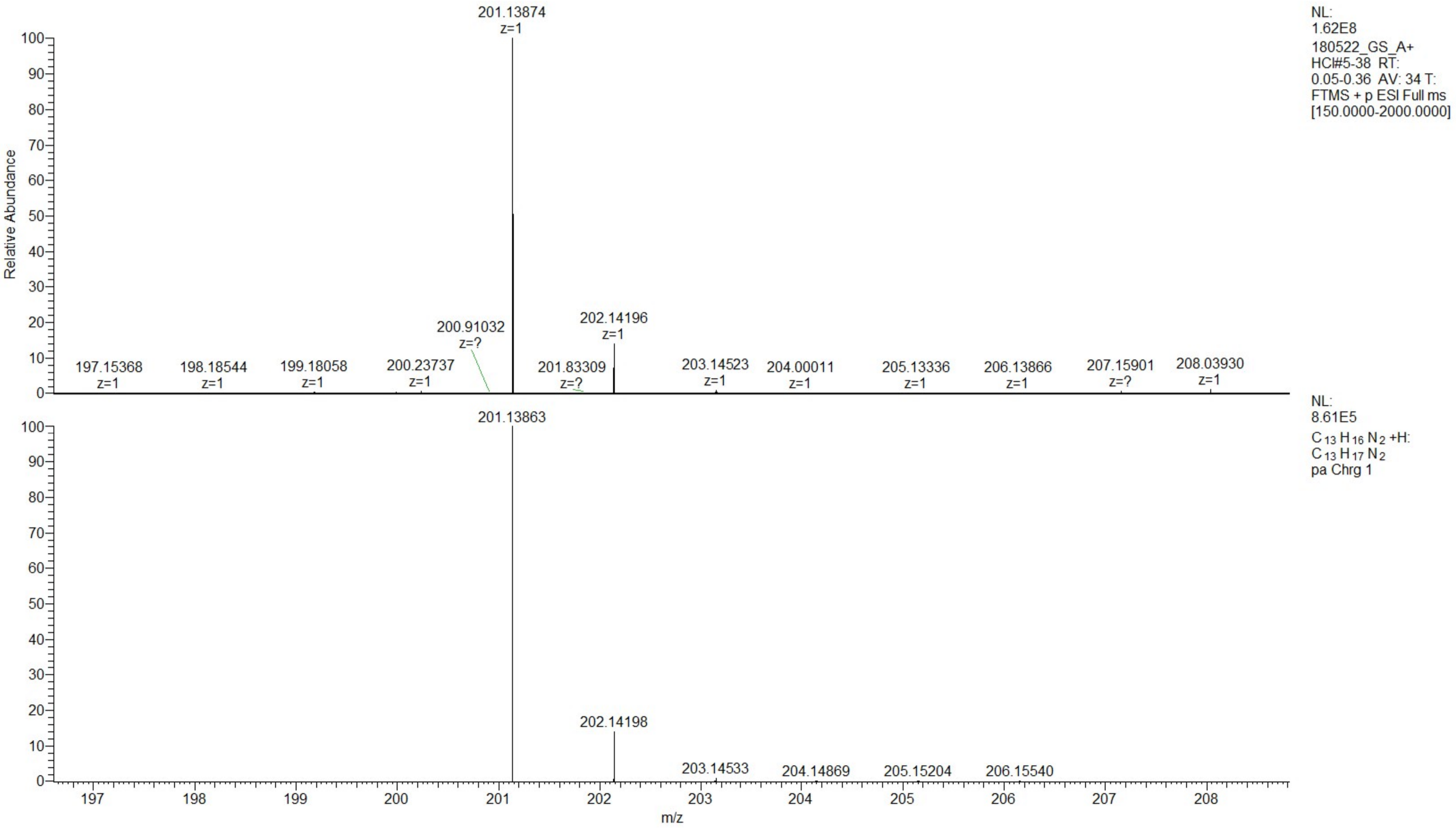

201.13874
z=1
202.14196
z=1
200.91032
z=?
197.15368
z=1
198.18544
z=1
199.18058
z=1
200.23737
z=1
201.83309
z=?
203.14523
z=1
204.00011
z=1
205.13336
z=1
206.13866
z=1
207.15901
z=?
208.03930
z=1
NL:
1.62E8
180522_GS_A+
HCl#5-38 RT:
0.05-0.36 AV: 34 T:
FTMS + p ESI Full ms
[150.0000-2000.0000]
201.13863
202.14198
203.14533
204.14869
205.15204
206.15540
NL:
8.61E5
C 13 H 16 N 2 +H:
C 13 H 17 N 2
pa Chrg 1
Relative Abundance
m/z

(S)-11a

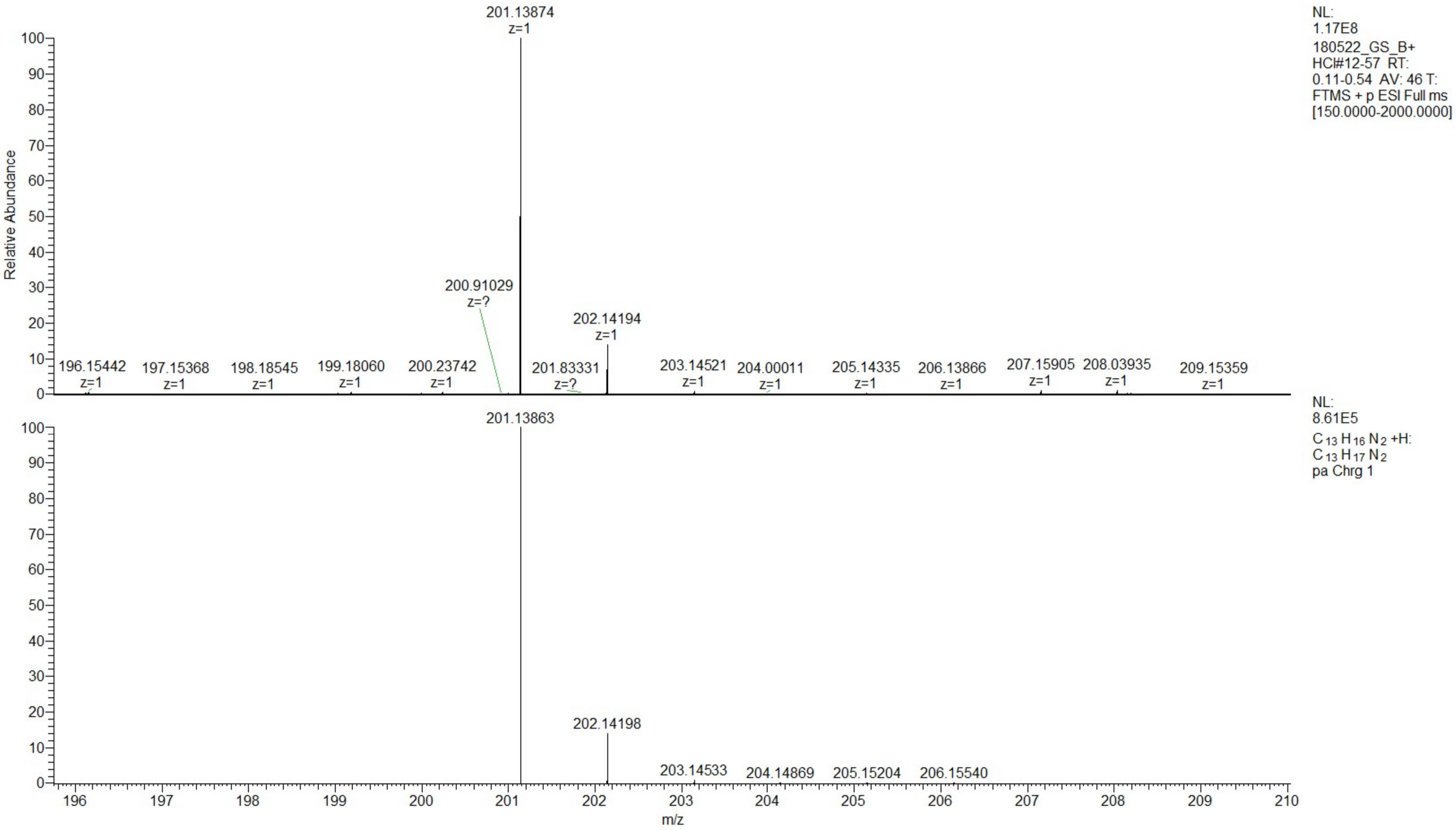

201.13874
z=1
NL:
1.17E8
180522_GS_B+
HCl#12-57 RT:
0.11-0.54 AV: 46 T:
FTMS + p ESI Full ms
[150.0000-2000.0000]
Relative Abundance
200.91029
z=?
202.14194
z=1
196.15442
z=1
197.15368
z=1
198.18545
z=1
199.18060
z=1
200.23742
z=1
201.83331
z=?
203.14521
z=1
204.00011
z=1
205.14335
z=1
206.13866
z=1
207.15905
z=1
208.03935
z=1
209.15359
z=1
NL:
8.61E5
C 13 H 16 N 2 +H:
C 13 H 17 N 2
pa Chrg 1
201.13863
202.14198
203.14533
204.14869
205.15204
206.15540
m/z

(R)-10b

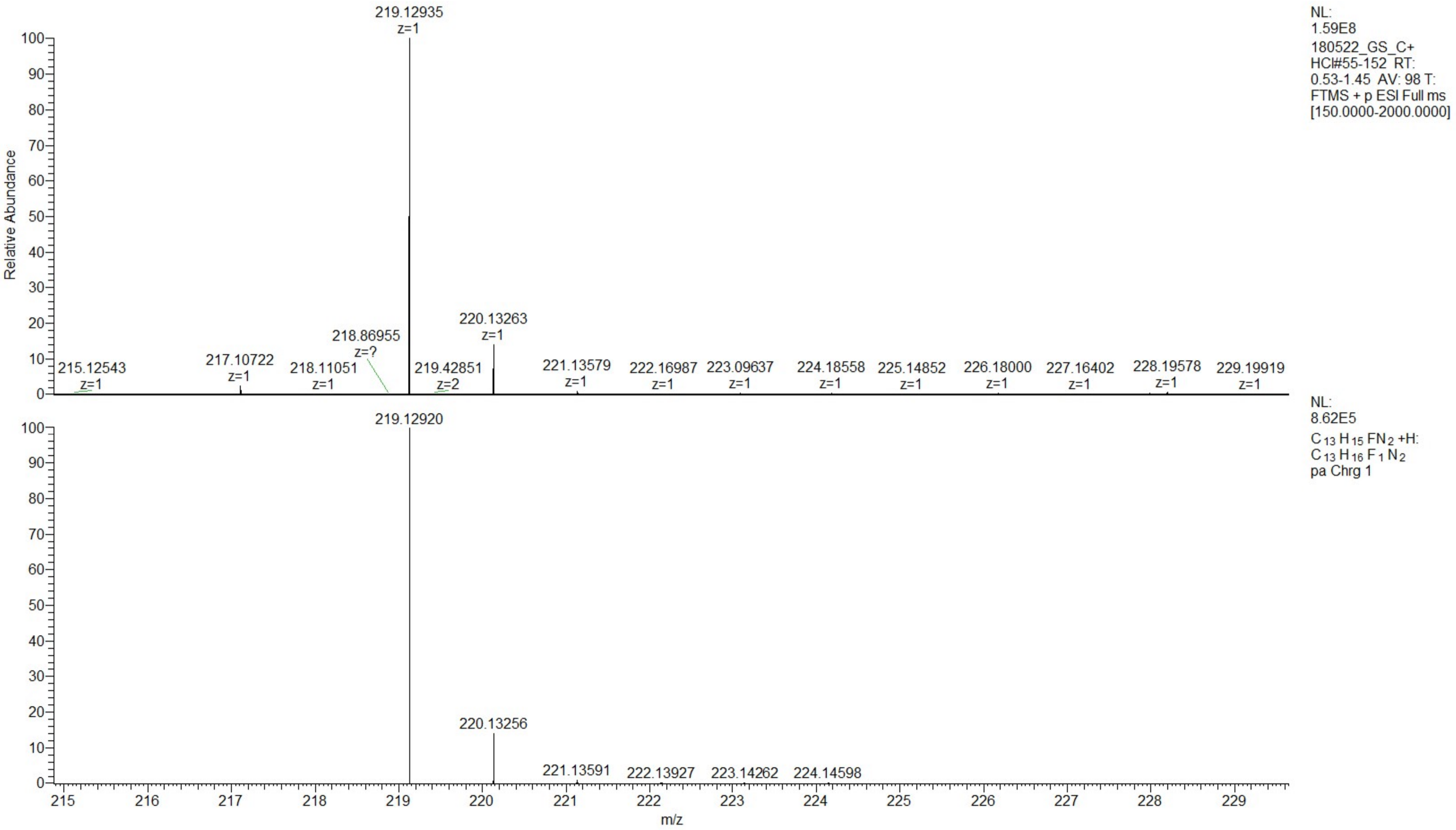

219.12935
z=1
220.13263
z=1
218.86955
z=?
215.12543
z=1
217.10722
z=1
218.11051
z=1
219.42851
z=2
221.13579
z=1
222.16987
z=1
223.09637
z=1
224.18558
z=1
225.14852
z=1
226.18000
z=1
227.16402
z=1
228.19578
z=1
229.19919
z=1
NL:
1.59E8
180522_GS_C+
HCl#55-152 RT:
0.53-1.45 AV: 98 T:
FTMS + p ESI Full ms
[150.0000-2000.0000]
219.12920
220.13256
221.13591
222.13927
223.14262
224.14598
NL:
8.62E5
C 13 H 15 FN 2 +H:
C 13 H 16 F 1 N 2
pa Chrg 1
Relative Abundance
m/z

(S)-11b

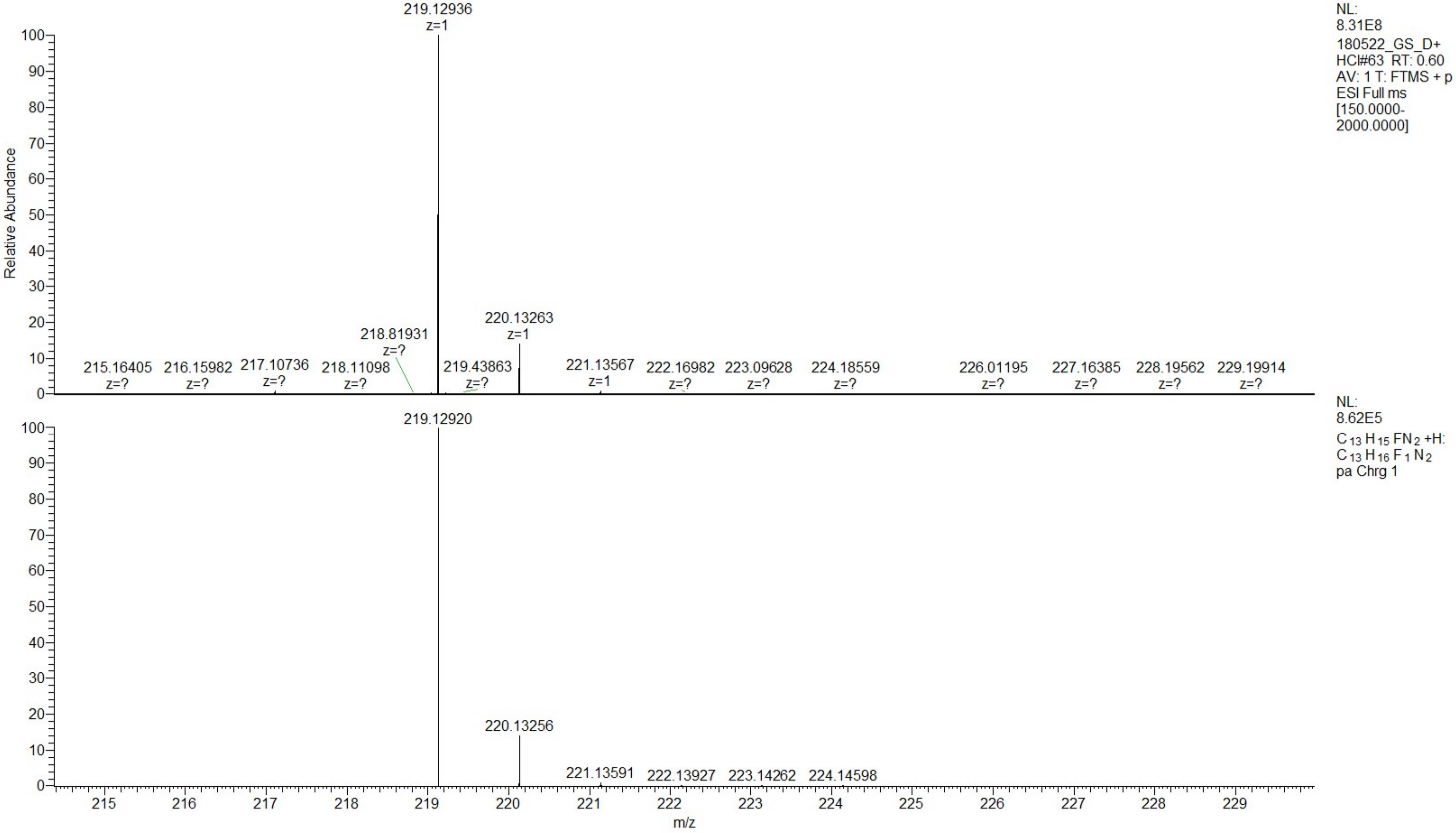

219.12936
z=1
NL:
8.31E8
180522_GS_D+
HCl#63 RT: 0.60
AV: 1 T: FTMS + p
ESI Full ms
[150.0000-
2000.0000]
Relative Abundance
218.81931
z=?
220.13263
z=1
215.16405
z=?
216.15982
z=?
217.10736
z=?
218.11098
z=?
219.43863
z=?
221.13567
z=1
222.16982
z=?
223.09628
z=?
224.18559
z=?
226.01195
z=?
227.16385
z=?
228.19562
z=?
229.19914
z=?
NL:
8.62E5
C 13 H 15 FN 2 +H:
C 13 H 16 F 1 N 2
pa Chrg 1
219.12920
220.13256
221.13591
222.13927
223.14262
224.14598
m/z

(R)-10c

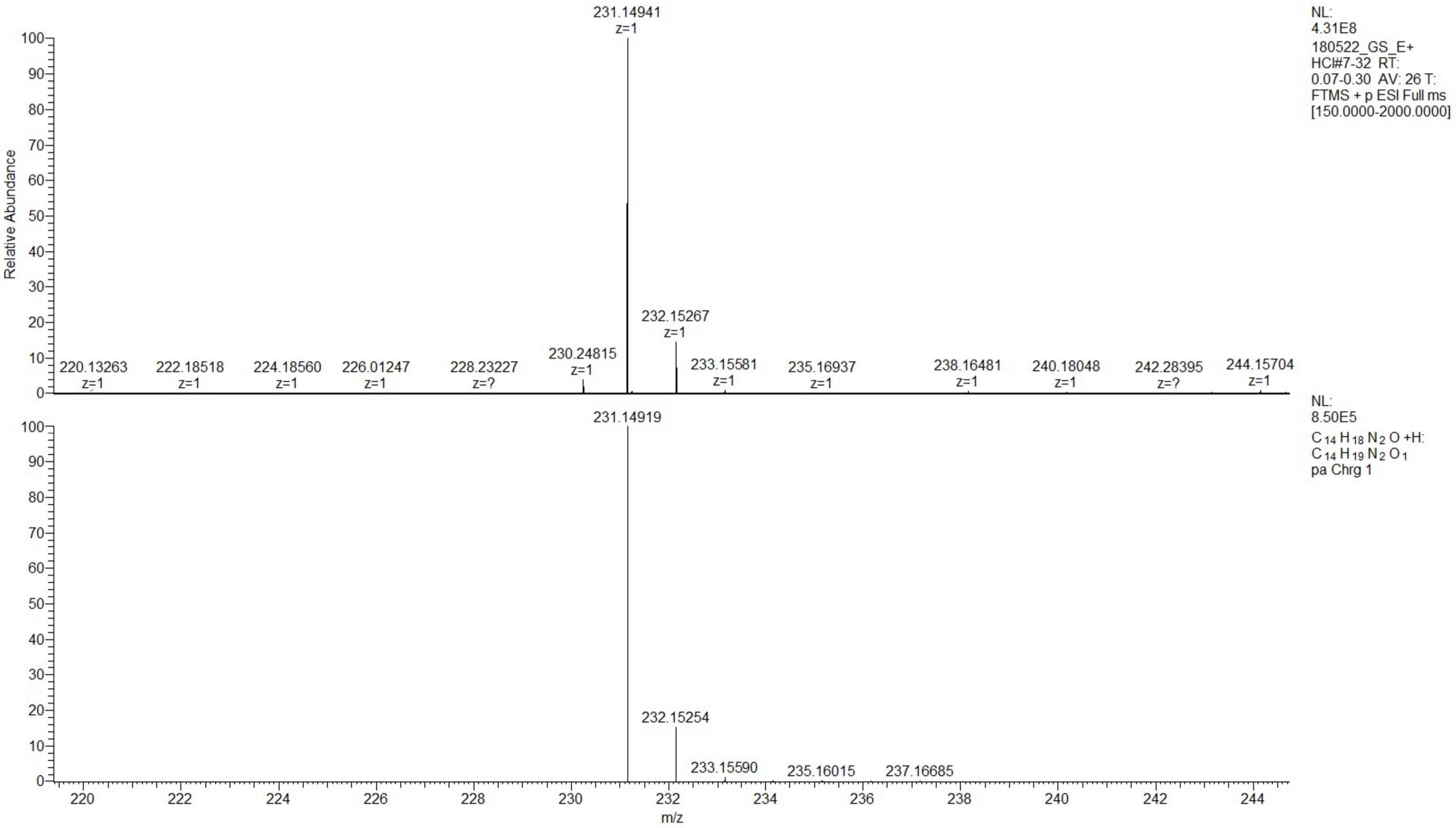
231.14941
z=1
NL:
4.31E8
180522_GS_E+
HCl#7-32 RT:
0.07-0.30 AV: 26 T:
FTMS + p ESI Full ms
[150.0000-2000.0000]
Relative Abundance
232.15267
z=1
230.24815
z=1
220.13263
z=1
222.18518
z=1
224.18560
z=1
226.01247
z=1
228.23227
z=?
233.15581
z=1
235.16937
z=1
238.16481
z=1
240.18048
z=1
242.28395
z=?
244.15704
z=1
NL:
8.50E5
C 14 H 18 N 2 O +H:
C 14 H 19 N 2 O 1
pa Chrg 1
231.14919
232.15254
233.15590
235.16015
237.16685
m/z

(S)-11c

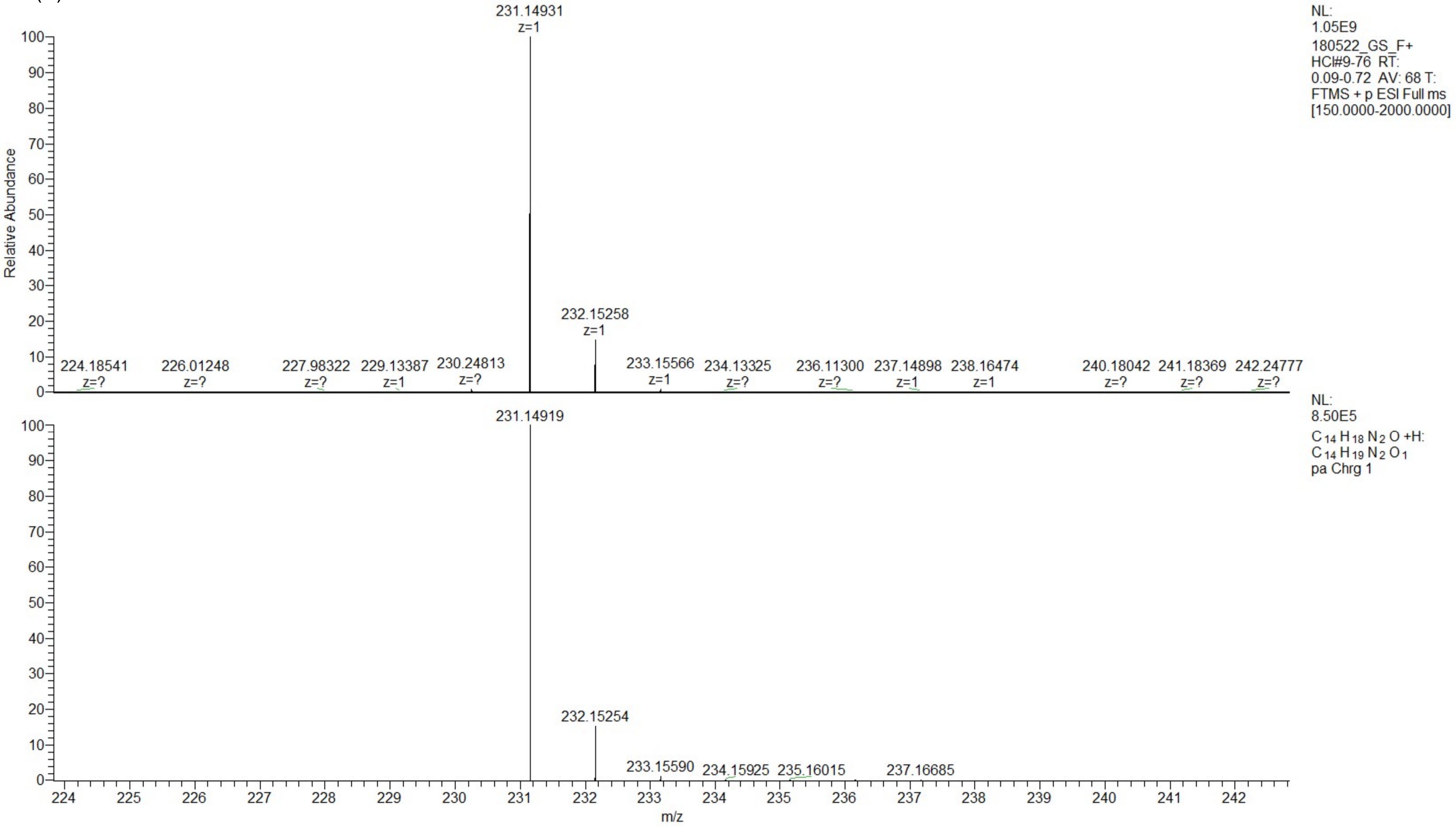
231.14931
z=1
NL:
1.05E9
180522_GS_F+
HCl#9-76 RT:
0.09-0.72 AV: 68 T:
FTMS + p ESI Full ms
[150.0000-2000.0000]
Relative Abundance
232.15258
z=1
224.18541
z=?
226.01248
z=?
227.98322
z=?
229.13387
z=1
230.24813
z=?
233.15566
z=1
234.13325
z=?
236.11300
z=?
237.14898
z=1
238.16474
z=1
240.18042
z=?
241.18369
z=?
242.24777
z=?
NL:
8.50E5
C 14 H 18 N 2 O +H:
C 14 H 19 N 2 O 1
pa Chrg 1
231.14919
232.15254
233.15590
234.15925
235.16015
237.16685
m/z

(R)-12c/(S)-13c

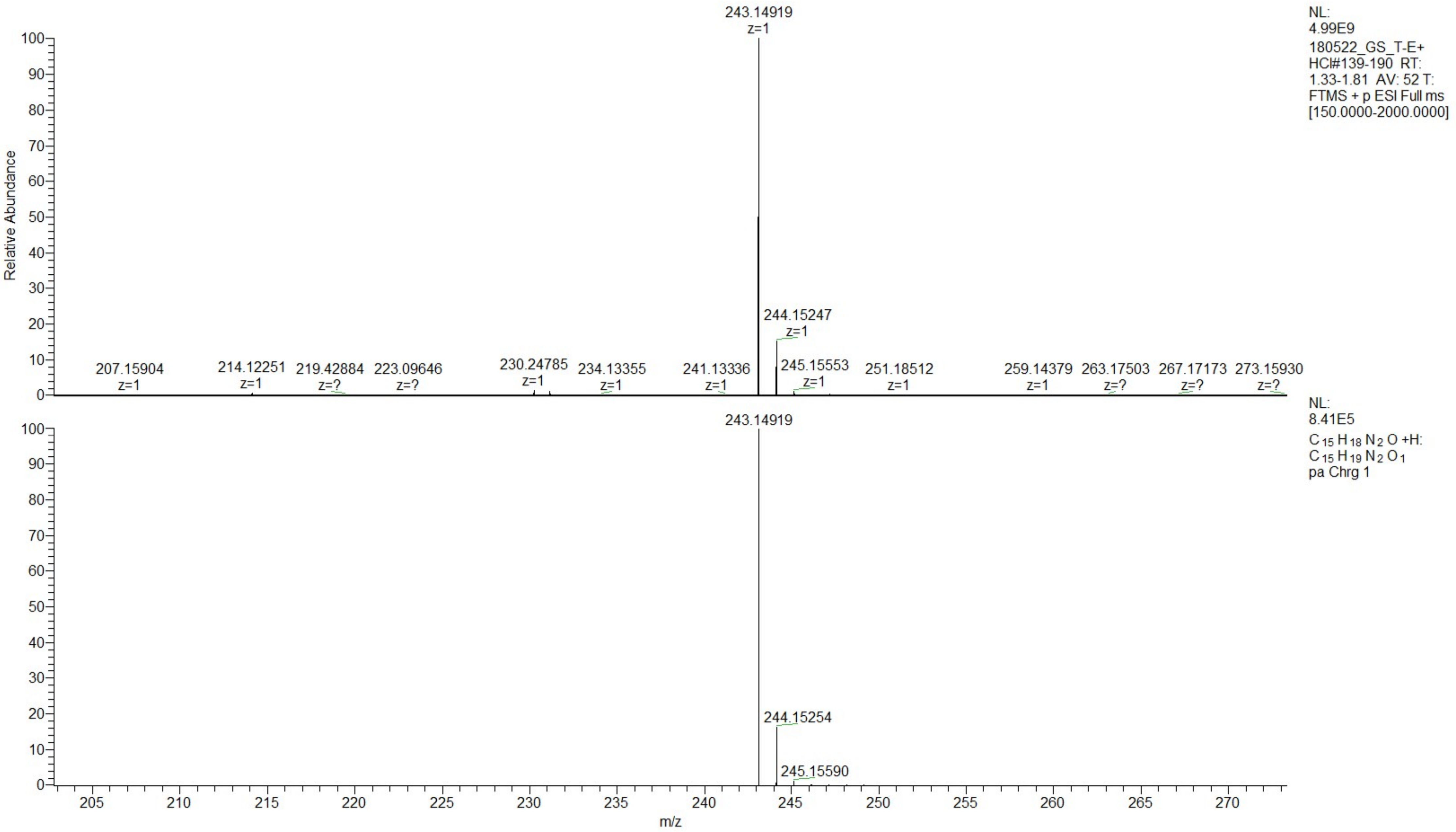
243.14919
z=1
NL:
4.99E9
180522_GS_T-E+
HCl#139-190 RT:
1.33-1.81 AV: 52 T:
FTMS + p ESI Full ms
[150.0000-2000.0000]
Relative Abundance
207.15904 z=1
214.12251 z=1
219.42884 z=?
223.09646 z=?
230.24785 z=1
234.13355 z=1
241.13336 z=1
244.15247 z=1
245.15553 z=1
251.18512 z=1
259.14379 z=1
263.17503 z=?
267.17173 z=?
273.15930 z=?
NL:
8.41E5
C 15 H 18 N 2 O +H:
C 15 H 19 N 2 O 1
pa Chrg 1
243.14919
244.15254
245.15590
m/z
