# Supplementary material for: The Synthesis and Absolute Configuration of Enantiomeric Pure (R)- and (S)-3-(piperidin-3-yl)-1H-Indole Derivatives
Source: Int J Mol Sci. 2022 Dec 28;24(1):517. doi: 10.3390/ijms24010517 (PMC9820373; doi:10.3390/ijms24010517)
Supplement: Supplementary file 1 [file ijms-24-00517-s001.zip › ijms-2050719-supplementary/NMR/NMR.pdf]

(S)-II

3537-1H

S-Tos-amid

Current Data Parameters
NAME 3537
EXPNO 1
PROCNO 1

F2 - Acquisition Parameters
Date_ 20170217
PROBHD 5 mm PABBI 1H/
PULPROG zg30
SOLVENT CDCl3
TE 298.2 K
SWH 10000.000 Hz
AQ 3.2767999 sec
NS 8
FIDRES 0.152588 Hz
D1 0 sec

======== CHANNEL f1 =======
SFO1 500.2030889 MHz
NUC1 1H
P1 8.00 use
PLW1 12.69999981 W

F2 - Processing parameters
SI 65536
LB 0 Hz

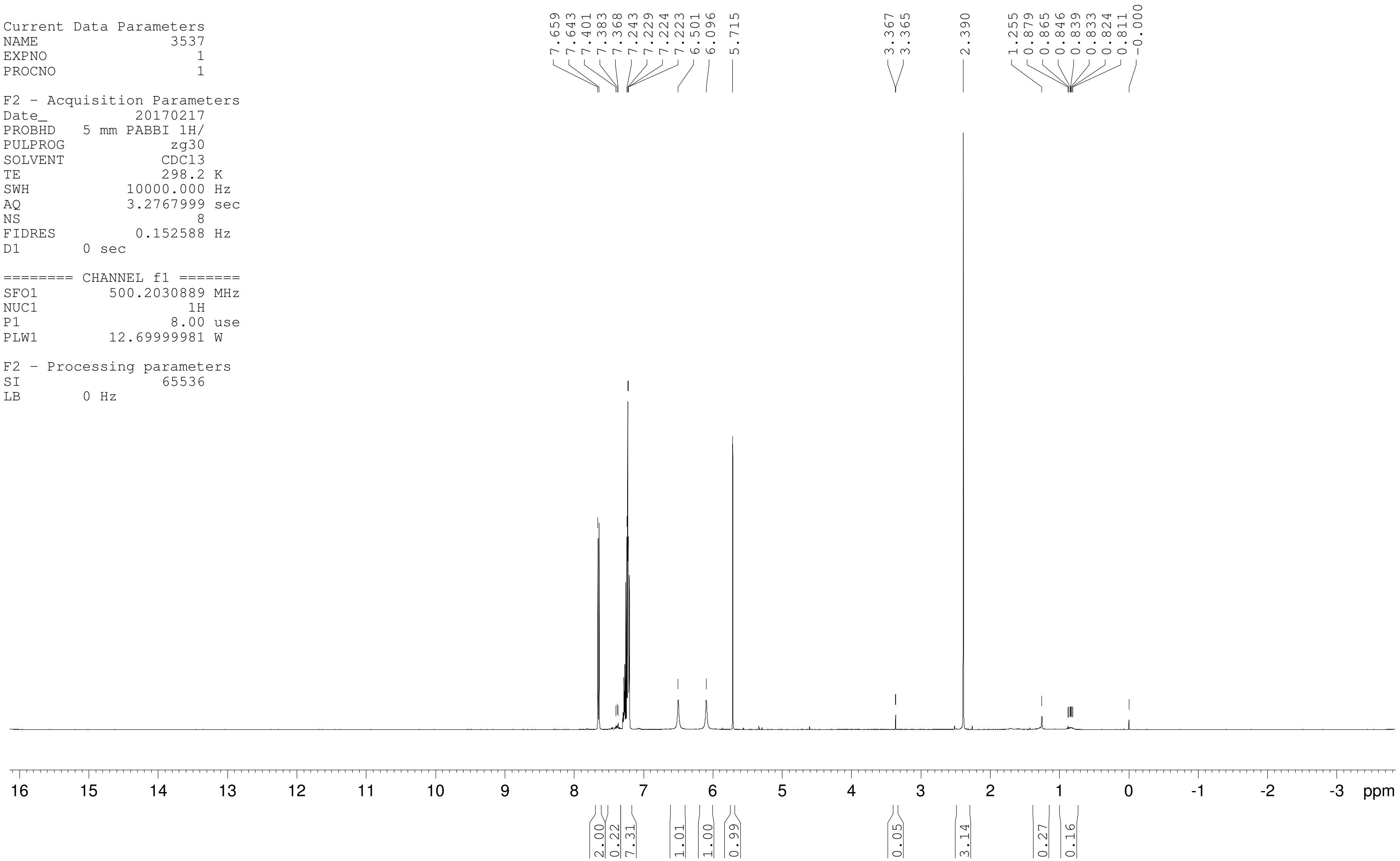

3537-1H
S-Tos-amid

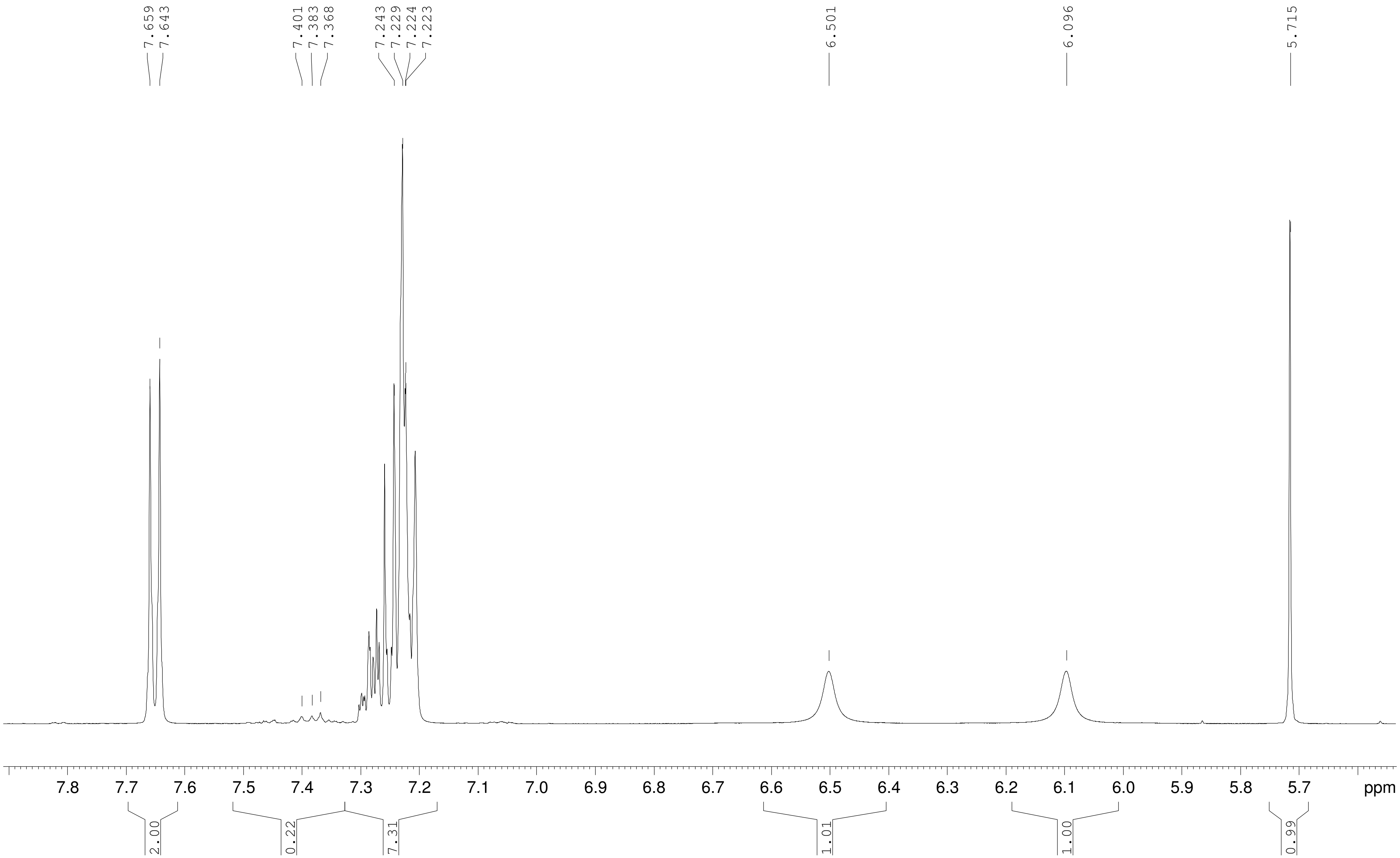

3537-1H
S-Tos-amid

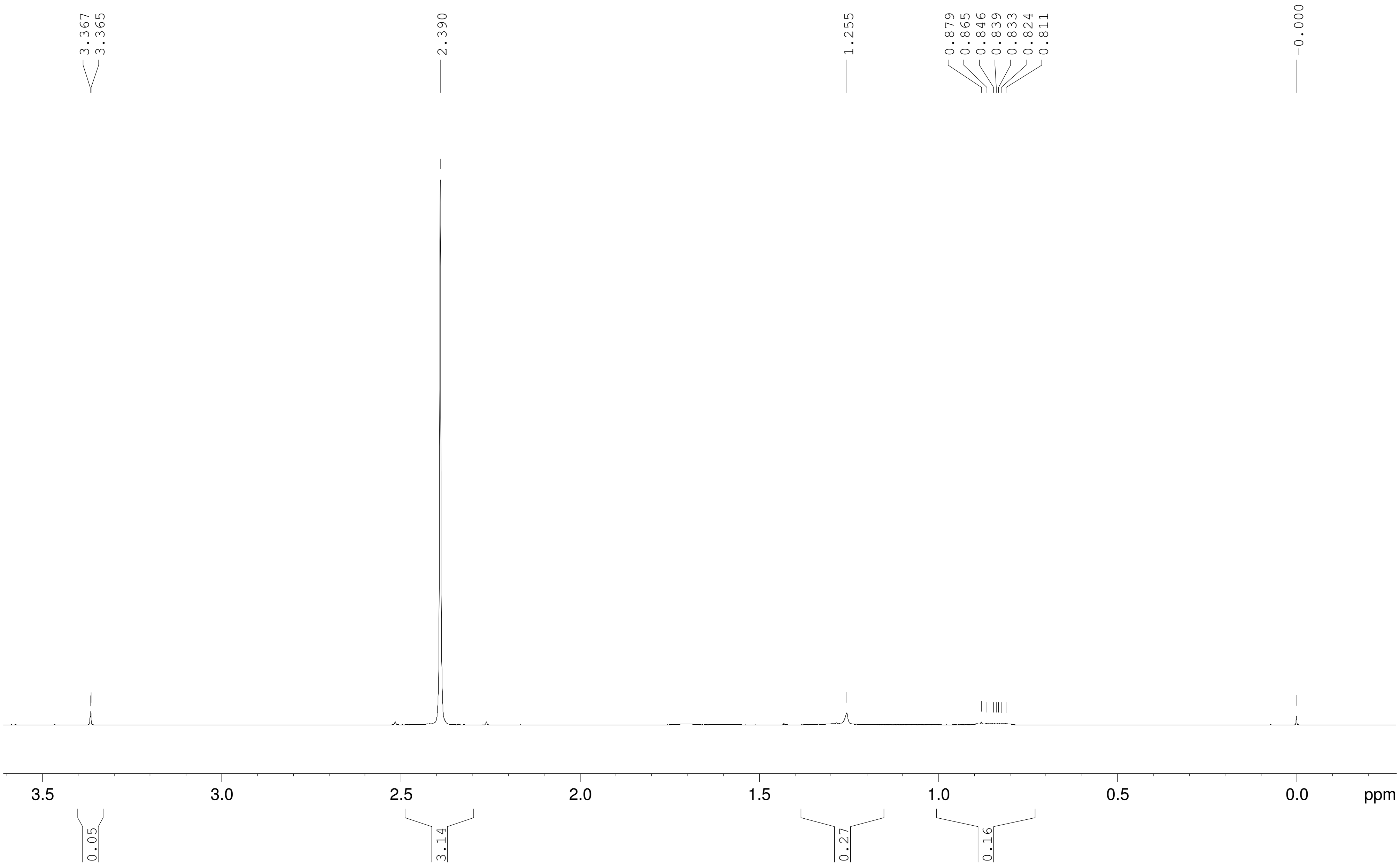

3537-13C
S-Tos-amid

Current Data Parameters

NAME	3537
EXPNO	2
PROCNO	1

F2 - Acquisition Parameters

Date_	20170217
PROBHD	5 mm PABBI 1H/
PULPROG	zg_pi_CPD
SOLVENT	CDCl3
TE	298.2 K
SWH	31249.381 Hz
AQ	1.0485969 sec
NS	124
FIDRES	0.476828 Hz
D1	1.50000000 sec
D11	0.03000000 sec
D14	0.00050000 sec

======== CHANNEL f1 ========

SFO1	125.7904831 MHz
NUC1	13C
P1	15.00 usec
P2	30.00 usec
PLW1	78.52400208 W

======== CHANNEL f2 ========

SFO2	500.2020008 MHz
NUC2	1H
PLW2	12.50000000 W
PLW12	0.12500000 W

F2 - Processing parameters

SI	65536
LB	0.50 Hz

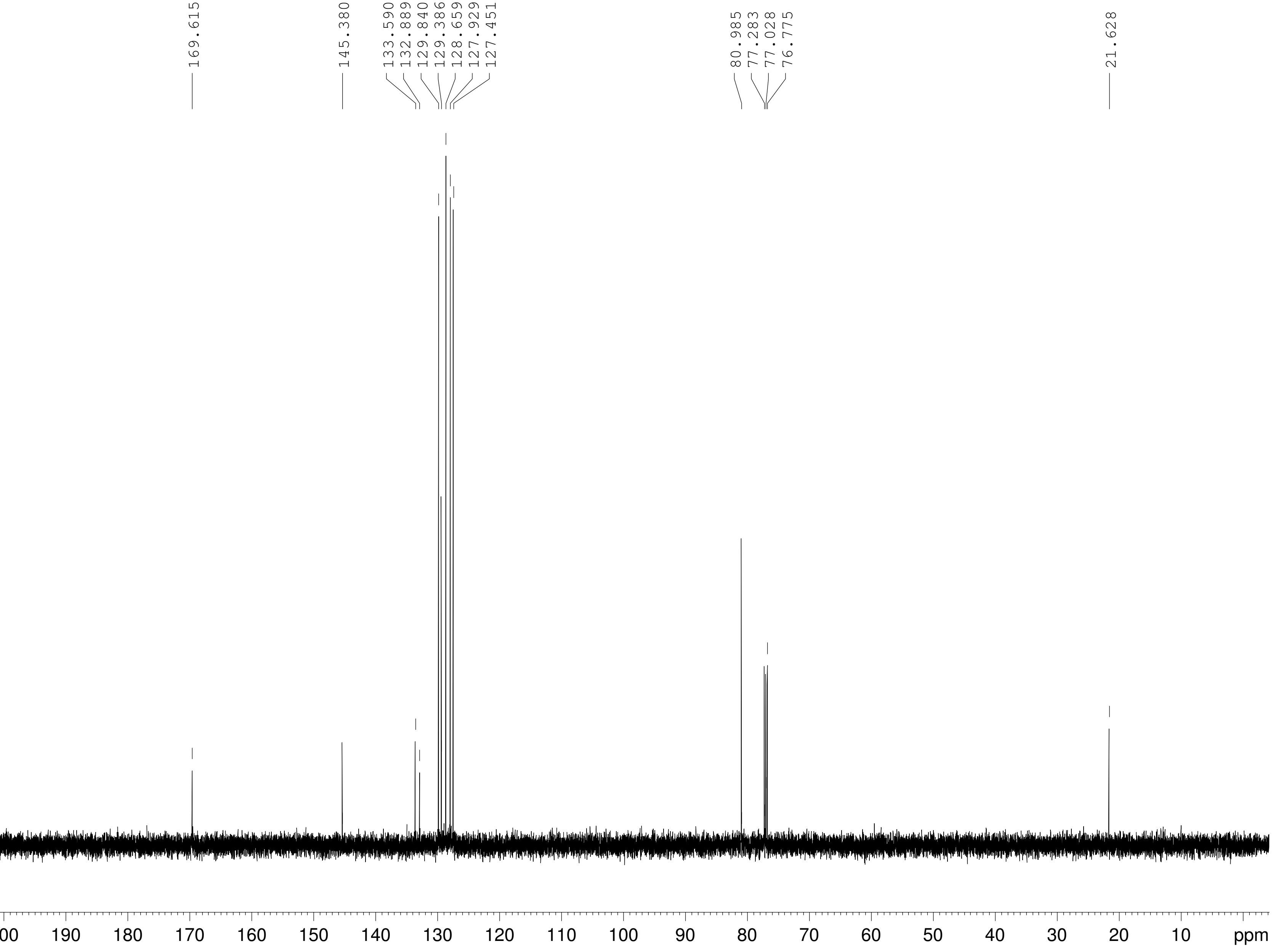

3537-13C
S-Tos-amid

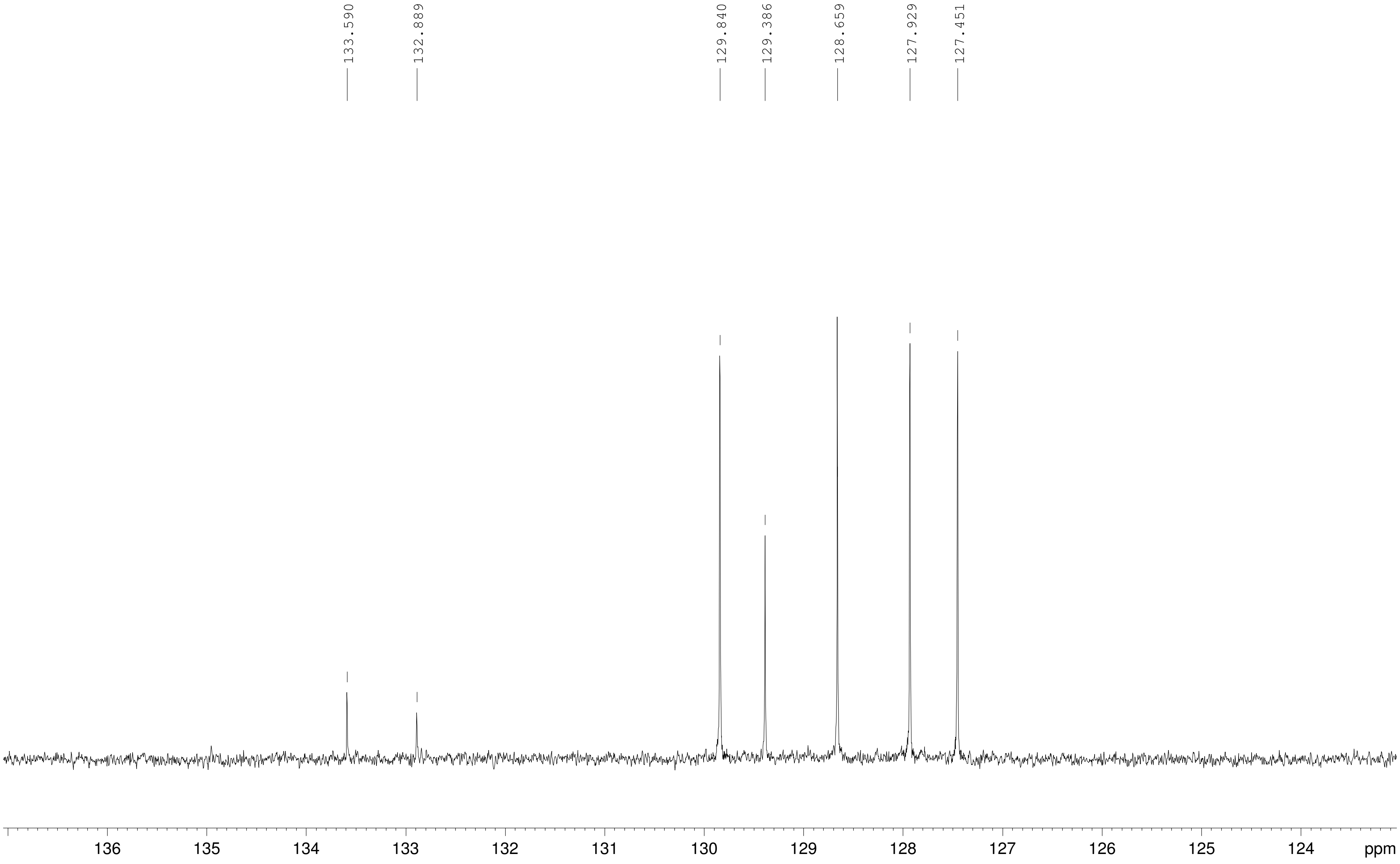

3537-COSY
S-Tos-amid

Current Data Parameters
NAME 3537
EXPNO 4
PROCNO 1

F2 - Acquisition Parameters
Date_ 20170217
PROBHD 5 mm PABBI 1H/
PULPROG cosygpppqf
SOLVENT CDCl3
TE 298.1 K
SWH 5530.974 Hz
AQ 0.1851392 sec
NS 1
FIDRES 2.700671 Hz
D0 0.00000300 sec
D1 2.00000000 sec
D11 0.03000000 sec
D12 0.00002000 sec
D13 0.00000400 sec
D16 0.00020000 sec

======== CHANNEL f1 ========
SFO1 500.2022861 MHz
NUC1 1H
P0 8.00 usec
P1 8.00 usec
P17 2500.00 usec
PLW1 12.69999981 W
PLW10 1.20239997 W

====== GRADIENT CHANNEL =====
P16 1000.00 usec

F1 - Acquisition parameters
TD 128
SFO1 500.2023 MHz
FIDRES 43.210732 Hz
SW 11.057 ppm
FnMODE QF

F2 - Processing parameters
SI 2048
LB 0 Hz

F1 - Processing parameters
SI 1024
MC2 QF
SF 500.2000127 MHz
WDW QSINE
SSB 0
LB 0 Hz
GB 0

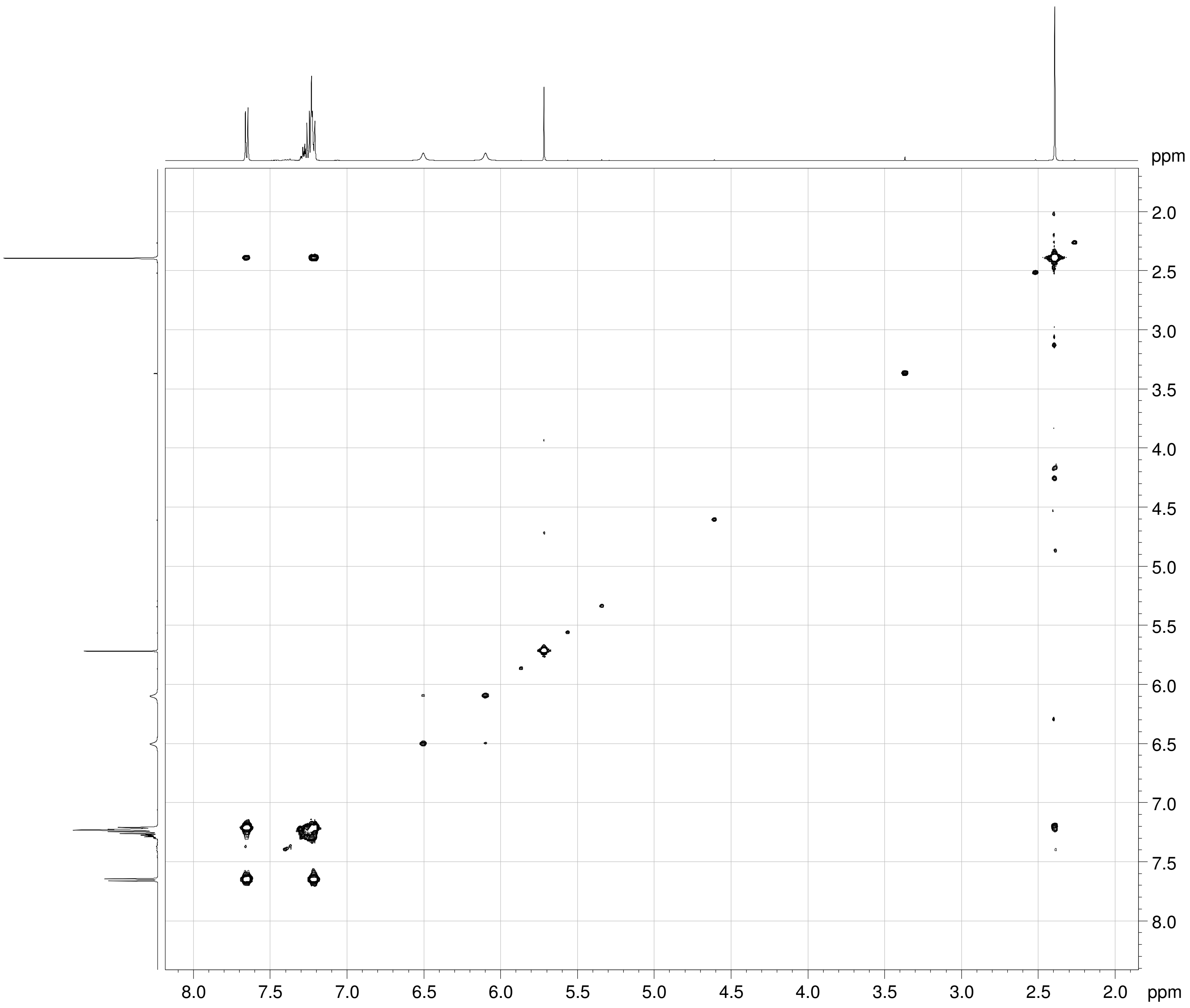

3537-HSQC
S-Tos-amid

```
Current Data Parameters
NAME                3537
EXPNO                  3
PROCNO                 1

F2 - Acquisition Parameters
Date_           20170217
PROBHD   5 mm PABBI 1H/
PULPROG  hsqcedetgpsisp2.3
SOLVENT            CDCl3
TE                 298.5 K
SWH             5530.974 Hz
AQ             0.1851392 sec
NS                     2
FIDRES          2.700671 Hz
D0            0.00000300 sec
D1            2.00000000 sec
D4            0.00172414 sec
D11           0.03000000 sec
D16           0.00020000 sec
D21           0.00360000 sec
D24           0.00089000 sec

======== CHANNEL f1 ========
SFO1        500.2022861 MHz
NUC1                  1H
P1                  8.00 usec
P2                 16.00 usec
P28                20.00 usec
PLW1         12.69999981 W

======== CHANNEL f2 ========
SFO2        125.7874959 MHz
NUC2                 13C
P3                 15.00 usec
P14               500.00 usec
P24              2000.00 usec
P31              1900.00 usec
PLW0      0 W
PLW2         78.52400208 W
PLW12         3.60570002 W

====== GRADIENT CHANNEL =====
P16              1000.00 usec
P19               600.00 usec

F1 - Acquisition parameters
TD                   128
SFO1            125.7875 MHz
FIDRES        190.548782 Hz
SW               193.900 ppm
FnMODE      Echo-Antiecho

F2 - Processing parameters
```

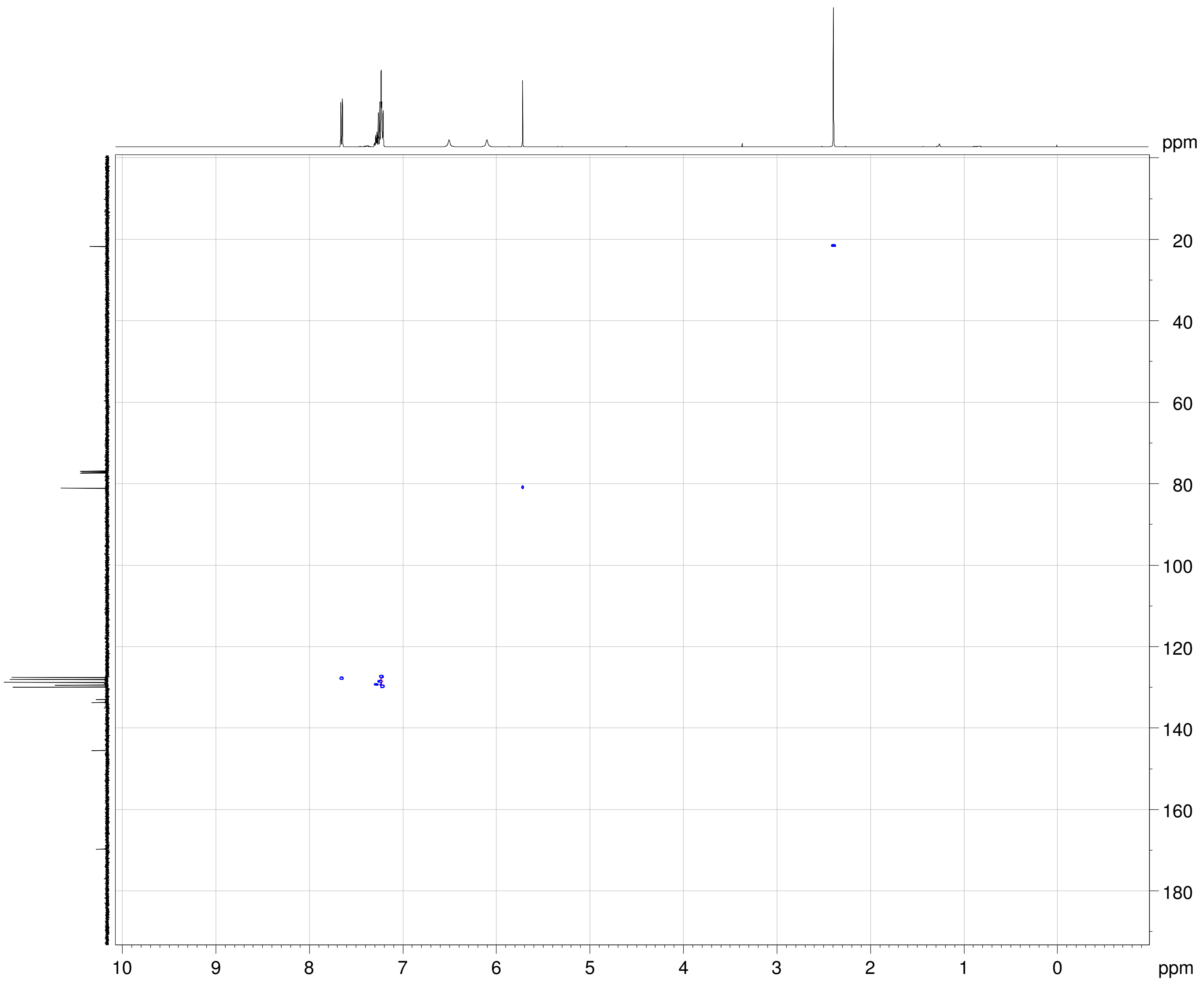

3537-HSQC
S-Tos-amid

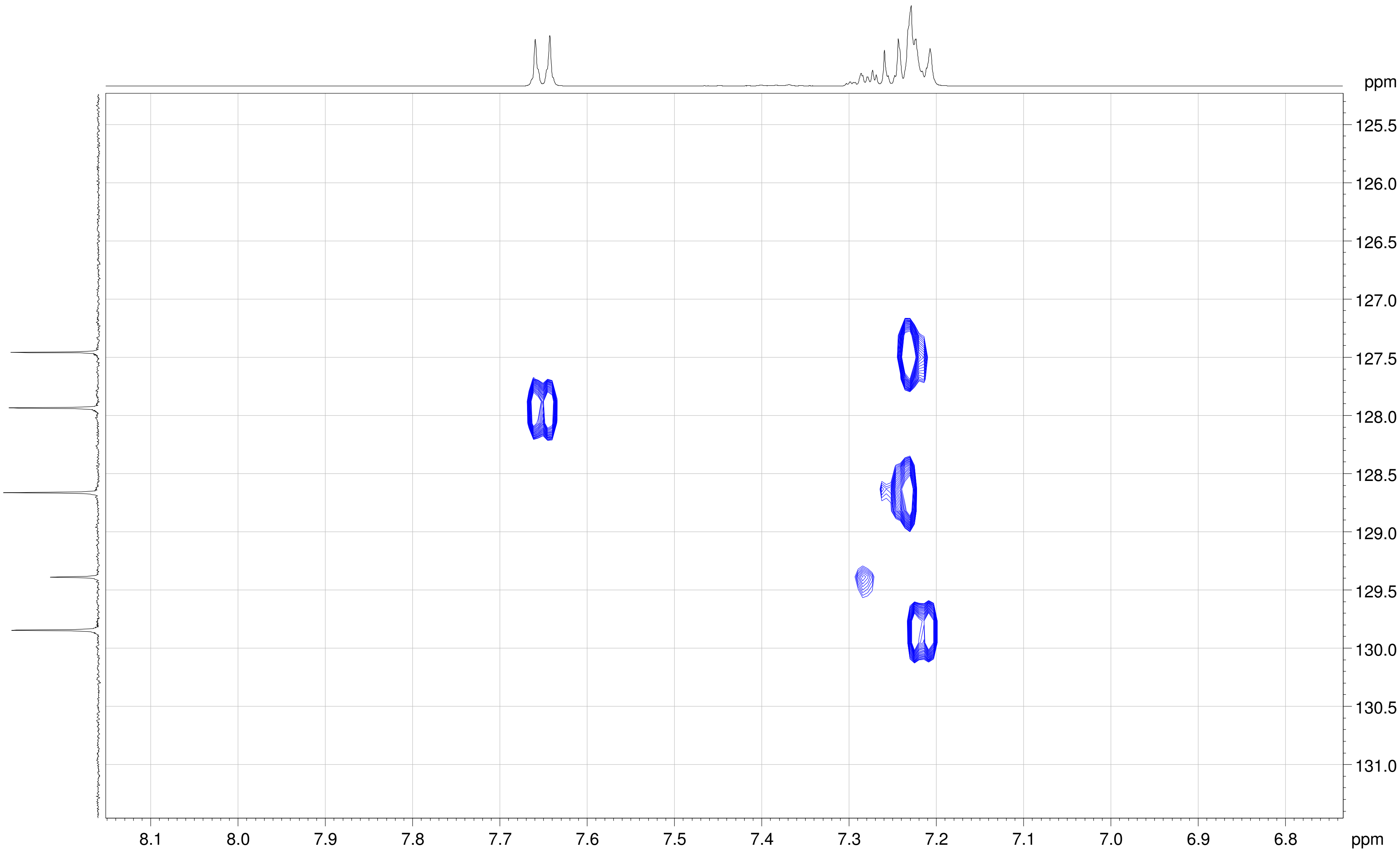

(3R,2S)-2a

3343-1H
H-mig-am-1

Current Data Parameters
NAME 3343
EXPNO 1
PROCNO 1

F2 - Acquisition Parameters
Date_ 20161004
PROBHD 5 mm CPPBBO BB
PULPROG zg30
SOLVENT CDCl3
TE 298.2 K
SWH 10000.000 Hz
AQ 3.2767999 sec
NS 8
FIDRES 0.152588 Hz
D1 0 sec

======== CHANNEL f1 ========
SFO1 500.2030889 MHz
NUC1 1H
P1 11.30 usec
PLW1 13.69999981 W

F2 - Processing parameters
SI 65536
LB 0 Hz

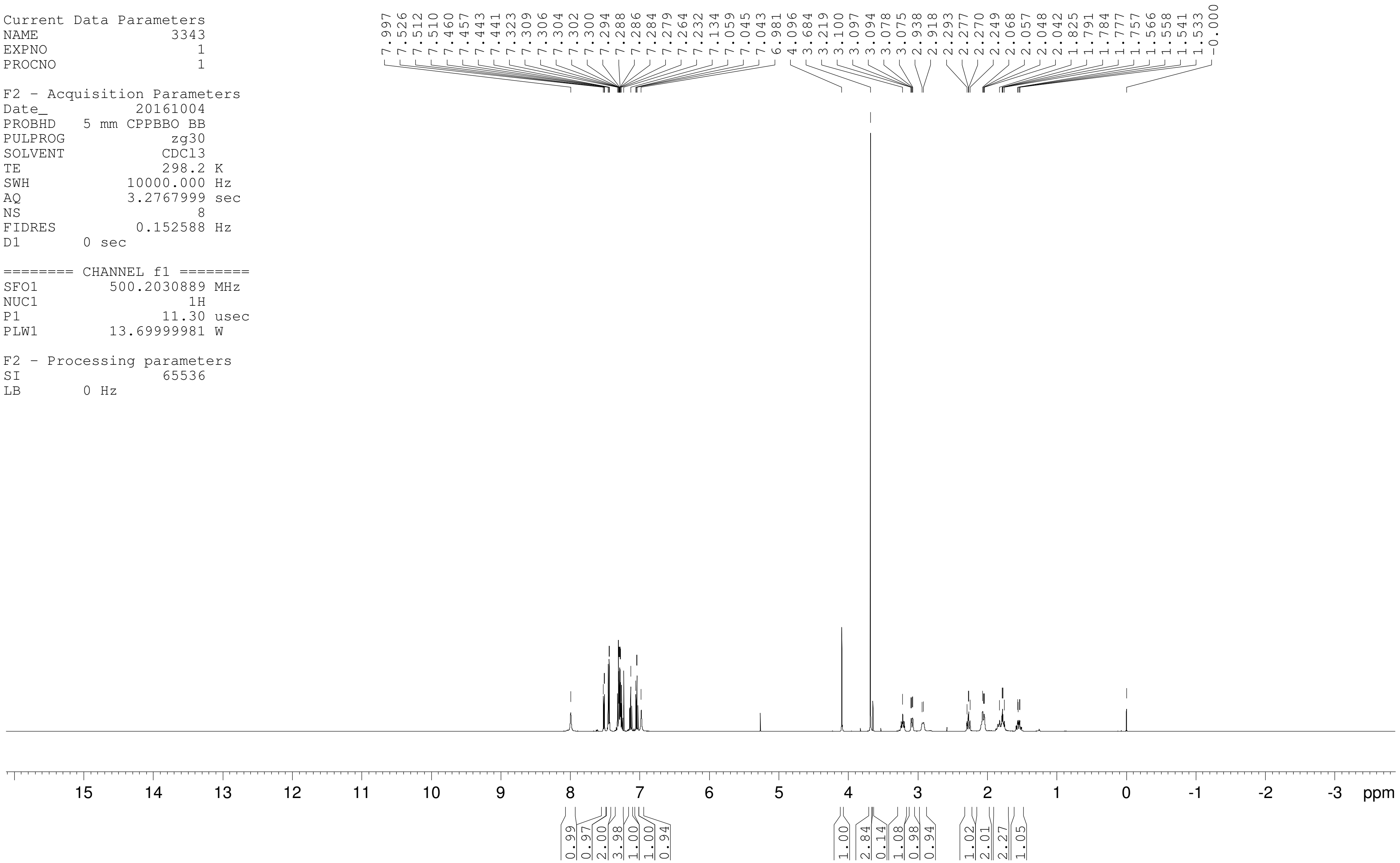

3343-1H
H-mig-am-1

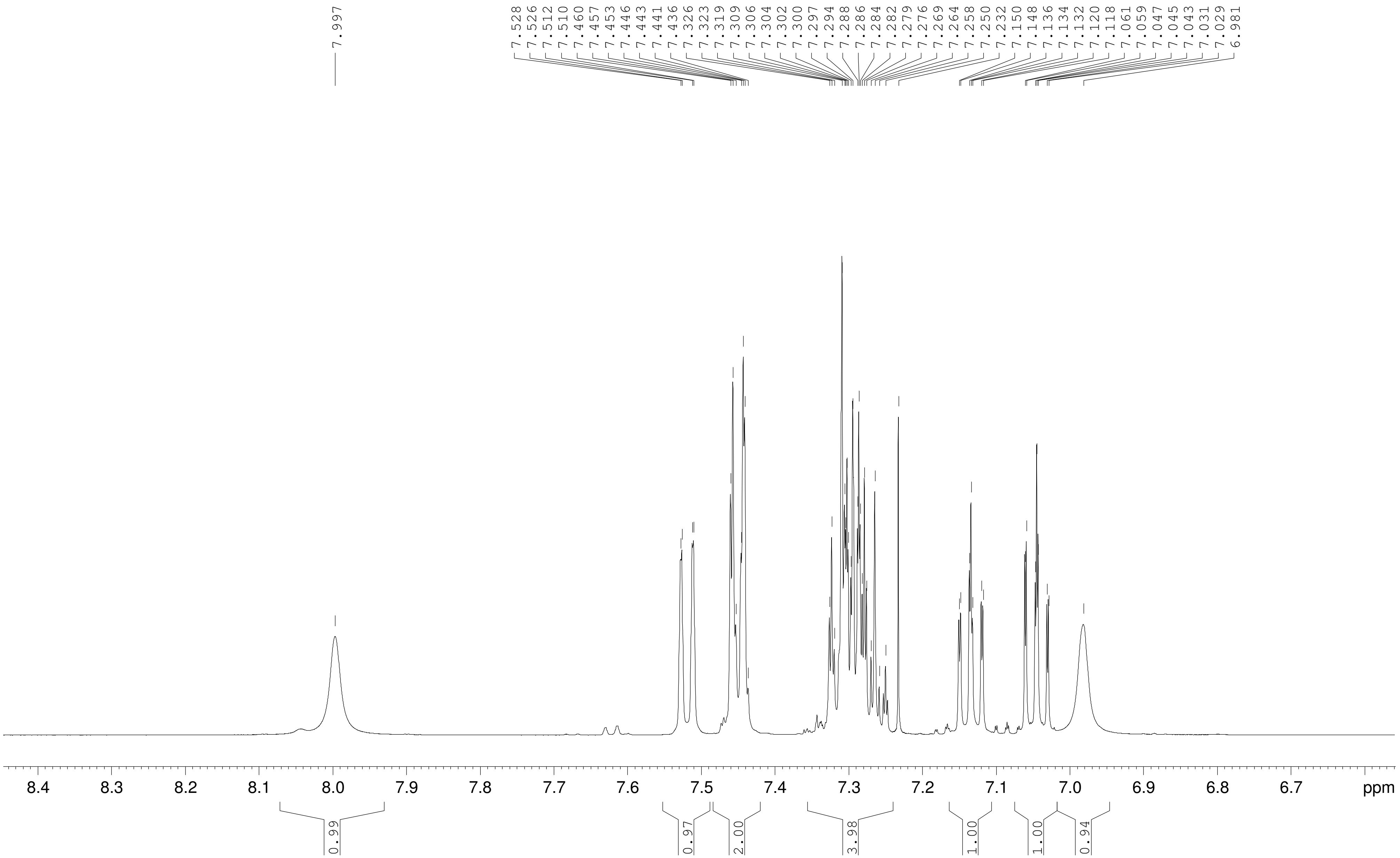

3343-1H
H-mig-am-1

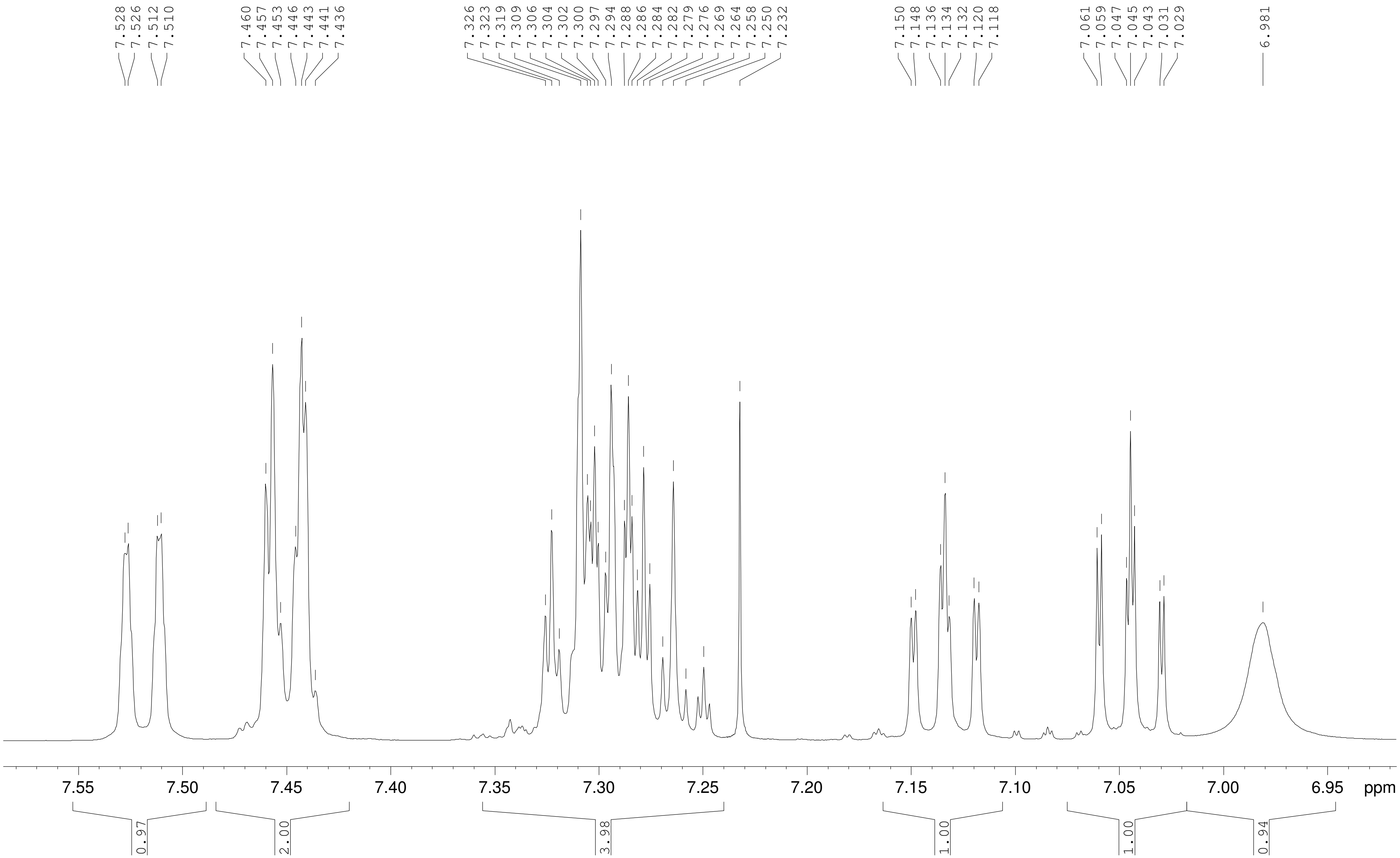

3343-1H
H-mig-am-1

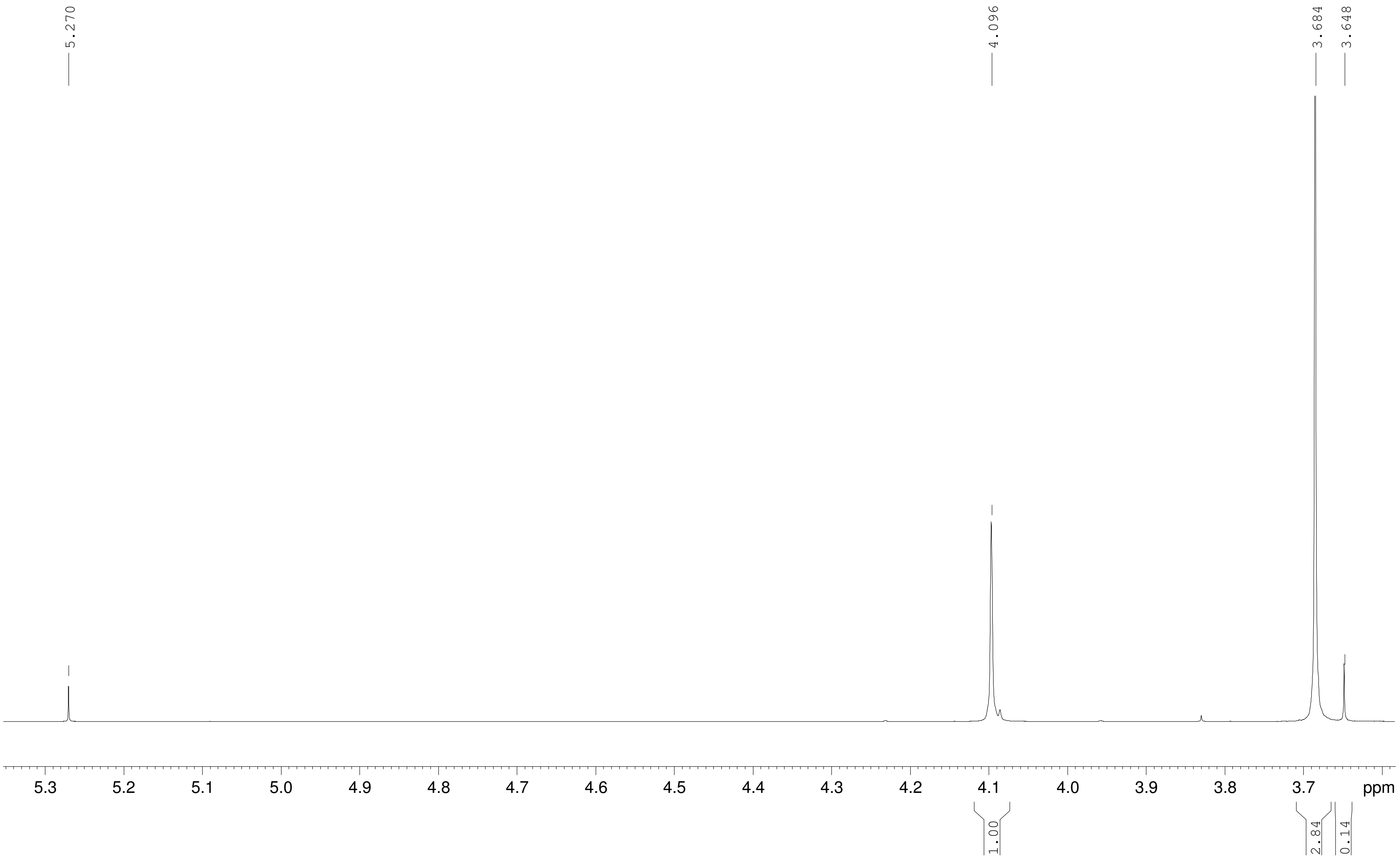

3343-1H
H-mig-am-1

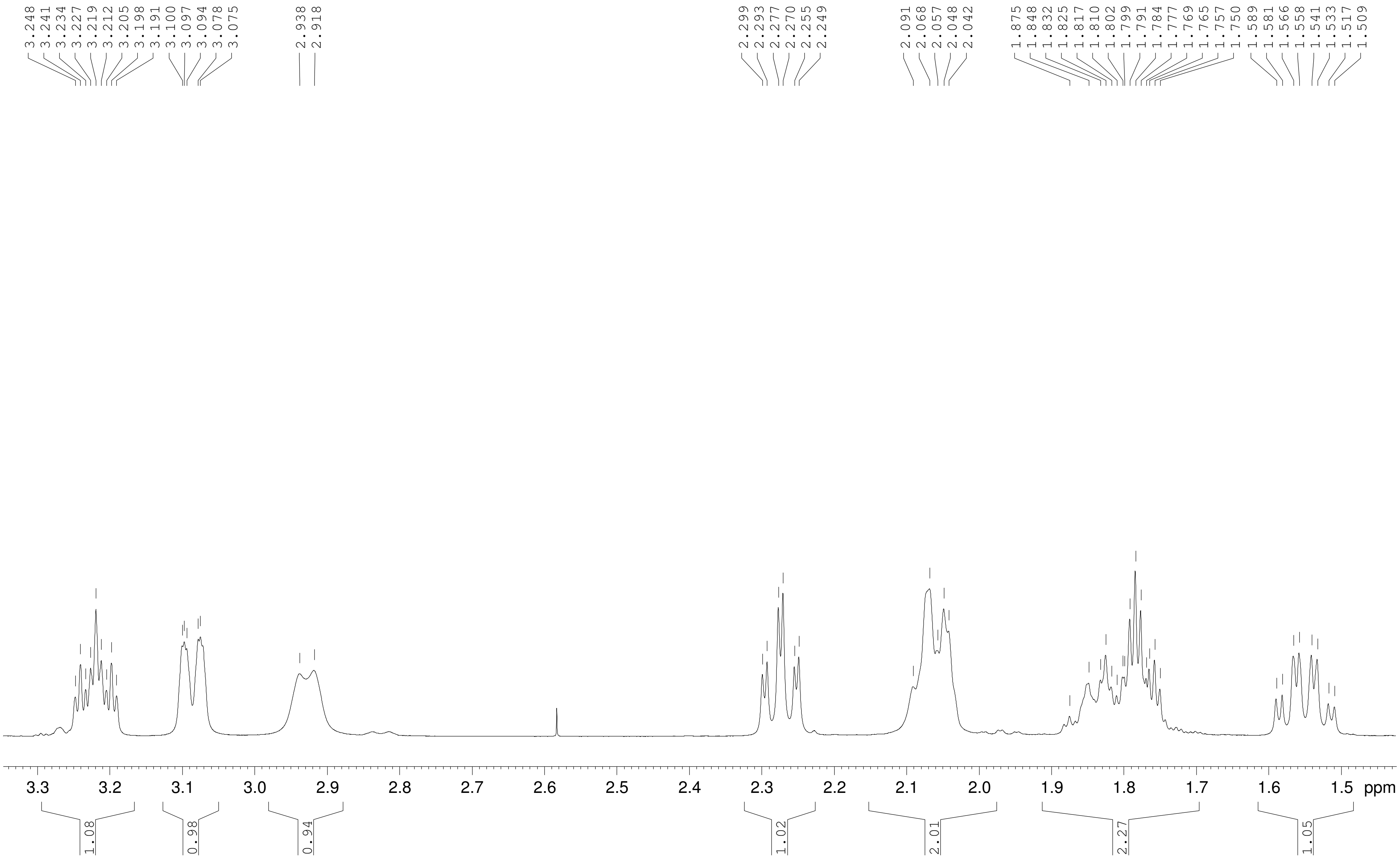

3343-13C
H-mig-am-1

Current Data Parameters
NAME 3343
EXPNO 2
PROCNO 1

F2 - Acquisition Parameters
Date_ 20161004
PROBHD 5 mm CPPBBO BB
PULPROG zg_pi_CPD
SOLVENT CDCl3
TE 298.1 K
SWH 31249.381 Hz
AQ 1.0485969 sec
NS 64
FIDRES 0.476828 Hz
D1 1.50000000 sec
D11 0.03000000 sec
D14 0.00050000 sec

======== CHANNEL f1 ========
SFO1 125.7904831 MHz
NUC1 13C
P1 10.00 usec
P2 20.00 usec
PLW1 55.00000000 W

======== CHANNEL f2 ========
SFO2 500.2020008 MHz
NUC2 1H
PLW2 13.00000000 W
PLW12 0.25937000 W

F2 - Processing parameters
SI 65536
LB 0.50 Hz

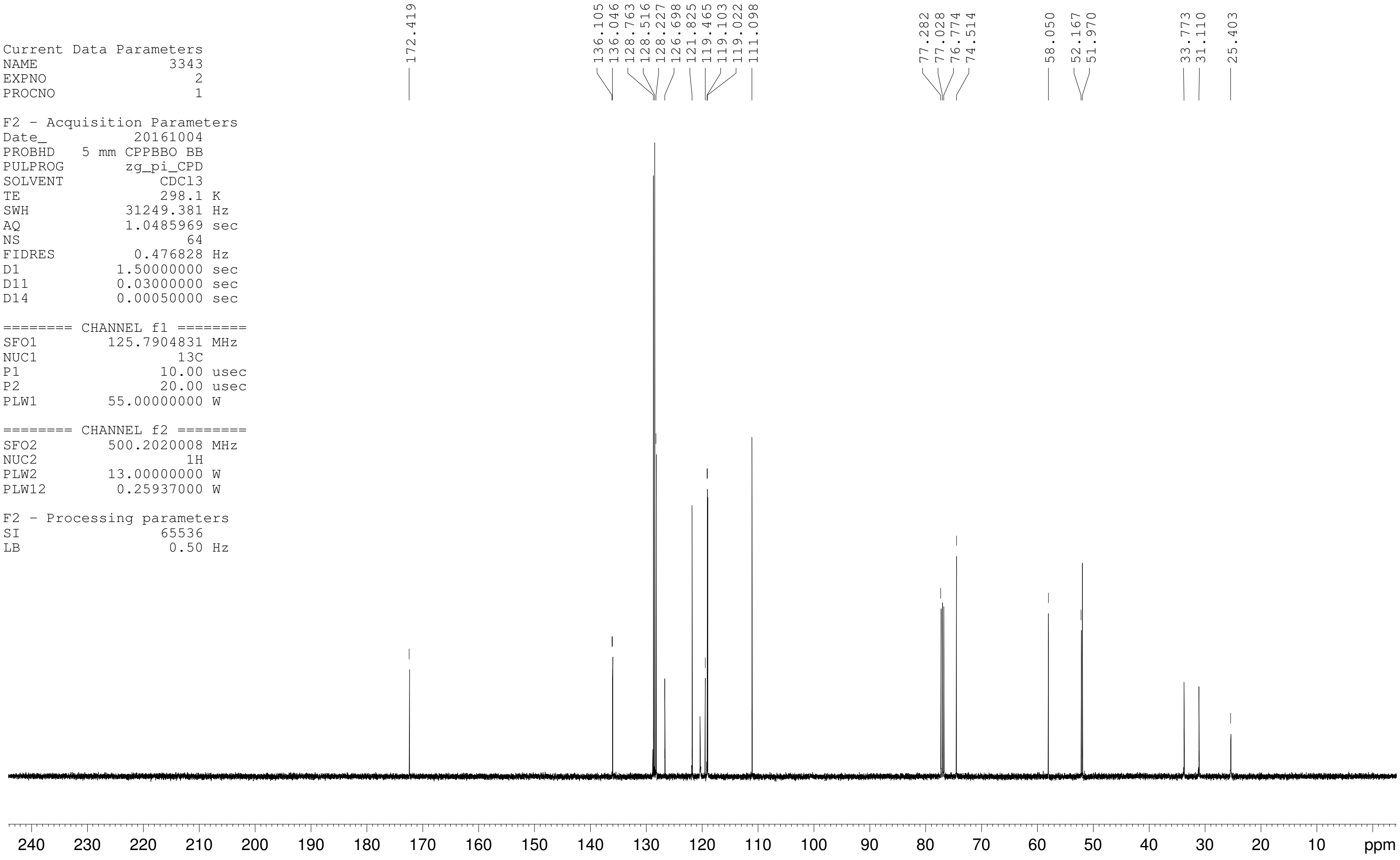

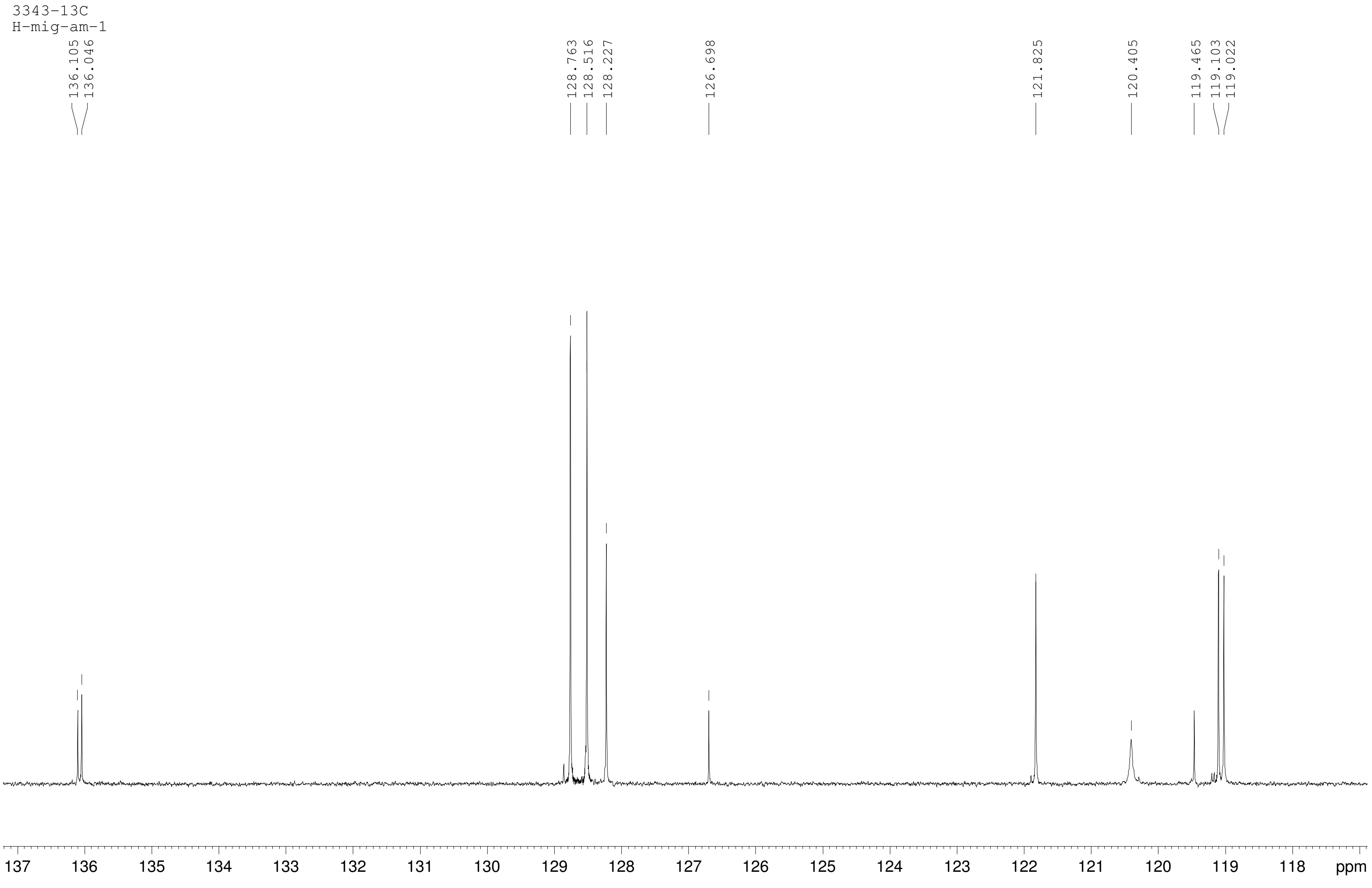
3343-13C
H-mig-am-1
136.105
136.046
128.763
128.516
128.227
126.698
121.825
120.405
119.465
119.103
119.022
137
136
135
134
133
132
131
130
129
128
127
126
125
124
123
122
121
120
119
118
ppm

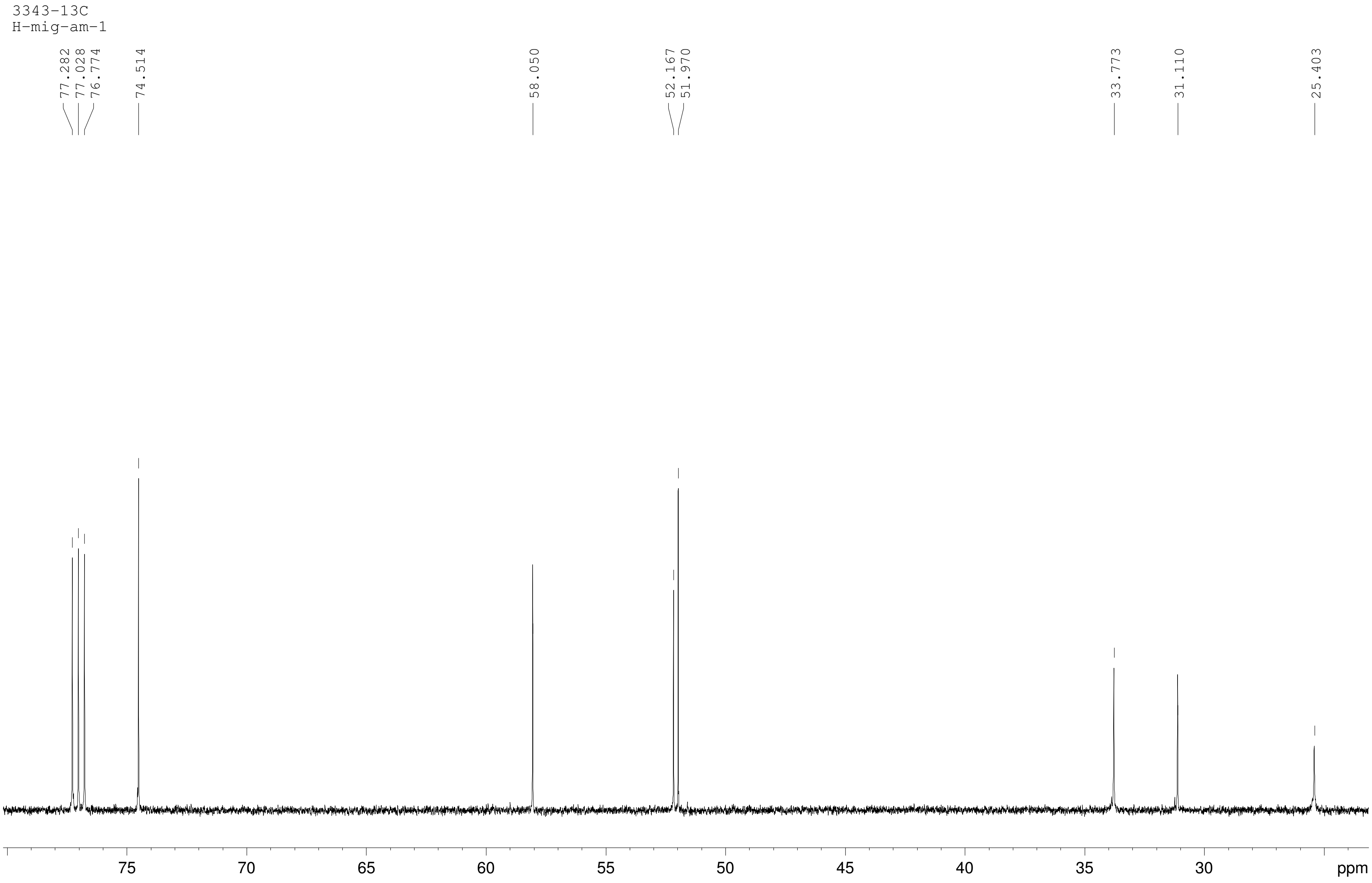
3343-13C
H-mig-am-1
77.282
77.028
76.774
74.514
58.050
52.167
51.970
33.773
31.110
25.403
75
70
65
60
55
50
45
40
35
30
ppm

3343-COSY
H-mig-am-1

Current Data Parameters
NAME 3343
EXPNO 3
PROCNO 1

F2 - Acquisition Parameters
Date_ 20161004
PROBHD 5 mm CPPBBO BB
PULPROG cosygpppqf
SOLVENT CDCl3
TE 298.1 K
SWH 5543.237 Hz
AQ 0.1847296 sec
NS 2
FIDRES 2.706659 Hz
D0 0.00000300 sec
D1 2.00000000 sec
D11 0.03000000 sec
D12 0.00002000 sec
D13 0.00000400 sec
D16 0.00020000 sec

======== CHANNEL f1 ========
SFO1 500.2022693 MHz
NUC1 1H
P0 11.30 usec
P1 11.30 usec
P17 0 usec
PLW1 13.69999981 W
PLW10 2.58780003 W

====== GRADIENT CHANNEL =====
P16 1000.00 usec

F1 - Acquisition parameters
TD 128
SFO1 500.2023 MHz
FIDRES 43.306541 Hz
SW 11.082 ppm
FnMODE QF

F2 - Processing parameters
SI 2048
LB 0 Hz

F1 - Processing parameters
SI 1024
MC2 QF
SF 500.2000256 MHz
WDW QSINE
SSB 0
LB 0 Hz
GB 0

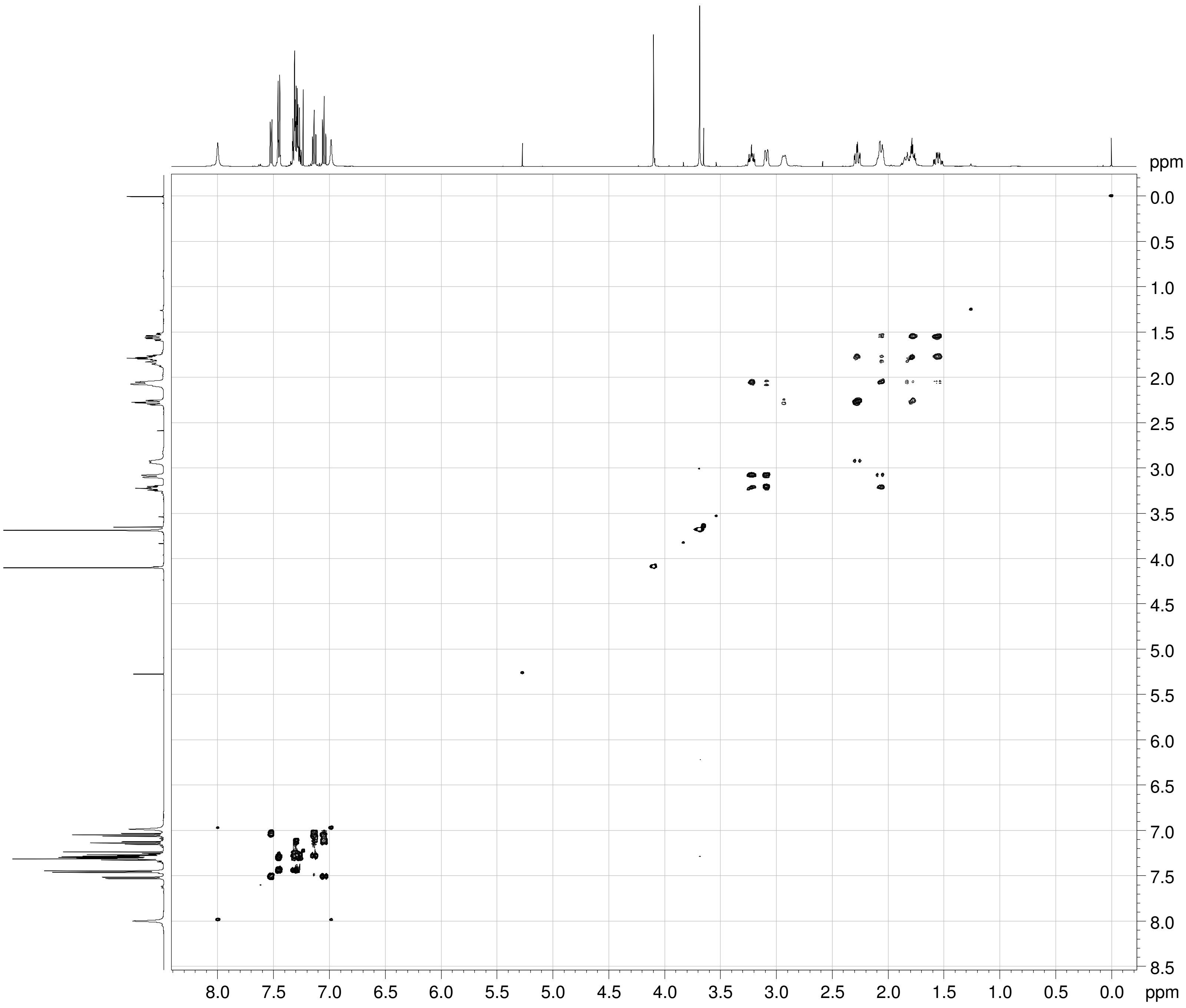

3343-COSY
H-mig-am-1

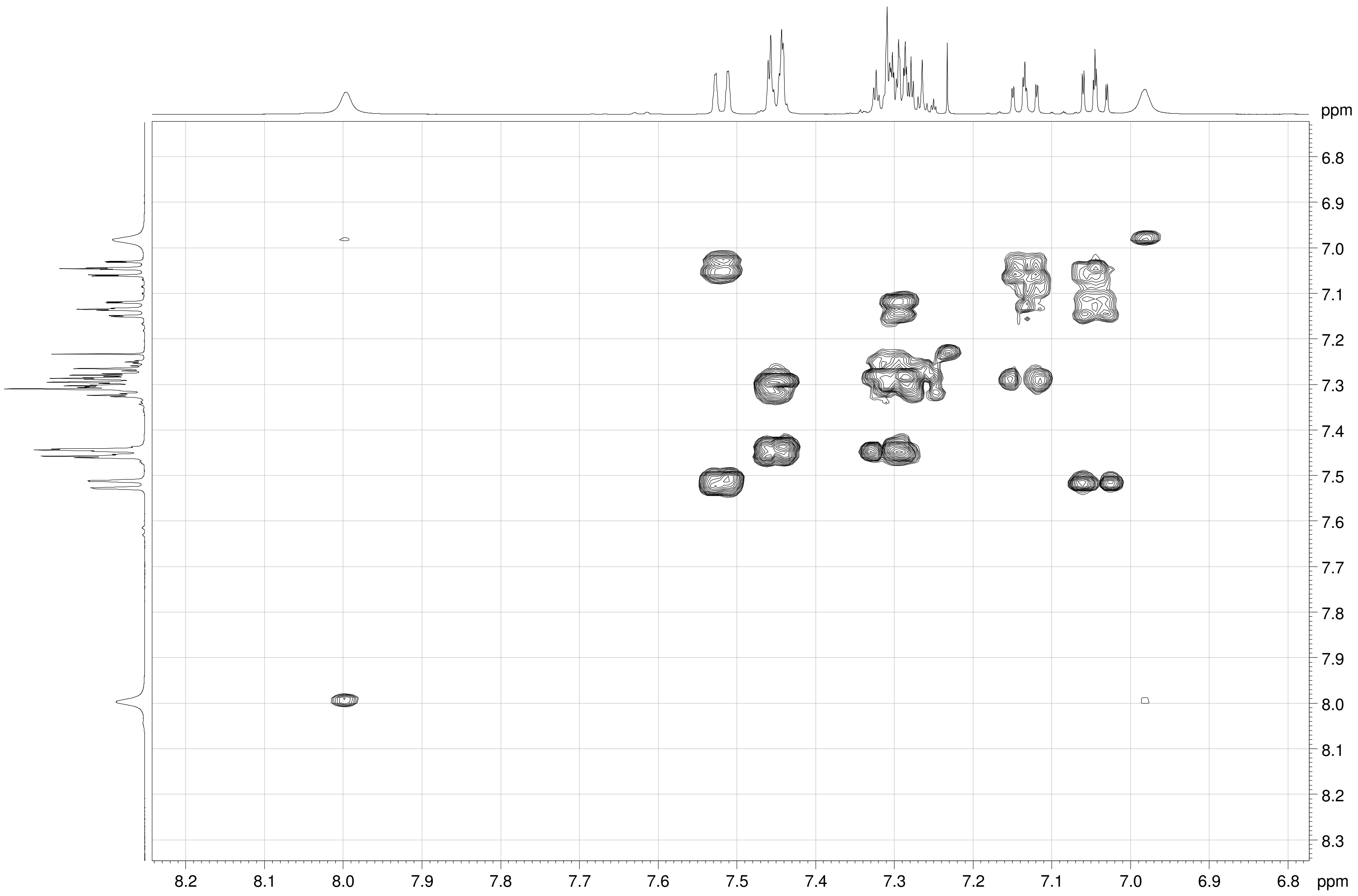

3343-COSY
H-mig-am-1

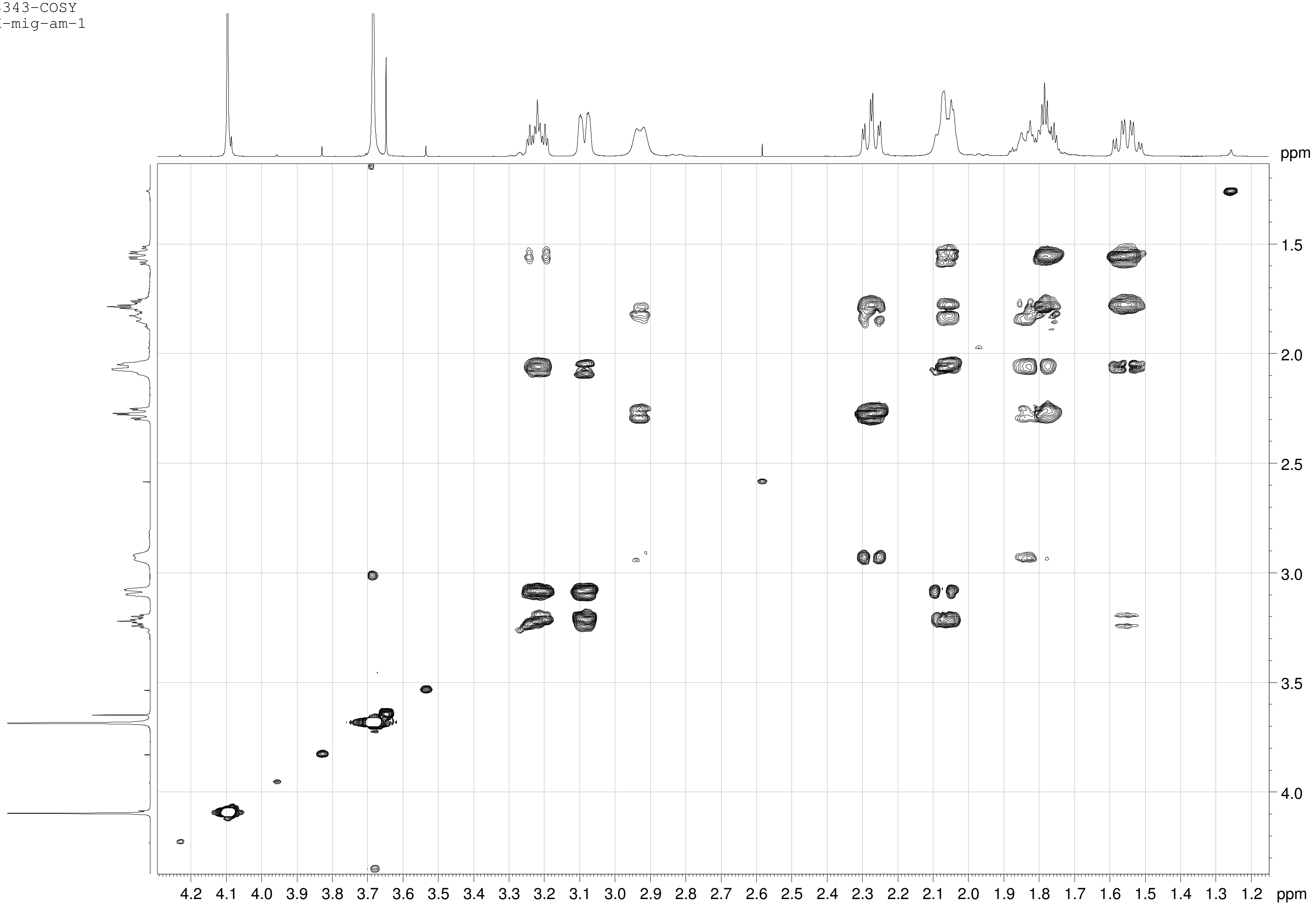

3343-HSQC
H-mig-am-1

Current Data Parameters
NAME 3343
EXPNO 4
PROCNO 1

F2 - Acquisition Parameters
Date_ 20161004
PROBHD 5 mm CPPBBO BB
PULPROG hsqcedetgpsisp2.3
SOLVENT CDCl3
TE 298.2 K
SWH 5543.237 Hz
AQ 0.1847296 sec
NS 4
FIDRES 2.706659 Hz
D0 0.00000300 sec
D1 2.00000000 sec
D4 0.00172414 sec
D11 0.03000000 sec
D16 0.00020000 sec
D21 0.00360000 sec
D24 0.00089000 sec

======== CHANNEL f1 ========
SFO1 500.2022693 MHz
NUC1 1H
P1 11.30 usec
P2 22.60 usec
P28 0 usec
PLW1 13.69999981 W

======== CHANNEL f2 ========
SFO2 125.7879682 MHz
NUC2 13C
P3 10.00 usec
P14 500.00 usec
P24 2000.00 usec
P31 1900.00 usec
PLW0 0 W
PLW2 55.00000000 W
PLW12 1.30180001 W

====== GRADIENT CHANNEL =====
P16 1000.00 usec
P19 600.00 usec

F1 - Acquisition parameters
TD 128
SFO1 125.788 MHz
FIDRES 177.556824 Hz
SW 180.679 ppm
FnMODE Echo-Antiecho

F2 - Processing parameters

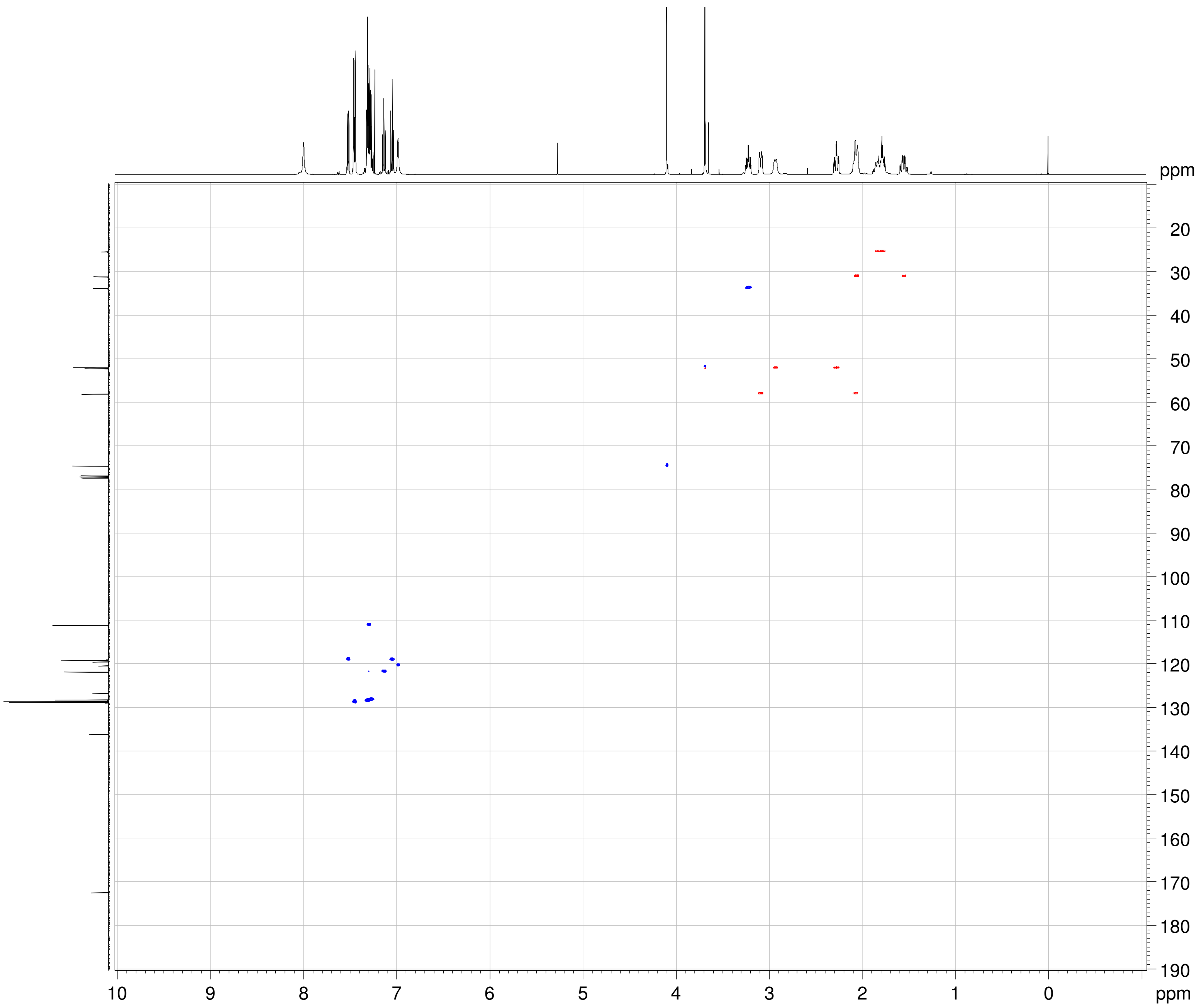

3343-HSQC
H-mig-am-1

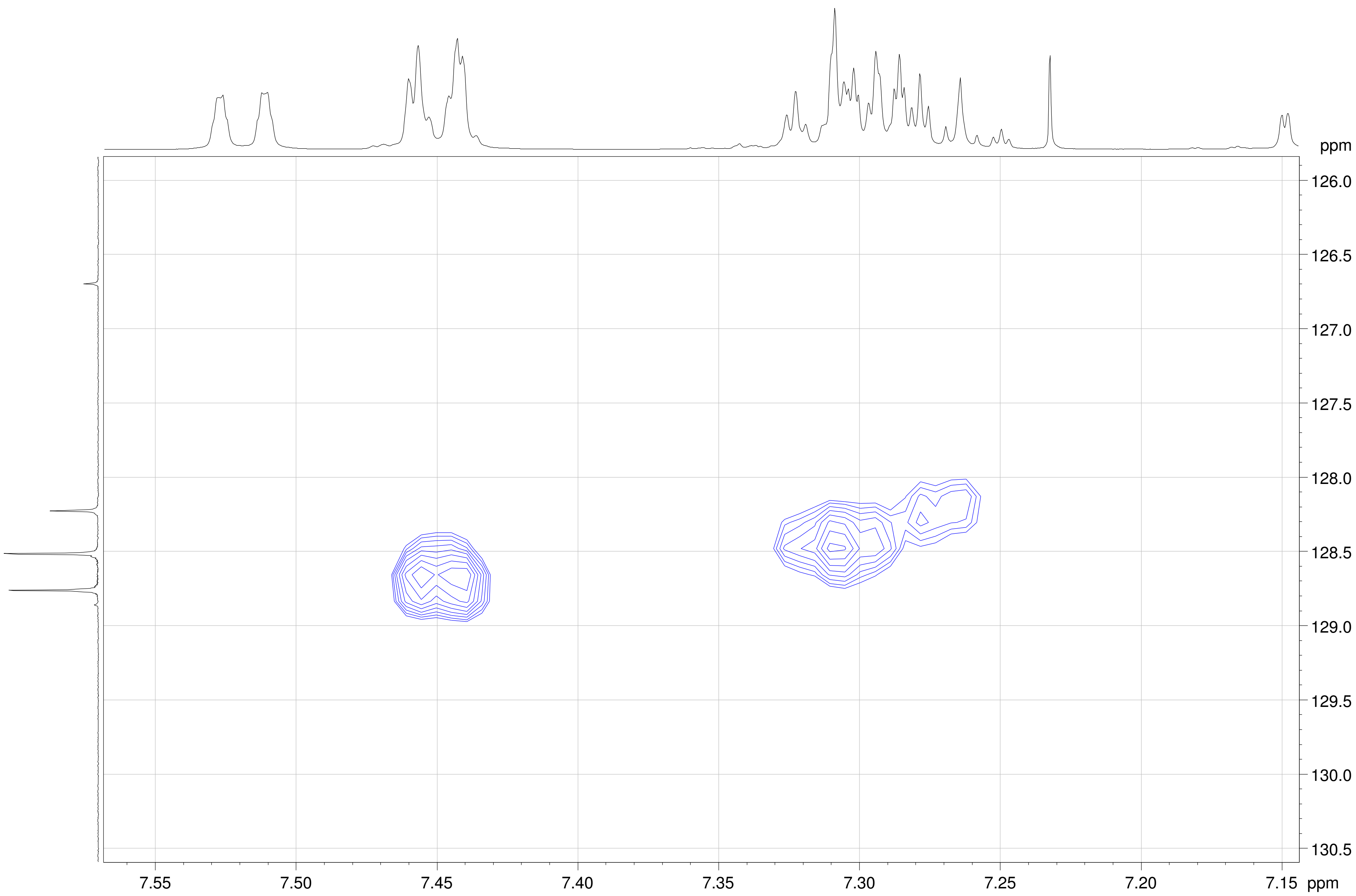

3343-HSQC
H-mig-am-1

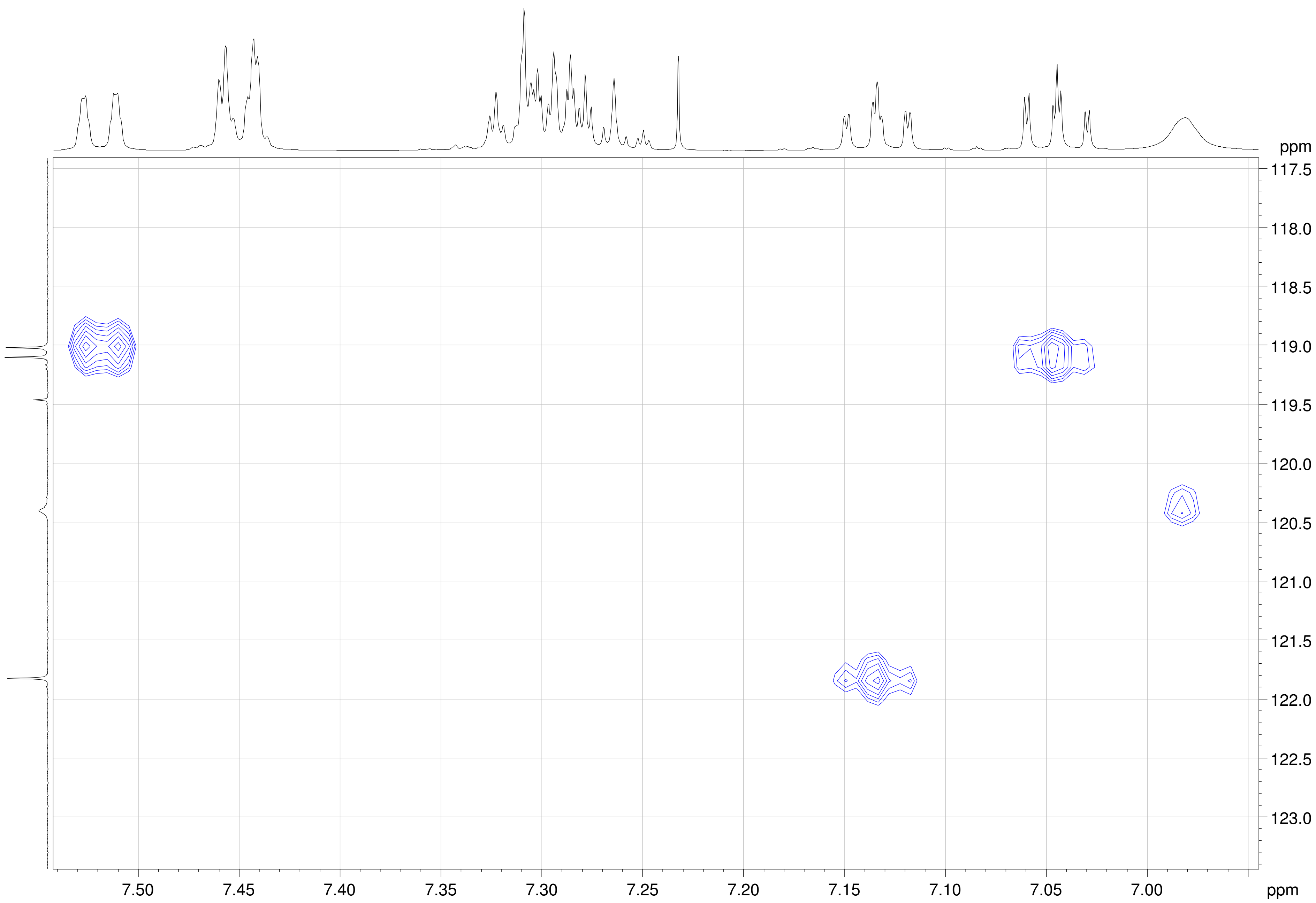

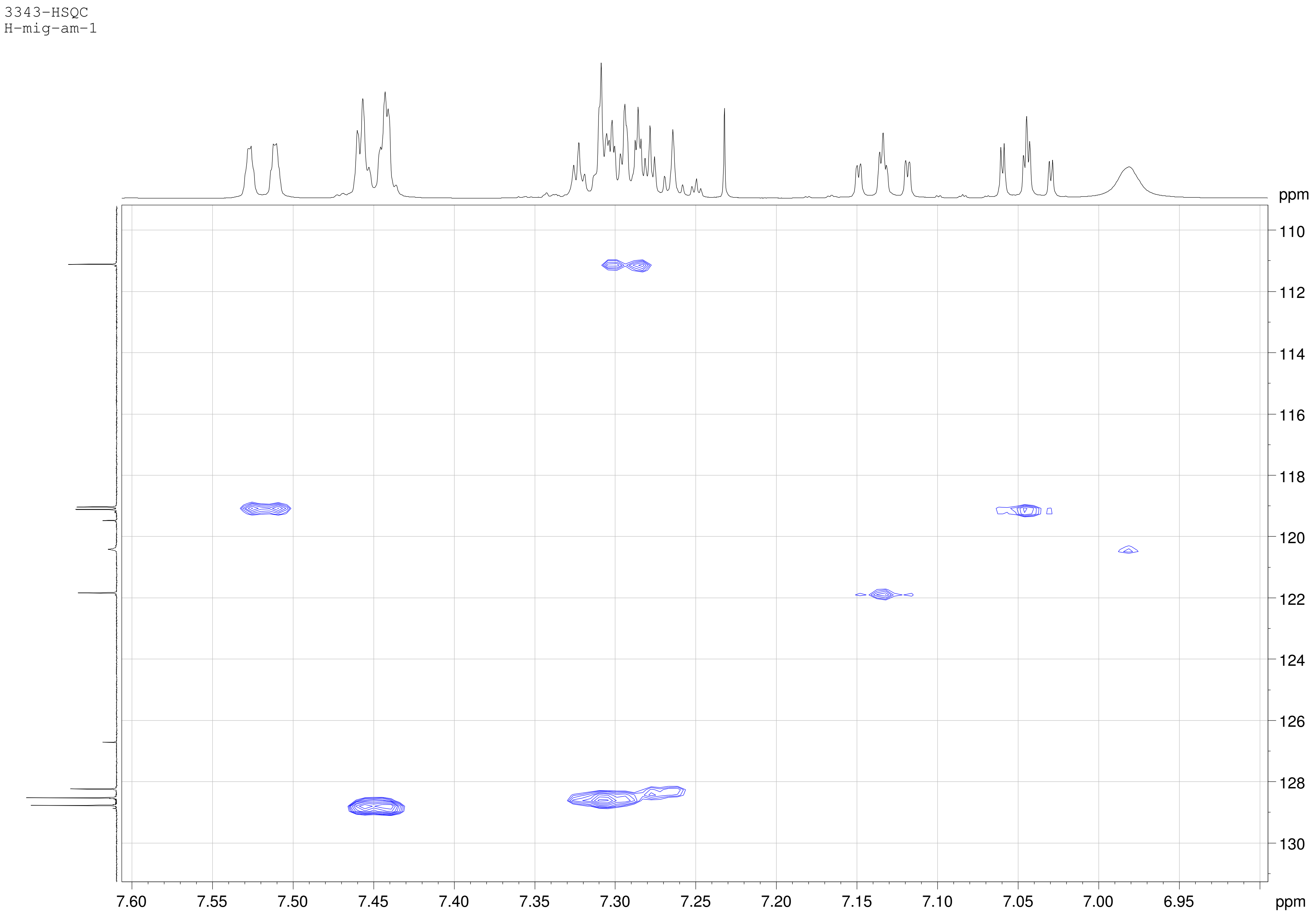
3343-HSQC
H-mig-am-1
ppm
110
112
114
116
118
120
122
124
126
128
130
7.60
7.55
7.50
7.45
7.40
7.35
7.30
7.25
7.20
7.15
7.10
7.05
7.00
6.95
ppm

3343-HSQC
H-mig-am-1

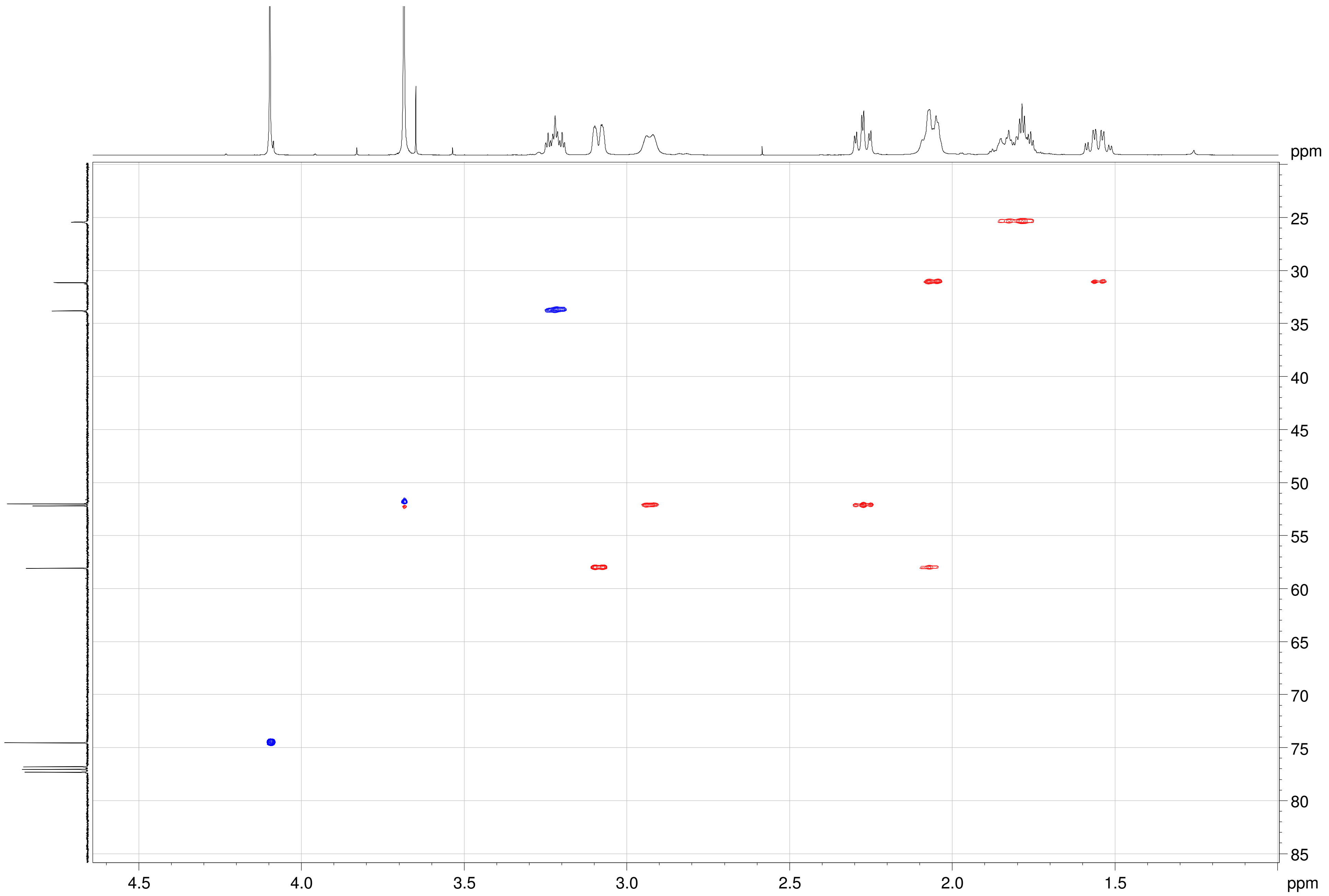

3343-HSQC
H-mig-am-1

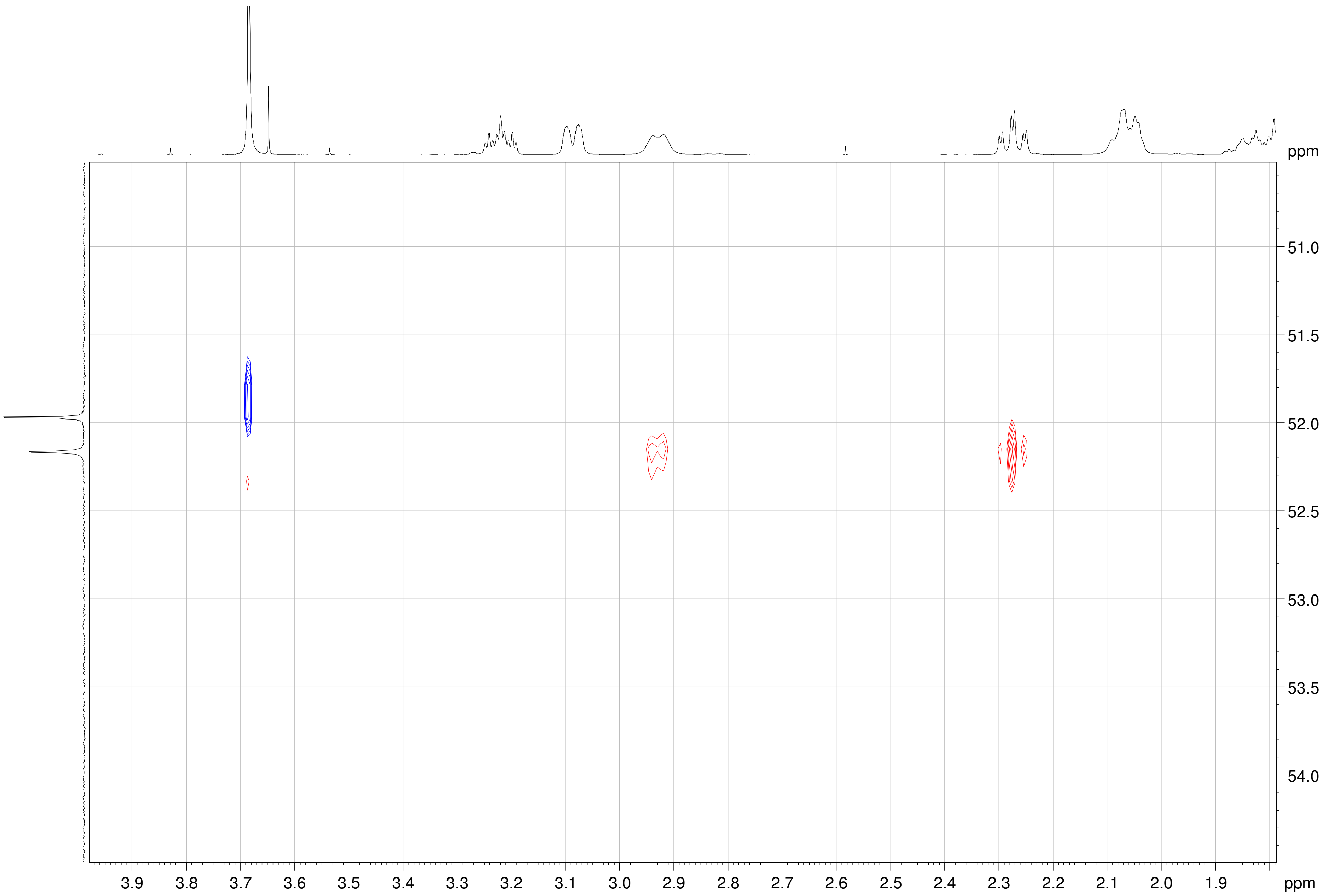

(3R,2R)-4a

3538-1H
A-amid

```
Current Data Parameters
NAME              3538
EXPNO                1
PROCNO               1

F2 - Acquisition Parameters
Date_         20170217
PROBHD   5 mm PABBI 1H/
PULPROG           zg30
SOLVENT          CDCl3
TE               298.2 K
SWH          10000.000 Hz
AQ           3.2767999 sec
NS                   8
FIDRES        0.152588 Hz
D1       0 sec

======== CHANNEL f1 =======
SFO1       500.2030889 MHz
NUC1                1H
P1                8.00 use
PLW1       12.69999981 W

F2 - Processing parameters
SI               65536
LB       0 Hz
```

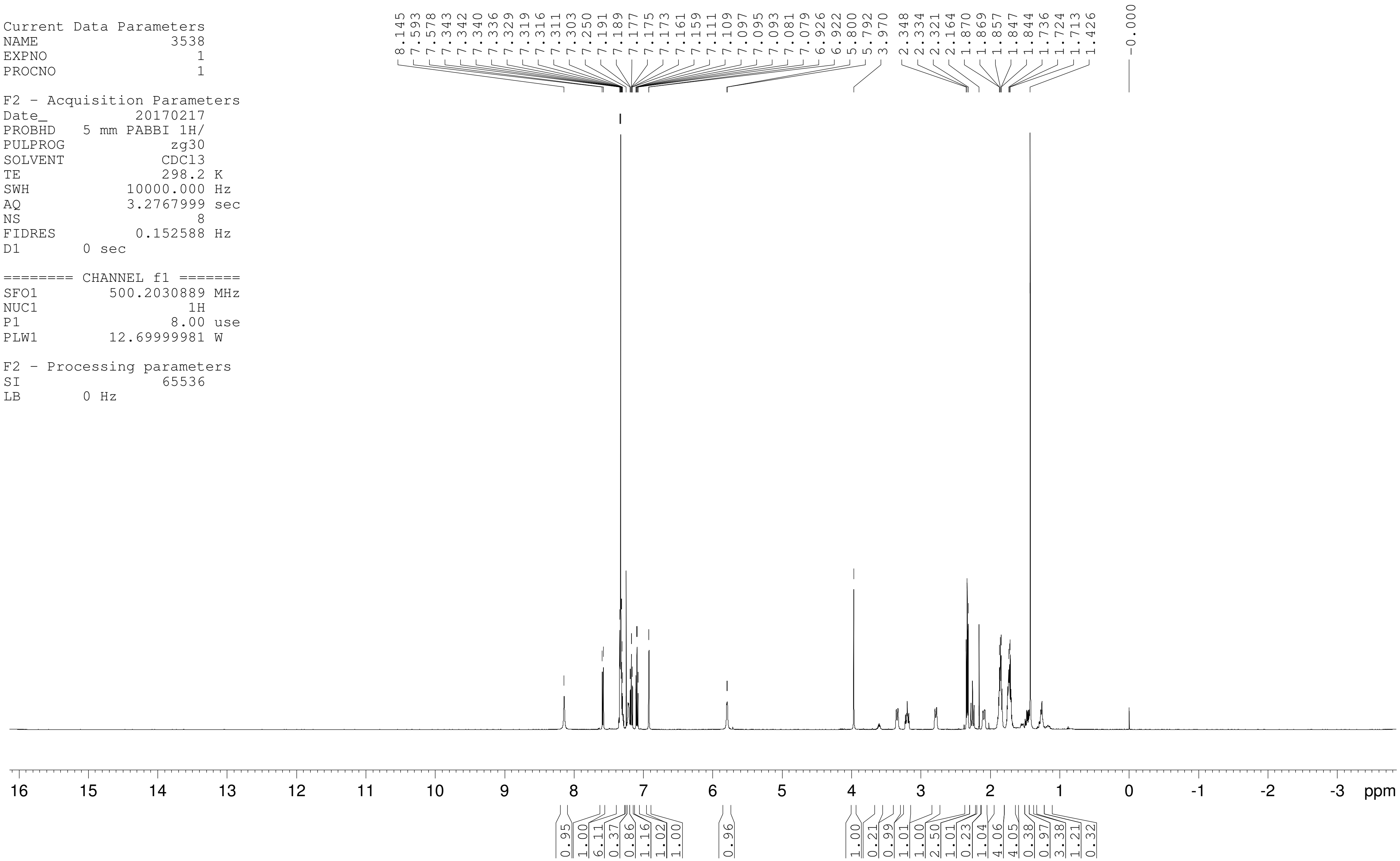

3538-1H
A-amid

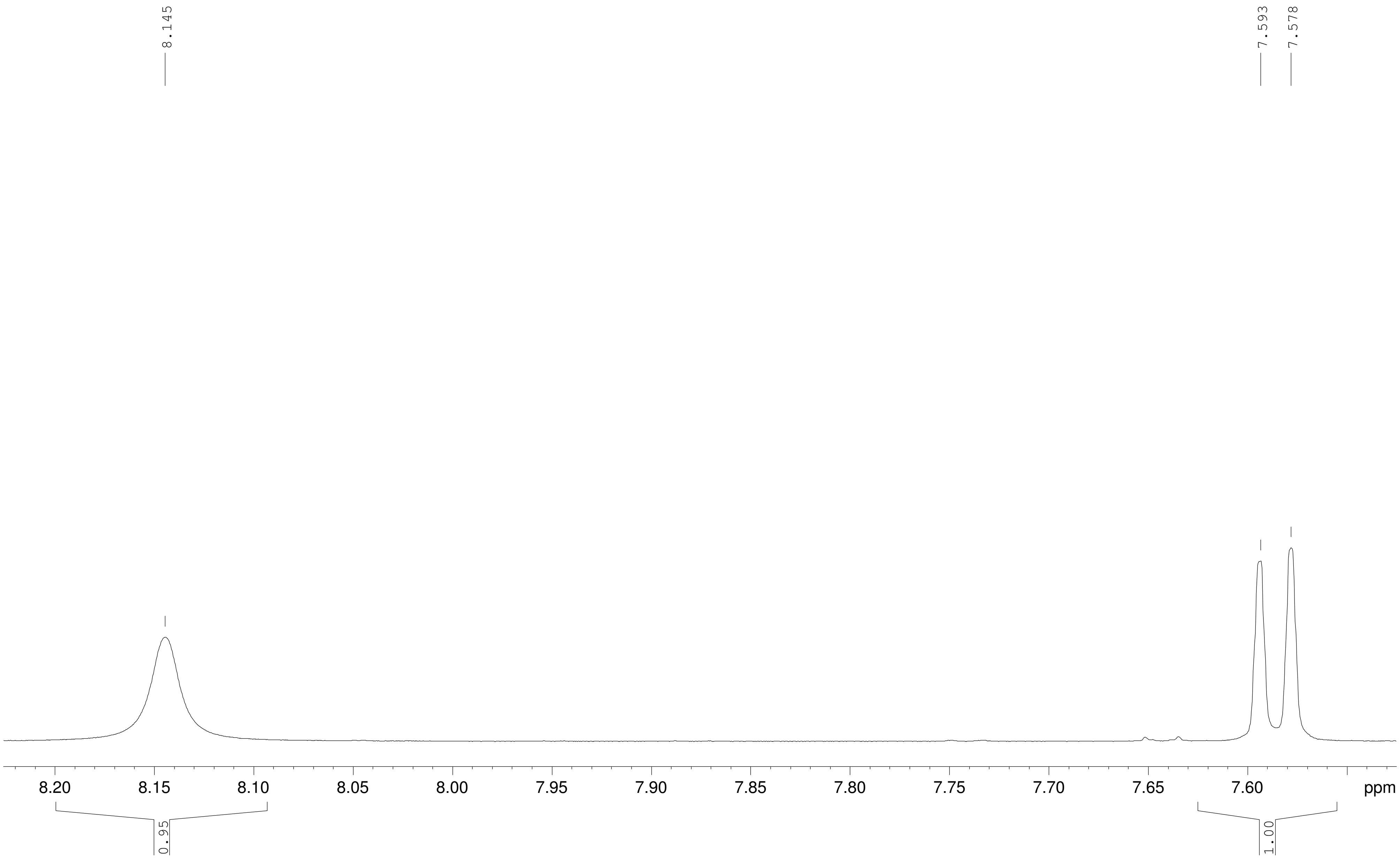

3538-1H
A-amid

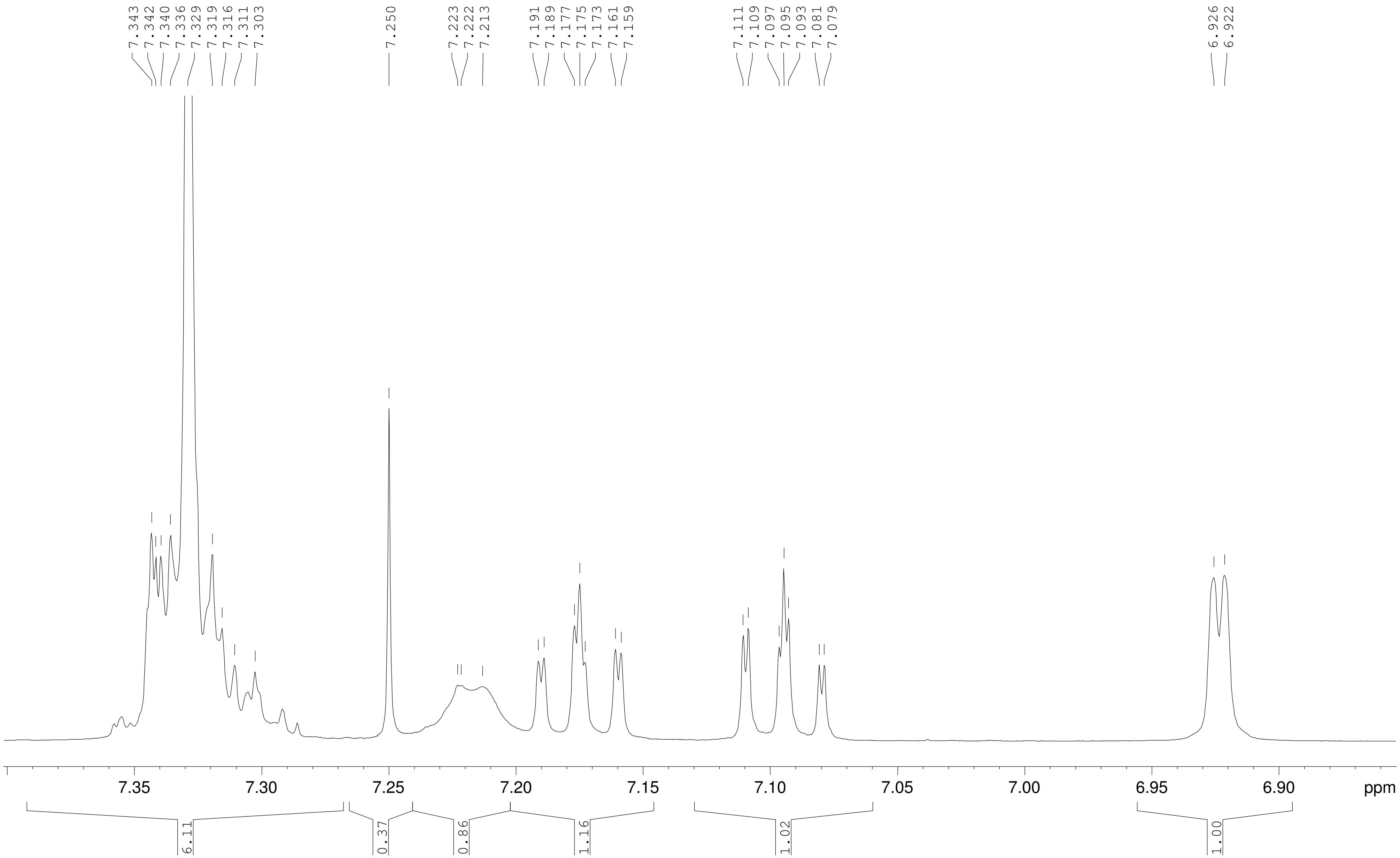

3538-1H
A-amid

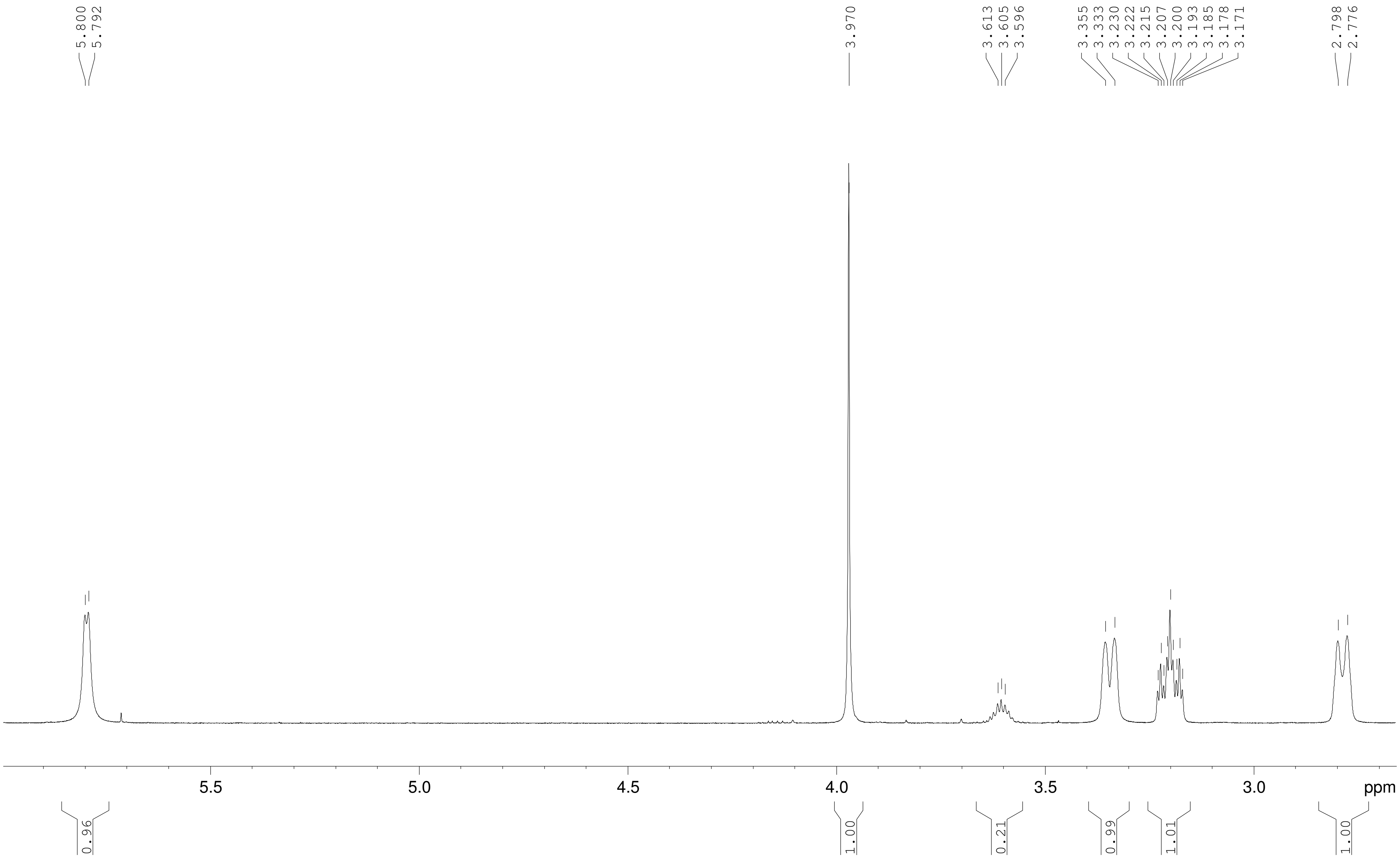

3538-1H
A-amid

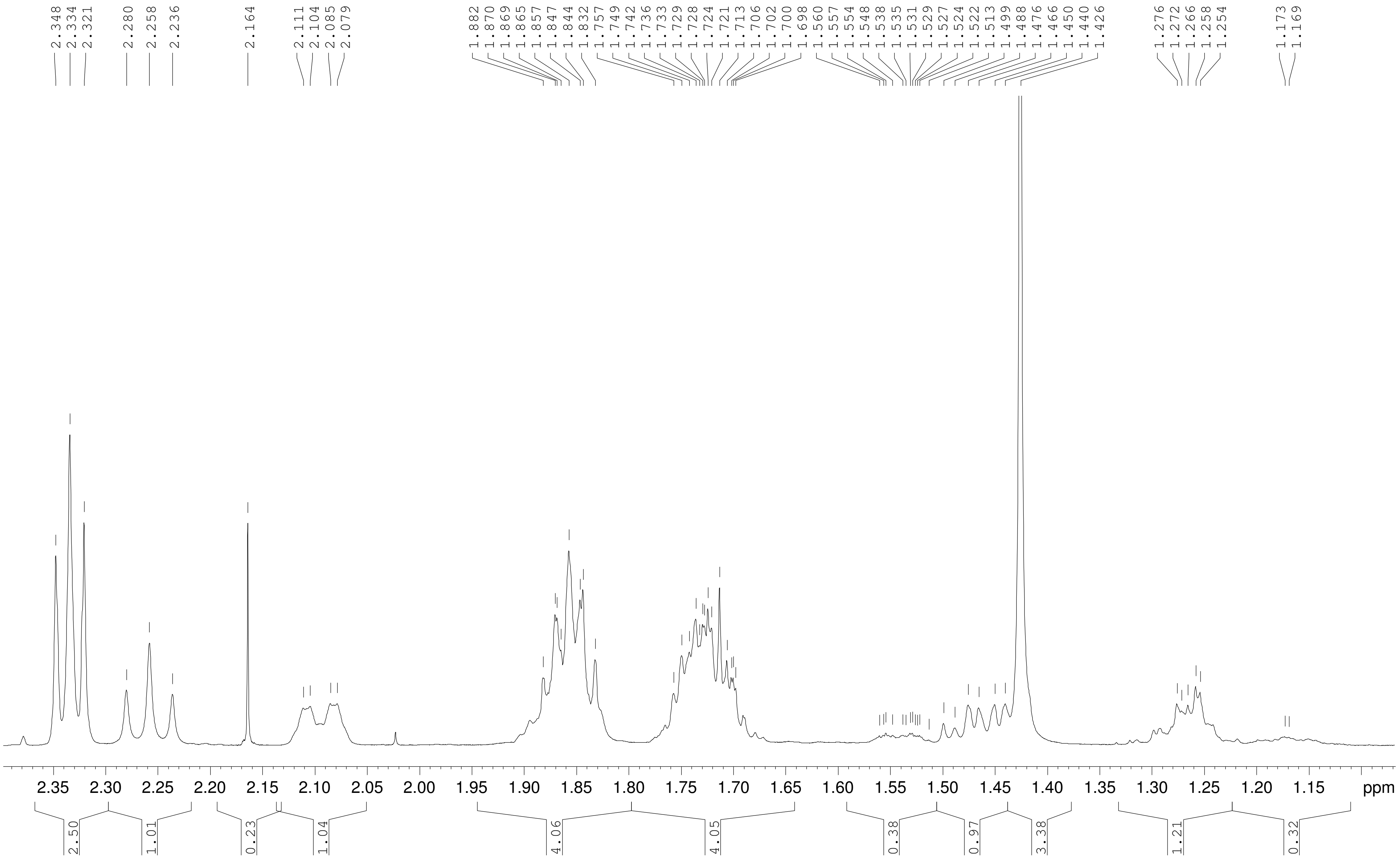

3538-13C
A-amid

Current Data Parameters
NAME 3538
EXPNO 2
PROCNO 1

F2 - Acquisition Parameters
Date_ 20170217
PROBHD 5 mm PABBI 1H/
PULPROG zg_pi_CPD
SOLVENT CDCl3
TE 298.2 K
SWH 31249.381 Hz
AQ 1.0485969 sec
NS 800
FIDRES 0.476828 Hz
D1 1.50000000 sec
D11 0.03000000 sec
D14 0.00050000 sec

======== CHANNEL f1 ========
SFO1 125.7904831 MHz
NUC1 13C
P1 15.00 usec
P2 30.00 usec
PLW1 78.52400208 W

======== CHANNEL f2 ========
SFO2 500.2020008 MHz
NUC2 1H
PLW2 12.50000000 W
PLW12 0.12500000 W

F2 - Processing parameters
SI 65536
LB 0.50 Hz

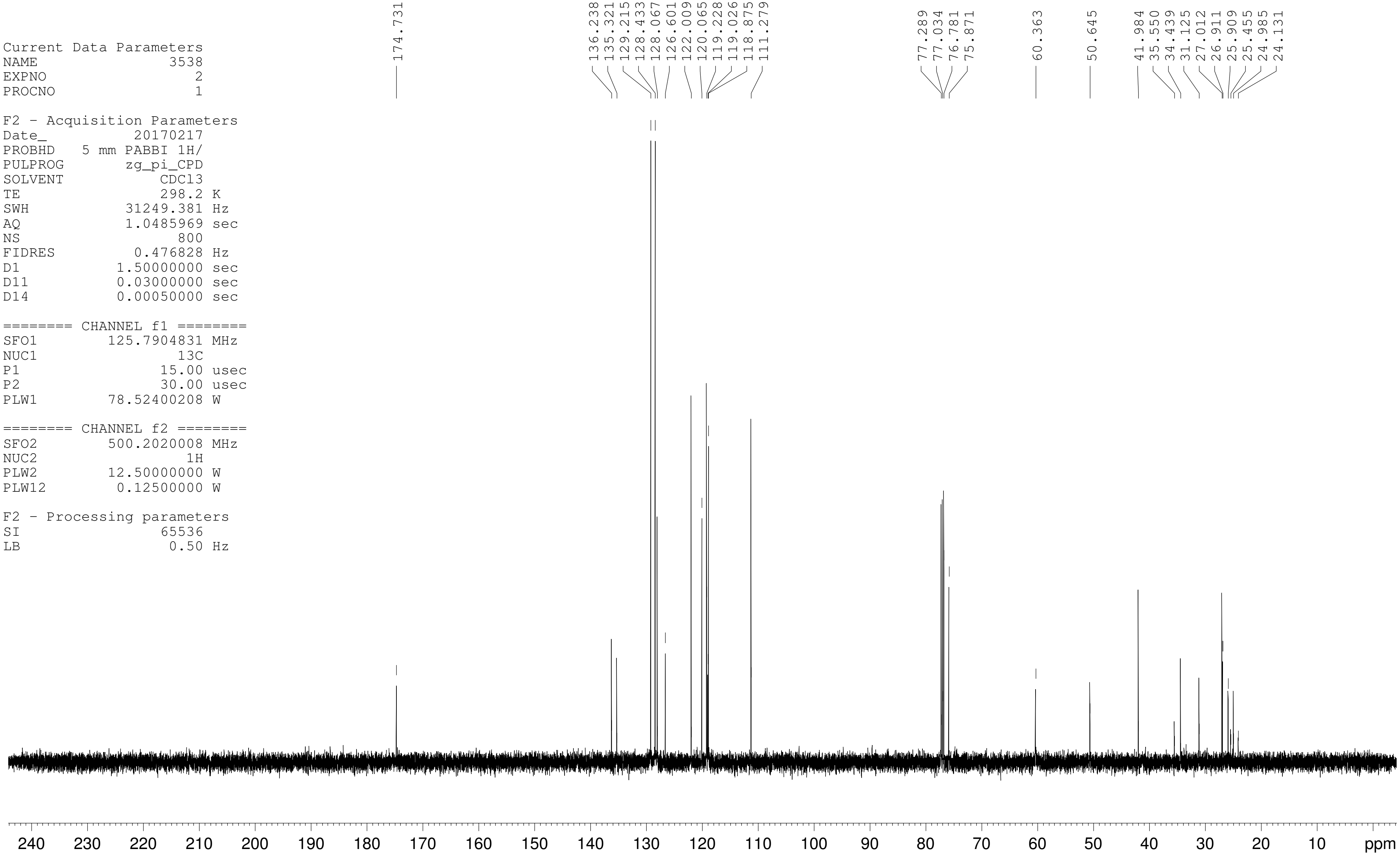

3538-13C
A-amid

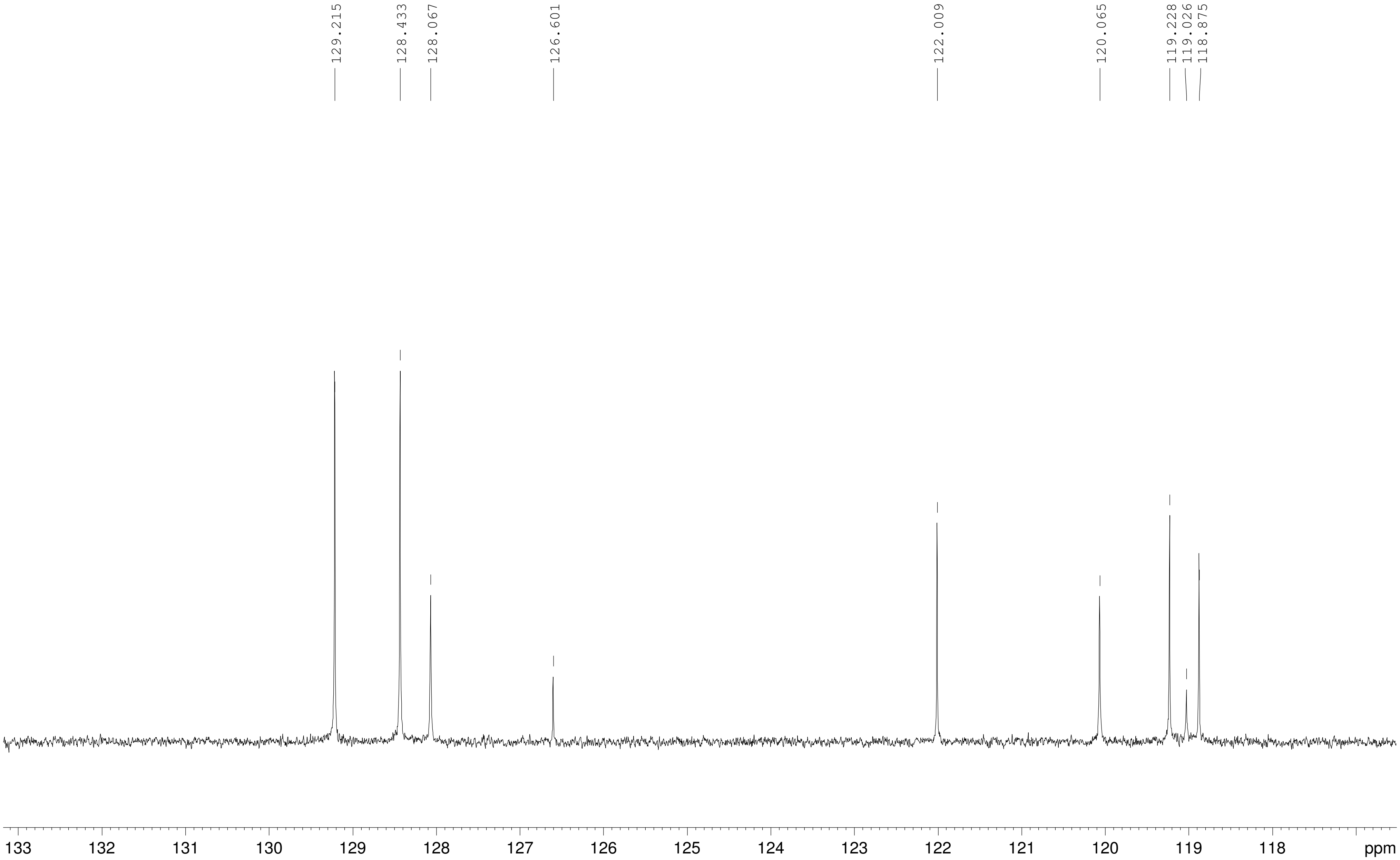

3538-13C
A-amid

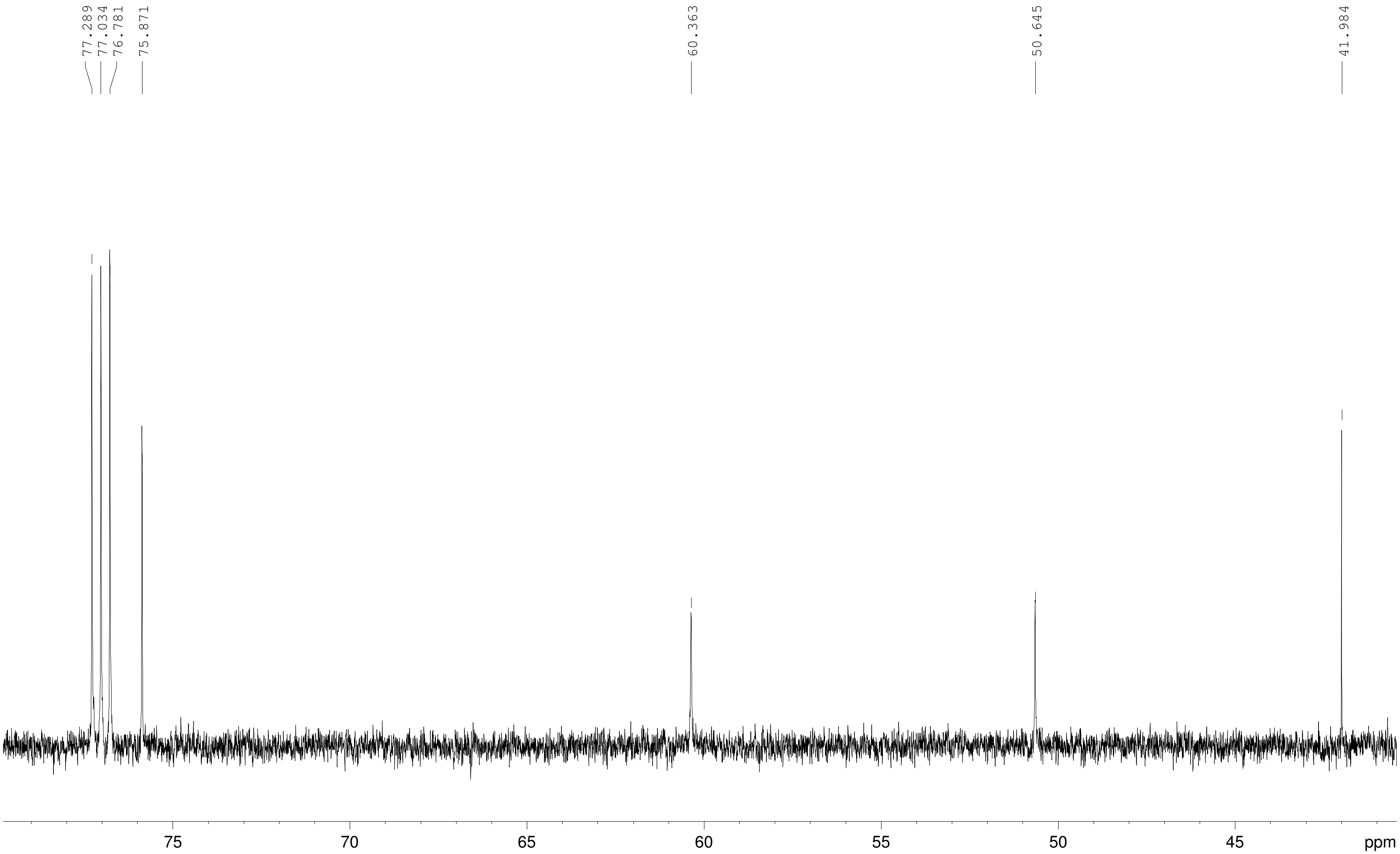

3538-13C
A-amid

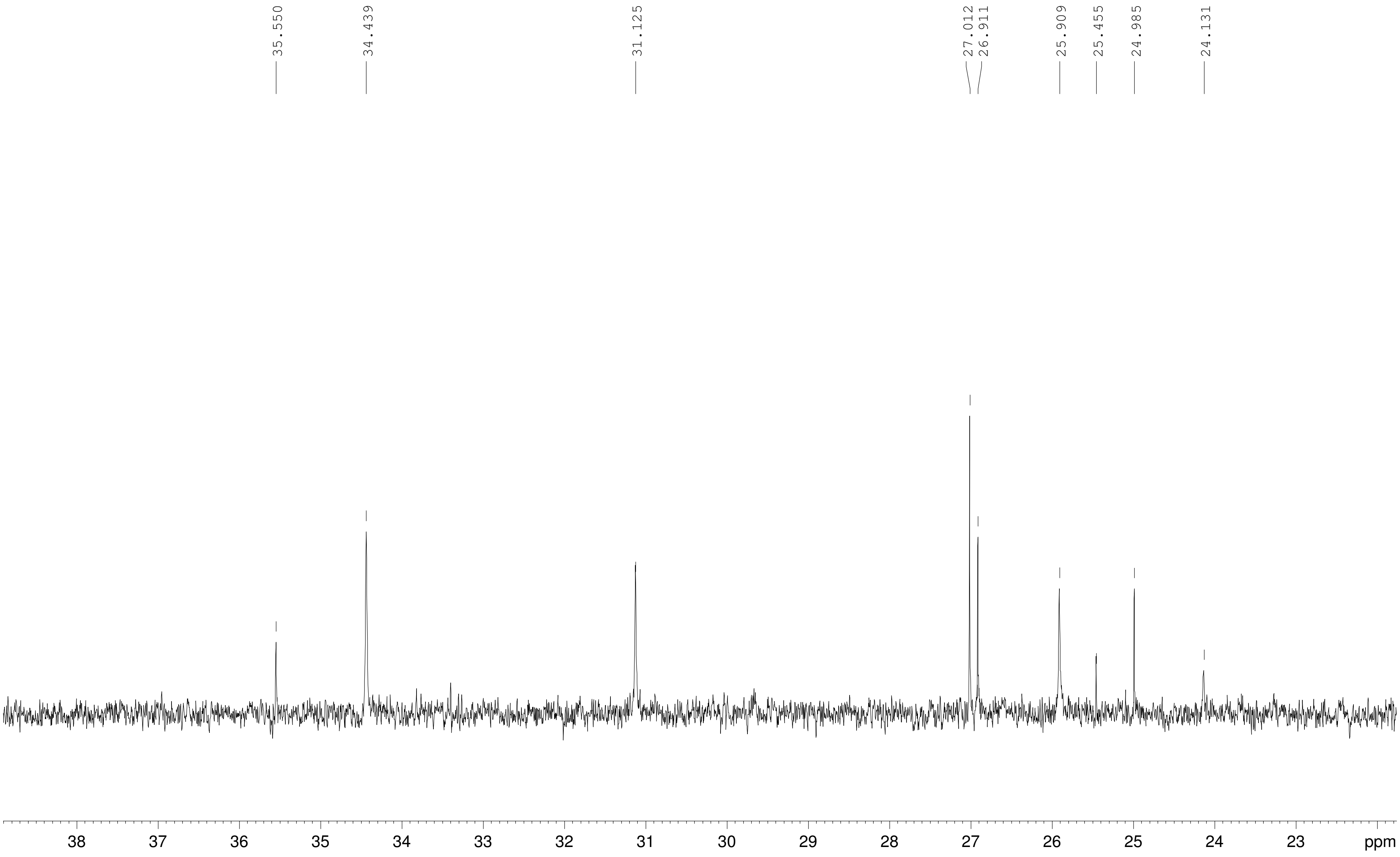

3538-COSY
A-amid

Current Data Parameters
NAME 3538
EXPNO 4
PROCNO 1

F2 - Acquisition Parameters
Date_ 20170217
PROBHD 5 mm PABBI 1H/
PULPROG cosygpppqf
SOLVENT CDCl3
TE 297.8 K
SWH 5154.639 Hz
AQ 0.1986560 sec
NS 1
FIDRES 2.516914 Hz
D0 0.00000300 sec
D1 2.00000000 sec
D11 0.03000000 sec
D12 0.00002000 sec
D13 0.00000400 sec
D16 0.00020000 sec

======== CHANNEL f1 ========
SFO1 500.2022372 MHz
NUC1 1H
P0 8.00 usec
P1 8.00 usec
P17 2500.00 usec
PLW1 12.69999981 W
PLW10 1.20239997 W

====== GRADIENT CHANNEL =====
P16 1000.00 usec

F1 - Acquisition parameters
TD 128
SFO1 500.2022 MHz
FIDRES 40.270618 Hz
SW 10.305 ppm
FnMODE QF

F2 - Processing parameters
SI 2048
LB 0 Hz

F1 - Processing parameters
SI 1024
MC2 QF
SF 500.2000171 MHz
WDW QSINE
SSB 0
LB 0 Hz
GB 0

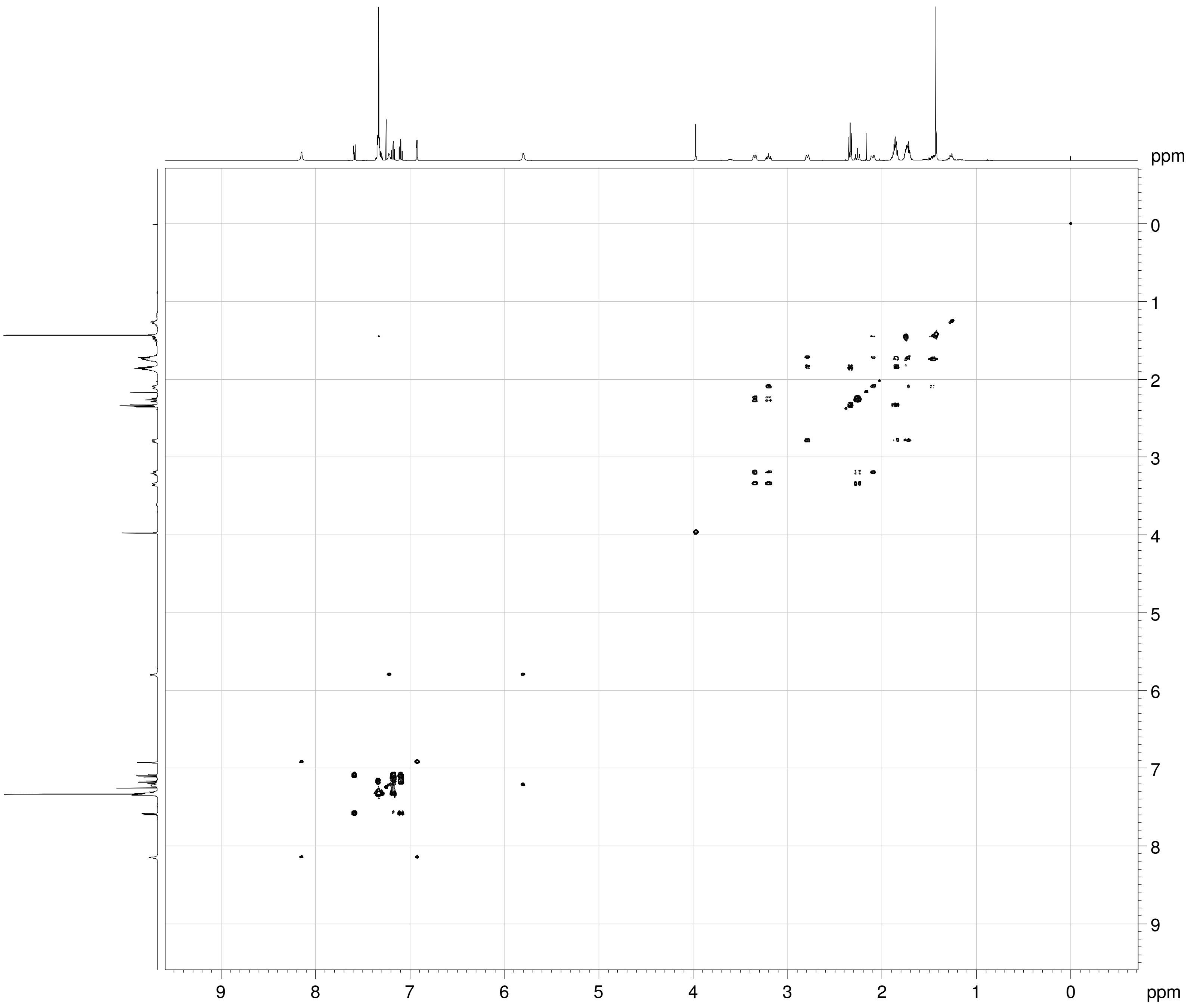

3538-COSY
A-amid

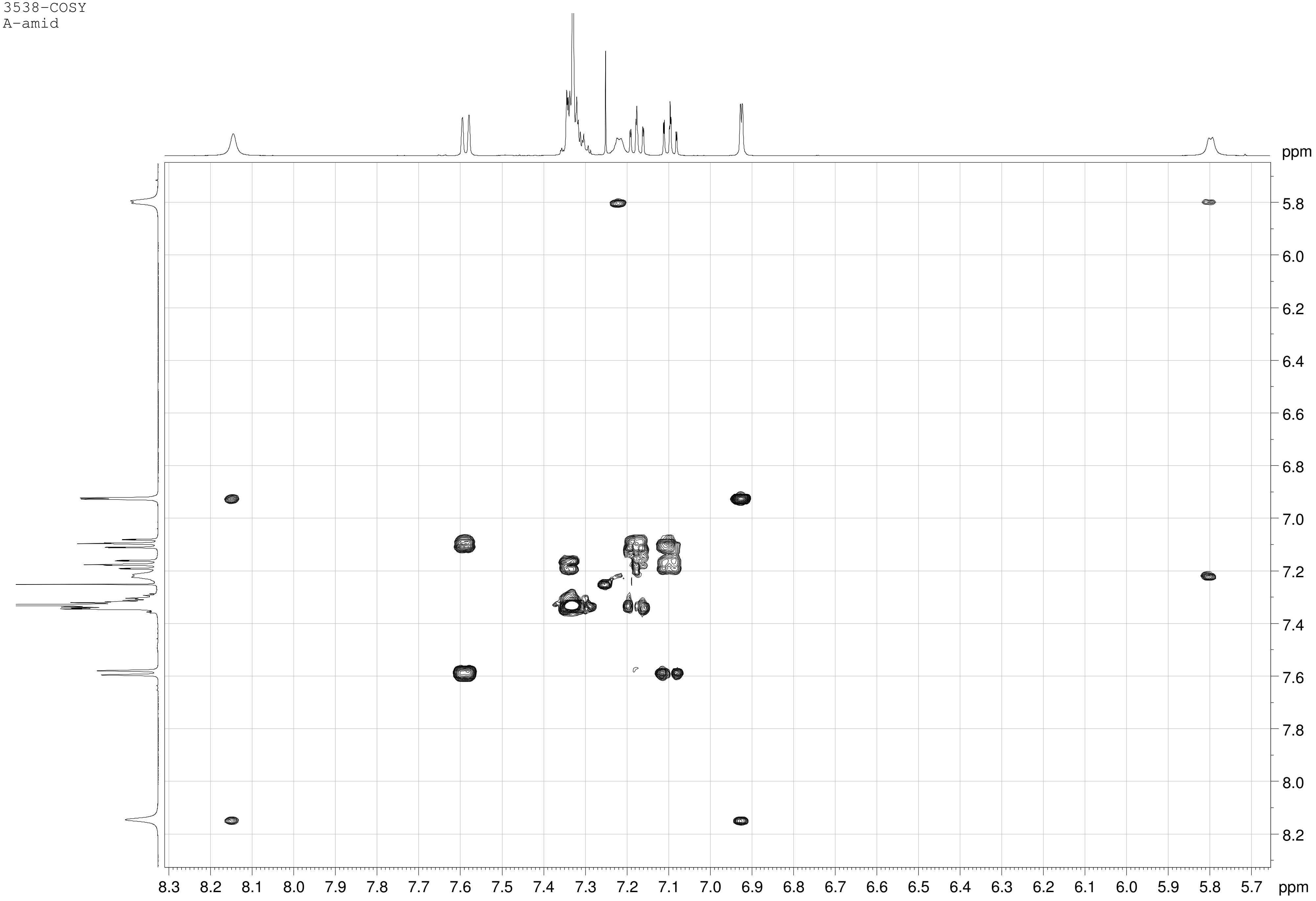

3538-COSY
A-amid

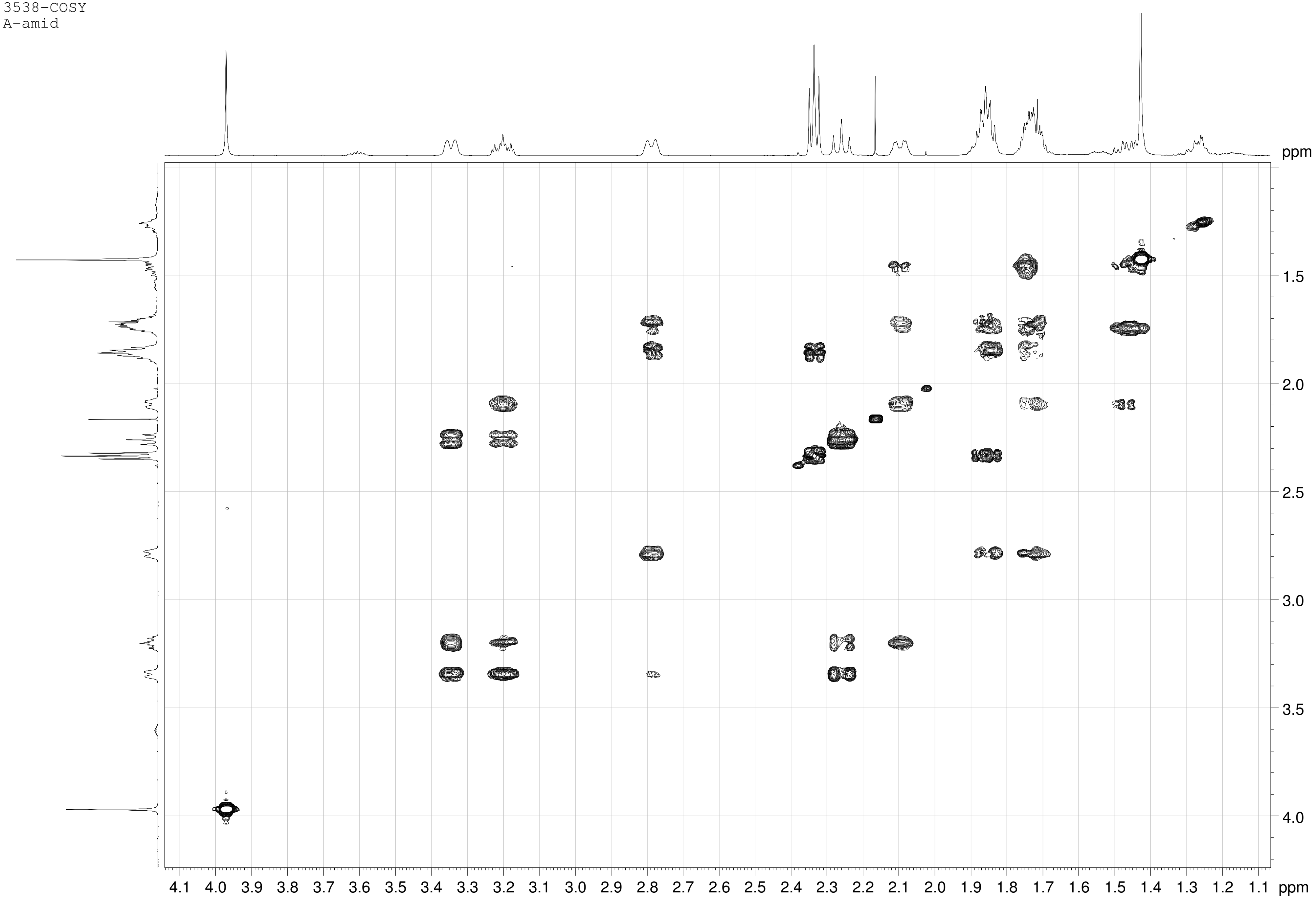

3538-HSQC
A-amid

Current Data Parameters
NAME 3538
EXPNO 3
PROCNO 1

F2 - Acquisition Parameters
Date_ 20170217
PROBHD 5 mm PABBI 1H/
PULPROG hsqcedetgpsisp2.3
SOLVENT CDCl3
TE 298.6 K
SWH 5154.639 Hz
AQ 0.1986560 sec
NS 2
FIDRES 2.516914 Hz
D0 0.00000300 sec
D1 2.00000000 sec
D4 0.00172414 sec
D11 0.03000000 sec
D16 0.00020000 sec
D21 0.00360000 sec
D24 0.00089000 sec

======== CHANNEL f1 ========
SFO1 500.2022372 MHz
NUC1 1H
P1 8.00 usec
P2 16.00 usec
P28 20.00 usec
PLW1 12.69999981 W

======== CHANNEL f2 ========
SFO2 125.7880076 MHz
NUC2 13C
P3 15.00 usec
P14 500.00 usec
P24 2000.00 usec
P31 1900.00 usec
PLW0 0 W
PLW2 78.52400208 W
PLW12 3.60570002 W

====== GRADIENT CHANNEL =====
P16 1000.00 usec
P19 600.00 usec

F1 - Acquisition parameters
TD 128
SFO1 125.788 MHz
FIDRES 198.286804 Hz
SW 201.774 ppm
FnMODE Echo-Antiecho

F2 - Processing parameters

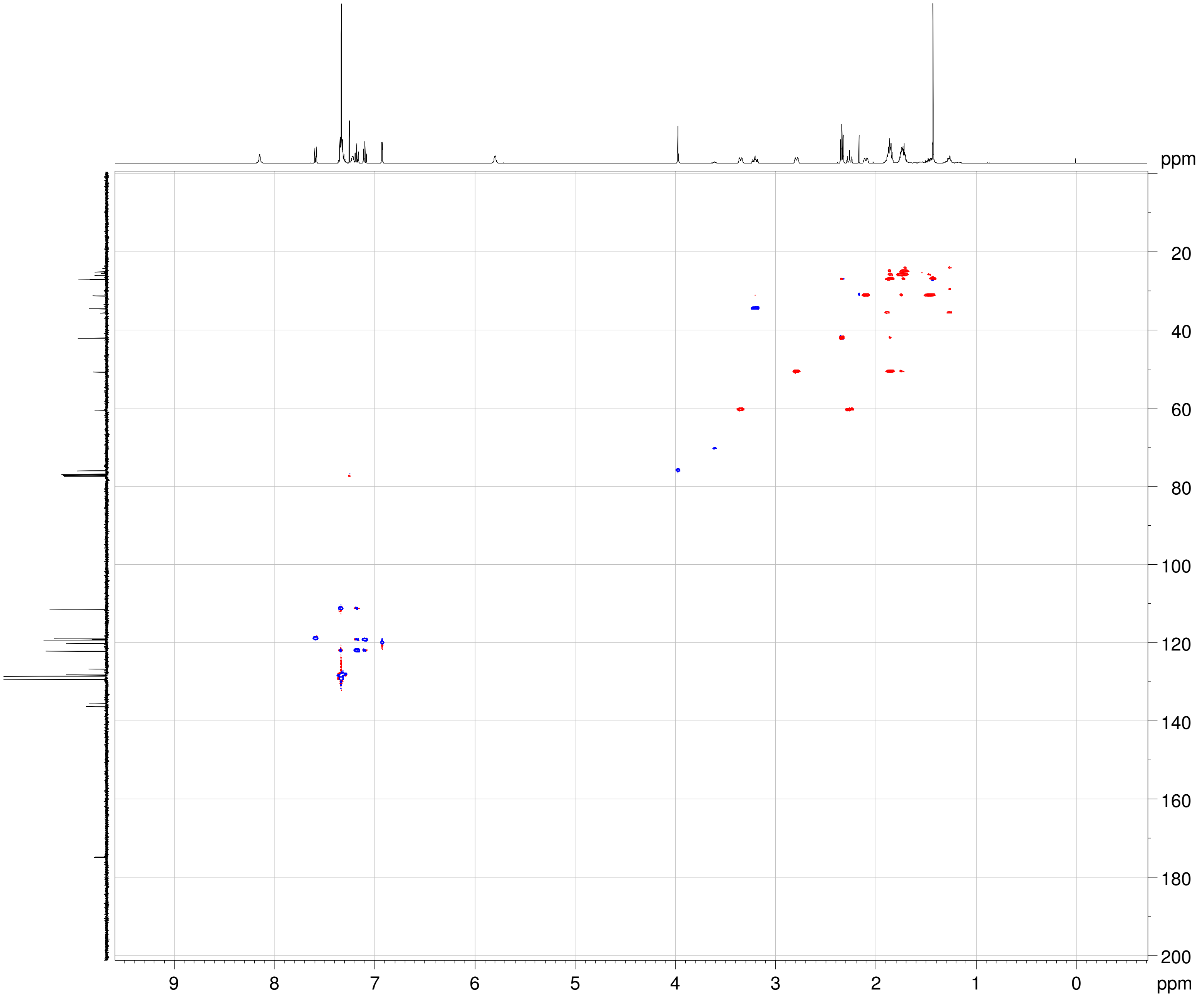

3538-HSQC
A-amid

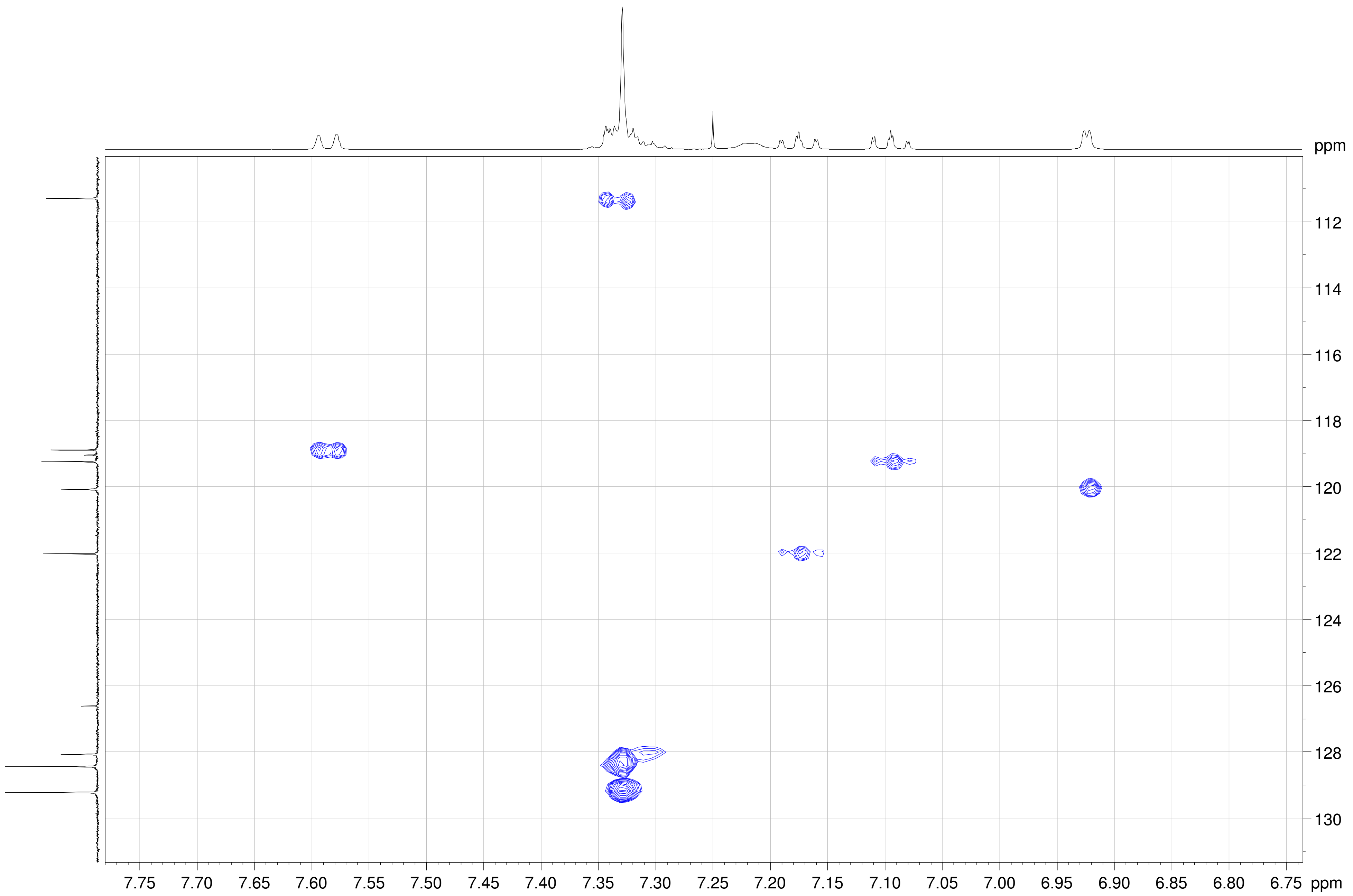

3538-HSQC
A-amid

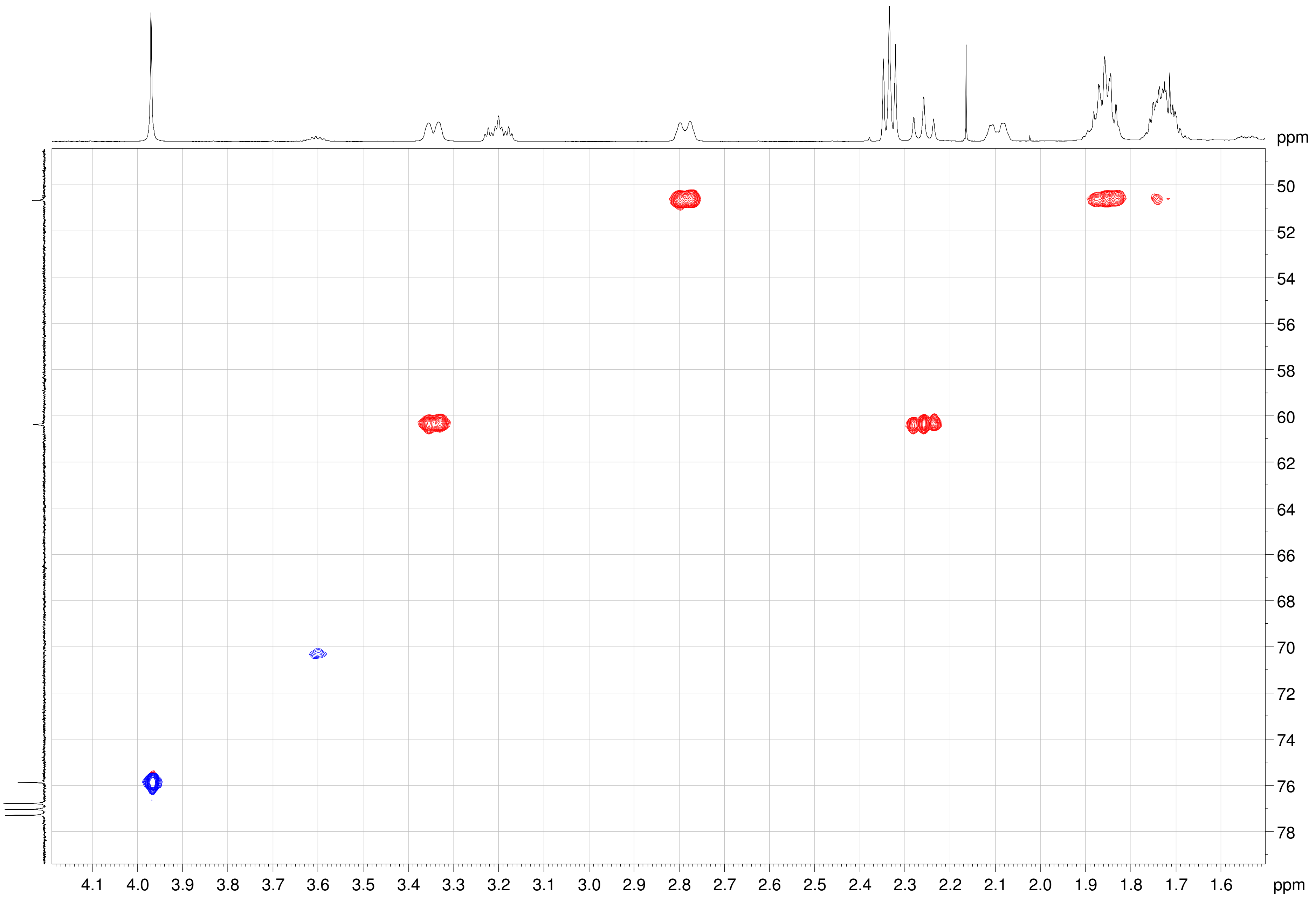

3538-HSQC
A-amid

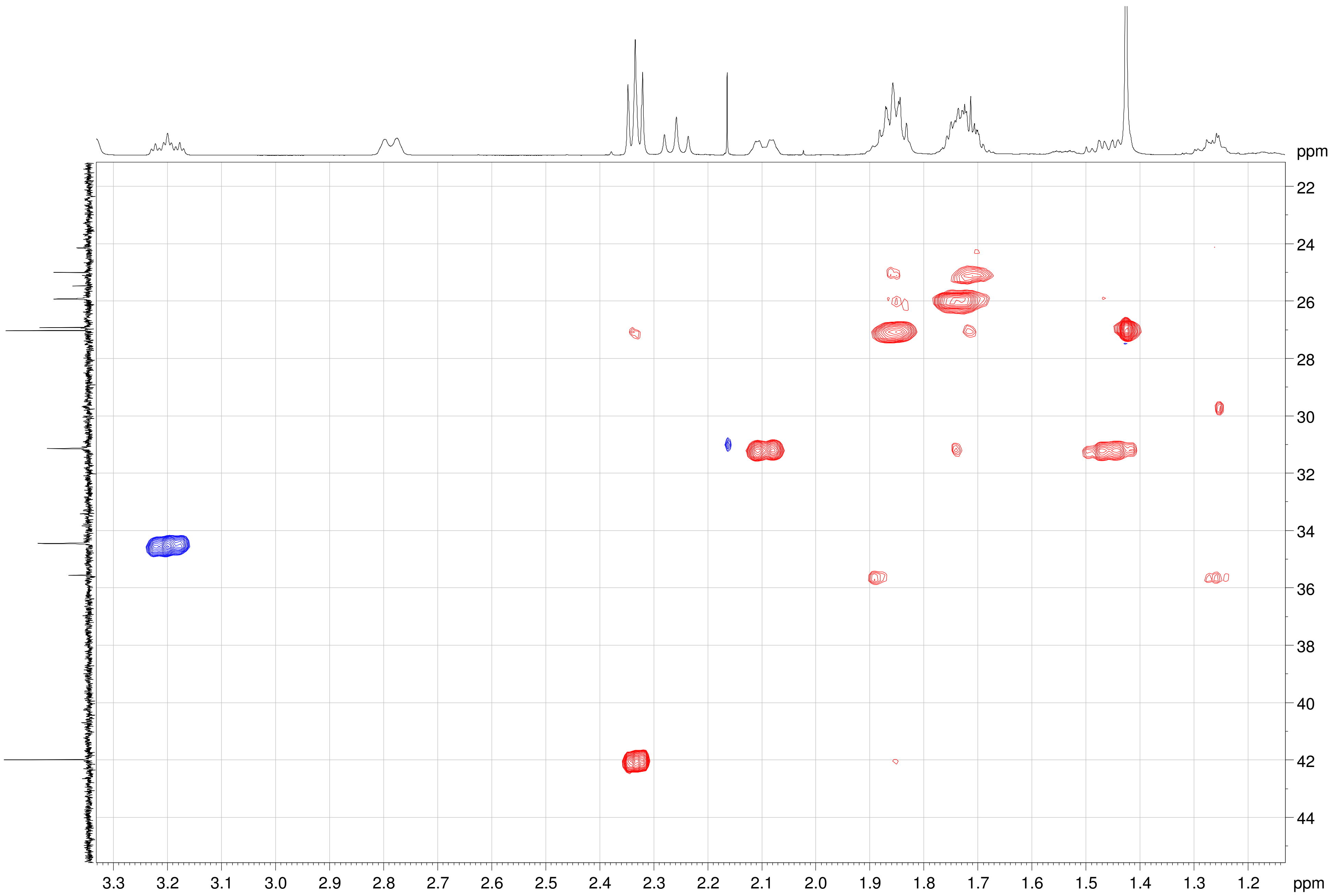

(3S,2R)-5a

3539-1H
B-amid

```
Current Data Parameters
NAME              3539
EXPNO                1
PROCNO               1

F2 - Acquisition Parameters
Date_          20170217
PROBHD   5 mm PABBI 1H/
PULPROG           zg30
SOLVENT          CDCl3
TE               298.2 K
SWH          10000.000 Hz
AQ           3.2767999 sec
NS                   8
FIDRES        0.152588 Hz
D1       0 sec

======== CHANNEL f1 =======
SFO1       500.2030889 MHz
NUC1                1H
P1                8.00 use
PLW1       12.69999981 W

F2 - Processing parameters
SI               65536
LB       0 Hz
```

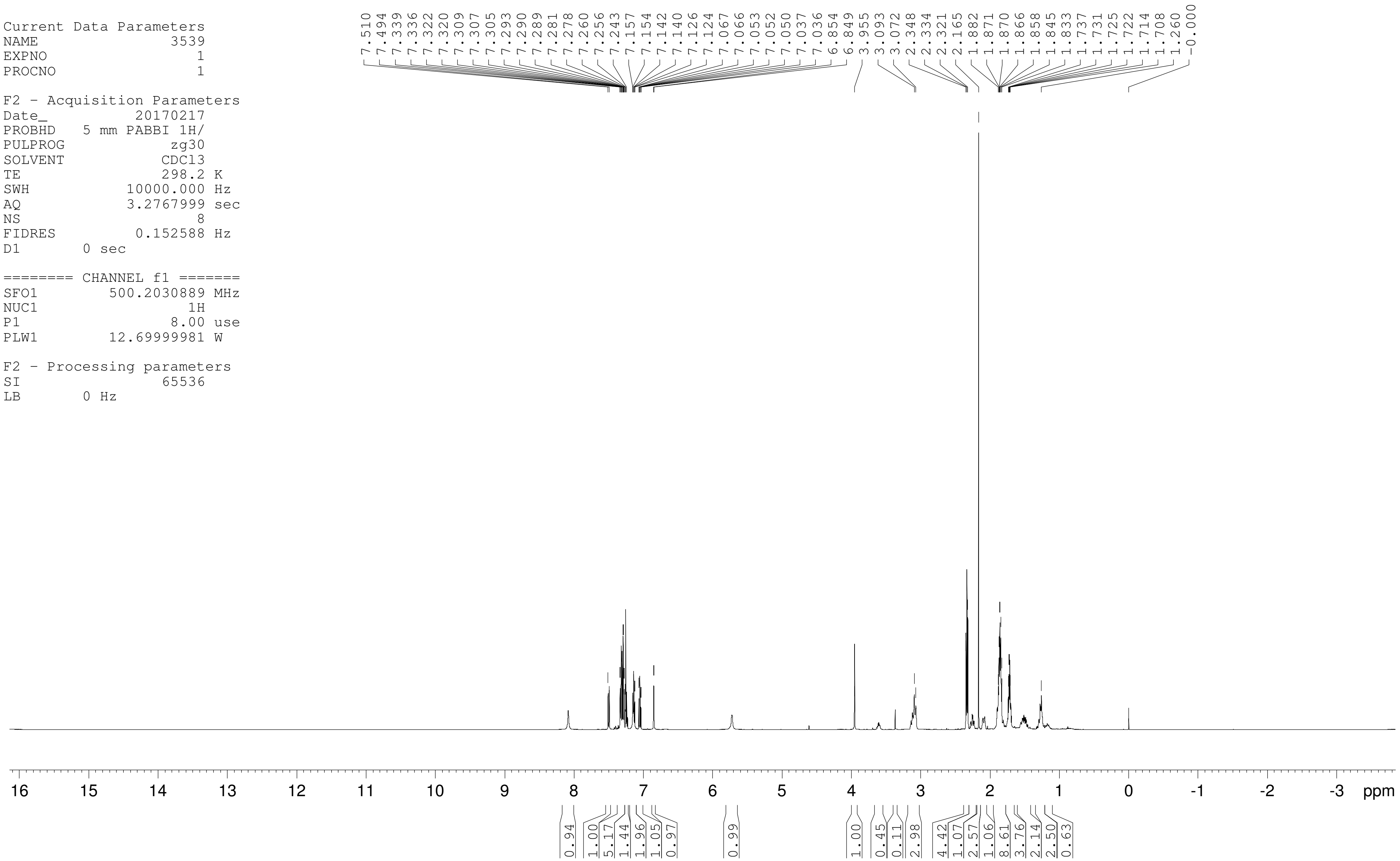

3539-1H
B-amid

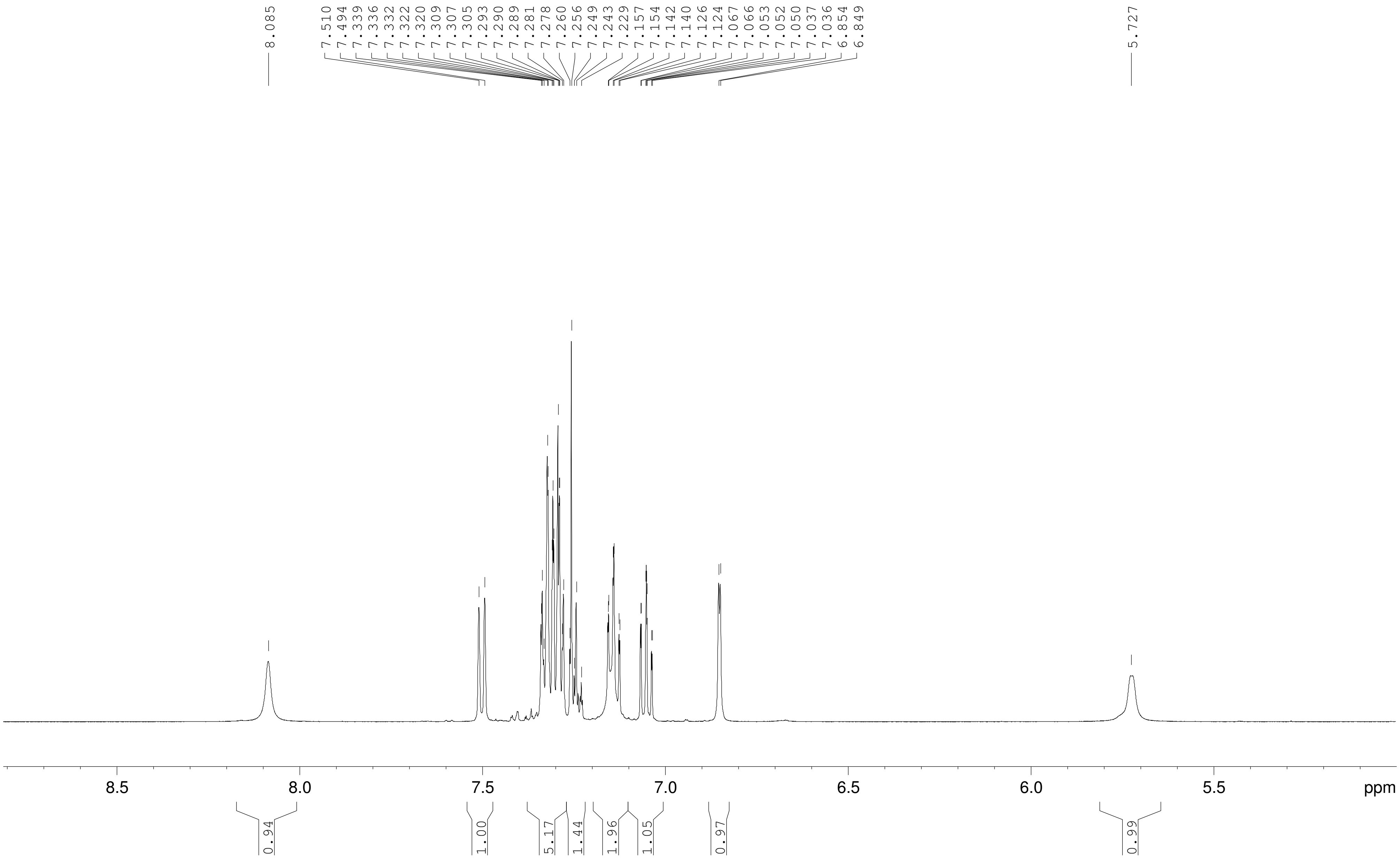

3539-1H
B-amid

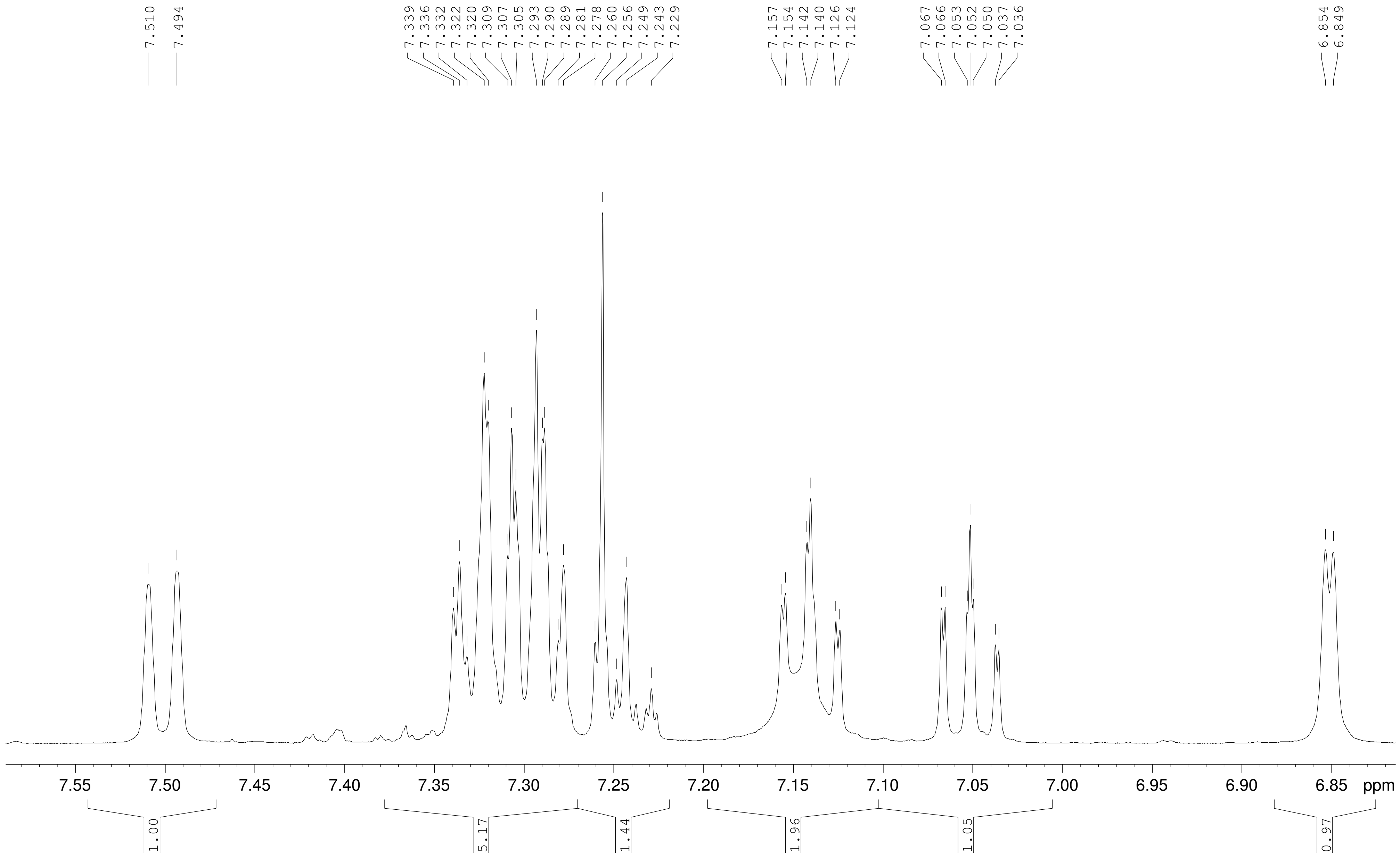

3539-1H
B-amid

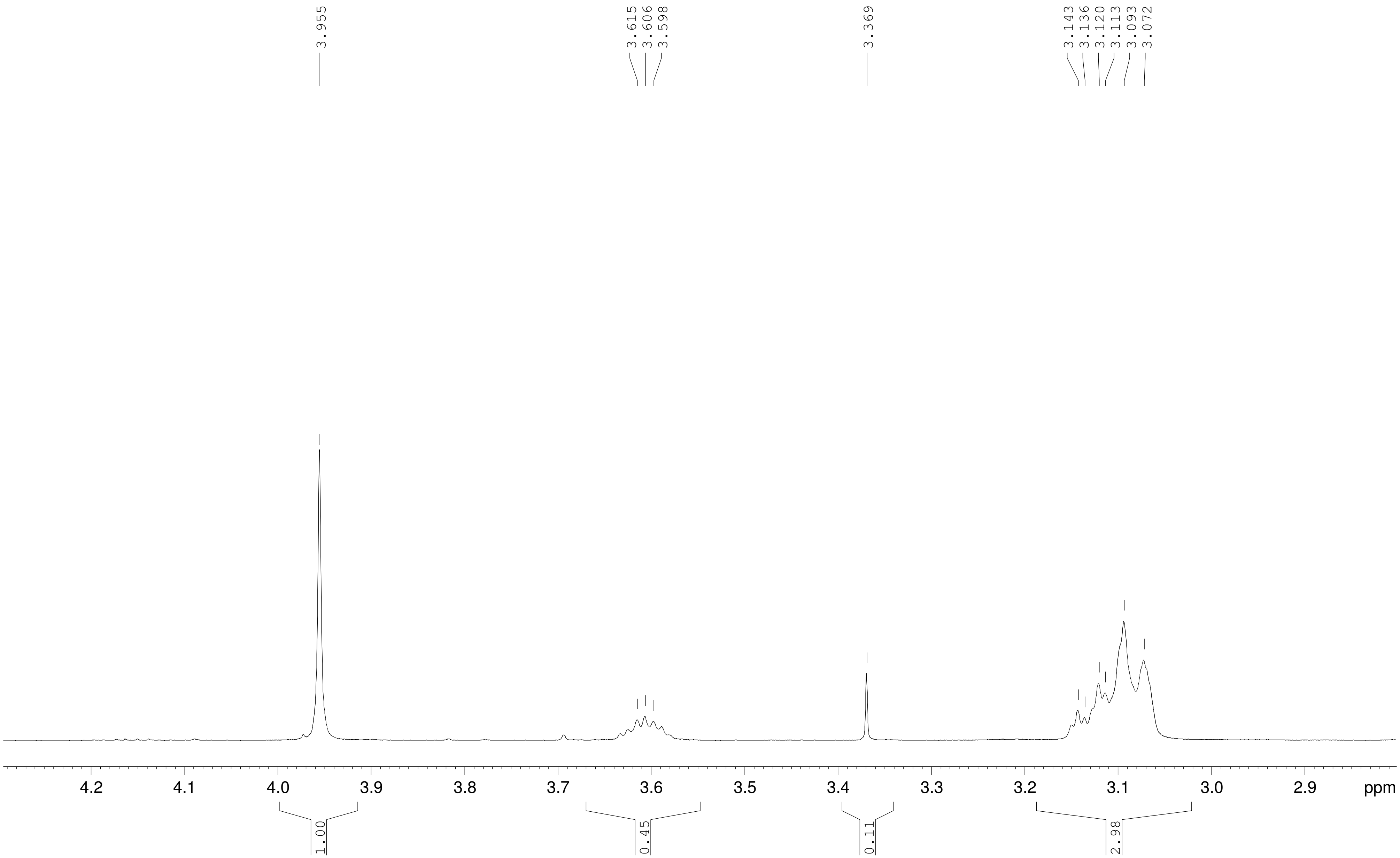

3539-1H
B-amid

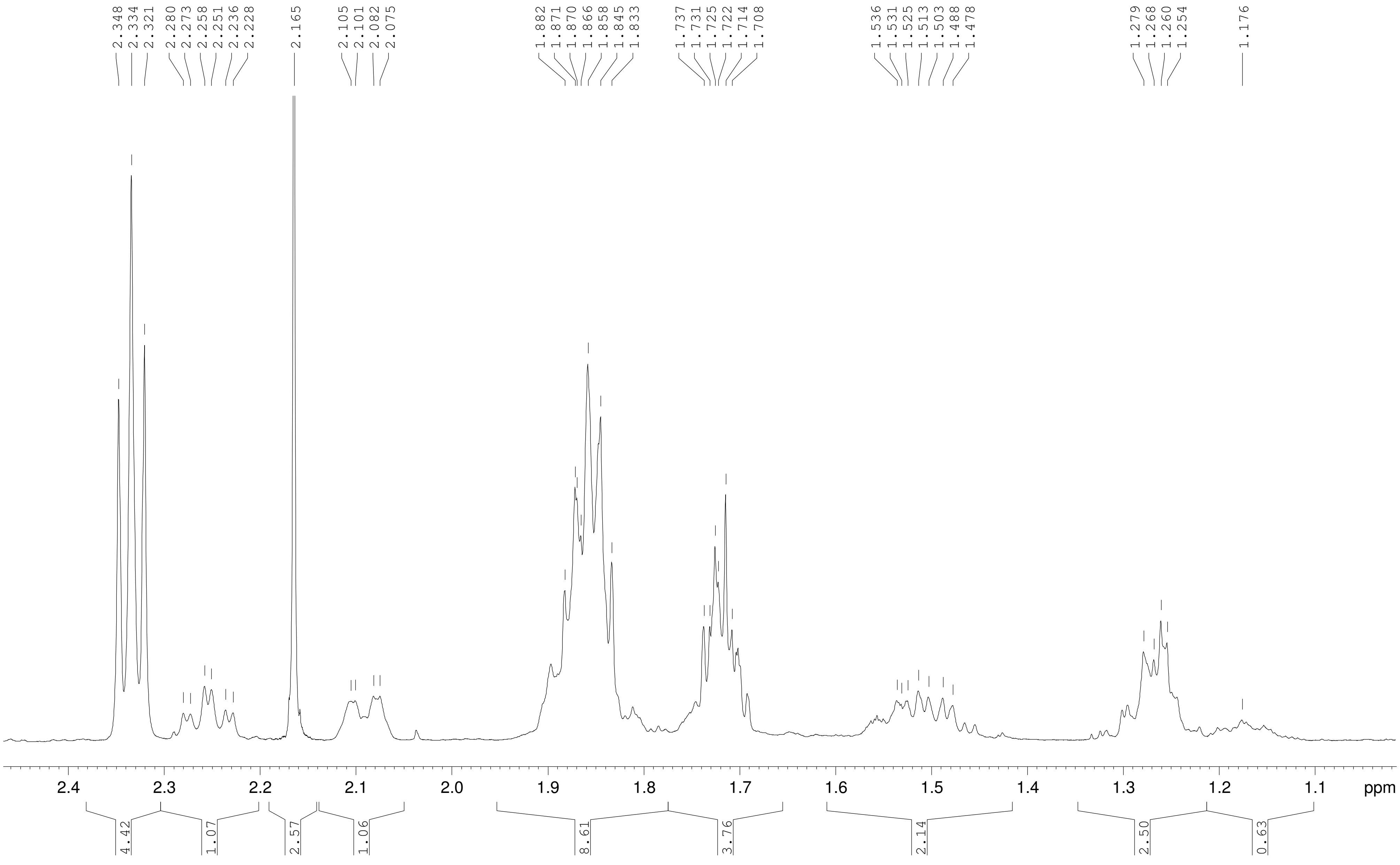

3539-13C
B-amid

Current Data Parameters
NAME 3539
EXPNO 2
PROCNO 1

F2 - Acquisition Parameters
Date_ 20170217
PROBHD 5 mm PABBI 1H/
PULPROG zg_pi_CPD
SOLVENT CDCl3
TE 298.2 K
SWH 31249.381 Hz
AQ 1.0485969 sec
NS 2040
FIDRES 0.476828 Hz
D1 1.50000000 sec
D11 0.03000000 sec
D14 0.00050000 sec

======== CHANNEL f1 ========
SFO1 125.7904831 MHz
NUC1 13C
P1 15.00 usec
P2 30.00 usec
PLW1 78.52400208 W

======== CHANNEL f2 ========
SFO2 500.2020008 MHz
NUC2 1H
PLW2 12.50000000 W
PLW12 0.12500000 W

F2 - Processing parameters
SI 65536
LB 0.50 Hz

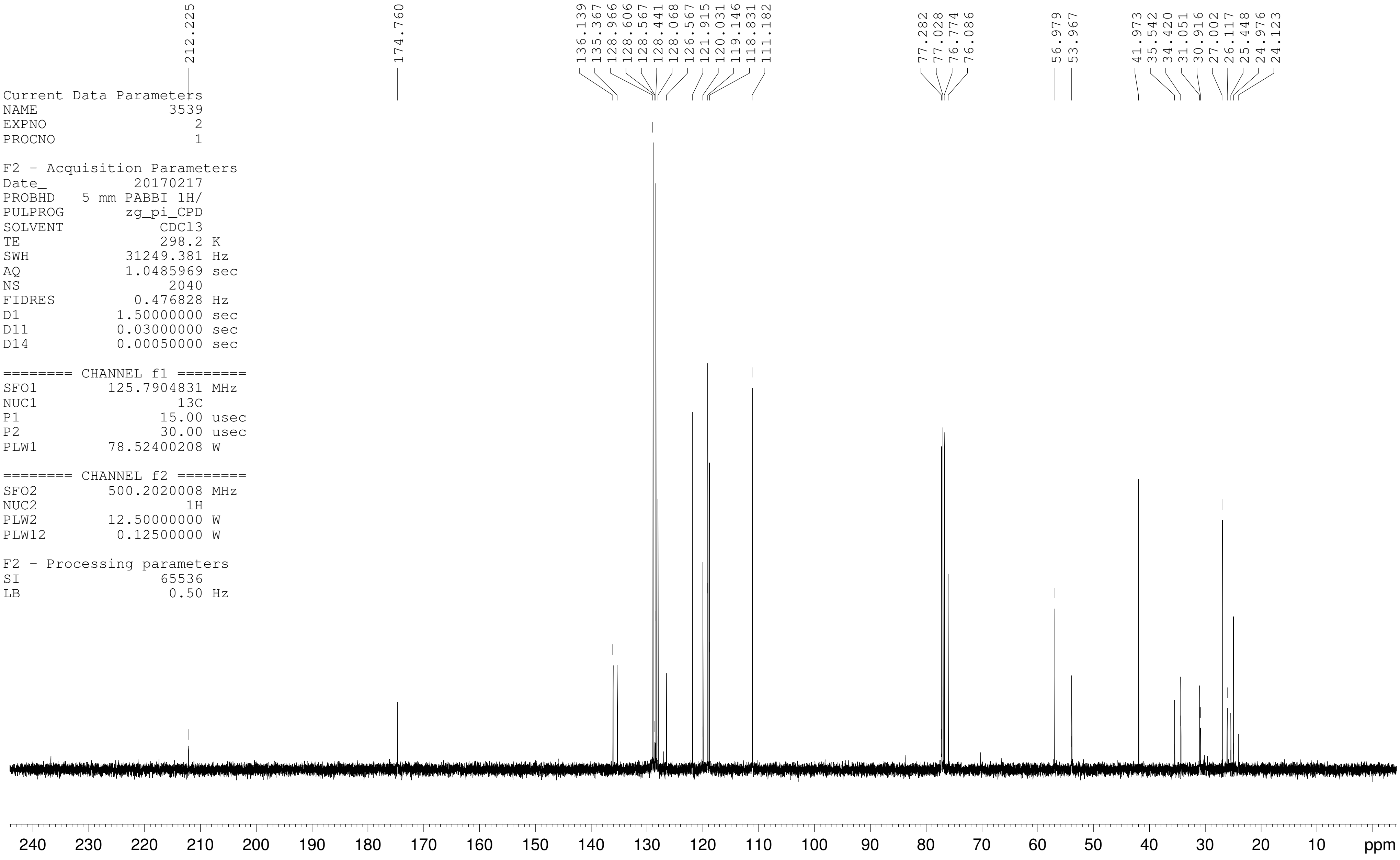

3539-13C
B-amid

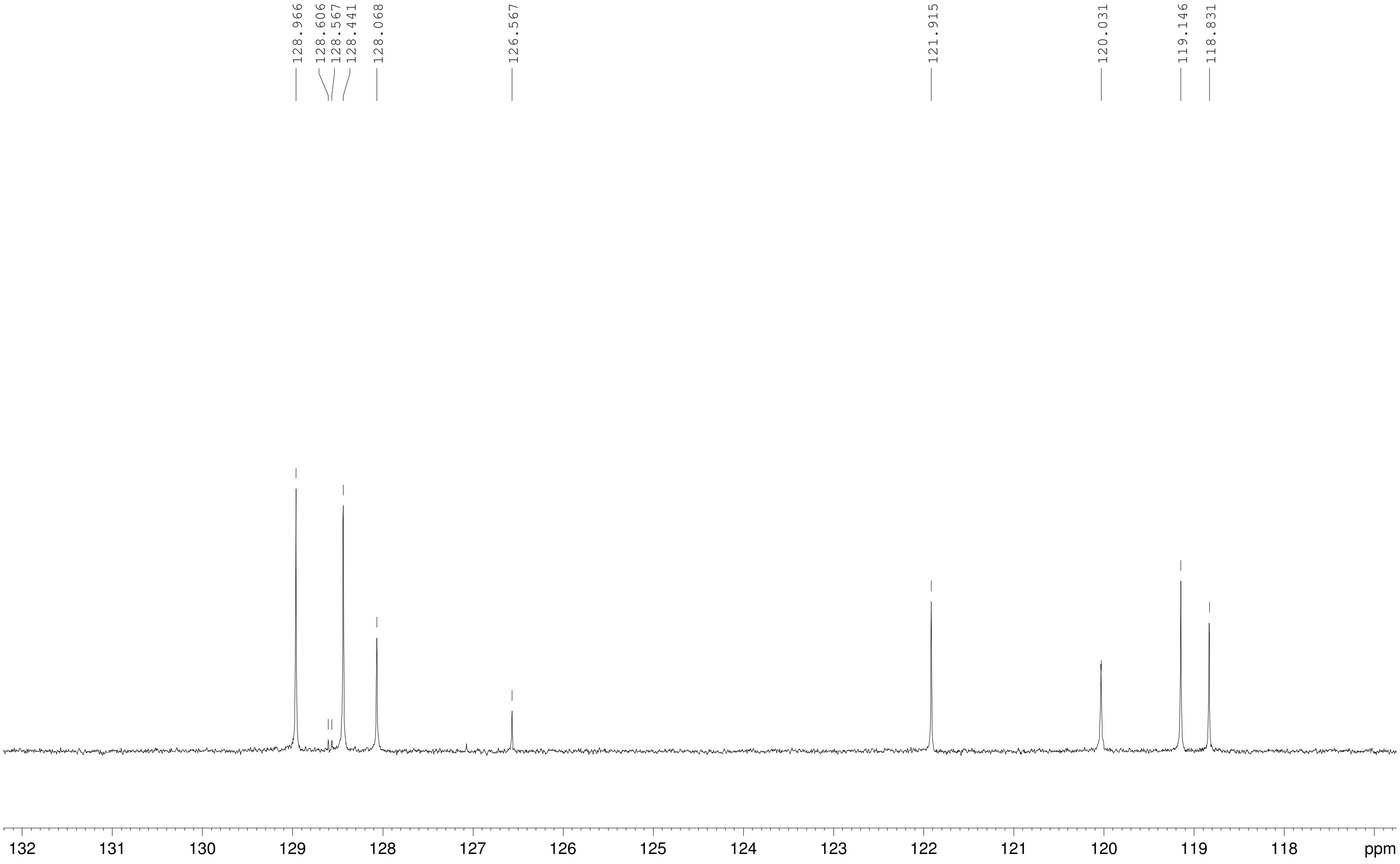

3539-13C
B-amid

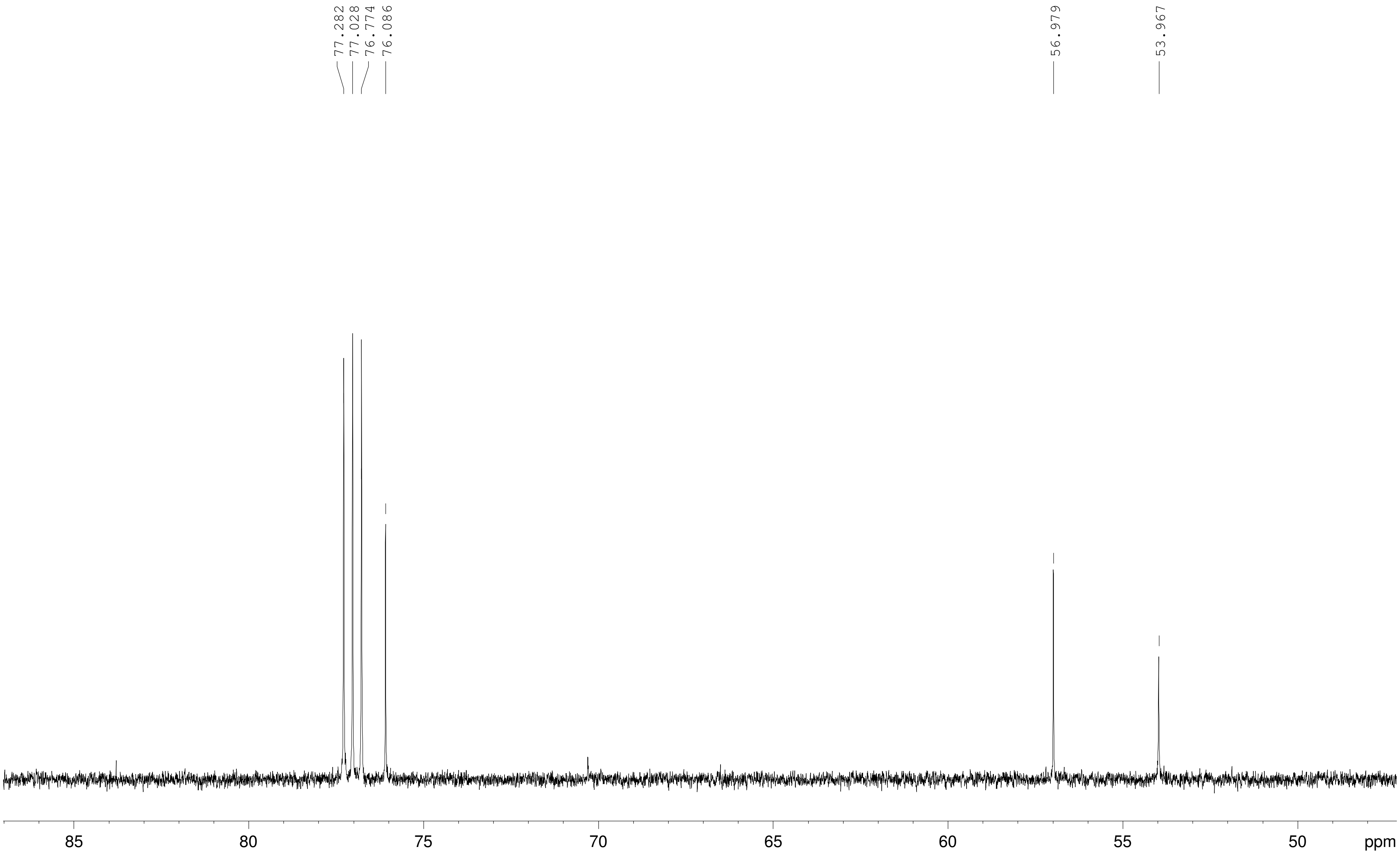

3539-13C
B-amid

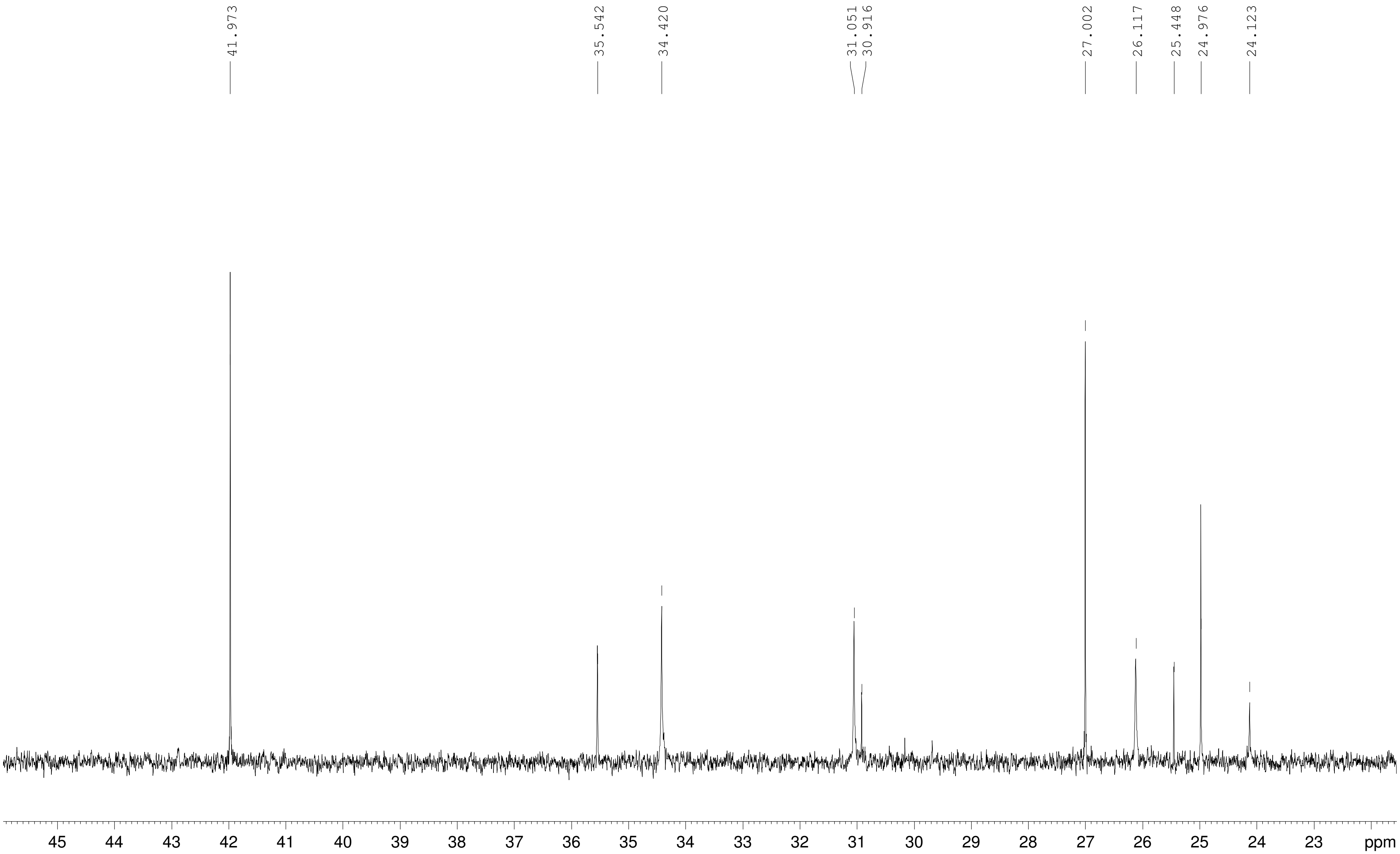

3539-COSY
B-amid

Current Data Parameters
NAME 3539
EXPNO 4
PROCNO 1

F2 - Acquisition Parameters
Date_ 20170217
PROBHD 5 mm PABBI 1H/
PULPROG cosygpppqf
SOLVENT CDCl3
TE 298.1 K
SWH 5151.099 Hz
AQ 0.1987925 sec
NS 1
FIDRES 2.515185 Hz
D0 0.00000300 sec
D1 2.00000000 sec
D11 0.03000000 sec
D12 0.00002000 sec
D13 0.00000400 sec
D16 0.00020000 sec

======== CHANNEL f1 ========
SFO1 500.2022151 MHz
NUC1 1H
P0 8.00 usec
P1 8.00 usec
P17 2500.00 usec
PLW1 12.69999981 W
PLW10 1.20239997 W

====== GRADIENT CHANNEL =====
P16 1000.00 usec

F1 - Acquisition parameters
TD 128
SFO1 500.2022 MHz
FIDRES 40.229145 Hz
SW 10.294 ppm
FnMODE QF

F2 - Processing parameters
SI 2048
LB 0 Hz

F1 - Processing parameters
SI 1024
MC2 QF
SF 500.2000140 MHz
WDW QSINE
SSB 0
LB 0 Hz
GB 0

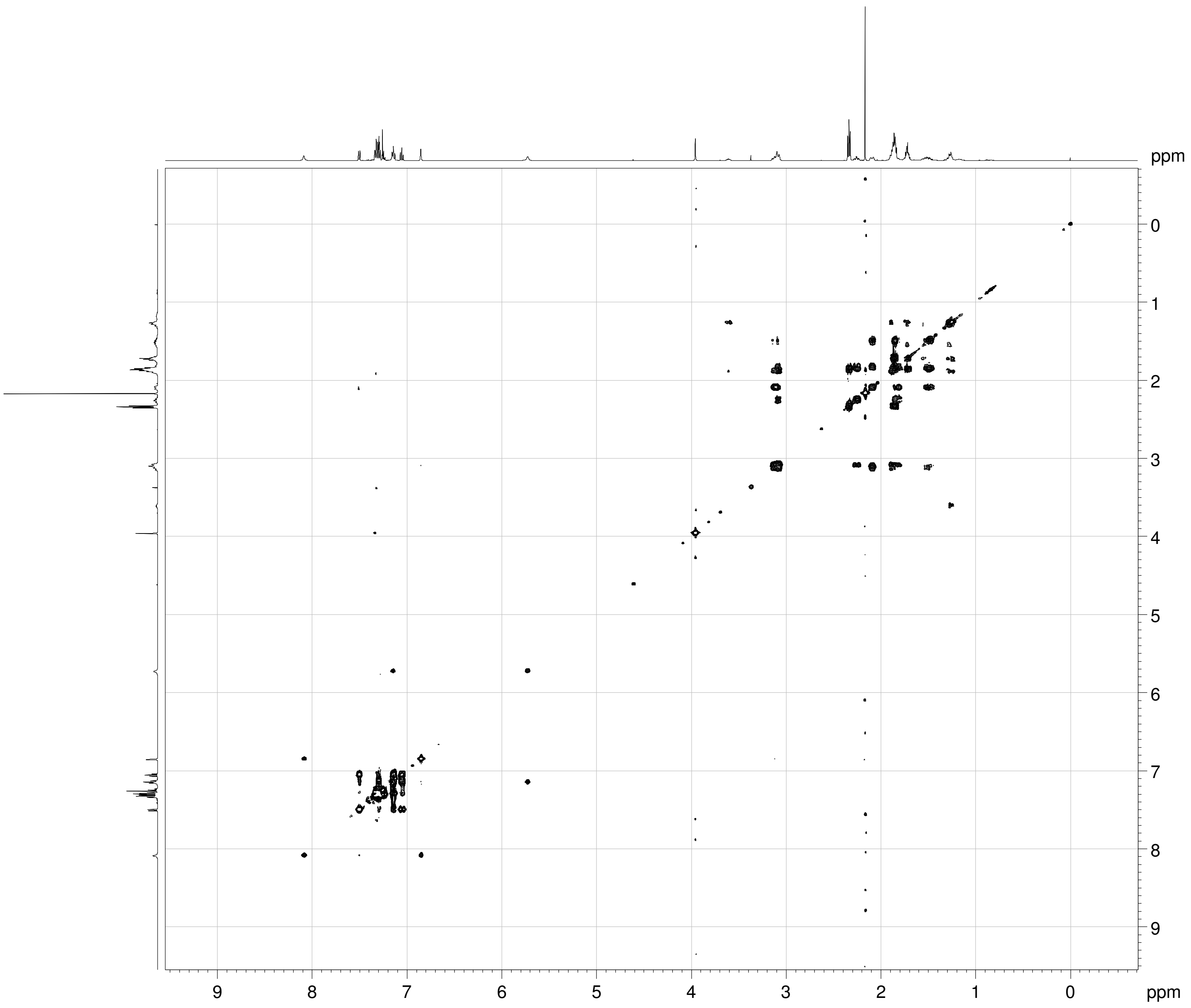

3539-COSY
B-amid

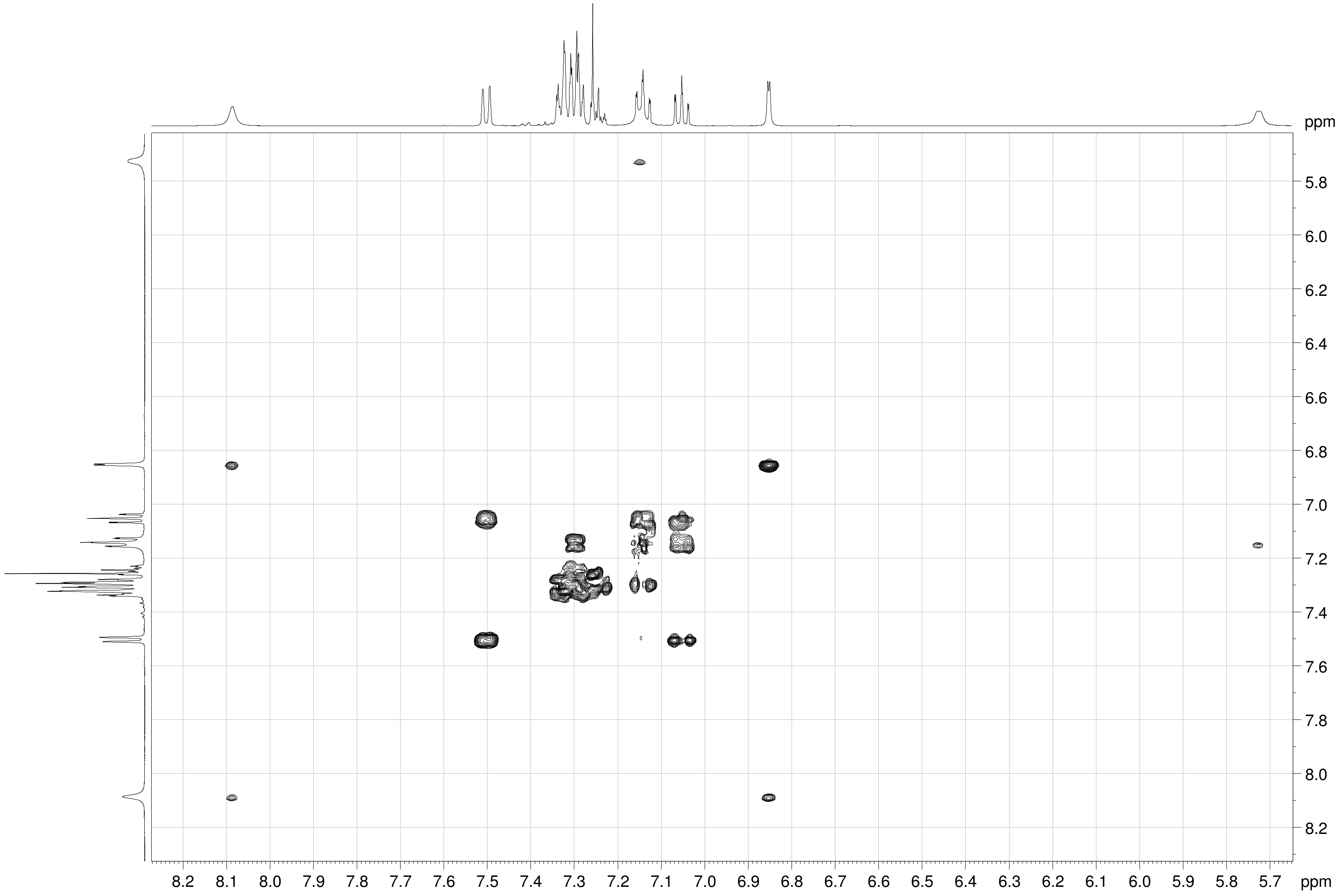

3539-COSY
B-amid

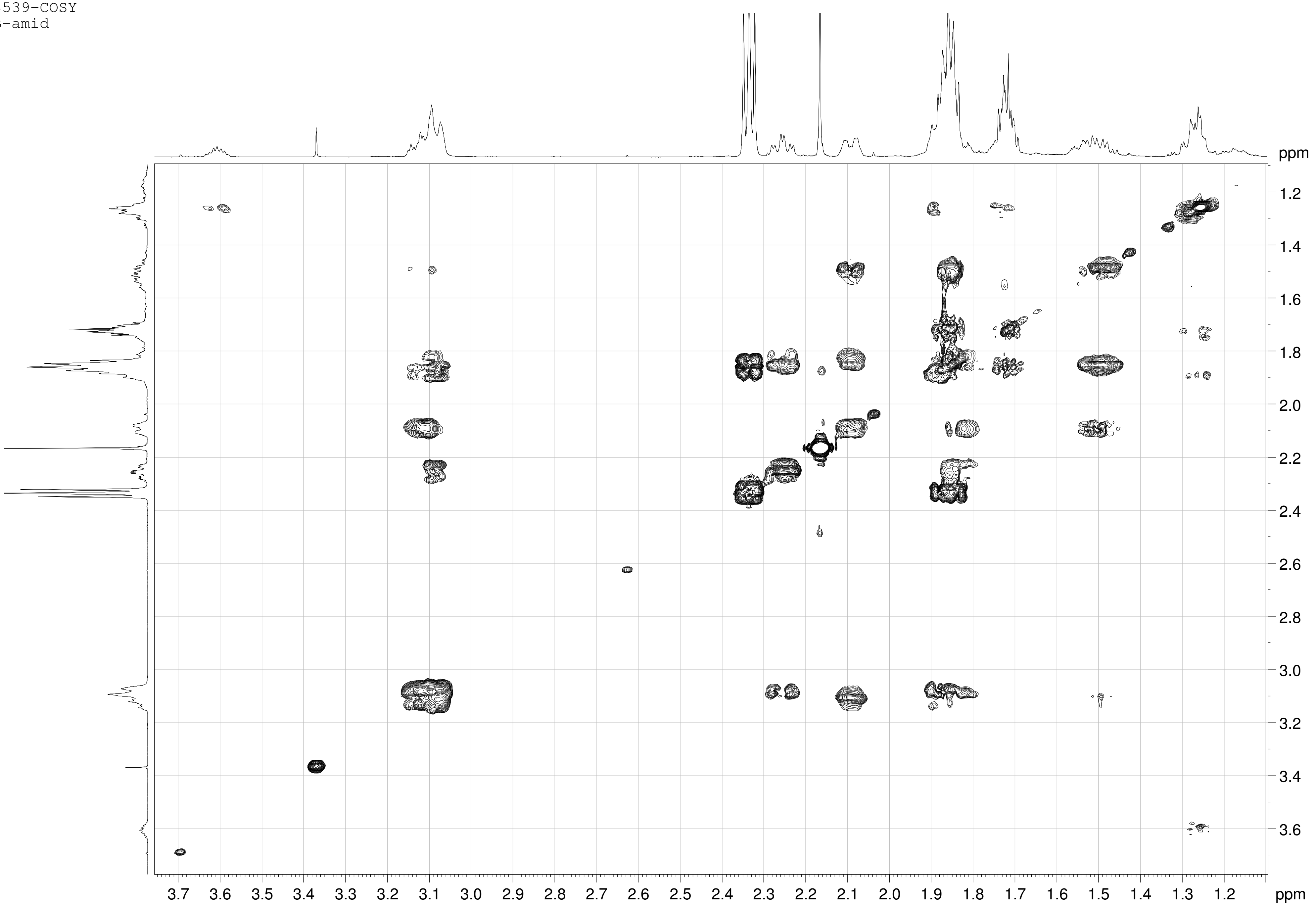

3539-HSQC
B-amid

Current Data Parameters
NAME 3539
EXPNO 3
PROCNO 1

F2 - Acquisition Parameters
Date_ 20170217
PROBHD 5 mm PABBI 1H/
PULPROG hsqcedetgpsisp2.3
SOLVENT CDCl3
TE 298.6 K
SWH 5151.099 Hz
AQ 0.1987925 sec
NS 2
FIDRES 2.515185 Hz
D0 0.00000300 sec
D1 2.00000000 sec
D4 0.00172414 sec
D11 0.03000000 sec
D16 0.00020000 sec
D21 0.00360000 sec
D24 0.00089000 sec

======== CHANNEL f1 ========
SFO1 500.2022151 MHz
NUC1 1H
P1 8.00 usec
P2 16.00 usec
P28 20.00 usec
PLW1 12.69999981 W

======== CHANNEL f2 ========
SFO2 125.7880080 MHz
NUC2 13C
P3 15.00 usec
P14 500.00 usec
P24 2000.00 usec
P31 1900.00 usec
PLW0 0 W
PLW2 78.52400208 W
PLW12 3.60570002 W

====== GRADIENT CHANNEL =====
P16 1000.00 usec
P19 600.00 usec

F1 - Acquisition parameters
TD 128
SFO1 125.788 MHz
FIDRES 200.320511 Hz
SW 203.843 ppm
FnMODE Echo-Antiecho

F2 - Processing parameters

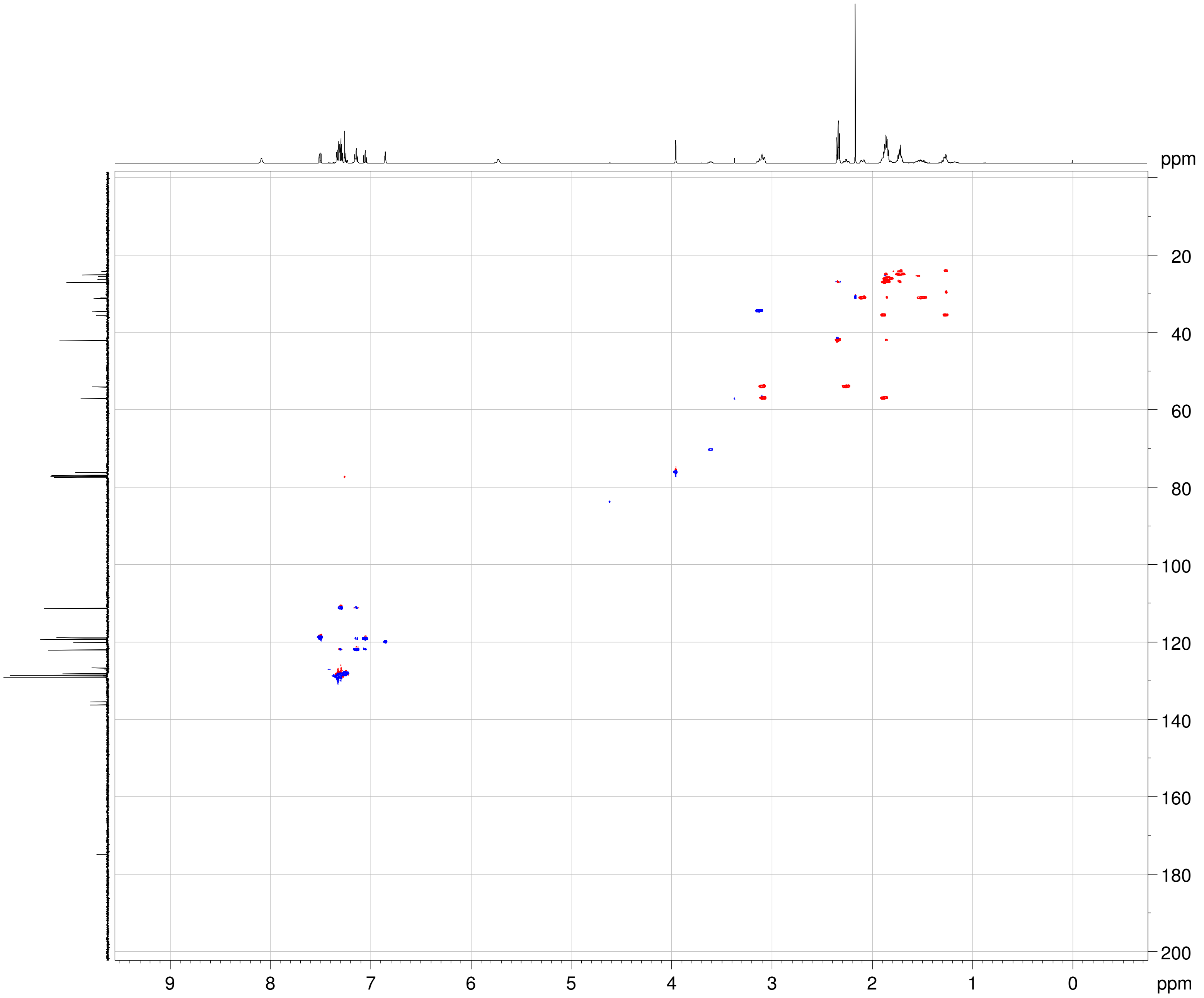

3539-HSQC
B-amid

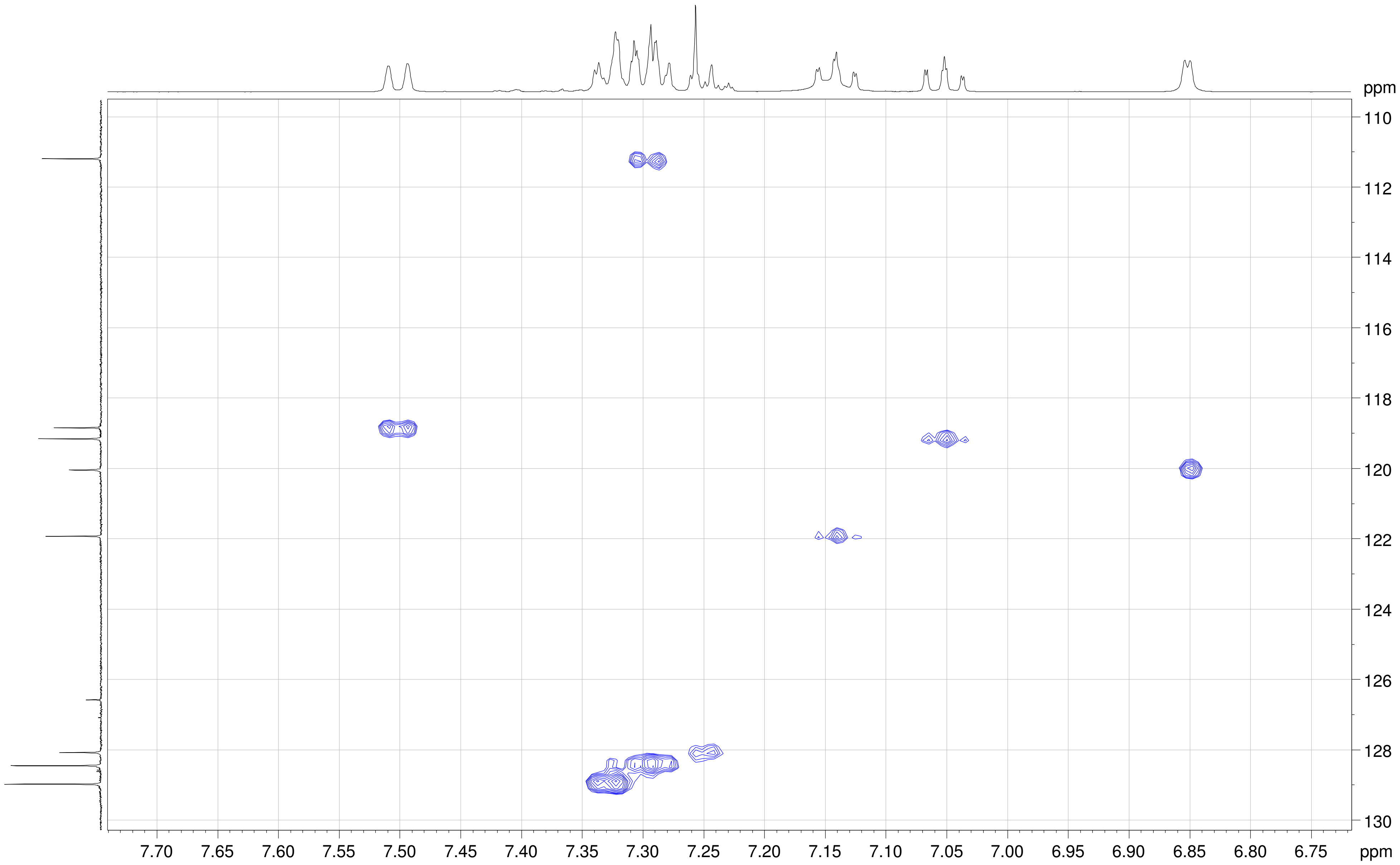

3539-HSQC
B-amid

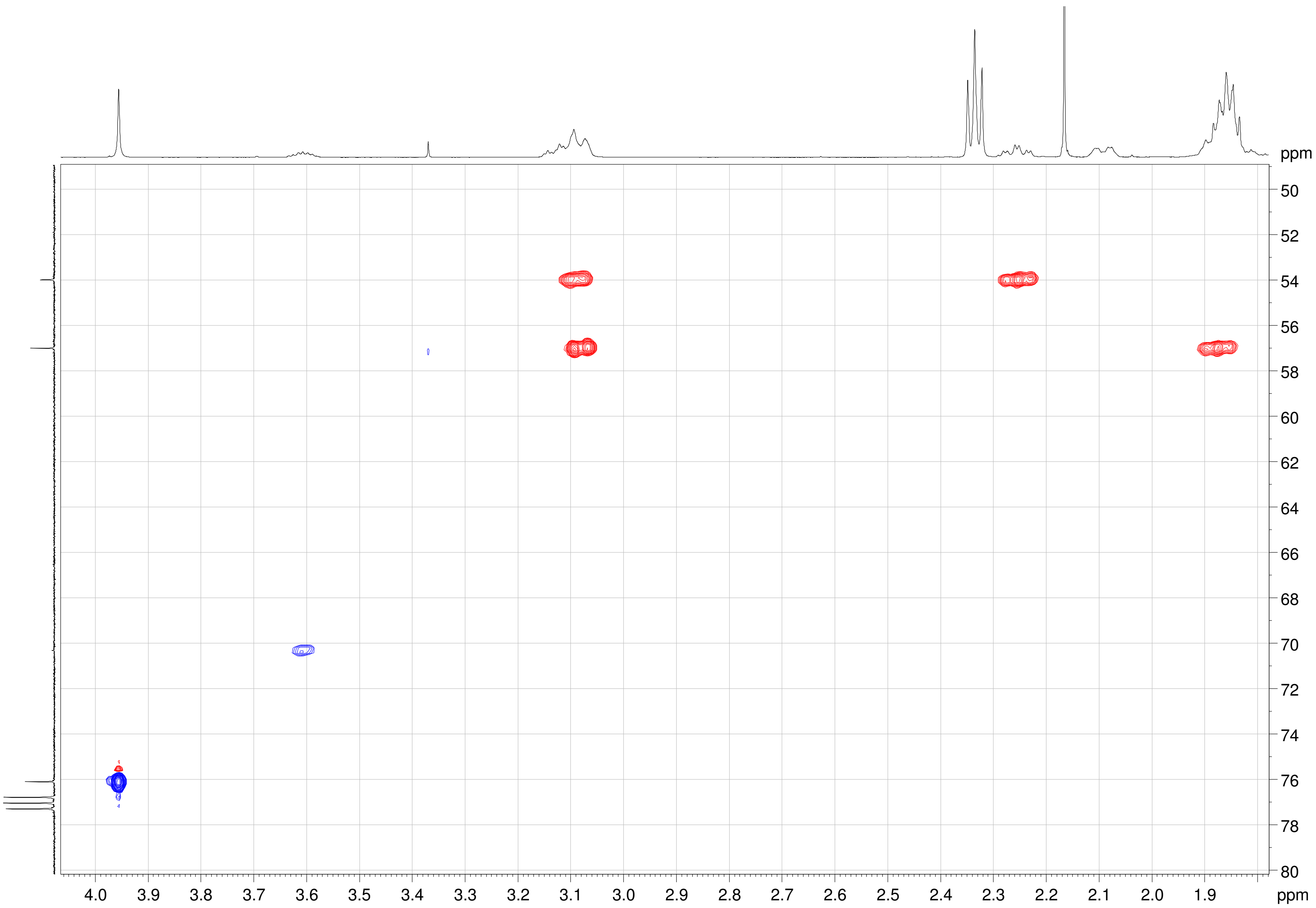

3539-HSQC
B-amid

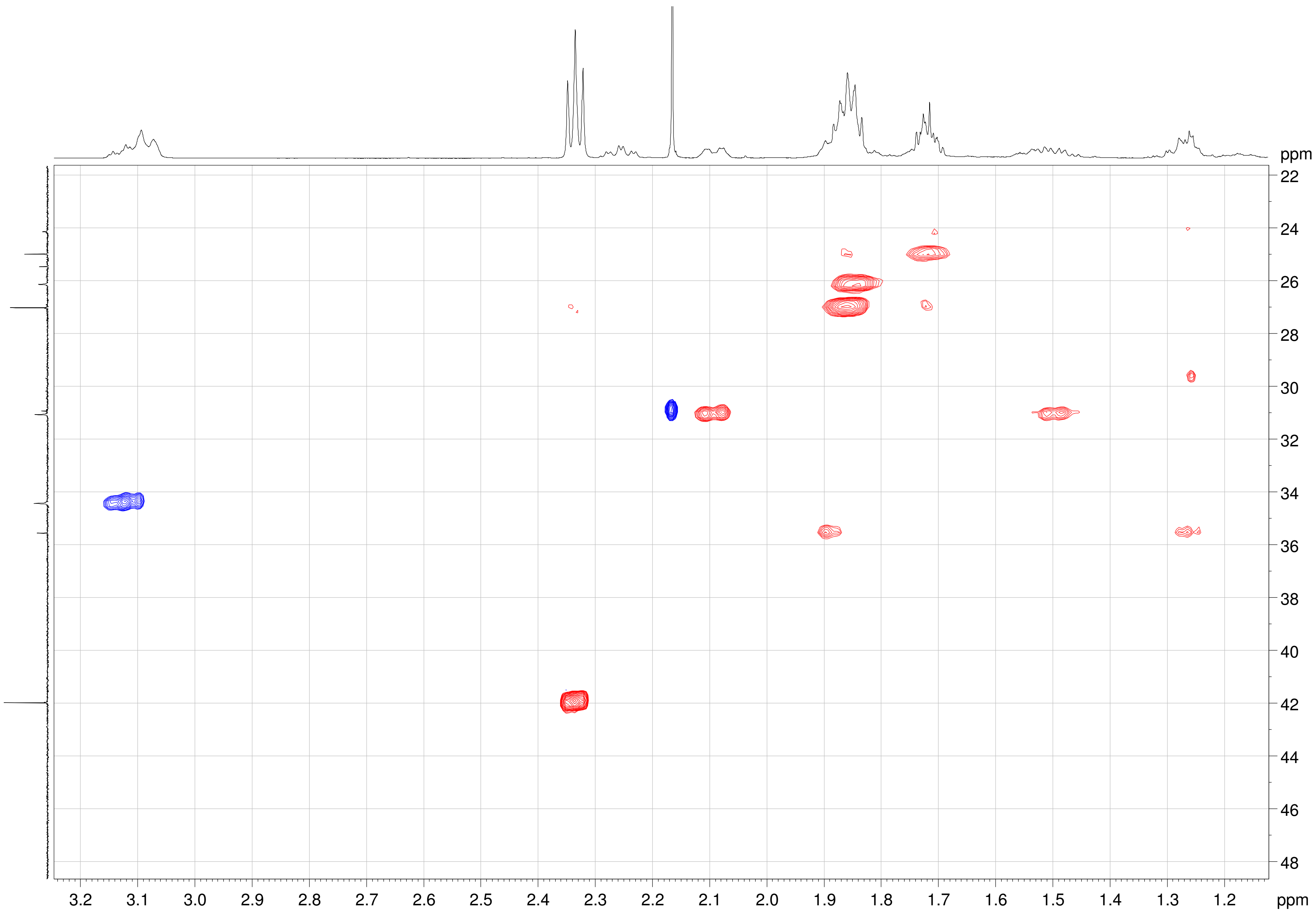

(3R,2R)-6b

4041-1H
C

```
Current Data Parameters
NAME                4041
EXPNO                  1
PROCNO                 1

F2 - Acquisition Parameters
Date_           20170811
PROBHD   5 mm PABBI 1H/
PULPROG             zg30
SOLVENT            CDCl3
TE                 298.2 K
SWH            10000.000 Hz
AQ             3.2767999 sec
NS                     8
FIDRES          0.152588 Hz
D1       0 sec

======== CHANNEL f1 =======
SFO1        500.2030889 MHz
NUC1                  1H
P1                  8.00 use
PLW1         12.69999981 W

F2 - Processing parameters
SI                 65536
LB       0 Hz
```

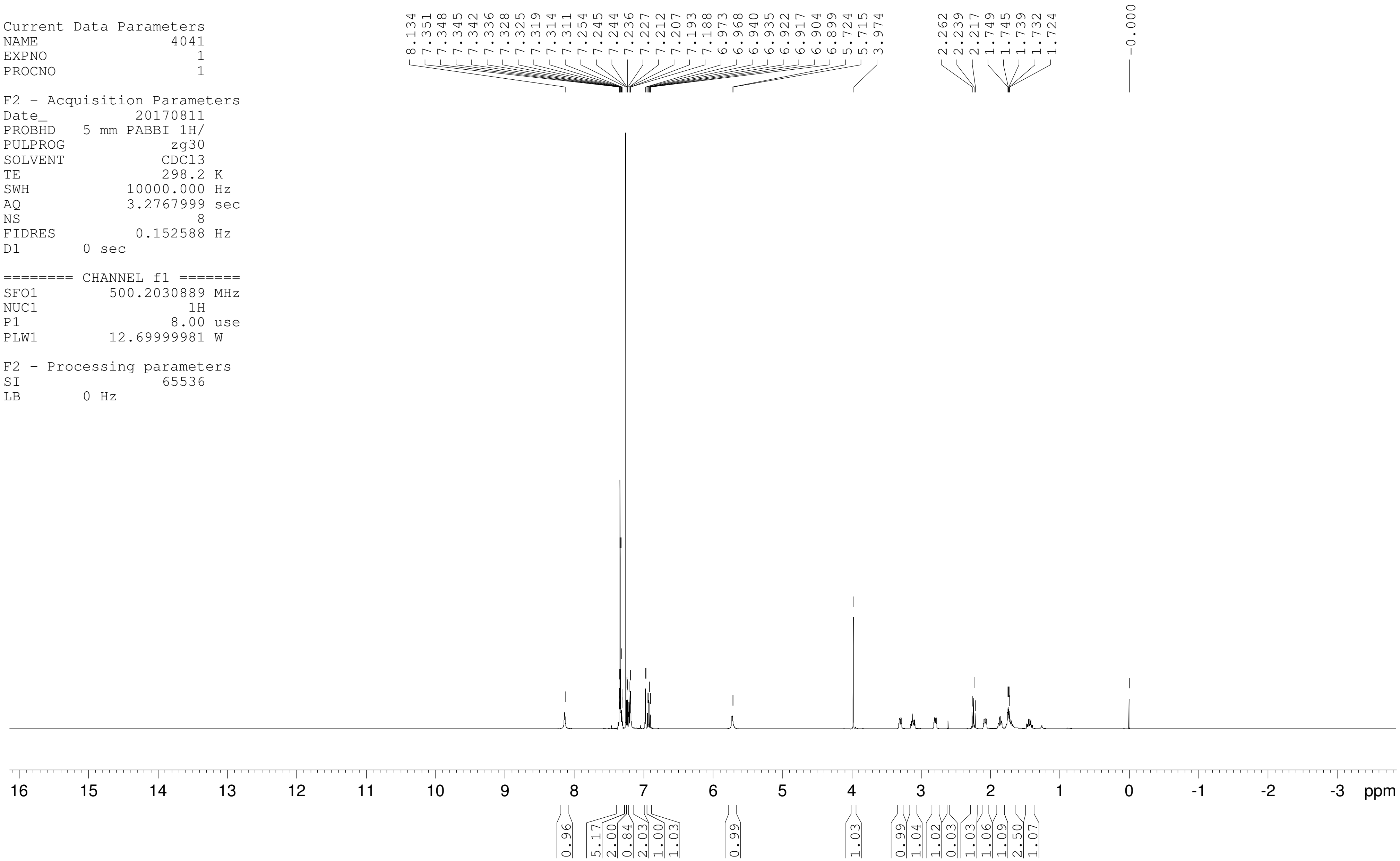

4041-1H
C

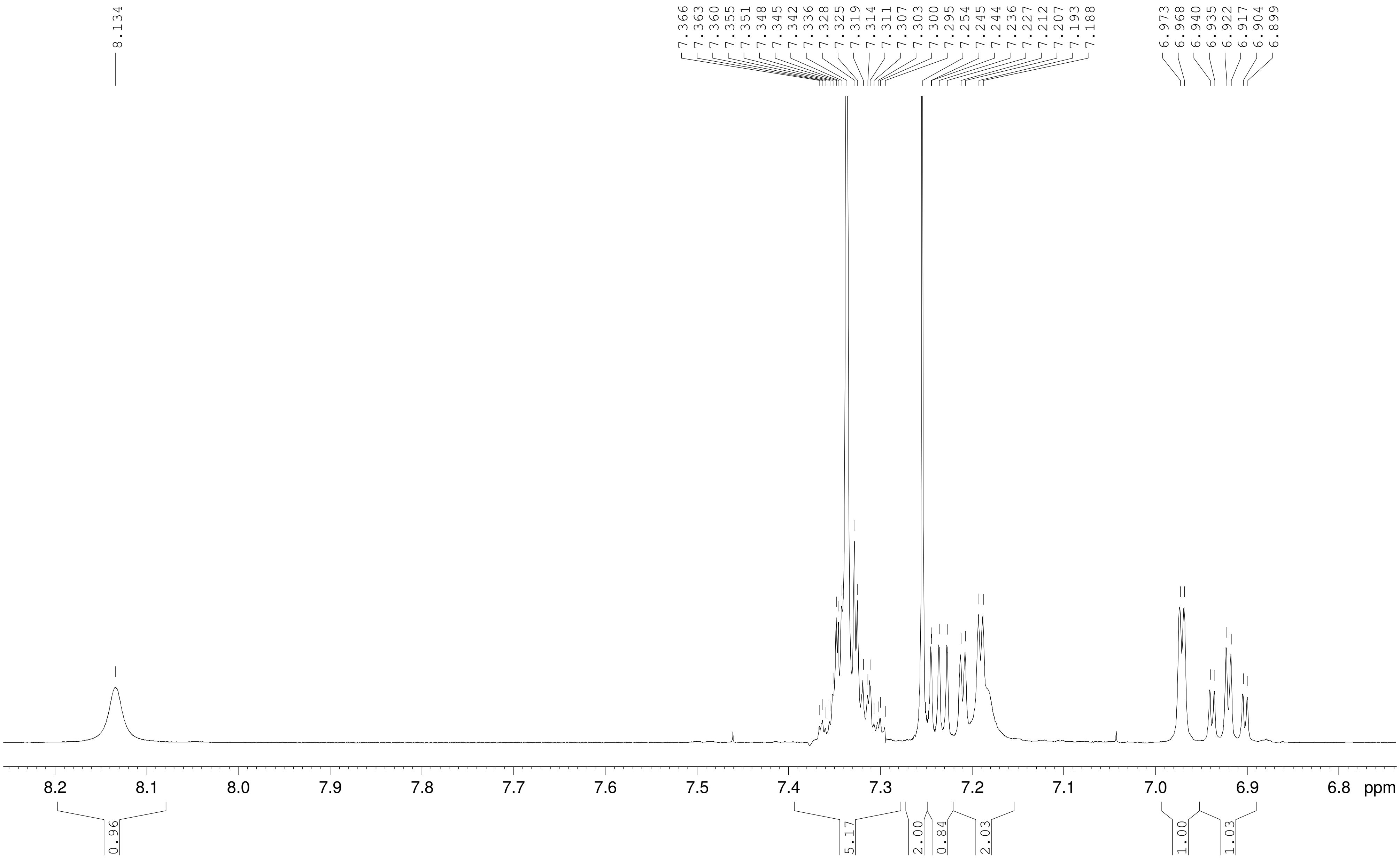

4041-1H
C

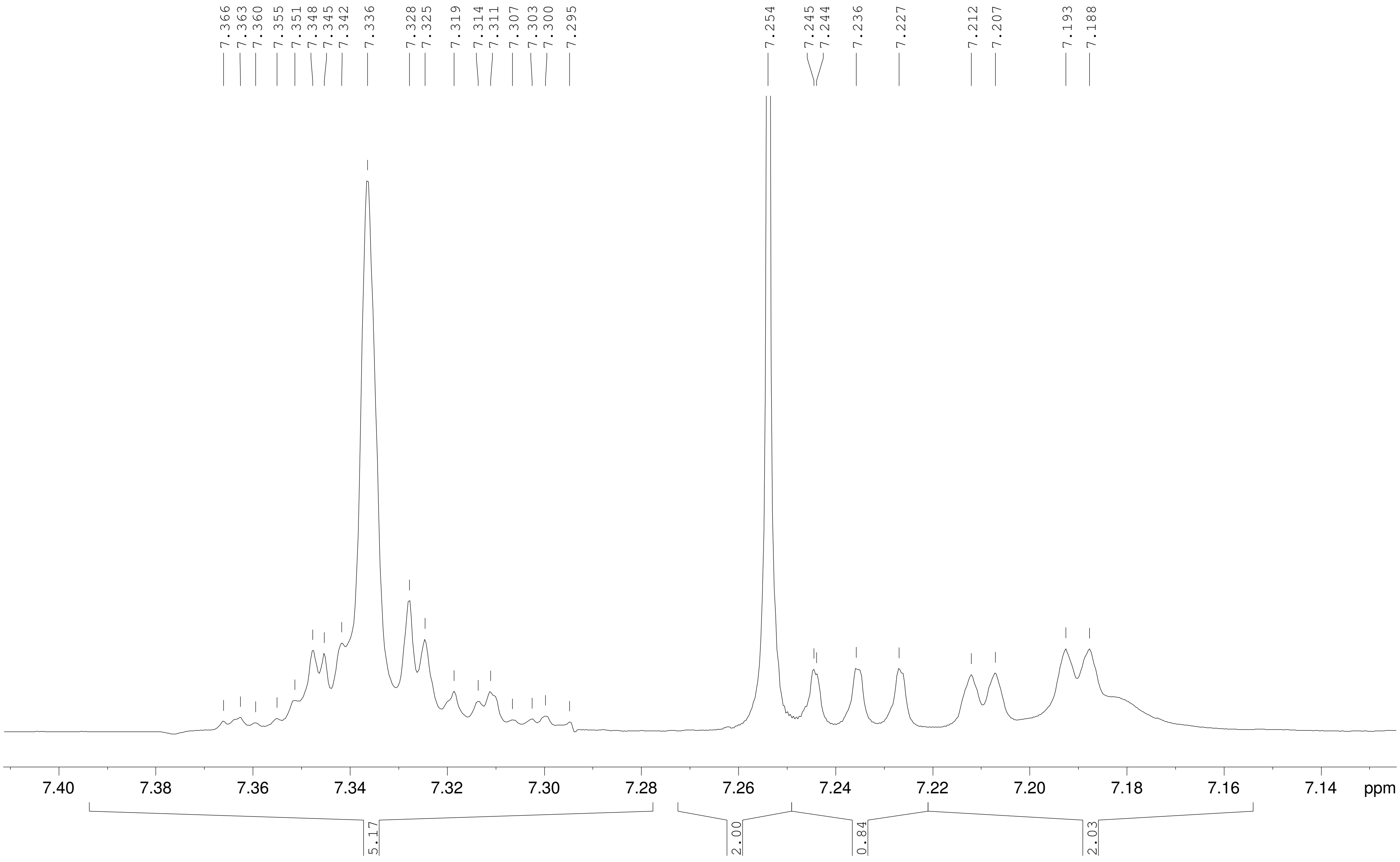

7.366
7.363
7.360
7.355
7.351
7.348
7.345
7.342
7.336
7.328
7.325
7.319
7.314
7.311
7.307
7.303
7.300
7.295
7.254
7.245
7.244
7.236
7.227
7.212
7.207
7.193
7.188
7.40
7.38
7.36
7.34
7.32
7.30
7.28
7.26
7.24
7.22
7.20
7.18
7.16
7.14
ppm
5.17
2.00
0.84
2.03

4041-1H
C

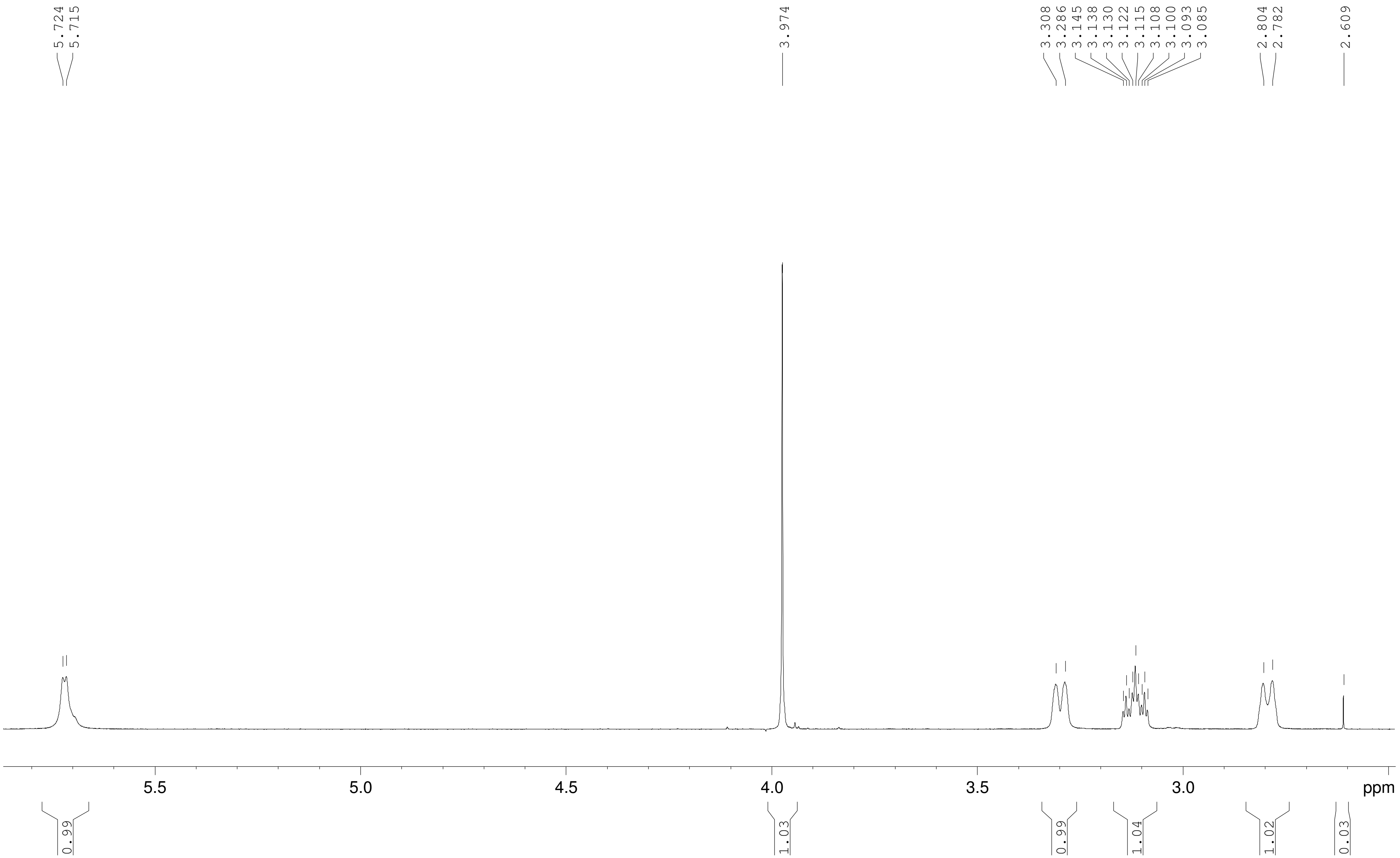
5.724
5.715
3.974
3.308
3.286
3.145
3.138
3.130
3.122
3.115
3.108
3.100
3.093
3.085
2.804
2.782
2.609
5.5
5.0
4.5
4.0
3.5
3.0
ppm
0.99
1.03
0.99
1.04
1.02
0.03

4041-1H
C

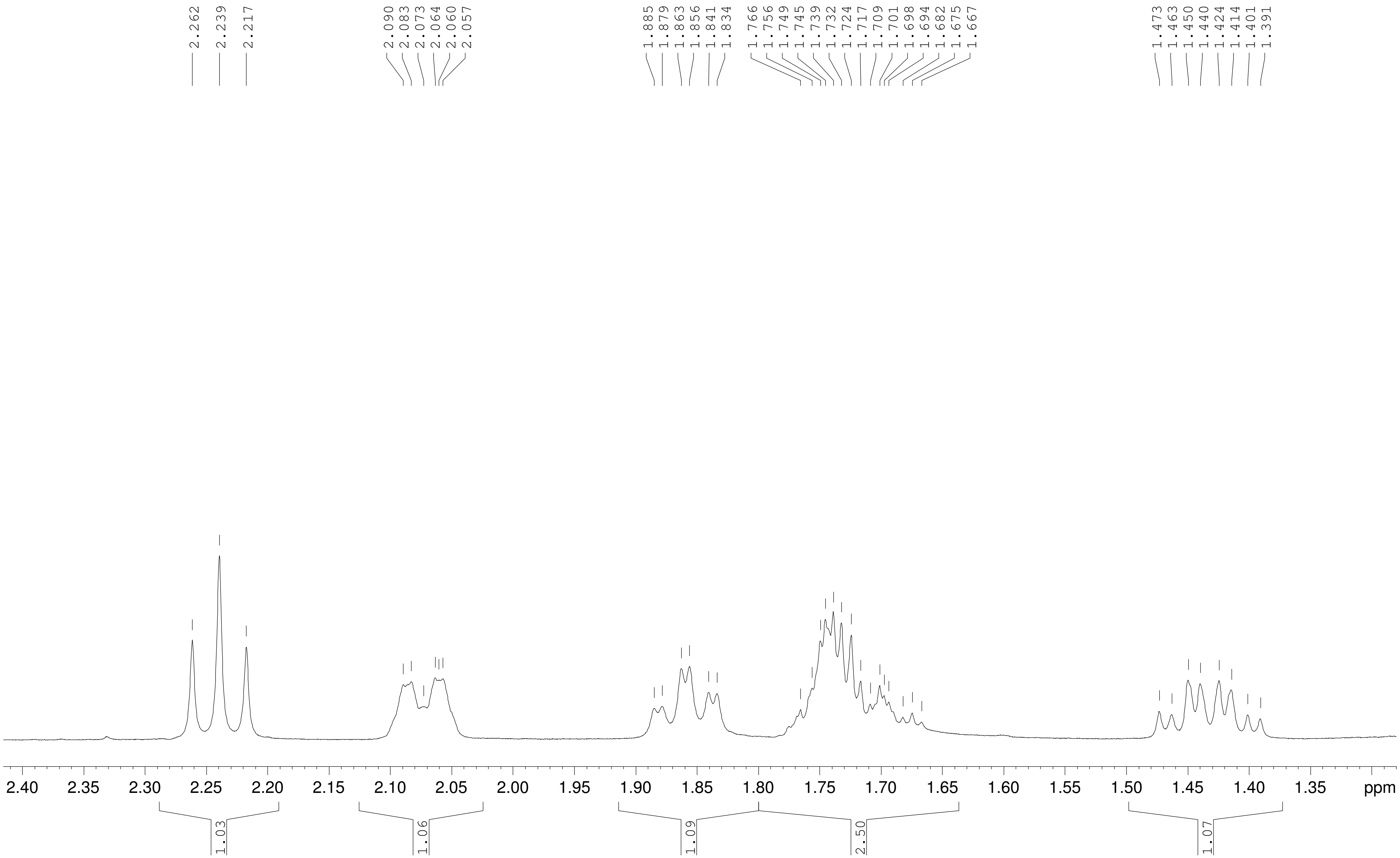

2.262
2.239
2.217
2.090
2.083
2.073
2.064
2.060
2.057
1.885
1.879
1.863
1.856
1.841
1.834
1.766
1.756
1.749
1.745
1.739
1.732
1.724
1.717
1.709
1.701
1.698
1.694
1.682
1.675
1.667
1.473
1.463
1.450
1.440
1.424
1.414
1.401
1.391
2.40
2.35
2.30
2.25
2.20
2.15
2.10
2.05
2.00
1.95
1.90
1.85
1.80
1.75
1.70
1.65
1.60
1.55
1.50
1.45
1.40
1.35
ppm
1.03
1.06
1.09
2.50
1.07

4041-13C
C

Current Data Parameters
NAME 4041
EXPNO 2
PROCNO 1

F2 - Acquisition Parameters
Date_ 20170811
PROBHD 5 mm PABBI 1H/
PULPROG zg_pi_CPD
SOLVENT CDCl3
TE 298.2 K
SWH 31249.381 Hz
AQ 1.0485969 sec
NS 1012
FIDRES 0.476828 Hz
D1 1.50000000 sec
D11 0.03000000 sec
D14 0.00050000 sec

======== CHANNEL f1 ========
SFO1 125.7904831 MHz
NUC1 13C
P1 15.00 usec
P2 30.00 usec
PLW1 78.52400208 W

======== CHANNEL f2 ========
SFO2 500.2020008 MHz
NUC2 1H
PLW2 12.50000000 W
PLW12 0.12500000 W

F2 - Processing parameters
SI 65536
LB 0.50 Hz

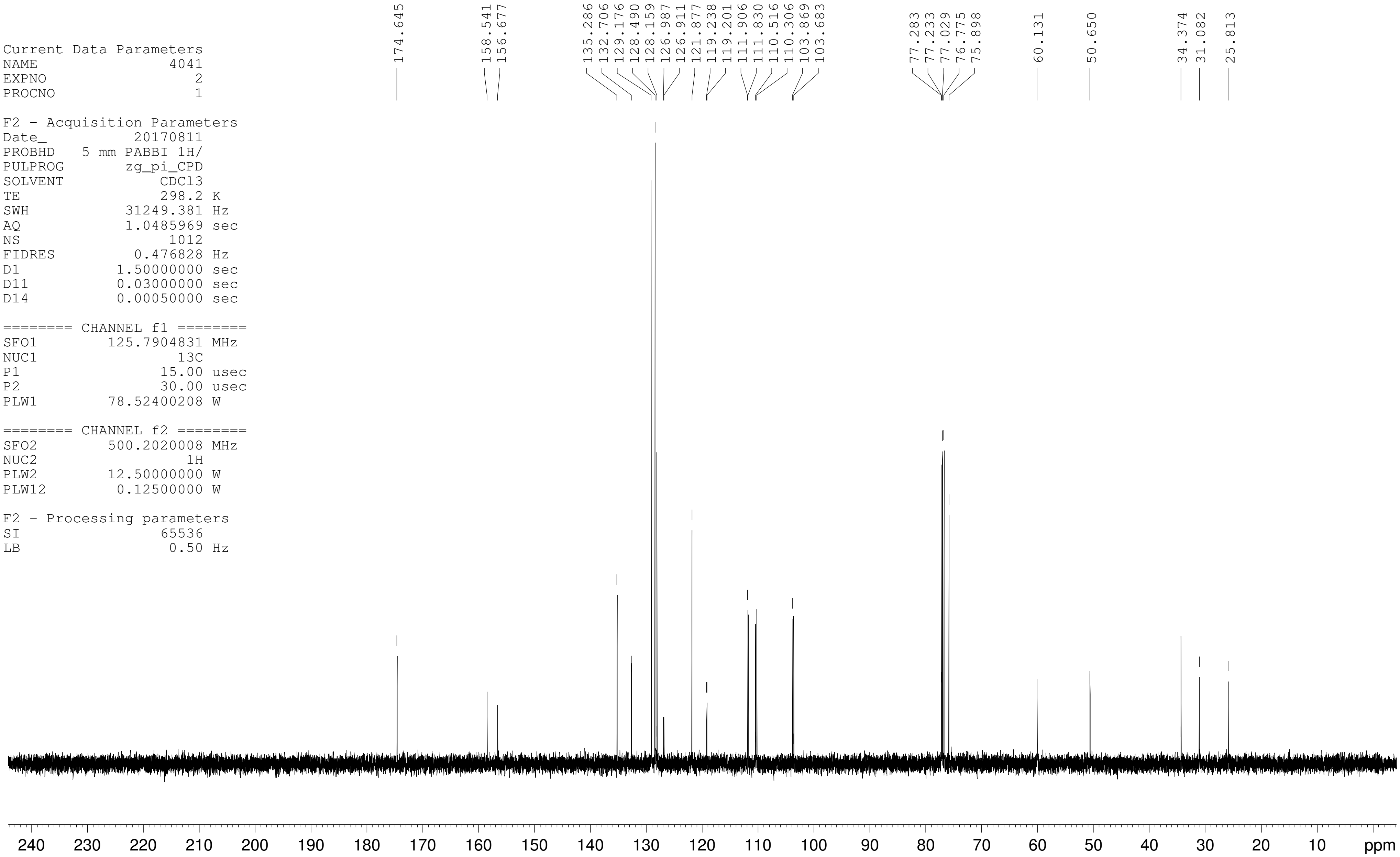

4041-13C
C

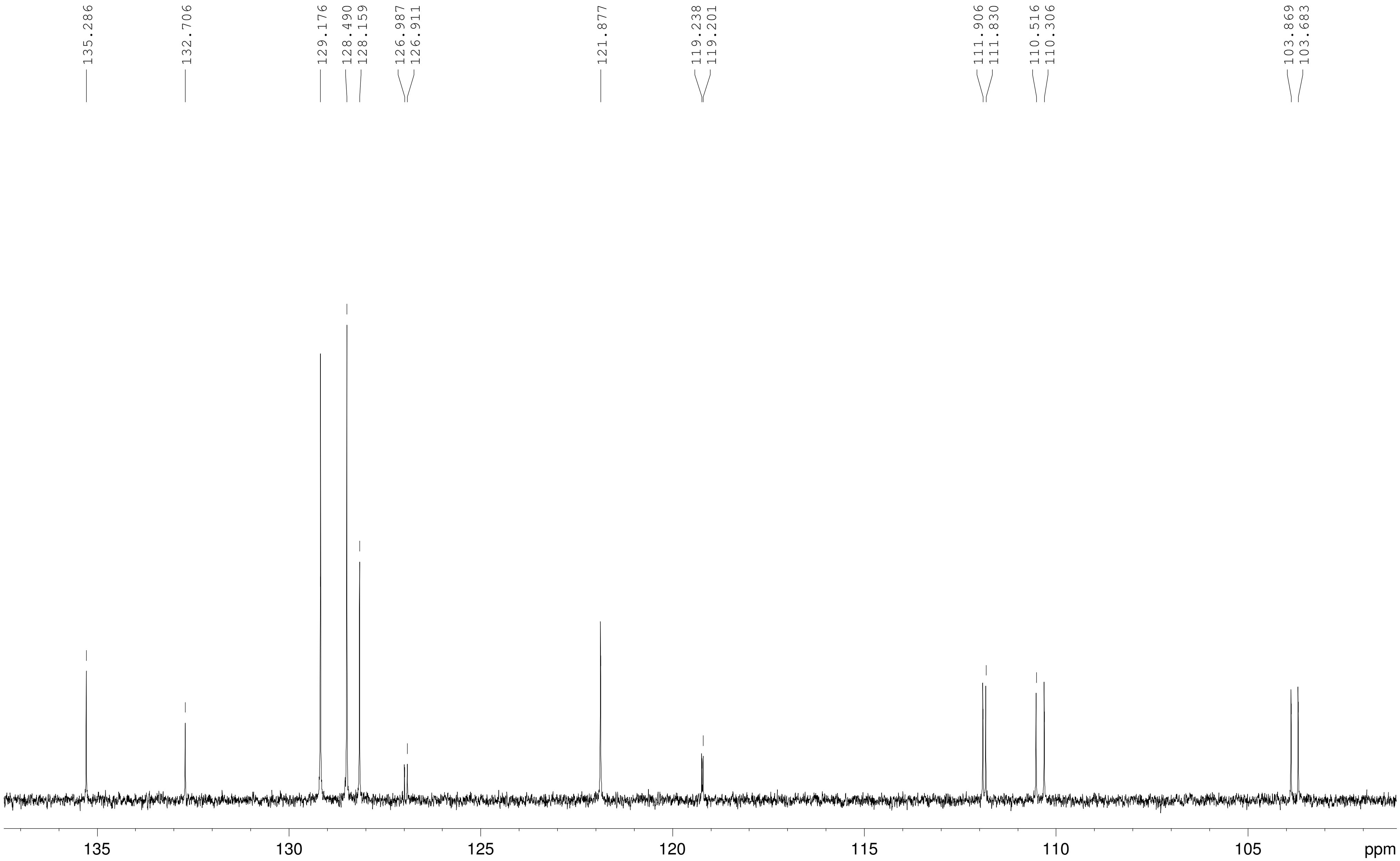

135.286
132.706
129.176
128.490
128.159
126.987
126.911
121.877
119.238
119.201
111.906
111.830
110.516
110.306
103.869
103.683
135
130
125
120
115
110
105
ppm

4041-13C
C

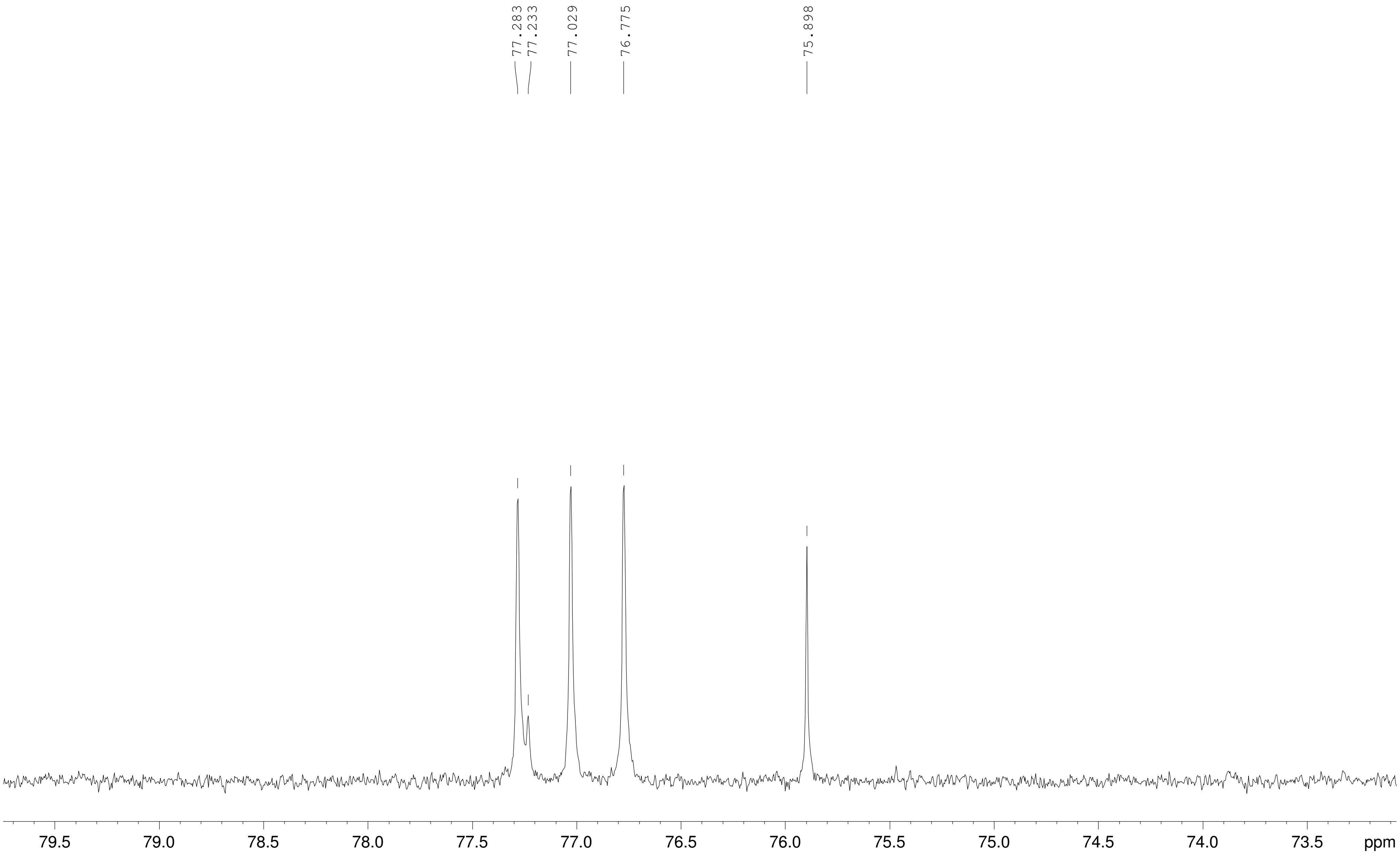
77.283
77.233
77.029
76.775
75.898
79.5
79.0
78.5
78.0
77.5
77.0
76.5
76.0
75.5
75.0
74.5
74.0
73.5
ppm

(3S,2R)-7b

4042-1H
D

Current Data Parameters
NAME 4042
EXPNO 1
PROCNO 1

F2 - Acquisition Parameters
Date_ 20170811
PROBHD 5 mm PABBI 1H/
PULPROG zg30
SOLVENT CDCl3
TE 298.1 K
SWH 10000.000 Hz
AQ 3.2767999 sec
NS 8
FIDRES 0.152588 Hz
D1 0 sec

======== CHANNEL f1 =======
SFO1 500.2030889 MHz
NUC1 1H
P1 8.00 use
PLW1 12.69999981 W

F2 - Processing parameters
SI 65536
LB 0 Hz

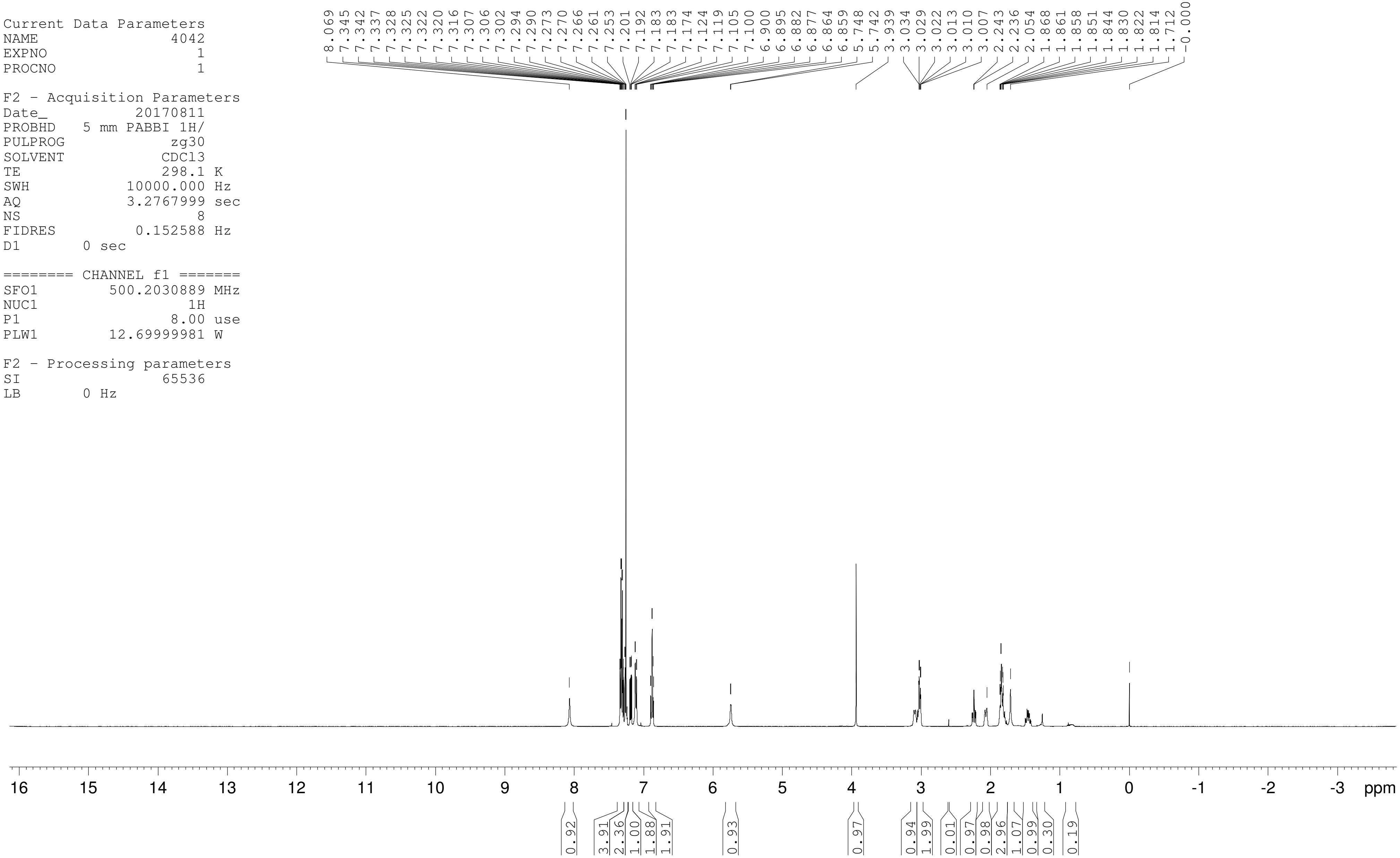

4042-1H
D

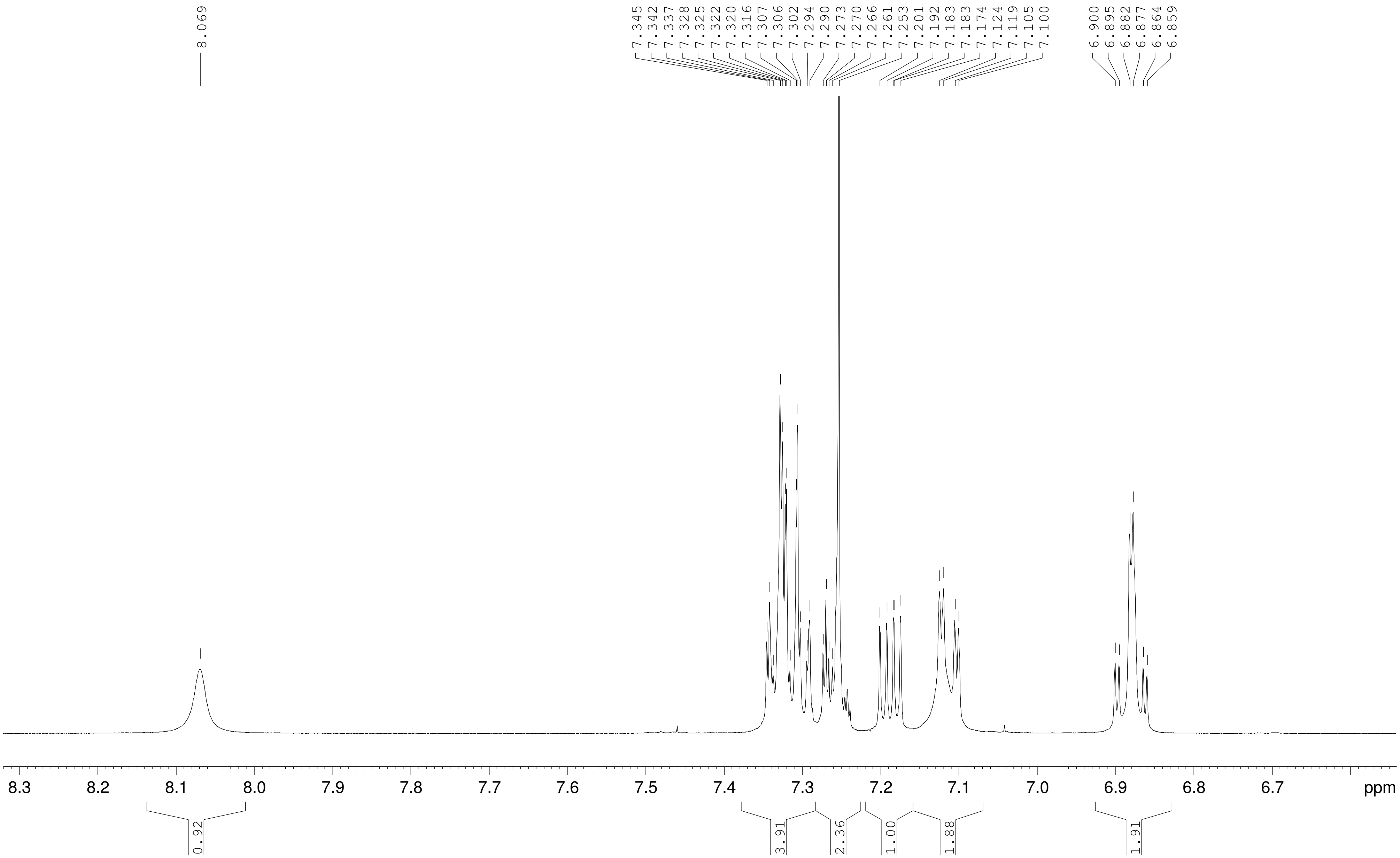
8.069
7.345
7.342
7.337
7.328
7.325
7.322
7.320
7.316
7.307
7.306
7.302
7.294
7.290
7.273
7.270
7.266
7.261
7.253
7.201
7.192
7.183
7.183
7.174
7.124
7.119
7.105
7.100
6.900
6.895
6.882
6.877
6.864
6.859
8.3
8.2
8.1
8.0
7.9
7.8
7.7
7.6
7.5
7.4
7.3
7.2
7.1
7.0
6.9
6.8
6.7
ppm
0.92
3.91
2.36
1.00
1.88
1.91

4042-1H
D

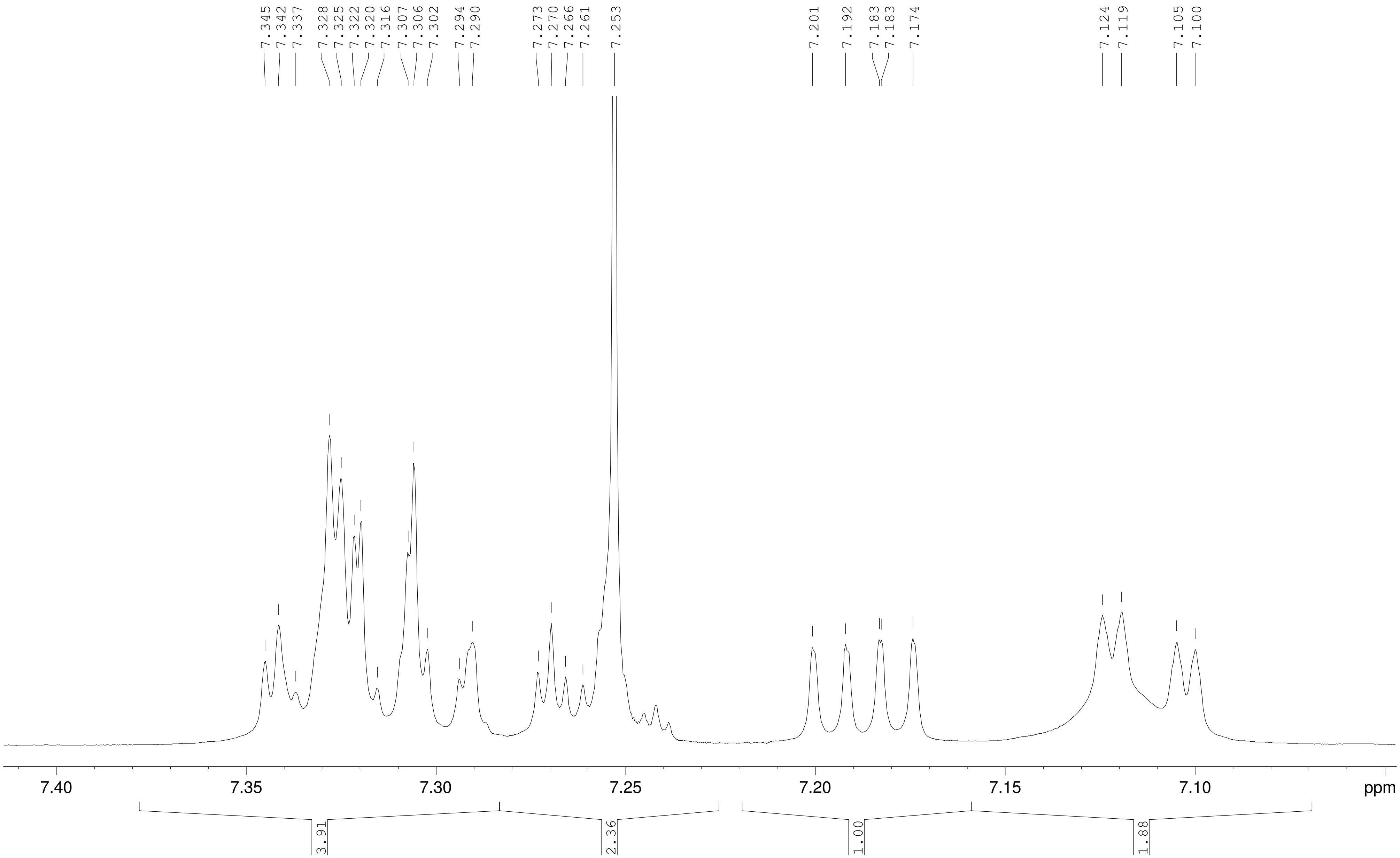

7.345
7.342
7.337
7.328
7.325
7.322
7.320
7.316
7.307
7.306
7.302
7.294
7.290
7.273
7.270
7.266
7.261
7.253
7.201
7.192
7.183
7.183
7.174
7.124
7.119
7.105
7.100
7.40
7.35
7.30
7.25
7.20
7.15
7.10
ppm
3.91
2.36
1.00
1.88

4042-1H
D

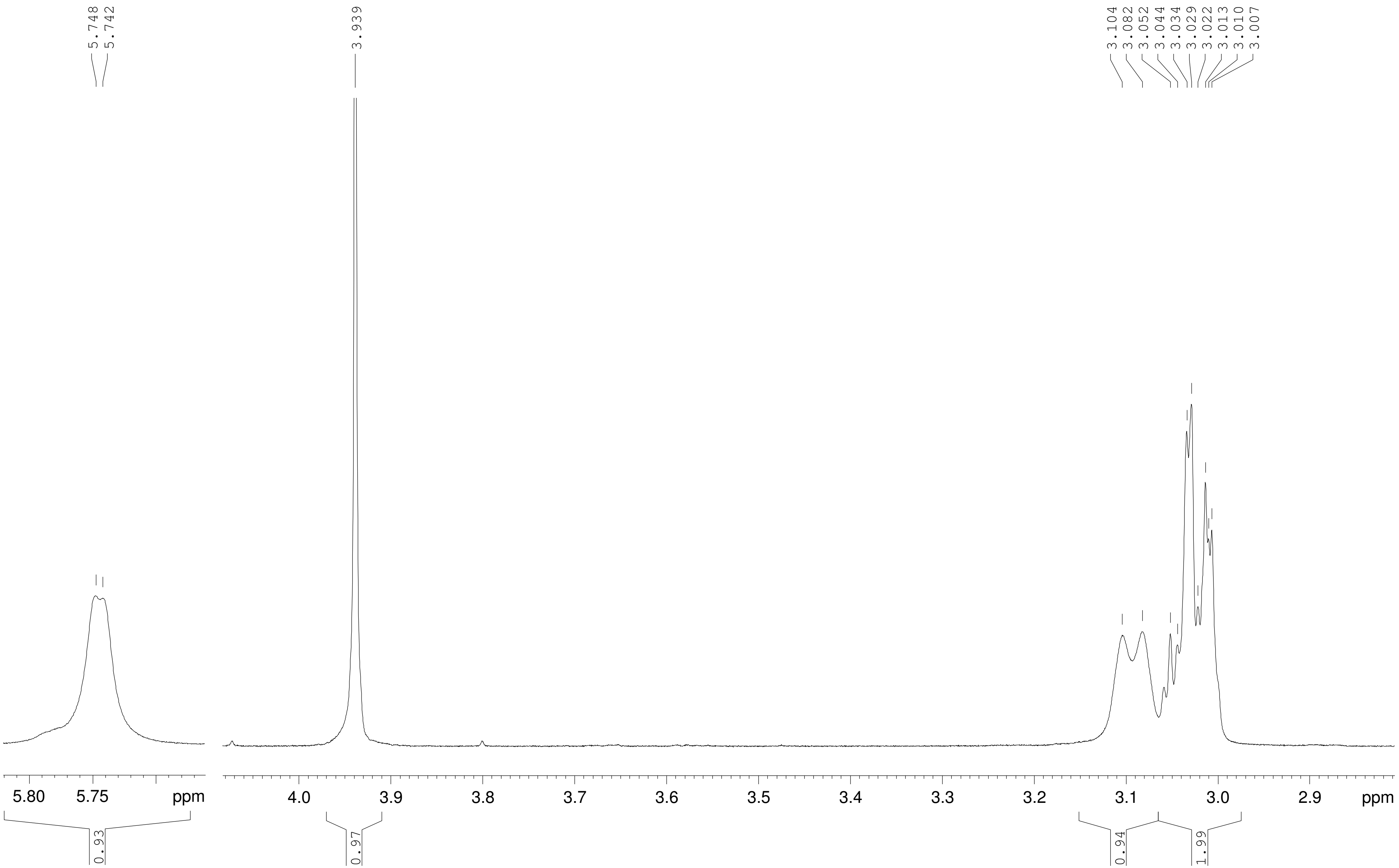
5.748
5.742
3.939
3.104
3.082
3.052
3.044
3.034
3.029
3.022
3.013
3.010
3.007
5.80
5.75
ppm
4.0
3.9
3.8
3.7
3.6
3.5
3.4
3.3
3.2
3.1
3.0
2.9
ppm
0.93
0.97
0.94
1.99

4042-1H
D

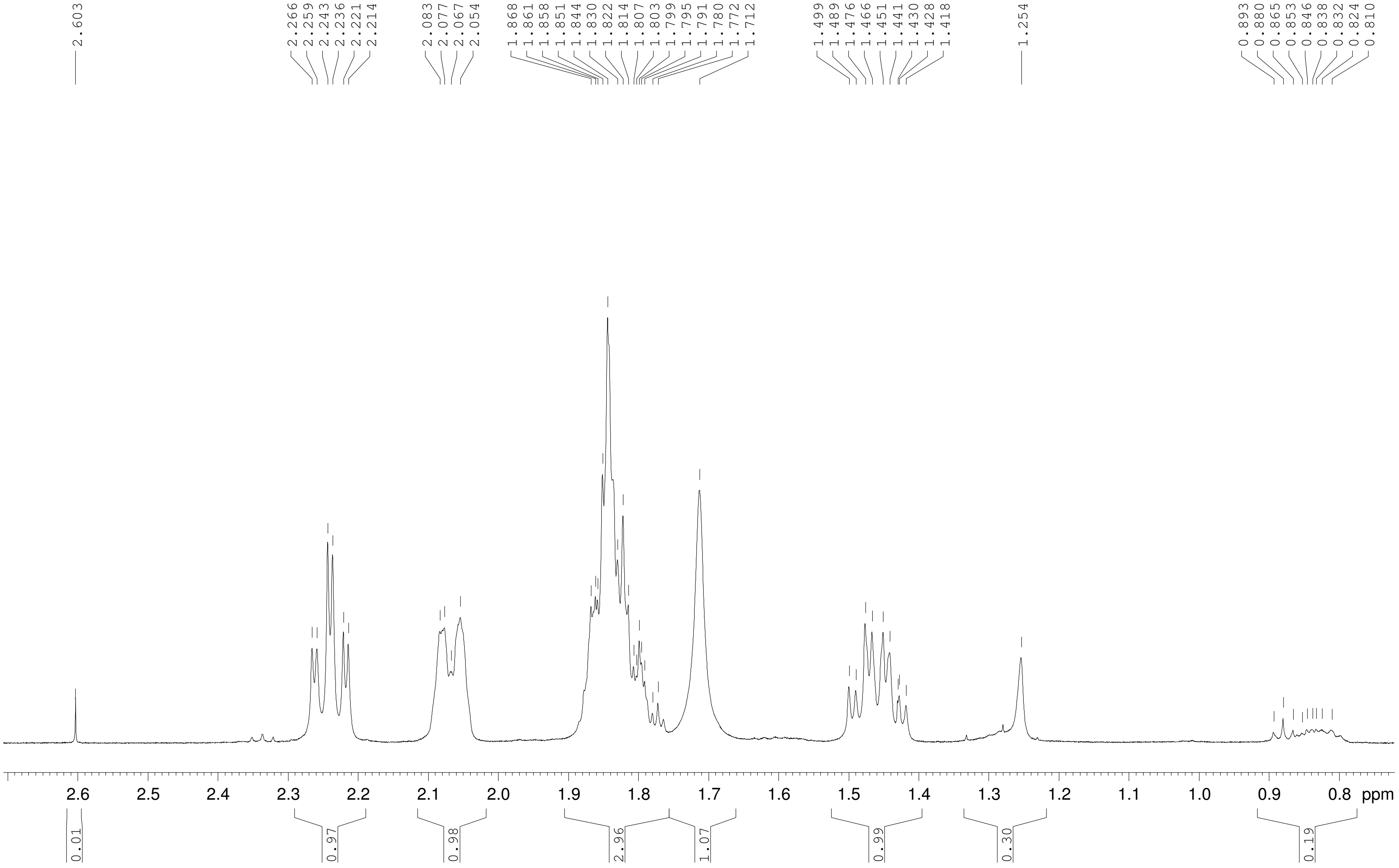
2.603
2.266 2.259 2.243 2.236 2.221 2.214
2.083 2.077 2.067 2.054
1.868 1.861 1.858 1.851 1.844 1.830 1.822 1.814 1.807 1.803 1.799 1.795 1.791 1.780 1.772 1.712
1.499 1.489 1.476 1.466 1.451 1.441 1.430 1.428 1.418
1.254
0.893 0.880 0.865 0.853 0.846 0.838 0.832 0.824 0.810
2.6 2.5 2.4 2.3 2.2 2.1 2.0 1.9 1.8 1.7 1.6 1.5 1.4 1.3 1.2 1.1 1.0 0.9 0.8 ppm
0.01 0.97 0.98 2.96 1.07 0.99 0.30 0.19

4042-13C
D

```
Current Data Parameters
NAME              4042
EXPNO                2
PROCNO               1

F2 - Acquisition Parameters
Date_          20170811
PROBHD   5 mm PABBI 1H/
PULPROG       zg_pi_CPD
SOLVENT           CDCl3
TE                298.2 K
SWH           31249.381 Hz
AQ            1.0485969 sec
NS                 1060
FIDRES         0.476828 Hz
D1           1.50000000 sec
D11          0.03000000 sec
D14          0.00050000 sec

======== CHANNEL f1 ========
SFO1        125.7904831 MHz
NUC1                13C
P1                15.00 usec
P2                30.00 usec
PLW1        78.52400208 W

======== CHANNEL f2 ========
SFO2        500.2020008 MHz
NUC2                 1H
PLW2        12.50000000 W
PLW12        0.12500000 W

F2 - Processing parameters
SI                65536
LB                 0.50 Hz
```

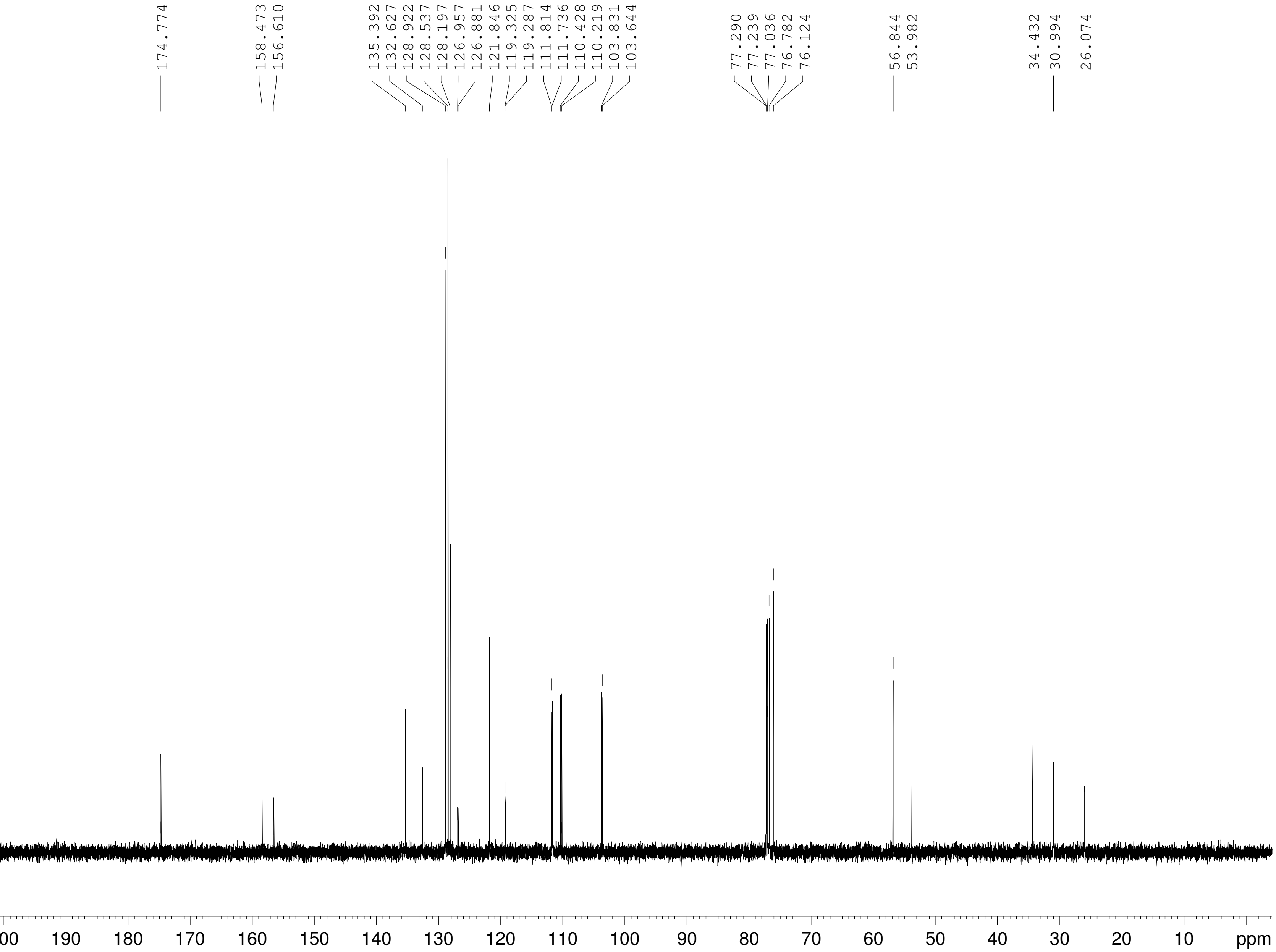

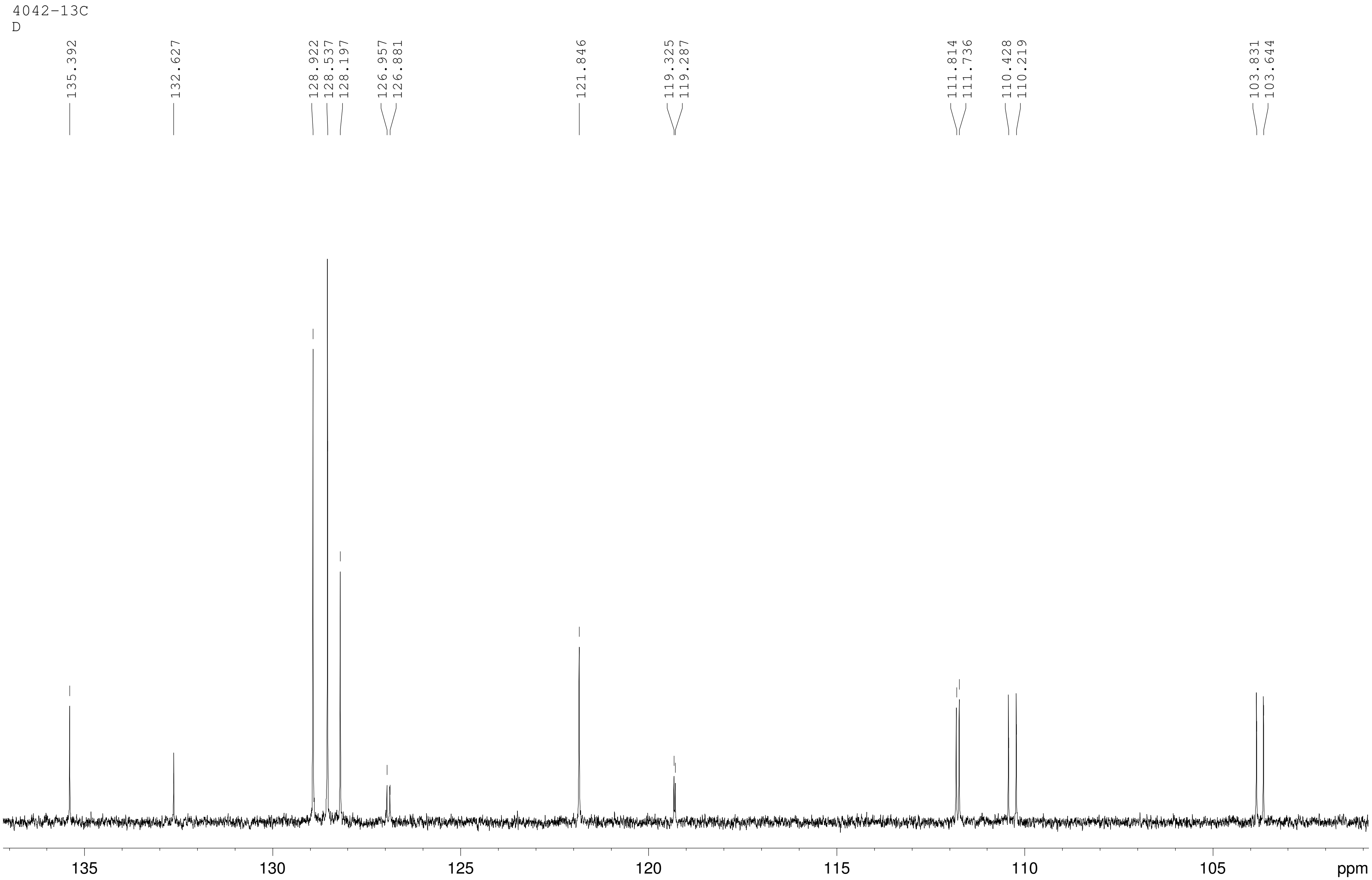

4042-13C
D
135.392
132.627
128.922
128.537
128.197
126.957
126.881
121.846
119.325
119.287
111.814
111.736
110.428
110.219
103.831
103.644
135
130
125
120
115
110
105
ppm

4042-13C
D

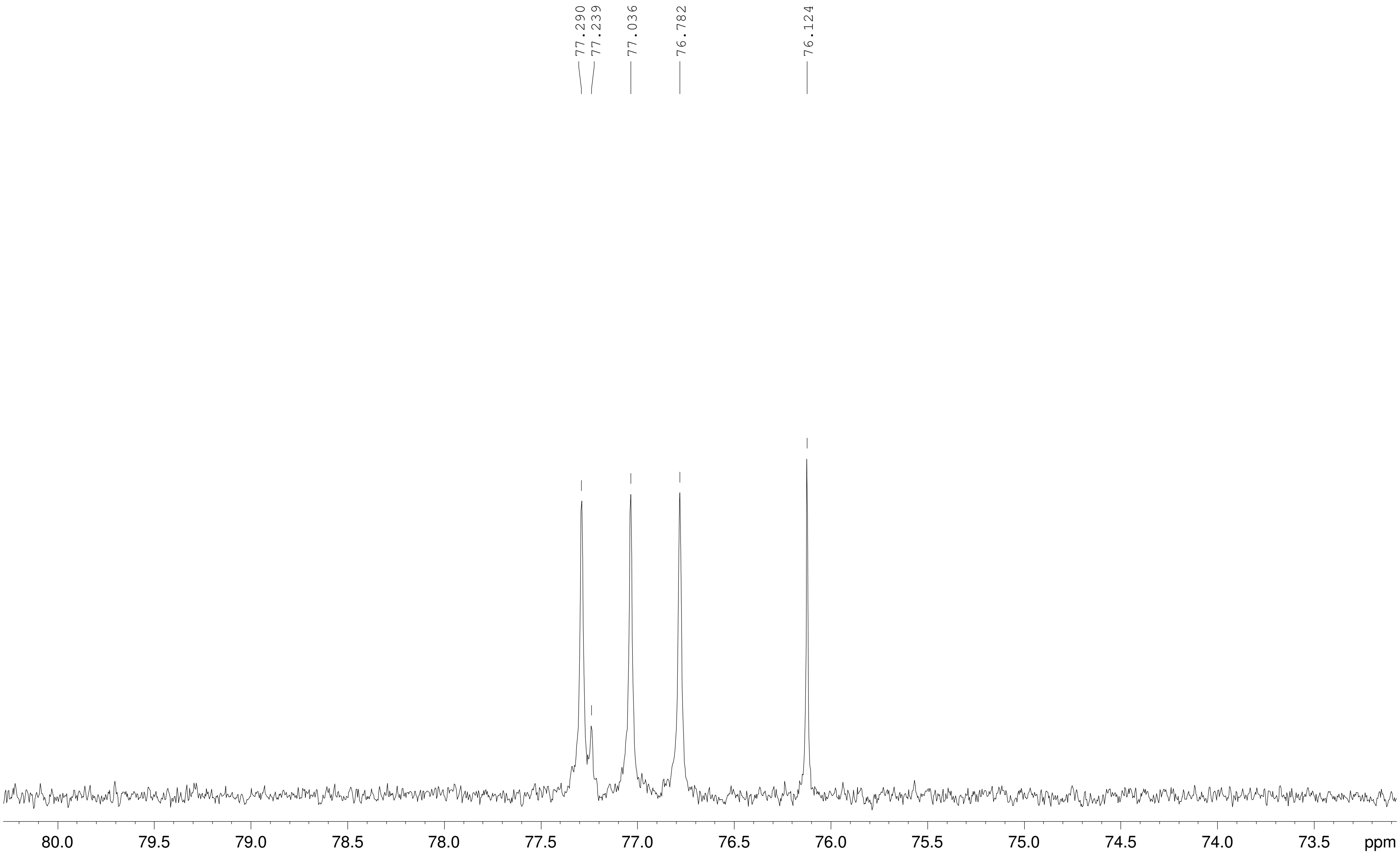
77.290
77.239
77.036
76.782
76.124
80.0
79.5
79.0
78.5
78.0
77.5
77.0
76.5
76.0
75.5
75.0
74.5
74.0
73.5
ppm

(3R,2R)-8c

4362-1H
E

```
Current Data Parameters
NAME              4362
EXPNO                1
PROCNO               1

F2 - Acquisition Parameters
Date_         20171129
PROBHD   5 mm CPPBBO BB
PULPROG           zg30
SOLVENT          CDCl3
TE               298.1 K
SWH          10000.000 Hz
AQ           3.2767999 sec
NS                   8
FIDRES        0.152588 Hz
D1       0 sec

======== CHANNEL f1 ========
SFO1       500.2030889 MHz
NUC1                1H
P1               11.30 usec
PLW1       13.69999981 W

F2 - Processing parameters
SI               65536
LB       0 Hz
```

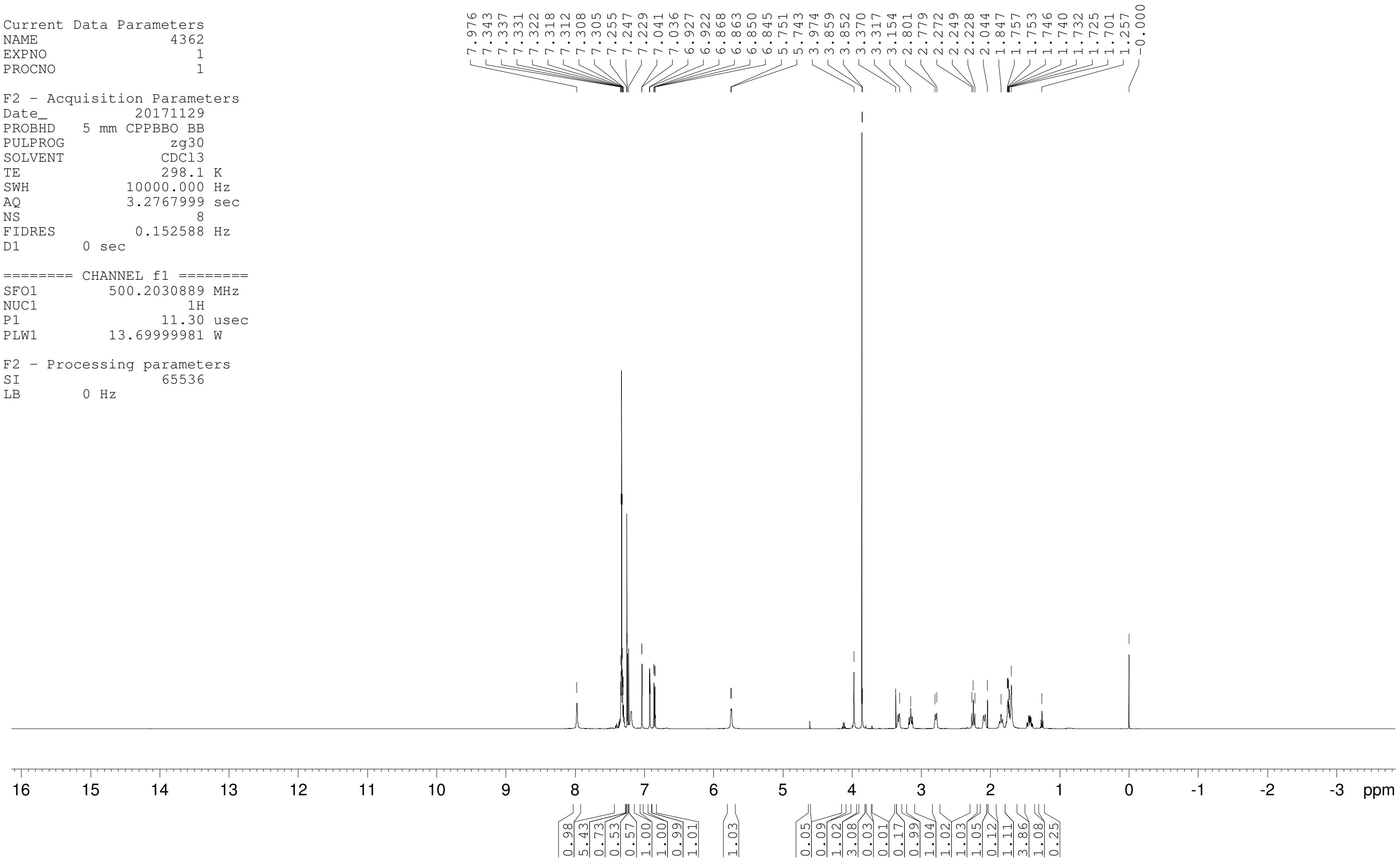

4362-1H
E

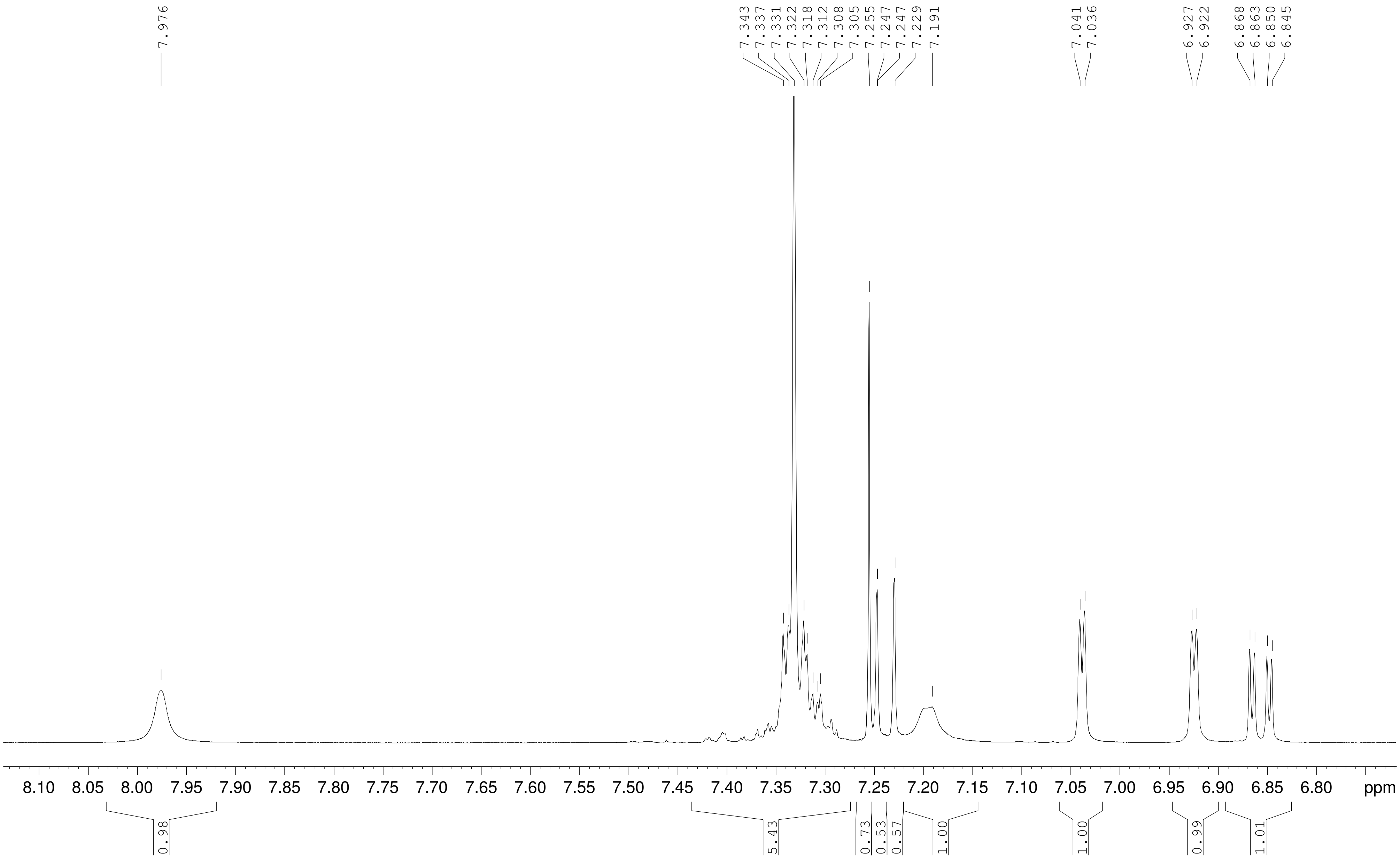

7.976
7.343 7.337 7.331 7.322 7.318 7.312 7.308 7.305 7.255 7.247 7.247 7.229 7.191
7.041 7.036
6.927 6.922
6.868 6.863 6.850 6.845
8.10 8.05 8.00 7.95 7.90 7.85 7.80 7.75 7.70 7.65 7.60 7.55 7.50 7.45 7.40 7.35 7.30 7.25 7.20 7.15 7.10 7.05 7.00 6.95 6.90 6.85 6.80 ppm
0.98
5.43
0.73 0.53 0.57
1.00
1.00
0.99
1.01

4362-1H
E

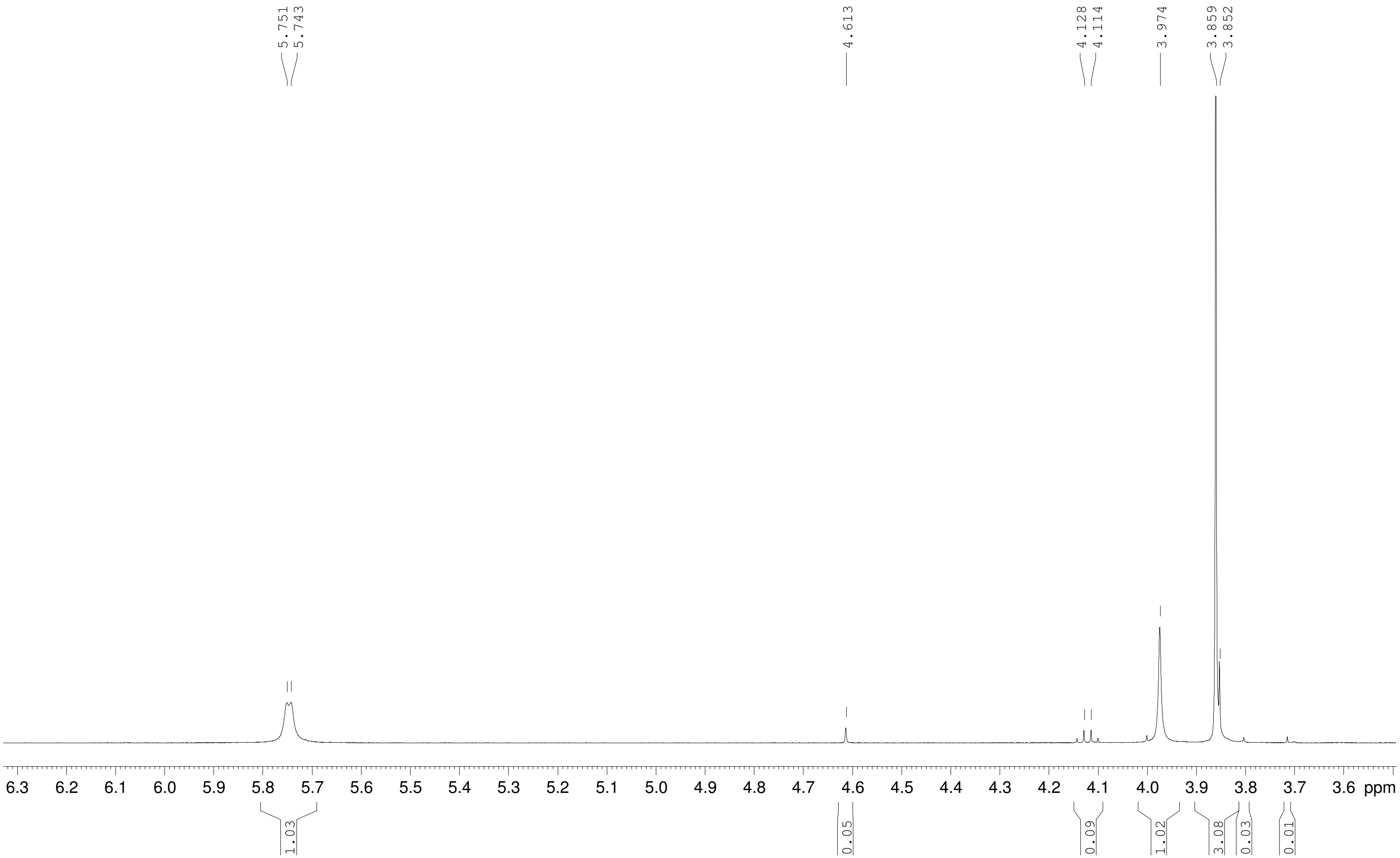

5.751
5.743
4.613
4.128
4.114
3.974
3.859
3.852
6.3 6.2 6.1 6.0 5.9 5.8 5.7 5.6 5.5 5.4 5.3 5.2 5.1 5.0 4.9 4.8 4.7 4.6 4.5 4.4 4.3 4.2 4.1 4.0 3.9 3.8 3.7 3.6 ppm
1.03
0.05
0.09
1.02
3.08
0.03
0.01

4362-1H
E

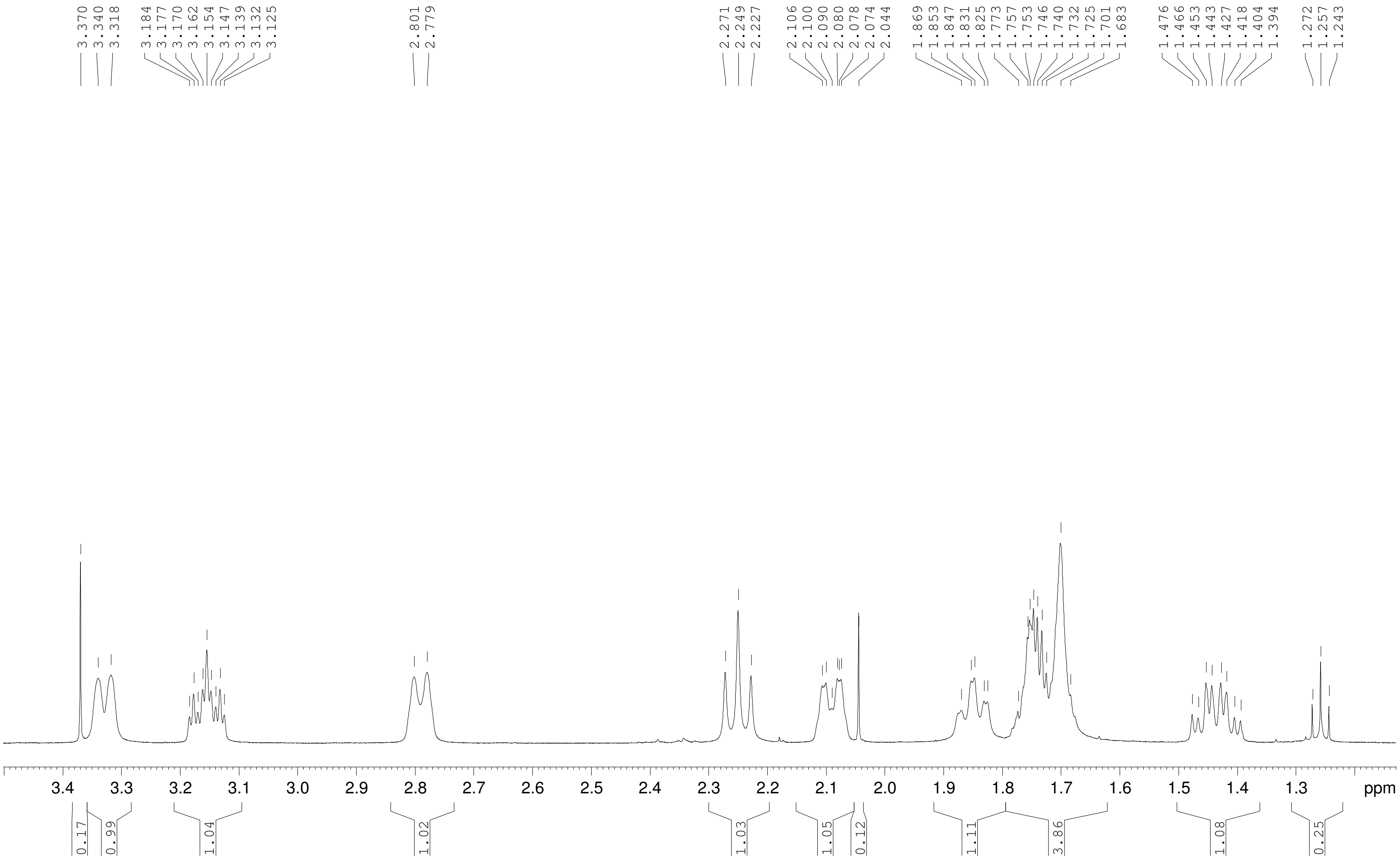
3.370
3.340
3.318
3.184
3.177
3.170
3.162
3.154
3.147
3.139
3.132
3.125
2.801
2.779
2.271
2.249
2.227
2.106
2.100
2.090
2.080
2.078
2.074
2.044
1.869
1.853
1.847
1.831
1.825
1.773
1.757
1.753
1.746
1.740
1.732
1.725
1.701
1.683
1.476
1.466
1.453
1.443
1.427
1.418
1.404
1.394
1.272
1.257
1.243
3.4
3.3
3.2
3.1
3.0
2.9
2.8
2.7
2.6
2.5
2.4
2.3
2.2
2.1
2.0
1.9
1.8
1.7
1.6
1.5
1.4
1.3
ppm
0.17
0.99
1.04
1.02
1.03
1.05
0.12
1.11
3.86
1.08
0.25

4501-13C
E

Current Data Parameters
NAME 4501
EXPNO 2
PROCNO 1

F2 - Acquisition Parameters
Date_ 20180124
PROBHD 5 mm CPPBBO BB
PULPROG zg_pi_CPD
SOLVENT CDCl3
TE 298.2 K
SWH 31249.381 Hz
AQ 1.0485969 sec
NS 992
FIDRES 0.476828 Hz
D1 1.50000000 sec
D11 0.03000000 sec
D14 0.00050000 sec

======== CHANNEL f1 ========
SFO1 125.7904831 MHz
NUC1 13C
P1 10.00 usec
P2 20.00 usec
PLW1 55.00000000 W

======== CHANNEL f2 ========
SFO2 500.2020008 MHz
NUC2 1H
PLW2 13.00000000 W
PLW12 0.25937000 W

F2 - Processing parameters
SI 65536
LB 0.50 Hz

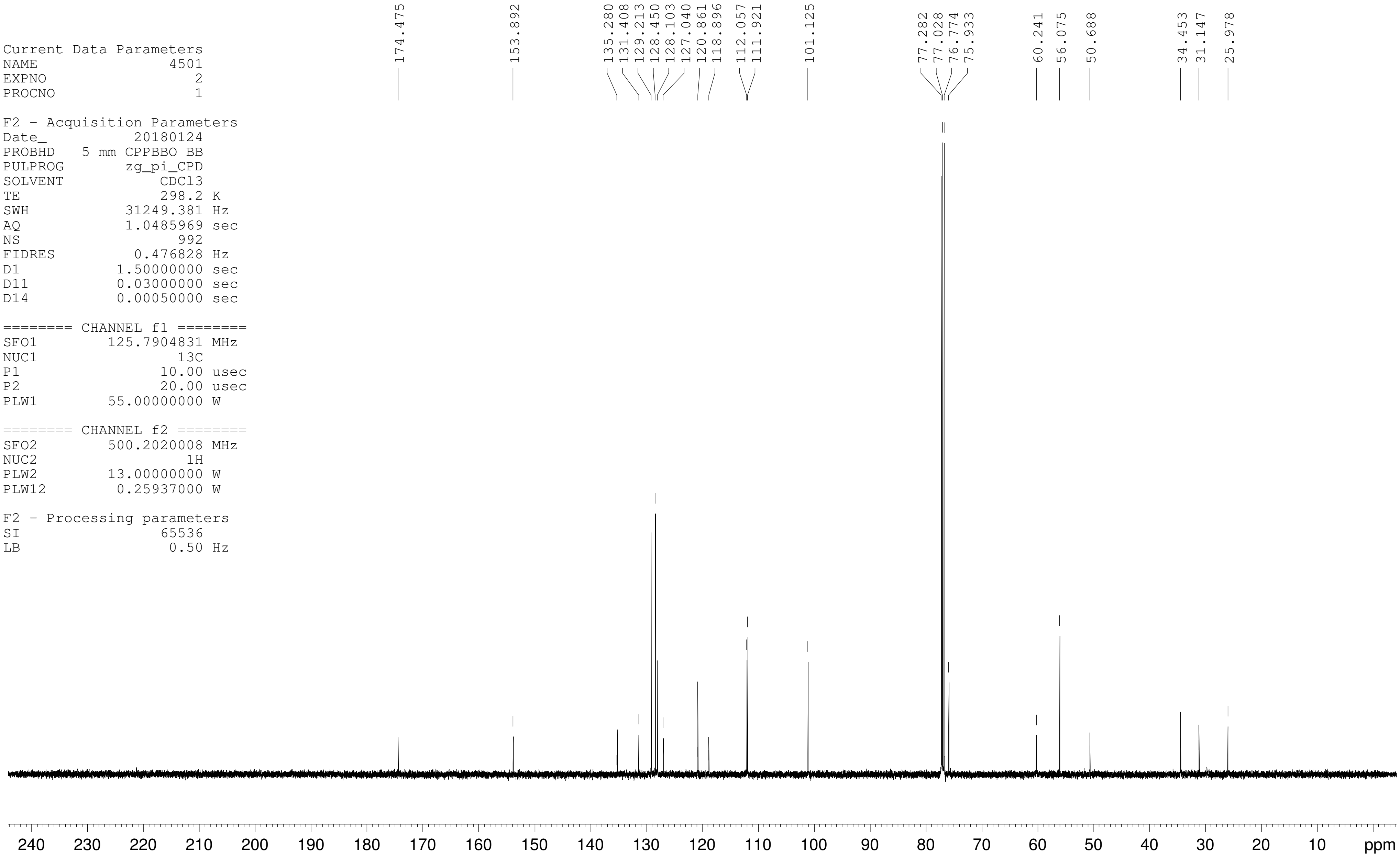

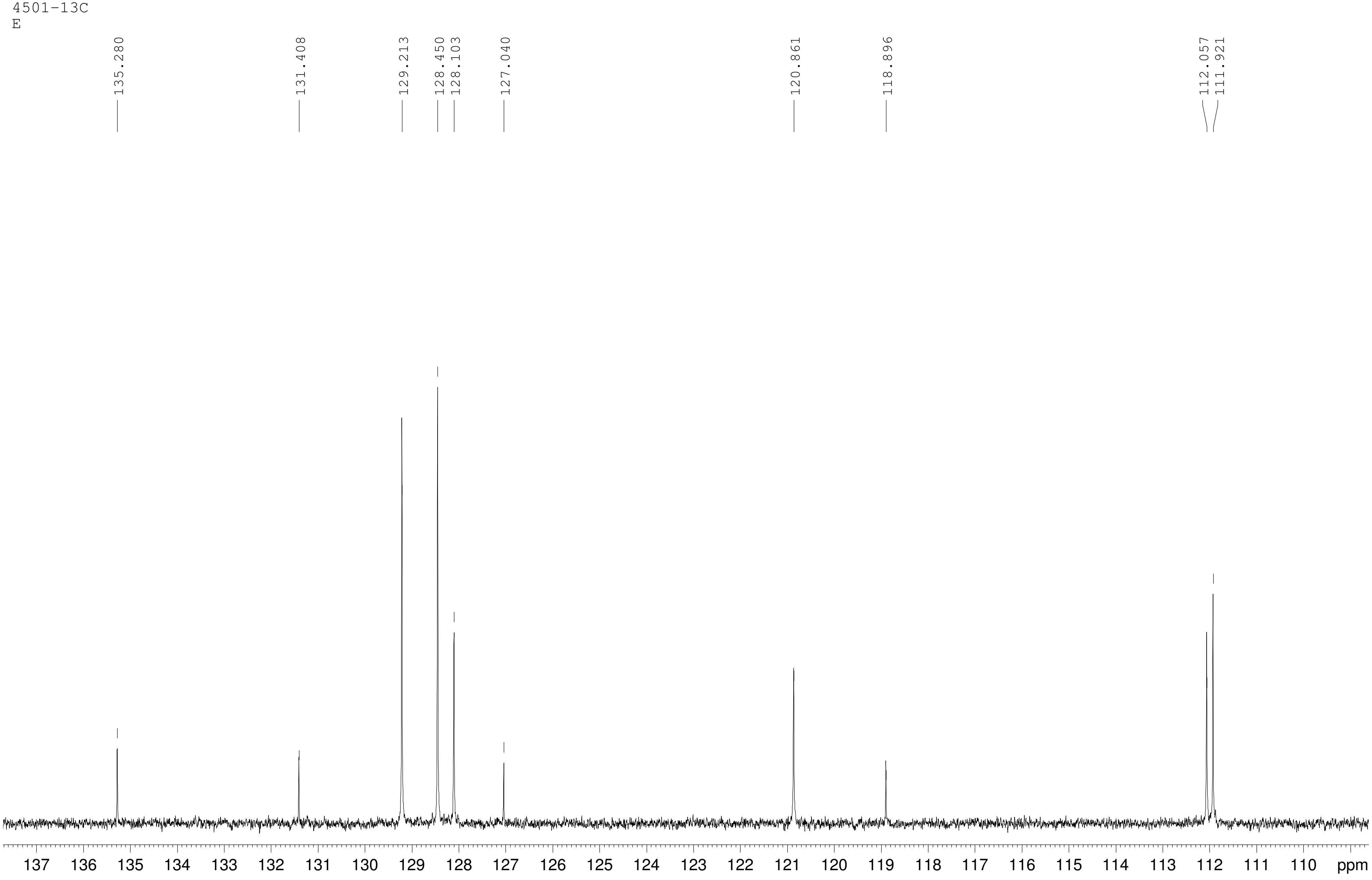
4501-13C
E
135.280
131.408
129.213
128.450
128.103
127.040
120.861
118.896
112.057
111.921
137 136 135 134 133 132 131 130 129 128 127 126 125 124 123 122 121 120 119 118 117 116 115 114 113 112 111 110 ppm

4501-13C
E

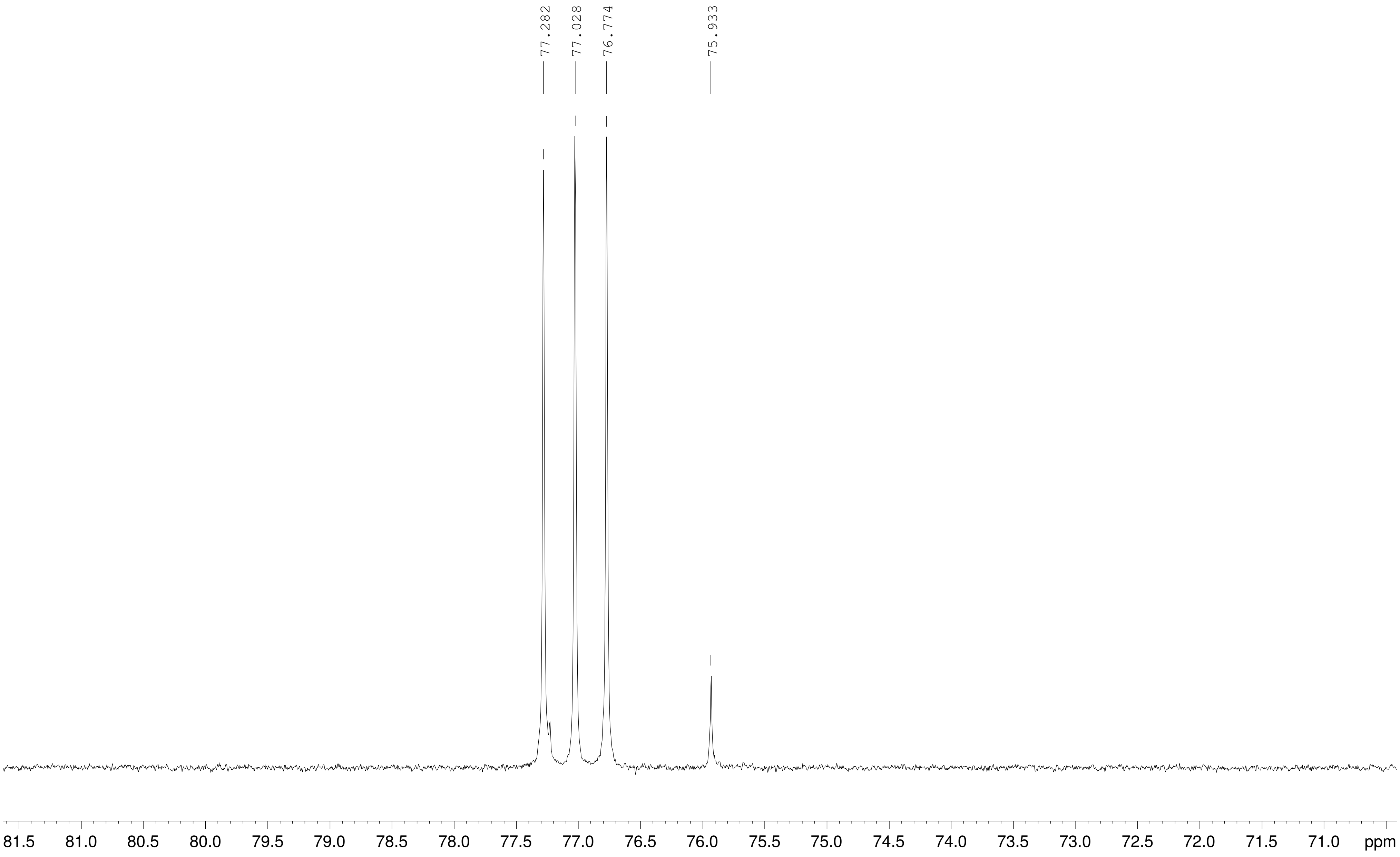
77.282
77.028
76.774
75.933
81.5 81.0 80.5 80.0 79.5 79.0 78.5 78.0 77.5 77.0 76.5 76.0 75.5 75.0 74.5 74.0 73.5 73.0 72.5 72.0 71.5 71.0 ppm

4391-COSY
E

Current Data Parameters
NAME 4391
EXPNO 2
PROCNO 1

F2 - Acquisition Parameters
Date_ 20171214
PROBHD 5 mm CPPBBO BB
PULPROG cosygpppqf
SOLVENT CDCl3
TE 298.1 K
SWH 5060.729 Hz
AQ 0.2023424 sec
NS 2
FIDRES 2.471059 Hz
D0 0.00000300 sec
D1 2.00000000 sec
D11 0.03000000 sec
D12 0.00002000 sec
D13 0.00000400 sec
D16 0.00020000 sec

======== CHANNEL f1 ========
SFO1 500.2020304 MHz
NUC1 1H
P0 11.30 usec
P1 11.30 usec
P17 0 usec
PLW1 13.69999981 W
PLW10 2.58780003 W

====== GRADIENT CHANNEL =====
P16 1000.00 usec

F1 - Acquisition parameters
TD 128
SFO1 500.202 MHz
FIDRES 39.536942 Hz
SW 10.117 ppm
FnMODE QF

F2 - Processing parameters
SI 2048
LB 0 Hz

F1 - Processing parameters
SI 1024
MC2 QF
SF 500.2000155 MHz
WDW QSINE
SSB 0
LB 0 Hz
GB 0

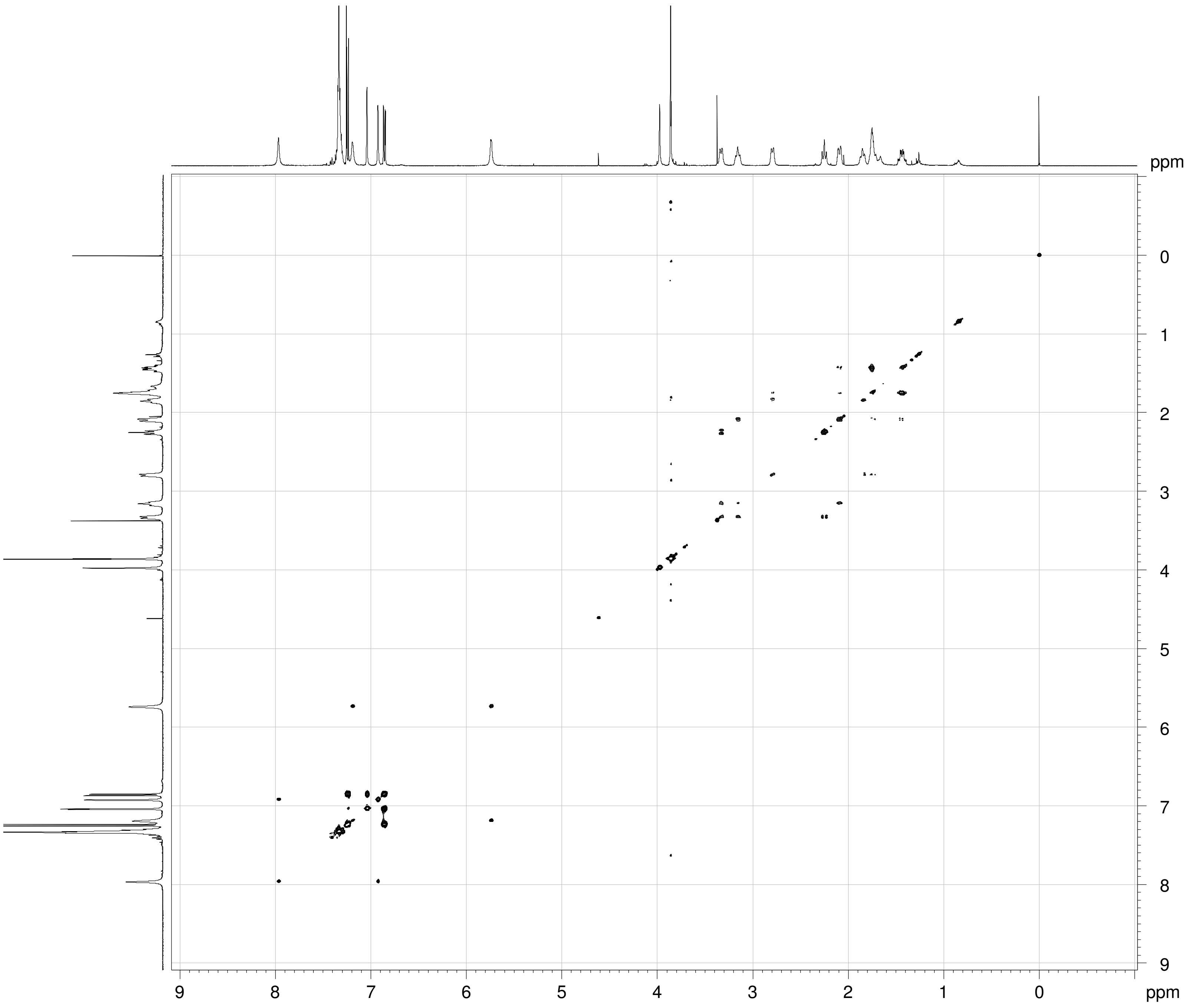

4391-COSY
E

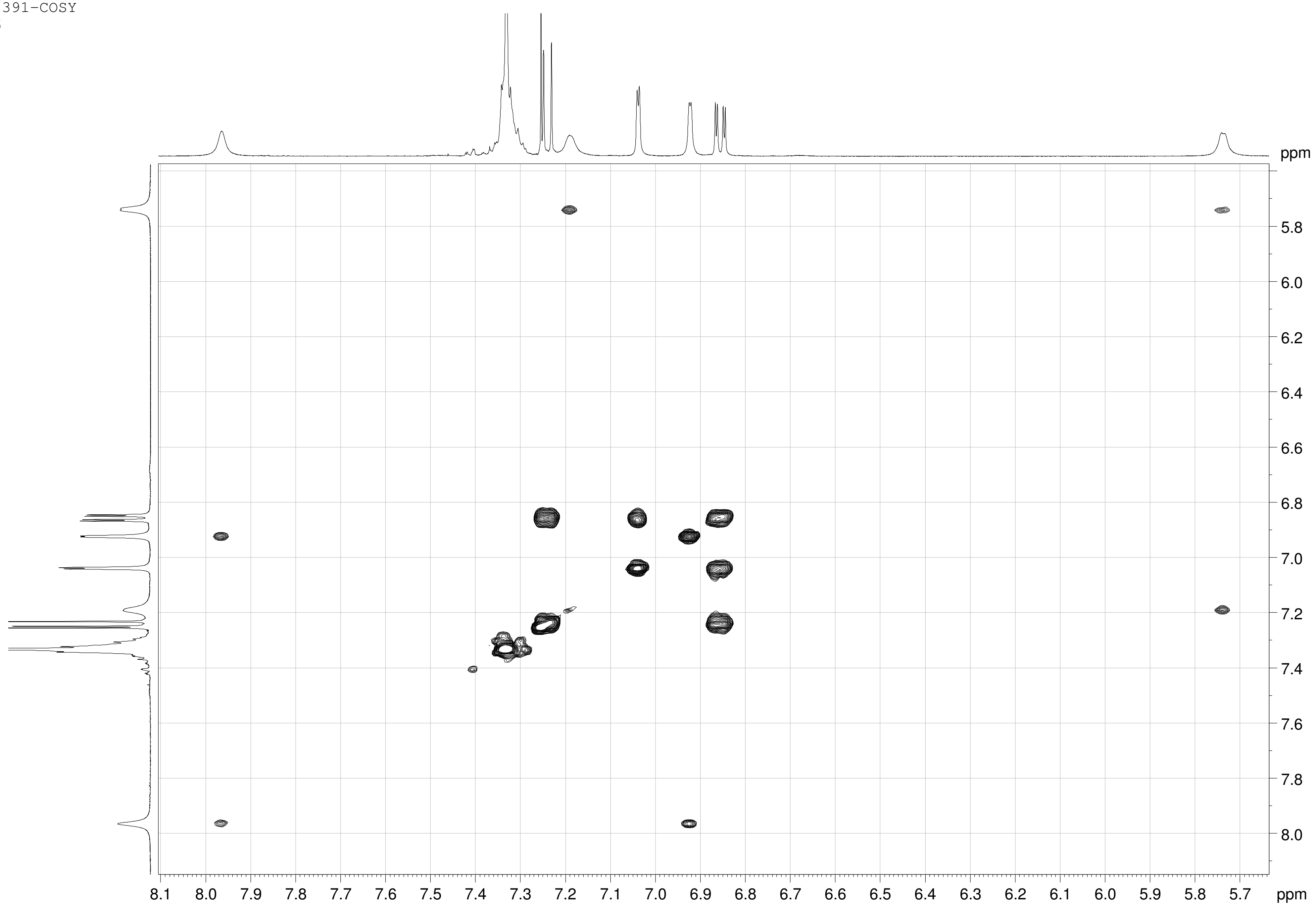

ppm
5.8
6.0
6.2
6.4
6.6
6.8
7.0
7.2
7.4
7.6
7.8
8.0
8.1 8.0 7.9 7.8 7.7 7.6 7.5 7.4 7.3 7.2 7.1 7.0 6.9 6.8 6.7 6.6 6.5 6.4 6.3 6.2 6.1 6.0 5.9 5.8 5.7
ppm

4391-COSY
E

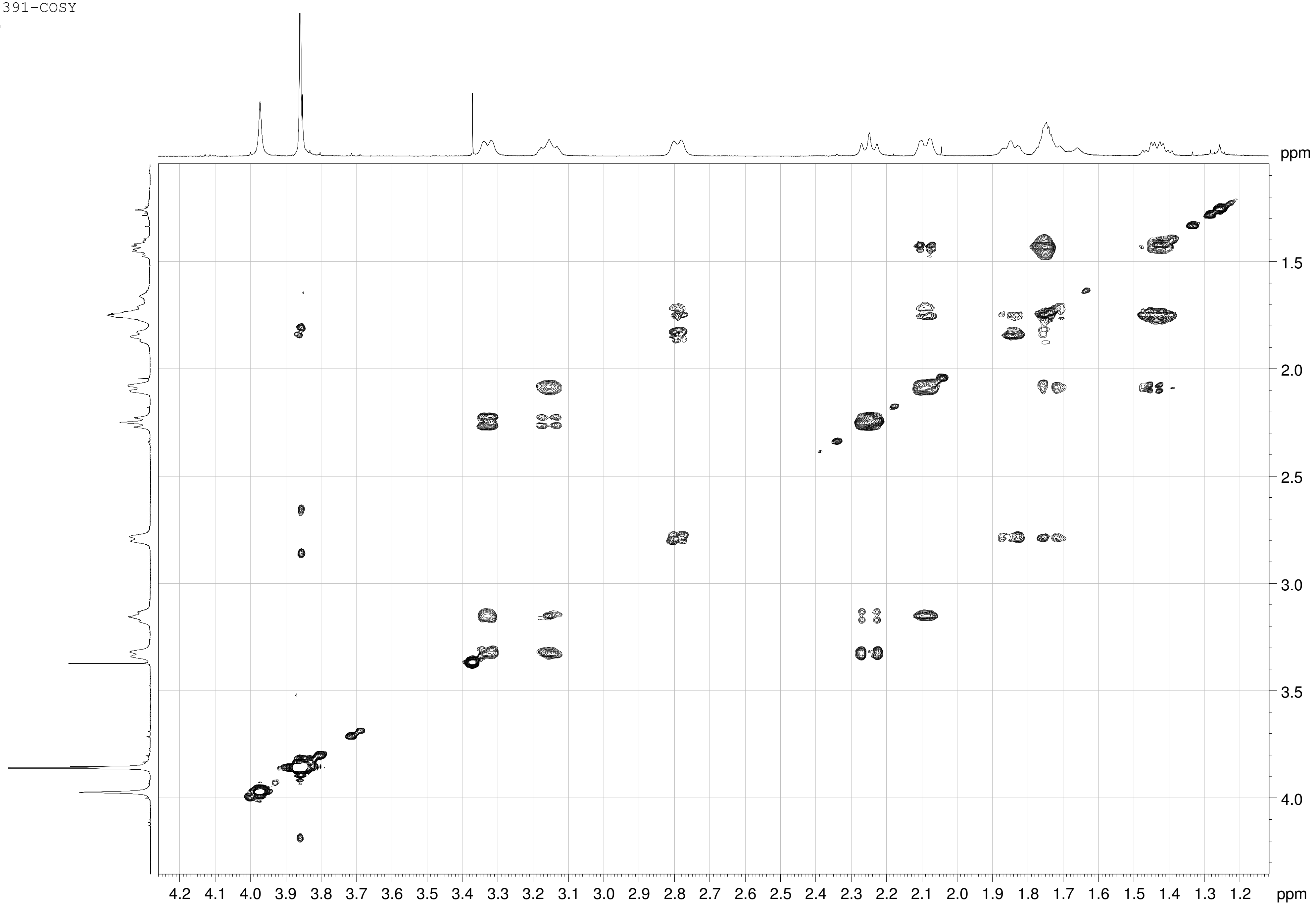
ppm
4.2 4.1 4.0 3.9 3.8 3.7 3.6 3.5 3.4 3.3 3.2 3.1 3.0 2.9 2.8 2.7 2.6 2.5 2.4 2.3 2.2 2.1 2.0 1.9 1.8 1.7 1.6 1.5 1.4 1.3 1.2
1.5 2.0 2.5 3.0 3.5 4.0

4501-HSQC
E

Current Data Parameters
NAME 4501
EXPNO 3
PROCNO 1

F2 - Acquisition Parameters
Date_ 20180124
PROBHD 5 mm CPPBBO BB
PULPROG hsqcedetgpsisp2.3
SOLVENT CDCl3
TE 298.2 K
SWH 5319.149 Hz
AQ 0.1925120 sec
NS 1
FIDRES 2.597241 Hz
D0 0.00000300 sec
D1 2.00000000 sec
D4 0.00172414 sec
D11 0.03000000 sec
D16 0.00020000 sec
D21 0.00360000 sec
D24 0.00089000 sec

======== CHANNEL f1 ========
SFO1 500.2021450 MHz
NUC1 1H
P1 11.30 usec
P2 22.60 usec
P28 0 usec
PLW1 13.69999981 W

======== CHANNEL f2 ========
SFO2 125.7879585 MHz
NUC2 13C
P3 10.00 usec
P14 500.00 usec
P24 2000.00 usec
P31 1900.00 usec
PLW0 0 W
PLW2 55.00000000 W
PLW12 1.30180001 W

====== GRADIENT CHANNEL =====
P16 1000.00 usec
P19 600.00 usec

F1 - Acquisition parameters
TD 128
SFO1 125.788 MHz
FIDRES 185.130325 Hz
SW 188.386 ppm
FnMODE Echo-Antiecho

F2 - Processing parameters

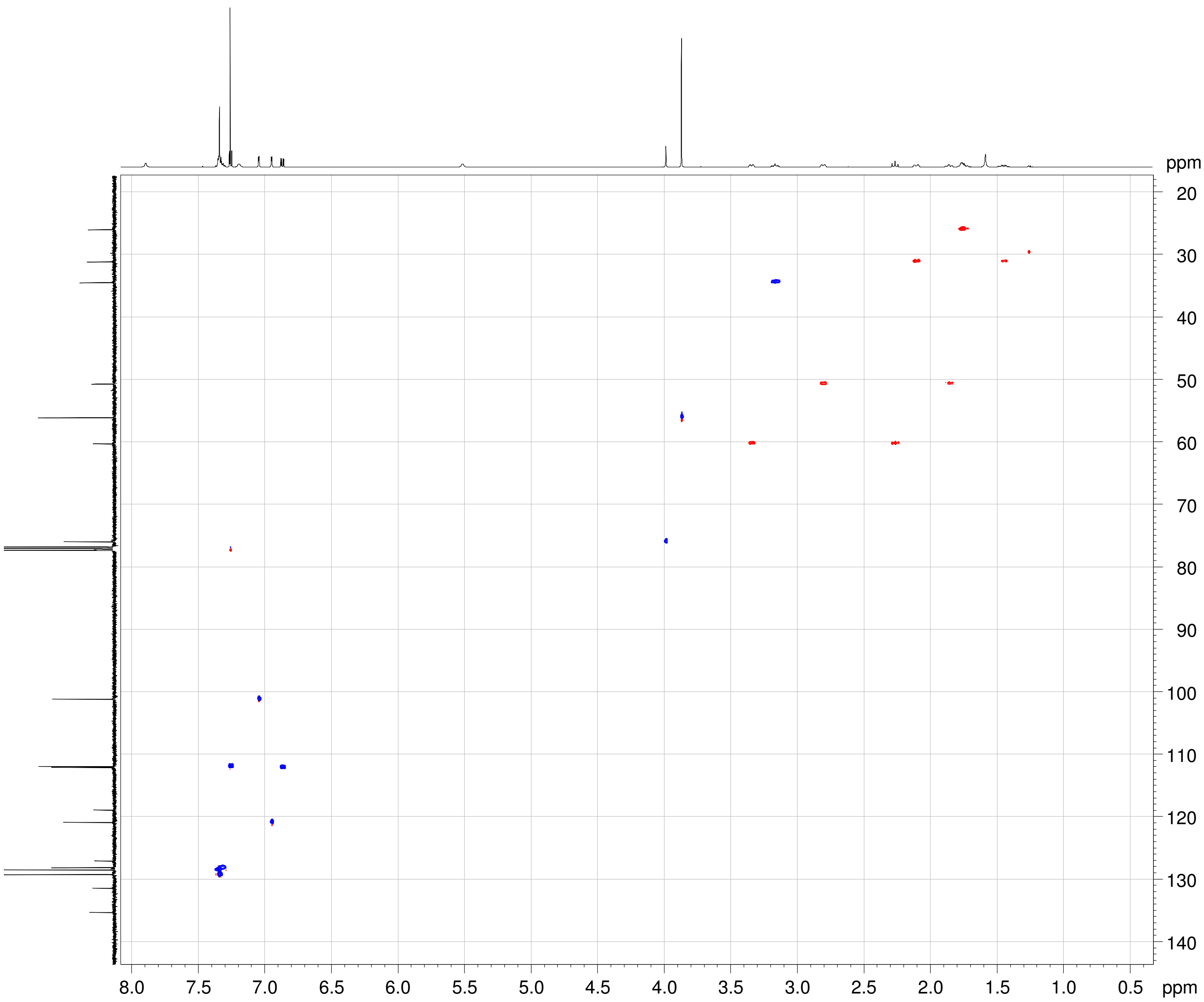

4501-HSQC
E

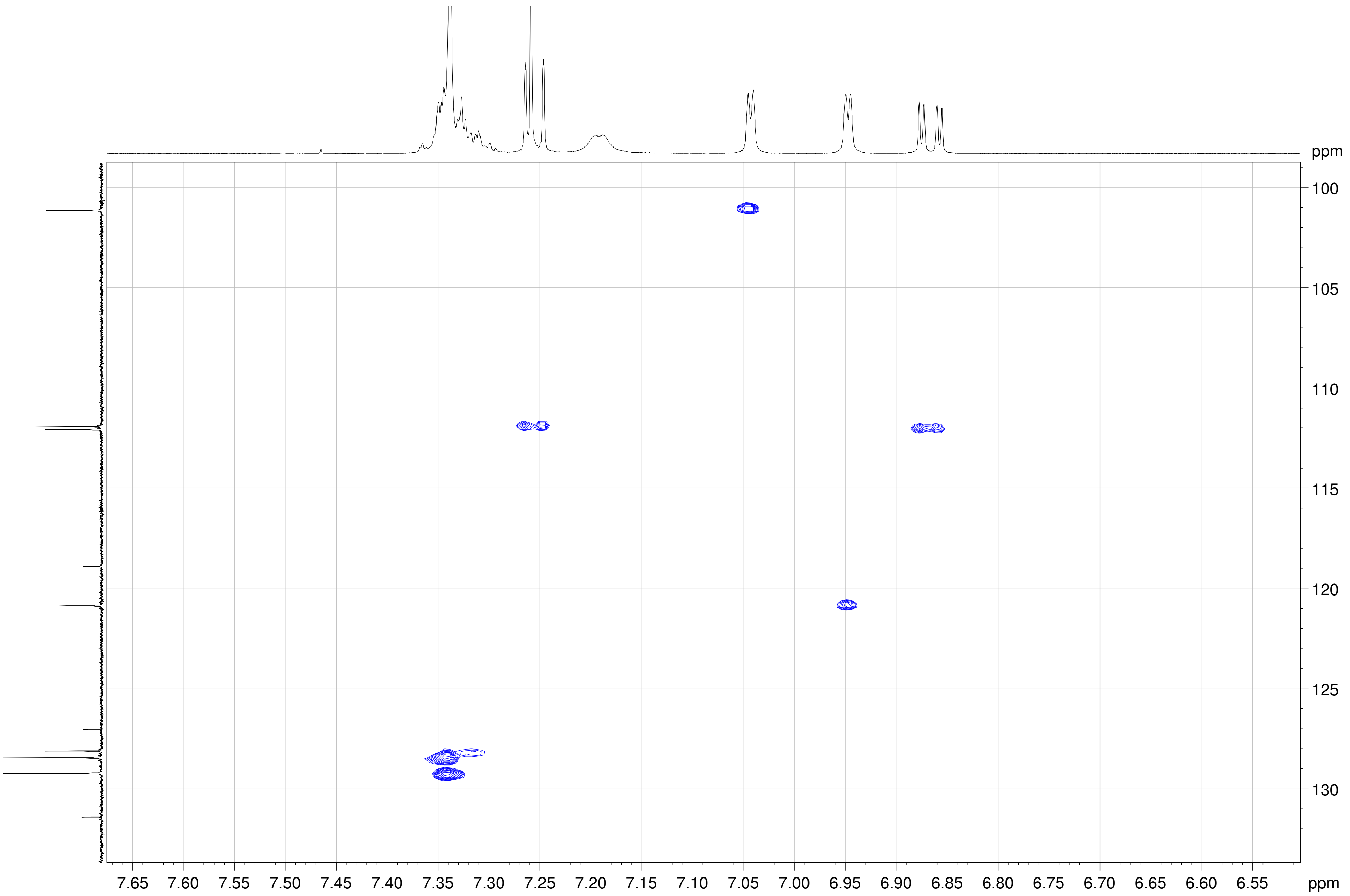

ppm
100
105
110
115
120
125
130
7.65
7.60
7.55
7.50
7.45
7.40
7.35
7.30
7.25
7.20
7.15
7.10
7.05
7.00
6.95
6.90
6.85
6.80
6.75
6.70
6.65
6.60
6.55
ppm

4501-HSQC
E

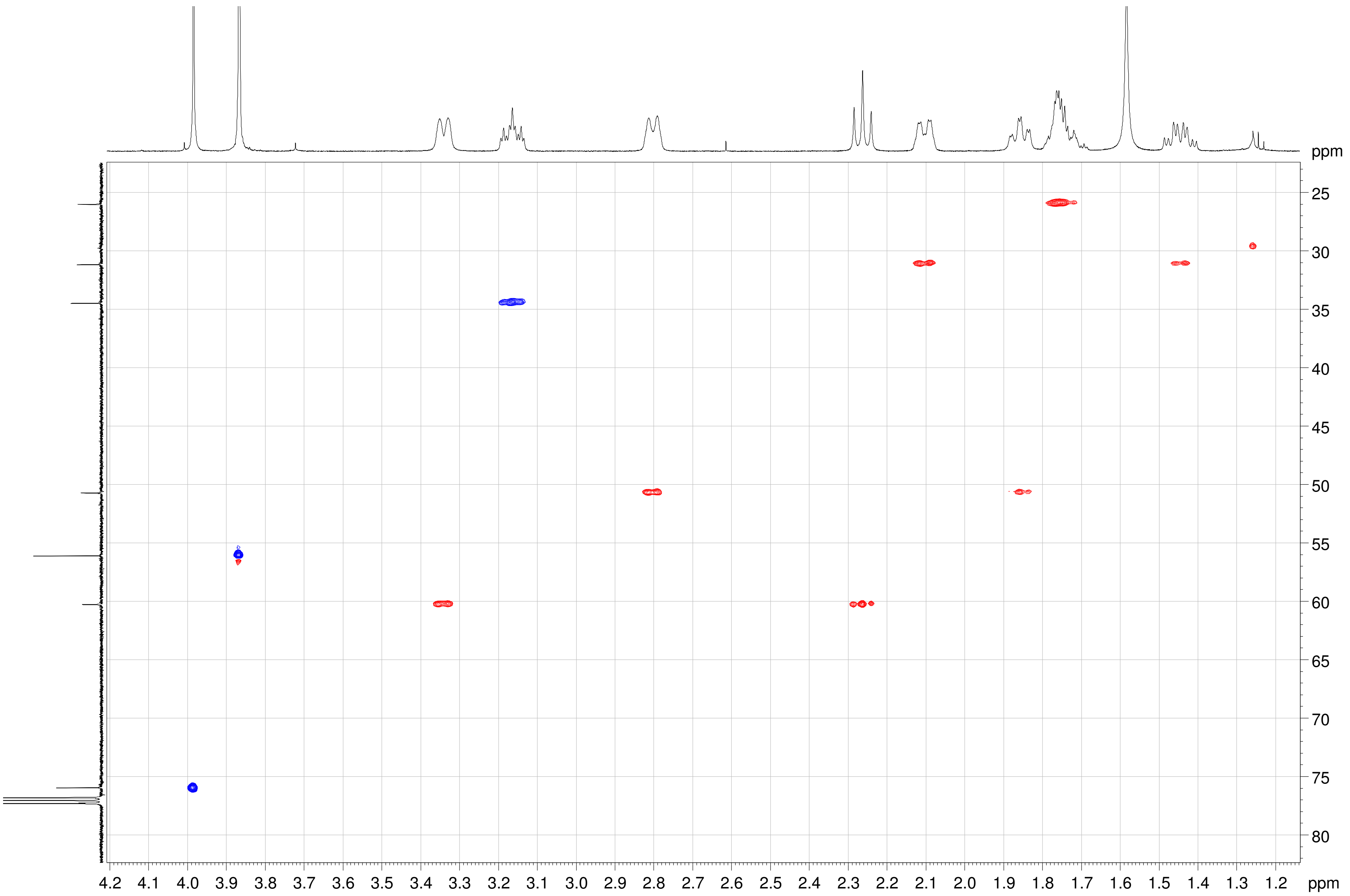

ppm
25
30
35
40
45
50
55
60
65
70
75
80
4.2 4.1 4.0 3.9 3.8 3.7 3.6 3.5 3.4 3.3 3.2 3.1 3.0 2.9 2.8 2.7 2.6 2.5 2.4 2.3 2.2 2.1 2.0 1.9 1.8 1.7 1.6 1.5 1.4 1.3 1.2
ppm

(3S,2R)-9c

4363-1H
F

Current Data Parameters
NAME 4363
EXPNO 1
PROCNO 1

F2 - Acquisition Parameters
Date_ 20171129
PROBHD 5 mm CPPBBO BB
PULPROG zg30
SOLVENT CDCl3
TE 298.2 K
SWH 10000.000 Hz
AQ 3.2767999 sec
NS 8
FIDRES 0.152588 Hz
D1 0 sec

======== CHANNEL f1 ========
SFO1 500.2030889 MHz
NUC1 1H
P1 11.30 usec
PLW1 13.69999981 W

F2 - Processing parameters
SI 65536
LB 0 Hz

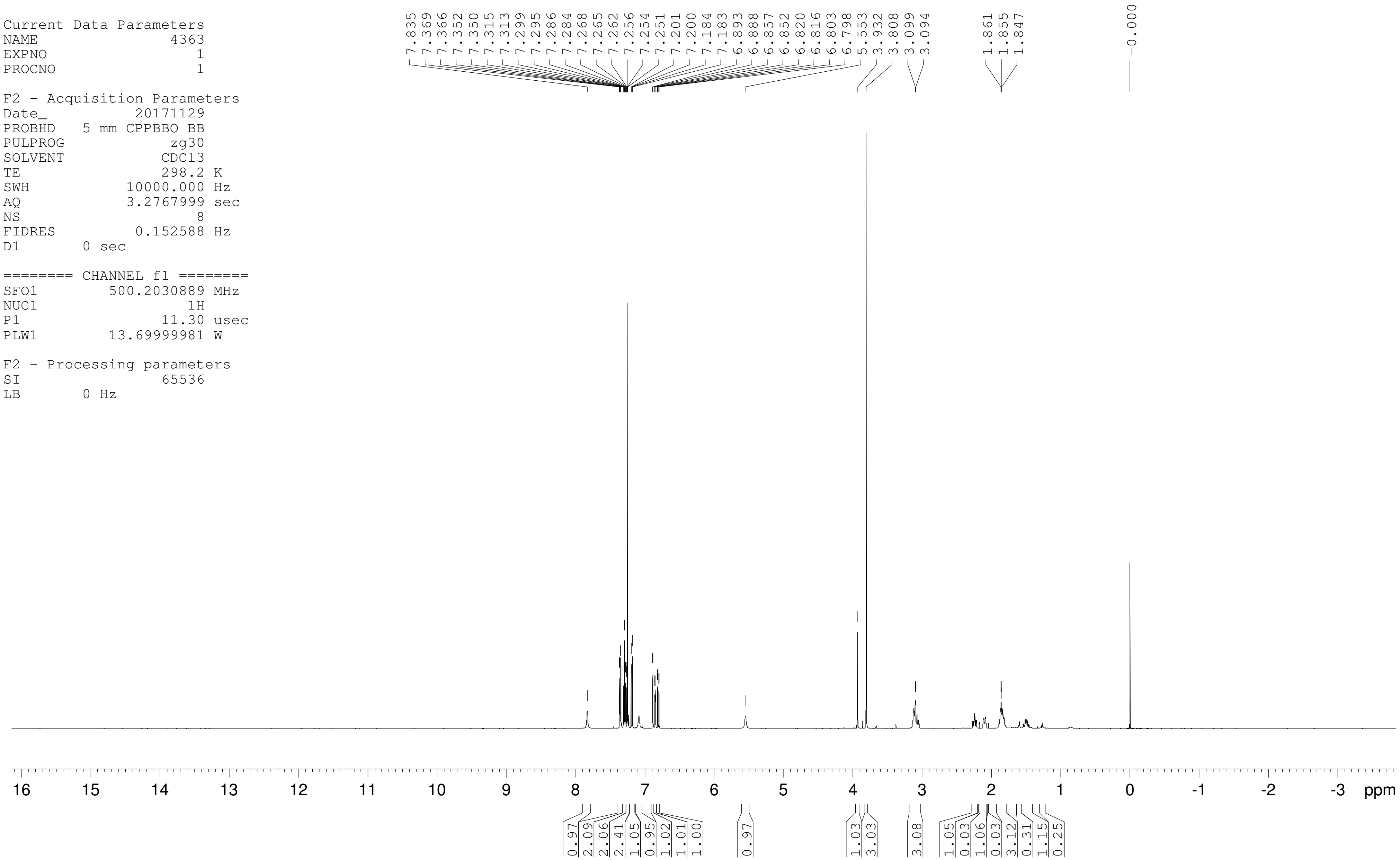

4363-1H
F

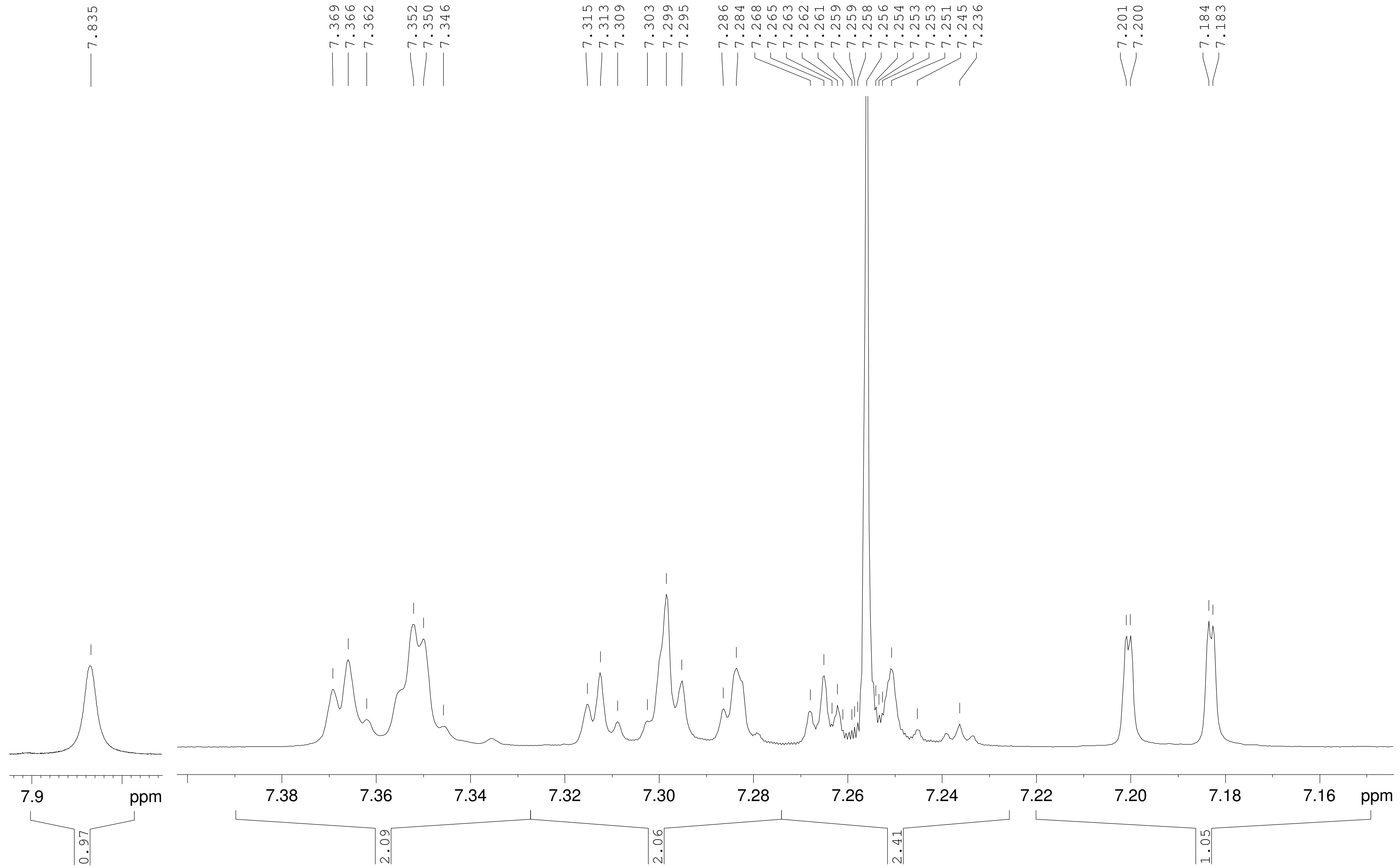

7.835
7.369
7.366
7.362
7.352
7.350
7.346
7.315
7.313
7.309
7.303
7.299
7.295
7.286
7.284
7.268
7.265
7.263
7.262
7.261
7.259
7.259
7.258
7.256
7.254
7.253
7.253
7.251
7.245
7.236
7.201
7.200
7.184
7.183
7.9
ppm
7.38
7.36
7.34
7.32
7.30
7.28
7.26
7.24
7.22
7.20
7.18
7.16
ppm
0.97
2.09
2.06
2.41
1.05

4363-1H
F

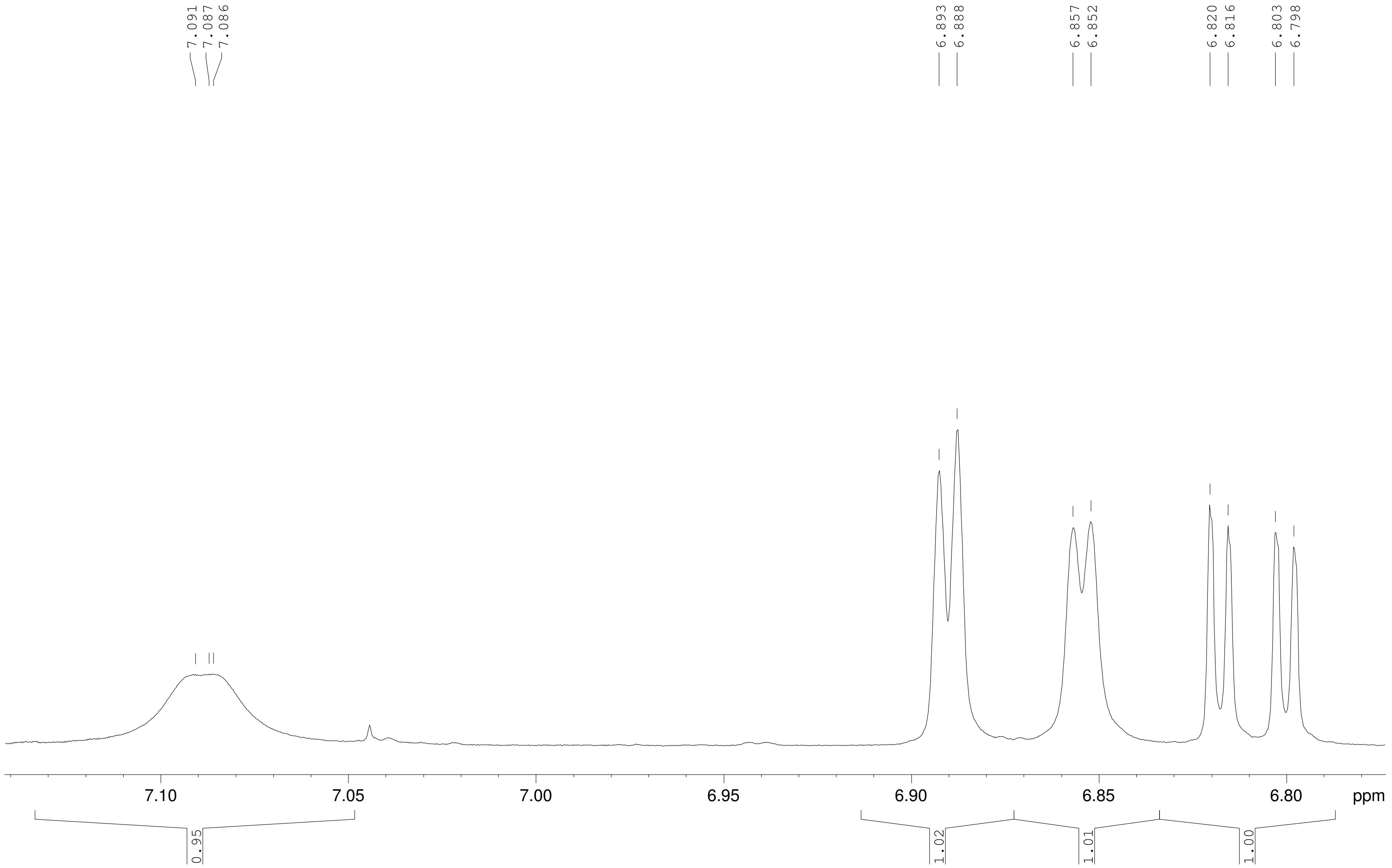
7.091
7.087
7.086
6.893
6.888
6.857
6.852
6.820
6.816
6.803
6.798
7.10
7.05
7.00
6.95
6.90
6.85
6.80
ppm
0.95
1.02
1.01
1.00

4363-1H
F

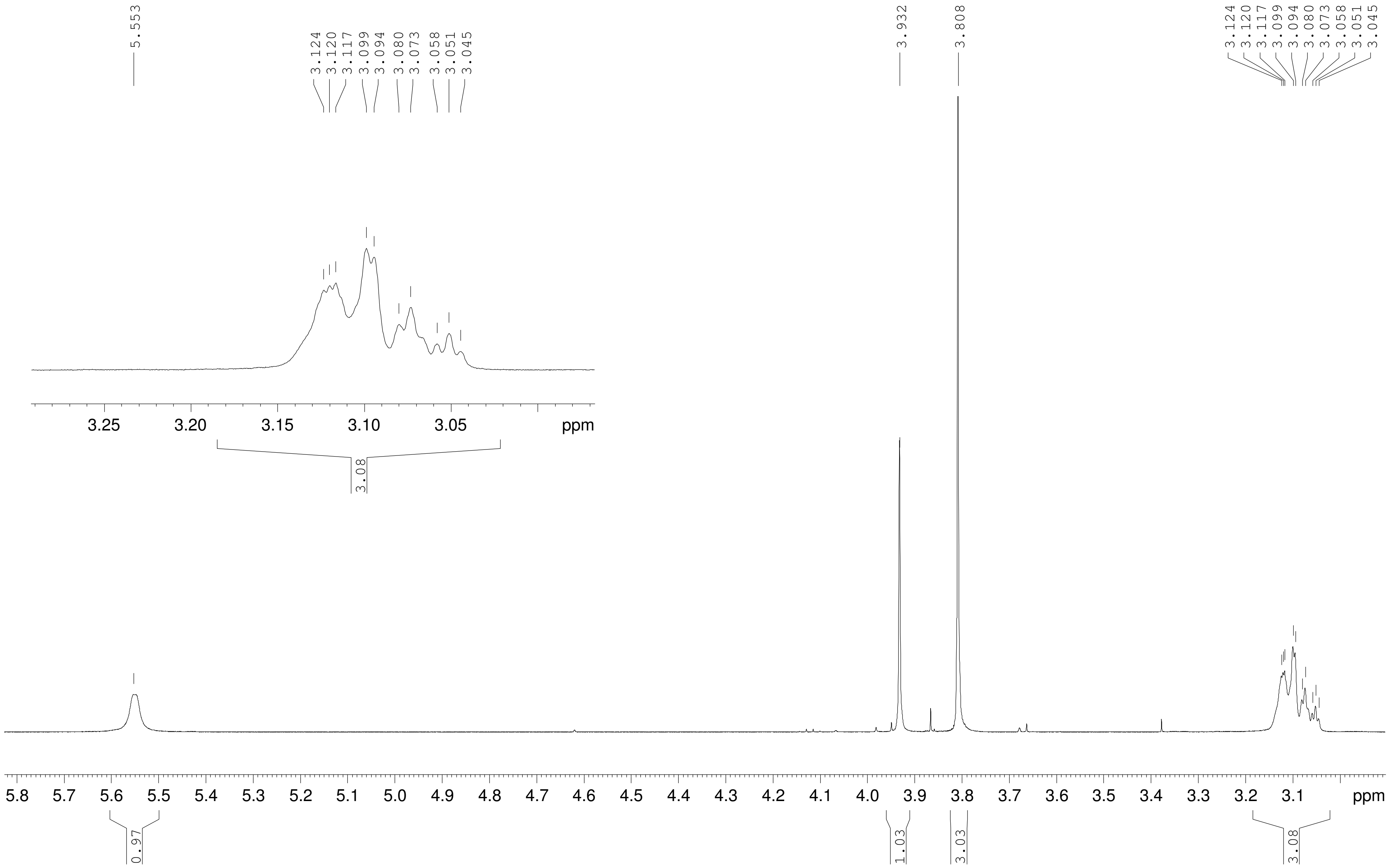

5.553
3.124
3.120
3.117
3.099
3.094
3.080
3.073
3.058
3.051
3.045
3.932
3.808
3.25
3.20
3.15
3.10
3.05
ppm
3.08
5.8
5.7
5.6
5.5
5.4
5.3
5.2
5.1
5.0
4.9
4.8
4.7
4.6
4.5
4.4
4.3
4.2
4.1
4.0
3.9
3.8
3.7
3.6
3.5
3.4
3.3
3.2
3.1
ppm
0.97
1.03
3.03
3.08

4363-1H
F

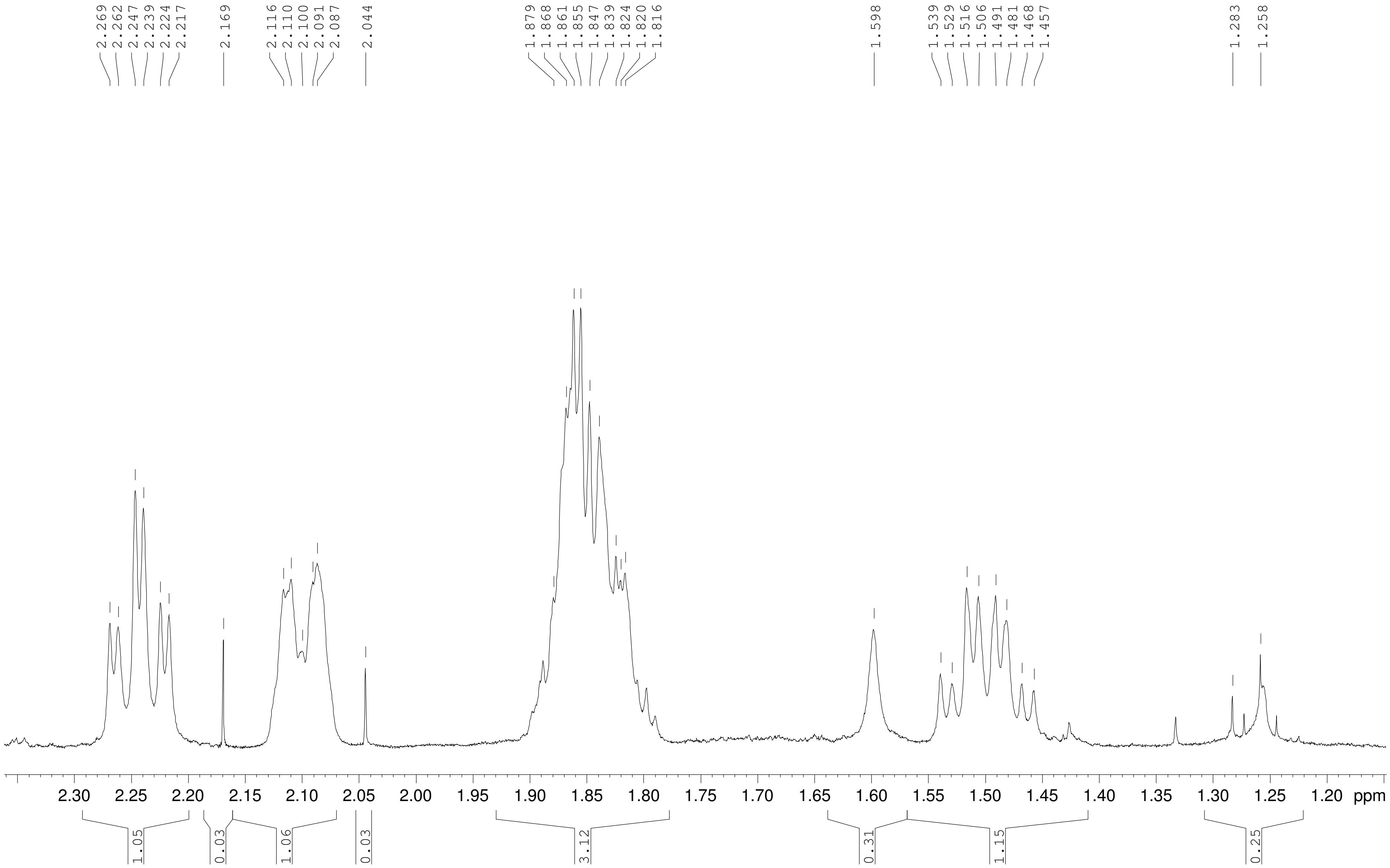
2.269
2.262
2.247
2.239
2.224
2.217
2.169
2.116
2.110
2.100
2.091
2.087
2.044
1.879
1.868
1.861
1.855
1.847
1.839
1.824
1.820
1.816
1.598
1.539
1.529
1.516
1.506
1.491
1.481
1.468
1.457
1.283
1.258
2.30 2.25 2.20 2.15 2.10 2.05 2.00 1.95 1.90 1.85 1.80 1.75 1.70 1.65 1.60 1.55 1.50 1.45 1.40 1.35 1.30 1.25 1.20 ppm
1.05
0.03
1.06
0.03
3.12
0.31
1.15
0.25

4502-13C
F

Current Data Parameters
NAME 4502
EXPNO 2
PROCNO 1

F2 - Acquisition Parameters
Date_ 20180124
PROBHD 5 mm CPPBBO BB
PULPROG zg_pi_CPD
SOLVENT CDCl3
TE 298.2 K
SWH 31249.381 Hz
AQ 1.0485969 sec
NS 908
FIDRES 0.476828 Hz
D1 1.50000000 sec
D11 0.03000000 sec
D14 0.00050000 sec

======== CHANNEL f1 ========
SFO1 125.7904831 MHz
NUC1 13C
P1 10.00 usec
P2 20.00 usec
PLW1 55.00000000 W

======== CHANNEL f2 ========
SFO2 500.2020008 MHz
NUC2 1H
PLW2 13.00000000 W
PLW12 0.25937000 W

F2 - Processing parameters
SI 65536
LB 0.50 Hz

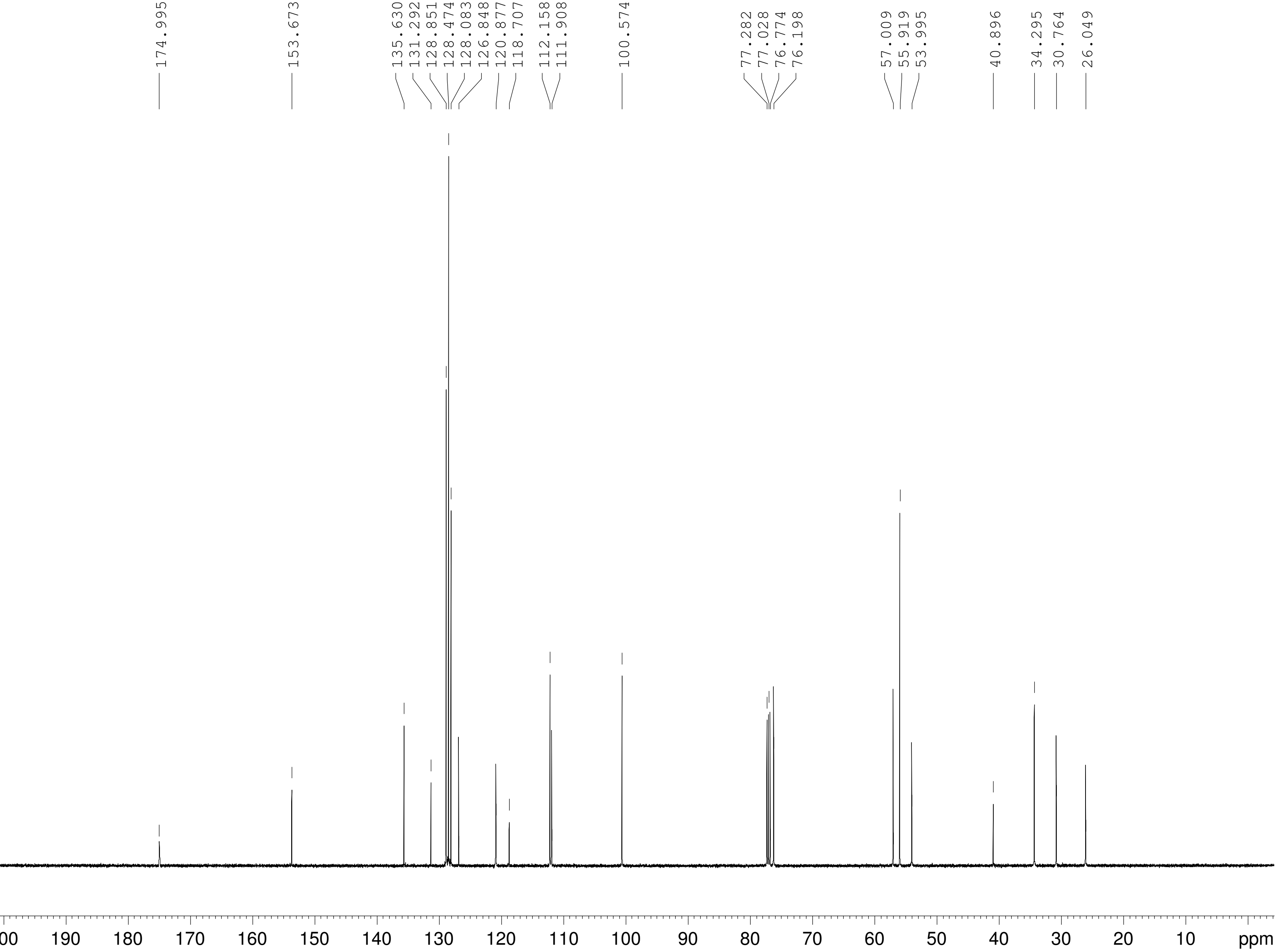

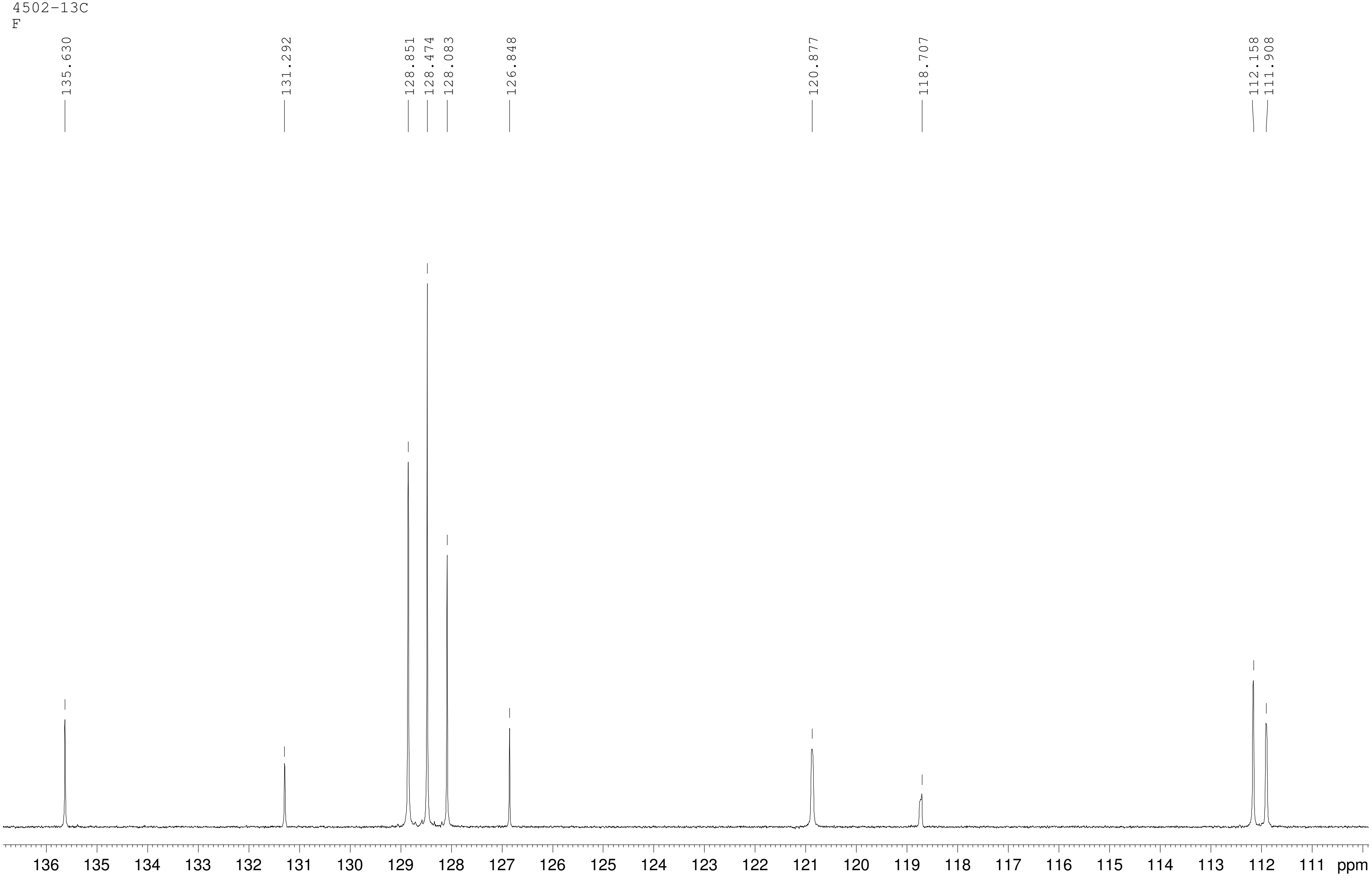

4502-13C
F
135.630
131.292
128.851
128.474
128.083
126.848
120.877
118.707
112.158
111.908
136 135 134 133 132 131 130 129 128 127 126 125 124 123 122 121 120 119 118 117 116 115 114 113 112 111 ppm

4502-13C
F

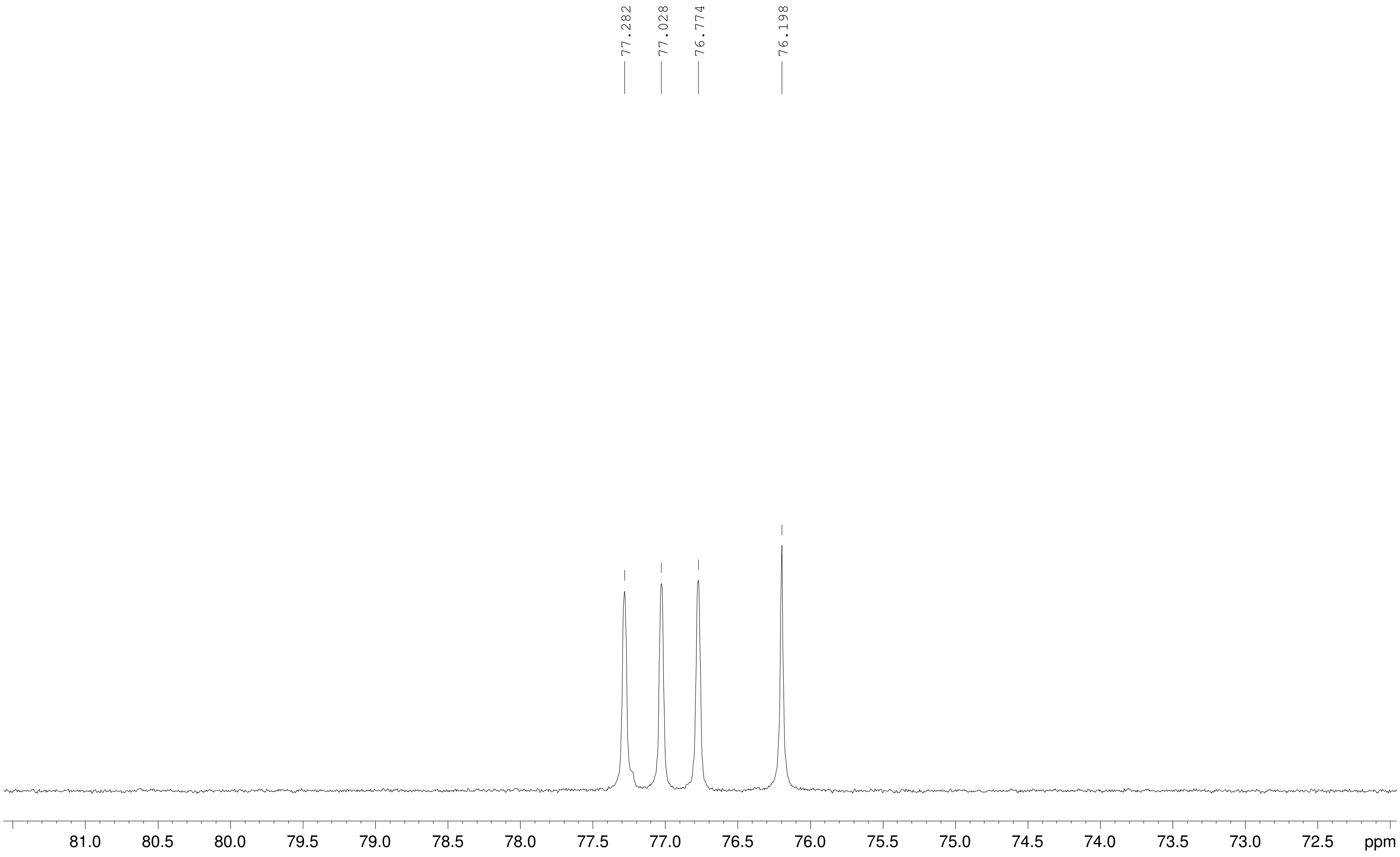
77.282
77.028
76.774
76.198
81.0
80.5
80.0
79.5
79.0
78.5
78.0
77.5
77.0
76.5
76.0
75.5
75.0
74.5
74.0
73.5
73.0
72.5
ppm

4392-COSY
F

Current Data Parameters
NAME 4392
EXPNO 2
PROCNO 1

F2 - Acquisition Parameters
Date_ 20171214
PROBHD 5 mm CPPBBO BB
PULPROG cosygpppqf
SOLVENT CDCl3
TE 298.2 K
SWH 5266.854 Hz
AQ 0.1944235 sec
NS 2
FIDRES 2.571706 Hz
D0 0.00000300 sec
D1 2.00000000 sec
D11 0.03000000 sec
D12 0.00002000 sec
D13 0.00000400 sec
D16 0.00020000 sec

======== CHANNEL f1 ========
SFO1 500.2020514 MHz
NUC1 1H
P0 11.30 usec
P1 11.30 usec
P17 0 usec
PLW1 13.69999981 W
PLW10 2.58780003 W

====== GRADIENT CHANNEL =====
P16 1000.00 usec

F1 - Acquisition parameters
TD 128
SFO1 500.2021 MHz
FIDRES 41.161751 Hz
SW 10.533 ppm
FnMODE QF

F2 - Processing parameters
SI 2048
LB 0 Hz

F1 - Processing parameters
SI 1024
MC2 QF
SF 500.2000131 MHz
WDW QSINE
SSB 0
LB 0 Hz
GB 0

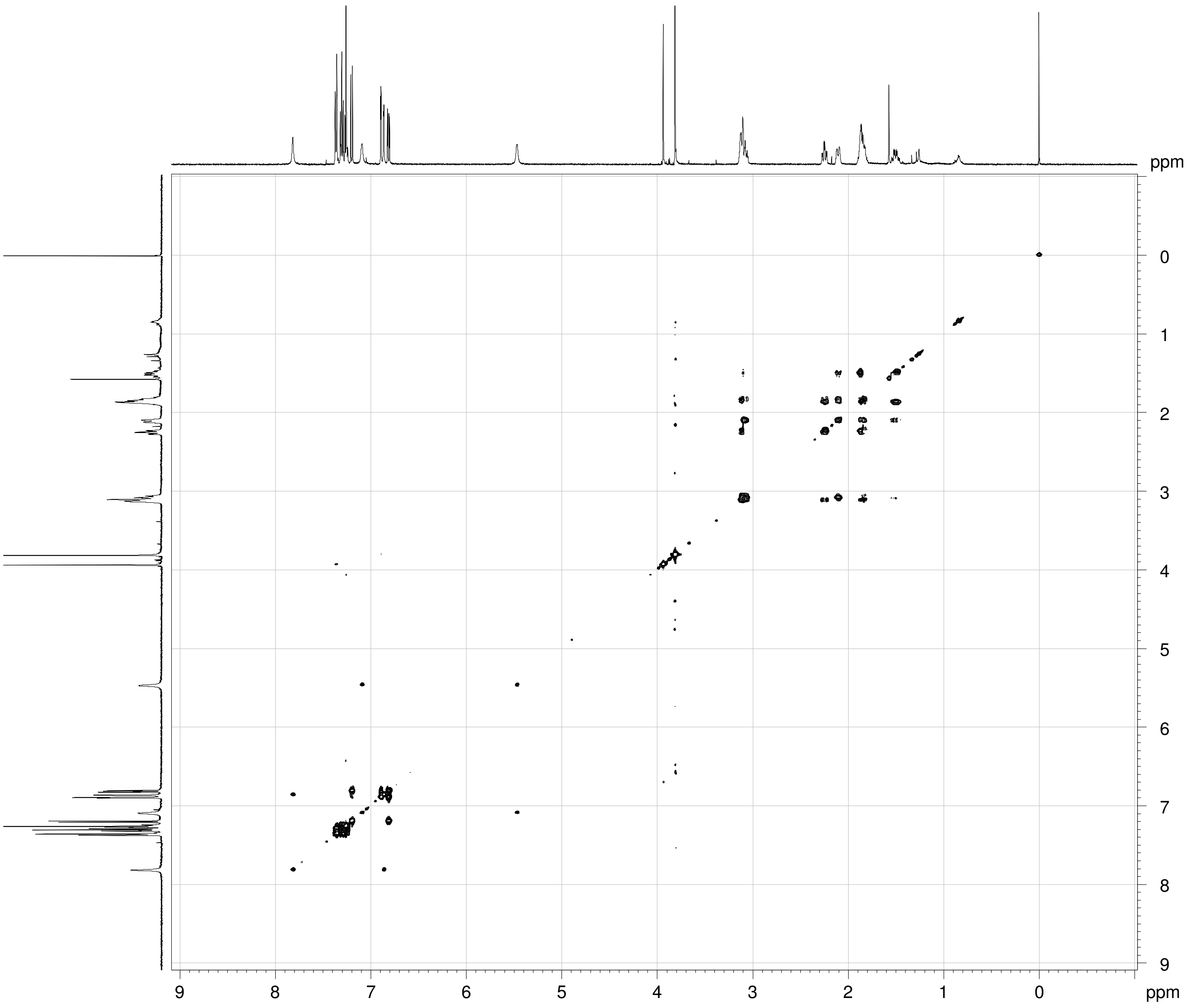

4392-COSY
F

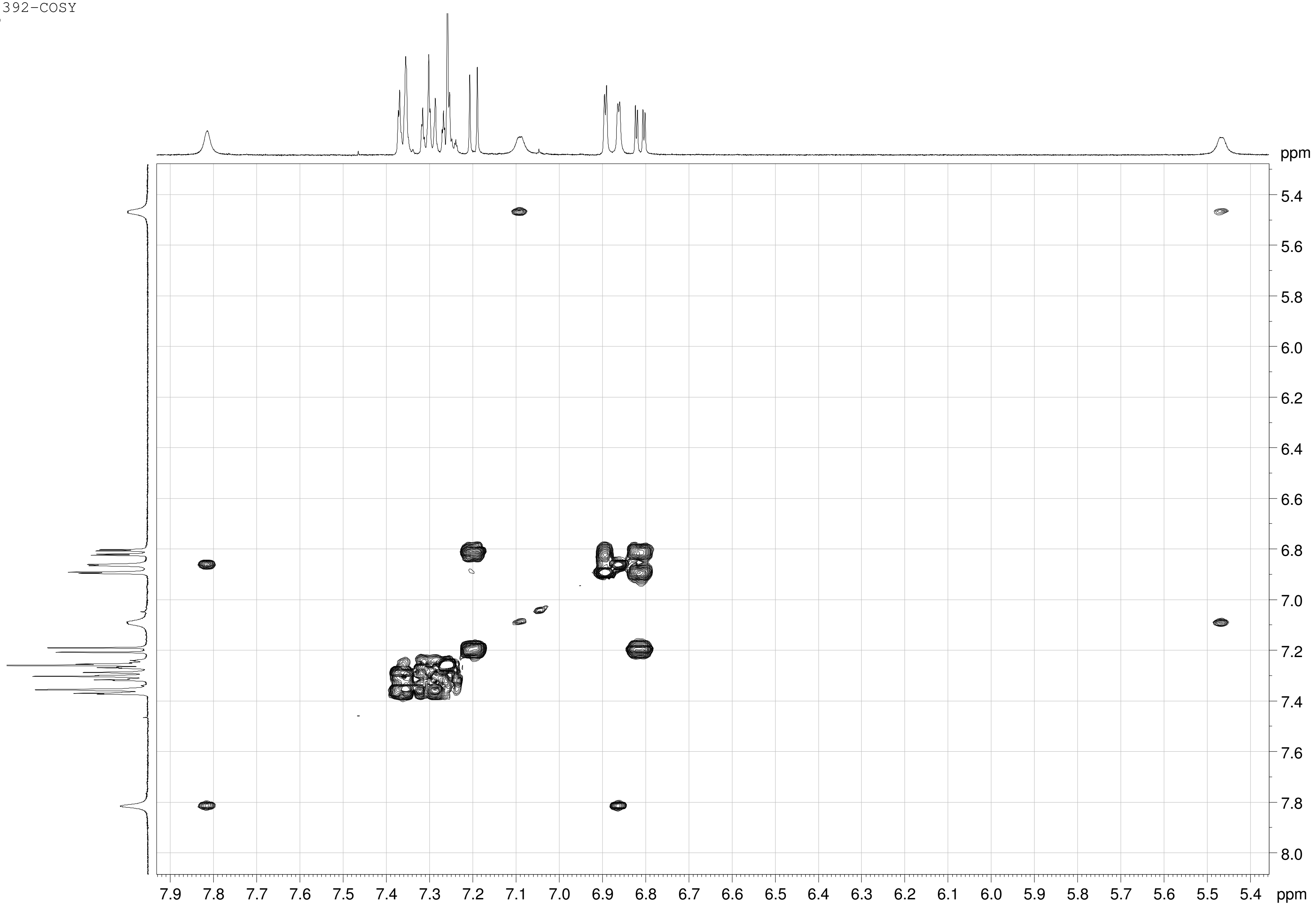
ppm
5.4
5.6
5.8
6.0
6.2
6.4
6.6
6.8
7.0
7.2
7.4
7.6
7.8
8.0
7.9 7.8 7.7 7.6 7.5 7.4 7.3 7.2 7.1 7.0 6.9 6.8 6.7 6.6 6.5 6.4 6.3 6.2 6.1 6.0 5.9 5.8 5.7 5.6 5.5 5.4
ppm

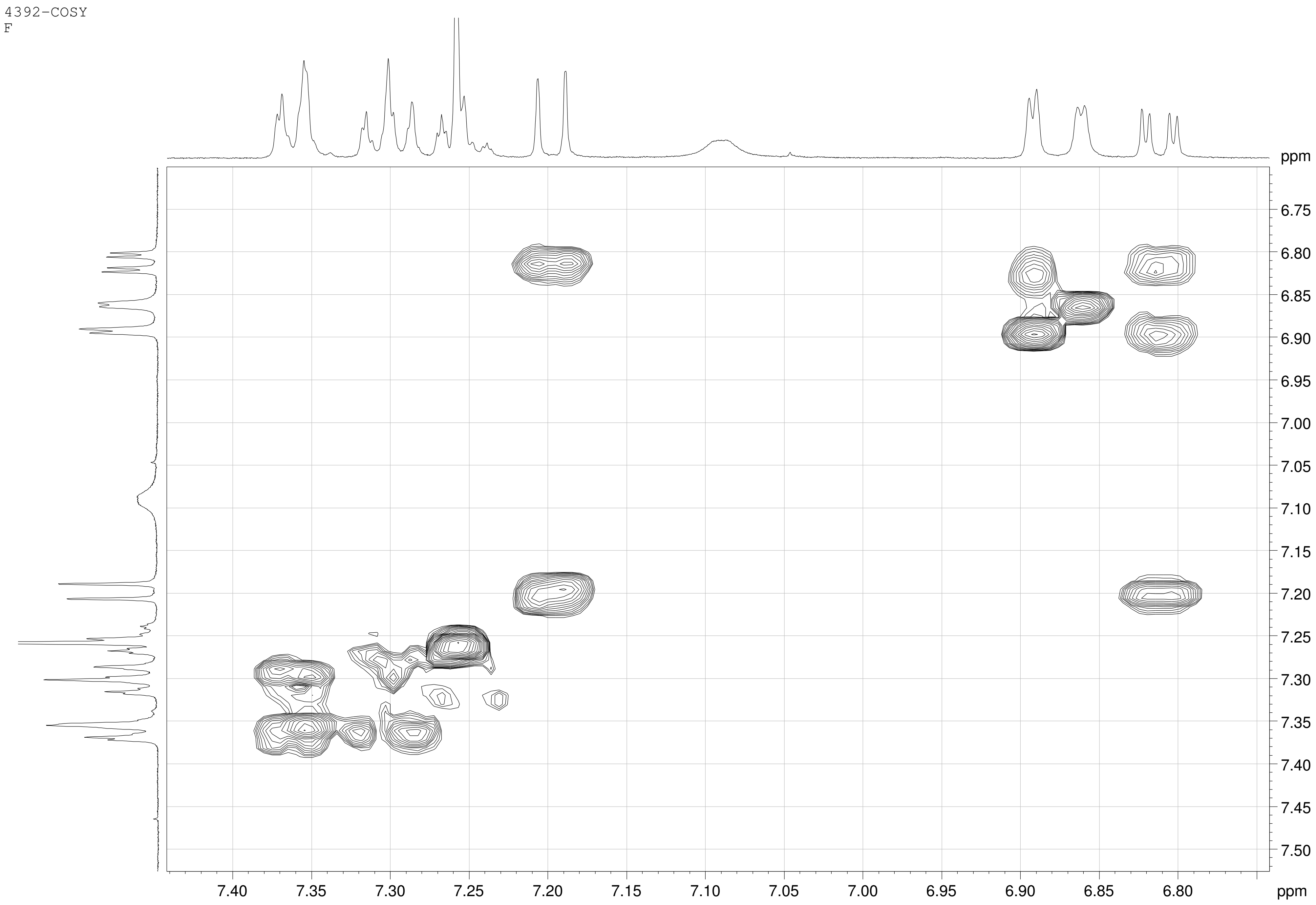
4392-COSY
F
ppm
6.75
6.80
6.85
6.90
6.95
7.00
7.05
7.10
7.15
7.20
7.25
7.30
7.35
7.40
7.45
7.50
7.40
7.35
7.30
7.25
7.20
7.15
7.10
7.05
7.00
6.95
6.90
6.85
6.80
ppm

4392-COSY
F

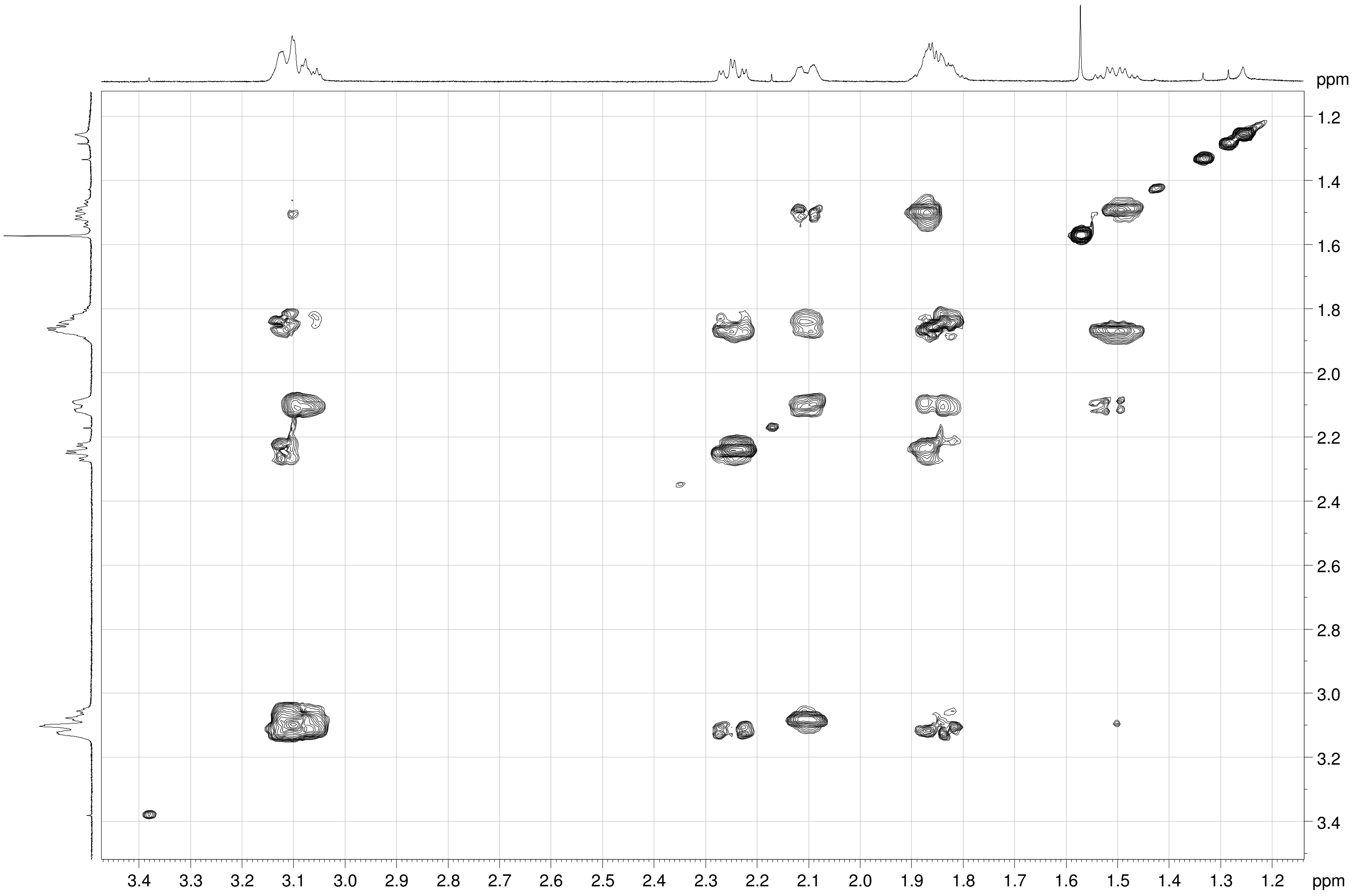

ppm
1.2
1.4
1.6
1.8
2.0
2.2
2.4
2.6
2.8
3.0
3.2
3.4
3.4 3.3 3.2 3.1 3.0 2.9 2.8 2.7 2.6 2.5 2.4 2.3 2.2 2.1 2.0 1.9 1.8 1.7 1.6 1.5 1.4 1.3 1.2
ppm

4502-HSQC
F

Current Data Parameters
NAME 4502
EXPNO 3
PROCNO 1

F2 - Acquisition Parameters
Date_ 20180124
PROBHD 5 mm CPPBBO BB
PULPROG hsqcedetgpsisp2.3
SOLVENT CDCl3
TE 298.2 K
SWH 5411.255 Hz
AQ 0.1892352 sec
NS 1
FIDRES 2.642215 Hz
D0 0.00000300 sec
D1 2.00000000 sec
D4 0.00172414 sec
D11 0.03000000 sec
D16 0.00020000 sec
D21 0.00360000 sec
D24 0.00089000 sec

======== CHANNEL f1 ========
SFO1 500.2021794 MHz
NUC1 1H
P1 11.30 usec
P2 22.60 usec
P28 0 usec
PLW1 13.69999981 W

======== CHANNEL f2 ========
SFO2 125.7881799 MHz
NUC2 13C
P3 10.00 usec
P14 500.00 usec
P24 2000.00 usec
P31 1900.00 usec
PLW0 0 W
PLW2 55.00000000 W
PLW12 1.30180001 W

====== GRADIENT CHANNEL =====
P16 1000.00 usec
P19 600.00 usec

F1 - Acquisition parameters
TD 128
SFO1 125.7882 MHz
FIDRES 188.707733 Hz
SW 192.026 ppm
FnMODE Echo-Antiecho

F2 - Processing parameters

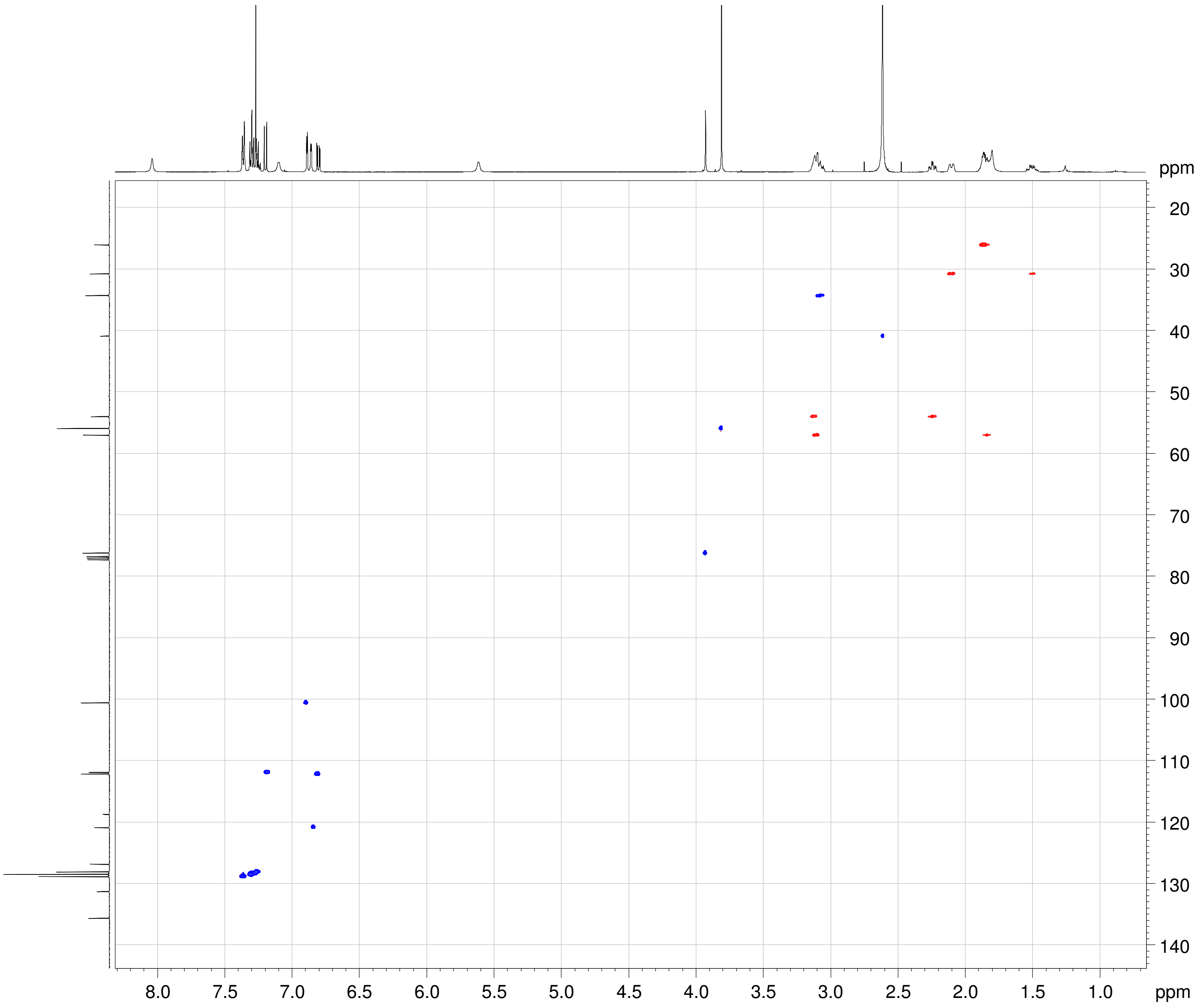

4502-HSQC
F

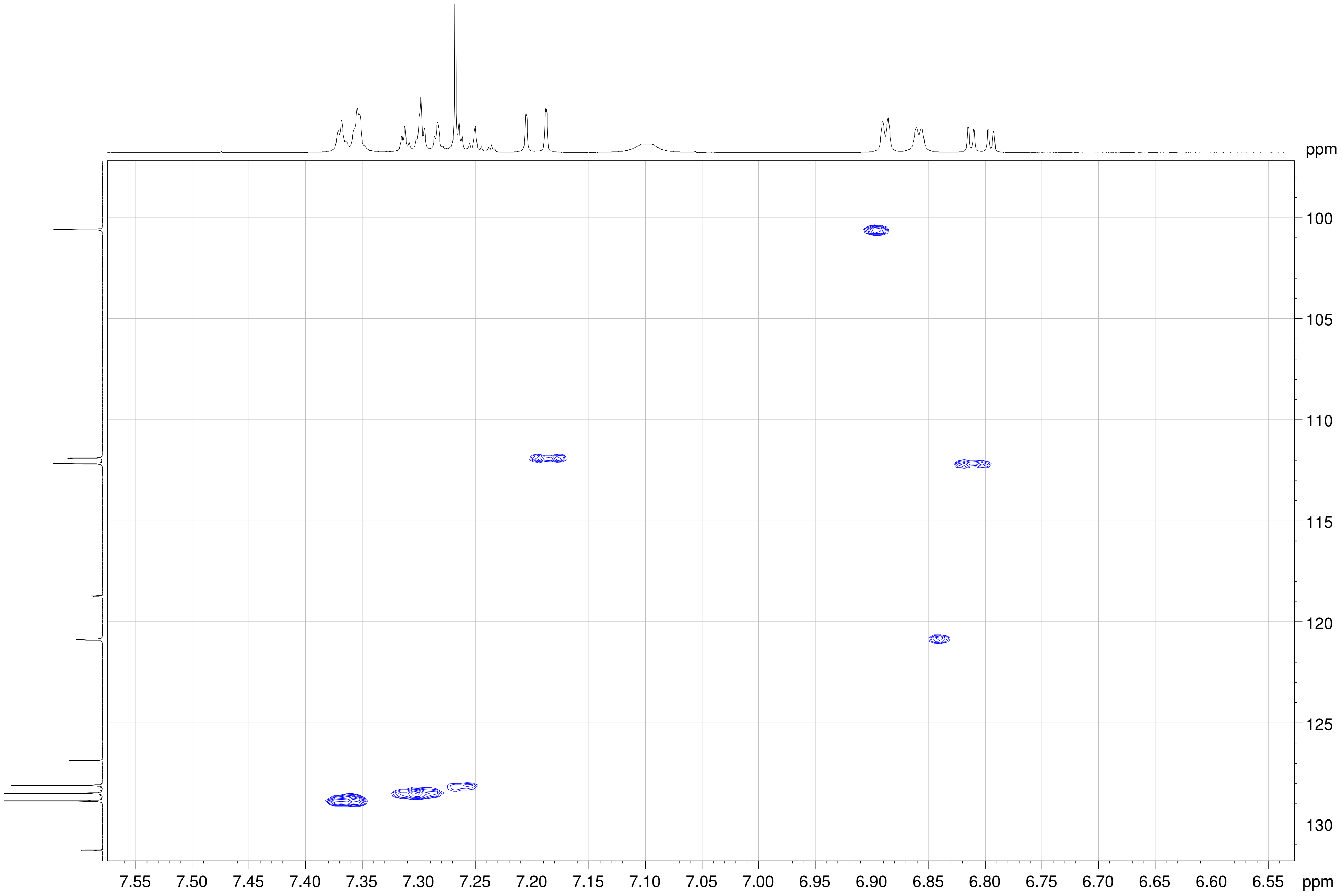

ppm
100
105
110
115
120
125
130
7.55 7.50 7.45 7.40 7.35 7.30 7.25 7.20 7.15 7.10 7.05 7.00 6.95 6.90 6.85 6.80 6.75 6.70 6.65 6.60 6.55
ppm

4502-HSQC
F

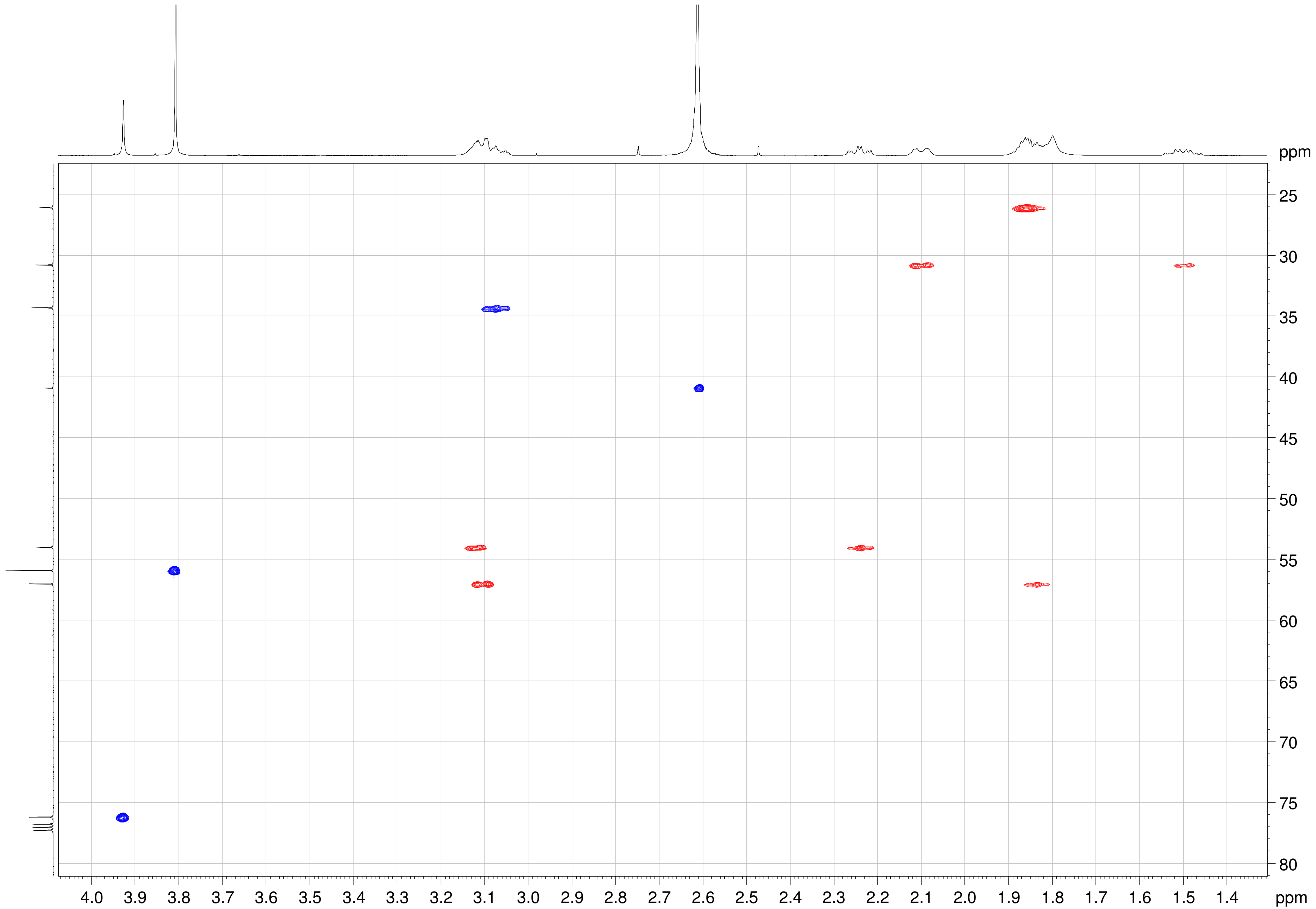
ppm
25
30
35
40
45
50
55
60
65
70
75
80
4.0 3.9 3.8 3.7 3.6 3.5 3.4 3.3 3.2 3.1 3.0 2.9 2.8 2.7 2.6 2.5 2.4 2.3 2.2 2.1 2.0 1.9 1.8 1.7 1.6 1.5 1.4
ppm

(R)-10a

3819-1H
(-)A x HCl

Current Data Parameters
NAME 3819
EXPNO 1
PROCNO 1

F2 - Acquisition Parameters
Date_ 20170531
PROBHD 5 mm PABBI 1H/
PULPROG zg30
SOLVENT D2O
TE 298.1 K
SWH 10000.000 Hz
AQ 3.2767999 sec
NS 8
FIDRES 0.152588 Hz
D1 0 sec

======== CHANNEL f1 =======
SFO1 500.2030889 MHz
NUC1 1H
P1 8.00 use
PLW1 12.69999981 W

F2 - Processing parameters
SI 65536
LB 0 Hz

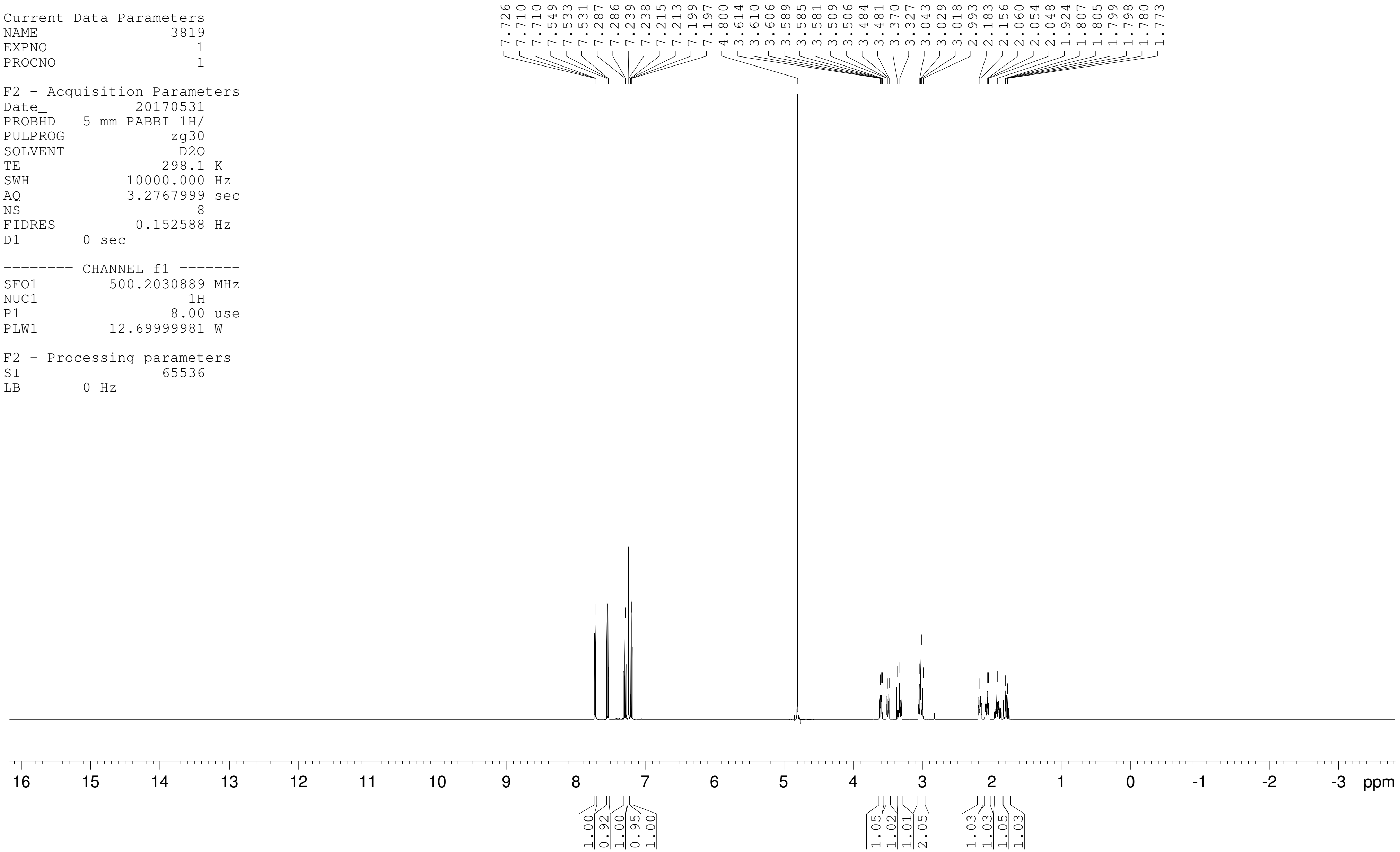

3819-1H
(-)A x HCl

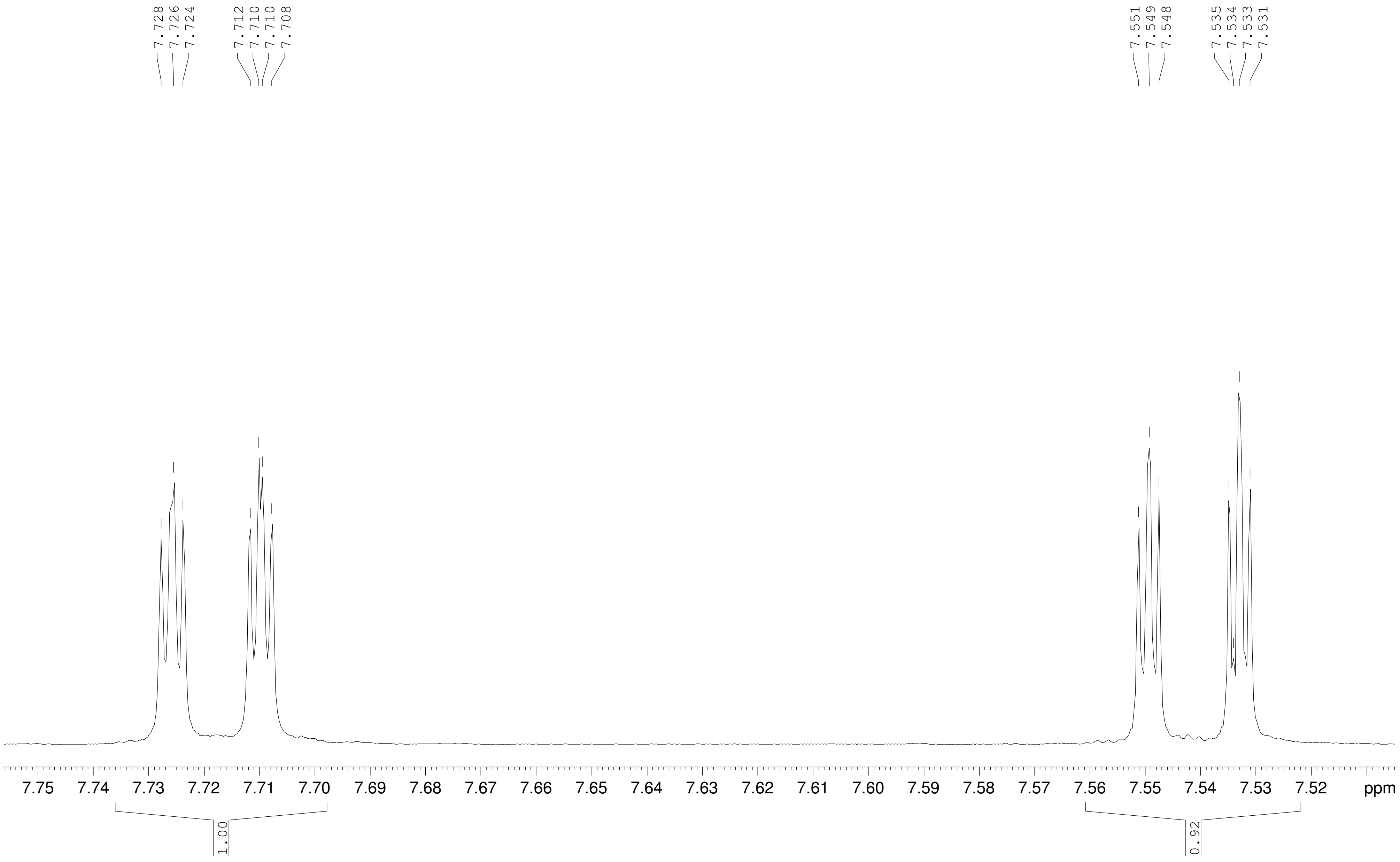

3819-1H
(-)A x HCl

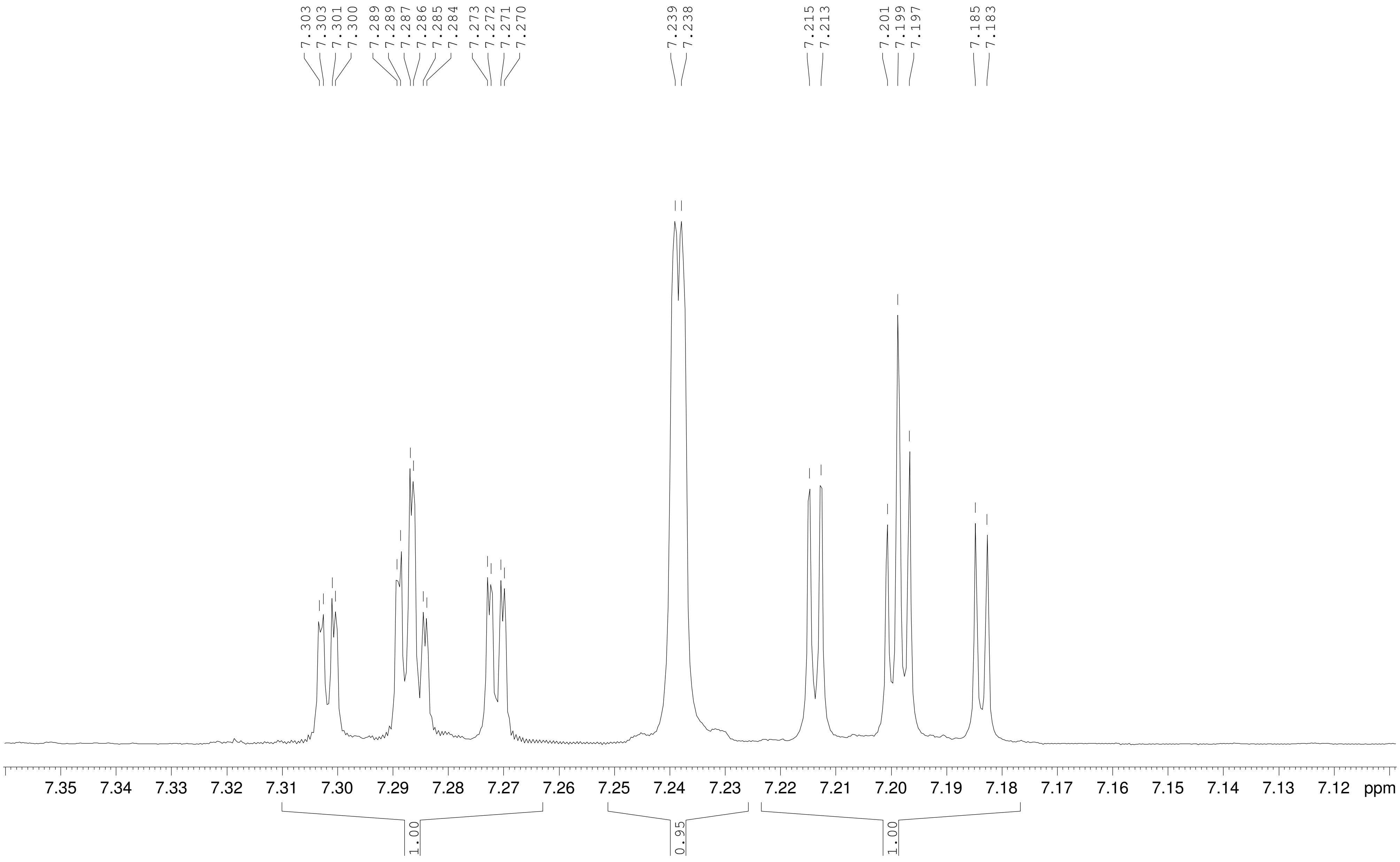

3819-1H
(-)A x HCl

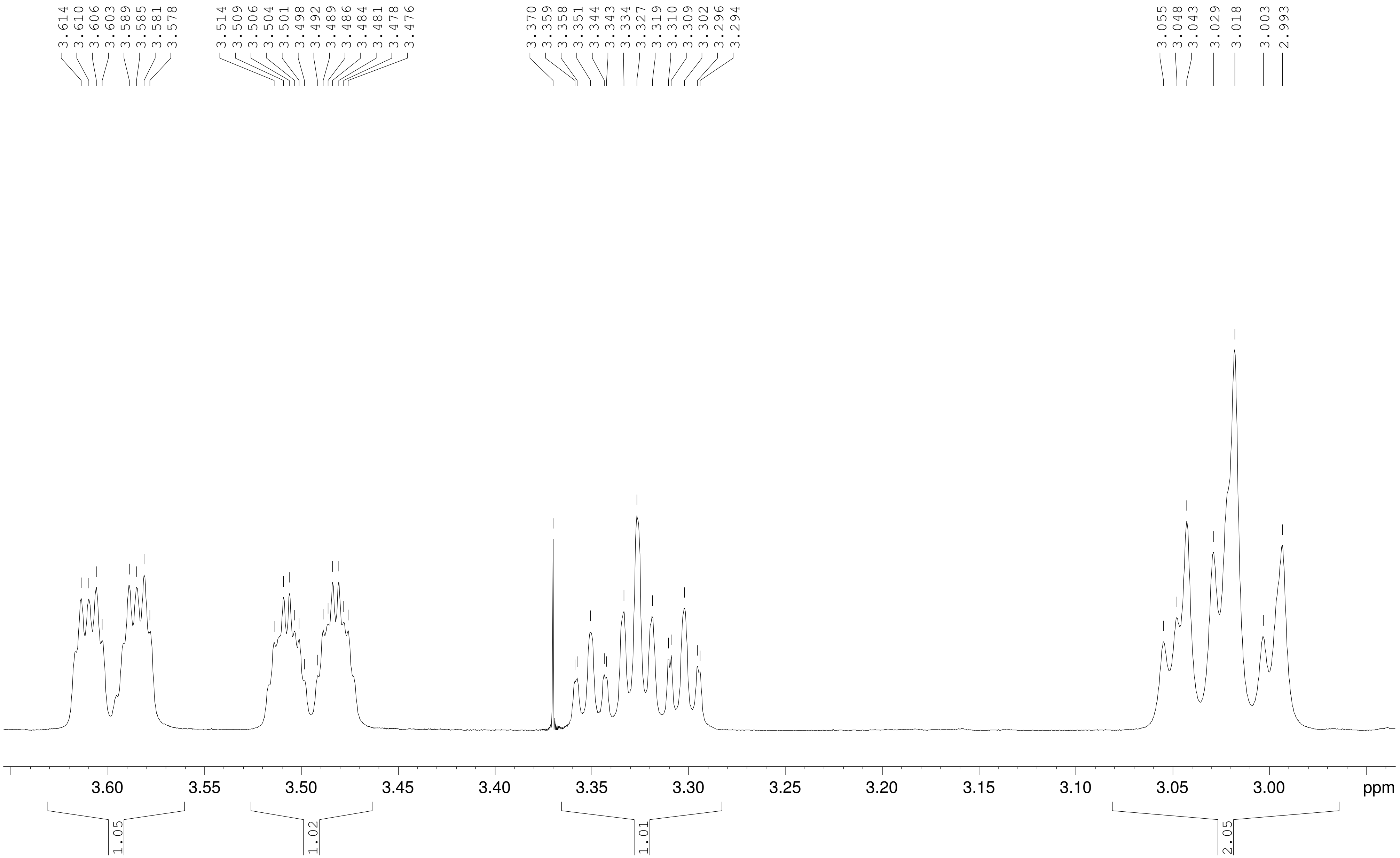

3819-1H
(-)A x HCl

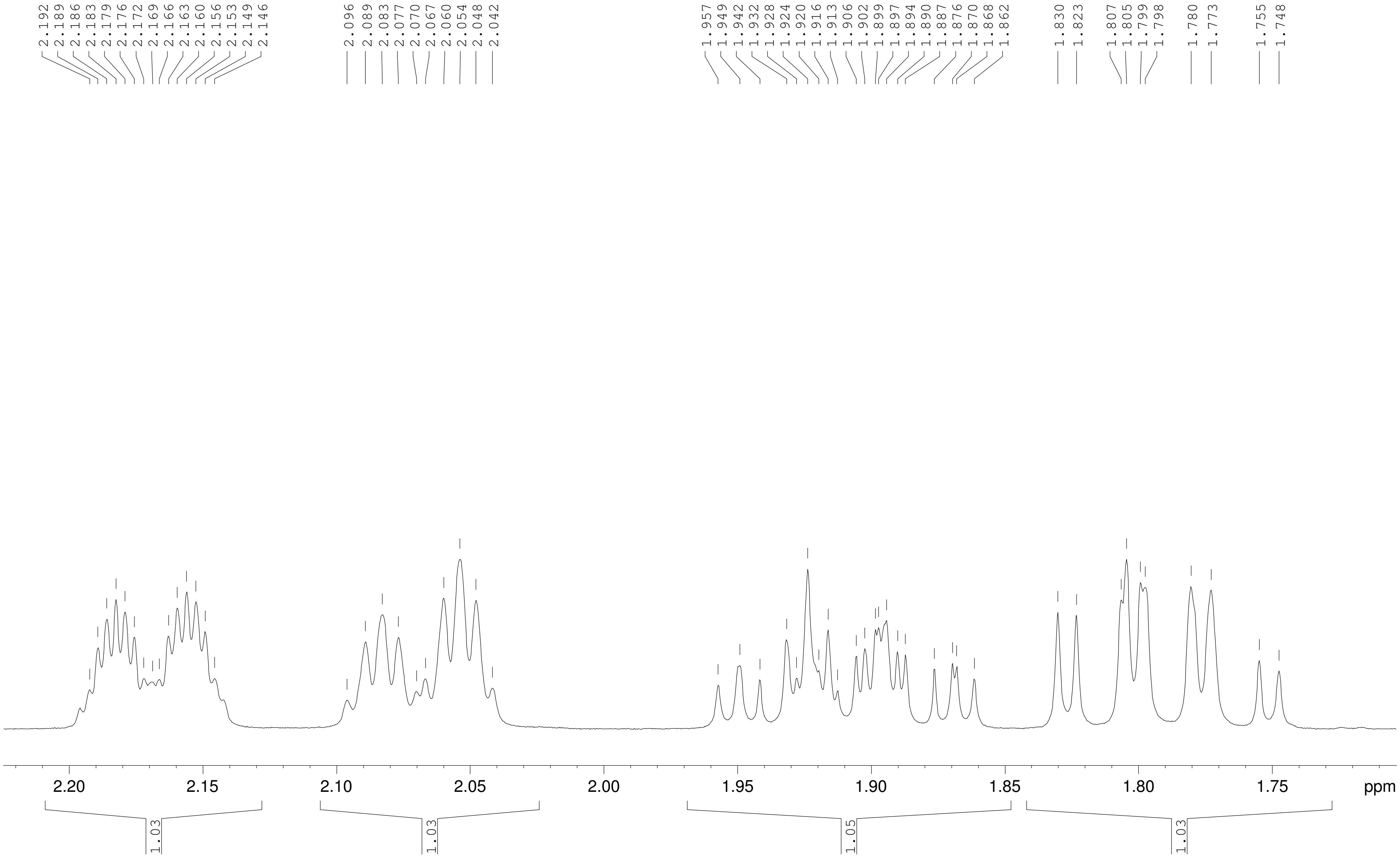

(S)-11a

[α] = + 0.03° B(+) [illegible] (R)

10 mg / 1 mL MeOH

3704-1H
I

Current Data Parameters
NAME 3704
EXPNO 11
PROCNO 1

F2 - Acquisition Parameters
Date_ 20170426
PROBHD 5 mm PABBI 1H/
PULPROG zg30
SOLVENT D2O
TE 298.2 K
SWH 10000.000 Hz
AQ 3.2767999 sec
NS 8
FIDRES 0.152588 Hz
D1 0 sec

======== CHANNEL f1 =======
SFO1 500.2030889 MHz
NUC1 1H
P1 8.00 use
PLW1 12.69999981 W

F2 - Processing parameters
SI 65536
LB 0 Hz

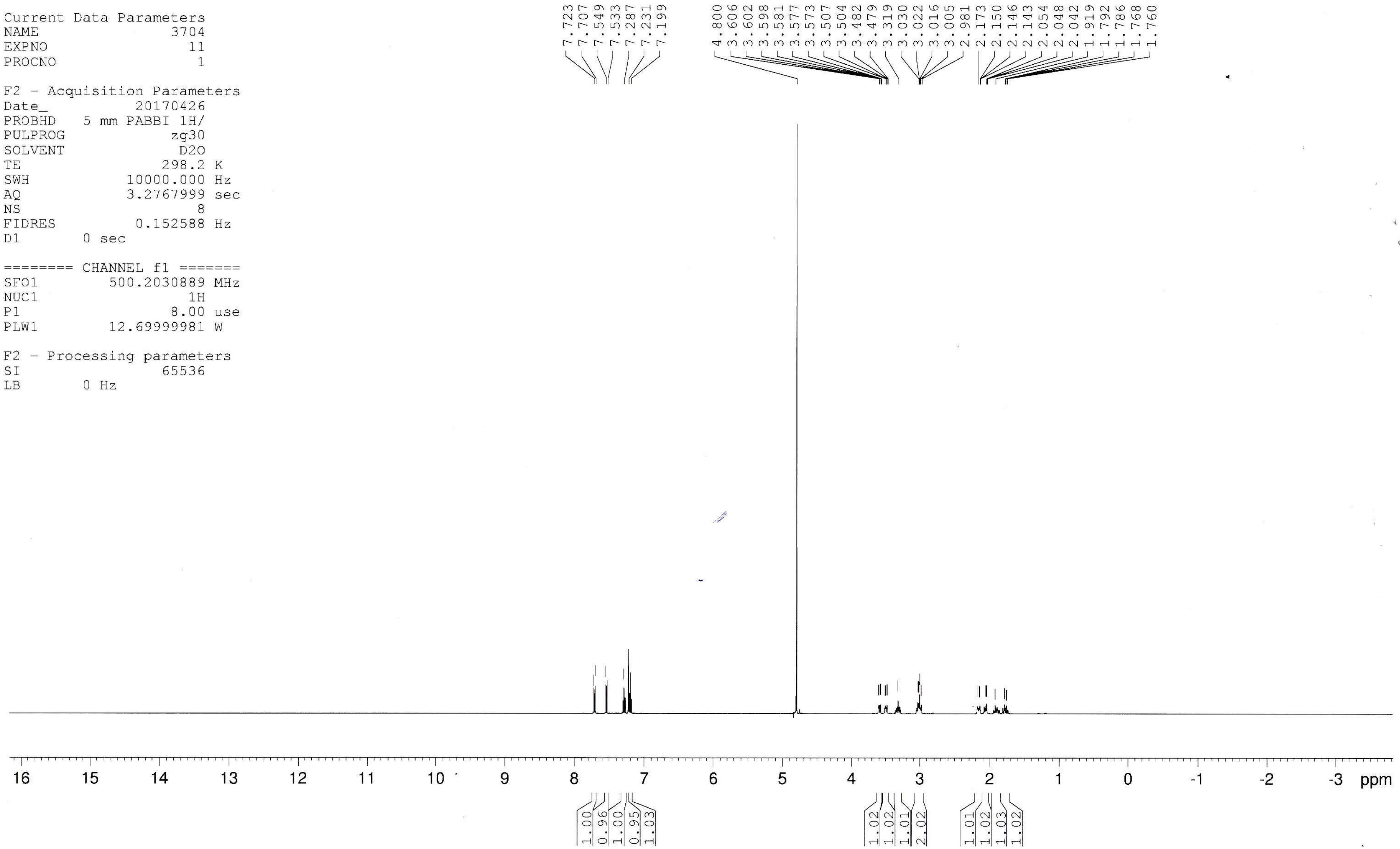

3704-1H
I

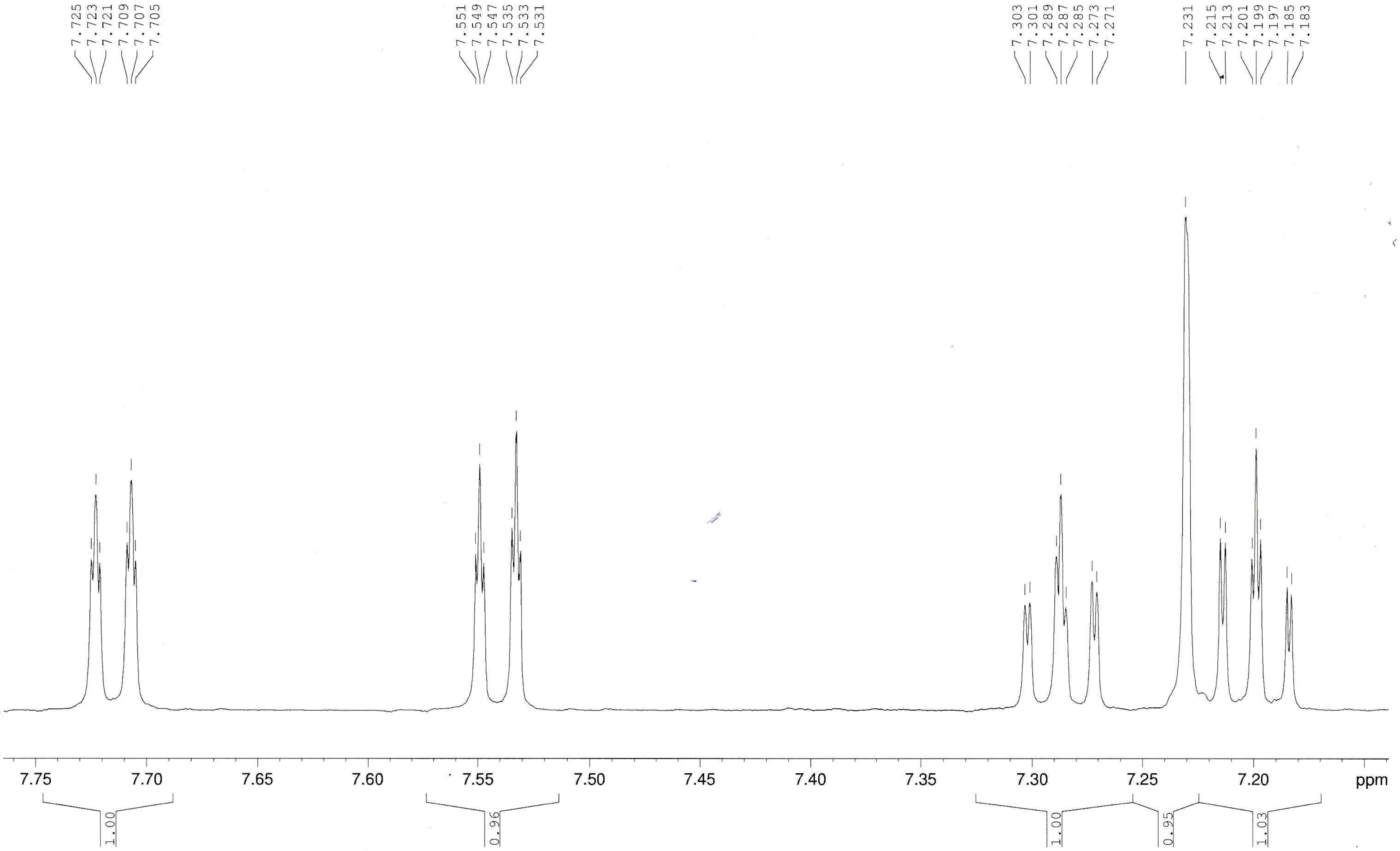

7.725
7.723
7.721
7.709
7.707
7.705
7.551
7.549
7.547
7.535
7.533
7.531
7.303
7.301
7.289
7.287
7.285
7.273
7.271
7.231
7.215
7.213
7.201
7.199
7.197
7.185
7.183
7.75
7.70
7.65
7.60
7.55
7.50
7.45
7.40
7.35
7.30
7.25
7.20
ppm
1.00
0.96
1.00
0.95
1.03

3704-1H
I

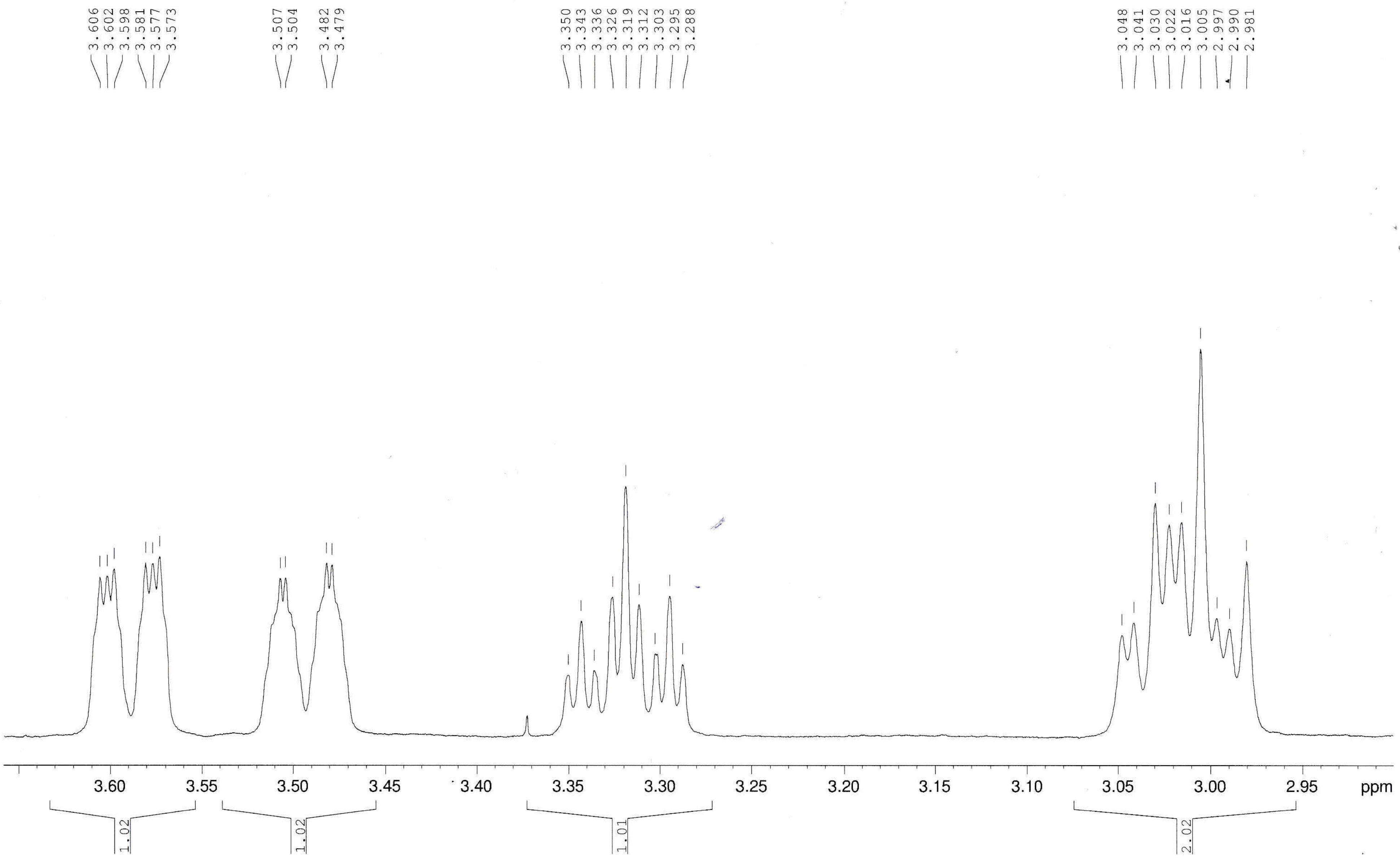

3.606
3.602
3.598
3.581
3.577
3.573
3.507
3.504
3.482
3.479
3.350
3.343
3.336
3.326
3.319
3.312
3.303
3.295
3.288
3.048
3.041
3.030
3.022
3.016
3.005
2.997
2.990
2.981
3.60 3.55 3.50 3.45 3.40 3.35 3.30 3.25 3.20 3.15 3.10 3.05 3.00 2.95 ppm
1.02
1.02
1.01
2.02

3704-1H
I

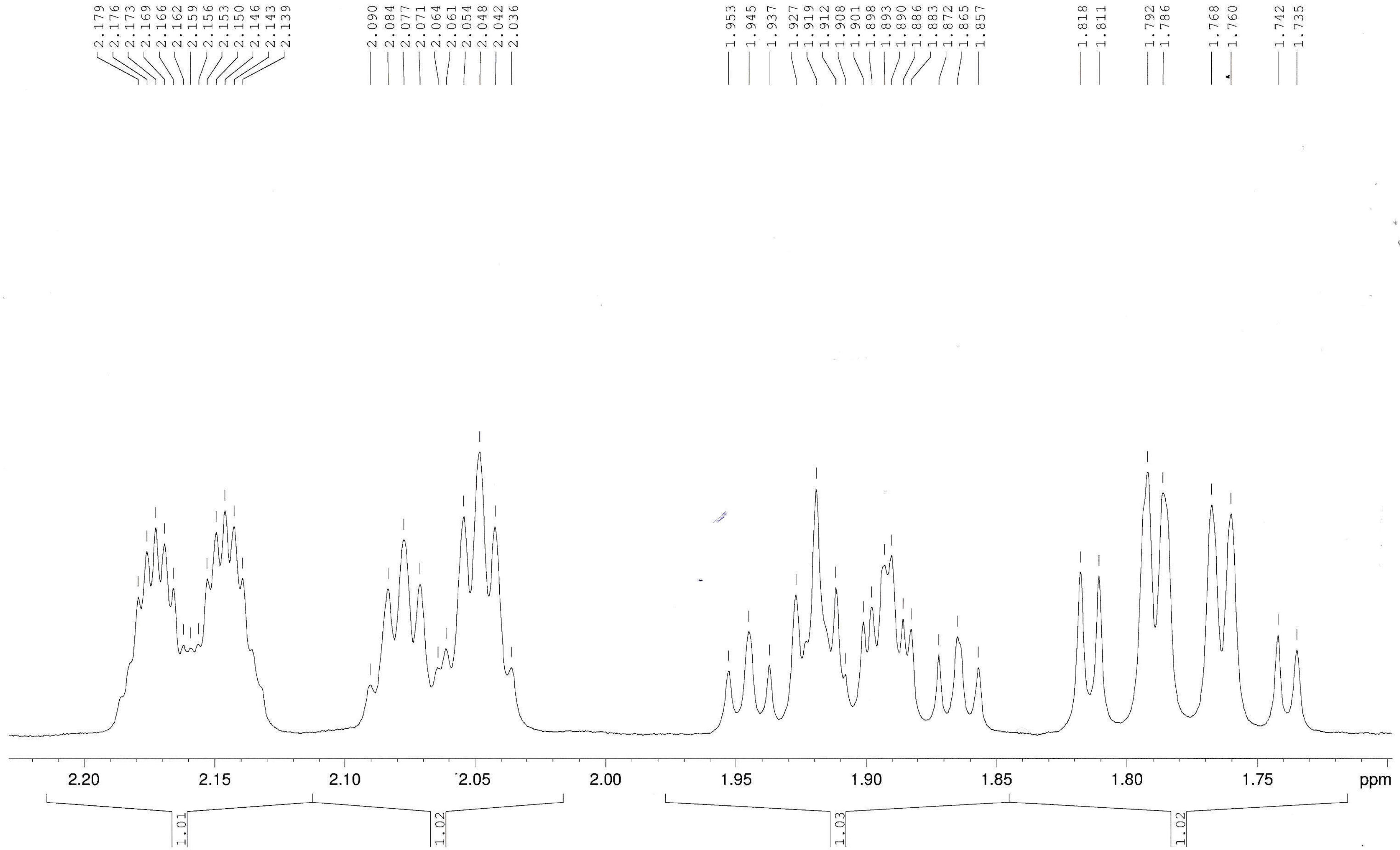

2.179
2.176
2.173
2.169
2.166
2.162
2.159
2.156
2.153
2.150
2.146
2.143
2.139
2.090
2.084
2.077
2.071
2.064
2.061
2.054
2.048
2.042
2.036
1.953
1.945
1.937
1.927
1.919
1.912
1.908
1.901
1.898
1.893
1.890
1.886
1.883
1.872
1.865
1.857
1.818
1.811
1.792
1.786
1.768
1.760
1.742
1.735
2.20
2.15
2.10
2.05
2.00
1.95
1.90
1.85
1.80
1.75
ppm
1.01
1.02
1.03
1.02

3704-13C
I

Current Data Parameters
NAME 3704
EXPNO 12
PROCNO 1

F2 - Acquisition Parameters
Date_ 20170426
PROBHD 5 mm PABBI 1H/
PULPROG zg_pi_CPD
SOLVENT D2O
TE 298.2 K
SWH 31249.381 Hz
AQ 1.0485969 sec
NS 280
FIDRES 0.476828 Hz
D1 1.50000000 sec
D11 0.03000000 sec
D14 0.00050000 sec

======== CHANNEL f1 ========
SFO1 125.7904831 MHz
NUC1 13C
P1 15.00 usec
P2 30.00 usec
PLW1 78.52400208 W

======== CHANNEL f2 ========
SFO2 500.2020008 MHz
NUC2 1H
PLW2 12.50000000 W
PLW12 0.12500000 W

F2 - Processing parameters
SI 65536
LB 0.50 Hz

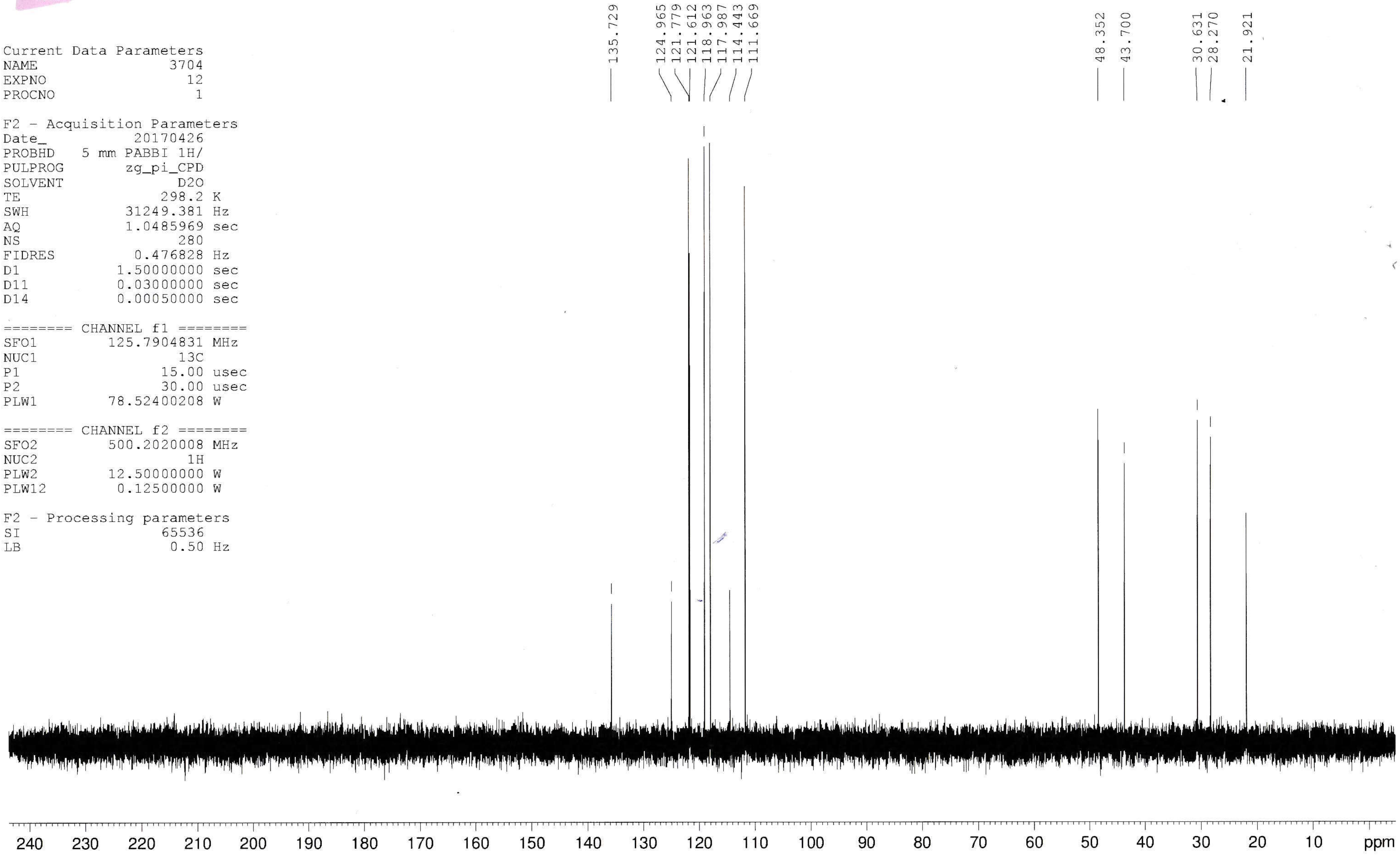

3704-13C
I

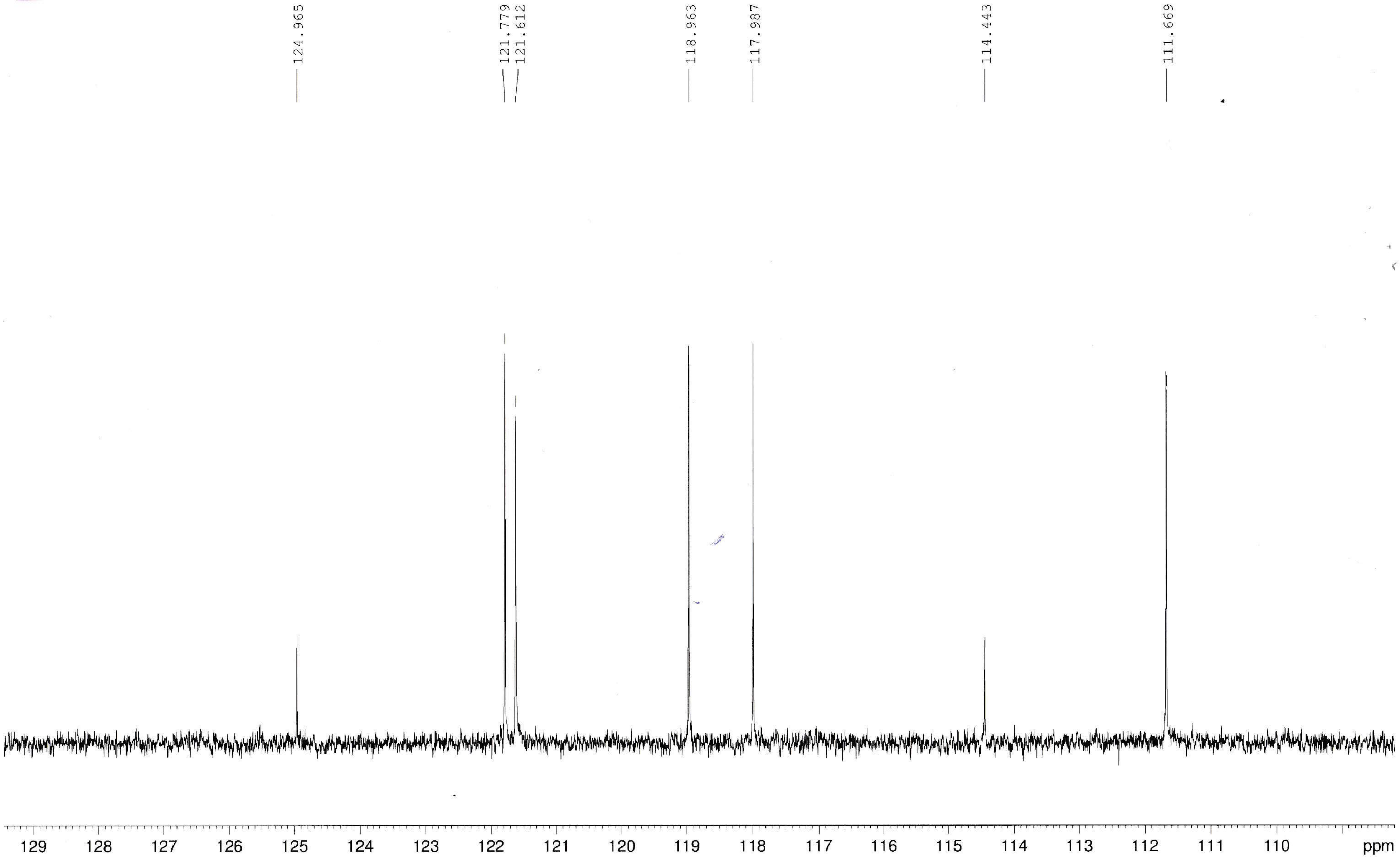

124.965
121.779
121.612
118.963
117.987
114.443
111.669
129
128
127
126
125
124
123
122
121
120
119
118
117
116
115
114
113
112
111
110
ppm

(R)-10b

4125-1H
CxHCl

Current Data Parameters
NAME 4125
EXPNO 1
PROCNO 1

F2 - Acquisition Parameters
Date_ 20170926
PROBHD 5 mm CPPBBO BB
PULPROG zg30
SOLVENT D2O
TE 298.2 K
SWH 10000.000 Hz
AQ 3.2767999 sec
NS 8
FIDRES 0.152588 Hz
D1 0 sec

======== CHANNEL f1 ========
SFO1 500.2030889 MHz
NUC1 1H
P1 11.30 usec
PLW1 13.69999981 W

F2 - Processing parameters
SI 65536
LB 0 Hz

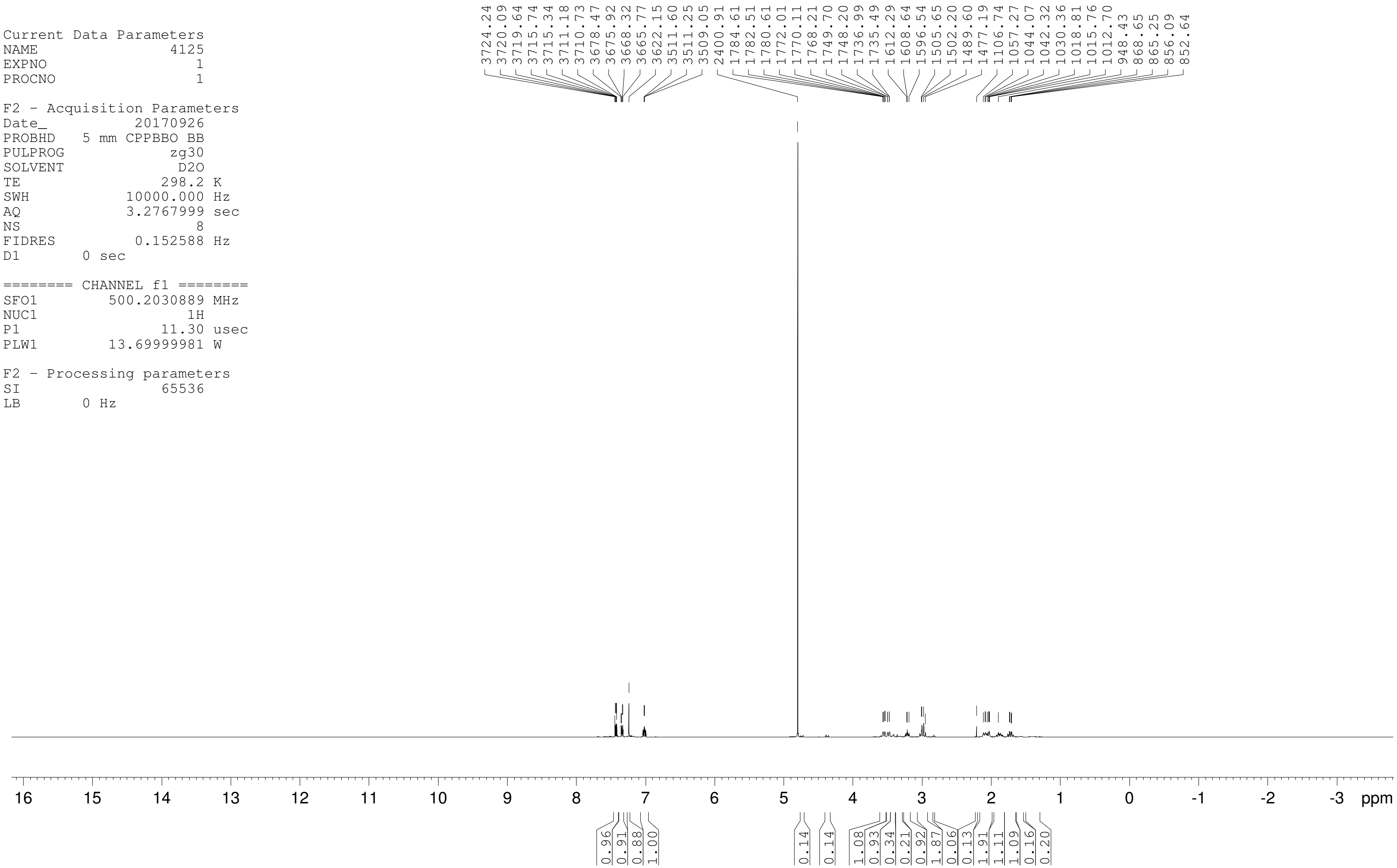

4125-1H
CxHCl

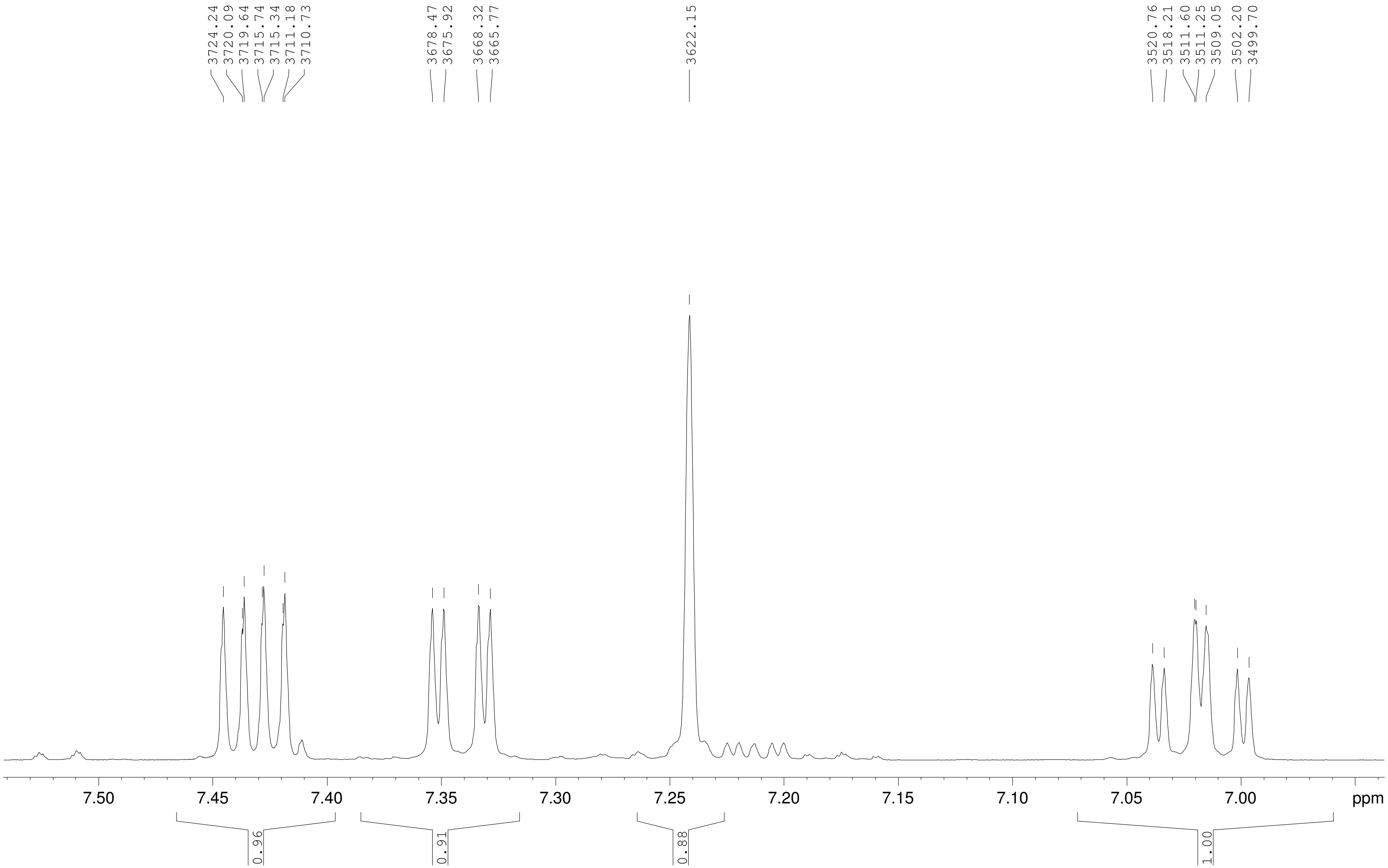

4125-1H
CxHCl

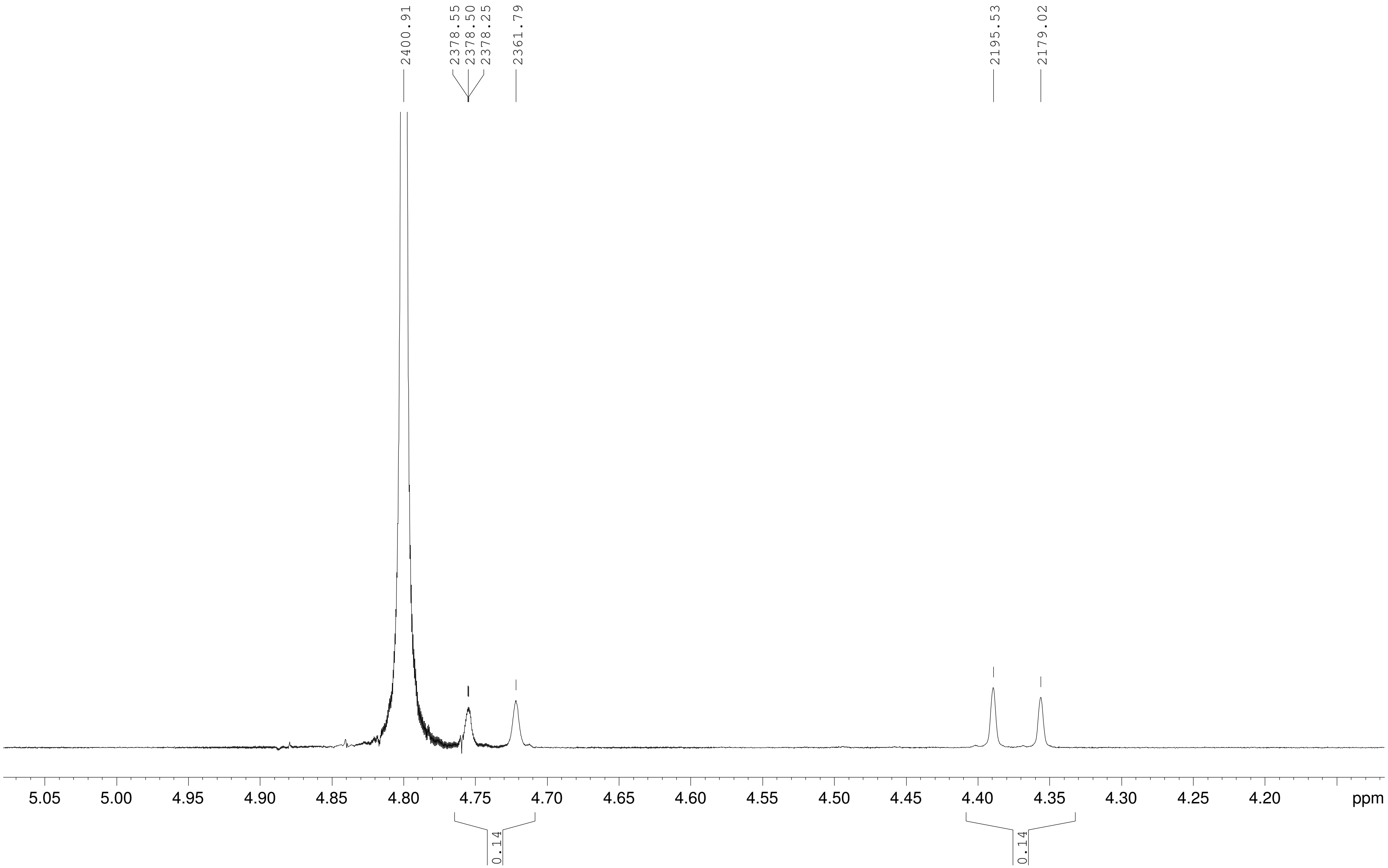

4125-1H
CxHCl

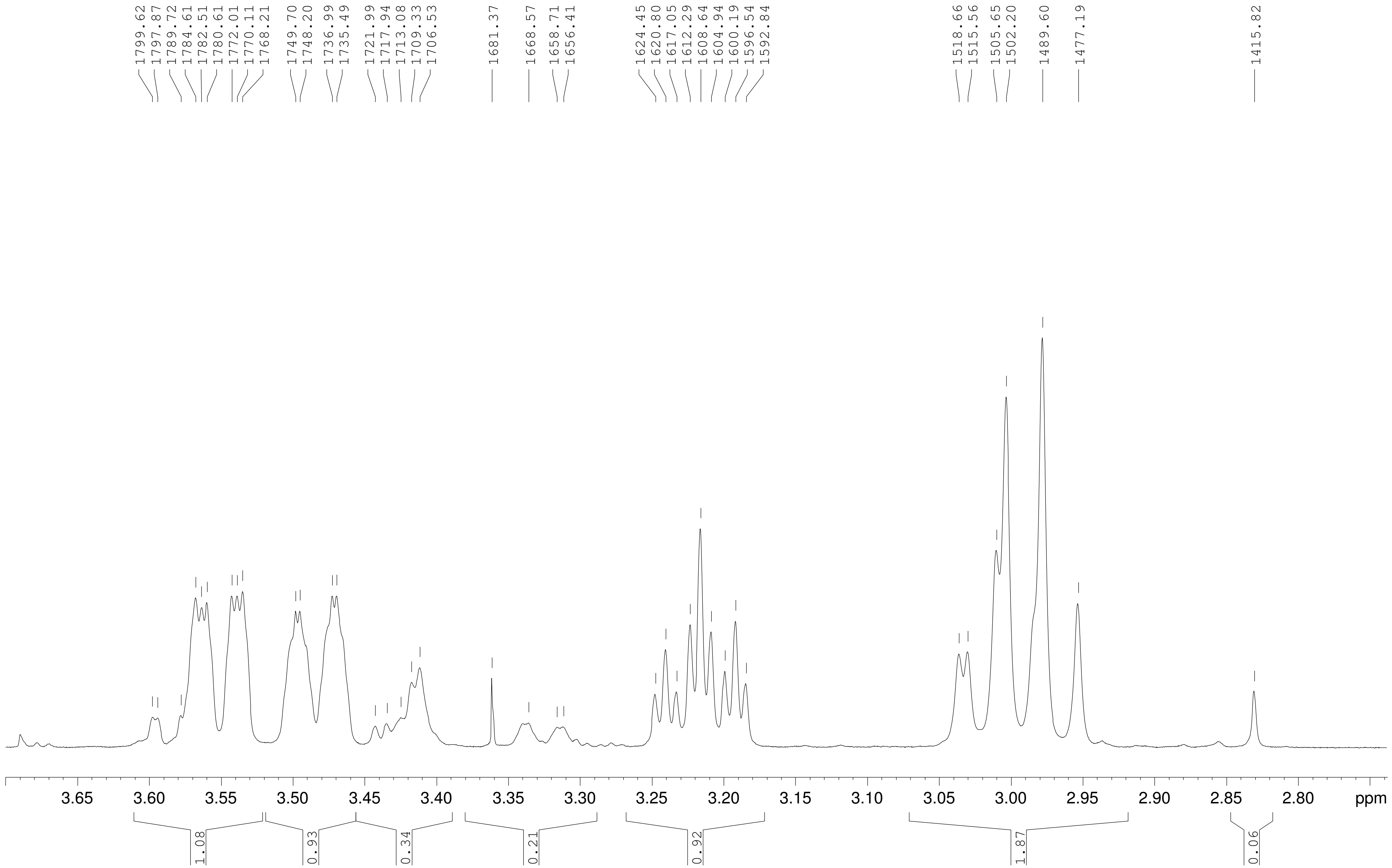

4125-1H
CxHCl

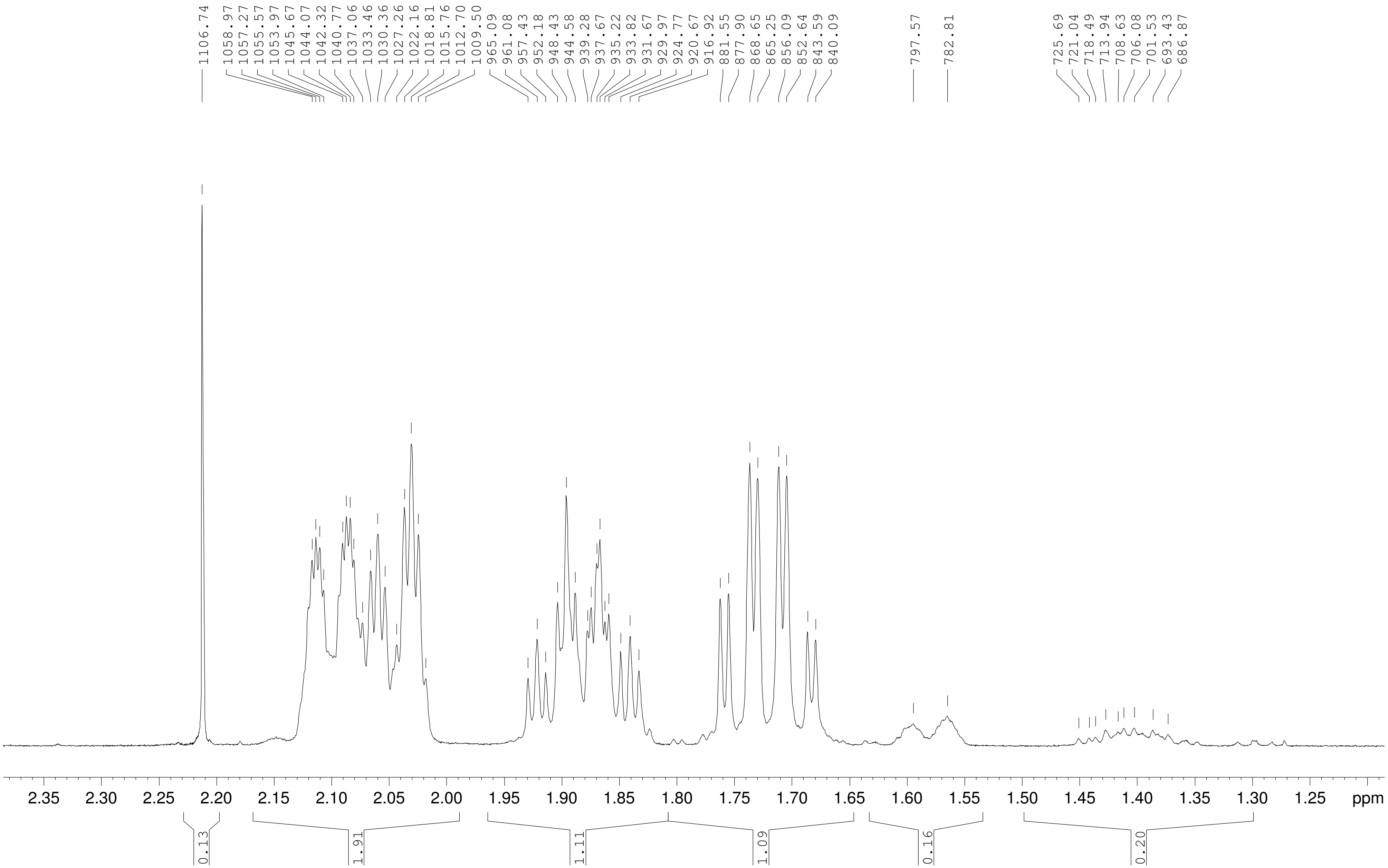

(S)-11b

4008-1H
A3

Current Data Parameters
NAME 4008
EXPNO 1
PROCNO 1

F2 - Acquisition Parameters
Date_ 20170727
PROBHD 5 mm PABBI 1H/
PULPROG zg30
SOLVENT D2O
TE 298.2 K
SWH 10000.000 Hz
AQ 3.2767999 sec
NS 8
FIDRES 0.152588 Hz
D1 0 sec

======== CHANNEL f1 =======
SFO1 500.2030889 MHz
NUC1 1H
P1 8.00 use
PLW1 12.69999981 W

F2 - Processing parameters
SI 65536
LB 0 Hz

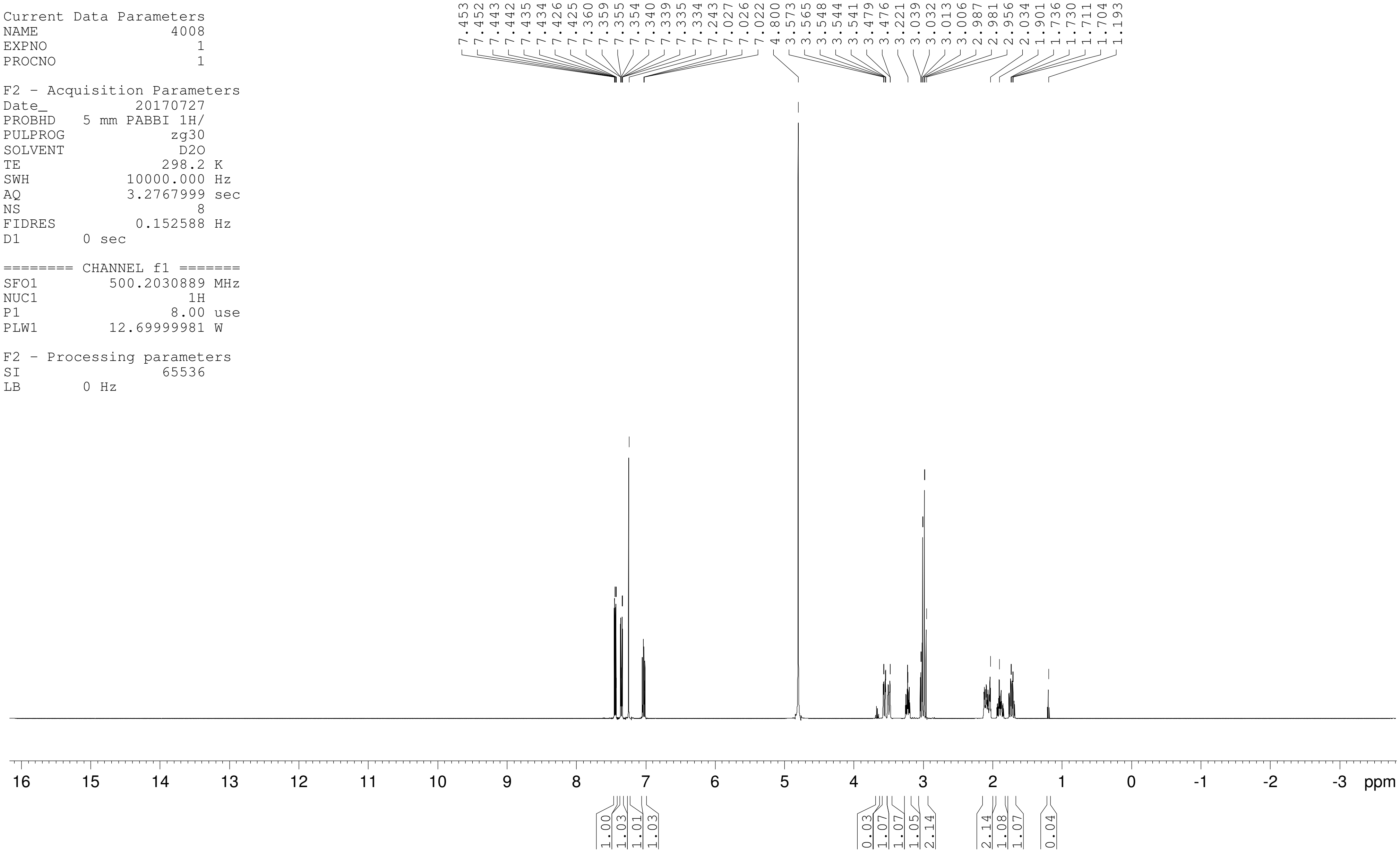

4008-1H
A3

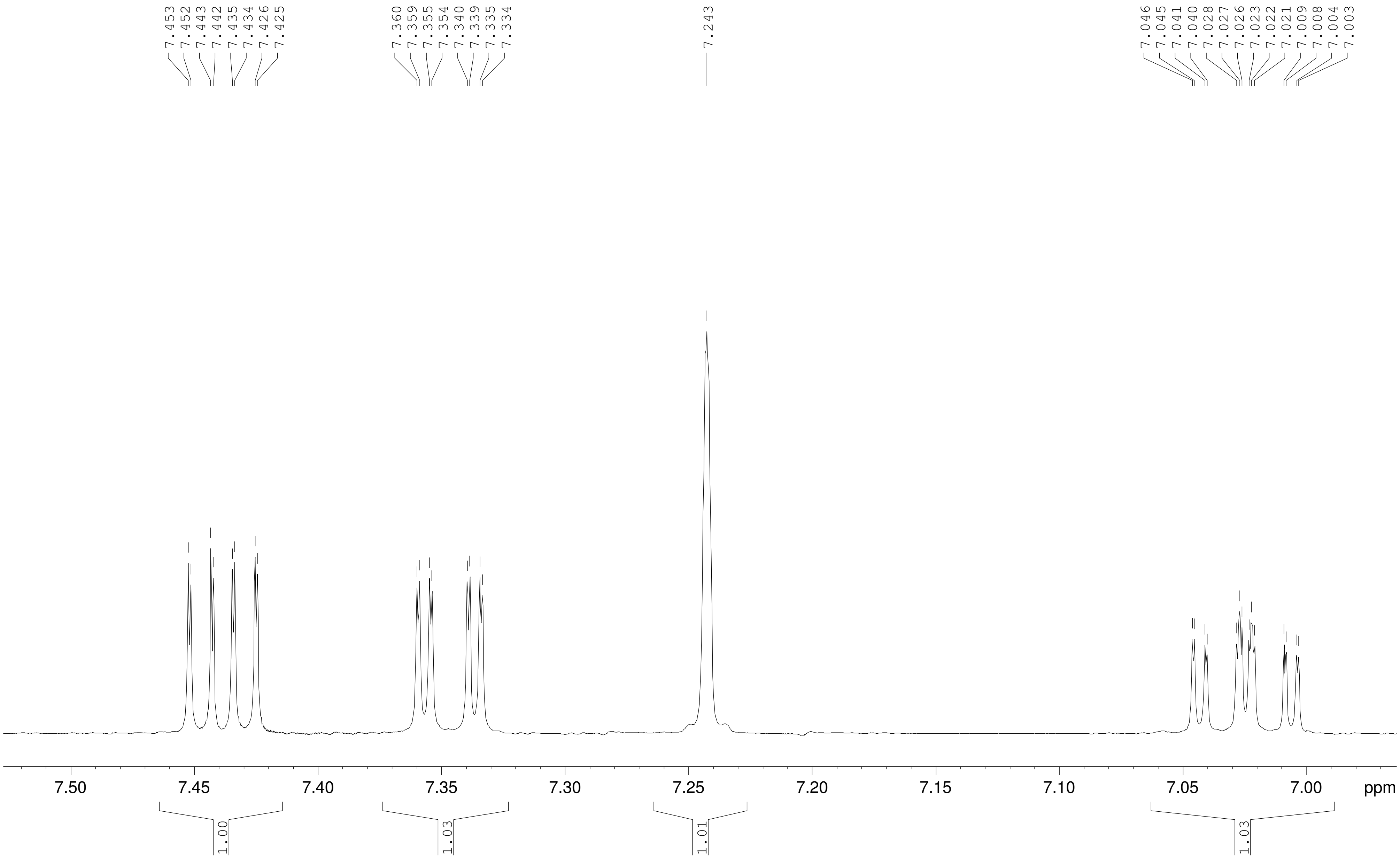
7.453
7.452
7.443
7.442
7.435
7.434
7.426
7.425
7.360
7.359
7.355
7.354
7.340
7.339
7.335
7.334
7.243
7.046
7.045
7.041
7.040
7.028
7.027
7.026
7.023
7.022
7.021
7.009
7.008
7.004
7.003
7.50
7.45
7.40
7.35
7.30
7.25
7.20
7.15
7.10
7.05
7.00
ppm
1.00
1.03
1.01
1.03

4008-1H
A3

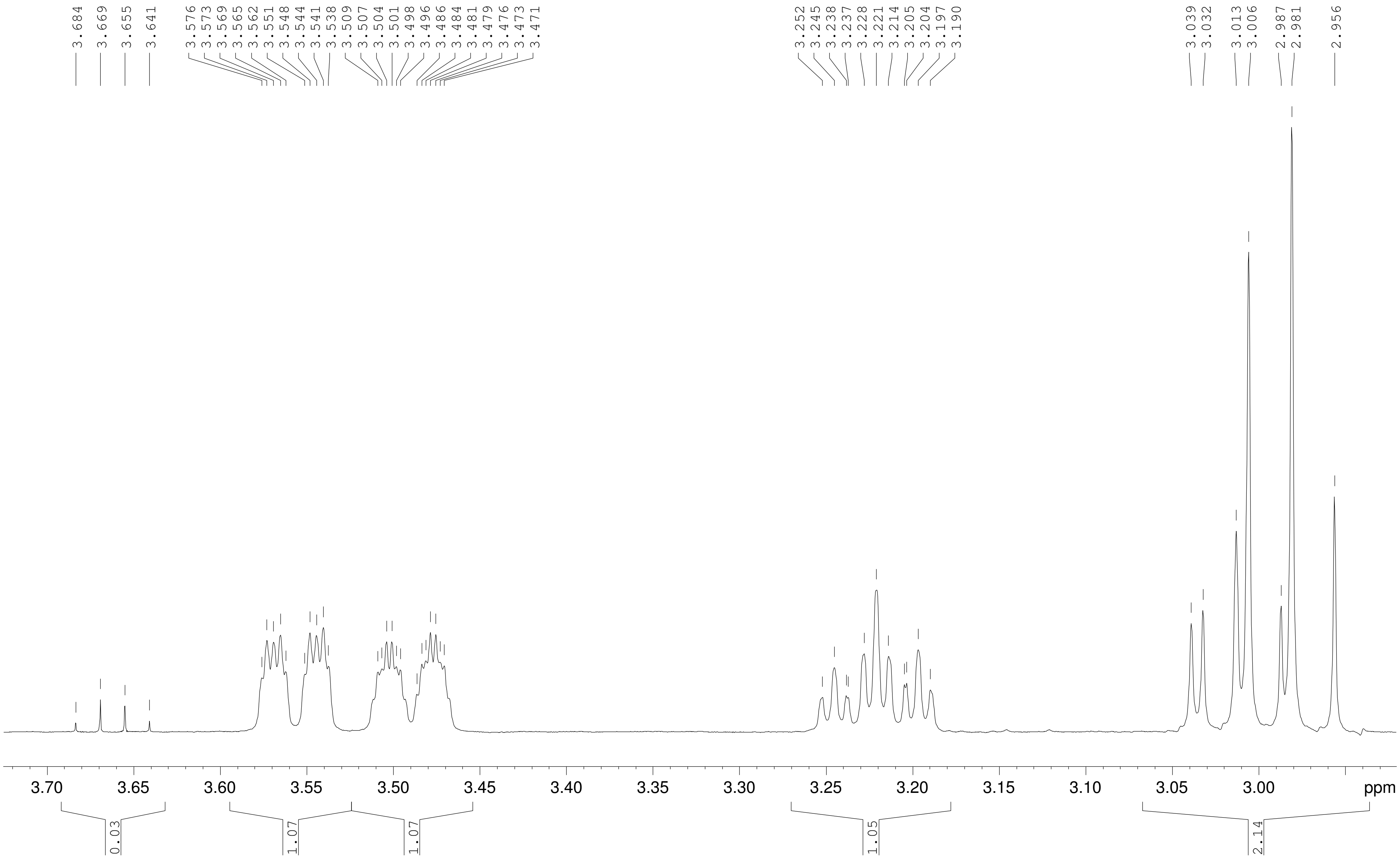
3.684
3.669
3.655
3.641
3.576
3.573
3.569
3.565
3.562
3.551
3.548
3.544
3.541
3.538
3.509
3.507
3.504
3.501
3.498
3.496
3.486
3.484
3.481
3.479
3.476
3.473
3.471
3.252
3.245
3.238
3.237
3.228
3.221
3.214
3.205
3.204
3.197
3.190
3.039
3.032
3.013
3.006
2.987
2.981
2.956
3.70 3.65 3.60 3.55 3.50 3.45 3.40 3.35 3.30 3.25 3.20 3.15 3.10 3.05 3.00 ppm
0.03
1.07
1.07
1.05
2.14

4008-1H
A3

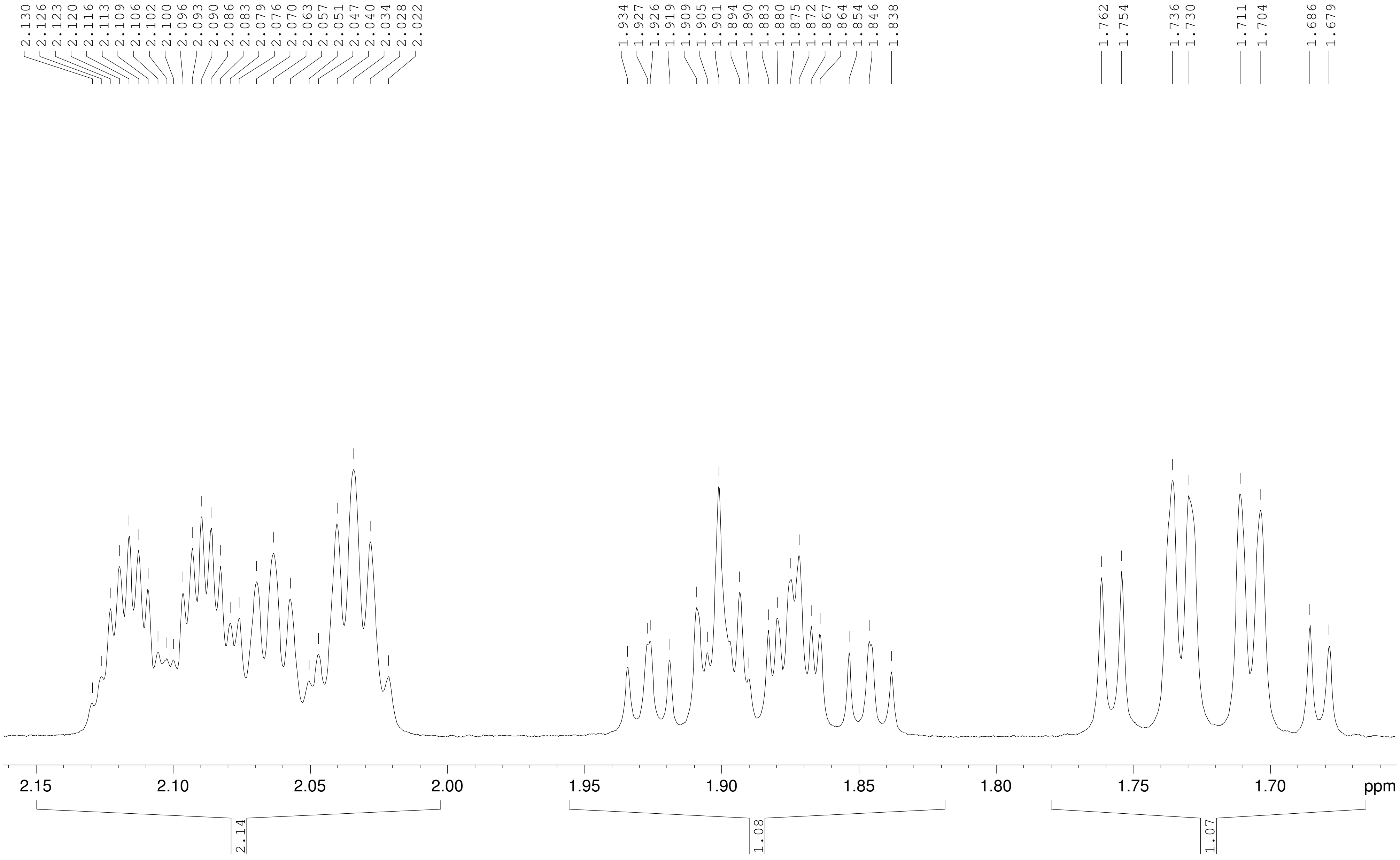
2.130 2.126 2.123 2.120 2.116 2.113 2.109 2.106 2.102 2.100 2.096 2.093 2.090 2.086 2.083 2.079 2.076 2.070 2.063 2.057 2.051 2.047 2.040 2.034 2.028 2.022
1.934 1.927 1.926 1.919 1.909 1.905 1.901 1.894 1.890 1.883 1.880 1.875 1.872 1.867 1.864 1.854 1.846 1.838
1.762 1.754 1.736 1.730 1.711 1.704 1.686 1.679
2.15 2.10 2.05 2.00 1.95 1.90 1.85 1.80 1.75 1.70 ppm
2.14
1.08
1.07

4008-1H
A3

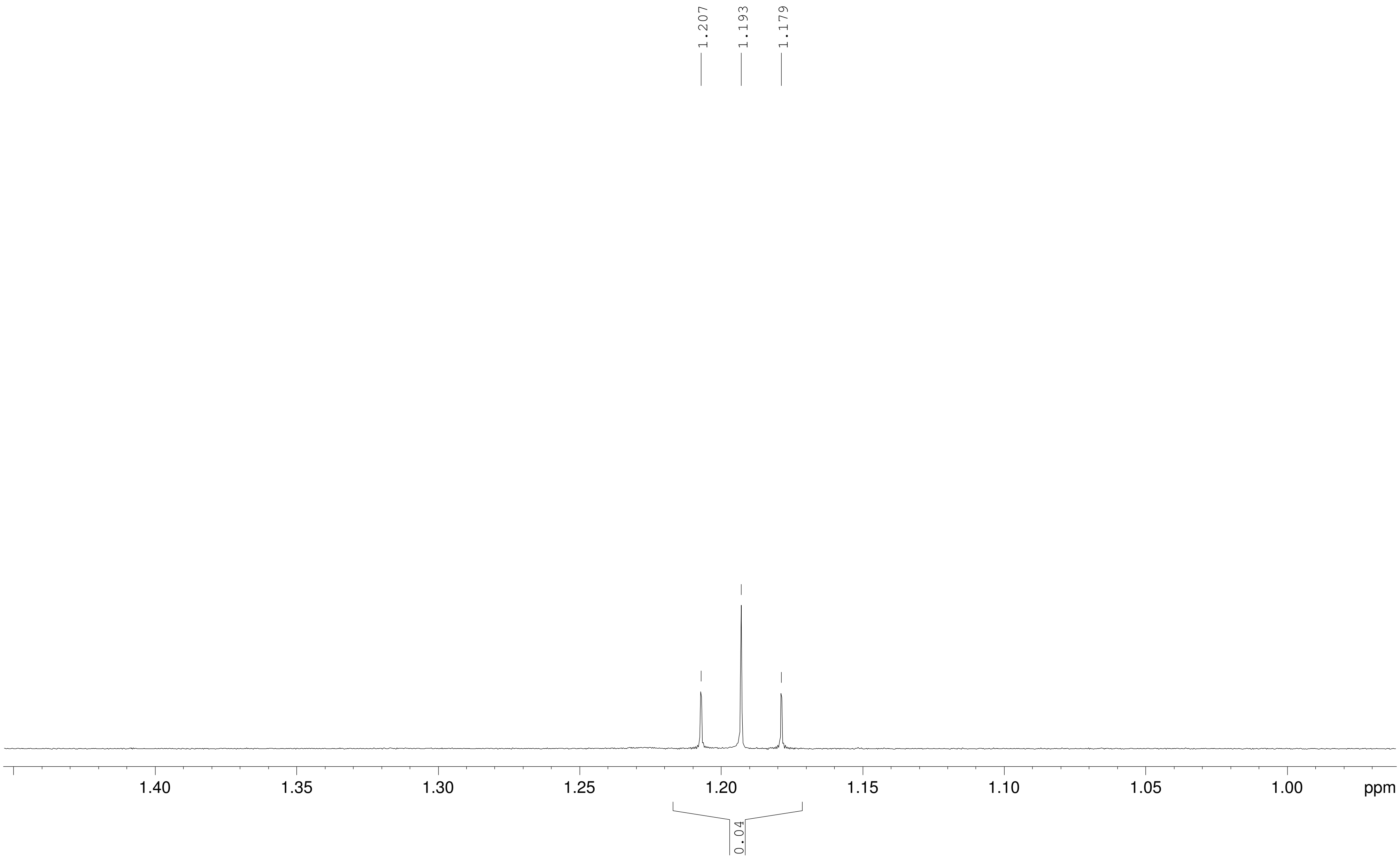

4008-13C
A3

Current Data Parameters
NAME 4008
EXPNO 2
PROCNO 1

F2 - Acquisition Parameters
Date_ 20170728
PROBHD 5 mm PABBI 1H/
PULPROG zg_pi_CPD
SOLVENT D2O
TE 298.2 K
SWH 31249.381 Hz
AQ 1.0485969 sec
NS 1116
FIDRES 0.476828 Hz
D1 1.50000000 sec
D11 0.03000000 sec
D14 0.00050000 sec

======== CHANNEL f1 ========
SFO1 125.7904831 MHz
NUC1 13C
P1 15.00 usec
P2 30.00 usec
PLW1 78.52400208 W

======== CHANNEL f2 ========
SFO2 500.2020008 MHz
NUC2 1H
PLW2 12.50000000 W
PLW12 0.12500000 W

F2 - Processing parameters
SI 65536
LB 0.50 Hz

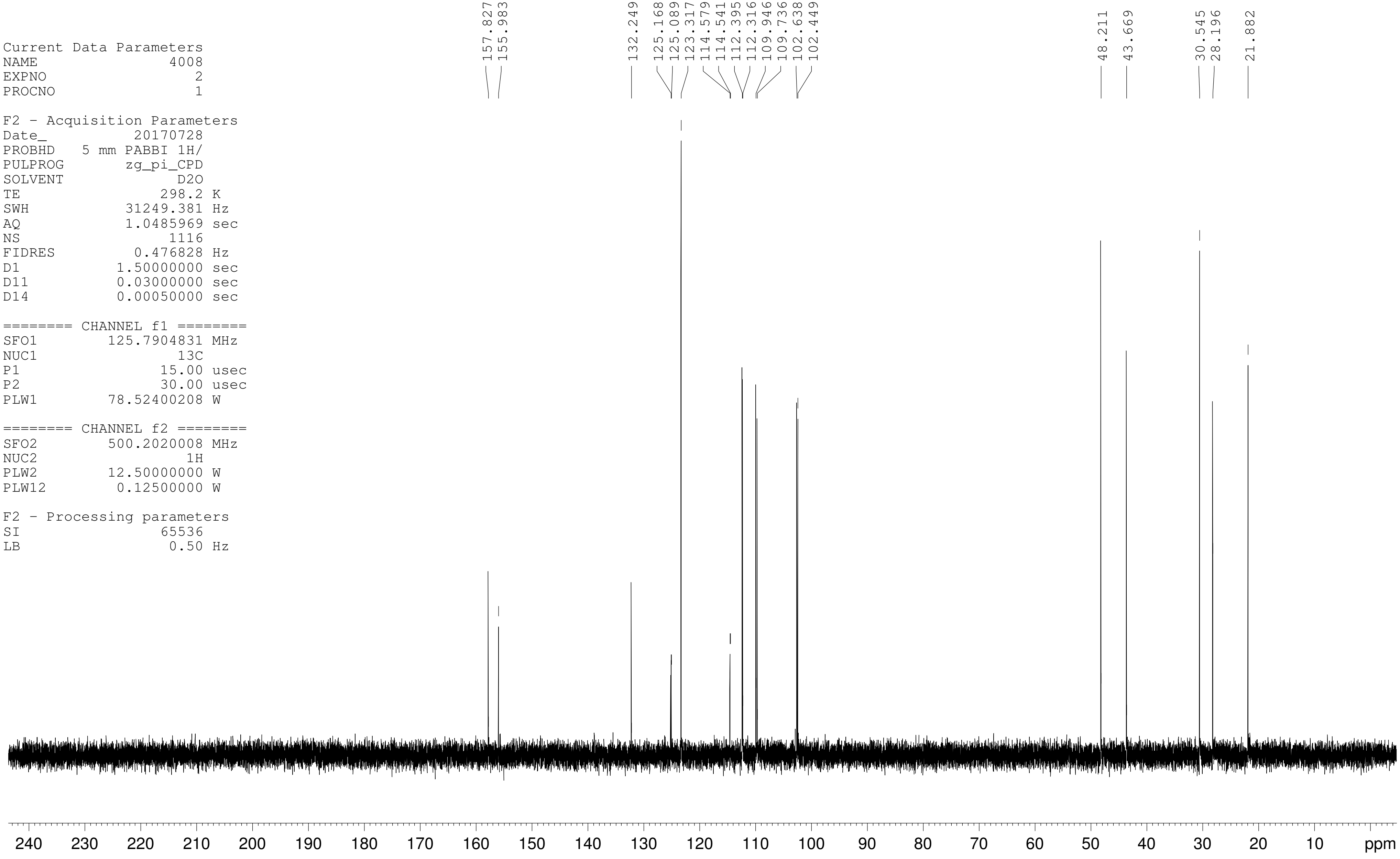

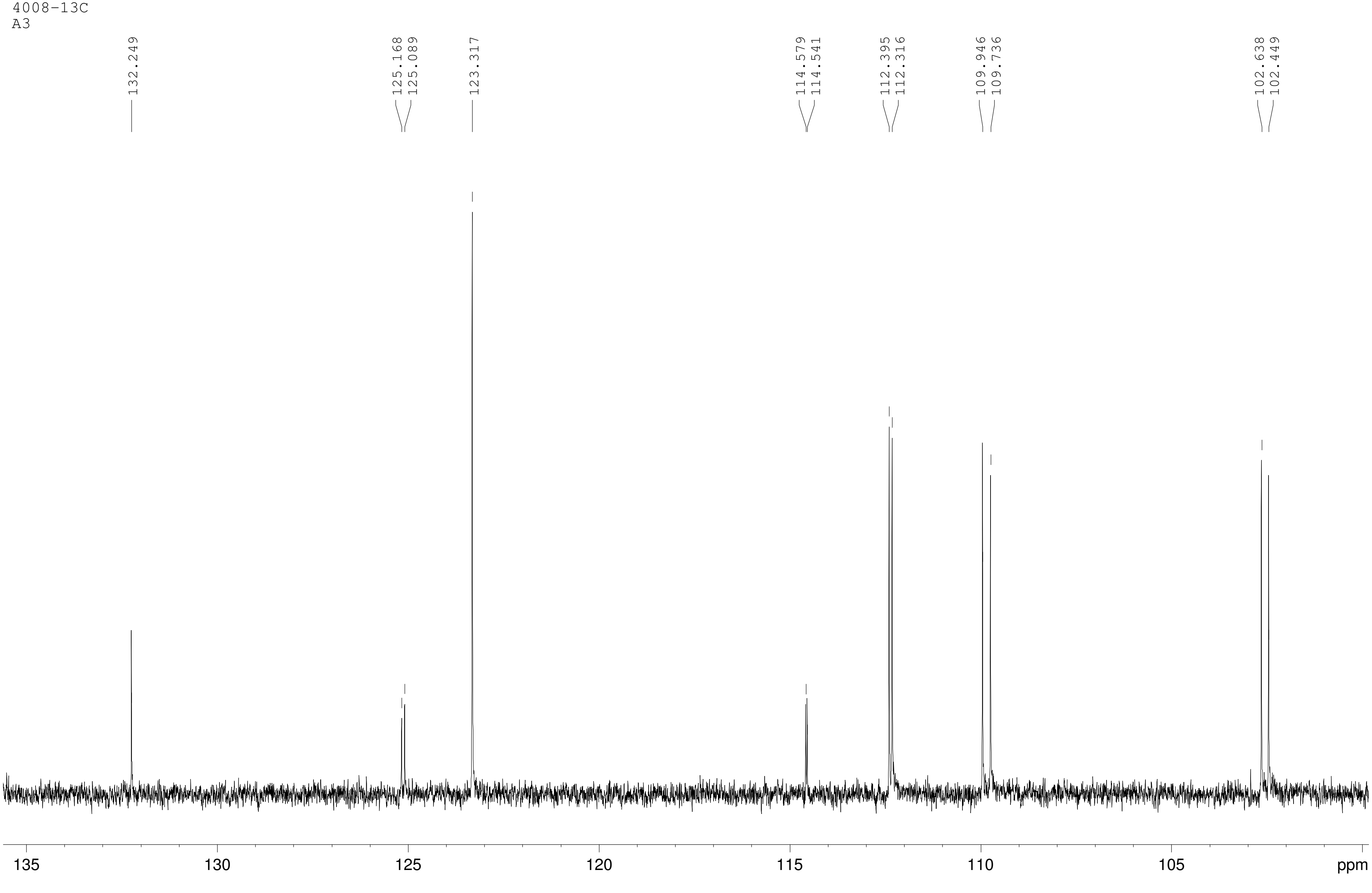
4008-13C
A3
132.249
125.168
125.089
123.317
114.579
114.541
112.395
112.316
109.946
109.736
102.638
102.449
135
130
125
120
115
110
105
ppm

4008-HMBC
A3

Current Data Parameters	
NAME	4008
EXPNO	4
PROCNO	1

F2 - Acquisition Parameters	
Date_	20170728
PROBHD	5 mm PABBI 1H/
PULPROG	hmbcgplpndqf
SOLVENT	D2O
TE	297.8 K
SWH	4870.130 Hz
AQ	0.2102613 sec
NS	4
FIDRES	2.377993 Hz
D0	0.00000300 sec
D1	1.50000000 sec
D2	0.00344828 sec
D6	0.05000000 sec
D16	0.00020000 sec

======== CHANNEL f1 ========	
SFO1	500.2022299 MHz
NUC1	1H
P1	8.00 usec
P2	16.00 usec
PLW1	12.69999981 W

======== CHANNEL f2 ========	
SFO2	125.7871163 MHz
NUC2	13C
P3	15.00 usec
PLW2	78.52400208 W

====== GRADIENT CHANNEL =====	
P16	1000.00 usec

F1 - Acquisition parameters	
TD	128
SFO1	125.7871 MHz
FIDRES	165.519073 Hz
SW	168.431 ppm
FnMODE	QF

F2 - Processing parameters	
SI	2048
LB	0 Hz

F1 - Processing parameters	
SI	1024
MC2	QF
SF	125.7754528 MHz
WDW	SINE
SSB	0

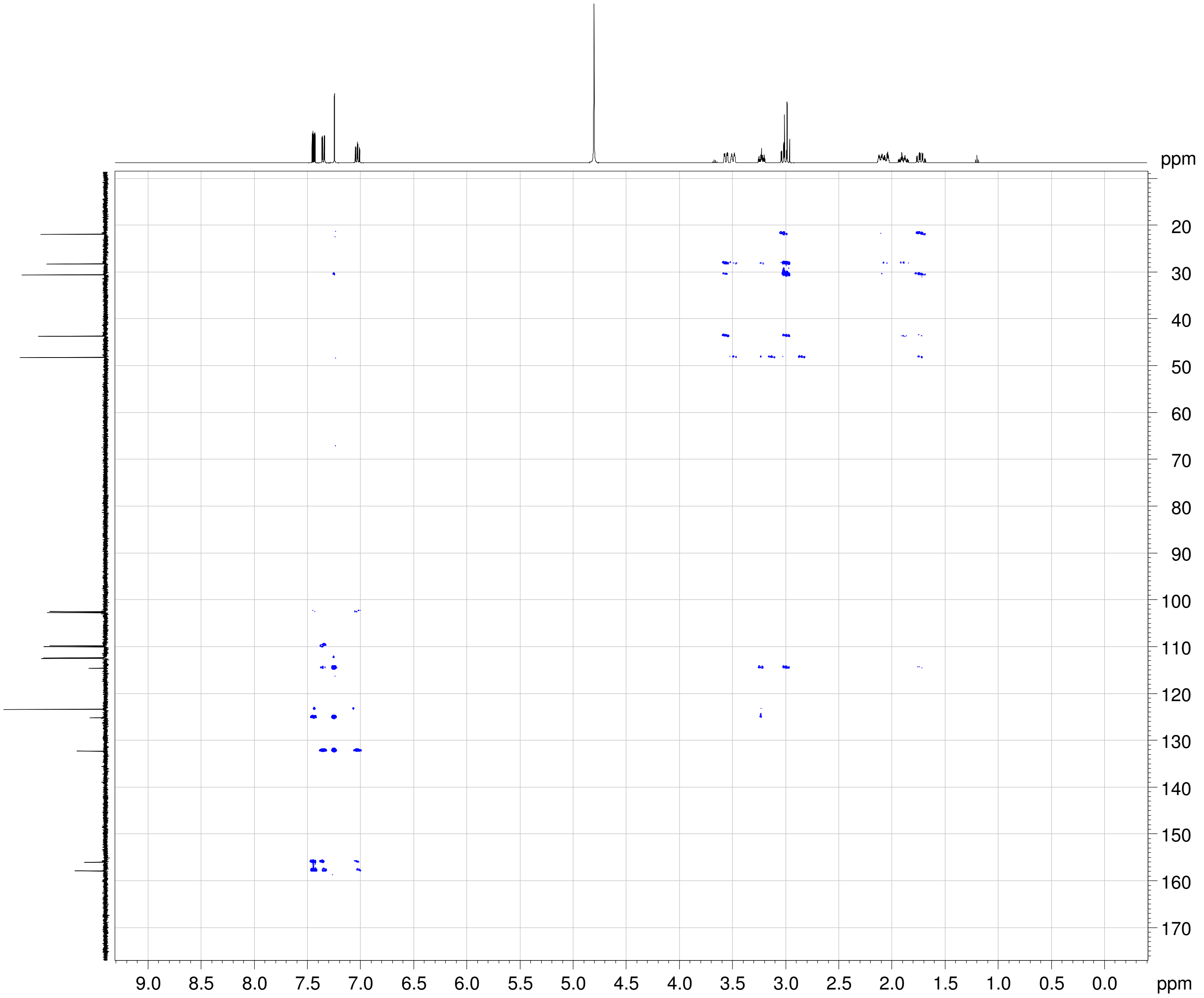

4008-HMBC
A3

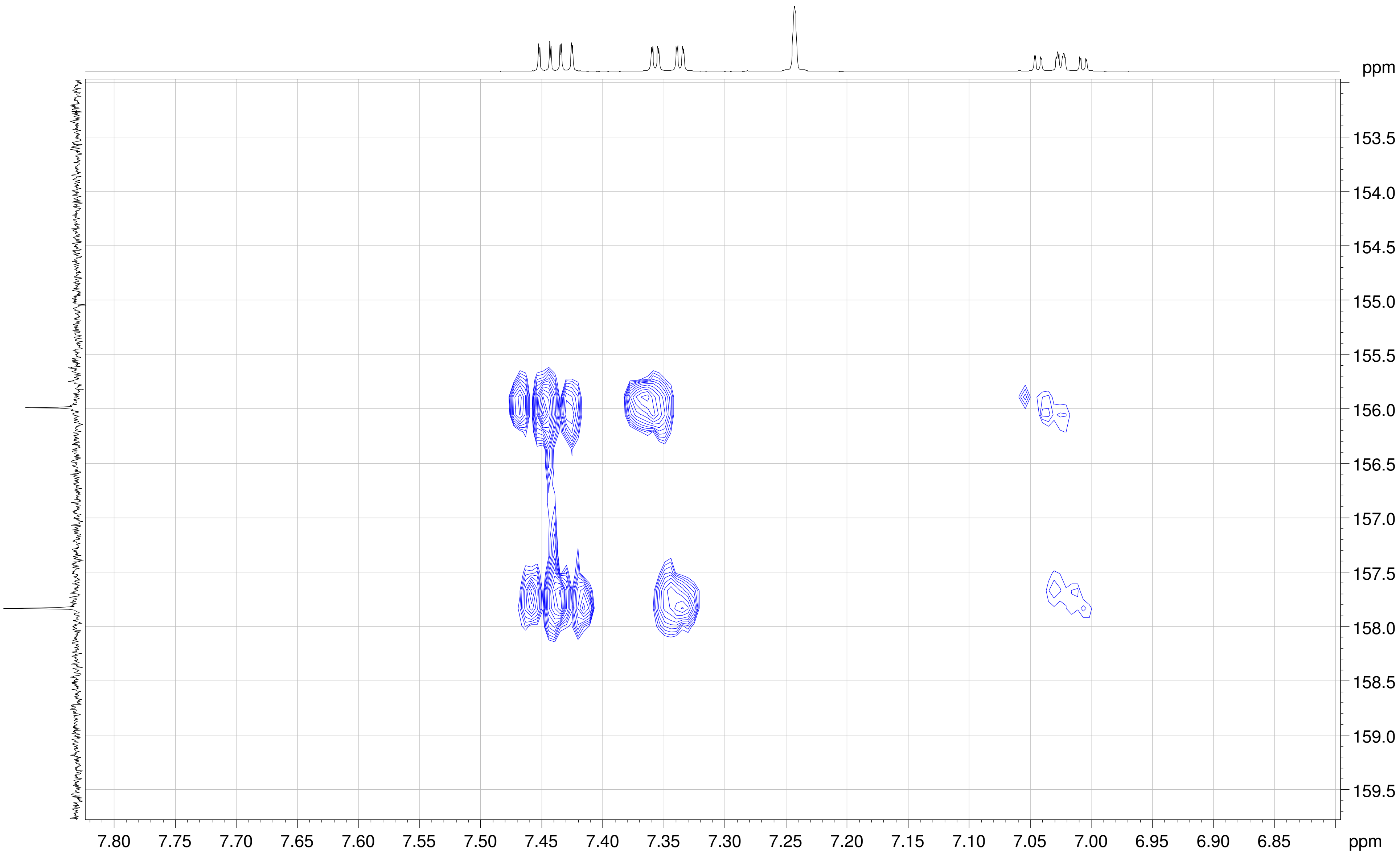

ppm
153.5
154.0
154.5
155.0
155.5
156.0
156.5
157.0
157.5
158.0
158.5
159.0
159.5
7.80 7.75 7.70 7.65 7.60 7.55 7.50 7.45 7.40 7.35 7.30 7.25 7.20 7.15 7.10 7.05 7.00 6.95 6.90 6.85
ppm

4008-HMBC
A3

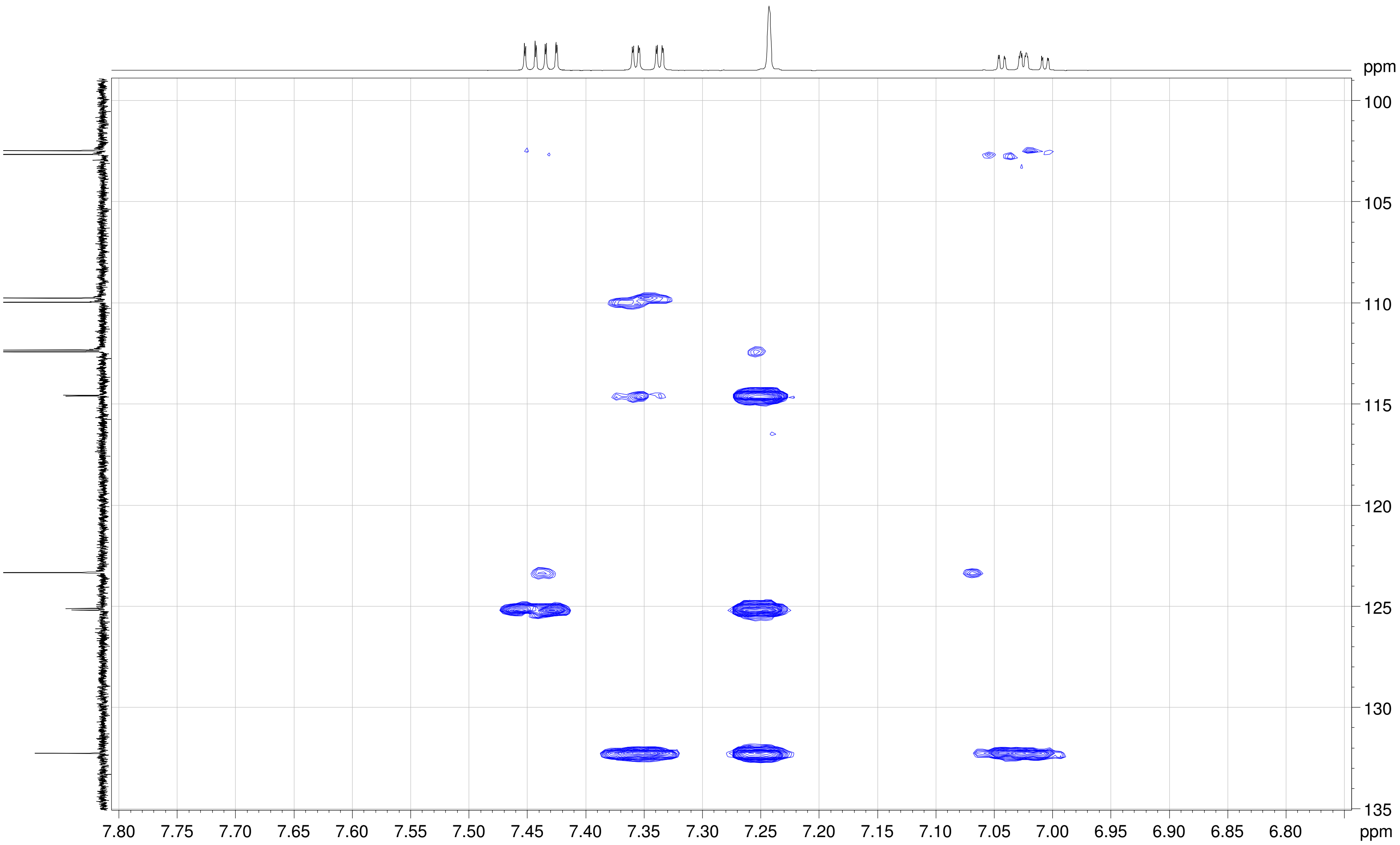

ppm
100
105
110
115
120
125
130
135
7.80 7.75 7.70 7.65 7.60 7.55 7.50 7.45 7.40 7.35 7.30 7.25 7.20 7.15 7.10 7.05 7.00 6.95 6.90 6.85 6.80
ppm

4008-HMBC
A3

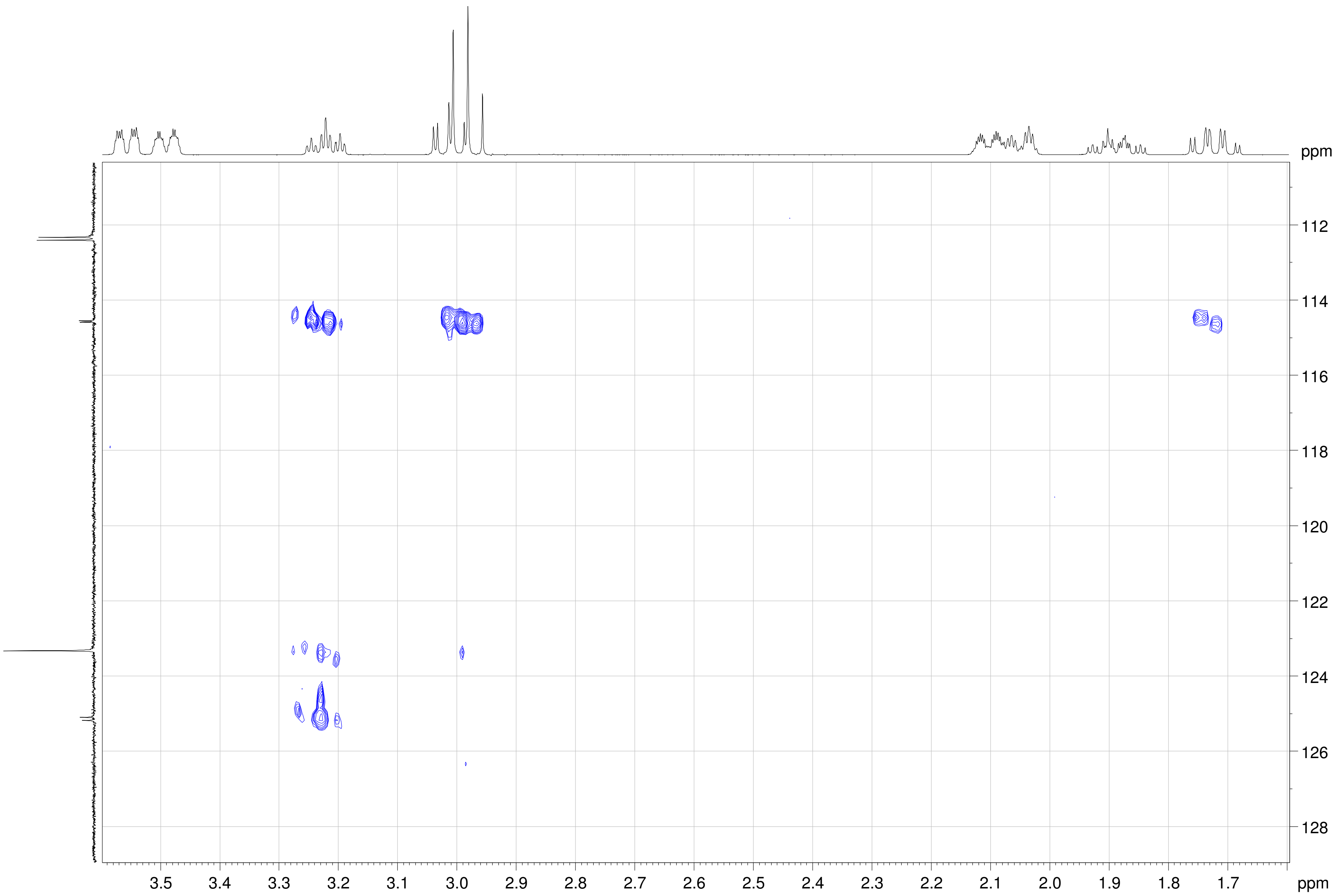
ppm
112
114
116
118
120
122
124
126
128
3.5
3.4
3.3
3.2
3.1
3.0
2.9
2.8
2.7
2.6
2.5
2.4
2.3
2.2
2.1
2.0
1.9
1.8
1.7
ppm

4008-HMBC
A3

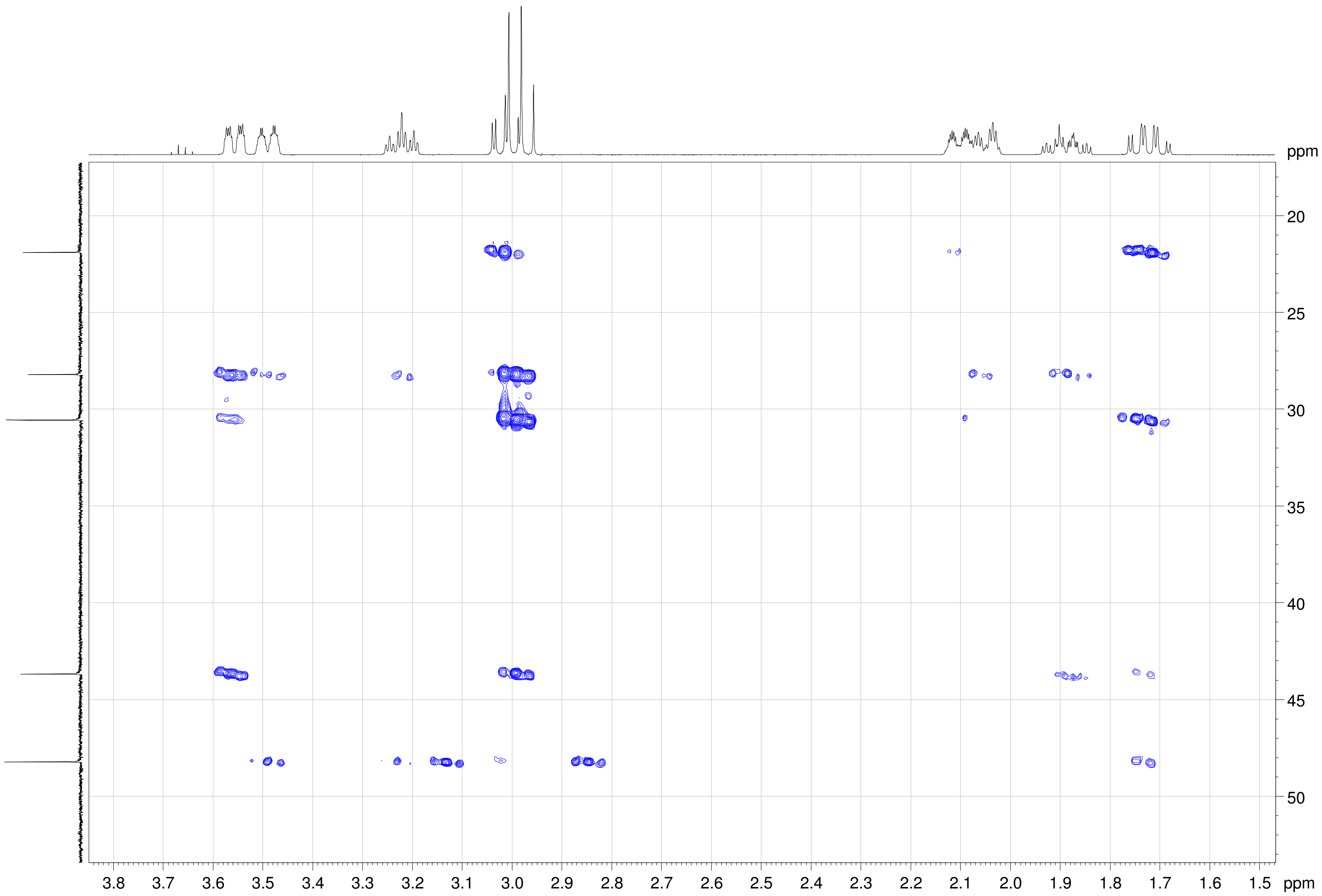
ppm
20
25
30
35
40
45
50
3.8 3.7 3.6 3.5 3.4 3.3 3.2 3.1 3.0 2.9 2.8 2.7 2.6 2.5 2.4 2.3 2.2 2.1 2.0 1.9 1.8 1.7 1.6 1.5 ppm

4008-HMBC
A3

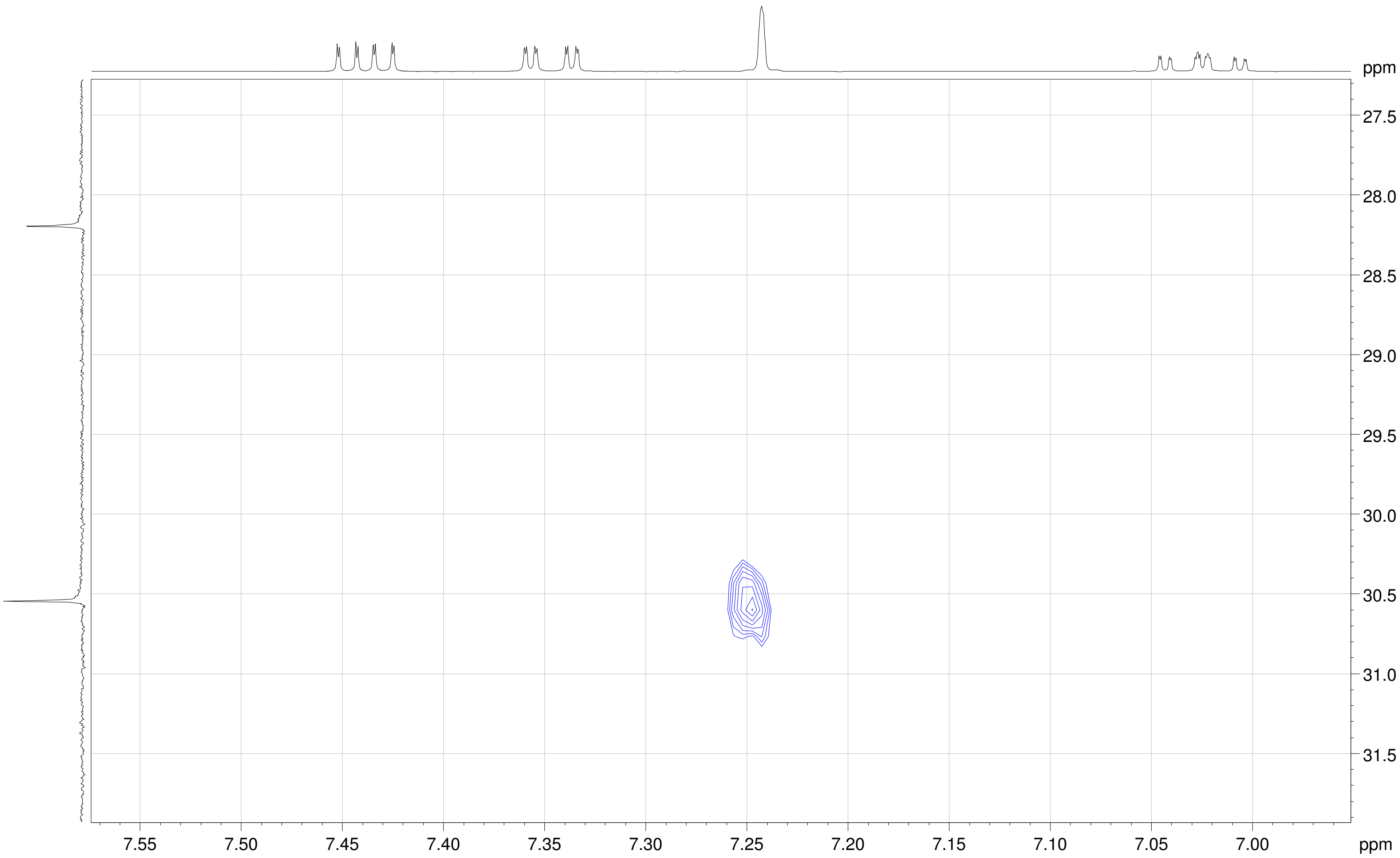
ppm
27.5
28.0
28.5
29.0
29.5
30.0
30.5
31.0
31.5
7.55
7.50
7.45
7.40
7.35
7.30
7.25
7.20
7.15
7.10
7.05
7.00
ppm

4008-HSQC
A3

```
Current Data Parameters
NAME                 4008
EXPNO                   3
PROCNO                  1

F2 - Acquisition Parameters
Date_            20170728
PROBHD   5 mm PABBI 1H/
PULPROG  hsqcedetgpsisp2.3
SOLVENT               D2O
TE                  298.5 K
SWH              4870.130 Hz
AQ              0.2102613 sec
NS                      2
FIDRES           2.377993 Hz
D0             0.00000300 sec
D1             2.00000000 sec
D4             0.00172414 sec
D11            0.03000000 sec
D16            0.00020000 sec
D21            0.00360000 sec
D24            0.00089000 sec

======== CHANNEL f1 ========
SFO1          500.2022299 MHz
NUC1                   1H
P1                   8.00 usec
P2                  16.00 usec
P28      0 usec
PLW1          12.69999981 W

======== CHANNEL f2 ========
SFO2          125.7871163 MHz
NUC2                  13C
P3                  15.00 usec
P14                500.00 usec
P24               2000.00 usec
P31               1900.00 usec
PLW0     0 W
PLW2          78.52400208 W
PLW12          3.60570002 W

====== GRADIENT CHANNEL =====
P16               1000.00 usec
P19                600.00 usec

F1 - Acquisition parameters
TD                    128
SFO1             125.7871 MHz
FIDRES         165.519073 Hz
SW                168.431 ppm
FnMODE       Echo-Antiecho

F2 - Processing parameters
```

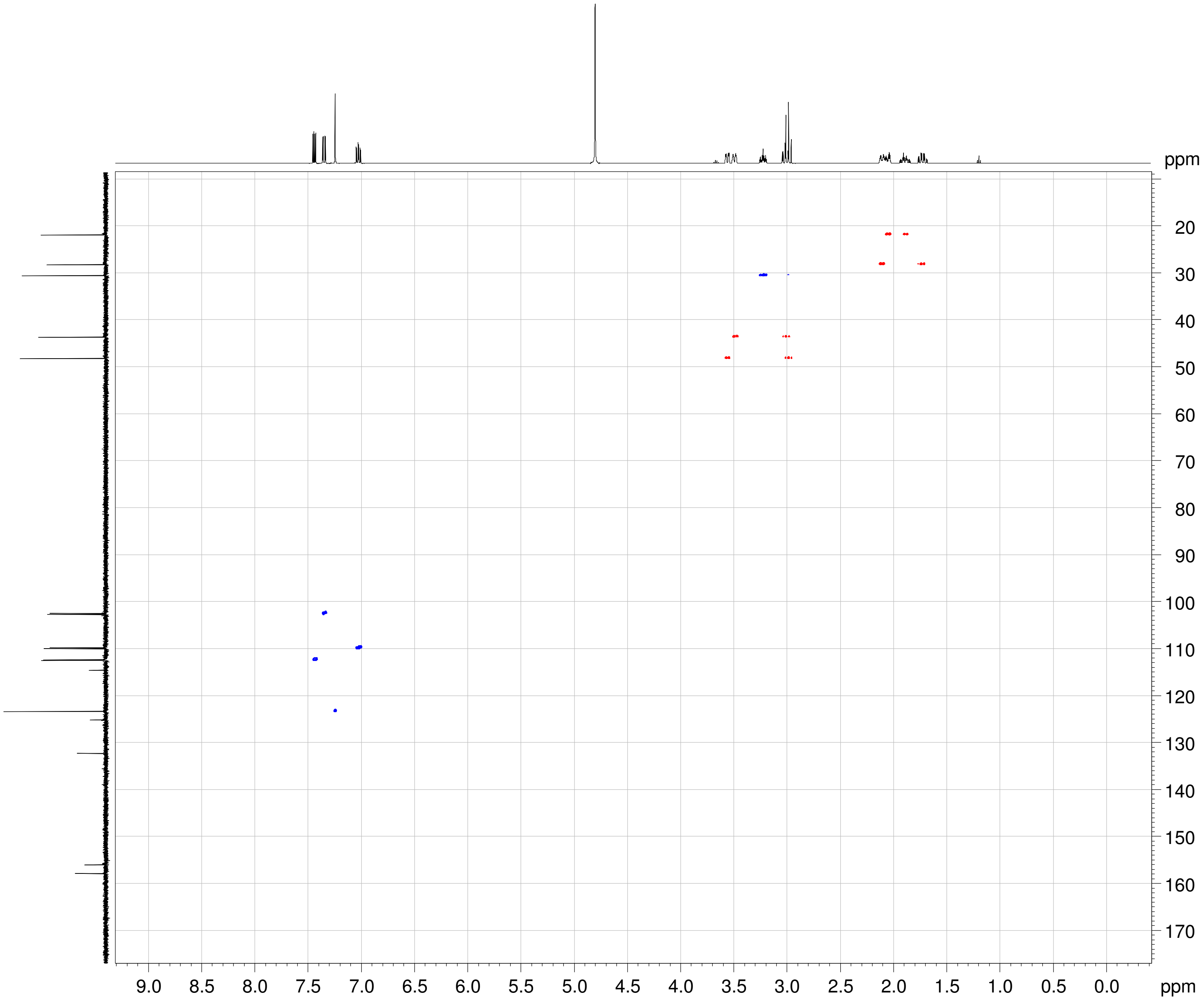

4008-HSQC
A3

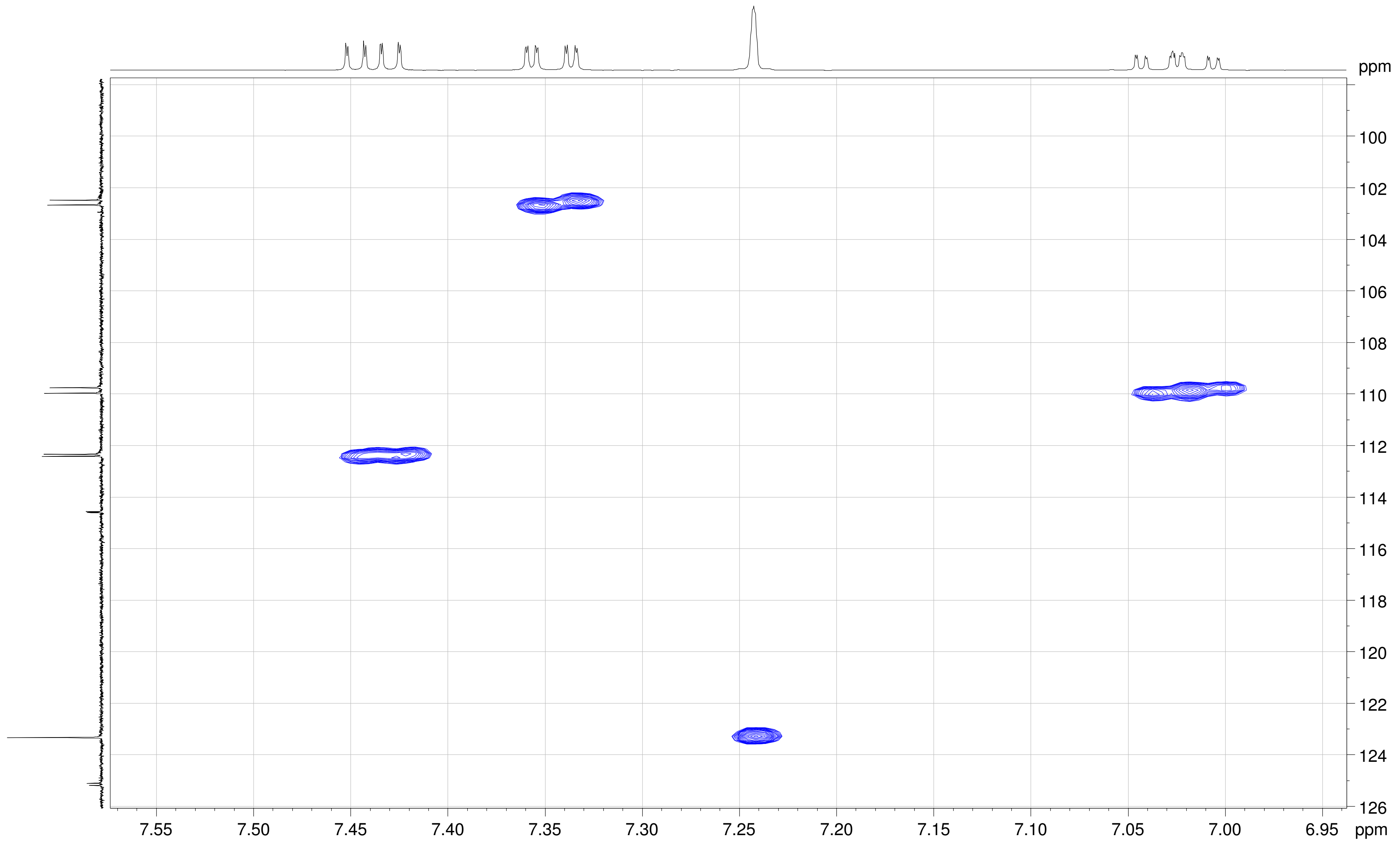

ppm
100
102
104
106
108
110
112
114
116
118
120
122
124
126
7.55
7.50
7.45
7.40
7.35
7.30
7.25
7.20
7.15
7.10
7.05
7.00
6.95
ppm

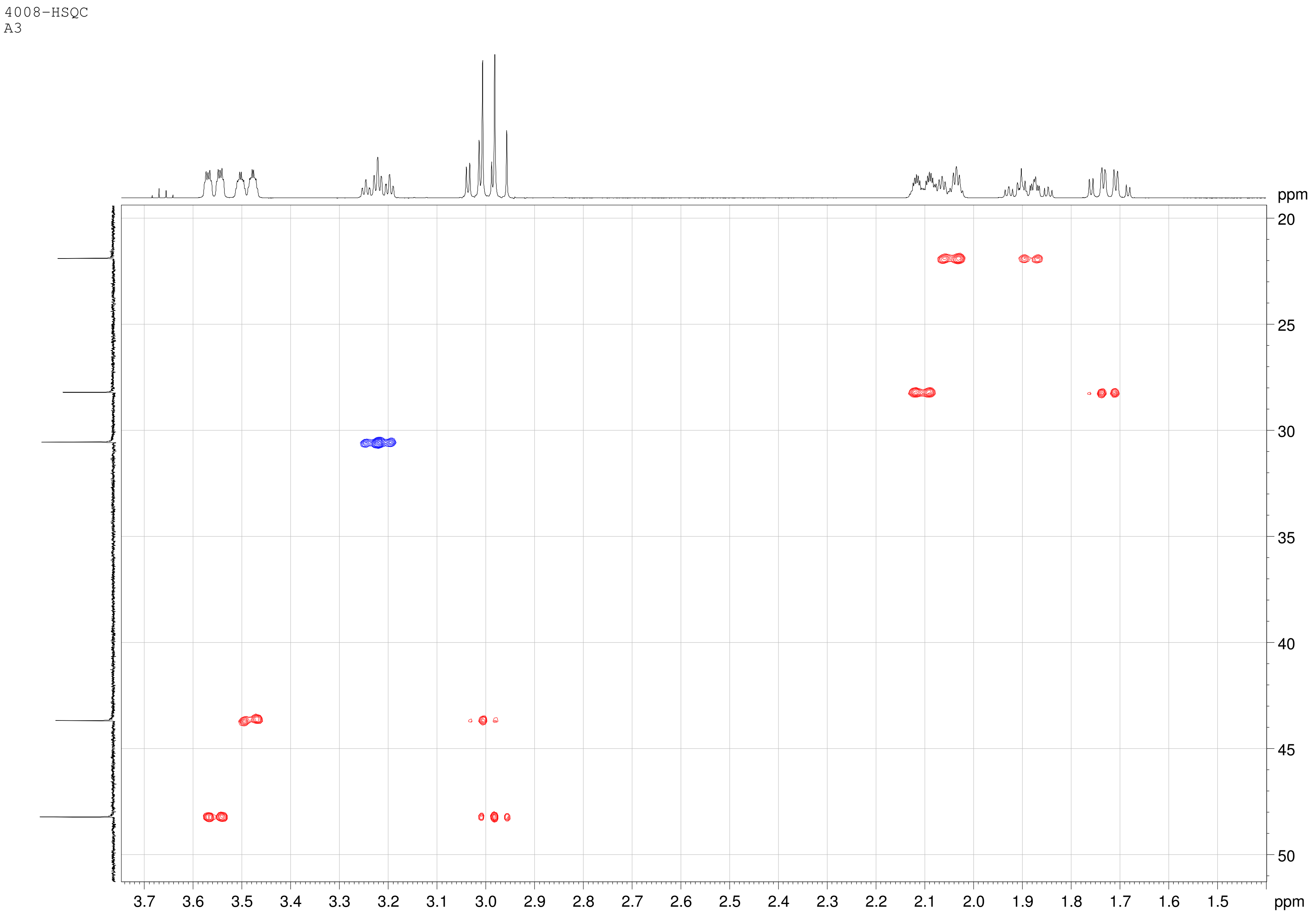

4008-HSQC
A3
ppm
20
25
30
35
40
45
50
3.7
3.6
3.5
3.4
3.3
3.2
3.1
3.0
2.9
2.8
2.7
2.6
2.5
2.4
2.3
2.2
2.1
2.0
1.9
1.8
1.7
1.6
1.5
ppm

(R)-10c

4499-1H
ExHCl 0,07

Current Data Parameters
NAME 4499
EXPNO 1
PROCNO 1

F2 - Acquisition Parameters
Date_ 20180118
PROBHD 5 mm CPPBBO BB
PULPROG zg30
SOLVENT D2O
TE 298.2 K
SWH 10000.000 Hz
AQ 3.2767999 sec
NS 8
FIDRES 0.152588 Hz
D1 0 sec

======== CHANNEL f1 ========
SFO1 500.2030889 MHz
NUC1 1H
P1 11.30 usec
PLW1 13.69999981 W

F2 - Processing parameters
SI 65536
LB 0.20 Hz

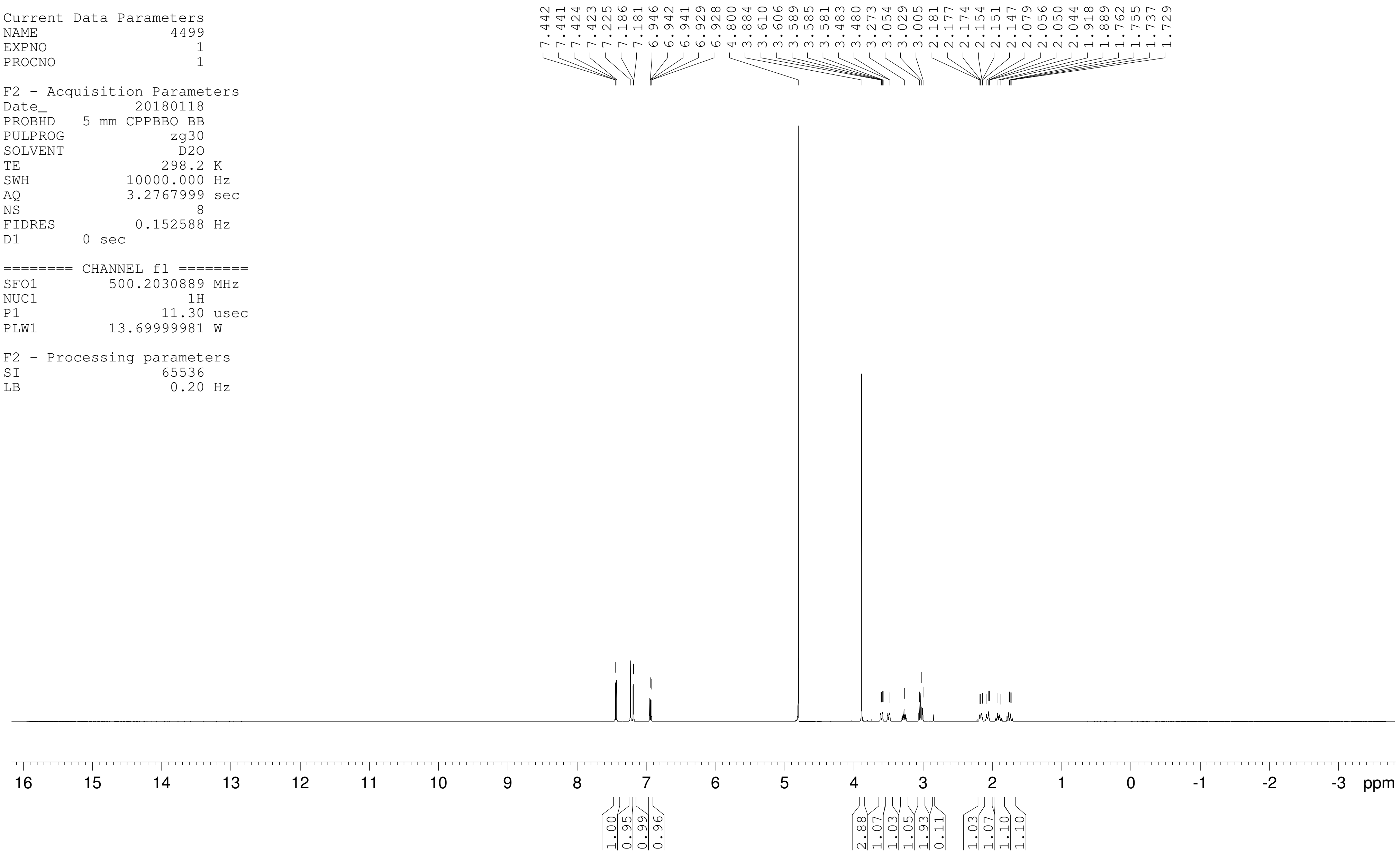

4499-1H
ExHCl 0,07

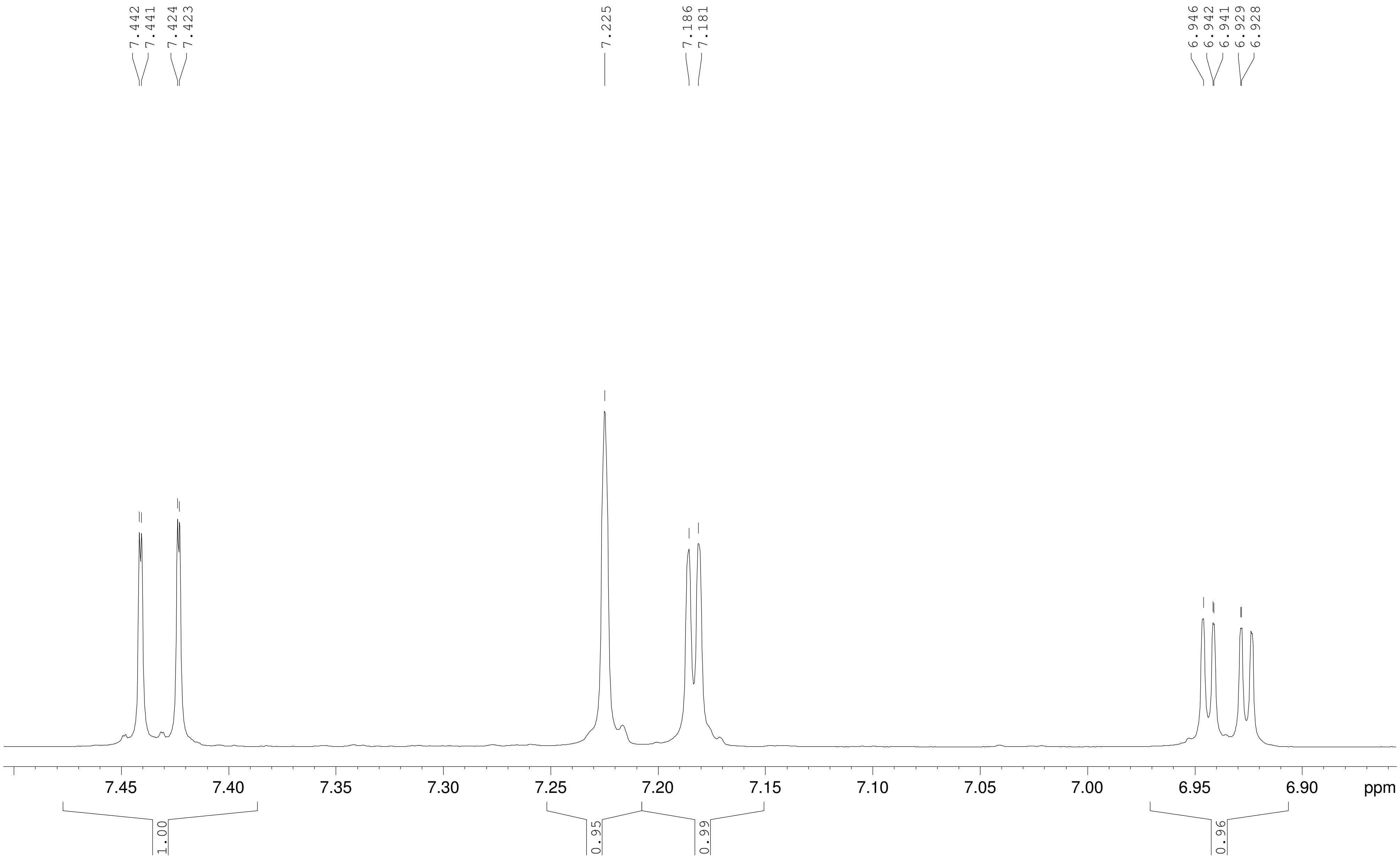

4499-1H
ExHCl 0,07

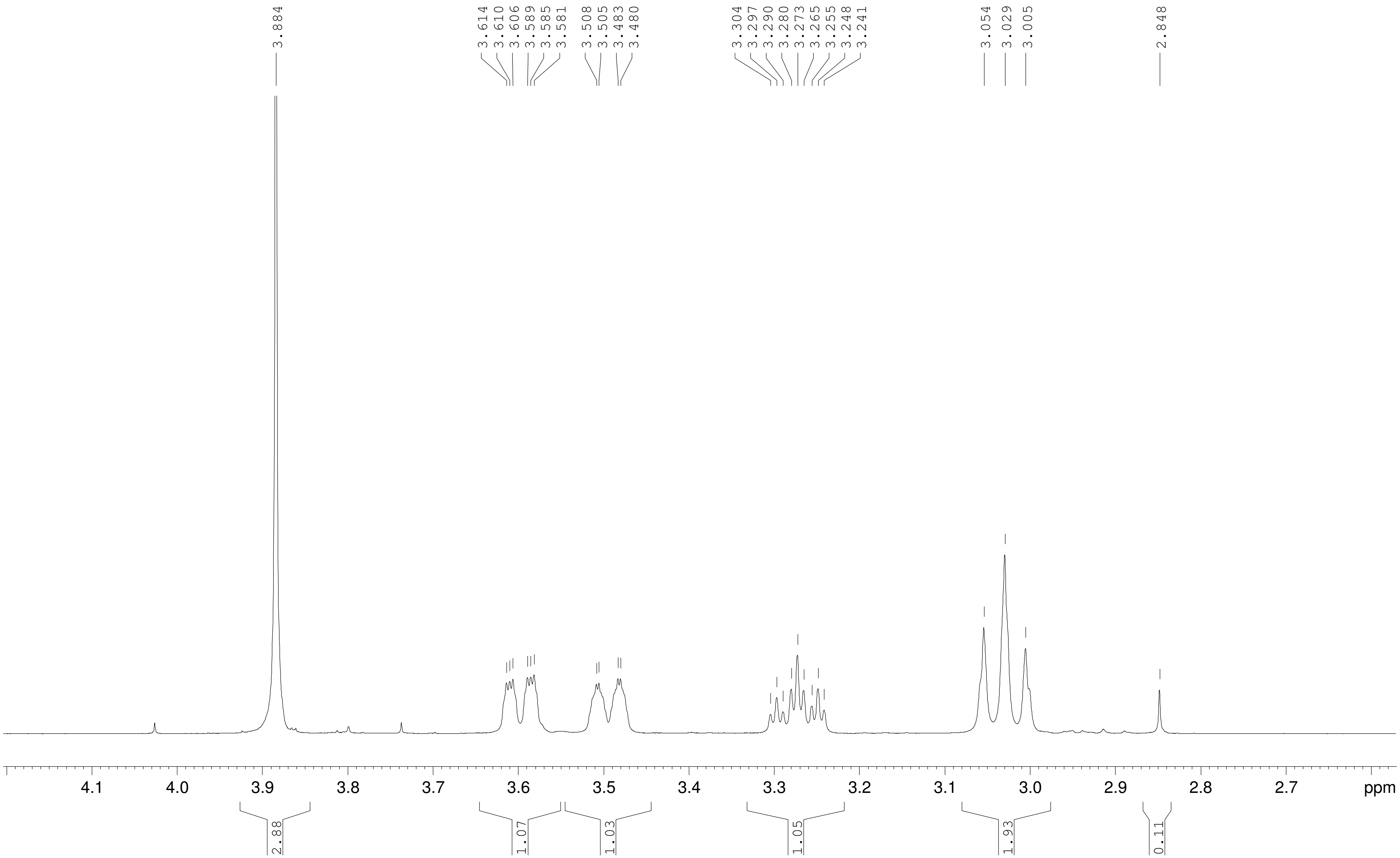

4499-1H
ExHCl 0,07

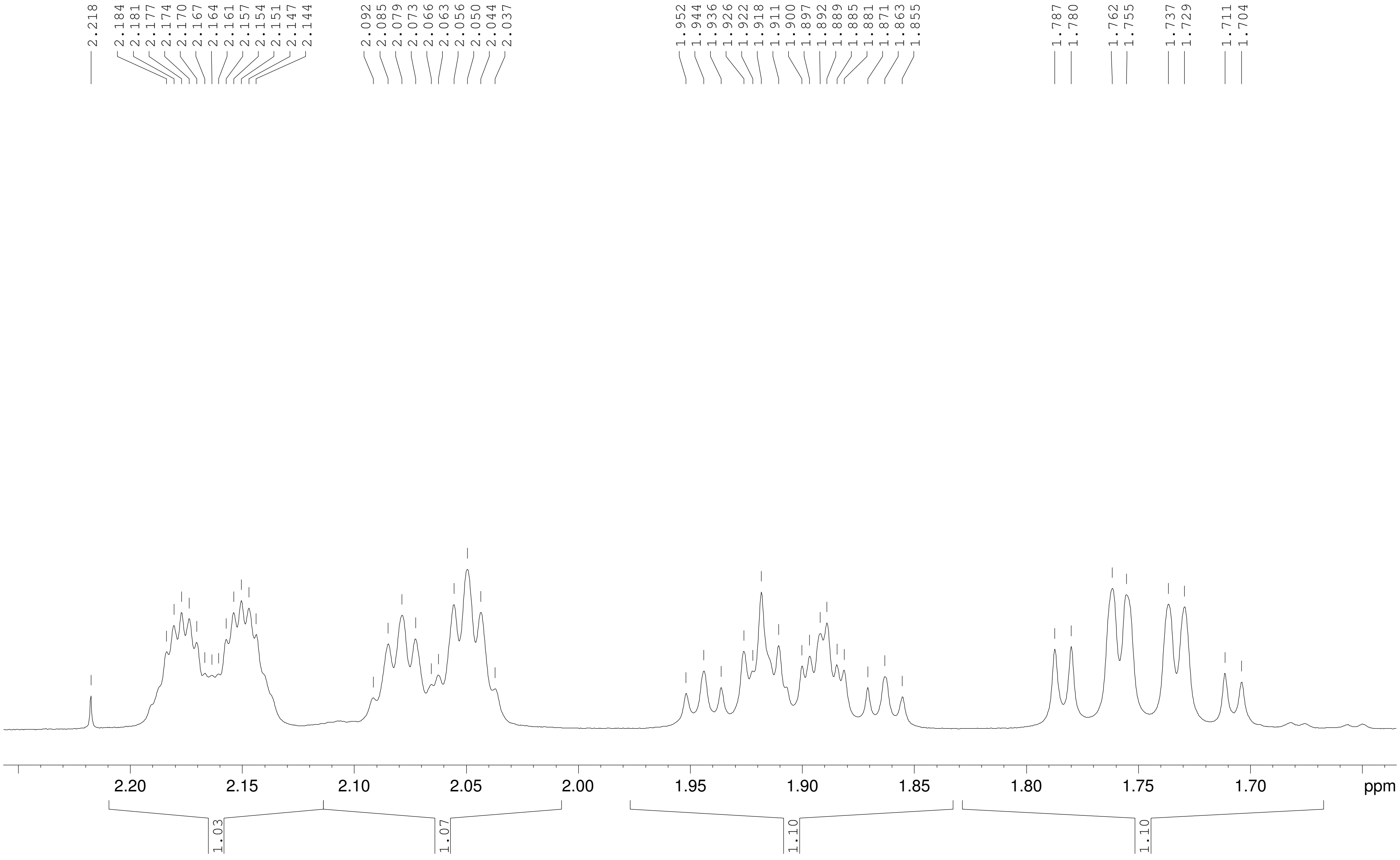

(S)-11c

1001-1H
A2

Current Data Parameters
NAME 1001
EXPNO 1
PROCNO 1

F2 - Acquisition Parameters
Date_ 20150323
PROBHD 5 mm CPPBBO BB
PULPROG zg30
SOLVENT D2O
TE 298.1 K
SWH 10000.000 Hz
AQ 3.2767999 sec
NS 8
FIDRES 0.152588 Hz
D1 0 sec

======== CHANNEL f1 ========
SFO1 500.2030889 MHz
NUC1 1H
P1 11.30 usec
PLW1 13.69999981 W

F2 - Processing parameters
SI 65536
LB 0 Hz

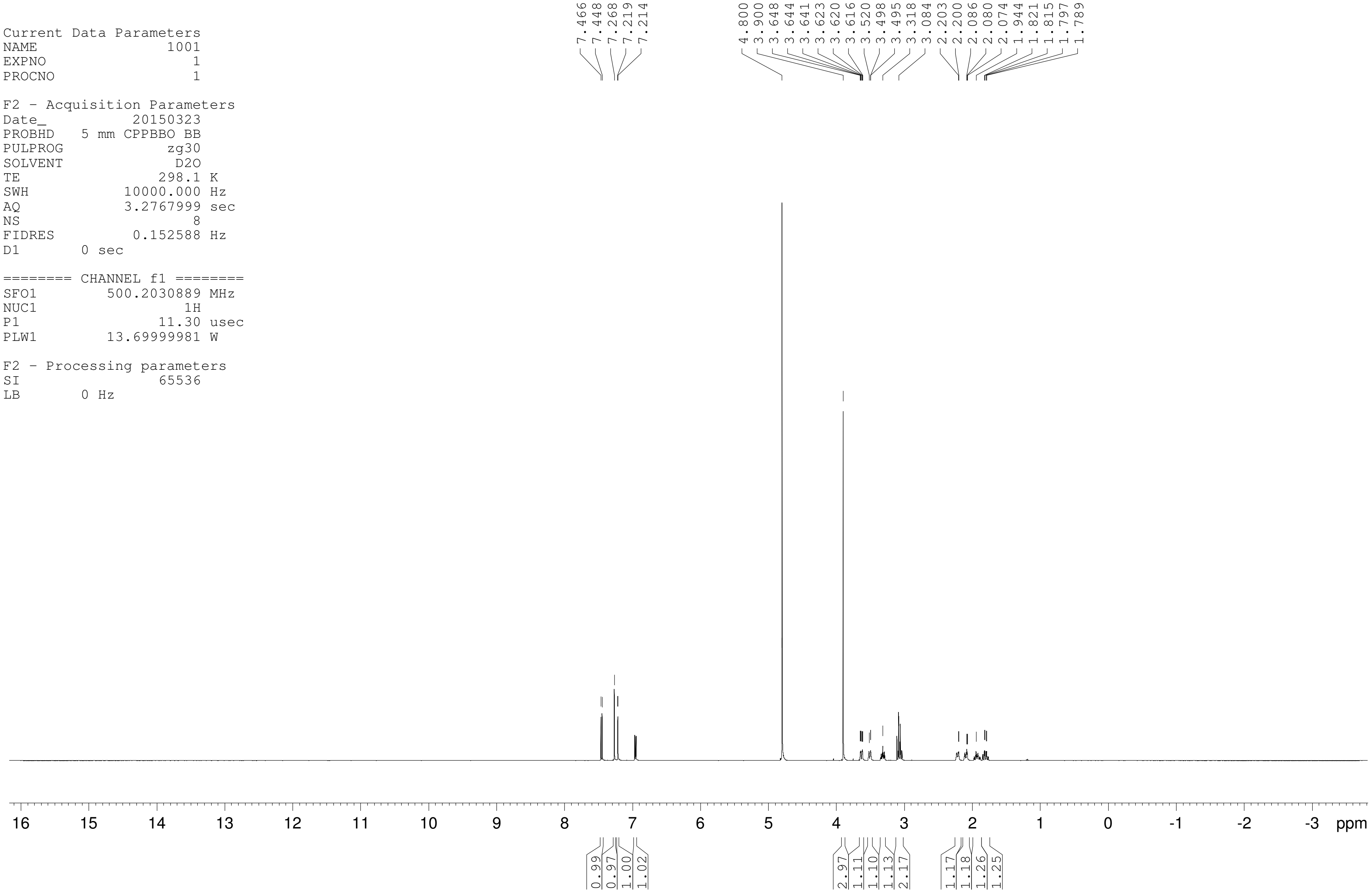

1001-1H
A2

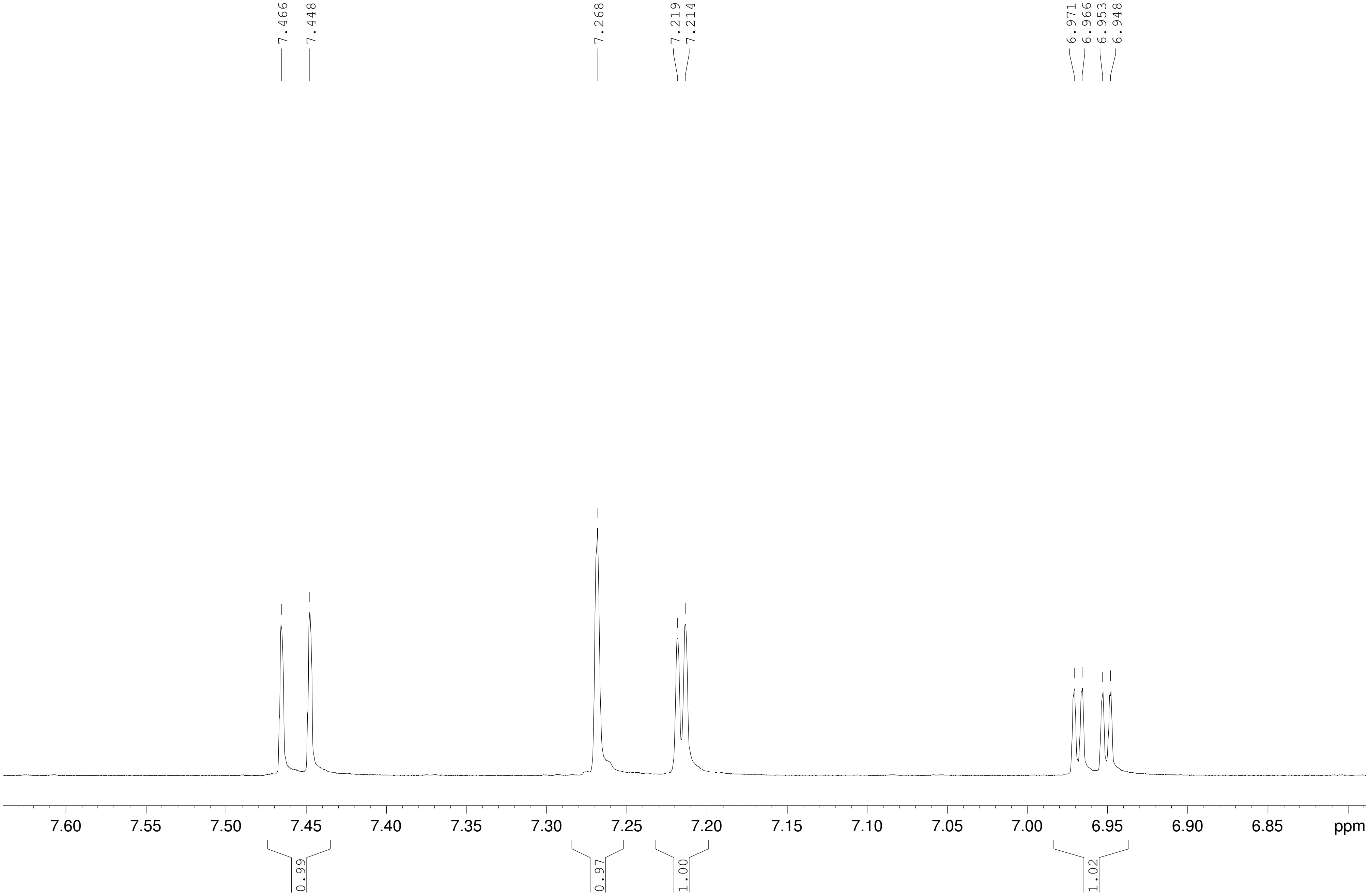
7.466
7.448
7.268
7.219
7.214
6.971
6.966
6.953
6.948
7.60
7.55
7.50
7.45
7.40
7.35
7.30
7.25
7.20
7.15
7.10
7.05
7.00
6.95
6.90
6.85
ppm
0.99
0.97
1.00
1.02

1001-1H
A2

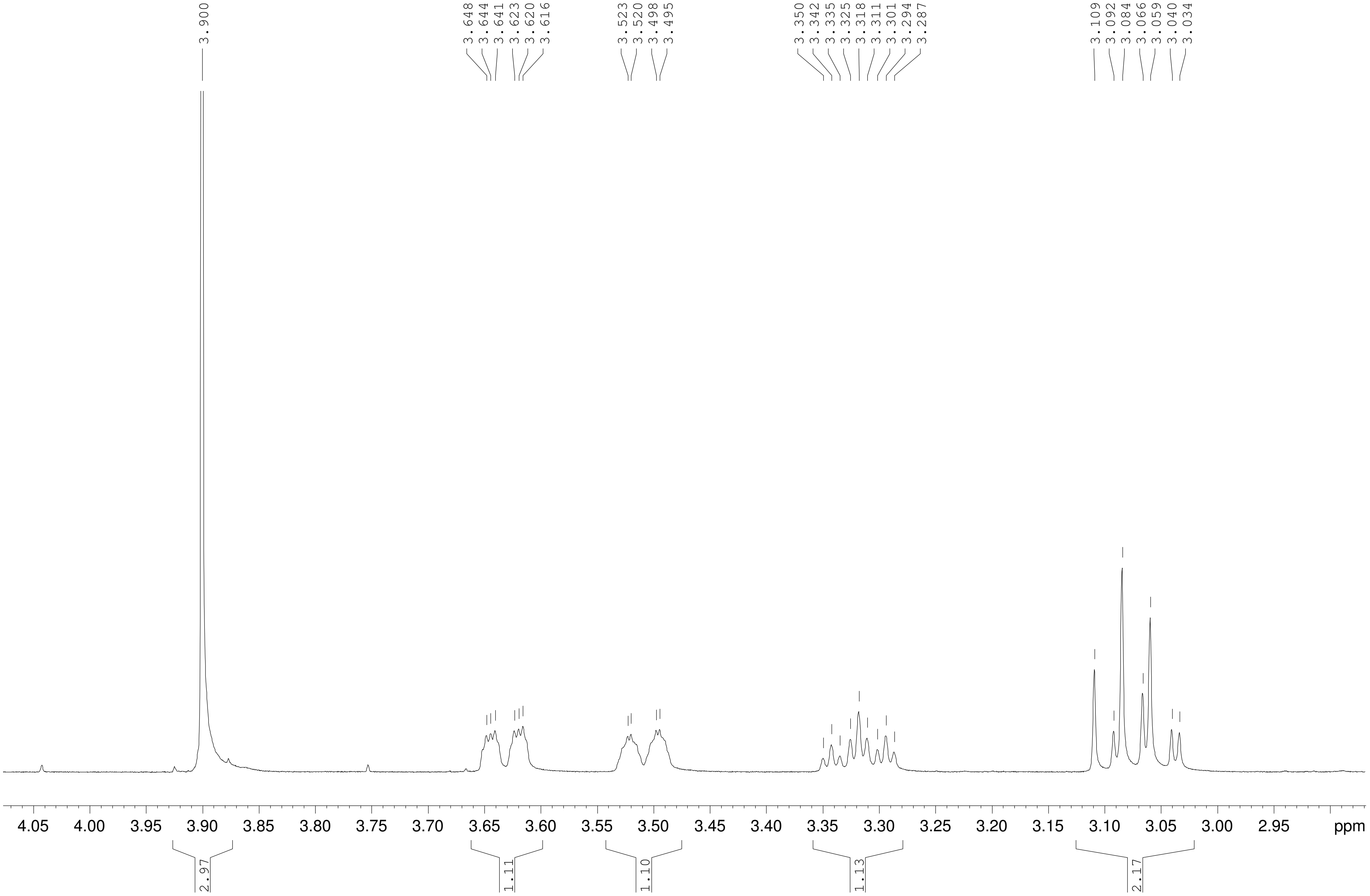
3.900
3.648
3.644
3.641
3.623
3.620
3.616
3.523
3.520
3.498
3.495
3.350
3.342
3.335
3.325
3.318
3.311
3.301
3.294
3.287
3.109
3.092
3.084
3.066
3.059
3.040
3.034
4.05 4.00 3.95 3.90 3.85 3.80 3.75 3.70 3.65 3.60 3.55 3.50 3.45 3.40 3.35 3.30 3.25 3.20 3.15 3.10 3.05 3.00 2.95 ppm
2.97
1.11
1.10
1.13
2.17

1001-1H
A2

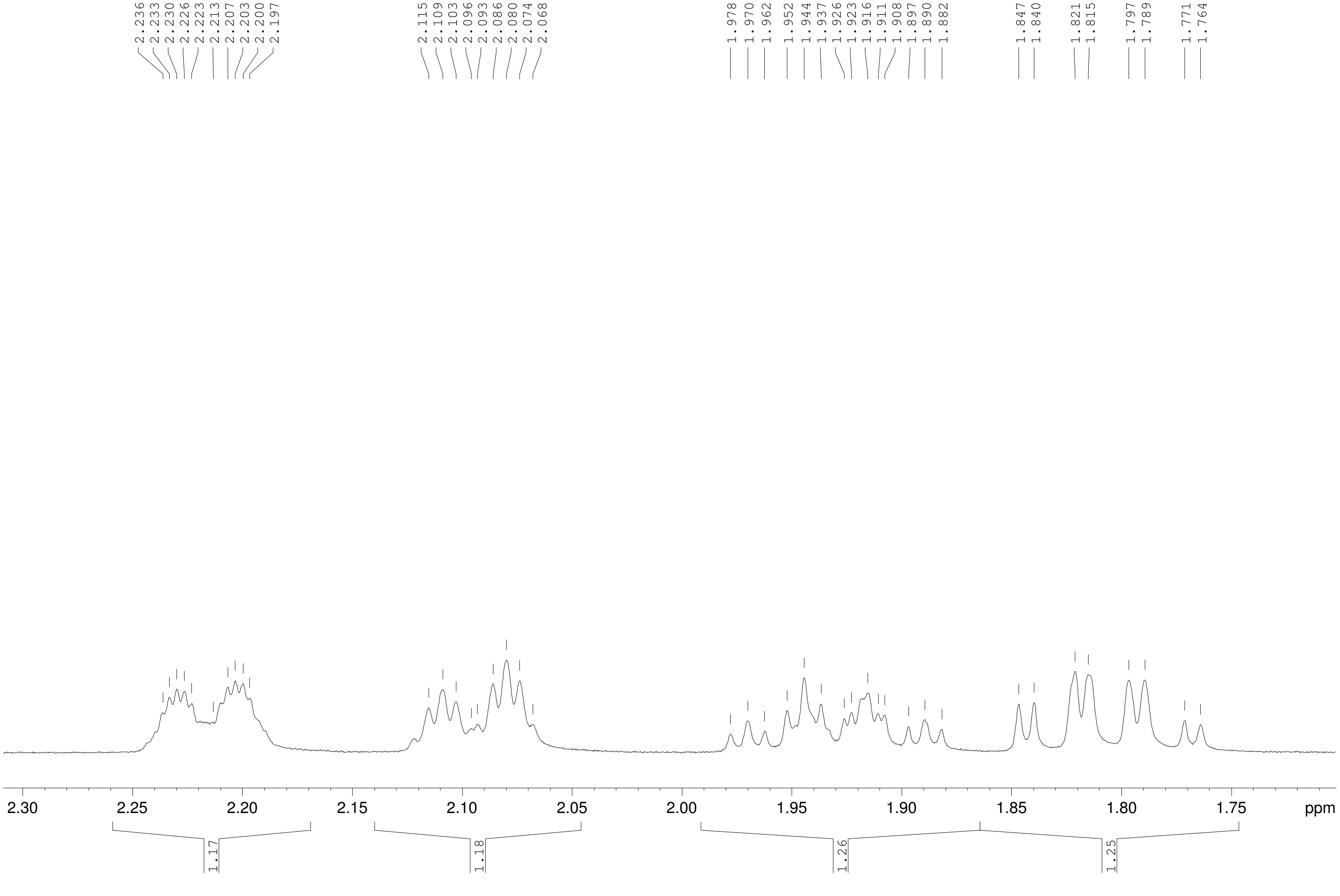

2.236
2.233
2.230
2.226
2.223
2.213
2.207
2.203
2.200
2.197
2.115
2.109
2.103
2.096
2.093
2.086
2.080
2.074
2.068
1.978
1.970
1.962
1.952
1.944
1.937
1.926
1.923
1.916
1.911
1.908
1.897
1.890
1.882
1.847
1.840
1.821
1.815
1.797
1.789
1.771
1.764
2.30
2.25
2.20
2.15
2.10
2.05
2.00
1.95
1.90
1.85
1.80
1.75
ppm
1.17
1.18
1.26
1.25

1001-13C
A2

Current Data Parameters
NAME 1001
EXPNO 2
PROCNO 1

F2 - Acquisition Parameters
Date_ 20150323
PROBHD 5 mm CPPBBO BB
PULPROG zg_pi_CPD
SOLVENT D2O
TE 298.1 K
SWH 31249.381 Hz
AQ 1.0485969 sec
NS 196
FIDRES 0.476828 Hz
D1 1.50000000 sec
D11 0.03000000 sec
D14 0.00050000 sec

======== CHANNEL f1 ========
SFO1 125.7904831 MHz
NUC1 13C
P1 10.00 usec
P2 20.00 usec
PLW1 55.00000000 W

======== CHANNEL f2 ========
SFO2 500.2020008 MHz
NUC2 1H
PLW2 13.00000000 W
PLW12 0.25937000 W

F2 - Processing parameters
SI 65536
LB 0.50 Hz

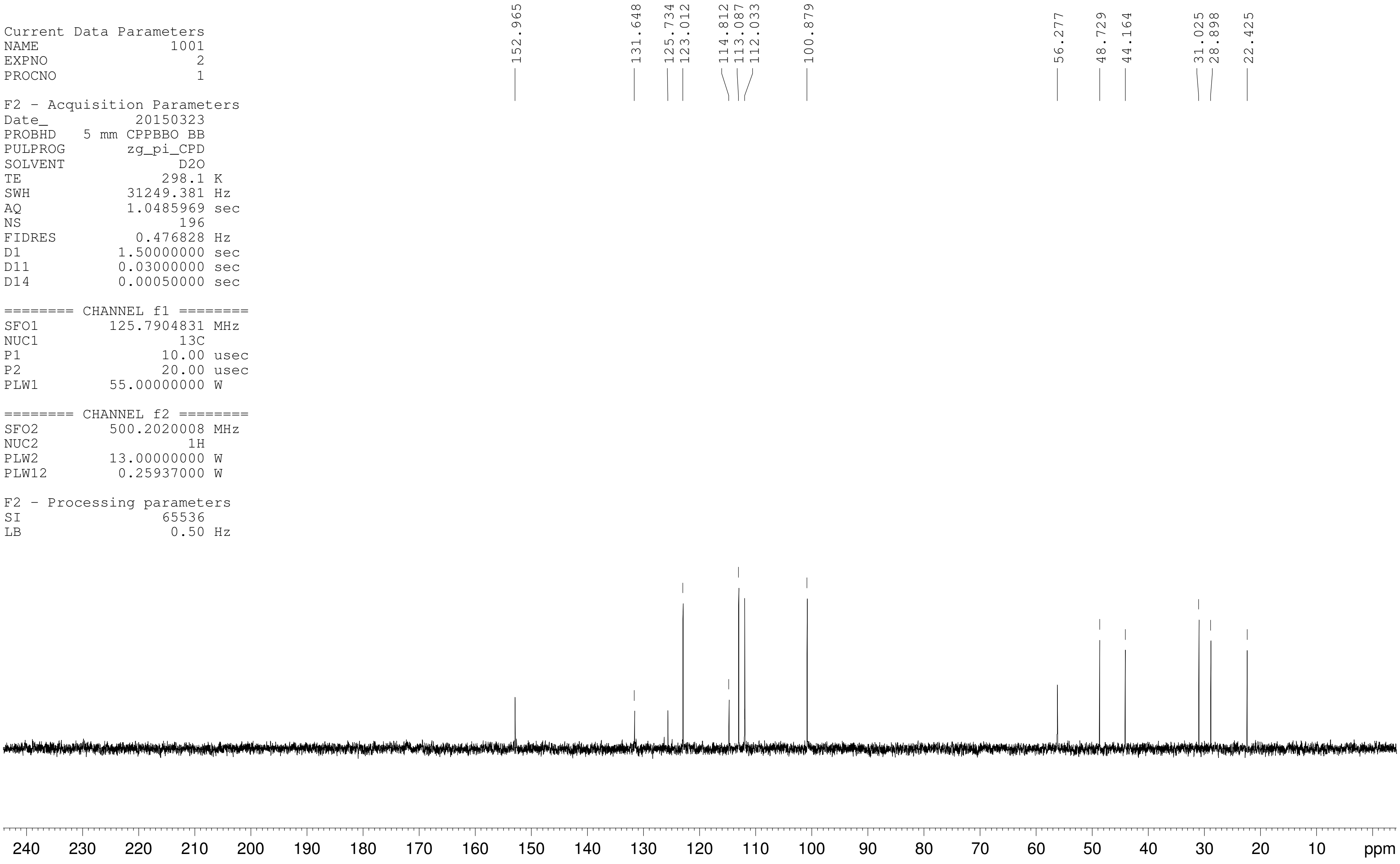

1001-COSY
A2

Current Data Parameters
NAME 1001
EXPNO 3
PROCNO 1

F2 - Acquisition Parameters
Date_ 20150424
PROBHD 5 mm CPPBBO BB
PULPROG cosygpppqf
SOLVENT D2O
TE 298.1 K
SWH 4520.796 Hz
AQ 0.2265088 sec
NS 2
FIDRES 2.207420 Hz
D0 0.00000300 sec
D1 2.00000000 sec
D11 0.03000000 sec
D12 0.00002000 sec
D13 0.00000400 sec
D16 0.00020000 sec

======== CHANNEL f1 ========
SFO1 500.2024010 MHz
NUC1 1H
P0 11.30 usec
P1 11.30 usec
P17 0 usec
PLW1 13.69999981 W
PLW10 2.58780003 W

====== GRADIENT CHANNEL =====
P16 1000.00 usec

F1 - Acquisition parameters
TD 512
SFO1 500.2024 MHz
FIDRES 8.829679 Hz
SW 9.038 ppm
FnMODE QF

F2 - Processing parameters
SI 2048
LB 0 Hz

F1 - Processing parameters
SI 512
MC2 QF
SF 500.2000000 MHz
WDW QSINE
SSB 0
LB 0 Hz
GB 0

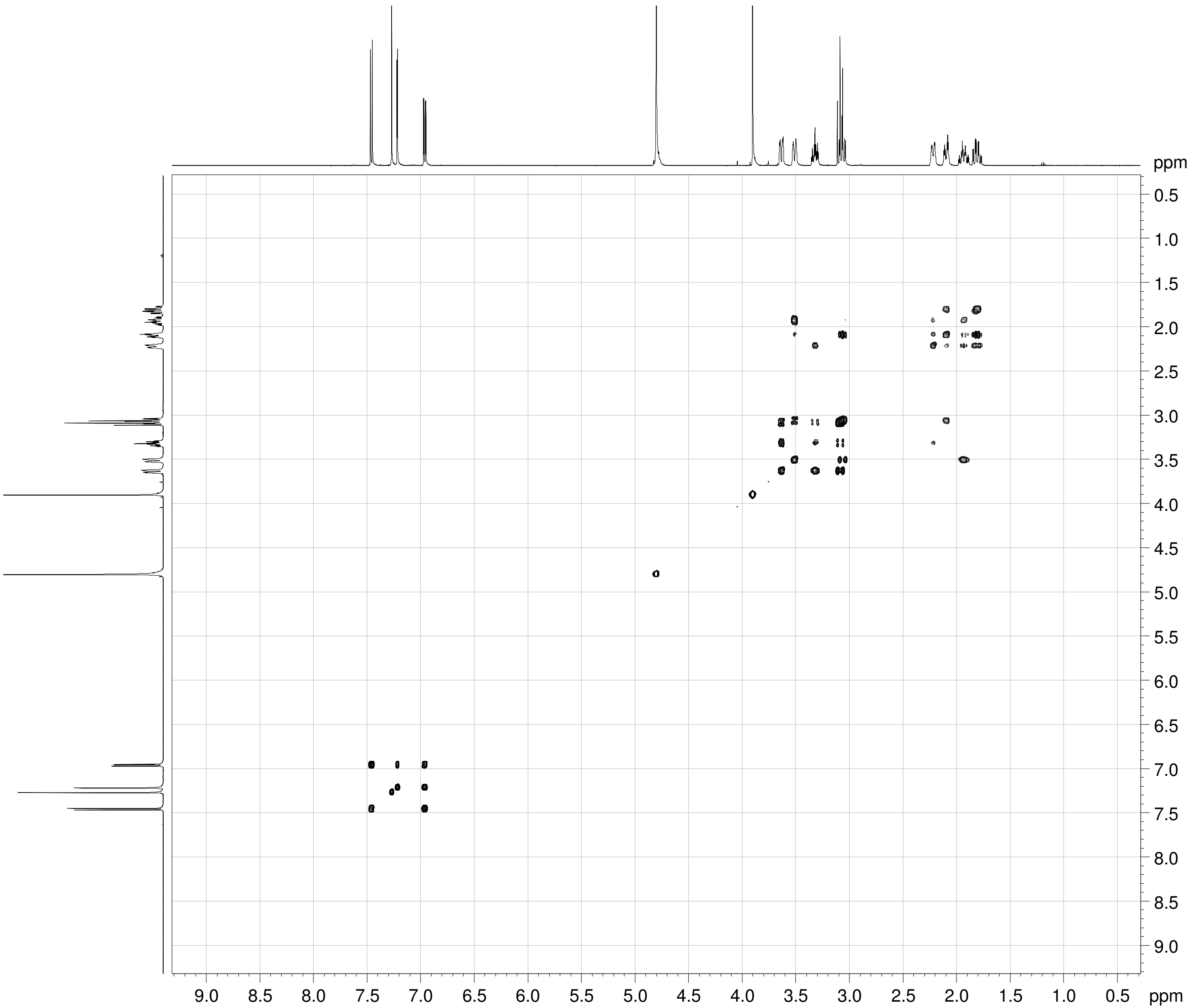

1001-COSY
A2

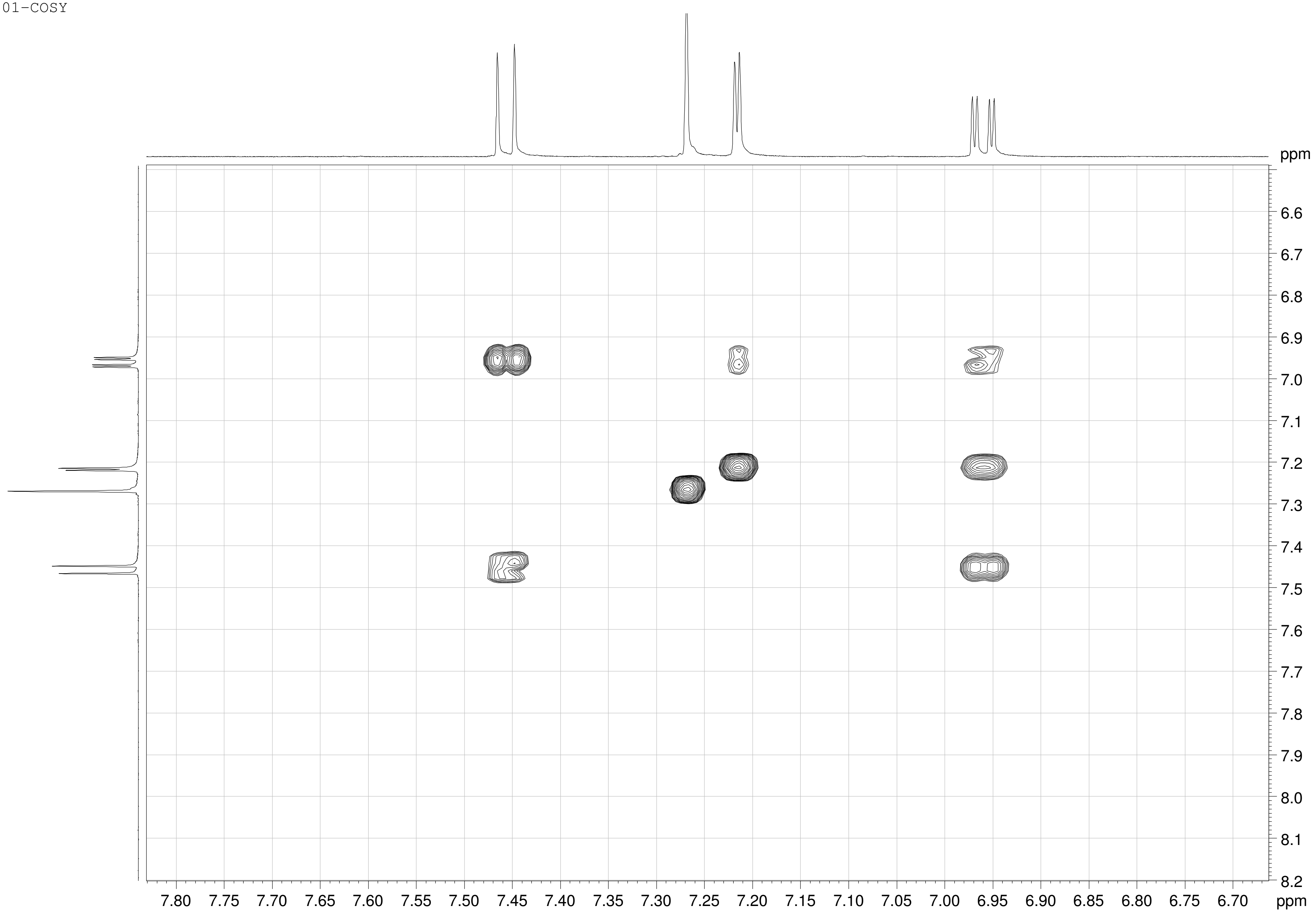
ppm
6.6
6.7
6.8
6.9
7.0
7.1
7.2
7.3
7.4
7.5
7.6
7.7
7.8
7.9
8.0
8.1
8.2
7.80 7.75 7.70 7.65 7.60 7.55 7.50 7.45 7.40 7.35 7.30 7.25 7.20 7.15 7.10 7.05 7.00 6.95 6.90 6.85 6.80 6.75 6.70
ppm

1001-COSY
A2

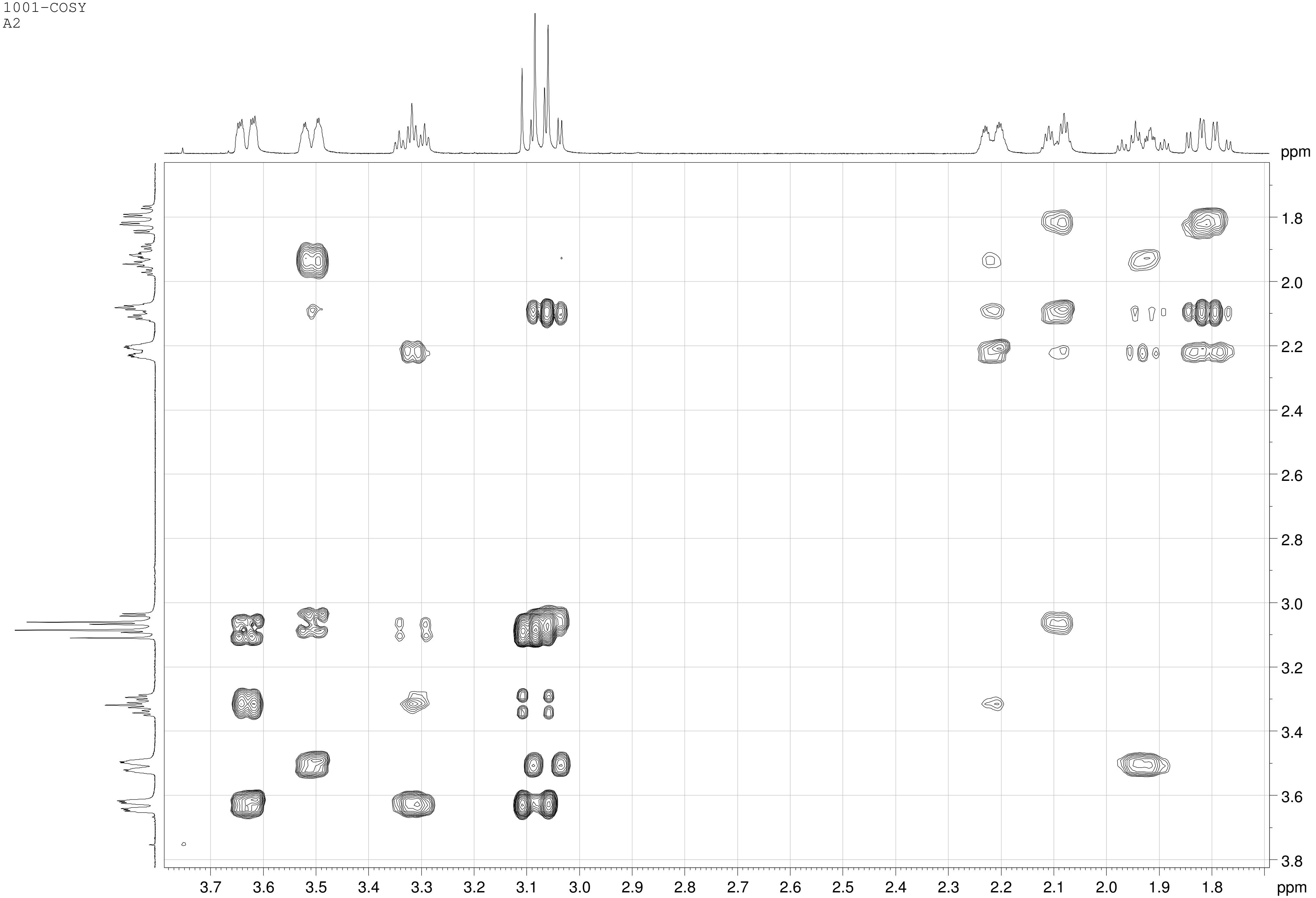
ppm
1.8
2.0
2.2
2.4
2.6
2.8
3.0
3.2
3.4
3.6
3.8
3.7 3.6 3.5 3.4 3.3 3.2 3.1 3.0 2.9 2.8 2.7 2.6 2.5 2.4 2.3 2.2 2.1 2.0 1.9 1.8
ppm

1001-HSQC
A2

```
Current Data Parameters
NAME                1001
EXPNO                  4
PROCNO                 1

F2 - Acquisition Parameters
Date_           20150424
PROBHD    5 mm CPPBBO BB
PULPROG  hsqcedetgpsisp2.3
SOLVENT              D2O
TE                 298.1 K
SWH             4485.646 Hz
AQ             0.1799072 sec
NS                     2
FIDRES          2.779211 Hz
D0            0.00000300 sec
D1            2.00000000 sec
D4            0.00172414 sec
D11           0.03000000 sec
D16           0.00020000 sec
D21           0.00360000 sec
D24           0.00089000 sec

======== CHANNEL f1 ========
SFO1         500.2022536 MHz
NUC1                  1H
P1                 11.30 usec
P2                 22.60 usec
P28      0 usec
PLW1         13.69999981 W

======== CHANNEL f2 ========
SFO2         125.7860290 MHz
NUC2                 13C
P3                 10.00 usec
P14               500.00 usec
P24              2000.00 usec
P31              1900.00 usec
PLW0     0 W
PLW2         55.00000000 W
PLW12         1.30180001 W

====== GRADIENT CHANNEL =====
P16              1000.00 usec
P19               600.00 usec

F1 - Acquisition parameters
TD                   512
SFO1             125.786 MHz
FIDRES         41.912552 Hz
SW               170.601 ppm
FnMODE      Echo-Antiecho

F2 - Processing parameters
SI                  2048
LB       0 Hz

F1 - Processing parameters
SI                   512
MC2         echo-antiecho
SF           125.7753924 MHz
WDW                QSINE
SSB                    2
LB       0 Hz
```

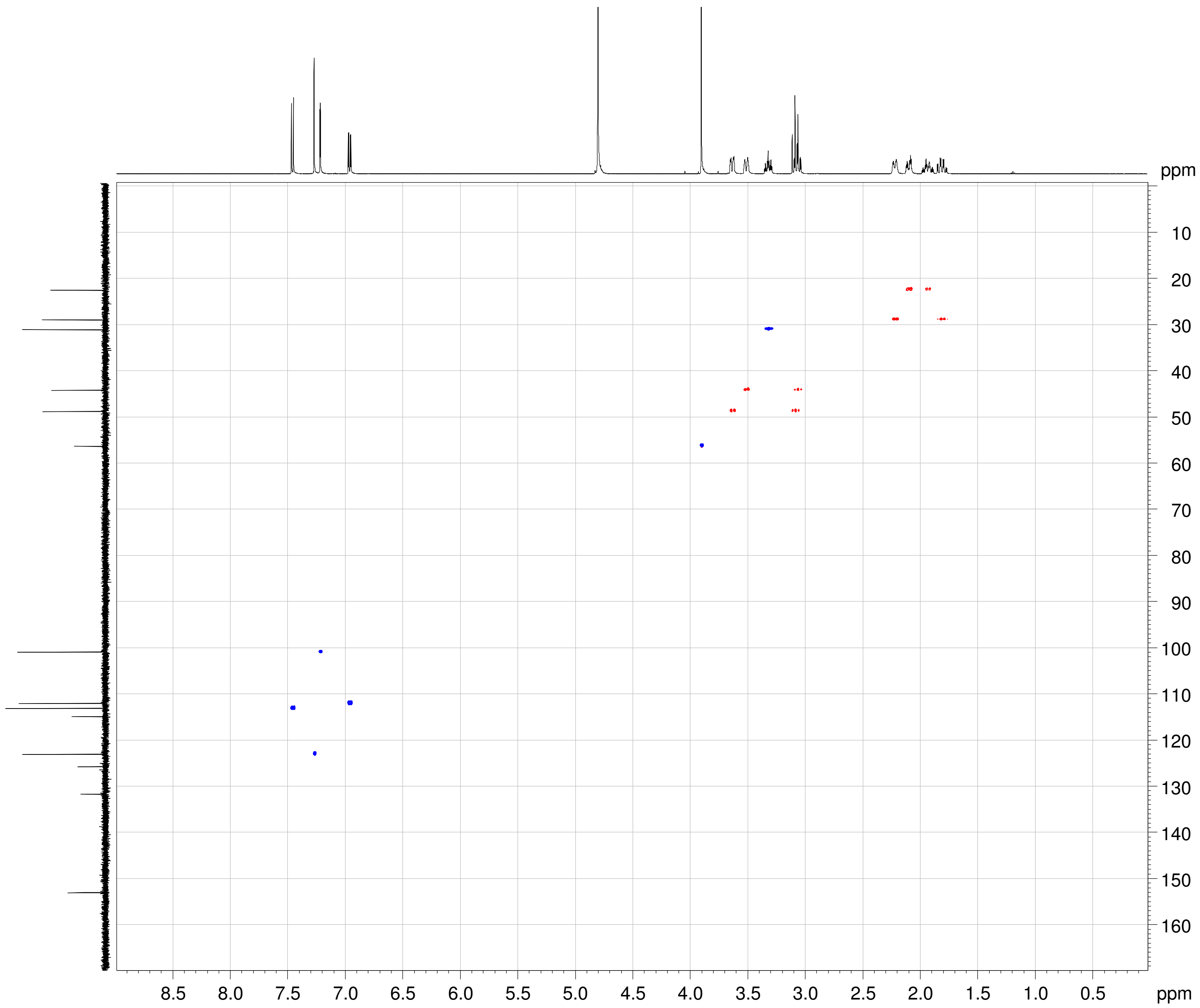

1001-HSQC
A2

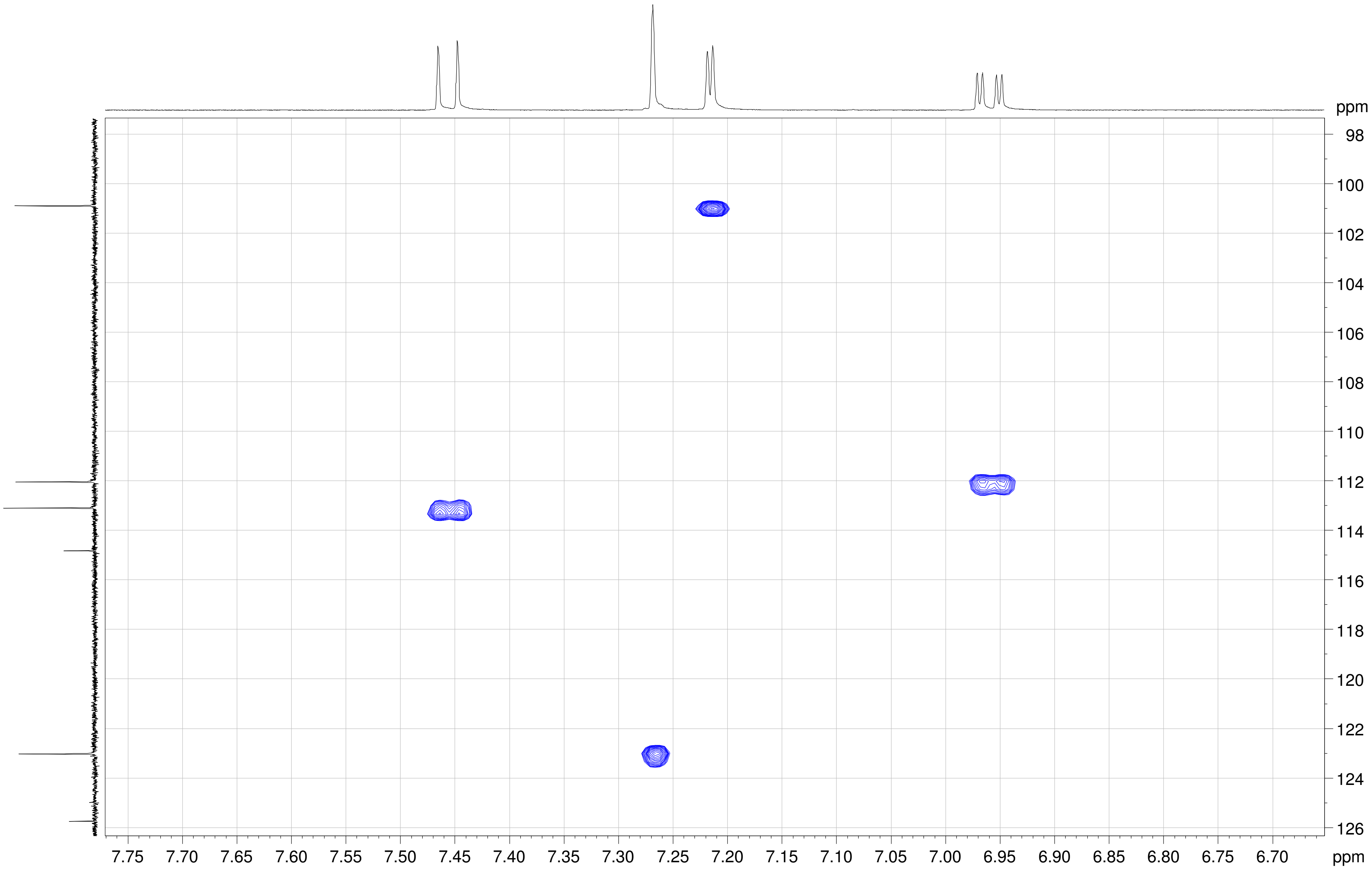

ppm
98
100
102
104
106
108
110
112
114
116
118
120
122
124
126
7.75 7.70 7.65 7.60 7.55 7.50 7.45 7.40 7.35 7.30 7.25 7.20 7.15 7.10 7.05 7.00 6.95 6.90 6.85 6.80 6.75 6.70
ppm

1001-HSQC
A2

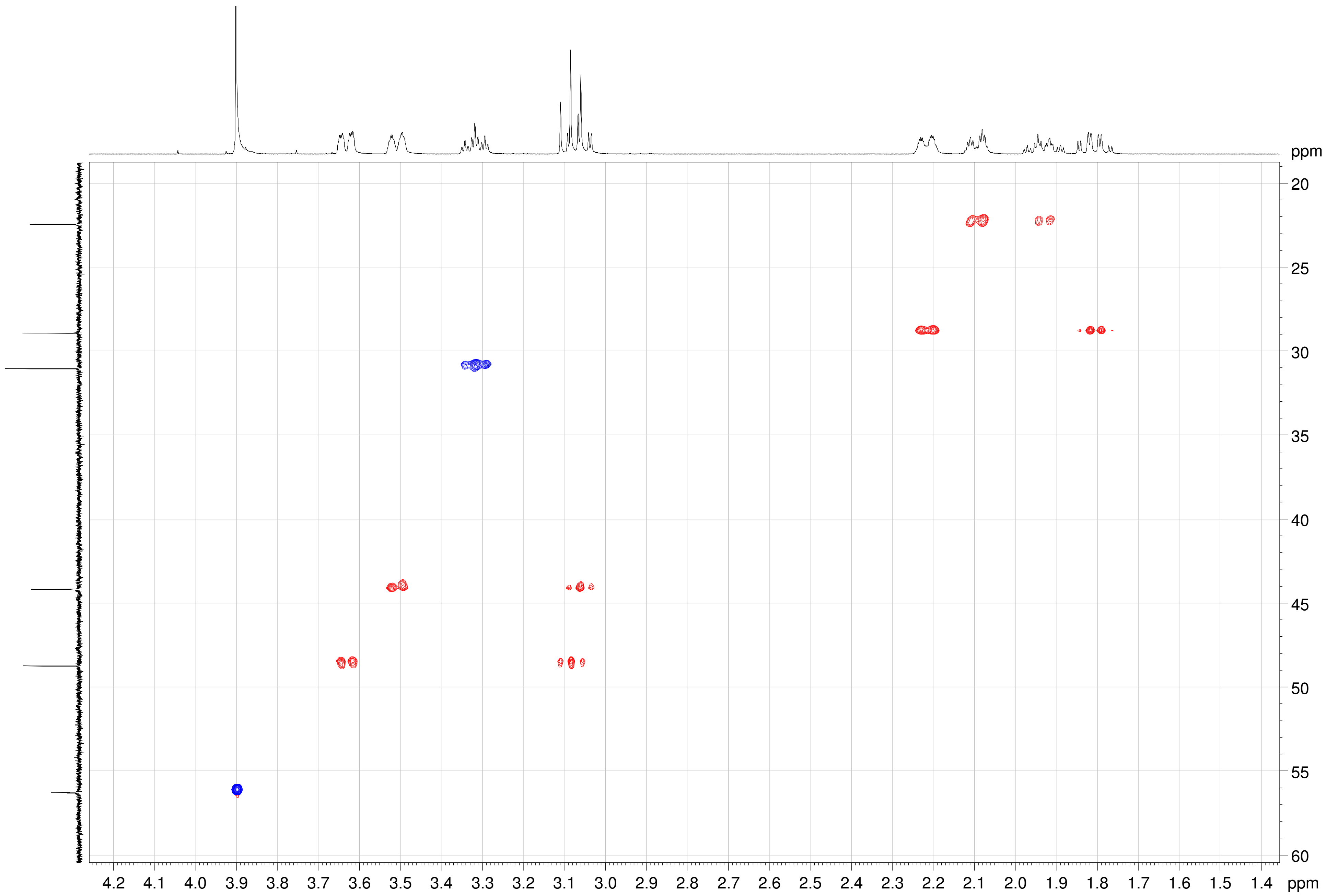
ppm
20
25
30
35
40
45
50
55
60
4.2 4.1 4.0 3.9 3.8 3.7 3.6 3.5 3.4 3.3 3.2 3.1 3.0 2.9 2.8 2.7 2.6 2.5 2.4 2.3 2.2 2.1 2.0 1.9 1.8 1.7 1.6 1.5 1.4
ppm

(R)-12c

4503-1H
Cyk.E

Current Data Parameters
NAME 4503
EXPNO 1
PROCNO 1

F2 - Acquisition Parameters
Date_ 20180122
PROBHD 5 mm CPPBBO BB
PULPROG zg30
SOLVENT MeOD
TE 298.1 K
SWH 10000.000 Hz
AQ 3.2767999 sec
NS 8
FIDRES 0.152588 Hz
D1 0 sec

======== CHANNEL f1 ========
SFO1 500.2030889 MHz
NUC1 1H
P1 11.30 usec
PLW1 13.69999981 W

F2 - Processing parameters
SI 65536
LB 0 Hz

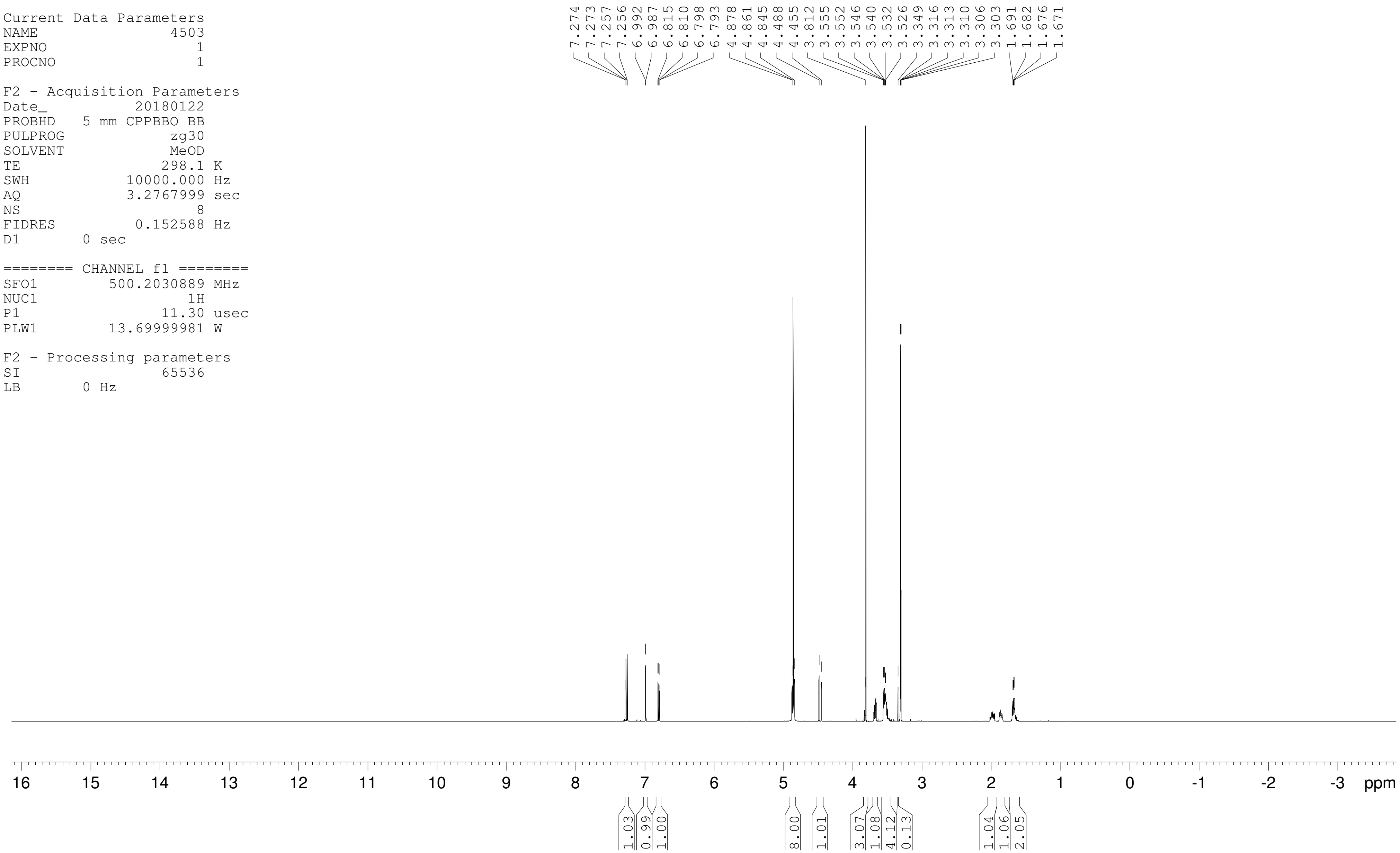

4503-1H
Cyk.E

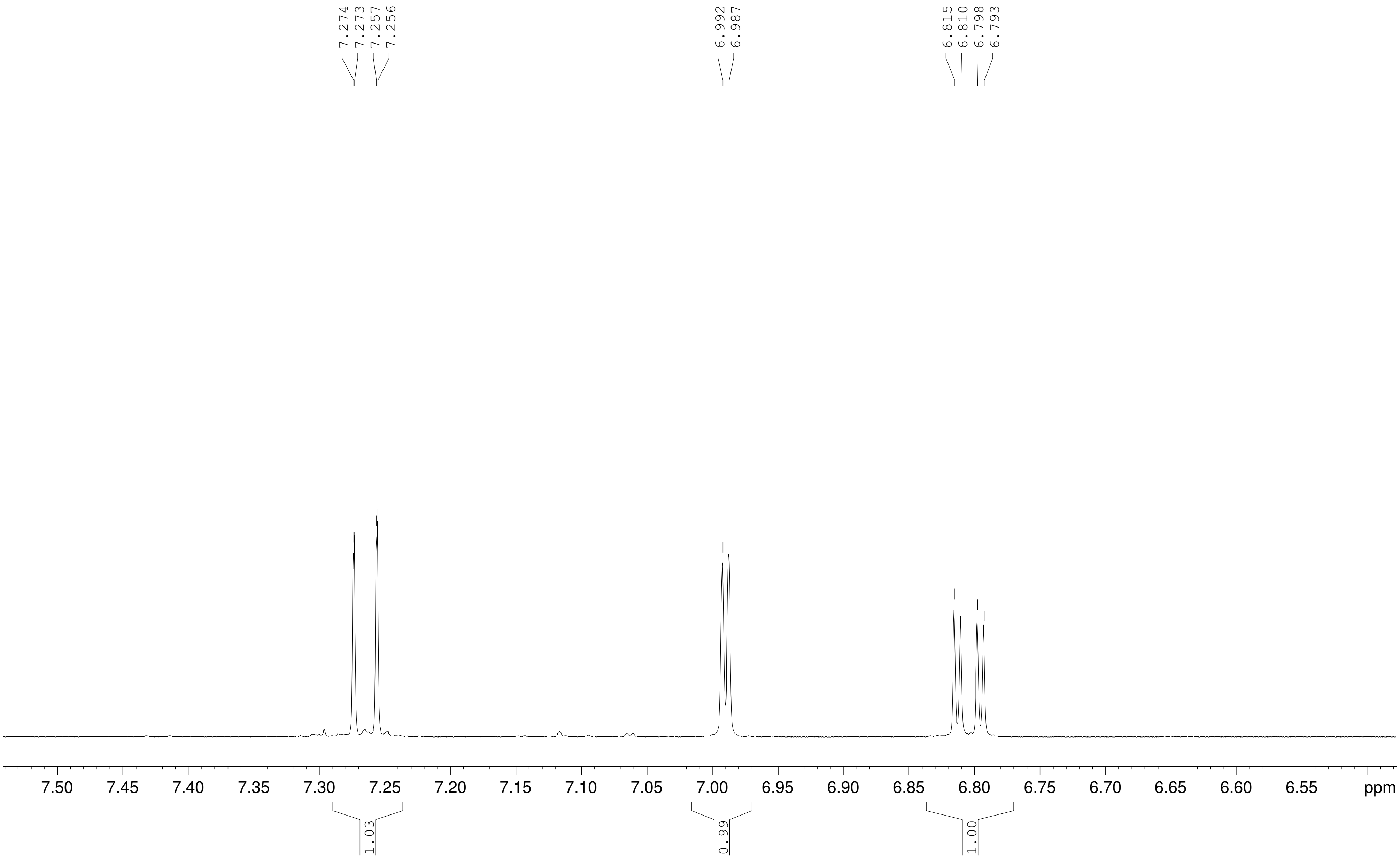

7.274
7.273
7.257
7.256
6.992
6.987
6.815
6.810
6.798
6.793
7.50
7.45
7.40
7.35
7.30
7.25
7.20
7.15
7.10
7.05
7.00
6.95
6.90
6.85
6.80
6.75
6.70
6.65
6.60
6.55
ppm
1.03
0.99
1.00

4503-1H
Cyk.E

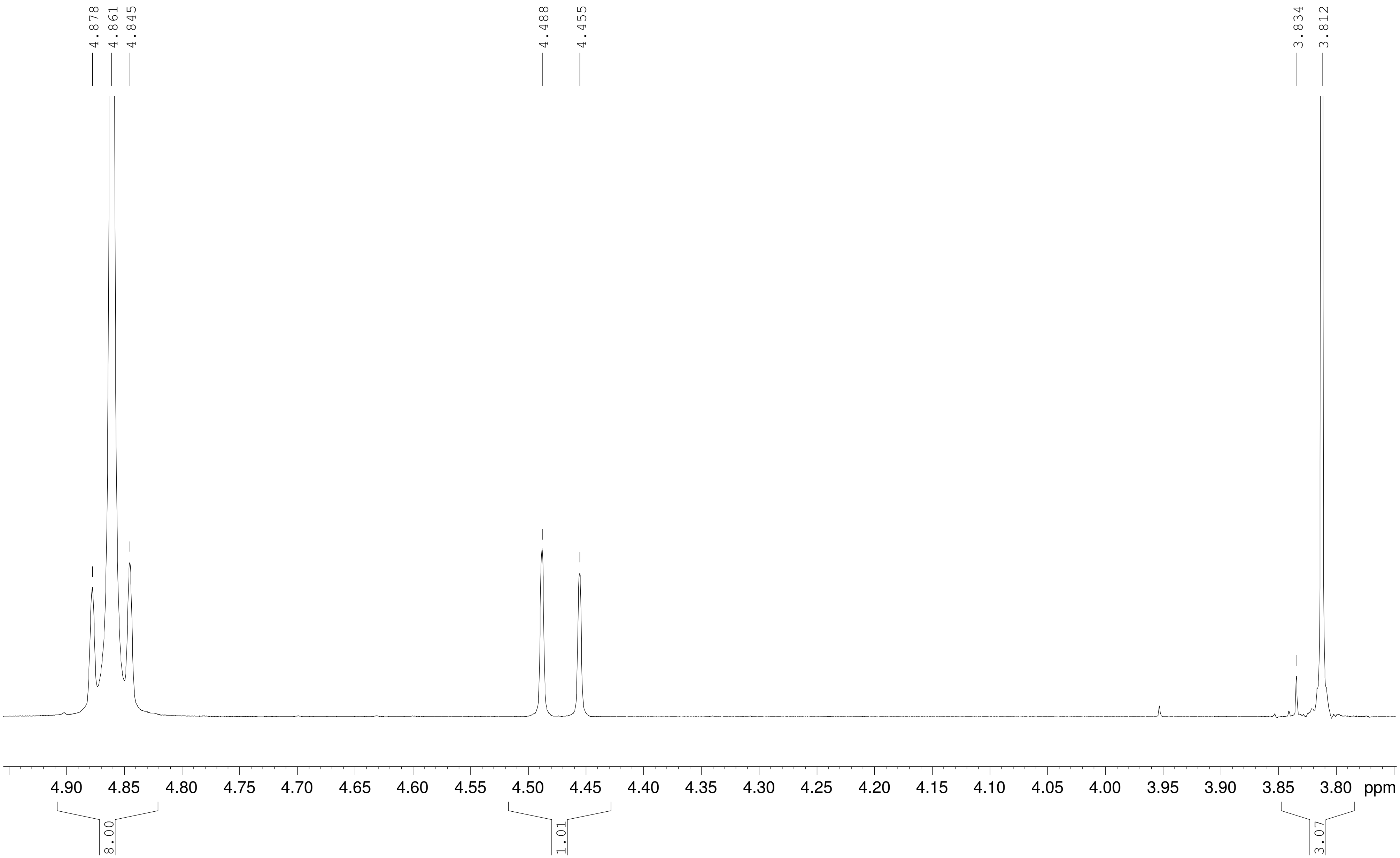

4.878
4.861
4.845
4.488
4.455
3.834
3.812
4.90 4.85 4.80 4.75 4.70 4.65 4.60 4.55 4.50 4.45 4.40 4.35 4.30 4.25 4.20 4.15 4.10 4.05 4.00 3.95 3.90 3.85 3.80 ppm
8.00
1.01
3.07

4503-1H
Cyk.E

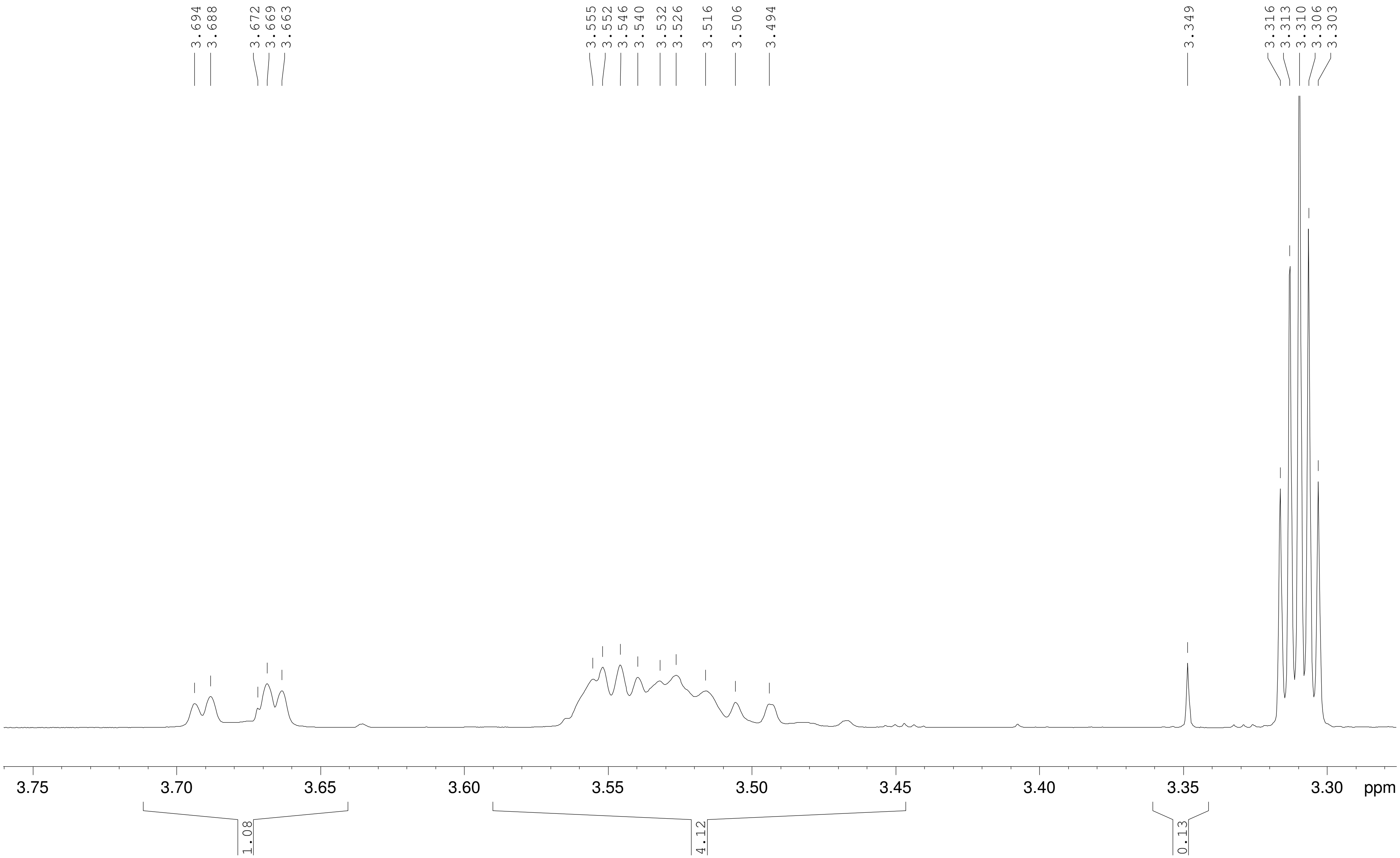
3.694
3.688
3.672
3.669
3.663
3.555
3.552
3.546
3.540
3.532
3.526
3.516
3.506
3.494
3.349
3.316
3.313
3.310
3.306
3.303
3.75
3.70
3.65
3.60
3.55
3.50
3.45
3.40
3.35
3.30
ppm
1.08
4.12
0.13

4503-1H
Cyk.E

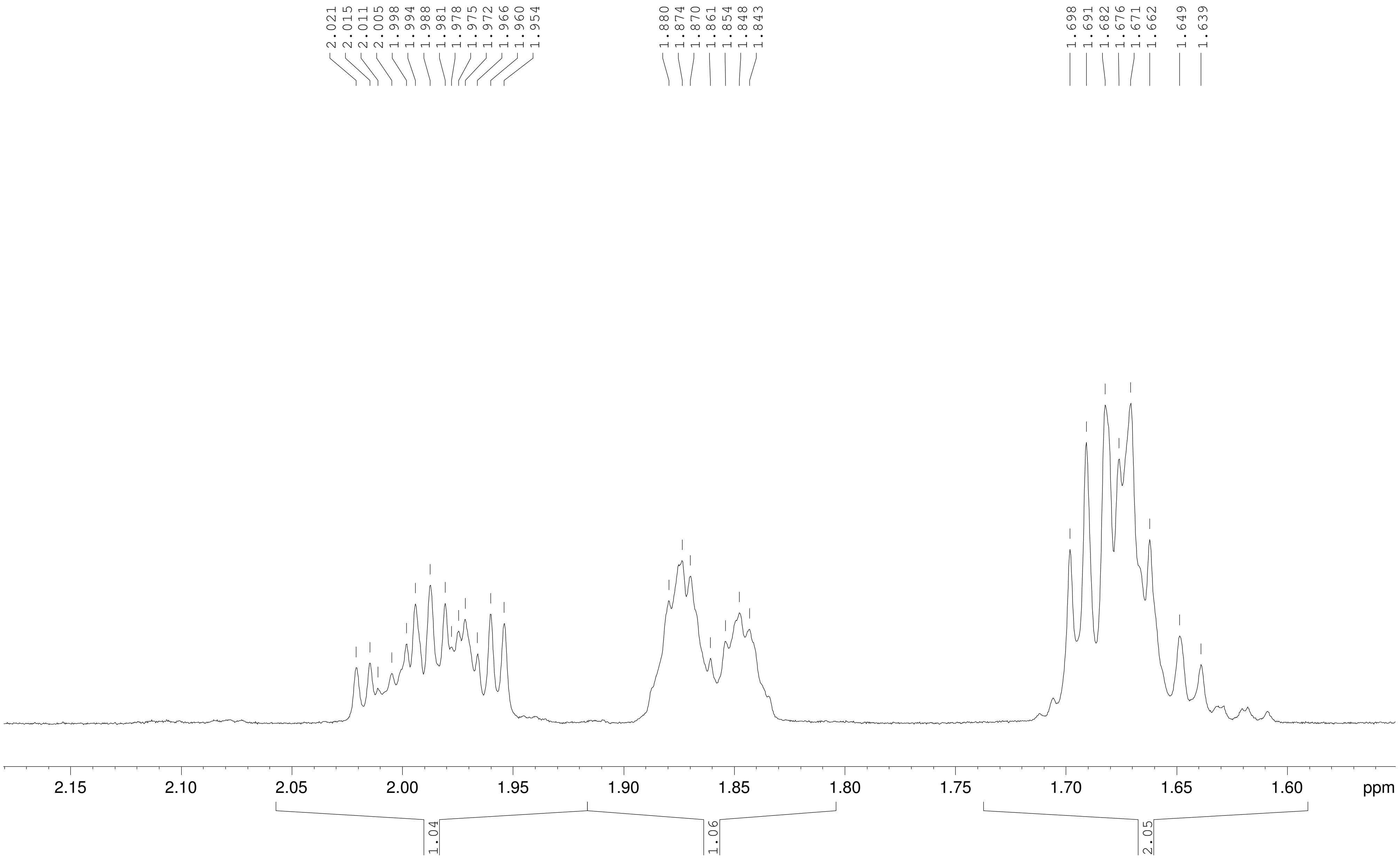

2.021
2.015
2.011
2.005
1.998
1.994
1.988
1.981
1.978
1.975
1.972
1.966
1.960
1.954
1.880
1.874
1.870
1.861
1.854
1.848
1.843
1.698
1.691
1.682
1.676
1.671
1.662
1.649
1.639
2.15
2.10
2.05
2.00
1.95
1.90
1.85
1.80
1.75
1.70
1.65
1.60
ppm
1.04
1.06
2.05

4503-13C
Cyk.E

Current Data Parameters
NAME 4503
EXPNO 2
PROCNO 1

F2 - Acquisition Parameters
Date_ 20180122
PROBHD 5 mm CPPBBO BB
PULPROG zg_pi_CPD
SOLVENT MeOD
TE 298.1 K
SWH 31249.381 Hz
AQ 1.0485969 sec
NS 376
FIDRES 0.476828 Hz
D1 1.50000000 sec
D11 0.03000000 sec
D14 0.00050000 sec

======== CHANNEL f1 ========
SFO1 125.7904831 MHz
NUC1 13C
P1 10.00 usec
P2 20.00 usec
PLW1 55.00000000 W

======== CHANNEL f2 ========
SFO2 500.2020008 MHz
NUC2 1H
PLW2 13.00000000 W
PLW12 0.25937000 W

F2 - Processing parameters
SI 65536
LB 0.50 Hz

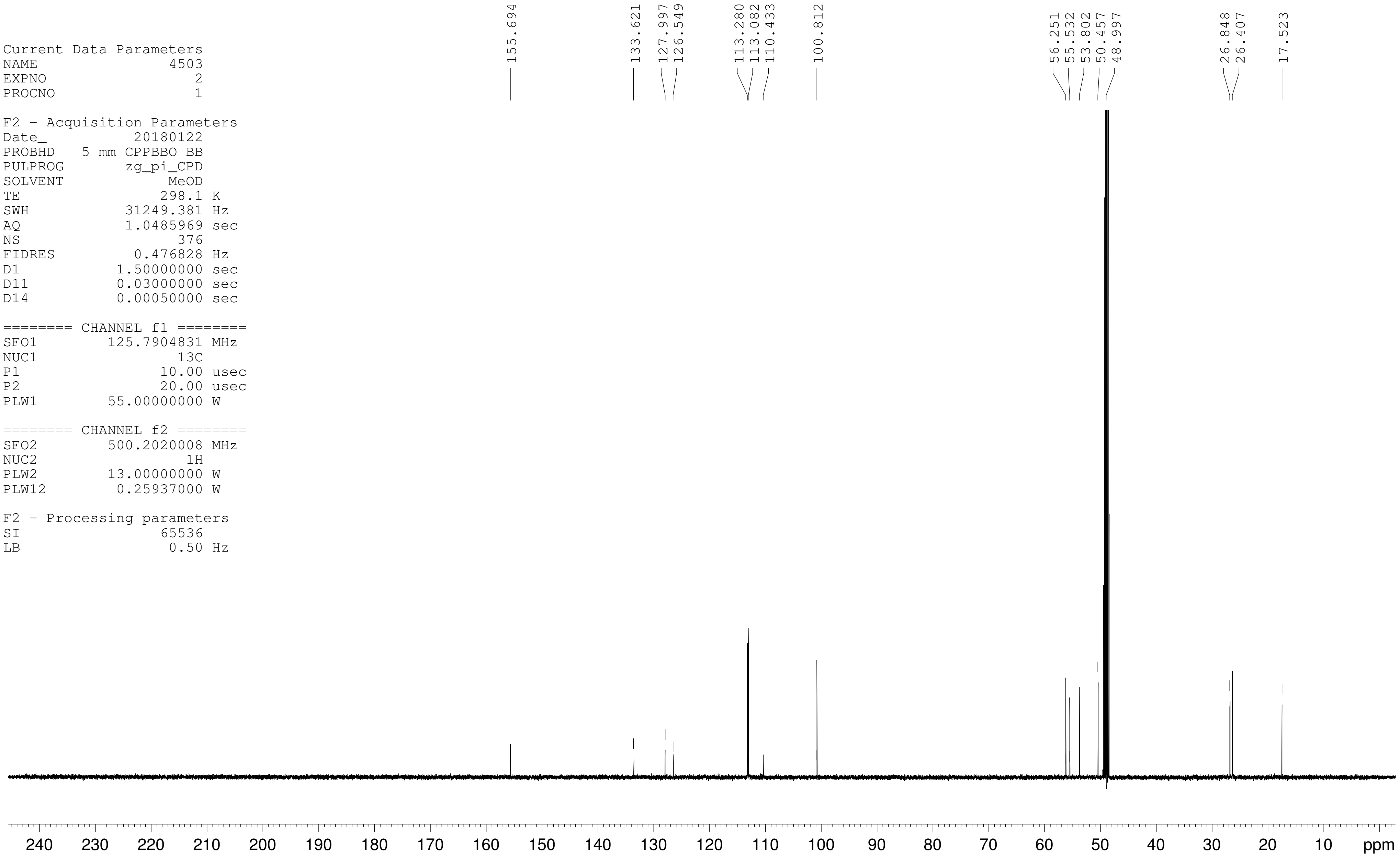

4503-13C
Cyk.E

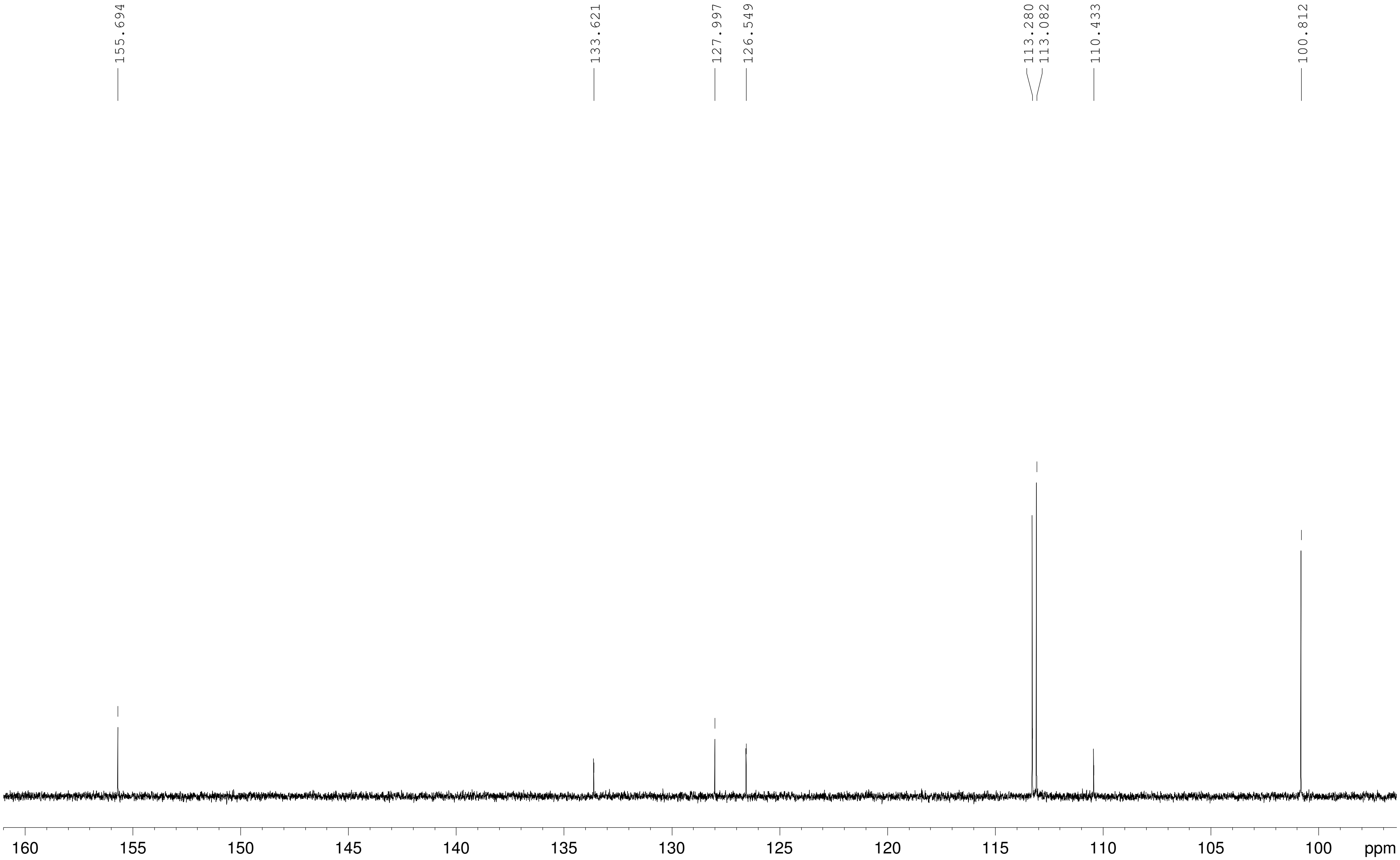

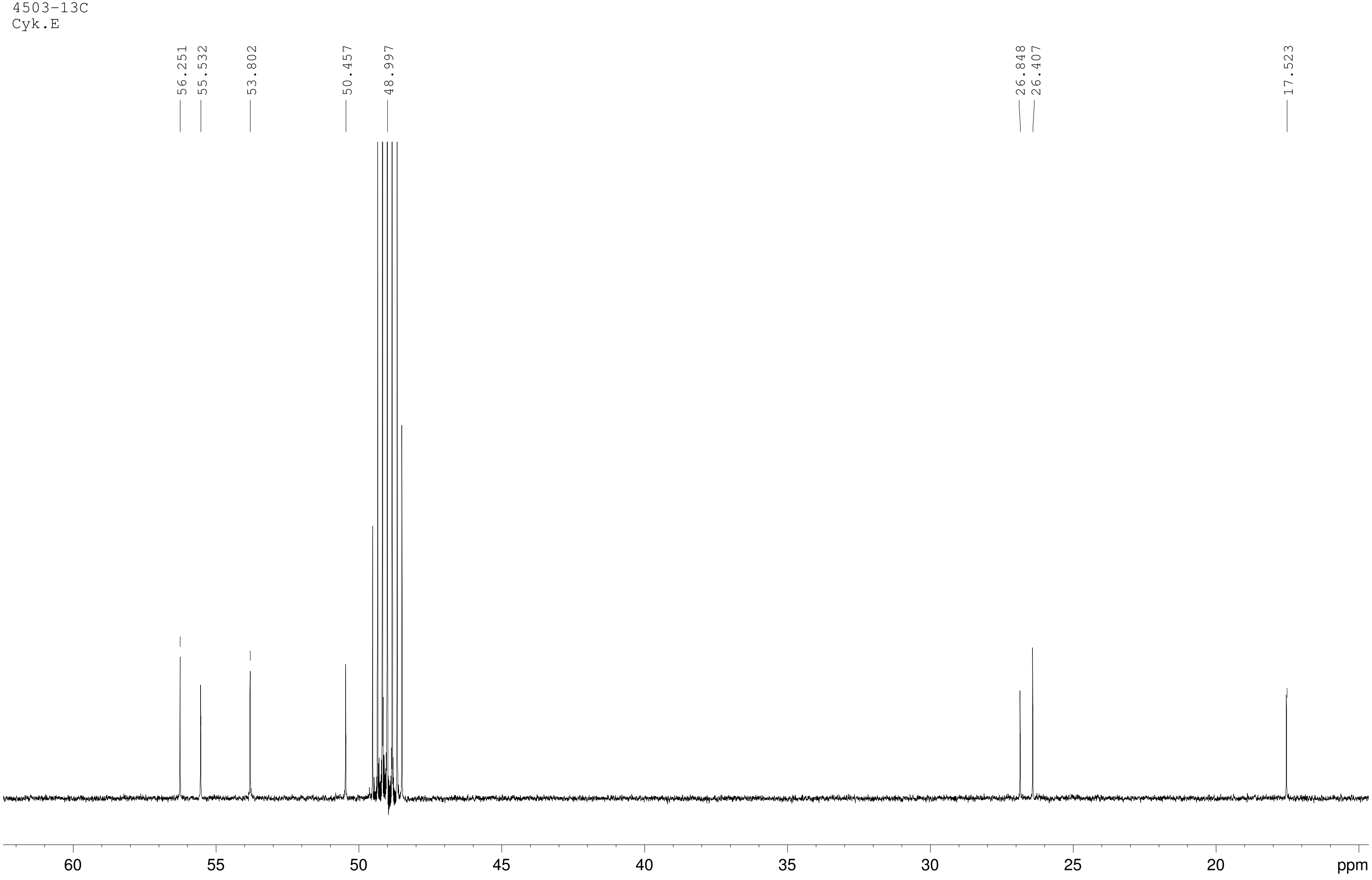
4503-13C
Cyk.E
56.251
55.532
53.802
50.457
48.997
26.848
26.407
17.523
60
55
50
45
40
35
30
25
20
ppm

4503-COSY
Cyk.E

```
Current Data Parameters
NAME                 4503
EXPNO                   3
PROCNO                  1

F2 - Acquisition Parameters
Date_          20180122
PROBHD   5 mm CPPBBO BB
PULPROG       cosygpppqf
SOLVENT            MeOD
TE                298.1 K
SWH            4761.905 Hz
AQ            0.1999200 sec
NS                    1
FIDRES         2.501000 Hz
D0           0.00000300 sec
D1           2.00000000 sec
D11          0.03000000 sec
D12          0.00002000 sec
D13          0.00000400 sec
D16          0.00020000 sec

======== CHANNEL f1 ========
SFO1        500.2021430 MHz
NUC1                 1H
P0                11.30 usec
P1                11.30 usec
P17      0 usec
PLW1        13.69999981 W
PLW10        2.58780003 W

====== GRADIENT CHANNEL =====
P16             1000.00 usec

F1 - Acquisition parameters
TD                  128
SFO1           500.2021 MHz
FIDRES        37.202381 Hz
SW                9.520 ppm
FnMODE               QF

F2 - Processing parameters
SI                 2048
LB       0 Hz

F1 - Processing parameters
SI                 1024
MC2                  QF
SF          500.2000147 MHz
WDW               QSINE
SSB      0
LB       0 Hz
GB       0
```

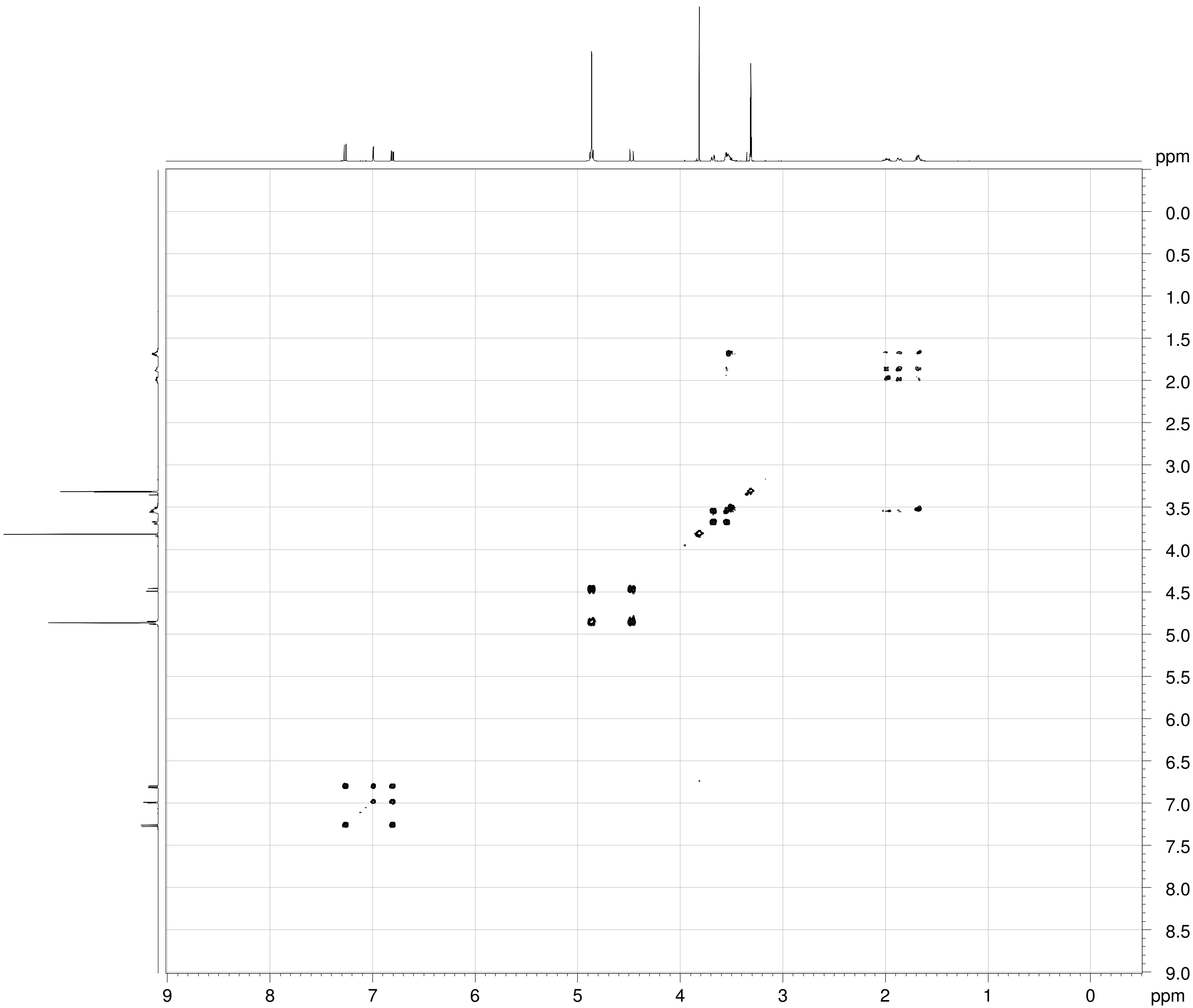

4503-COSY
Cyk.E

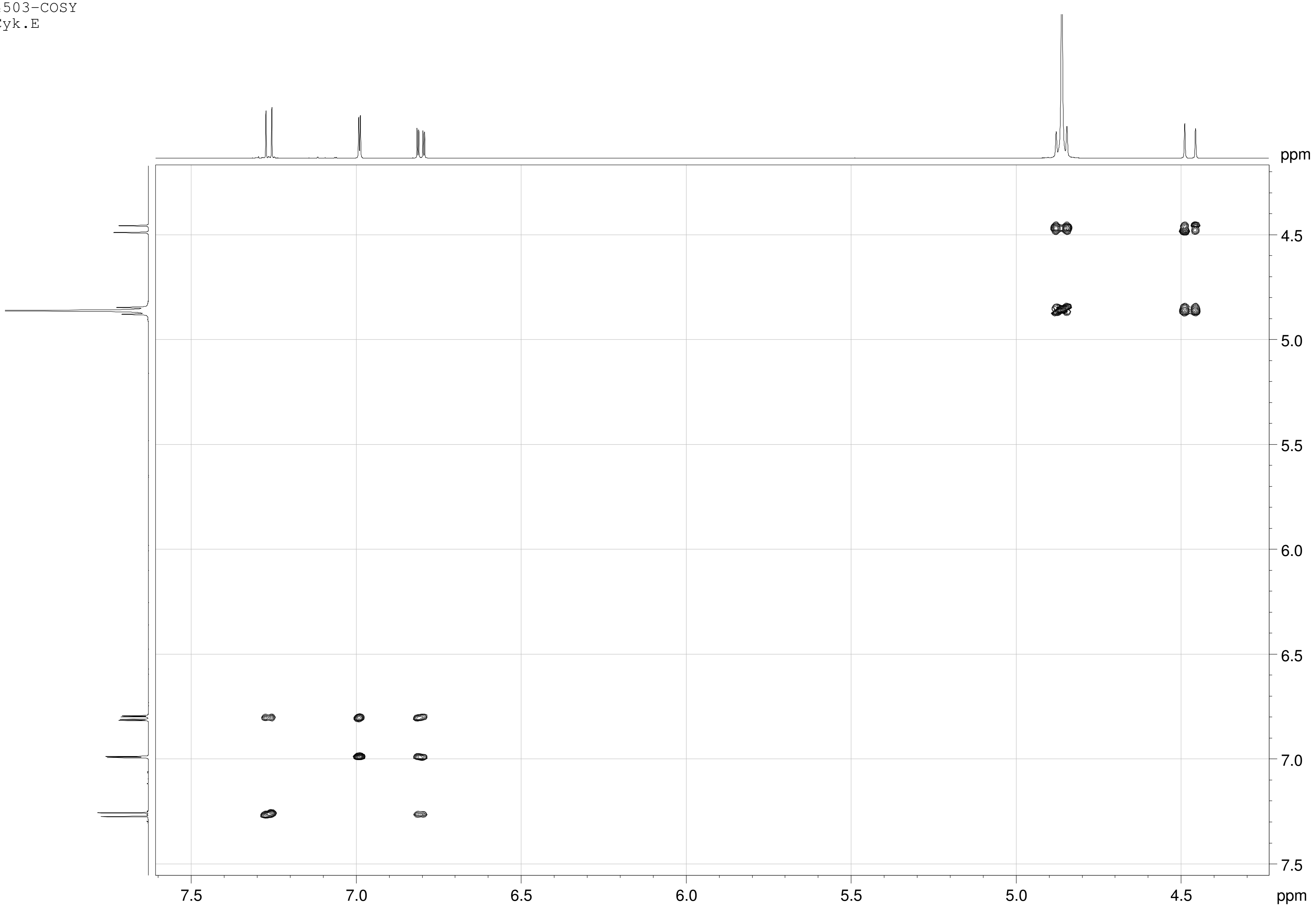

4503-COSY
Cyk.E

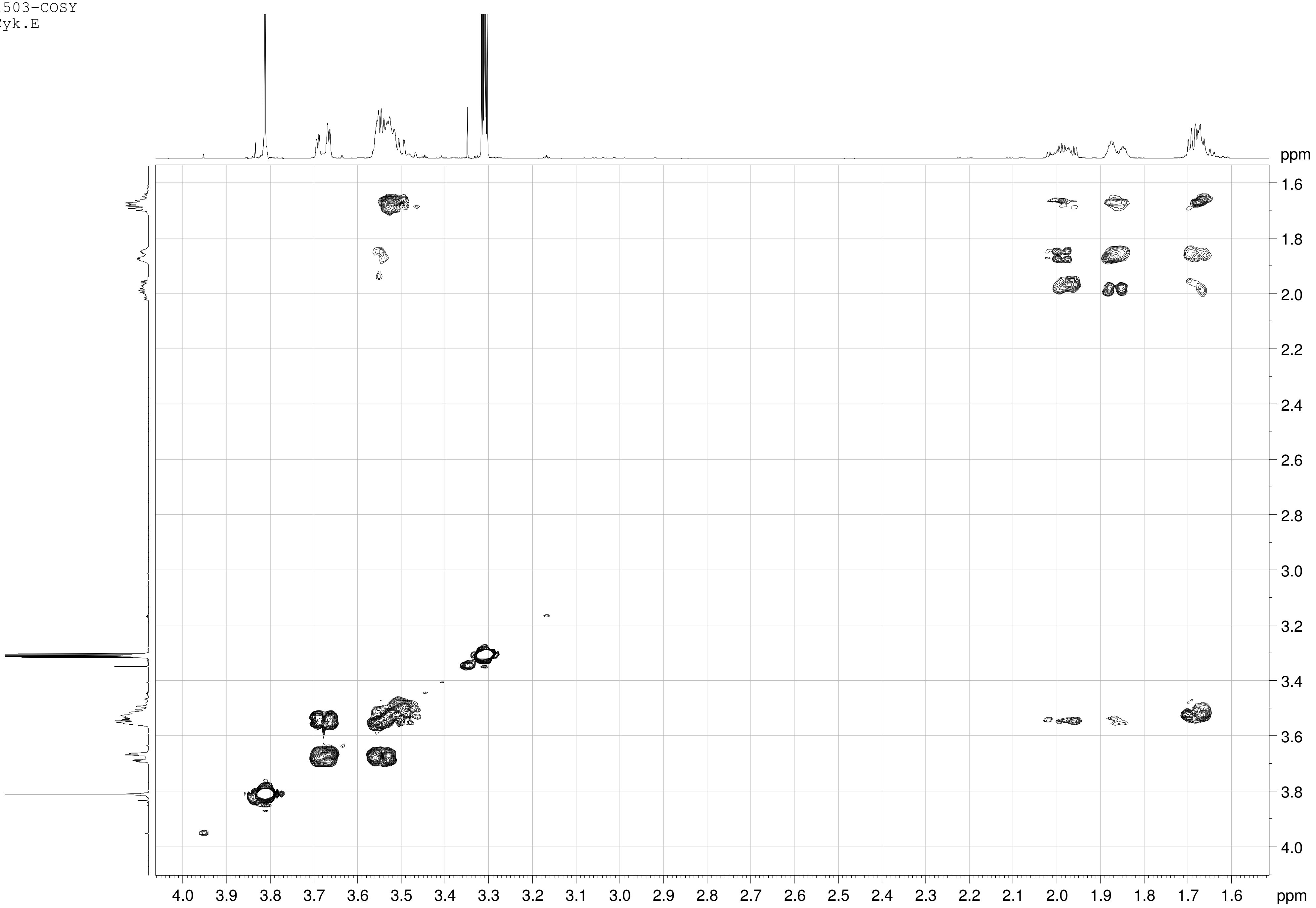

4503-HSQC
Cyk.E

Current Data Parameters
NAME 4503
EXPNO 4
PROCNO 1

F2 - Acquisition Parameters
Date_ 20180122
PROBHD 5 mm CPPBBO BB
PULPROG hsqcedetgpsisp2.3
SOLVENT MeOD
TE 298.1 K
SWH 4761.905 Hz
AQ 0.2150400 sec
NS 2
FIDRES 2.325149 Hz
D0 0.00000300 sec
D1 2.00000000 sec
D4 0.00172414 sec
D11 0.03000000 sec
D16 0.00020000 sec
D21 0.00360000 sec
D24 0.00089000 sec

======== CHANNEL f1 ========
SFO1 500.2021430 MHz
NUC1 1H
P1 11.30 usec
P2 22.60 usec
P28 0 usec
PLW1 13.69999981 W

======== CHANNEL f2 ========
SFO2 125.7865857 MHz
NUC2 13C
P3 10.00 usec
P14 500.00 usec
P24 2000.00 usec
P31 1900.00 usec
PLW0 0 W
PLW2 55.00000000 W
PLW12 1.30180001 W

====== GRADIENT CHANNEL =====
P16 1000.00 usec
P19 600.00 usec

F1 - Acquisition parameters
TD 128
SFO1 125.7866 MHz
FIDRES 180.011520 Hz
SW 183.179 ppm
FnMODE Echo-Antiecho

F2 - Processing parameters

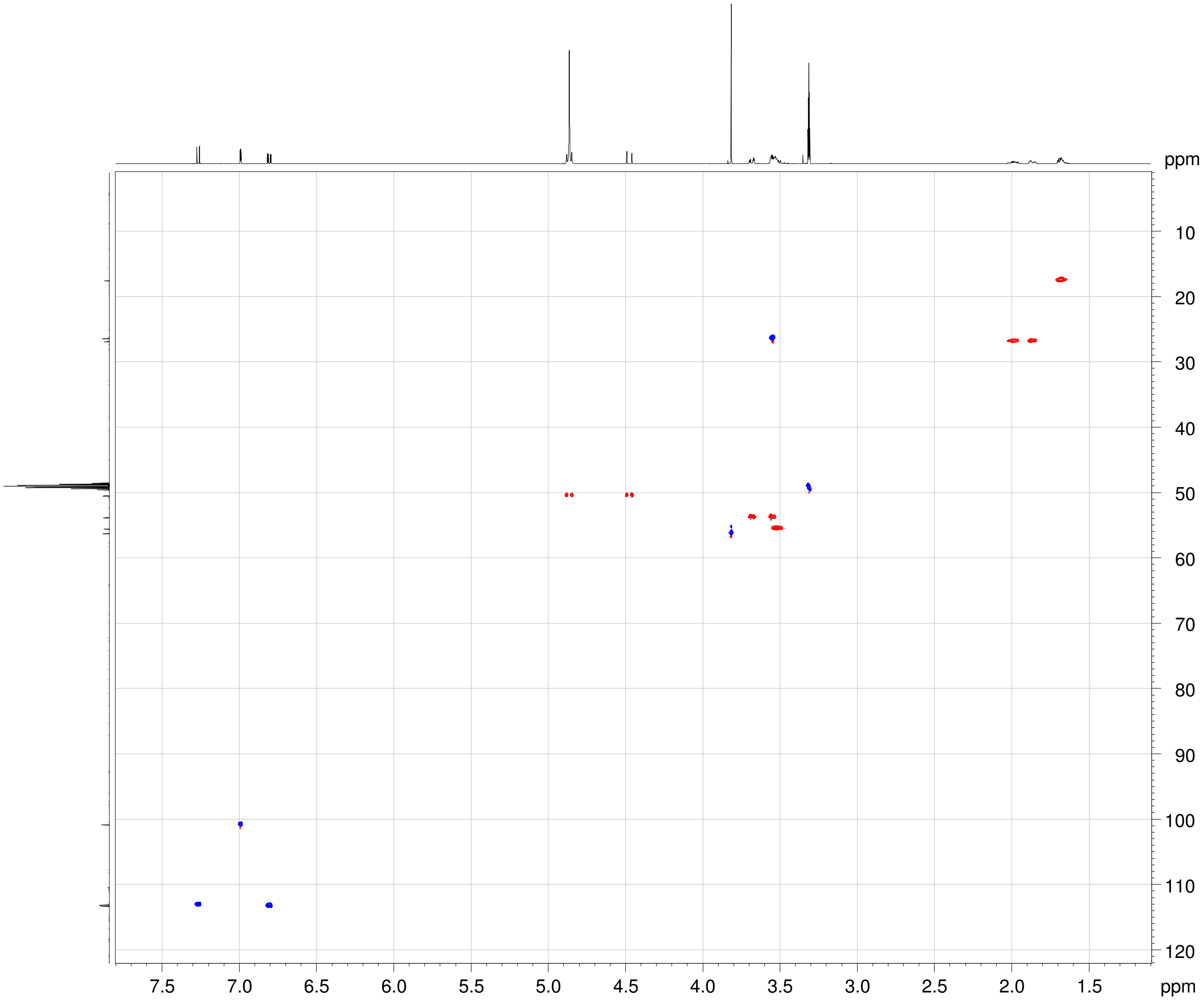

4503-HSQC
Cyk.E

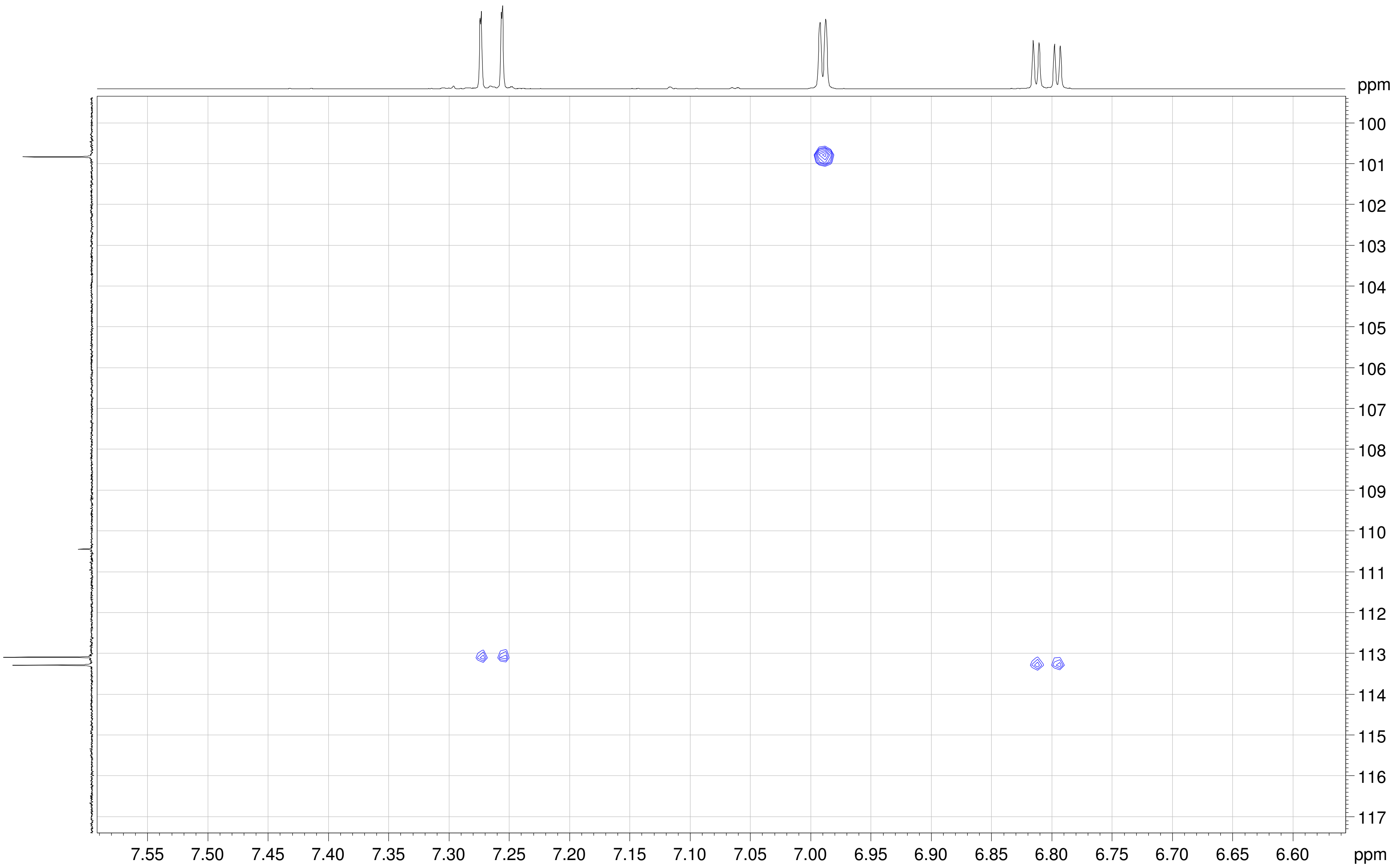

4503-HSQC
Cyk.E

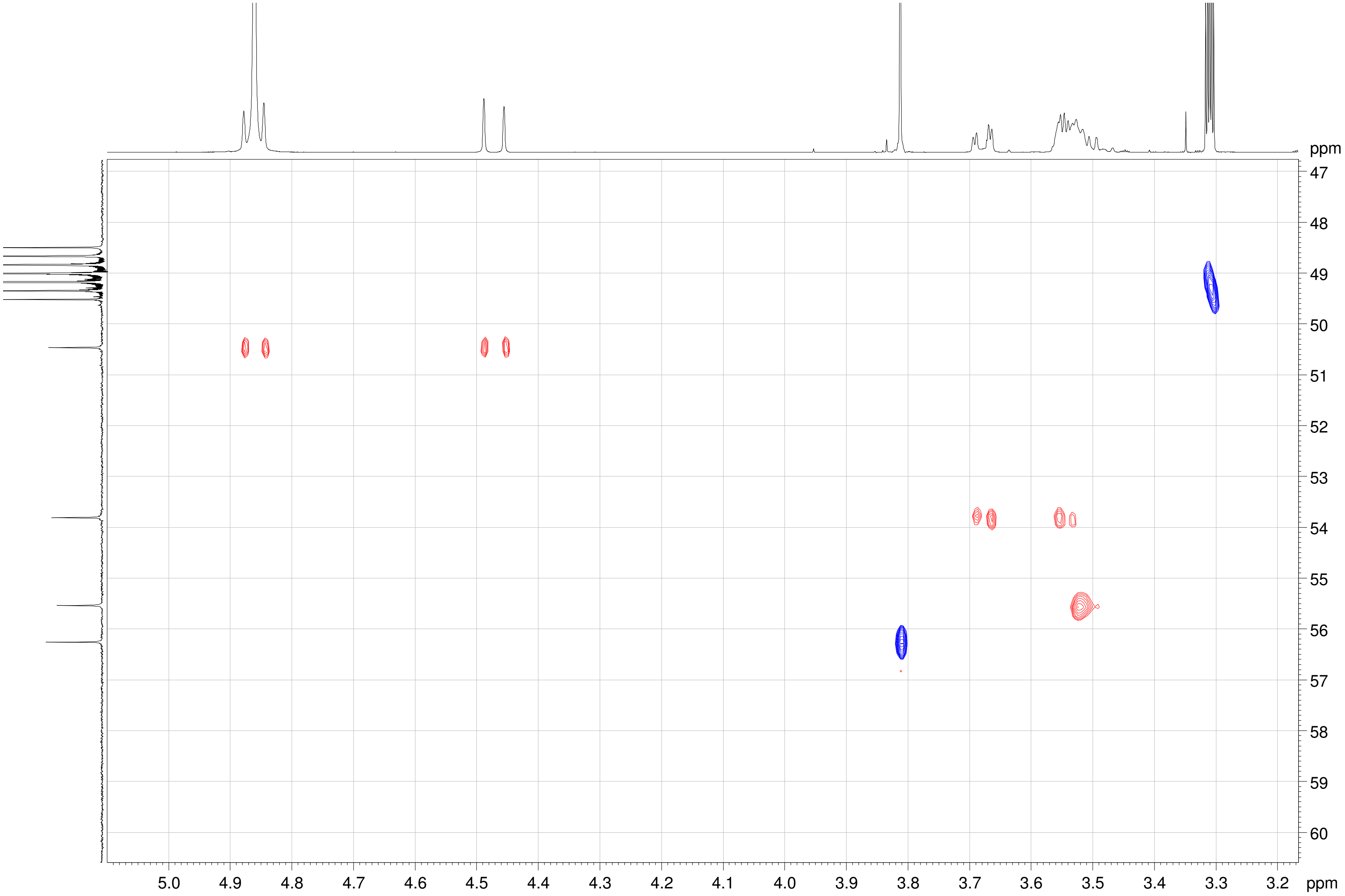

4503-HSQC
Cyk.E

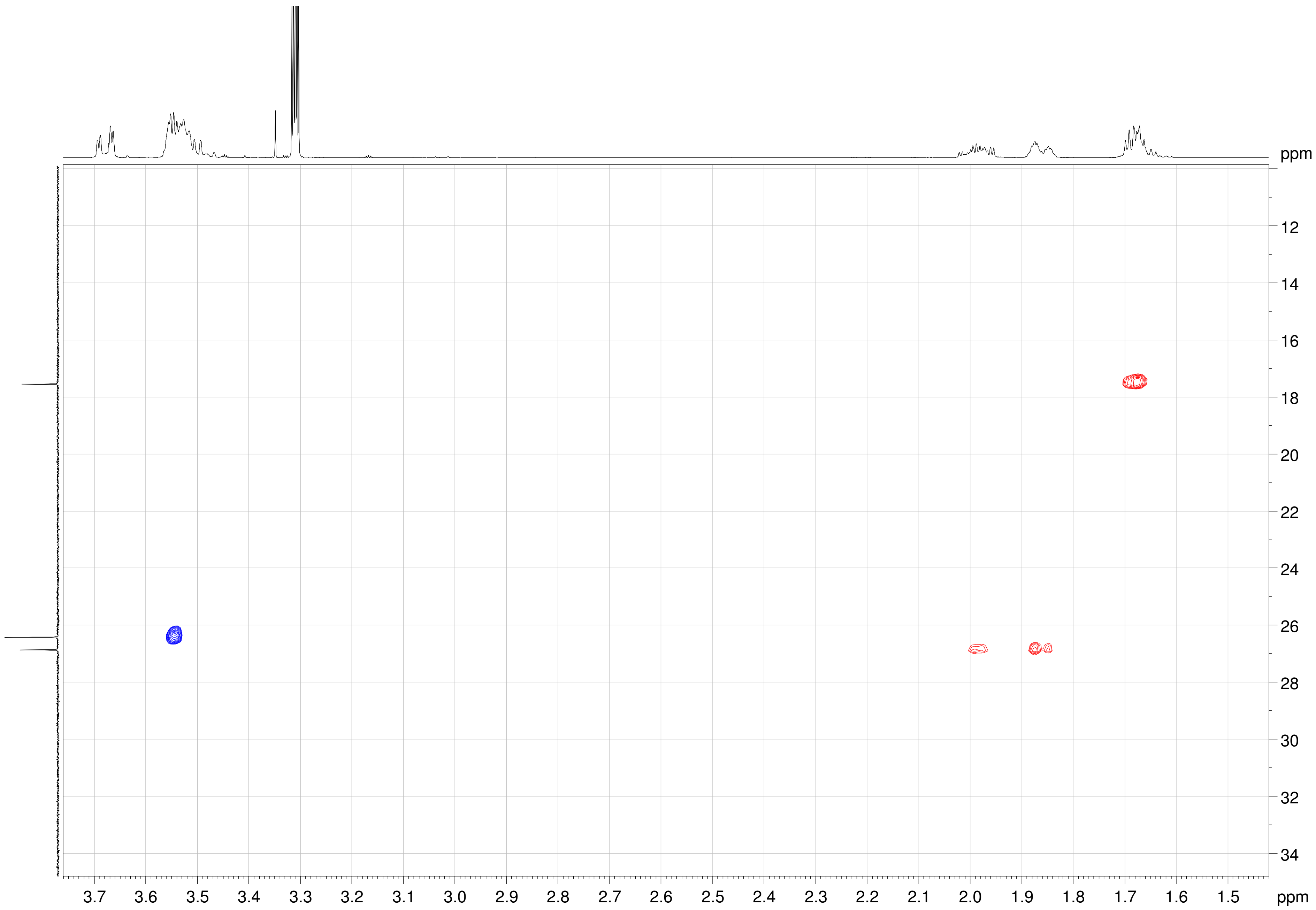

(S)-13c

4934-1H
T-F x HCl

Current Data Parameters
NAME 4934
EXPNO 1
PROCNO 1

F2 - Acquisition Parameters
Date_ 20180529
PROBHD 5 mm CPPBBO BB
PULPROG zg30
SOLVENT MeOD
TE 298.2 K
SWH 10000.000 Hz
AQ 3.2767999 sec
NS 8
FIDRES 0.152588 Hz
D1 0 sec

======== CHANNEL f1 ========
SFO1 500.2030889 MHz
NUC1 1H
P1 11.30 usec
PLW1 13.69999981 W

F2 - Processing parameters
SI 65536
LB 0 Hz

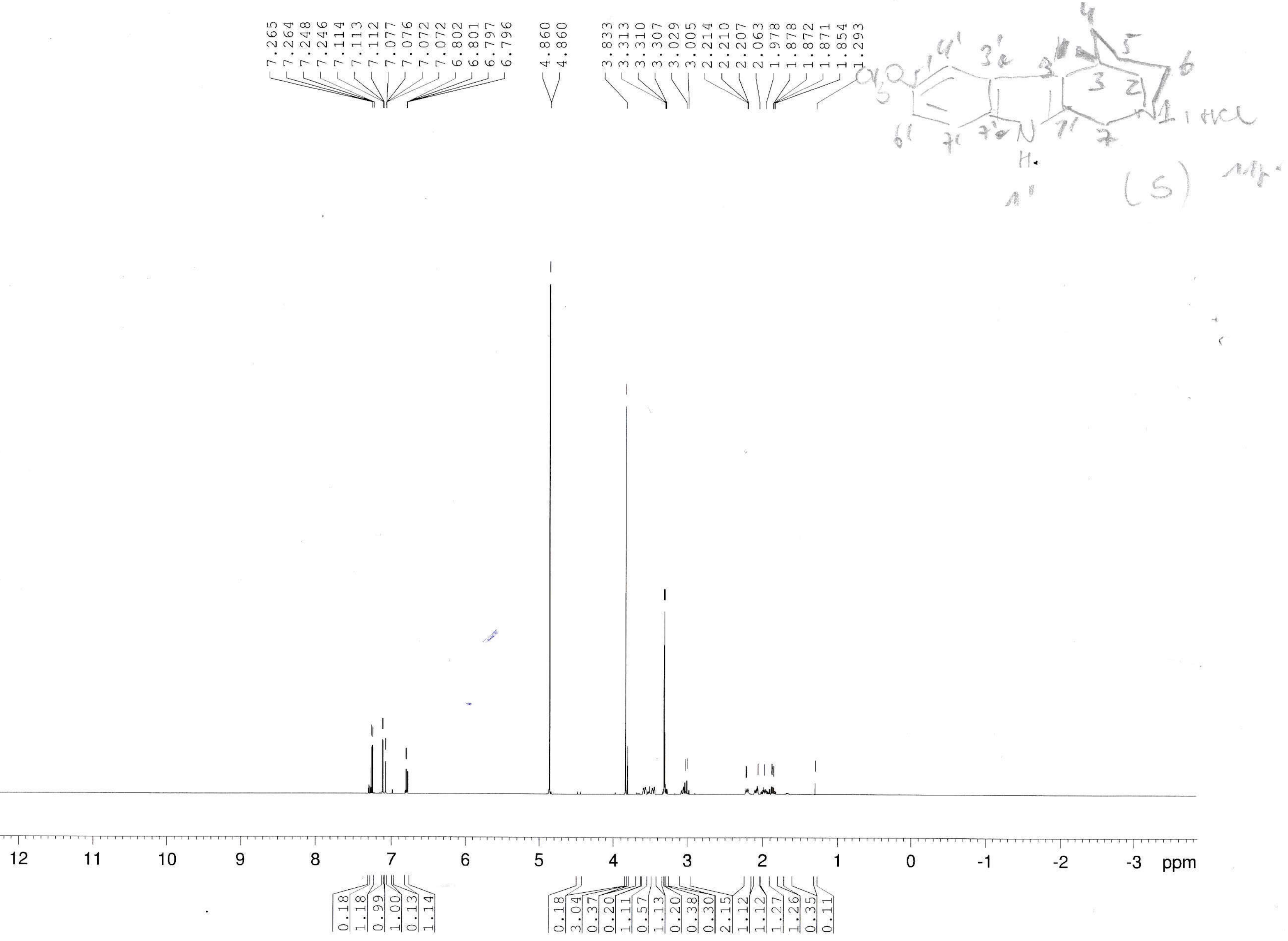

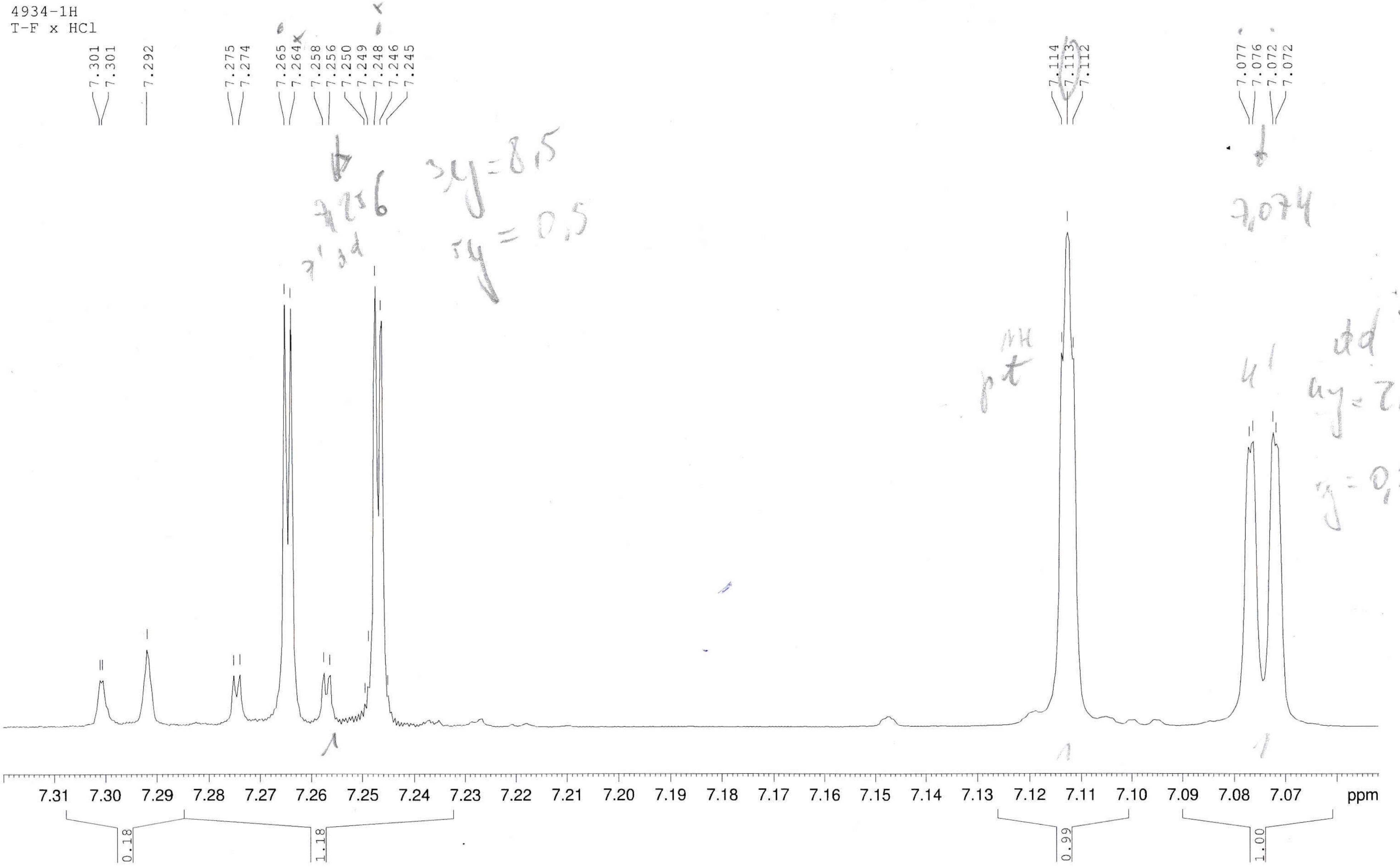
4934-1H
T-F x HCl
7.301
7.301
7.292
7.275
7.274
7.265
7.264
7.258
7.256
7.250
7.249
7.248
7.246
7.245
7.114
7.113
7.112
7.077
7.076
7.072
7.072
7.31 7.30 7.29 7.28 7.27 7.26 7.25 7.24 7.23 7.22 7.21 7.20 7.19 7.18 7.17 7.16 7.15 7.14 7.13 7.12 7.11 7.10 7.09 7.08 7.07 ppm
0.18
1.18
0.99
1.00

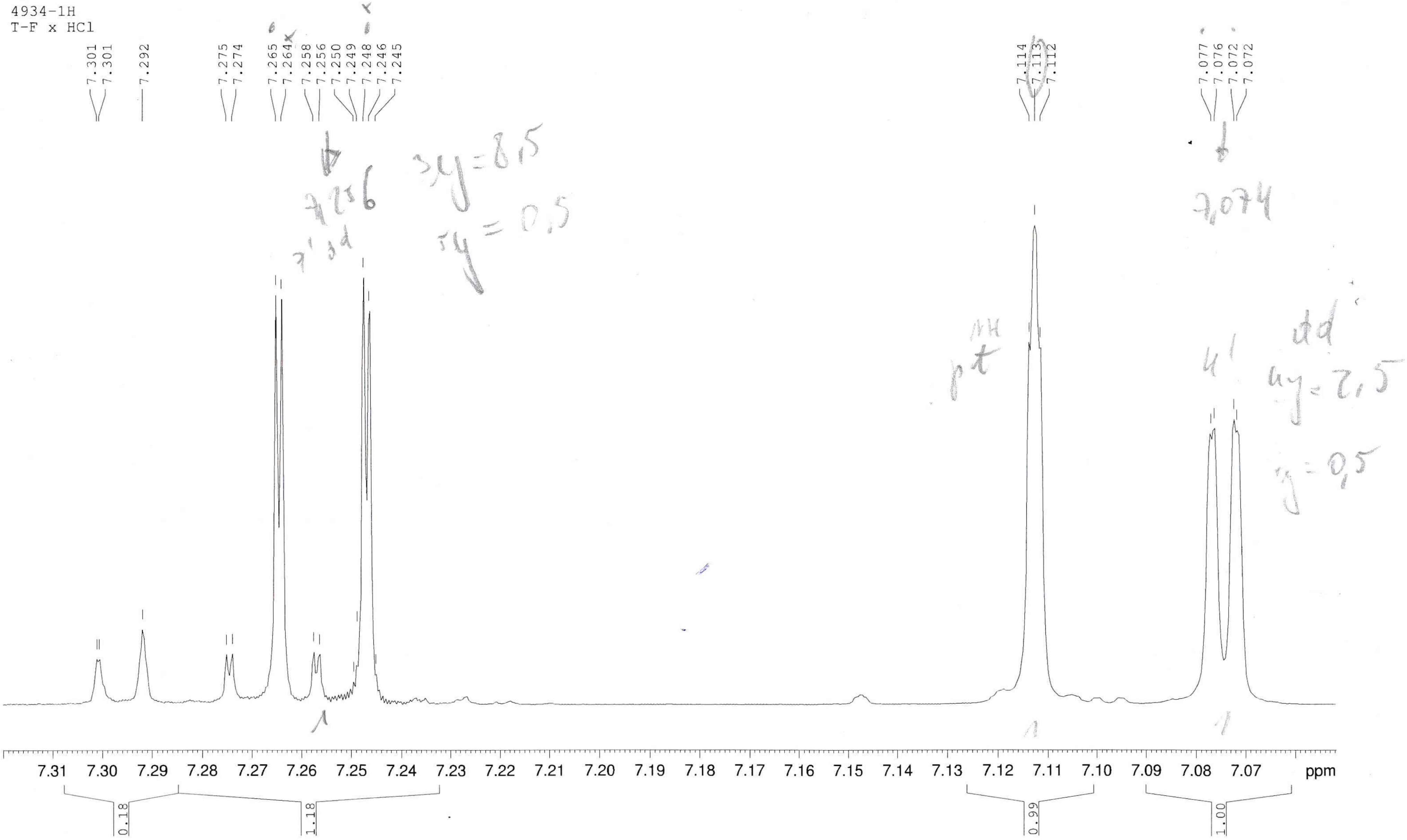
4934-1H
T-F x HCl
7.301
7.301
7.292
7.275
7.274
7.265
7.264
7.258
7.256
7.250
7.249
7.248
7.246
7.245
7.114
7.113
7.112
7.077
7.076
7.072
7.072
7.31 7.30 7.29 7.28 7.27 7.26 7.25 7.24 7.23 7.22 7.21 7.20 7.19 7.18 7.17 7.16 7.15 7.14 7.13 7.12 7.11 7.10 7.09 7.08 7.07 ppm
0.18
1.18
0.99
1.00

4934-1H
T-F x HCl

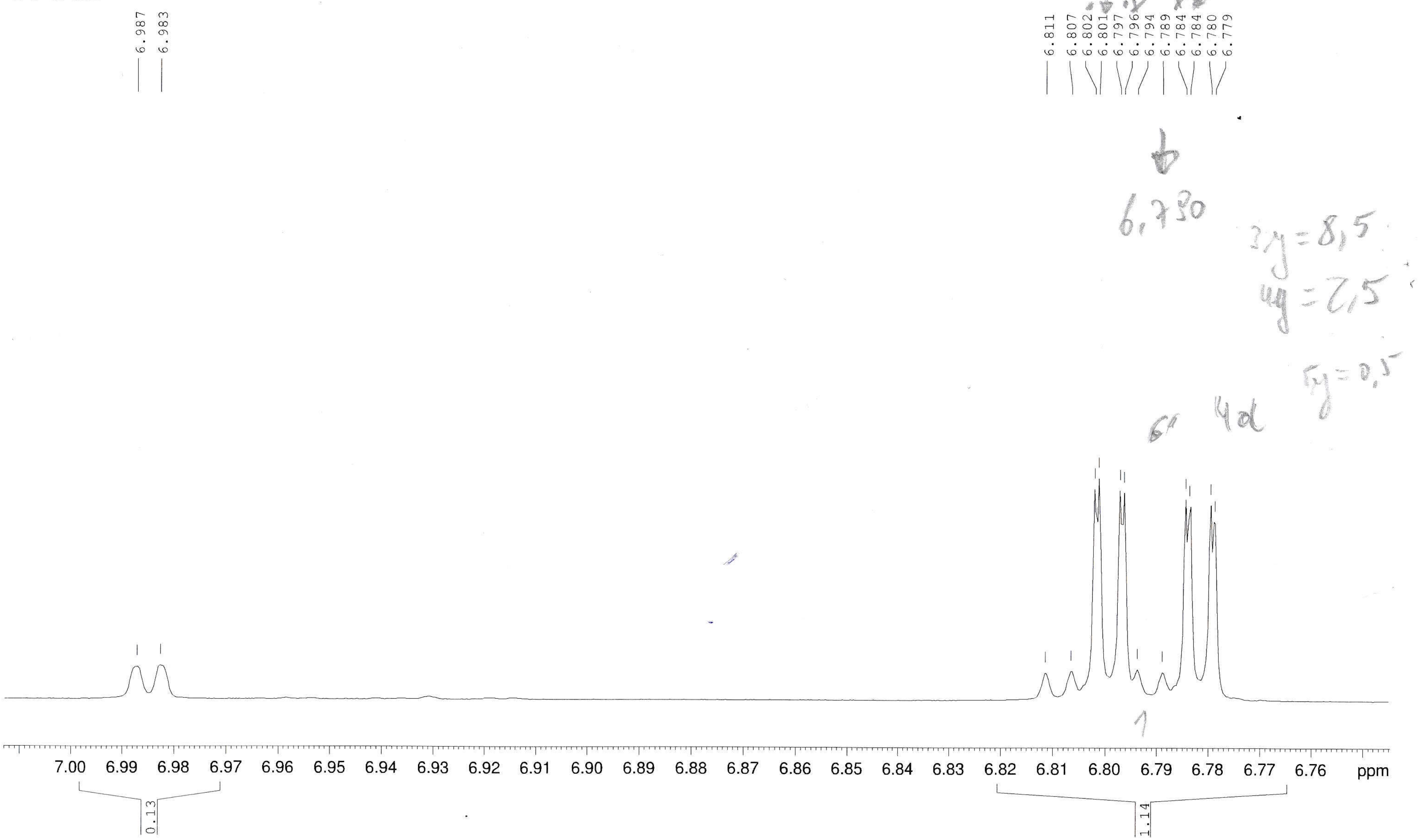

4934-1H
T-F x HCl

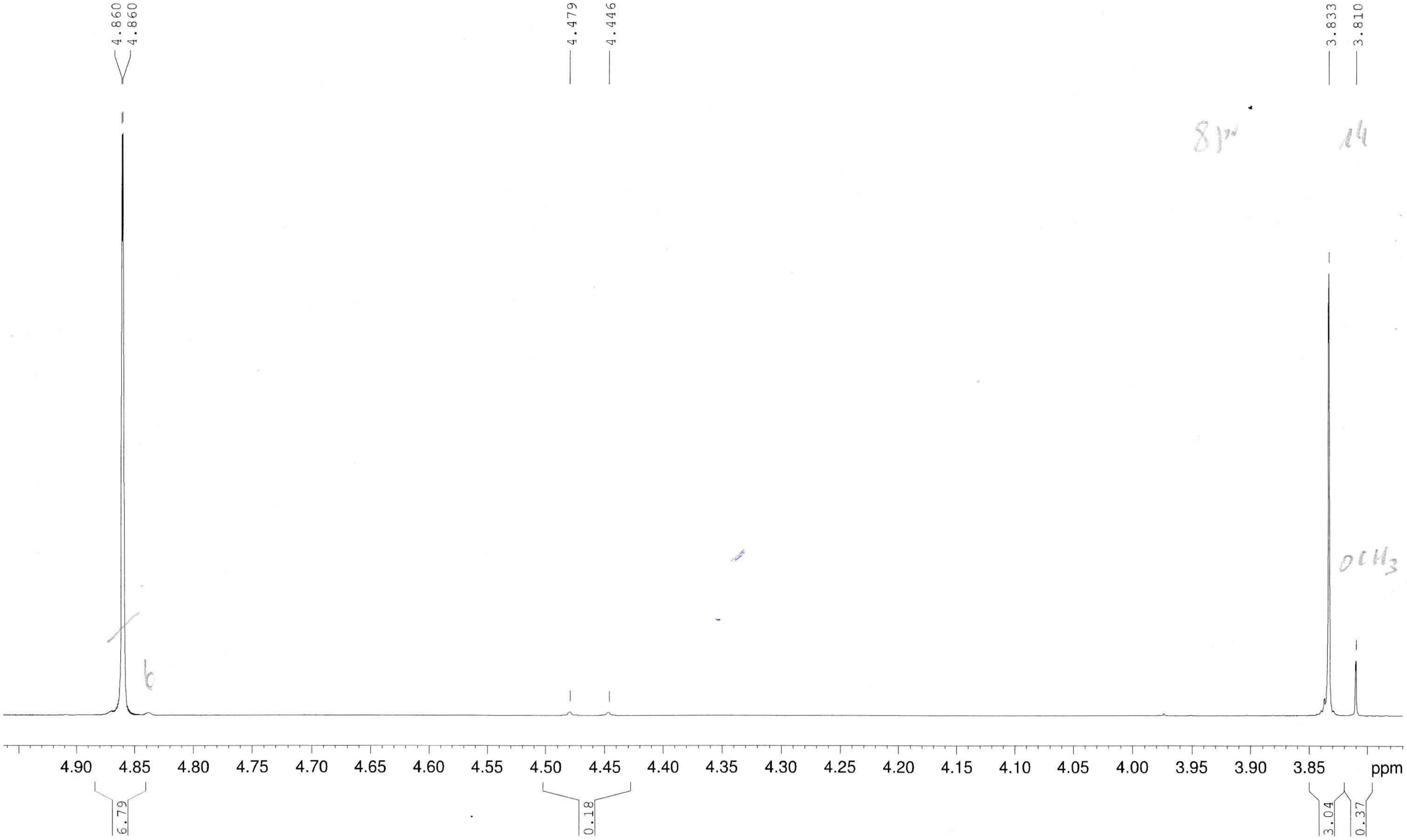

4934-1H
T-F x HCl

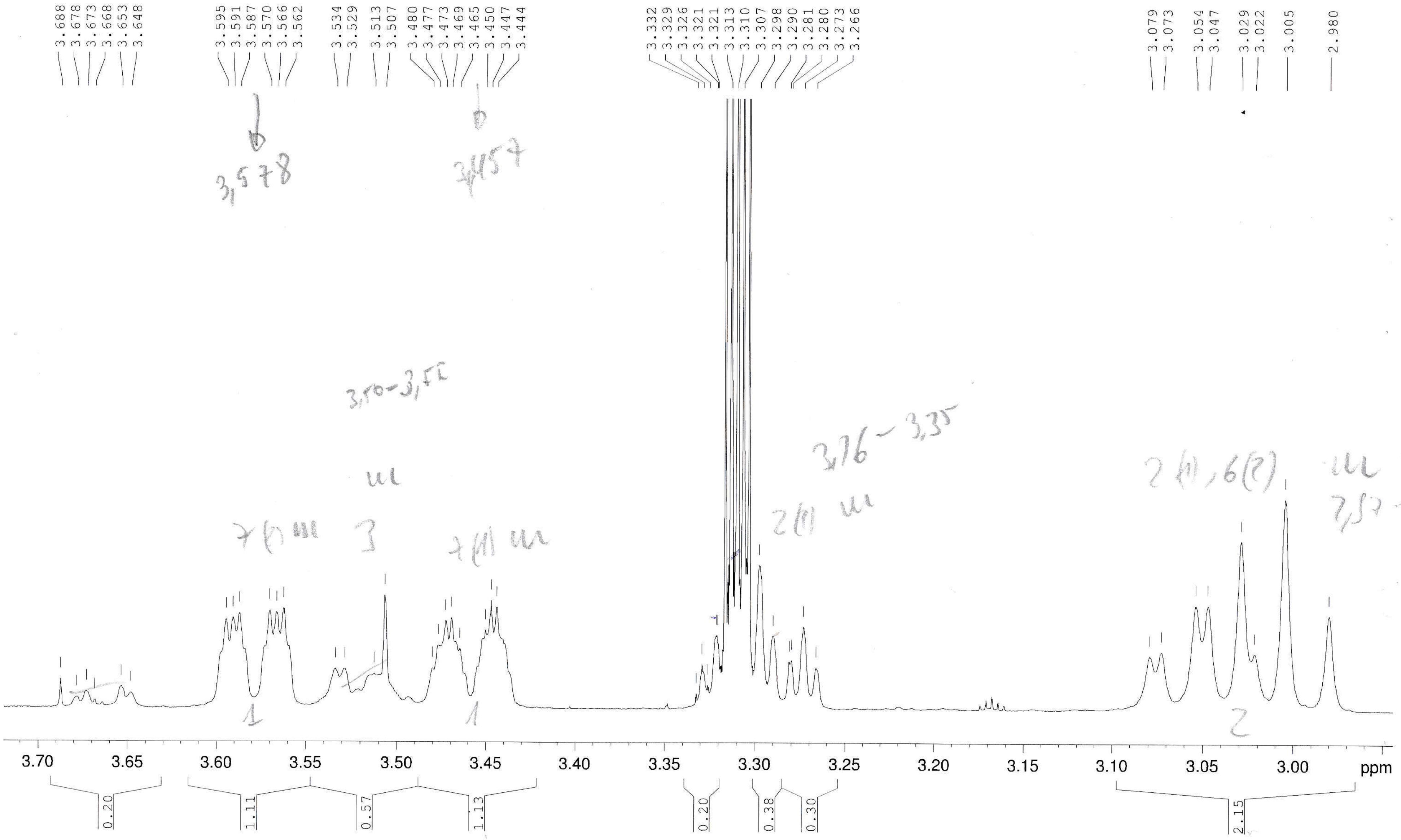

4934-1H
T-F x HCl

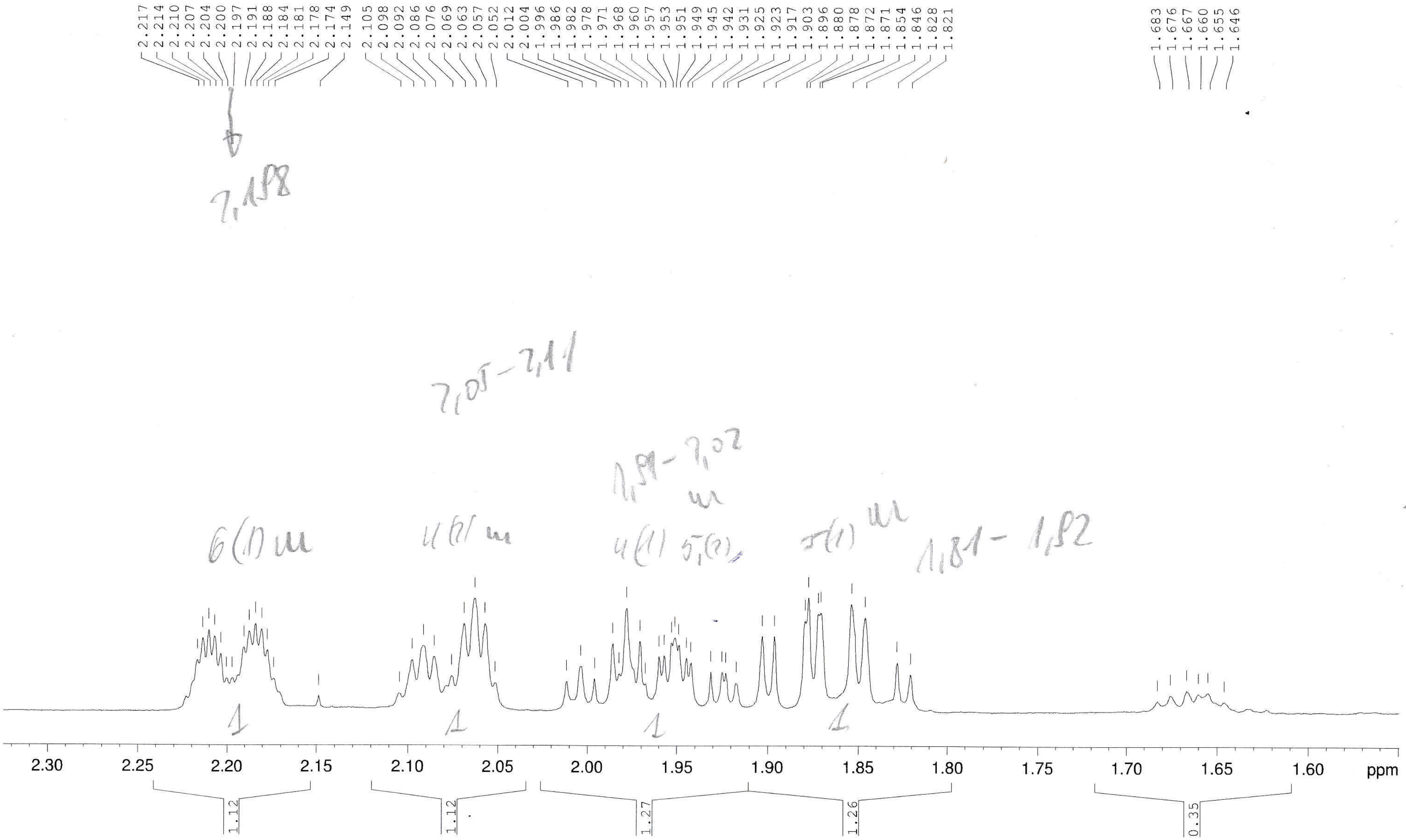

4934-13C
T-F x HCl

Current Data Parameters
NAME 4934
EXPNO 2
PROCNO 1

F2 - Acquisition Parameters
Date_ 20180529
PROBHD 5 mm CPPBBO BB
PULPROG zg_pi_CPD
SOLVENT MeOD
TE 298.2 K
SWH 31249.381 Hz
AQ 1.0485969 sec
NS 376
FIDRES 0.476828 Hz
D1 1.50000000 sec
D11 0.03000000 sec
D14 0.00050000 sec

======== CHANNEL f1 ========
SFO1 125.7904831 MHz
NUC1 13C
P1 10.00 usec
P2 20.00 usec
PLW1 55.00000000 W

======== CHANNEL f2 ========
SFO2 500.2020008 MHz
NUC2 1H
PLW2 13.00000000 W
PLW12 0.25937000 W

F2 - Processing parameters
SI 65536
LB 0.50 Hz

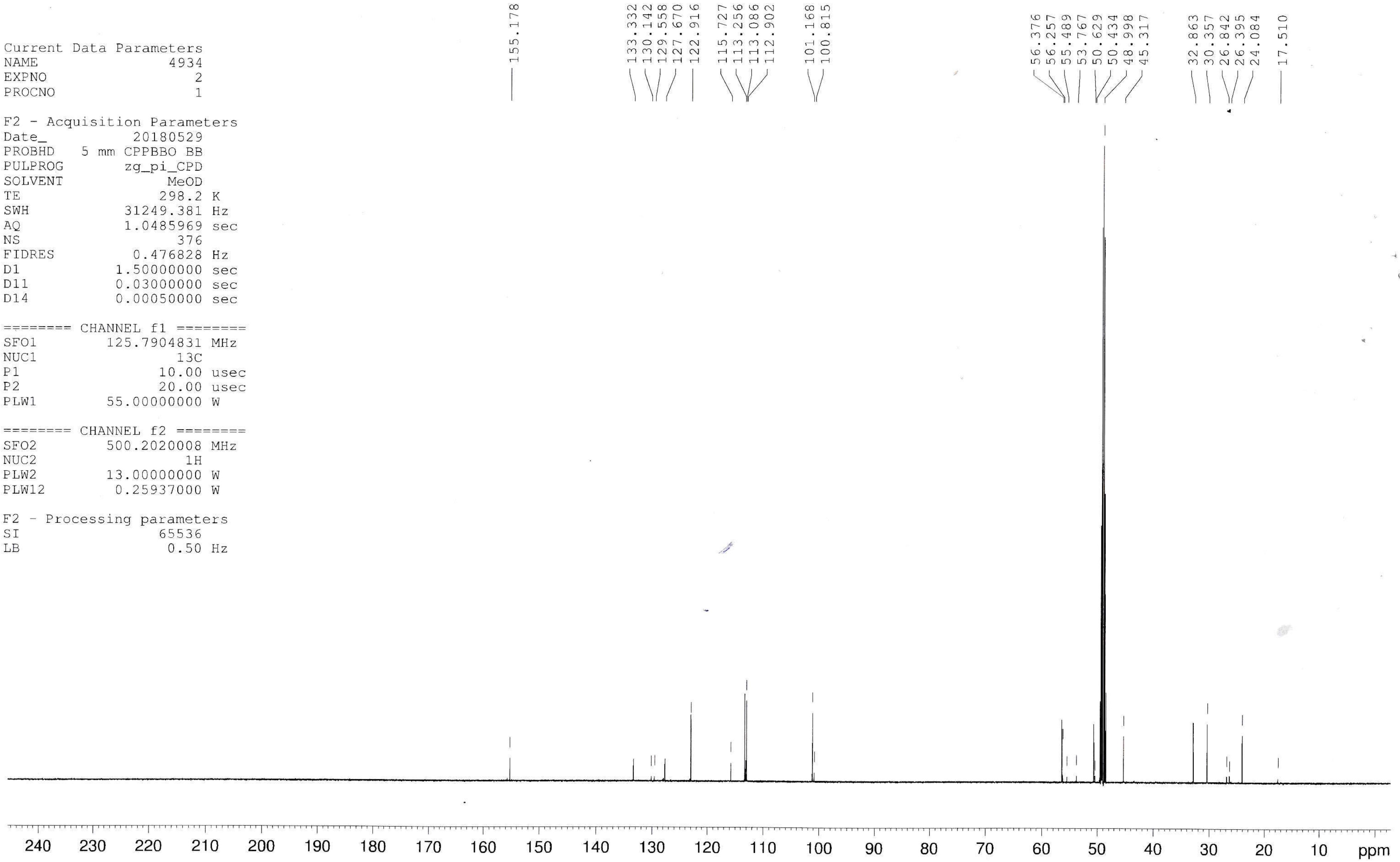

4934-13C
T-F x HCl

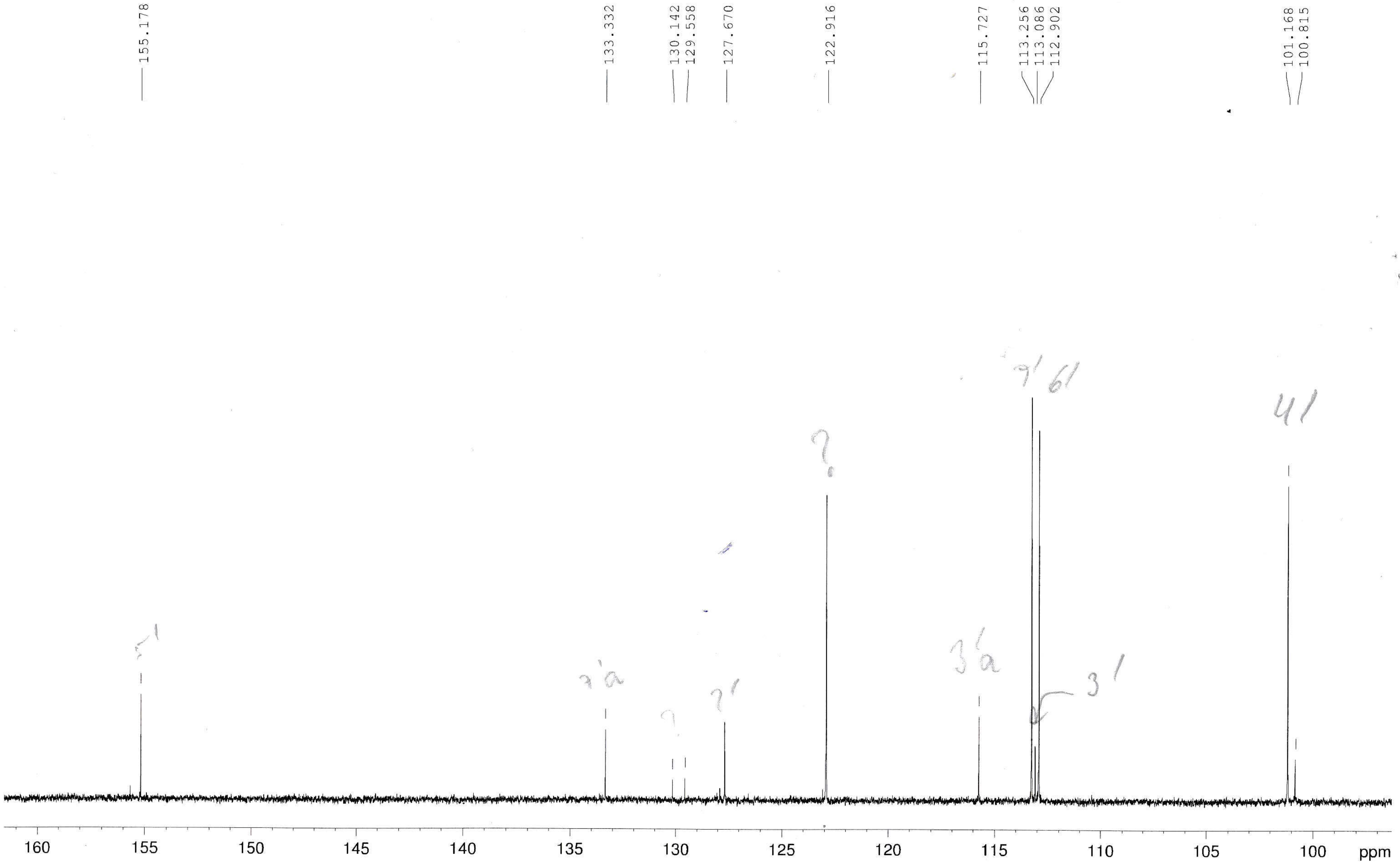

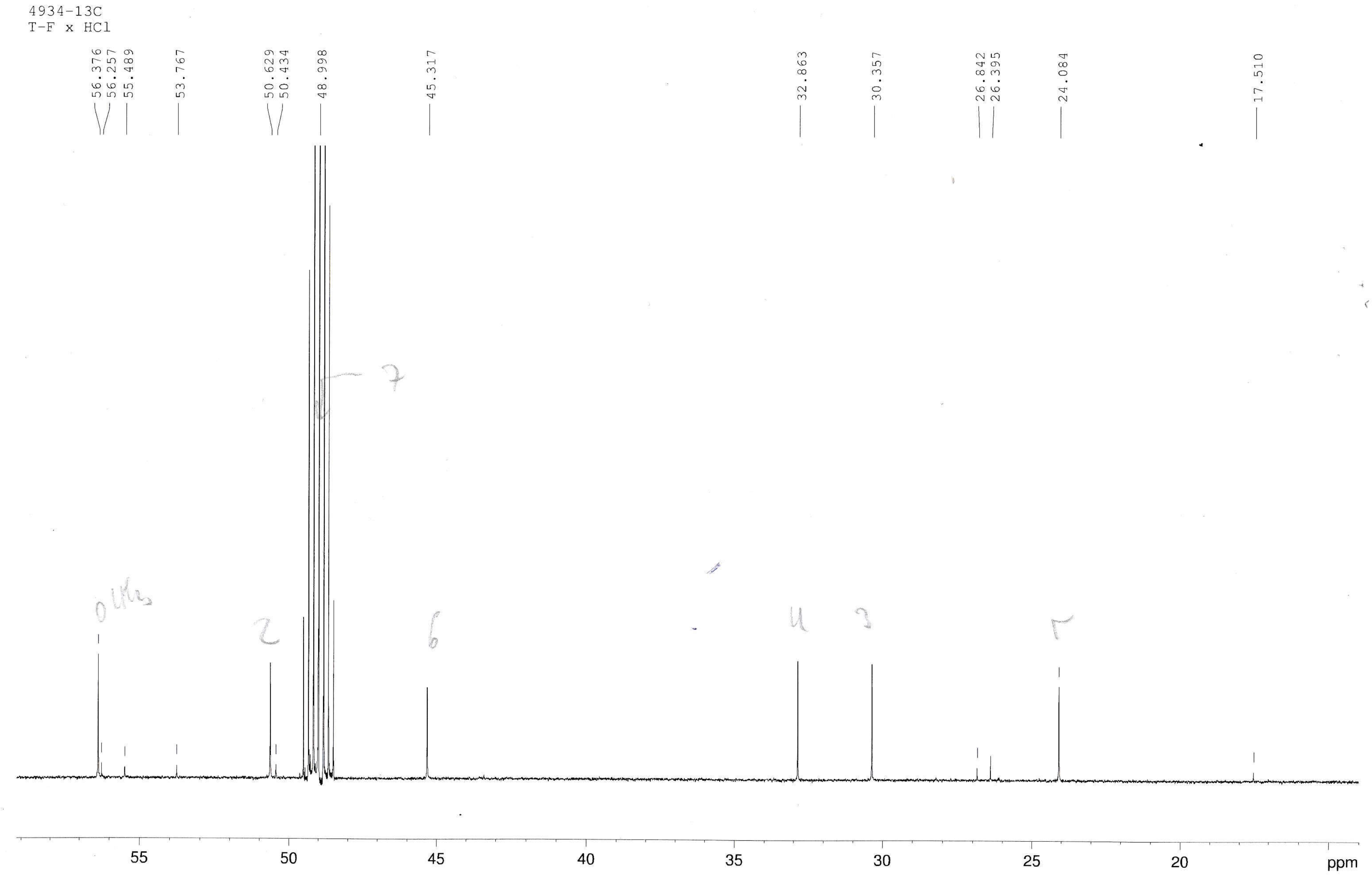
4934-13C
T-F x HCl
56.376
56.257
55.489
53.767
50.629
50.434
48.998
45.317
32.863
30.357
26.842
26.395
24.084
17.510
55
50
45
40
35
30
25
20
ppm
